# Stereoselective Synthesis of Axially Chiral 5,5′-Linked *bis*-1-Arylisochromans with Antibacterial Activity

**DOI:** 10.3390/ijms26167777

**Published:** 2025-08-12

**Authors:** Zoltán Czenke, Attila Mándi, Gergely Miklós Fedics, Roland Albert Barta, Attila Kiss-Szikszai, Anna Kurucz-Szabados, István Timári, Attila Bényei, Sándor Balázs Király, Eszter Ostorházi, Changsheng Zhang, Máté Kicsák, Tibor Kurtán

**Affiliations:** 1 Department of Organic Chemistry, University of Debrecen, P.O. Box 400, 4002 Debrecen, Hungary; czenke.zoltan@science.unideb.hu (Z.C.); mandi.attila@science.unideb.hu (A.M.); kiss.attila@science.unideb.hu (A.K.-S.); timari.istvan@science.unideb.hu (I.T.); kiraly.sandor.balazs@science.unideb.hu (S.B.K.); 2 Doctoral School of Chemistry, University of Debrecen, Egyetem tér 1., 4032 Debrecen, Hungary; 3 Department of Physical Chemistry, University of Debrecen, Egyetem tér 1., 4032 Debrecen, Hungary; benyei.attila@science.unideb.hu; 4 Department of Medical Microbiology, Semmelweis University, 1085 Budapest, Hungary; droeszter@gmail.com; 5 Key Laboratory of Tropical Marine Bio-Resources and Ecology, Guangdong Key Laboratory of Marine Materia Medica, Institutions of South China Sea Ecology and Environmental Engineering, South China Sea Institute of Oceanology, Chinese Academy of Sciences, Guangzhou 510301, China; czhang@scsio.ac.cn

**Keywords:** heterodimeric biaryl-type axially chiral *bis*-isochromans, stereoselective Suzuki–Miyaura cross-coupling, antibacterial, vibrational circular dichroism, chiral induction from central to axial, stereoisomeric isochromans with central and axial chirality, axially chiral *ortho*-quinones

## Abstract

Inspired by naturally occurring *bis*-isochromans such as penicisteckins, we envisaged the first synthesis of biaryl-type *bis*-1-arylisochromans containing a stereogenic *ortho*-trisubstituted biaryl axis. We achieved the stereoselective synthesis of 5,5′-linked heterodimeric *bis*-isochromans containing both central and axial chirality elements by performing diastereoselective Suzuki–Miyaura biaryl coupling reactions on two optically active 1-arylpropan-2-ol derivatives, followed by two oxa-Pictet–Spengler cyclizations with aryl aldehydes or methoxymethyl chloride. We studied the diastereoselectivity of the cyclization step, separated the stereoisomeric products with chiral preparative HPLC and determined the absolute configuration through a combination of vibrational circular dichroism (VCD), NMR and single-crystal X-ray diffraction analysis. We demonstrated that different aryl groups could be introduced into the two isochroman subunits, since the dimethoxyaryl subunit reacted faster, enabling the two oxa-Pictet–Spengler cyclizations to be performed separately with different aryl aldehydes. We also explored the acid-catalyzed isomerization and oxidation to axially chiral *ortho*-quinones in order to produce stereoisomeric and oxidized analogs, respectively. We identified the antibacterial activity of our target *bis*-isochromans against *Bacillus subtilis* and *Enterococcus faecalis* with minimum inhibitory concentrations down to 4.0 and 0.5 μg/mL, respectively, which depend on the stereochemistry and substitution pattern of the *bis*-isochroman skeleton.

## 1. Introduction

**Scheme 1:**
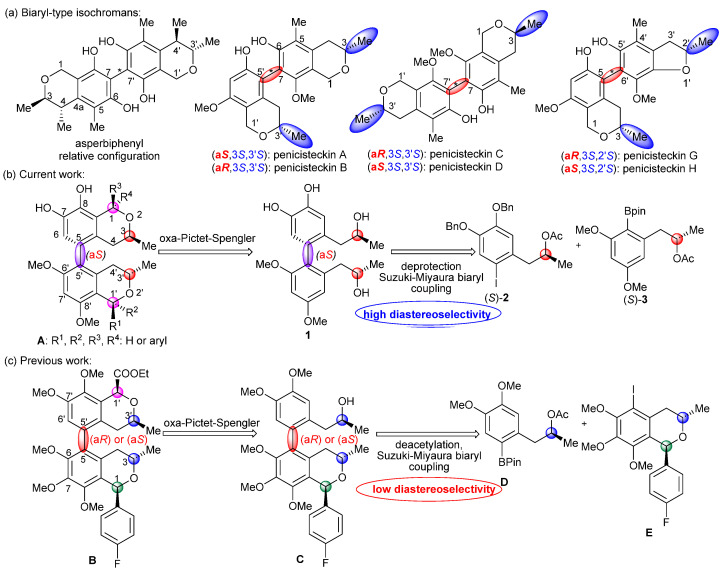
(**a**) Structures of biaryl-type axially chiral natural isochroman derivatives. (**b**) Retrosynthetic scheme for the preparation of axially chiral heterodimeric *bis*-isochromans. (**c**) Retrosynthetic scheme for the reported biaryl coupling between a 1-arylisochroman and a 1-arylpropan-2-ol derivative producing low atropodiastereoselectivity.

Isochroman (3,4-dihydro-1*H*-2-benzopyran) derivatives represent a subgroup of benzene-condensed *O*-heterocyclic derivatives, and their natural representatives often contain hydroxyl, alkoxy or alkyl functional groups on the fused aromatic ring or at the C-1, C-3, C-4 positions of the heteroring. Several optically active substituted isochromans of natural or synthetic origin exhibit remarkable bioactivity [1] such as central nervous system activity [2,3,4,5,6], antioxidant [7,8,9], antibacterial [10], antifungal [11], antihypertensive [12], antineoplastic/cytotoxic [13] and anti-inflammatory [14] activity. More specifically, 1-arylisochromans containing phenolic hydroxyl groups were reported as natural products of olive with antioxidant, anti-inflammatory, and neuroprotective activity [8,9,15]. The condensed benzene ring of natural isochromans often contains activating substituents, such as hydroxyl or alkoxy, that can facilitate oxidative biaryl coupling reactions to form biaryl-type *bis*-isochroman derivatives. However, only two papers have reported natural *bis*-isochromans with a stereogenic biaryl axis [16,17]. The homodimeric 7,7′-linked antiviral asperbiphenyl, the first axially chiral *bis*-isochroman, isolated from the marine fungus *Aspergillus* sp. contains an *ortho*-tetrasubstituted stereogenic biaryl axis and four chirality centers (Scheme 1a) [16]. Penicisteckins A–D, two pairs of atropodiastereomeric biaryl-type hetero- and homodimeric *bis*-isochromans with 7,5*′*- and 7,7*′*-linkages, were isolated from *Penicillium steckii* HNNU-5B18 [17]. Recently, penicisteckins G and H, axially chiral biaryl-type isochroman-dihydrobenzo[*b*]furan dimers with antibacterial activity, have been reported from the marine coral-derived fungus *Penicillium steckii* SCISO 41228 [18].

Inspired by natural *bis*-isochromans, we developed a stereoselective synthetic strategy for 5,5′-linked heterodimeric *bis*-1-arylisochromans **A**, as shown in the retrosynthetic scheme of Scheme 1b. In the final step of the sequence, we prepared the isochroman moieties of the target molecules **A** by using two oxa-Pictet–Spengler cyclizations on the biaryl precursor **1**. Precursor **1** was produced via diastereoselective Suzuki–Miyaura biaryl coupling reactions of the optically active 1-arylpropan-2-ol derivatives (*S*)-**2** and (*S*)-**3**, followed by the removal of the protecting groups. In our previous work [19], we had synthesized axially chiral *bis*-isochromans **B** via the Suzuki–Miyaura biaryl coupling reactions using the optically active 1-arylpropan-2-ol derivative **D** and the 1-arylisochroman **E**. The biaryl coupling of **D** and **E** had low atropodiastereoselectivity (*dr* 63:37), while in the current work, more efficient central-to-axial chirality induction occurred with 1-arylpropan-2-ol derivatives (*S*)-**2** and (*S*)-**3**, resulting in a high diastereomeric ratio (*dr* 95:5). In addition, the dimethoxy substitution of the upper aryl moiety in precursor **C** did not enable the oxa-Pictet–Spengler cyclization with aryl aldehyde derivatives and thus *bis*-1-arylisochromans were not accessible in this sequence. In contrast, the two aryl moieties of precursor **1** of the current sequence promoted the cyclization with aryl aldehydes, which allowed us to efficiently prepare 5,5′-linked *bis*-1-arylisochromans **A** with different absolute configurations at C-1 and C-1′. We also demonstrated that the lower aryl moiety of precursor **1** was more reactive in the oxa-Pictet–Spengler cyclization. This allowed us to cyclize first the lower part and use a different aryl aldehyde for the cyclization of the upper aryl moiety. Six single-crystal X-ray geometries of the target compounds aided the structural elucidation together with 2D NMR measurements. Some of the axially chiral *bis*-isochroman products **A** showed antibacterial activity against *B. subtilis* and *E. faecalis* with minimum inhibitory concentrations down to 4.0 and 0.5 μg/mL, respectively.

## 2. Results and Discussion

**Scheme 2:**
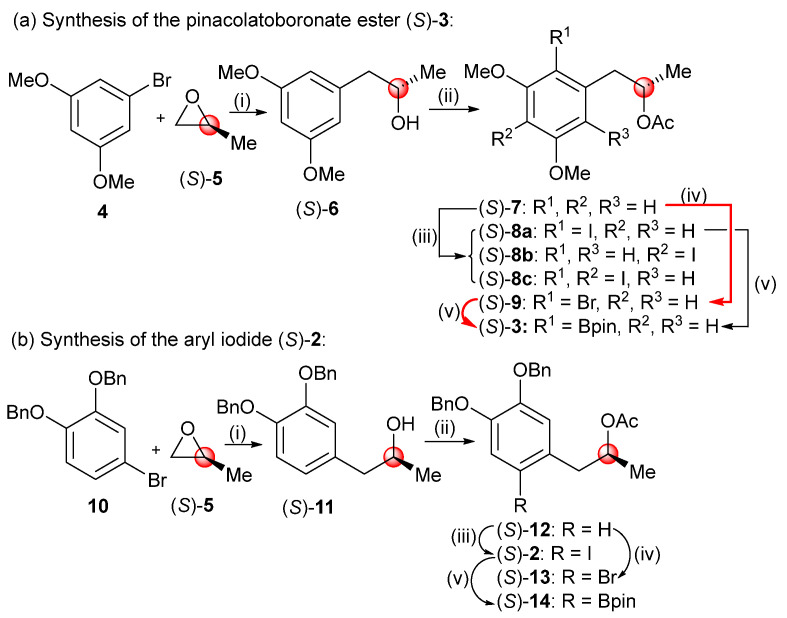
Synthesis of the cross-coupling partners for the Suzuki–Miyaura reaction. Reagents and conditions: (i) (a) *n*-BuLi, Ar/N_2_, THF, −80 °C, 20 min, (b) (*S*)-propylene oxide [(*S*)-**5**], Ar/N_2_, −78 °C, 20 min, (c) BF_3_.Et_2_O, Ar/N_2_, −80 °C, 30 min, (*S*)-**6** (86%), (*S*)-**11** (90%); (ii) AcCl, C_5_H_5_N, CH_2_Cl_2_, rt, 3 h, (*S*)-**7** (91%), 3 h, (*S*)-**12** (82%); (iii) NIS, F_3_CCOOH, MeCN, rt, 22 h, (*S*)-**8a**–**c**, 16 h, (*S*)-**2** (96%); (iv) NBS, MeCN, rt, 16 h, (*S*)-**9** (94%), 16 h, (*S*)-**13** (97%); (v) (a) (Ph_3_P)_2_PdCl_2_, PPh_3_, KOAc, Ar/N_2_, DMF, rt, 15 min, (b) B_2_pin_2_ [*bis*(pinacolato)diboron], Ar/N_2_, 150 °C, 2 h, (*S*)-**3** (from (*S*)-**8a**: 35%, from (*S*)-**9**: 62%), 3 h, (*S*)-**14** (92%).

The optically active coupling partners of the Suzuki–Miyaura biaryl cross-coupling reactions, the pinacolatoboronate ester (*S*)-**3** and the aryl iodide derivative (*S*)-**2** were prepared in short sequences starting from the corresponding aryl bromide derivatives **4** or **10** and (*S*)-propylene oxide [(*S*)-**5**] (Scheme 2).

In the first step, the aryl lithium reagents, formed in situ in the reaction of 1-bromo-3,5-dimethoxybenzene (**4**) or 4-bromo-1,2-dibenzyloxybenzene (**10**) with *n*-butyllithium, opened the epoxide ring regioselectively producing the (*S*)-1-arylpropan-2-ol derivatives (*S*)-**6** and (*S*)-**11**, respectively. Acetylation of (*S*)-**6** and (*S*)-**11** followed by halogenation with *N*-halosuccinimide afforded the corresponding aryl halides. When using *N*-iodosuccinimide (NIS) in the reaction of (*S*)-**7**, an inseparable mixture of the monoiodo regioisomers (*S*)-**8a**,**b** and the diiodo derivative (*S*)-**8c** was formed. Thus, the Miyaura borylation of a mixture of monoiodo derivatives (*S*)-**8a**,**b** resulted in the desired boronate ester (*S*)-**3** with a low yield of 35%, which was also heightened by a dehalogenation side-reaction. To improve the yield and avoid working with regioisomers, the bromination of (*S*)-**7** was carried out with *N*-bromosuccinimide (NBS), which occurred regioselectively, and (*S*)-**9** was isolated with 94% yield. Then, the Miyaura borylation of (*S*)-**9** produced the pinacolatoboronate ester (*S*)-**3** with 68% yield (Scheme 2). On the other hand, both the iodination and bromination of (*S*)-**12** could be carried out regioselectively to obtain the iodo and bromo derivatives (*S*)-**2** and (*S*)-**13**, respectively, with high yields. The iodo derivatives (*S*)-**2** was also converted to the pinacolatoboronate ester (*S*)-**14**. However, since we could not synthesize (*S*)-**8a** regioselectively with high yield, we did not use (*S*)-**14** for the coupling reactions.

**Scheme 3:**
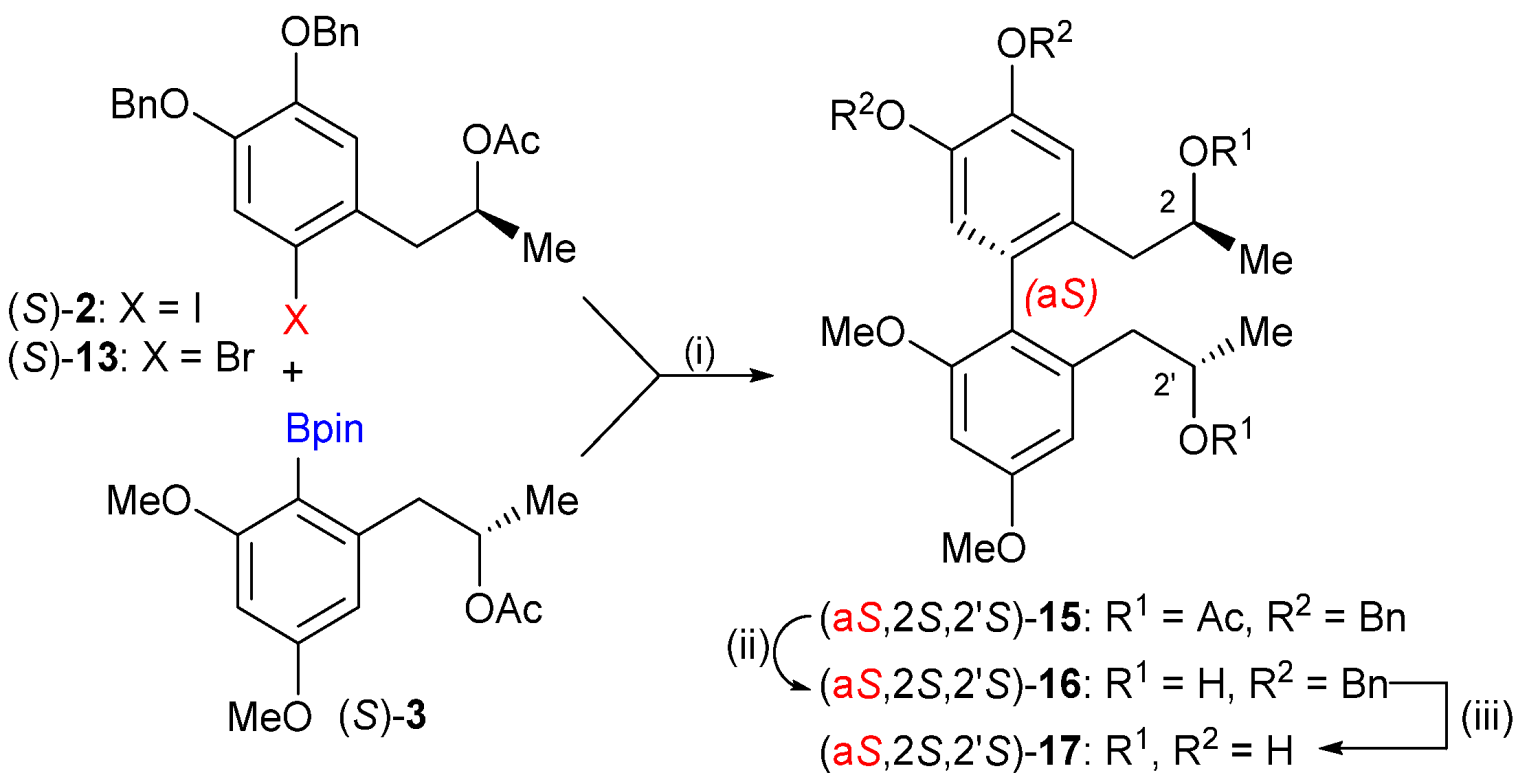
Suzuki cross-coupling reactions of (*S*)-**2** and (*S*)-**3**, and subsequent removal of the protecting groups. Reagents and conditions: (i) (a) (*S*)-**2**, phosphine ligand, Pd(OAc)_2_, Ar/N_2_, DMF, rt, 1 h, (b) (*S*)-**3**, CsF, Ar/N_2_, DMF, rt 30 min, (c) a + b, Ar/N_2_, 150 °C, 2 h; (ii) LiOH, MeOH, rt, 1.5 h, (a*S*,2*S*,2′*S*)-**16** (85%); (iii) H_2_, Pd(C), THF, rt, 6 h, (a*S*,2*S*,2′*S*)-**17** (97%); (a*S*) *de* > 90%. Bpin: pinacolboryl.

In the Suzuki biaryl coupling reaction, we tested (*S*)-**3** as the pinacolatoboronate ester component and (*S*)-**13** and (*S*)-**2** as the aryl halide (Table 1, Scheme 3). The bromo derivative (*S*)-**13** was not reactive enough under the applied conditions (entry 1) and only traces of the product were observed. Coupling (*S*)-**3** with (*S*)-**2** using Pd(OAc)_2_ and various phosphine ligands such as PPh_3_ (36%), XPhos (2-dicyclohexylphosphino-2′,4′,6′-triisopropylbiphenyl, 47%) and Xantphos [4,5-*bis*(diphenylphosphino)-9,9-dimethylxanthene, 63%] produced the axially chiral biaryl (a*S*,2*S*,2′*S*)-**15** with high atropodiastereoselectivity (*dr* > 95:5), favoring the (a*S*) atropodiastereomer (Table 1, entries 2–4). Due to the *ortho*-trisubstituated biaryl axis, the rotation is hindered along the stereogenic biaryl axis, giving rise to a high rotational energy barrier (∆G^‡^_300K_ >> 93,5 kJ mol^−1^) and hence axial chirality [20].

**Scheme 4:**
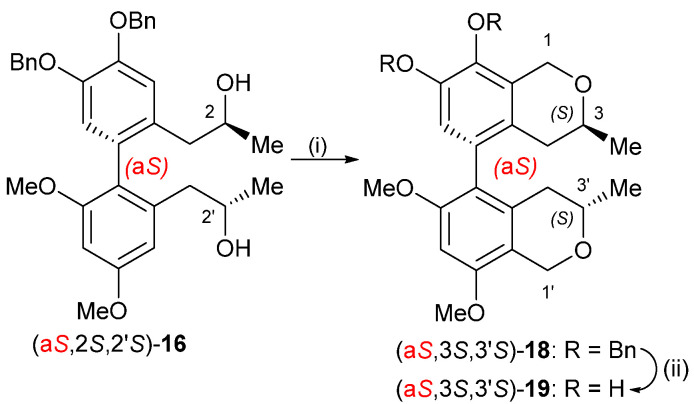
Oxa-Pictet–Spengler cyclization reaction of (a*S*,2*S*,2′*S*)-**16** with MOMCl and subsequent removal of the benzyl protecting groups. Reagents and conditions: (i) MOMCl, ZnCl_2_, THF, 0 °C → rt, 20 h, (a*S*,3*S*,3′*S*)-**18** (42%); (ii) H_2_, Pd(C), THF, rt, 3 h, (a*S*,3*S*,3′*S*)-**19** (80%); (a*S*) *de* > 90%.

After removing the acetyl groups with LiOH to get (a*S*,2*S*,2′*S*)-**16** from (a*S*,2*S*,2′*S*)-**15** (Scheme 3), we performed an oxa-Pictet–Spengler cyclization using methoxymethyl chloride (MOMCl) and ZnCl_2_ to produce the isochroman derivative (a*S*,3*S*,3′*S*)-**18**, containing methylene groups at C-1 and C-1′ (Scheme 4).

**Figure 1:**
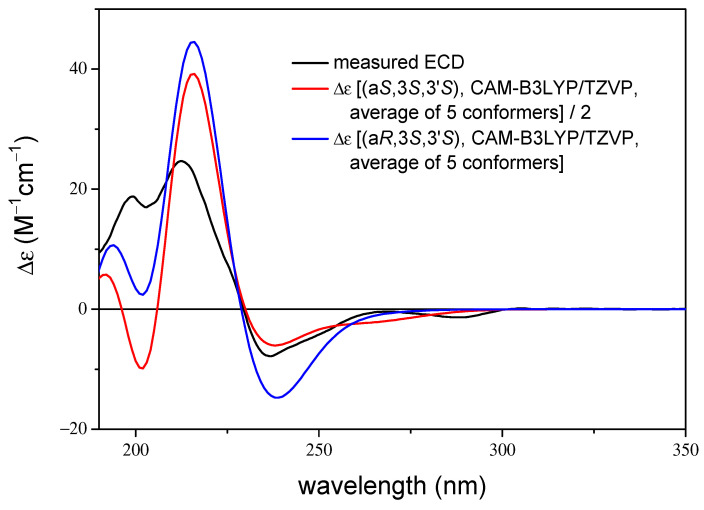
Comparison of the experimental ECD spectrum of (a*S*,3*S*,3′*S*)-**19** (black) with the CAM-B3LYP/TZVP PCM/MeCN**//**ωB97X/TZVP PCM/MeCN average ECD spectra of (a*S*,3*S*,3′*S*)-**19** (red) and (a*R*,3*S*,3′*S*)-**19** (blue).

Subsequent catalytic hydrogenation removed the two benzyl groups, affording the target compound (a*S*,3*S*,3′*S*)-**19**. To determine the axial chirality (a*S*,3*S*,3′*S*)-**19** and its precursors, we measured and computed the ECD (electronic circular dichroism) and VCD spectra. The experimental ECD spectrum of (a*S*,3*S*,3′*S*)-**19** showed two weak negative Cotton effects (CEs) above 230 nm and two more intense positive ones in the 190–230 nm range (Figure 1).

**Figure 2:**
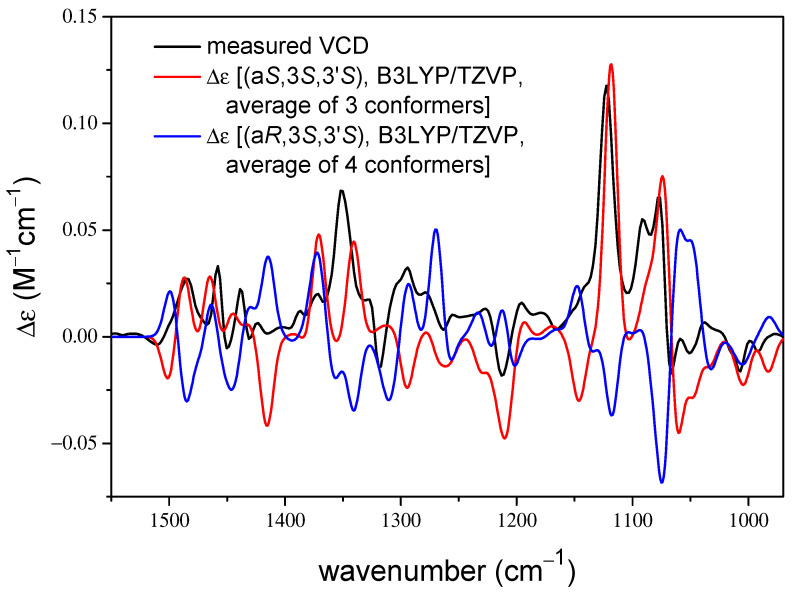
Comparison of the experimental VCD spectrum of (a*S*,3*S*,3′*S*)-**19** (black) with the B3LYP/TZVP PCM/CHCl_3_//B3LYP/TZVP PCM/CHCl_3_ average VCD spectra of (a*S*,3*S*,3′*S*)-**19** (red) and (a*R*,3*S*,3′*S*)-**19** (blue).

The computed CAM-B3LYP/TZVP PCM/MeCN ECD spectra of (a*S*,3*S*,3′*S*)-**19** and (a*R*,3*S*,3′*S*)-**19** were quite similar, reproducing well the three high-wavelength CEs observed in the experimental ECD spectrum. The only difference manifested in a negative computed CE for (a*S*,3*S*,3′*S*)-**19**, which was absent from the experimental and the computed ECDs of (a*R*,3*S*,3′*S*)-**19**. This suggested a tentative assignment of axial chirality as (a*R*), which would have been incorrect. Thus ECD spectra did not contain relevant information about the axial chirality; rather, it depended on the central chirality elements. Unlike the computed ECD spectra, the B3LYP/TZVP PCM/CHCl_3_ VCD spectra of the (a*S*,3*S*,3′*S*)-**19** and (a*R*,3*S*,3′*S*)-**19** had many near mirror image VCD transitions, which reflected well the different axial chirality of the atropodiastereomers (Figure 2). Comparing the experimental VCD spectrum with the experimental ones allowed determining the axial chirality of **19** as (a*S*), specifically by using the intense VCD transitions in the 1050–1150 cm^–1^ range. The ECD and VCD calculations of (a*S*,3*S*,3′*S*)-**19** and (a*R*,3*S*,3′*S*)-**19** revealed that the ECD spectra do not reflect the axial chirality, while characteristic VCD transitions can be utilized to determine the axial chirality unambiguously. This contradicts the ECD spectra of penicisteckins′ atropodiastereomers [17,19] in which the different substitution pattern of the condensed benzene ring resulted in near mirror-image experimental ECD spectra for the isolated atropodiastereomers.

**Scheme 5:**
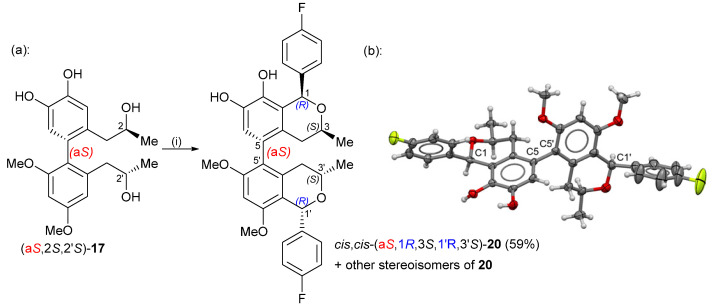
(**a**) Oxa-Pictet–Spengler cyclization of (a*S*,2*S*,2′*S*)-**17** with 4-fluorobenzaldehyde. Reagents and conditions: (i) 4-fluorobenzaldehyde (6 eq.), (+)-camphorsulfonic acid (1 eq.), toluene–MeOH 4:1, 80 °C. (**b**) ORTEP view of *cis*,*cis*-(a*S*,1*R*,3*S*,1′*R*,3′*S*)-**20** at 50% probability level (CCDC deposition no.: 2467523). Only one molecule from the asymmetric unit is shown. Single crystals were produced from CHCl_3_/MeOH 4:1.

To facilitate the oxa-Pictet–Spengler cyclization of our biaryl precursor with various aryl aldehydes, we removed the two benzyl protecting groups of (a*S*,2*S*,2′*S*)-**16** via catalytic hydrogenation to get (a*S*,2*S*,2′*S*)-**17** (Scheme 3), containing two activating hydroxyl groups. Then, we performed the acid-catalyzed oxa-Pictet–Spengler cyclization of (a*S*,2*S*,2′*S*)-**17** with six equivalents of 4-fluorobenzaldehyde, which cyclized both subunits and produced the (a*S*,1*R*,3*S*,1′*R*,3′*S*)-**20** as the major product. We could purify this product by column chromatography, obtaining it as a single stereoisomer with 59% yield (Scheme 5).

The cyclization introduced two additional chirality centers at the C-1 and C-1′ and the inherent stereogenic elements, especially the C-3 and C-3′ chirality centers, governed the diastereoselective formation of the new ones. Single-crystal X-ray analysis confirmed the (a*S*) axial chirality of (a*S*,1*R*,3*S*,1′*R*,3′*S*)-**20**, which is consistent with the VCD calculation result for (a*S*,3*S*,3′*S*)-**19** (CCDC deposition no.: 2467523). The analysis also confirmed the *cis* relative configuration of the C-1/C-3 and C-1′/C-3′ substituents in the isochroman subunits. This relative configuration of the central chirality elements is denoted as *cis*,*cis*. In the *cis* relative configuration, both the C-1 aryl and the C-3 methyl group adopt the favorable *pseudoequatorial* arrangement. We detected the formation of other stereoisomeric products of **20** as minor products but could not efficiently separate them with column chromatography. Thus we prepared them independently in the acid-catalyzed isomerization experiments of (a*S*,1*R*,3*S*,1′*R*,3′*S*)-**20** and purified them by preparative HPLC (*vide infra*).

**Scheme 6:**
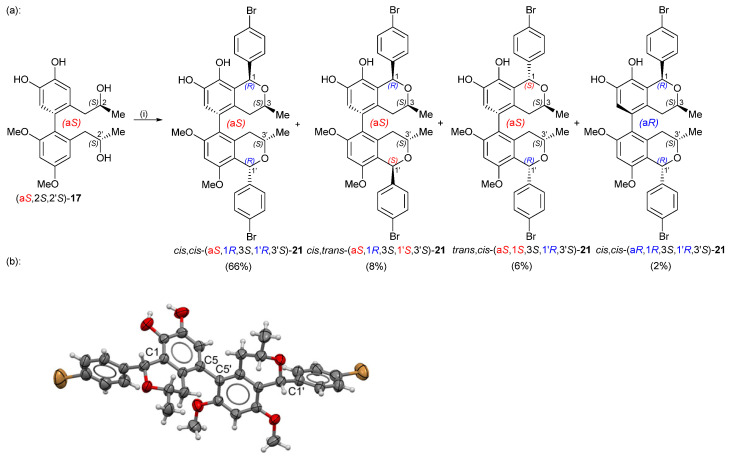
(**a**) Oxa-Pictet–Spengler cyclization of (a*S*,2*S*,2′*S*)-**17** with 4-bromobenzaldehyde. Reagents and conditions: (i) 4-bromobenzaldehyde (6 eq.), (+)-camphorsulfonic acid (1 eq.), toluene–MeOH 4:1, 80 °C. (**b**) ORTEP view of *cis*,*cis*-(a*S*,1*R*,3*S*,1′*R*,3′*S*)-**21** at 50% probability level (CCDC deposition no.: 2467524). Single crystals were produced from MeOH/H_2_O 5:2.

The oxa-Pictet–Spengler cyclization with an excess of 4-bromobenzaldehyde afforded four stereoisomeric *bis*-isochroman products **21**, with the *cis*,*cis*-(a*S*,1*R*,3*S*,1′*R*,3′*S*)-**21** being the major one (Scheme 6a). This major product could be separated from the other three stereoisomers by column chromatography and isolated with a 66% yield. We isolated the stereoisomeric side-products *cis*,*trans*-(a*S*,1*R*,3*S*,1′*S*,3′*S*)-**21**, *trans*,*cis*-(a*S*,1*S*,3*S*,1′*R*,3′*S*)-**21** and *cis*,*cis*-(a*R*,1*R*,3*S*,1′*R*,3′*S*)-**21** as an inseparable mixture by column chromatography and separated them using preparative chiral HPLC.

**Figure 3:**
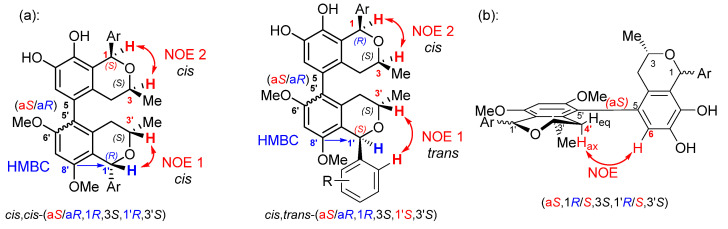
(**a**) Characteristic NOE/ROE correlations to determine the *cis* or *trans* relative configuration of the isochroman units. (**b**) Characteristic NOE/ROE correlation to determine the axial chirality.

We grew single crystals of the major product *cis*,*cis*-(a*S*,1*R*,3*S*,1′*R*,3′*S*)-**21** suitable for X-ray diffraction analysis (Scheme 6b, CCDC deposition no.: 2467524). This analysis confirmed the planar structure and absolute configuration of *cis*,*cis*-(a*S*,1*R*,3*S*,1′*R*,3′*S*)-**21**. We also determined the absolute configurations of the stereoisomers of **21** with 2D NMR experiments. Due to the heterodimeric nature of our *bis*-isochroman products, we could distinguish and assign the separate proton signals for the H-1/H-1′ and H-3/H-3′ pairs. NOE or ROE correlations were observed for the axially oriented H-1/H-3 or H-1′/H-3′ protons when the isochroman units adopted a *cis* relative configuration (Figure 3a). In the absence of H-1′/H-3′ correlation, the axial H-3′ gives an NOE correlation with the *ortho*-protons of the axial C-1′ aryl group, proving the *trans* relative configuration of the isochroman. Since the (3*S*) and (3′*S*) absolute configurations are retained during the synthesis, the assignment of the relative configuration for C-1 and C-1′ allows determining the absolute one as well. Regardless the *cis* or *trans* relative configuration of the isochroman units, the condensed heterocyclic ring always adopted a half-chair conformation, which placed the C-3 or C-3′ methyl group to a *pseudoequatorial* orientation. Knowing the (3′*S*) absolute configuration and the preferred helicity of the heterocyclic ring, we could use the NOE correlation between the H-4′_ax_ and H-6 protons to determine the (a*S*) absolute configuration of the biaryl axis (Figure 3b).

**Scheme 7:**
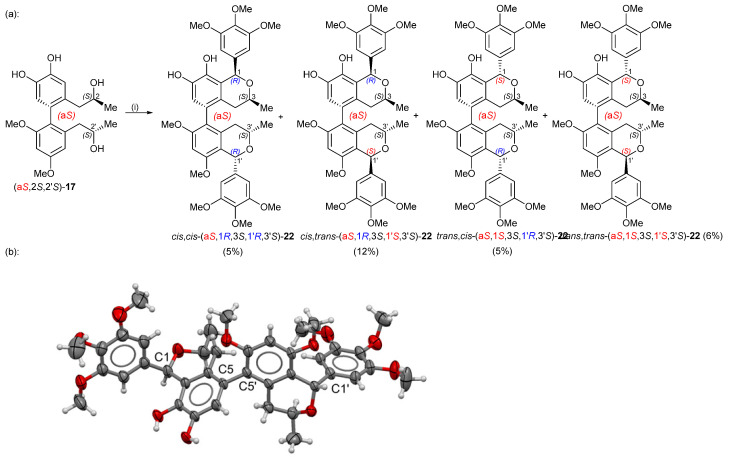
(**a**) Oxa-Pictet–Spengler cyclization of (a*S*,2*S*,2′*S*)-**17** with 3,4,5-trimethoxybenzaldehyde. Reagents and conditions: (i) 3,4,5-trimethoxybenzaldehyde (6 eq.), (+)-camphorsulfonic acid (1 eq.), toluene–MeOH 4:1, 80 °C. (**b**) ORTEP view of *cis*,*trans*-(a*S*,1*R*,3*S*,1′*S*,3′*S*)-**22** at 50% probability level (CCDC deposition no.: 2467525). Single crystals were produced from CHCl_3_/hexane 1:3.

The precursor (a*S*,2*S*,2′*S*)-**17** was also reacted with 3,4,5-trimethoxybenzaldehyde, and two fractions of the resulting products were collected using column chromatography (Scheme 7). These fractions were found to be mixtures of stereoisomeric products **22**. Preparative HPLC analysis allowed separating and isolating all four possible stereoisomers of **22**. However, in contrast to the previous examples, the *trans* products were found to be the major products of this reaction. This occurred due to an increased repulsive interaction between the *meta* substituents of the C-1/C-1′ aryl group and the C-8/C-8′ *peri* substituent, which decreased significantly when the C-1/C-1′ aryl group adopted a *pseudoaxial* orientation in the *trans* stereoisomer.

A single-crystal X-ray diffraction analysis of the major product, *cis*,*trans*-(a*S*,1*R*,3*S*,1′*S*,3′*S*)-**22**, confirmed its planar structure and absolute configuration (CCDC deposition no.: 2467525). It also revealed that the C-1′ aryl group adopted a *pseudoaxial* orientation with a *trans* relative configuration of the isochroman subunit. For the other three products, the absolute configurations of C-1 and C-1′ chirality centers and the biaryl axis were determined by NMR experiments.

**Scheme 8:**
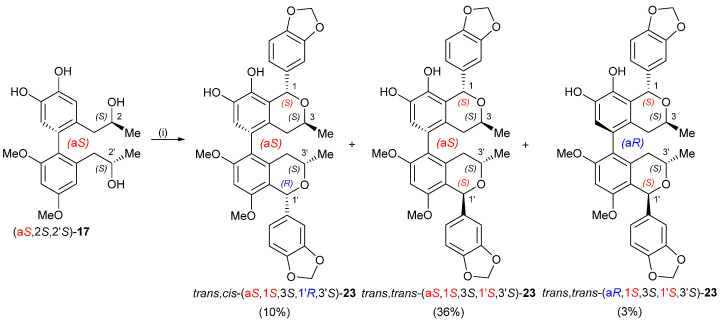
Oxa-Pictet–Spengler cyclization of (a*S*,2*S*,2′*S*)-**17** with piperonal [3,4-(methylenedioxy)benzaldehyde]. Reagents and conditions: (i) piperonal (6 eq.), (+)-camphorsulfonic acid (1 eq.), toluene–MeOH 4:1, 80 °C.

Using piperonal [3,4-(methylenedioxy)benzaldehyde] as a reagent, the *trans*,*trans*-(a*S*,1*S*,3*S*,1′*S*,3′*S*)-**23** was isolated by column chromatography as the major product (36%) of the cyclization (Scheme 8). Chiral preparative HPLC analysis enabled the separation and isolation of two additional stereoisomeric products; *trans*,*cis*-(a*S*,1*S*,3*S*,1′*R*,3′*S*)-**23** and *trans*,*trans*-(a*R*,1*S*,3*S*,1′*S*,3′*S*)-**23**. The latter minor product, *trans*,*trans*-(a*R*,1*S*,3*S*,1′*S*,3′*S*)-**23**, has (a*R*) axial chirality, which derived from the small diastereomeric (a*R*,2*S*,2′*S*)-**17** impurity (< 5%) of the starting material (a*S*,2*S*,2′*S*)-**17**.

**Scheme 9:**
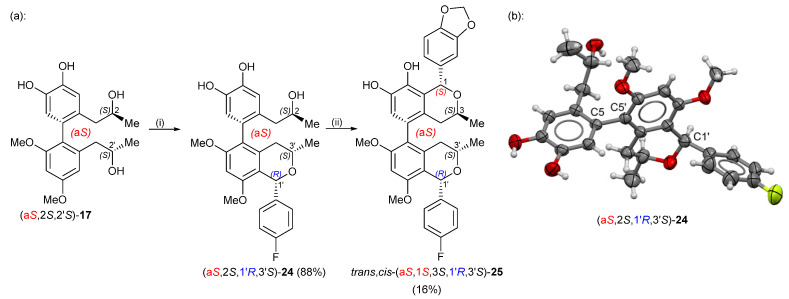
(**a**) Step-wise oxa-Pictet–Spengler cyclization of (a*S*,2*S*,2′*S*)-**17** to introduce different C-1/C-1′ aryl substituents. Reagents and conditions: (i) 4-fluorobenzaldehyde (1.2 eq.), (+)-camphorsulfonic acid (0.5 eq.), toluene/MeOH 4:1, 80 °C; (ii) piperonal (2.0 eq.), (+)-camphorsulfonic acid (1.0 eq.), toluene/MeOH 4:1, 80 °C. (**b**) ORTEP view of *cis*-(a*S*,2*S*,1′*R*,3′*S*)-**24** at 50% probability level (CCDC deposition no.: 2467526). Single crystals were produced from EtOAc.

During the cyclization with aryl aldehydes we observed that the dimethoxyaryl subunit of (a*S*,2*S*,2′*S*)-**17** reacted faster than the dihydroxyaryl one, which could be exploited to introduce different aryl groups to C-1 and C-1′. The greater reactivity of the dimethoxyaryl subunit was attributed to the presence a methoxy group in the *para* position relative to the cyclization site, which significantly promoted the S_E_Ar step. In contrast, the dihydroxyaryl subunit does not have a substituent in the *para* position to the cyclization site, and thus it is less activated, even though the hydroxy groups are stronger activating substituents than the methoxy groups. To facilitate introducing different aryl groups, we first reacted (a*S*,2*S*,2′*S*)-**17** with one equivalent of 4-fluorobenzaldehyde, which afforded selectively the expected *cis*-(a*S*,2*S*,1′*R*,3′*S*)-**24** with a high yield (88%) (Scheme 9). The planar structure and absolute configuration of *cis*-(a*S*,2*S*,1′*R*,3′*S*)-**24** were confirmed by single-crystal X-ray diffraction analysis (CCDC deposition no.: 2467526). Then 2.0 equivalents of piperonal were reacted with *cis*-(a*S*,2*S*,1′*R*,3′*S*)-**24** to cyclize the second aryl unit. During the second cyclization, different stereoisomers formed and the existing stereogenic elements also underwent isomerization, which reduced the yield. By means of column chromatography, we could isolate *trans*,*cis*-(a*S*,1*S*,3*S*,1′*R*,3′*S*)-**25** with 16% yield, while the mixture of other stereoisomers was not analyzed further. In accordance with the previous observations, the second cyclization with piperonal occurred with *trans* diastereoselectivity to reduce the repulsive *peri* interaction.

**Scheme 10:**
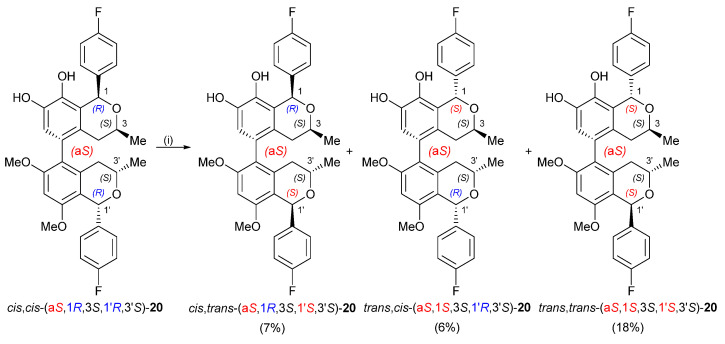
Isomerization studies of *cis*,*cis*-(a*S*,1*R*,3*S*,1′*R*,3′*S*)-**20** to produce other stereoisomers. Reagents and conditions: (i) TfOH (4.0 equiv.), 1,4-dioxane, reflux, 3 h, 90% conversion.

We performed acid-catalyzed isomerization experiments of *cis*,*cis*-(a*S*,1*R*,3*S*,1′*R*,3′*S*)-**20**, the major product in the cyclization with 4-fluorobenzaldehyde, to prepare the other minor stereoisomers of **20** with *trans* relative configuration in the isochroman units. The effects of temperature, reaction time, and the quality and quantity of Brønsted acids on the isomerization reactions were examined using 1,4-dioxane as a solvent. Higher temperatures and larger amounts of the Brønsted acid accelerated the isomerization. Among the tested trifluoromethanesulfonic acid (TfOH), hydrochloric acid and (+)-camphorsulfonic acid, TfOH was found the most effective. Based on our experimental results, the isomerization reaction of (a*S*,1*R*,3*S*,1′*R*,3′*S*)-**20** successfully produced the other three diastereomers with 90% conversion (Scheme 10). The stereoisomers *cis*,*trans*-**20**, *trans*,*cis*-**20** and *trans*,*trans*-**20** were separated and isolated with chiral preparative HPLC, and *trans*,*trans*-**20** was the major product of the isomerization. We also experienced a high amount of decomposition, which was responsible for the lower yields. Further studies revealed that decomposition can be cut back by using water/acetic acid 9:1 as a solvent with TfOH at 100 °C.

**Scheme 11:**
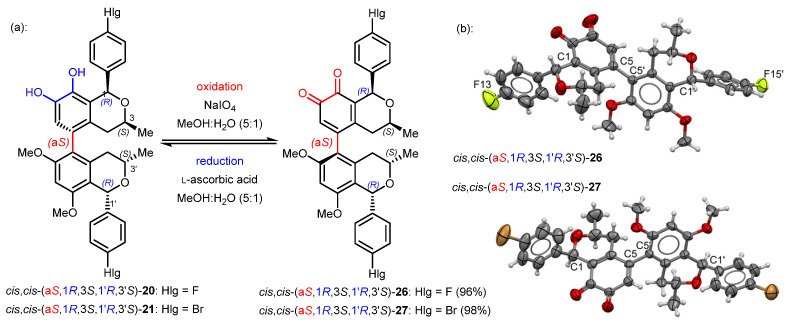
(**a**) Redox reaction of axially chiral *bis*-1,1′-biarylisochroman derivatives. (**b**) ORTEP views of *cis*,*cis*-(a*S*,1*R*,3*S*,1′*R*,3′*S*)-**26** (CCDC deposition no.: 2467527) and *cis*,*cis*-(a*S*,1*R*,3*S*,1′*R*,3′*S*)-**27** (CCDC deposition no.: 2467528) at 50% probability level.

We observed the oxidation of the 7,8-catechol moiety of our *bis*-isochromans to an *ortho*-quinone subunit as a side-reaction during oxa-Pictet–Spengler cyclizations in solution. We then carried out the oxidation reaction of *cis*,*cis*-(a*S*,1*R*,3*S*,1′*R*,3′*S*)-**20** and *cis*,*cis*-(a*S*,1*R*,3*S*,1′*R*,3′*S*)-**21** with sodium metaperiodate (NaIO_4_) to produce the corresponding *ortho*-quinone derivatives *cis*,*cis*-(a*S*,1*R*,3*S*,1′*R*,3′*S*)-**26** and *cis*,*cis*-(a*S*,1*R*,3*S*,1′*R*,3′*S*)-**27** with excellent yields (Scheme 11a). Their planar structures and absolute configurations were confirmed by single-crystal X-ray diffraction analysis (Scheme 11b, CCDC deposition numbers: 2467527 and 2467528). Conversely, the reduction of the *ortho*-quinone derivatives was carried out quickly using L-ascorbic acid (Scheme 11), which can also be used to stabilize catechol derivatives, as in parenteral pharmaceutical products (e.g., adrenaline injections). The optimized redox reactions take place instantaneously.

The antibacterial activities of the target compounds were evaluated against four Gram-positive and one Gram-negative bacterial strain: *Bacillus subtilis* ATCC 6633, *Enterococcus faecalis* ATCC 51299, methicillin-sensitive *Staphylococcus aureus* (MSSA) ATCC 29213, methicillin-resistant *Staphylococcus aureus* (MRSA) ATCC 33591, and *Acinetobacter baumannii* ATCC BAA1605. While most of the target compounds exhibited antibacterial activity against *Bacillus subtilis* and *Enterococcus faecalis,* with low µg/mL MIC values (Table 2), they were inactive against the other three strains (MIC > 64 µg/mL).

The MIC values, tabulated in Table 2 (in µg/mL), demonstrate the observed antibacterial activities are markedly influenced by both the stereochemistry and the substitution pattern. For instance, *cis*,*cis*-(a*S*,1*R*,3*S*,1′*R*,3′*S*)-**20**, the major product of the cyclization with 4-fluorobenzaldehyde, was inactive (entry 2, MIC > 64 µg/mL) against *Bacillus subtilis* and *Enterococcus faecalis*, while its stereoisomers containing at least one *trans*-isochroman moiety (entries 3–5) exhibited the most potent activities down to 0.5 µg/mL and 4 µg/mL MIC values. Furthermore, the *cis*,*cis*-(a*S*,1*R*,3*S*,1′*R*,3′*S*)-**21**, the bromo analog of *cis*,*cis*-(a*S*,1*R*,3*S*,1′*R*,3′*S*)-**20**, was active against both strains with 4 µg/mL and 1 µg/mL MIC values, respectively (entry 6). Of the four diastereoisomers of **22**, differing in the absolute configuration at C-1 and C-1′, *cis*,*trans*-(a*S*,1*R*,3*S*,1′*S*,3′*S*)-**22** exhibited the greatest activity with 4 µg/mL MIC values against both strains (entries 7–10). In addition to the central chirality elements C-1 and C-1′, the (a*S*)/(a*R*) axial chirality also affected the antibacterial activity as demonstrated by the fourfold difference in activity between the atropodiastereomers *trans*,*trans*-(a*S*,1*S*,3*S*,1′*S*,3′*S*)-**23** and *trans*,*trans*-(a*R*,1*S*,3*S*,1′*S*,3′*S*)-**23** (entries 11–13).

## 3. Materials and Methods

### 3.1. General Information

Chemicals were purchased puriss p.a. from commercial suppliers. The indicated higher/lower temperature values (°C) other than room temperature (25 °C) for the reactions were referred to the temperature of the heating/cooling units (oil bath, iced water, acetone cooled by liquid nitrogen). Thin layer chromatography (TLC) was performed on Silica gel 60 F_254_ (Merck & Co., Inc., Rahway, NJ, USA) with visualization by UV-light (254 nm) and immersing into ethanolic solution of sulfuric acidic vanillin (2 g vanillin and 2 mL cc. H_2_SO_4_ in 98 mL 96% ethanol) followed by heating. Column chromatography was performed on Silica gel 60 (Merck 0.040–0.063 mm for flash column chromatography and 0.063–0.200 mm for conventional column chromatography). Melting points were determined on a Kofler hot-stage apparatus and are uncorrected. Anhydrous solvents were used for all the reactions and distilled solvents were used as eluent for column chromatography. HPLC-grade solvents were used for chiral HPLC separations. Preparative chiral HPLC was performed by Agilent 1260 Infinity II apparatus using Lux i-Amylose-5 and Lux i-Cellulose-5 columns.

The ^1^H NMR (360 MHz, 400 MHz, 500 MHz, 700 MHz) and ^13^C NMR (90 MHz, 100 MHz, 125 MHz, 175 MHz) spectra were recorded with Bruker Avance DRX 360 MHz, Bruker Avance I 400 MHz, Bruker Avance II 500 MHz and Bruker Avance Neo 700 MHz spectrometers at 298 K. Chemical shifts are referenced to Me_4_Si (CDCl_3_, acetone-*d*_6_: 0.00 ppm for ^1^H) and to the residual solvent signals (CDCl_3_: 77.16 ppm for ^13^C, acetone-*d*_6_: 29.84 ppm for ^13^C, acetonitril-*d_3_*: 2.13 ppm for ^1^H and 118.26 ppm for ^13^C). Chemical shifts were reported as *δ* in ppm, and ^1^*J*_C-F_, ^2^*J*_H-H_, ^2^*J*_C-F_, ^3^*J*_H-H_, ^3^*J*_C-F_, ^4^*J*_H-H_ and ^4^*J*_C-F_ coupling constants in Hz. IR spectra were recorded on a JASCO FT/IR-4100 spectrometer (JASCO Corporation, Tokyo, Japan) and absorption bands are presented as wavenumber in cm^−1^. Optical rotations were measured at room temperature with a Perkin-Elmer 241 automatic polarimeter (*c* [g/100mL]) (PerkinElmer, Shelton, CT, USA). ECD spectra were recorded on a J-810 spectropolarimeter (JASCO Corporation, Tokyo, Japan). VCD measurements were performed on a BioTools ChiralIR-2X spectrometer (BioTools, Inc., West Palm Beach, FL, USA) at a resolution of 4 cm^−1^ under ambient temperature for 18 × 3000 scans, respectively. Samples were dissolved in CDCl_3_ and the solutions were placed in a 100 μm BaF_2_ cell. For spectroscopic measurements spectroscopic grade solvents were used.

#### 3.1.1. Syntheses and Characterization of the Compounds

##### General Procedure for Synthesis of Optically Active 1-arylpropan-2-ols

The corresponding aryl bromide (1.5 equiv) was dissolved in anhydrous THF (~1 g aryl bromide/10 mL anhydrous THF) under argon atmosphere and the solution was cooled to −78 °C. Then 2.5 M *n*-BuLi in hexane (1.5 equiv.) was added and after stirring for 20 min, (*S*)-propylene oxide (1.0 equiv., ≥98.0 *ee*%) was added and the reaction was stirred for 20 min at −78 °C. Next BF_3_.Et_2_O (1.1 equiv.) was added to the solution, which was stirred further for 30 min at −78 °C. Then the cooling was stopped and a saturated solution of NH_4_Cl was added to the reaction mixture. The mixture was stirred for 10 min and concentrated in *vacuo*. The suspension was diluted with EtOAc and water. The two layers were separated in a separatory funnel. The aqueous phase was washed three times with EtOAc. The combined organic phases were washed with brine. The organic phase was dried over anhydrous MgSO_4_, filtered, and the solvent was evaporated in *vacuo*. The residue was purified by flash chromatography to yield the optically active 1-arylpropan-2-ol derivative.

**(*S*)-1-(3,4-dibenzyloxyphenyl)propan-2-ol [(*S*)-11]**

Flash chromatography: hexanes/acetone 4:1. (*S*)-**11**: 5.38 g (yield: 90%) colorless oil. R_f_ = 0.27 (hexanes/acetone 4:1). [α${]}_{D}^{20}$ +10 (*c* = 0.32; CHCl_3_). ^1^H NMR (500 MHz, CDCl_3_) *δ* = 7.44–7.40 (m, 4H, H-12, H-16, H-19, H-23), 7.33 (t, *J* = 7.3 Hz, 4H, H-13, H-15, H-20, H-22), 7.27 (t, *J* = 7.3 Hz, 2H, H-14, H-21), 6.87 (d, *J* = 8.1 Hz, 1H, H-8), 6.79 (d, *J* = 1.9 Hz, 1H, H-5), 6.70 (dd, *J* = 8.1, 1.9 Hz, 1H, H-9), 5.13, 5.11 (2s, 2 × 2H, H-10, H-17), 3.93–3.86 (m, 1H, H-2), 2.65 (dd, *J* = 13.6, 4.7 Hz, 1H, H-1-a), 2.55 (dd, *J* = 13.6, 7.9 Hz, 1H, H-1-b), 1.53 (s, 1H, OH), 1.16, (d, *J* = 6.2 Hz, 3H, H-3); ^13^C NMR (125 MHz, CDCl_3_) *δ* = 149.0, 148.0 (2C, C-6, C-7), 137.6, 137.4 (2C, C-11, C-18), 132.0, (1C, C-4), 128.5, 127.5, 127.4 (8C, C-12, C-13, C-15, C-16, C-19, C-20, C-22, C-23), 127.9, 127.8 (2C, C-14, C-21), 122.4, 116.9, 115.6 (3C, C-5, C-8, C-9), 71.6, 71.5 (2C, C-10, C-17), 68.9 (1C, C-2), 45.3 (1C, C-1), 22.8 (1C, C-3). IR (KBr): 3390, 2960, 1514, 1260, 1232, 1136, 1117, 1012, 1000, 742, 697 cm^–1^. HRMS (ESI) calcd. for C_23_H_24_NaO_3_ [M+Na]^+^ 371.1618, found 371.1616.

**(*S*)-1-(3,5-dimethoxyphenyl)propan-2-ol [(*S*)-6]**

Flash chromatography: hexanes/EtOAc 4:1 → 3:1. (*S*)-**6**: 3.62 g (yield: 92%) colorless oil. R_f_ = 0.24 (hexanes/EtOAc 3:1). [α${]}_{D}^{20}$ +18, (*c* = 0.34; CHCl_3_). ^1^H NMR (400 MHz, CDCl_3_) *δ* = 6.36 (d, *J* = 2.3 Hz, 2H, H-5, H-9), 6.34 (t, *J* = 2.3 Hz, 1H, H-7), 4.05–3.95 (m, 1H, H-2), 3.77 (s, 6H, H-10, H-11), 2.70 (dd, *J* = 13.4, 4.9 Hz, 1H, H-1-a), 2.62 (dd, *J* = 13.4, 8.0 Hz, 1H, H-1-b), 1.23 (d, *J* = 6.2 Hz, 3H, 3-H); ^13^C NMR (100 MHz, CDCl_3_) *δ* = 160.9 (2C, C-6, C-8), 141.0 (1C, C-4), 107.4 (2C, C-5, C-9), 98.5 (1C, C-7), 68.8 (1C, C-2), 55.3 (2C, C-10, C-11), 46.1 (1C, C-1), 22.8 (1C, C-3). IR (KBr): 3419, 2965, 2934, 2839, 1596, 1205, 1150, 1068, 827, 701 cm^–1^. HRMS (ESI) calcd. for C_11_H_16_NaO_3_ [M+Na]^+^ 219.0992, found 219.0983.

##### General Procedure for Acetylation of Chiral Non-Racemic 1-arylpropan-2-ols

The corresponding 1-arylpropan-2-ol derivative (1.0 equiv.) was dissolved in anhydrous CH_2_Cl_2_ and anhydrous C_5_H_5_N (1.5 equiv.) was added to the solution. The mixture was cooled to 0 °C and AcCl (1.2 equiv.) was added, then the reaction was stirred at room temperature. After the starting material was consumed (1.5–3 h) on the basis of TLC monitoring, water was added to the mixture and stirred for 5 min. The mixture was diluted with CH_2_Cl_2_ and extracted with a 6N solution of HCl. The aqueous phase was washed three times with CH_2_Cl_2_, then the combined organic phases were washed with brine, dried over anhydrous MgSO_4_. After filtration, the solvent was evaporated in vacuo. The residue was purified by flash chromatography to yield the 1-arylpropan-2-yl acetate target derivative.

**(*S*)-1-(3,4-dibenzyloxyphenyl)propan-2-yl acetate [(*S*)-12]**

Flash chromatography: hexanes/EtOAc 10:1. (*S*)-**12**: 4.92 g (yield: 82%) colorless oil. R_f_ = 0.55 (hexanes/EtOAc 4:1). [α${]}_{D}^{20}$ −10 (*c* = 0.38; CHCl_3_). ^1^H NMR (500 MHz, CDCl_3_) *δ* = 7.46–7.41 (m, 4H, H-12, H-16, H-19, H-23), 7.34 (t, *J* = 7.6 Hz, 4H, H-13, H-15, H-20, H-22), 7.28 (t, *J* = 7.3 Hz, 2H, H-14, H-21), 6.85 (d, *J* = 8.2 Hz, 1H, H-8), 6.79 (d, *J* = 2.0 Hz, 1H, H-5), 6.69 (dd, *J* = 8.2, 2.0 Hz, 1H, H-9), 5.13, 5.12 (2s, 2 × 2H, H-10, H-17), 5.06–4.99 (m, 1H, 2-H), 2.81 (dd, *J* = 13.7, 6.5 Hz, 1H, H-1-a), 2.62 (dd, *J* = 13.7, 6.7 Hz, 1H, H-1-b), 1.95 (s, 3H, H-25), 1.14 (d, *J* = 6.3 Hz, 3H, H-3); ^13^C NMR (125 MHz, CDCl_3_) *δ* = 170.6 (1C, C-24), 148.8, 147.8 (2C, C-6, C-7), 137.5, 137.4 (2C, C-11, C-18), 131.1 (1C, C-4), 128.6, 127.5, 127.4 (8C, C-12, C-13, C-15, C-16, C-19, C-20, C-22, C-23), 127.9, 127.8 (2C, C-14, C-21), 122.5, 116.7, 115.3 (3C, C-5, C-8, C-9), 71.6 (1C, C-2), 71.5 (2C, C-10, C-17), 41.8 (1C, C-1), 21.4, 19.4 (2C, C-3, C-25). IR (KBr): 3445, 1734, 1511, 1373, 1246, 1137, 1017, 737, 697 cm^–1^. HRMS (ESI) calcd. for C_25_H_26_NaO_4_ [M+Na]^+^ 413.1723, found 413.1720.

**(*S*)-1-(3,5-dimethoxyphenyl)propan-2-yl acetate [(*S*)-7]**

Flash chromatography: hexanes/EtOAc 8:1. (*S*)-**7**: 3.68 g (yield: 91%) colorless oil. R_f_ = 0.55 (hexanes/EtOAc 4:1). [α${]}_{D}^{20}$ −11 (*c* = 0.33; CHCl_3_). ^1^H NMR (400 MHz, CDCl_3_) *δ* = 6.35 (d, *J* = 2.3 Hz, 2H, H-5, H-9), 6.33 (t, *J* = 2.3 Hz, 1H, H-7), 5.16–5.05 (m, 1H, H-2), 3.77 (s, 6H, H-10, H-11), 2.88 (dd, *J* = 13.5, 6.6 Hz, 1H, H-1-a), 2.66 (dd, *J* = 13.5, 6.7 Hz, 1H, H-1-b), 2.01 (s, 3H, H-13), 1.21 (d, *J* = 6.3 Hz, 3H, H-3); ^13^C NMR (100 MHz, CDCl_3_) *δ* = 170.6 (1C, C-12), 160.8, (2C, C-6, C-8), 140.0 (1C, C-4), 107.5 (2C, C-5, C-9), 98.6 (1C, C-7), 71.4 (1C, C-2), 55.3 (2C, C-10, C-11), 42.6 (1C, C-1), 21.4, 19.6 (2C, C-3, C-13). IR (KBr): 3447, 2934, 2839, 1732, 1595, 1240, 1203, 1149, 1054, 831, 702 cm^–1^. HRMS (ESI) calcd. for C_13_H_18_NaO_4_ [M+Na]^+^ 261.1097, found 261.1092.

##### General Procedure for the Halogenation of 1-arylpropan-2-yl Acetates with *N*-Halosuccinimides (NXS, X = I: iodo, B: Bromo)

The corresponding 1-arylpropan-2-yl acetate (1.0 equiv.) was dissolved in anhydrous MeCN (20–30 mL), then NIS (1.2 equiv.) and F_3_CCOOH (0.3 equiv.) or NBS (1.05 equiv.) were added, and the mixture was stirred at room temperature. When the starting material was consumed (1.5–16 h) on the basis of TLC monitoring, the solvent was evaporated in vacuo. EtOAc and water were added to the residual solid, and the phases were separated in a separatory funnel. The aqueous phase was washed three times with EtOAc, then the combined organic phases were washed with a 10% aqueous solution of Na_2_S_2_O_3_ and with brine. The organic phase was dried over anhydrous MgSO_4_, filtered, and the solvent was evaporated in vacuo. The residue was purified by trituration or flash chromatography to yield the 1-(2-haloaryl)propan-2-yl-acetate target derivatives.

**(*S*)-1-[(4,5-dibenzyloxy)-2-iodophenyl]propan-2-yl acetate [(*S*)-2]**

The crude brown-orange oil can be purified by trituration with hexanes or flash chromatography (hexanes/EtOAc 10:1). (*S*)-**2**: 6.23 g (yield: 96%) white-beige amorphous solid. R_f_ = 0.41 (hexanes/EtOAc 6:1). [α${]}_{D}^{20}$ +3 (*c* = 0.41; CHCl_3_). ^1^H NMR (500 MHz, CDCl_3_) *δ* = 7.46–7.23 (m, 10H, H-12, H-13, H-14, H-15, H-16, H-19, H-20, H-21, H-22, H-23), 7.33, 6.80 (2s, 2 × 1H, H-6, H-9), 5.18–5.09 (m, 1H, H-2), 5.10, 5.07 (2s, 2 × 2H, H-10, H-17), 2.90 (dd, *J* = 14.0, 7.6 Hz, 1H, H-1-a), 2.81 (dd, *J* = 14.0, 5.8 Hz, 1H, H-1-b), 1.92 (s, 3H, H-25), 1.20 (d, *J* = 6.3, 3H, H-3); ^13^C NMR (125 MHz, CDCl_3_) *δ* = 170.5 (1C, C-24), 149.0, 148.5 (2C, C-7, C-8), 137.0, 136.8 (2C, C-11, C-18), 133.9 (1C, C-4), 128.6, 127.5, 127.4 (8C, C-12, C-13, C-15, C-16, C-19, C-20, C-22, C-23), 128.1, 128.0 (2C, C-14, C-21), 125.3, 117.2 (2C, C-6, C-9), 90.0 (1C, C-5), 71.6, 71.5 (2C, C-10, C-17), 70.8 (1C, C-2), 46.0 (1C, C-1), 21.4, 19.7 (2C, C-3, C-25). IR (KBr): 3432, 2973, 1722, 1502, 1384, 1373, 1262, 1220, 732, 695 cm^–1^. HRMS (ESI) calcd. for C_25_H_25_INaO_4_ [M+Na]^+^ 539.0690, found 539.0684.

**(*S*)-1-[(4,5-dibenzyloxy)-2-bromophenyl]propan-2-yl acetate [(*S*)-13]**

Flash chromatography: hexanes/EtOAc 95:5 → 85:15. (*S*)-**13**: 466 mg (yield: 97%) white solid, mp 42–45 °C. R_f_ = 0.41 (hexanes/EtOAc 6:1). [α${]}_{D}^{20}$ +1 (*c* = 0.48; CHCl_3_). ^1^H NMR (500 MHz, CDCl_3_) *δ* = 7.45–7.23 (m, 10H, H-12, H-13, H-14, H-15, H-16, H-19, H-20, H-21, H-22, H-23), 7.10, 6.80 (2s, 2 × 1H, H-6, H-9), 5.15–5.06 (m, 1H, H-2), 5.10, 5.09 (2s, 2 × 2H, H-10, H-17), 2.92–2.80 (m, 2H, H-1-a,b), 1.92 (s, 3H, H-25), 1.19 (d, *J* = 6.3 Hz, 3H, H-3); ^13^C NMR (125 MHz, CDCl_3_) *δ* = 170.5 (1C, C-24), 148.6, 148.1 (2C, C-7, C-8), 137.0, 136.8 (2C, C-11, C-18), 130.0 (1C, C-4), 128.6, 127.5, 127.4 (8C, C-12, C-13, C-15, C-16, C-19, C-20, C-22, C-23), 128.1, 128.0 (2C, C-14, C-21), 119.0, 118.2 (2C, C-6, C-9), 115.8 (1C, C-5), 71.7, 71.6 (2C, C-10, C-17), 70.6 (1C, C-2), 41.6 (1C, C-1), 21.4, 19.7 (2C, C-3, C-25). IR (KBr): 3424, 2980, 1721, 1512, 1390, 1371, 1221, 1179, 734, 696 cm^–1^. HRMS (ESI) calcd. for C_25_H_25_BrNaO_4_ [M+Na]^+^ 491.0828, found 491.0824.

**(*S*)-1-(2-bromo-3,5-dimethoxyphenyl)propan-2-yl acetate [(*S*)-9]**

Flash chromatography: hexanes/acetone 9:1. (*S*)-**9**: 2.12 g (yield: 94%) colorless oil. R_f_ = 0.38 (hexanes/acetone 5:1). [α${]}_{D}^{20}$ +3 (*c* = 0.35; CHCl_3_). ^1^H NMR (400 MHz, CDCl_3_) *δ* = 6.42, 6.38 (d, *J* = 2.8 Hz, 1H, d, *J* = 2.8 Hz, 1H, H-7, H-9), 5.22 (m, 1H, H-2), 3.85, 3.78 (2s, 2 × 3H, H-10, H-11), 3.01 (d, *J* = 6.6 Hz, 2H, H-1), 1.99 (s, 3H, H-13), 1.27 (d, *J* = 6.3 Hz, 3H, H-3); ^13^C NMR (100 MHz, CDCl_3_) *δ* = 170.4 (1C, C-12), 159.4, 156.8 (2C, C-6, C-8), 139.2 (1C, C-4), 107.7, 98.2 (2C, C-7, C-9), 105.5 (1C, C-5), 70.5 (1C, C-2), 56.3, 55.5 (2C, C-10, C-11), 42.3 (1C, C-1), 21.4, 19.8 (2C, C-3, C-13). IR (KBr): 3453, 3084, 2932, 1731, 1584, 1455, 1328, 1237, 950, 830, 605 cm^–1^. HRMS (ESI) calcd. for C_13_H_17_BrNaO_4_ [M+Na]^+^ 339.0202, found 339.0201.

**(*S*)-1-(2-iodo-3,5-dimethoxyphenyl)propan-2-yl acetate [(*S*)-8a] and (*S*)-1-(4-iodo-3,5-dimethoxyphenyl)propan-2-yl [(*S*)-8b] acetate regioisomeric mixture (ratio 1:1)**

Flash chromatography: hexanes/EtOAc 8:1. Regioisomeric mixture of (*S*)-**8a** and (*S*)-**8b**: colorless oil. R_f_ = 0.39 (hexanes/acetone 5:1). ^1^H NMR (360 MHz, CDCl_3_) *δ* = 6.46 (d, *J* = 2.6 Hz, 1H, H-9), 6.35 (s, 2H, H-5′, H-9′), 6.32 (d, *J* = 2.6 Hz, 1H, H-7), 5.26–5.17 (m, 1H, H-2), 5.17–5.08 (m, 1H, H-2′), 3.87 (s, 6H, H-10′, H-11′), 3.85, 3.79 (2s, 2 × 3H, H-10, H-11), 3.07 (dd, *J* = 13.9, 5.9 Hz, 1H, H-1-a), 3.01 (dd, *J* = 13.9, 7.5 Hz, 1H, H-1-b), 2.92 (dd, *J* = 13.6, 6.9 Hz, 1H, H-1′-a), 2.72 (dd, *J* = 13.6, 6.5 Hz, 1H, H-1′-b), 2.01 (1s, 3H, H-13′), 1.99 (s, 3H, H-13), 1.30 (d, *J* = 6.3 Hz, 3H, H-3), 1.23 (d, *J* = 6.3 Hz, 3H, H-3′); ^13^C NMR (90 MHz, CDCl_3_) *δ* = 170.6 (1C, C-12′), 170.5 (1C, C-12), 160.8, 159.0, 142.8 (3C, C-4, C-6, C-8), 159.5, 140.2 (3C, C-4′, C-6′, C-8′), 107.7, 97.3 (2C, C-7, C-9), 105.5 (2C, C-5′, C-9′), 100.1 (1C, C-7′), 82.8 (1C, C-5), 71.2 (1C, C-2′), 70.9 (1C, C-2), 56.7 (2C, C-10′, C-11′), 56.6, 55.6 (2C, C-10, C-11), 46.8 (1C, C-1), 42.7 (1C, C-1′), 21.5, 20.0, 19.7 (4C, C-3, C-13, C-3′, C-13′). IR (KBr): 2975, 2935, 2839, 1732, 1578, 1238, 1200, 1162, 1120, 1056, 1010, 952, 830, 735 cm^–1^. HRMS (ESI) calcd. for C_13_H_17_INaO_4_ [M+Na]^+^ 387.0064, found 387.0061.

**(*S*)-1-(2,6-diiodo-3,5-dimethoxyphenyl)propan-2-yl acetate [(*S*)-8c]**

Flash chromatography: hexanes/EtOAc 8:1. (*S*)-**8c**: white amorphous solid. R_f_ = 0.24 (hexanes/acetone 5:1). [α${]}_{D}^{20}$ −25 (*c* = 0.31; CHCl_3_). ^1^H NMR (360 MHz, CDCl_3_) *δ* = 6.31 (s, 1H, H-7), 5.43–5.30 (m, 1H, H-2), 3.89 (s, 6H, H-10, H-11), 3.67 (dd, *J* = 13.8, 9.1 Hz, 1H, H-1-a), 3.42 (dd, *J* = 13.9, 4.2 Hz, 1H, H-1-b), 1.96 (s, 3H, H-13), 1.38 (d, *J* = 6.2 Hz, 3H, H-3); ^13^C NMR (90 MHz, CDCl_3_) *δ* = 170.5 (1C, C-12), 159.4 (2C, C-6, C-8), 144.4 (C-4), 93.7 (1C, C-7), 83.0 (2C, C-5, C-9), 71.0 (C-2), 56.9 (2C, C-10, C-11), 51.2 (1C, C-1), 21.5, 20.3 (2C, C-3, C-13). IR (KBr): 3432, 1728, 1566, 1321, 1249, 1213, 1083, 800 cm^–1^. HRMS (ESI) calcd. for C_13_H_16_I_2_NaO_4_ [M+Na]^+^ 512.9030, found 512.9026.

##### General Procedure for Miyaura Borylation of Chiral Non-Racemic 1-(2-haloaryl)propan-2-yl Acetates

To the solution of the corresponding 1-(2-haloaryl)propan-2-yl acetate (1.0 equiv.) in anhydrous DMF (30–40 mL) Ph_3_P (0.2 equiv.), (Ph_3_P)_2_PdCl_2_ (0.1 equiv.) and freshly annealed KOAc (4.0 equiv.) were added under Ar atmosphere, and the mixture was stirred for 15 min with inert gas bubbling at room temperature. Then B_2_pin_2_ (3.0 equiv.) was added and the temperature was raised to 150 °C. After the starting material was consumed (1–3 h) on the basis of TLC monitoring, the reaction mixture was poured on ice and diluted with Et_2_O. The mixture was filtered on a short pad of Celite^®^ using a glass filter. The Celite^®^ was washed three times with Et_2_O. Next, the two layers were extracted and separated in a separatory funnel. The aqueous phase was washed three times with Et_2_O. The combined organic layers were washed with brine, dried over anhydrous MgSO_4_, filtered, and the solvent was evaporated in *vacuo*. The residue was purified by column chromatography to yield the 1-[2-(pinacolatoboryl)aryl]propan-2-yl acetate target derivative.

**(*S*)-1-[4,5-*bis*(benzyloxy)-2-(4,4,5,5-tetramethyl-1,3,2-dioxaborolan-2-yl)phenyl]propan-2-yl acetate[(*S*)-14]**

Conventional column chromatography: hexanes/acetone 16:1 → 15:1 → 12:1. (*S*)-**14**: 5.69 g (yield: 92%) colorless oil. R_f_ = 0.23 (toluenes/EtOAc 10:0.25). [α${]}_{D}^{20}$ −1 (*c* = 0.45; CHCl_3_). ^1^H NMR (400 MHz, CDCl_3_) *δ* = 7.50–7.40, 7.38–7.23 (2m, 10H, H-12, H-13, H-14, H-15, H-16, H-19, H-20, H-21, H-22, H-23), 7.43, 6.78 (2s, 2 × 1H, H-6, H-9), 5.16, 5.13 (2s, 2 × 2H, H-10, H-17), 5.07–4.97 (m, 1H, H-2), 3.13 (dd, *J* = 13.2, 5.8 Hz, 1H, H-1-a), 2.99 (dd, *J* = 13.2, 7.5 Hz, 1H, H-1-b), 1.89 (s, 3H, H-25), 1.32 (1s, 12H, H-31, H-32, H-33, H-34), 1.16 (d, *J* = 6.2 Hz, 3H, H-3); ^13^C NMR (100 MHz, CDCl_3_) *δ* = 170.5 (1C, C-24), 150.9, 146.9, 139.5, 137.6, 137.2 (5C, C-4, C-7, C-8, C-11, C-18), 128.5, 128.4, 127.7, 127.2 (8C, C-12, C-13, C-15, C-16, C-19, C-20, C-22, C-23), 127.8 (2C, C-14, C-21), 122.4, 117.0 (2C, C-6, C-9), 83.5 (2C, C-29, C-30), 73.2 (1C, C-2), 71.5, 70.8 (2C, C-10, C-17), 41.2 (1C, C-1), 25.0 (4C, C-31, C-32, C-33, C-34), 21.4, 19.6 (2C, C-3, C-25). IR (KBr): 3433, 2979, 2931, 1734, 1411, 1372, 1247, 1144, 850, 741, 696 cm^–1^. HRMS (ESI) calcd. for C_31_H_37_BNaO_6_ [M+Na]^+^ 539.2575, found 539.2574.

**(*S*)-1-[3,5-dimethoxy-2-(4,4,5,5-tetramethyl-1,3,2-dioxaborolan-2-yl)phenyl]propan-2-yl acetate [(*S*)-3]**

Flash chromatography: hexanes/EtOAc 6:1 → 5:1. (*S*)-**3**: 2.66 g (yield: 62%) colorless oil. R_f_ = 0.23 (hexanes/EtOAc 5:1). [α${]}_{D}^{20}$ −5 (*c* = 0.51; CHCl_3_). ^1^H NMR (360 MHz, CDCl_3_) *δ* = 6.34, 6.26 (d, *J* = 2.1 Hz, 1H, d, *J* = 2.1 Hz, 1H, H-7, H-9), 5.16–5.04 (m, 1H, H-2), 3.78, 3.74 (2s, 2 × 3H, H-10, H-11), 3.00 (dd, *J* = 13.4, 7.5 Hz, 1H, H-1-a), 2.73 (dd, *J* = 13.4, 6.2 Hz, 1H, H-1-b), 2.00 (s, 3H, H-13), 1.38, 1.37 (2s, 2 × 6H, H-19, H-20, H-21, H-22), 1.21 (d, *J* = 6.2 Hz, 3H, H-3); ^13^C NMR (90 MHz, CDCl_3_) *δ* = 170.6 (1C, C-12), 164.7, 161.9 (2C, C-6, C-8), 144.4 (1C, C-4), 106.6, 96.1 (2C, C-7, C-9), 83.6 (2C, C-17, C-18), 72.2 (1C, C-2), 55.7, 55.2 (2C, C-10, C-11), 42.4 (1C, C-1), 25.1, 24.7 (4C, C-19, C-20, C-21, C-22), 21.5, 19.8 (2C, C-3, C-13). IR (KBr): 2977, 2930, 2842, 1729, 1602, 1575, 1318, 1233, 1213, 1144, 964, 860, 837, 805, 687 cm^–1^. HRMS (ESI) calcd. for C_19_H_29_BNaO_6_ [M+Na]^+^ 387.1949, found 387.1945.

##### Suzuki Coupling Reaction of (*S*)-1-[(4,5-dibenzyloxy)-2-iodophenyl]propan-2-yl acetate [(*S*)-**2**] and (*S*)-1-[3,5-dimethoxy-2-(4,4,5,5-tetramethyl-1,3,2-dioxaborolan-2-yl)phenyl]propan-2-yl acetate [(*S*)-**3**]

To the solution of (*S*)-**2** (3.74 g, 7.25 mmol, 1.06 equiv.) in anhydrous DMF (30 mL), Xantphos (396 mg, 0.684 mmol, 0.1 equiv.) and Pd(OAc)_2_ (185 mg, 0.821 mmol, 0.12 equiv.) were added under Ar atmosphere, and the solution was stirred for 1 h with inert gas bubbling at room temperature. To the solution of (*S*)-**3** (2.49 g, 6.84 mmol, 1.0 equiv.) in anhydrous DMF (30 mL), CsF (2.28 g, 15.0 mmol, 2.2 equiv.) was added under Ar atmosphere, and the solution was stirred for 30 min with inert gas bubbling at room temperature. The first solution was merged with the second, and the reaction was stirred at 150 °C. When one of the starting material was consumed (1.5–2 h) on the basis of TLC monitoring, the reaction mixture was poured on ice and diluted with Et_2_O. The mixture was filtered on a short pad of Celite^®^ (Merck & Co., Rahway, NJ, USA) using glass filter. The Celite^®^ was washed three times with Et_2_O. The two layers were extracted and separated in a separatory funnel. The aqueous phase was washed three times with Et_2_O. The combined organic layers were washed with brine, dried over anhydrous MgSO_4_, filtered, and the solvent was evaporated in *vacuo*. The residue was purified by flash chromatography to yield the {4,5-*bis*(benzyloxy)-4′,6′-dimethoxy-[1,1′-biphenyl]-2,2′-diyl}*bis*(propane-2,1-diyl) diacetate target derivative [(a*S*,2*S*,2′*S*)-**15**].

**(a*S*,2*S*,2′*S*)-{(a*S*)-4,5-*bis*(benzyloxy)-4′,6′-dimethoxy-[1,1′-biphenyl]-2,2′-diyl}*bis*(propane-2,1-diyl) diacetate [(a*S*,2*S*,2′*S*)-15]**

Flash chromatography: hexanes/EtOAc 5:1 → 4:1. (a*S*,2*S*,2′*S*)-**15**: 2.70 g (yield: 63%) brown oil. R_f_ = 0.28 (hexanes/EtOAc 3:1). [α${]}_{D}^{20}$ −35, (*c* = 0.26; CHCl_3_). ^1^H NMR (400 MHz, CDCl_3_) *δ* = 7.50–7.45, 7.44–7.39, 7.38–7.23 (3m, 10H, H-12, H-13, H-14, H-15, H-16, H-19, H-20, H-21, H-22, H-23), 6.90, 6.70 (2s, 2 × 1H, H-6, H-9), 6.46, 6.40 (d, *J* = 2.4 Hz, 1H, d, *J* = 2.4 Hz, 1H, H-7′, H-9′), 5.18, 5.12, 5.09 (3s, 4H, H-10, H-17), 5.05–4.97, 4.93–4.83 (2m, 2 × 1H, H-2, H-2′), 3.81, 3.65 (2s, 2 × 3H, H-10′, H-11′), 2.56, 2.50 (dd, *J* = 14.1, 6.7 Hz, 1H, dd, *J* = 14.4, 8.0 Hz, 1H, H-1-a, H-1′-a), 2.40, 2.35 (dd, *J* = 14.3, 5.5 Hz, 1H, dd, *J* = 14.1, 7.0 Hz, 1H, H-1-b, H-1′-b), 1.93, 1.90 (2s, 2 × 3H, H-25, H-13′), 1.06, 0.99 (d, *J* = 6.2 Hz, 3H, d, *J* = 6.2 Hz, 3H, H-3, H-3′); ^13^C NMR (100 MHz, CDCl_3_) *δ* = 170.2 (2C, C-24, C-12′), 159.7, 158.0, 147.7, 147.1, 138.3, 137.5, 137.4, 130.2, 129.8, 122.3 (10C, C-4, C-5, C-7, C-8, C-11, C-18, C-4′, C-5′, C-6′, C-8′), 128.4, 128.3, 127.4, 127.3 (8C, C-12, C-13, C-15, C-16, C-19, C-20, C-22, C-23), 127.7, 127.6 (2C, C-14, C-21), 118.0, 116.6, 105.4, 96.6 (4C, C-6, C-9, C-7′, C-9′), 71.3, 71.1 (2C, C-10, C-17), 70.7, 70.6 (2C, C-2, C-2′), 55.4, 55.2 (2C, C-10′, C-11′), 38.9, 38.8 (2C, C-1, C-1′), 21.3, 20.0, 19.5 (4C, C-3, C-25, C-3′, C-13′). IR (KBr): 3032, 2979, 2933, 1733, 1604, 1455, 1372, 1244, 1158, 1056, 698 cm^–1^. HRMS (ESI) calcd. for C_38_H_42_NaO_8_ [M+Na]^+^ 649.2772, found 649.2768.

##### Deacetylation Reaction of (a*S*,2*S*,2′*S*)-{(a*S*)-4,5-*bis*(benzyloxy)-4′,6′-dimethoxy-[1,1′-biphenyl]-2,2′-diyl}*bis*(propane-2,1-diyl) diacetate [(a*S*,2*S*,2′*S*)-**15**]

To the solution of (a*S*,2*S*,2′*S*)-**15** (2.70 g, 4.31 mmol, 1.0 equiv.) in MeOH (25 mL) LiOH (413 mg, 17.2 mmol, 4.0 equiv.) was added and it was stirred at room temperature for 1.5 h. After the starting material was consumed on the basis of TLC monitoring, the solvent was evaporated in *vacuo*. The residue was dissolved in EtOAc and it was extracted with water. The aqueous phase was washed three times with EtOAc. The combined organic phases were washed with brine, dried over anhydrous MgSO_4_, filtered, and the solvent was evaporated in *vacuo*. The crude product was purified by flash chromatography to yield the product.

**(a*S*,2*S*,2′*S*)-1,1′-{(a*S*)-4,5-*bis*(benzyloxy)-4′,6′-dimethoxy-[1,1′-biphenyl]-2,2′-diyl}*bis*(propan-2-ol) [(a*S*,2*S*,2′*S*)-16]**

Flash chromatography: hexanes/EtOAc 1:1. (a*S*,2*S*,2′*S*)-**16**: 2.00 g (yield: 85%) white-pale yellow oil. R_f_ = 0.39 (hexanes/EtOAc 1:2). [α${]}_{D}^{20}$ +55 (*c* = 0.28; CHCl_3_). ^1^H NMR (360 MHz, CDCl_3_) *δ* = 7.51–7.45, 7.42–7.24 (2m, 10H, H-12, H-13, H-14, H-15, H-16, H-19, H-20, H-21, H-22, H-23), 6.90, 6.62 (2s, 2 × 1H, H-6, H-9), 6.46, 6.42 (d, *J* = 2.4 Hz, 1H, d, *J* = 2.4 Hz, 1H, H-7′, H-9′), 5.19, 5.14, 5.09 (3s, 4H, H-10, H-17), 3.90–3.78, 3.68–3.58 (2m, 2 × 1H, H-2, H-2′), 3.83, 3.65 (2s, 2 × 3H, H-10′, H-11′), 2.43, 2.33 (dd, *J* = 13.9, 3.2 Hz, 1H, dd, *J* = 13.6, 5.0 Hz, 1H, H-1-a, H-1′-a), 2.25, 2.16 (dd, *J* = 13.6, 8.2 Hz, 1H, dd, *J* = 13.9, 9.5 Hz, 1H, H-1-b, H-1′-b), 1.06, 0.98 (d, *J* = 6.1 Hz, 3H, d, *J* = 6.1 Hz, 3H, H-3, H-3′); ^13^C NMR (90 MHz, CDCl_3_) *δ* = 159.9, 157.8, 148.3, 147.1, 139.4, 137.5, 137.4, 131.0, 130.0, 122.6 (10C, C-4, C-5, C-7, C-8, C-11, C-18, C-4′, C-5′, C-6′, C-8′), 128.6, 128.5, 127.6, 127.4 (8C, C-12, C-13, C-15, C-16, C-19, C-20, C-22, C-23), 127.9, 127.7 (2C, C-14, C-21), 117.8, 116.0, 106.7, 96.9 (4C, C-6, C-9, C-7′, C-9′), 71.4, 71.0 (2C, C-10, C-17), 68.9, 67.7 (2C, C-2, C-2′), 55.7, 55.4 (2C, C-10′, C-11′), 43.2, 42.6 (2C, C-1, C-1′), 23.2 (2C, C-3, C-3′). IR (KBr): 3433, 2965, 2930, 1603, 1455, 1318, 1202, 1157, 737, 698 cm^–1^. HRMS (ESI) calcd. for C_34_H_38_NaO_6_ [M+Na]^+^ 565.2561, found 565.2555.

##### General Procedure for Debenzylation (Hydrogenation) of Benzyl-Protected Biaryl *bis*(propan-2-ol) Derivatives

Pd/C catalyst (10 *w*/*w*%, 0.26 equiv.) was dispersed in THF and it was stirred at room temperature for 20 min under hydrogen atmosphere. The corresponding benzyl-protected biaryl derivative (1.0 equiv.) was added to the suspension, and the reaction mixture was stirred further under H_2_ atmosphere at room temperature until the end of hydrogen lessening. After that, the mixture was filtered through a short pad of Celite^®^. The Celite^®^ was washed with THF, and the solvent was evaporated in *vacuo*. The residue was purified by column chromatography to yield the pyrocatechol target derivatives.

**(a*S*)-2′,6-*bis*[(*S*)-2-hydroxypropyl]-4′,6′-dimethoxy-[1,1′-biphenyl]-3,4-diol [(a*S*,2*S*,2′*S*)-17]**

Flash chromatography: hexanes/acetone 1.5:1. (a*S*,2*S*,2′*S*)-**17**: 1.00 g (yield: 97%) white foam. R_f_ = 0.78 (hexanes/acetone 1:1). [α${]}_{D}^{20}$ +55 (*c* = 0.20; CHCl_3_). ^1^H NMR (400 MHz, CDCl_3_) *δ* = 6.71, 6.51 (2s, 2 × 1H, H-6, H-9), 6.44, 6.40 (d, *J* = 2.2 Hz, 1H, d, *J* = 2.2 Hz, 1H, H-7′, H-9′), 3.90–3.75 (m, 2H, H-2, H-2′), 3.83, 3.63 (2s, 2 × 3H, H-10′, H-11′), 2.48–2.34 (m, 3H, H-1-a, H-1′-a, H-1-b or H-1′-b), 2.15 (dd, *J* = 13.8, 9.6 Hz, 1H, H-1-b or H-1′-b), 1.08, 1.04 (d, *J* = 6.1 Hz, 3H, d, *J* = 6.0 Hz, 3H, H-3, H-3′); ^13^C NMR (100 MHz, CDCl_3_) *δ* = 159.8, 157.8, 143.8, 142.6, 139.4, 129.9, 128.8, 122.8 (8C, C-4, C-5, C-7, C-8, C-4′, C-5′, C-6′, C-8′), 118.4, 116.5, 106.7, 96.9 (4C, C-6, C-9, C-7′ C-9′), 68.9, 68.6 (2C, C-2, C-2′), 55.7, 55.5 (2C, C-10′, C-11′), 43.2, 42.2 (2C, C-1, C-1′), 23.2, 22.9 (2C, C-3, C-3′). IR (KBr): 3376, 2970, 2932, 2841, 1606, 1456, 1158, 1068, 831 cm^–1^. HRMS (ESI) calcd. for C_20_H_26_NaO_6_ [M+Na]^+^ 385.1622, found 385.1619.

**(a*S*,3*S*,3′*S*)-6′,8′-dimethoxy-3,3′-dimethyl-[5,5′-*bis*-isochroman]-7,8-diol [(a*S*,3*S*,3′*S*)-19]**

Conventional column chromatography: hexanes/acetone 3:1. (a*S*,3*S*,3′*S*)-**19**: 70 mg (yield: 80%) white crystals, mp 122–124 °C. R_f_ = 0.15 (hexanes/acetone 3:1). [α${]}_{D}^{20}$ +71 (*c* = 0.28; CHCl_3_). ECD: (*c* = 1.98 × 10^−4^ M; MeCN) λ [nm], (Δε) = 289sh (−1.34), 237 (−7.92), 213 (24.79), 199sh (19.06). ^1^H NMR (400 MHz, CDCl_3_) *δ* = 7.60 (bs, 1H, OH), 6.36, 6.35 (2s, 2 × 1H, H-6, H-7′), 5.77 (bs, 1H, OH), 5.06, 5.00 (d, *J* = 15.6 Hz, 1H, d, *J* = 15.2 Hz, 1H, H-1-a, H-1′-a), 4.75, 4.67 (d, *J* = 15.6 Hz, 1H, d, *J* = 15.3 Hz, 1H, H-1-b, H-1′-b), 3.83, 3.71 (2s, 2 × 3H, H-9′, H-10′), 3.74–3.60 (m, 2H, H-3, H-3′), 2.43–2.23, 2.06–1.93 (2m, 2 × 2H, H-4, H-4′), 1.25, 1.23 (d, *J* = 6.5 Hz, 3H, d, *J* = 6.4 Hz, 3H, H-9, H-11′); ^13^C NMR (100 MHz, CDCl_3_) *δ* = 156.6, 155.4, 140.7, 139.8, 133.7, 126.5, 125.3, 121.5, 120.2, 114.8 (10C, C-4a, C-5, C-7, C-8, C-8a, C-4a′, C-5′, C-6′, C-8′, C-8a′), 115.3, 92.8 (2C, C-6, C-7′), 71.4, 71.0 (2C, C-3, C-3′), 64.9, 64.8 (2C, C-1, C-1′), 56.0, 55.3 (2C, C-9′, C-10′), 35.5, 33.3 (2C, C-4, C-4′), 21.7, 21.4 (2C, C-9, C-11′). IR (KBr): 3419, 2969, 2838, 1597, 1455, 1317, 1209, 1121, 1069, 936, 838 cm^−1^. HRMS (ESI) calcd. for C_22_H_26_NaO_6_ [M+Na]^+^ 409.1622, found 409.1621.

##### Cyclization Reaction by Chloromethyl Methyl ether (MOMCl) of Benzyl-Protected (a*S*,2*S*,2′*S*)-1,1′-{(a*S*)-4,5-*bis*(benzyloxy)-4′,6′-dimethoxy-[1,1′-biphenyl]-2,2′-diyl}*bis*(propan-2-ol) [(a*S*,2*S*,2′*S*)-**16**]

The benzyl-protected (a*S*,2*S*,2′*S*)-**16** (300 mg, 0.553 mmol, 1.0 equiv.) was dissolved in anhydrous THF (10 mL). The mixture was cooled to 0 °C then MOMCl (134 mg, 127 µL, 1.66 mmol, 3.0 equiv.) and freshly annealed ZnCl_2_ (23 mg, 0.166 mmol, 0.3 equiv.) were added under Ar atmosphere. The reaction mixture was stirred at room temperature until the starting material was consumed (ca. 20 h) on the basis of TLC monitoring. Then the mixture was quenched and stirred with water for 5 min. The mixture was diluted with EtOAc and the phases were separated in a separatory funnel. The aqueous phase was extracted three times with EtOAc. The combined organic phases were washed with a saturated solution of NaHCO_3_ and brine, dried over anhydrous MgSO_4_. After filtration, the solvent was evaporated in *vacuo*. The residue was purified by flash chromatography to yield the C1–C1′ unsubstituted *bis*-isochroman target derivative.

**(a*S*,3*S*,3′*S*)-7,8-*bis*(benzyloxy)-6′,8′-dimethoxy-3,3′-dimethyl-5,5′-*bis*-isochroman [(a*S*,3*S*,3′*S*)-18]**

Flash chromatography: hexanes/EtOAc 6.5:1 → 3:1. (a*S*,3*S*,3′*S*)-**18**: 130 mg (yield: 42%) white powder, mp 59–62 °C. R_f_ = 0.71 (hexanes/EtOAc 2:1). [α${]}_{D}^{20}$ +72 (*c* = 0.23; CHCl_3_). ^1^H NMR (400 MHz, CDCl_3_) *δ* = 7.45–7.37, 7.39–7.27 (2m, 10H, H-12, H-13, H-14, H-15, H-16, H-19, H-20, H-21, H-22, H-23), 6.64, 6.39 (2s, 2 × 1H, H-7, H-6′), 5.20, 5.12, 5.07, 5.03 (d, *J* = 11.1 Hz, 1H, d, *J* = 11.9 Hz, 1H, d, *J* = 11.8 Hz, 1H, d, *J* = 10.6 Hz, 1H, H-10, H-17), 5.06, 4.94 (d, *J* = 15.8 Hz, 1H, d, *J* = 15.3 Hz, 1H, H-1-a, H-1′-a), 4.68, 4.63 (d, *J* = 15.8 Hz, 1H, d, *J* = 15.4 Hz, 1H, H-1-b, H-1′-b), 3.85, 3.72 (2s, 2 × 3H, H-9′, H-10′), 3.65–3.51 (m, 2H, H-3, H-3′), 2.30, 2.17, 2.01, 1.87, (dd, *J* = 16.3, 10.9 Hz, 1H, dd, *J* = 16.5, 11.4 Hz, 1H, dd, *J* = 16.4, 2.7 Hz, 1H, dd, *J* = 16.5, 1.5 Hz, 1H, H-4, H-4′), 1.22, 1.21 (d, *J* = 5.9 Hz, 3H, d, *J* = 5.9 Hz, 3H, H-9, H-11′); ^13^C NMR (100 MHz, CDCl_3_) *δ* = 156.3, 155.6, 148.8, 142.8, 138.1, 137.2, 134.1, 131.5, 128.8, 126.4, 120.1, 115.6 (12C, C-4a, C-5, C-7, C-8, C-8a, C-11, C-18, C-4a′, C-5′, C-7′, C-8′, C-8a′), 128.6, 128.4, 128.3, 127.5 (8C, C-12, C-13, C-15, C-16, C-19, C-20, C-22, C-23), 128.0 (2C C-14, C-21), 115.0, 92.7 (2C, C-7, C-6′), 74.3, 70.7 (2C, C-10′, C-17′), 70.8, 70.6 (2C, C-3, C-3′), 65.4, 64.8 (2C, C-1, C-1′), 56.0, 55.3 (2C, C-9′, C-10′), 35.0, 33.3 (2C, C-4, C-4′), 21.8, 21.7 (2C, C-11, C-9′). IR (KBr): 3446, 2967, 2931, 2836, 1598, 1454, 1316, 1209, 1122, 1078, 736, 697 cm^−1^. HRMS (ESI) calcd. for C_36_H_38_NaO_6_ [M+Na]^+^ 589.2561, found 589.2557.

##### General Procedure for Brønsted-Acid Catalyzed Oxa-Pictet–Spengler Reaction by Aromatic Aldehydes

To the solution of the corresponding *bis*(propan-2-ol) derivative (1.0 equiv.) in MeOH toluene was added (toluene/MeOH 4:1). Then aromatic aldehyde (6.0 equiv) and (1*S*)-(+)-10-camphorsulfonic acid (1.0 equiv.) were added. The reaction mixture was stirred at 80 °C until the starting material and the mono-cyclized intermediate products were consumed (ca. 8–16 heating hours of 24–48 h stirring) on the basis of TLC monitoring. After that, a saturated solution of NaHCO_3_ was added to the reaction and the mixture was stirred for 10 min, then it was concentrated in *vacuo*. The suspension was diluted with EtOAc and the mixture was extracted with water. The aqueous phase was washed three times with EtOAc. The combined organic phases were washed with brine, dried over anhydrous MgSO_4_, filtered, and the solvent was evaporated in *vacuo*. The residue was purified by flash chromatography and preparative chiral HPLC to yield the 5,5′-linked *bis*-isochroman target derivatives.

**(a*S*,1*R*,3*S*,1′*R*,3′*S*)-1,1′-*bis*(4-fluorophenyl)-6′,8′-dimethoxy-3,3′-dimethyl-[5,5′-*bis*-isochroman]-7,8-diol [*cis*,*cis*-(a*S*,1*R*,3*S*,1′*R*,3′*S*)-20]**

Flash chromatography: hexanes/acetone 4:1. *cis*,*cis*-(a*S*,1*R*,3*S*,1′*R*,3′*S*)-**20**: 234 mg (yield: 59%) white crystals, mp 225–228 °C. R_f_ = 0.53 (hexanes/acetone 1.5:1). [α${]}_{D}^{20}$ −147 (*c* = 0.25; CHCl_3_). ECD: (*c* = 1.25 × 10^−4^ M; MeCN) λ [nm], (Δε) = 294 (−2.21), 251sh (−5.36), 223 (−28.04), 215sh (−14.91), 199 (0.68), 193 (−12.64). Single crystals were grown in CHCl_3_:MeOH 4:1 at room temperature. ^1^H NMR (400 MHz, CDCl_3_) *δ* = 7.45–7.35 (m, 2H, H-11, H-15), 7.30–7.21 (m, 2H, H-13′, H-17′), 7.10–7.00 (m, 2H, H-12, H-14), 6.92–6.82 (m, 2H, H-14′, H-16′), 6.40 (s, 1H, H-6), 6.36 (s, 1H, H-7′), 5.88 (s, 1H, H-1′), 5.85 (s, 1H, H-1), 5.72 (bs, 1H, OH), 4.67 (bs, 1H, OH), 3.82–3.70 (m, 2H, H-3, H-3′), 3.75 (s, 3H, H-9′), 3.52 (s, 3H, H-10′), 2.55 (dd, *J* = 16.3, 10.4 Hz, 1H, H-4′_ax_), 2.47 (dd, *J* = 15.6, 10.4 Hz, 1H, H-4_ax_), 2.10–2.00 (m, 2H, H-4_eq_, H-4′_eq_), 1.23 (d, *J* = 5.8 Hz, 2 × 3H, H-9, H-11′); ^13^C NMR (100 MHz, CDCl_3_) *δ* = 162.7 (d, *J_C-F_* = 246.6 Hz, C-13), 163.2 (d, *J_C–F_* = 245.0 Hz, C-15′), 157.1 (1C, C-6′), 156.5 (1C, C-8′), 141.7, 139.4 (2C, C-7, C-8), 139.7 (d, *J_C–F_* = 2.6 Hz, 1C, C-12′), 138.0 (d, *J_C–F_* = 2.7 Hz, 1C, C-10), 135.6 (1C, C-4a′), 130.5 (d, *J_C–F_* = 8.2 Hz, 2C, C-11, C-15), 129.7 (d, *J_C–F_* = 8.1 Hz, 2C, C-13′, C-17′), 127.3 (1C, C-4a), 127.2 (1C, C-5), 123.9 (1C, C-8a), 120.2 (1C, C-5′), 118.4 (C-8a′), 116.6 (1C, C-6), 115.7 (d, *J_C–F_* = 21.5 Hz, 2C, C-12, C-14), 114.9 (d, *J_C–F_* = 21.4 Hz, 2C, C-14′, C-16′), 94.1 (C-7′), 77.2 (1C, C-1′), 77.0 (1C, C-1), 71.1 (1C, C-3), 70.7 (1C, C-3′), 56.0 (1C, C-9′), 55.3 (1C, C-10′), 36.9 (1C, C-4′), 34.5 (1C, C-4), 21.9 (1C, C-9), 21.6 (1C, C-11′). IR (KBr): 3476, 2972, 2934, 2841, 1595, 1509, 1321, 1224, 1208, 1114, 830, 556 cm^−1^. HRMS (ESI) calcd. for C_34_H_32_F_2_NaO_6_ [M+Na]^+^ 597.2059, found 597.2054.

**(a*S*,1*R*,3*S*,1′*R*,3′*S*)-1,1′-*bis*(4-bromophenyl)-6′,8′-dimethoxy-3,3′-dimethyl-[5,5′-*bis*-isochroman]-7,8-diol [*cis*,*cis*-(a*S*,1*R*,3*S*,1′*R*,3′*S*)-21**]

Flash chromatography: hexanes/acetone 4:1. *cis*,*cis*-(a*S*,1*R*,3*S*,1′*R*,3′*S*)-**21**: 317 mg (yield: 66%) white crystals, mp 213–215 °C. R_f_ = 0.69 (hexanes/acetone 1.5:1). [α${]}_{D}^{20}$ −158 (*c* = 0.20; CHCl_3_). ECD: (*c* = 7.69 × 10^−5^ M; MeCN) λ [nm], (Δε) = 293 (−2.93), 272 (−0.19), 230 (−49.41), 214 (7.19), 207sh (1.30), 204 (−3.14), 192 (41.01). Single crystals were grown in MeOH:H_2_O 5:2 at room temperature. ^1^H NMR (700 MHz, acetonitrile-*d*_3_) *δ* = 7.53–7.50 (m, 2H, H-12, H-14), 7.50–7.47 (m, 2H, H-14′, H-16′), 7.27–7.26 (m, 2H, H-13′, H-17′), 7.26–7.24 (m, 2H, H-11, H-15), 6.62 (s, 1H, H-6), 6.52 (s, 1H, H-7′), 5.89 (s, 1H, H-1), 5.81 (s, 1H, H-1′), 3.79 (s, 3H, H-9′), 3.77–3.72 (m, 1H, H-3), 3.72–3.67 (m, 1H, H-3′), 3.57 (s, 3H, H-10′), 2.49 (dd, *J* = 16.1, 10.8 Hz, 1H, H-4′_ax_), 2.39 (ddd, *J* = 15.8, 10.9, 0.8 Hz, 1H, H-4_ax_), 2.09 (dd, *J* = 15.7 Hz, 1H, H-4′_eq_), 2.06 (dd, *J* = 15.8, 1.0 Hz, 1H, H-4_eq_), 1.18 (d, *J* = 6.1 Hz, 3H, H-11′), 1.16 (d, *J* = 6.1 Hz, 3H, H-9); ^13^C NMR (175 MHz, acetonitrile-*d*_3_) *δ* = 158.1 (1C, C-6′), 157.3 (1C, C-8′), 145.0 (1C, C-12′), 144.2 (1C, C-10), 142.6, 141.2 (2C, C-7, C-8), 136.7 (1C, C-4a′), 131.8 (2C, C-12, C-14), 131.6 (2C, C-14′, C-16′), 131.3 (2C, C-11, C-15), 131.1 (2C, C-13′, C-17′), 128.2 (1C, C-4a), 127.8 (1C, C-5), 125.0 (1C, C-8a), 121.4 (1C, C-13), 121.1 (1C, C-15′), 120.8 (1C, C-5′), 118.7 (1C, C-8a′), 117.4 (1C, C-6), 95.0 (1C, C-7′), 77.5 (1C, C-1′), 77.3 (1C, C-1), 71.2 (1C, C-3), 70.9 (1C, C-3′), 56.3 (1C, C-9′), 55.8 (1C, C-10′), 37.0 (1C, C-4′), 35.2 (1C, C-4), 21.8 (2C, C-9, C-11′). IR (KBr): 3433, 2970, 2929, 1594, 1485, 1321, 1208, 1071, 1012, 817 cm^−1^. HRMS (ESI) calcd. for C_34_H_32_Br_2_NaO_6_ [M+Na]^+^ 717.0458, found 717.0453.

**(a*S*,1*R*,3*S*,1′*S*,3′*S*)-1,1′-*bis*(4-bromophenyl)-6′,8′-dimethoxy-3,3′-dimethyl-[5,5′-*bis*-isochroman]-7,8-diol [*cis*,*trans*-(a*S*,1*R*,3*S*,1′*S*,3′*S*)-21]**

Flash chromatography: hexanes/acetone 4:1. HPLC: Lux i-Cellulose-5 (150 × 21.2 mm), *n*-heptane/2-PrOH 90:10, 215 nm, t_R, prep_ = 3.56 min. *cis*,*trans*-(a*S*,1*R*,3*S*,1′*S*,3′*S*)-**21**: 27 mg (yield: 8%) white-beige crystals, mp 117–120 °C. R_f_ = 0.62 (hexanes/acetone 1.5:1). [α${]}_{D}^{20}$ −63 (*c* = 0.15; CHCl_3_). ECD: (*c* = 1.05 × 10^−4^ M; MeCN) λ [nm], (Δε) = 284sh (−2.69), 237 (−35.86), 217 (21.74), 205 (9.99), 197 (28.20). ^1^H NMR (700 MHz, acetonitrile-*d*_3_) *δ* = 7.56–7.48 (m, 4H, H-12, H-14, H-14′, H-16′), 7.29–7.22 (m, 2H, H-11, H-15), 7.18–7.10 (m, 2H, H-13′, H-17′), 6.65 (s, 1H, H-7′), 6.53 (s, 1H, H-6), 5.93 (s, 1H, H-1′), 5.88 (s, 1H, H-1), 3.83 (s, 3H, H-9′), 3.77–3.73 (m, 1H, H-3), 3.73 (s, 3H, H-10′), 3.54–3.49 (m, 1H, H-3′), 2.37 (ddd, *J* = 16.0, 10.8, 1.5 Hz, 1H, H-4_ax_), 2.33 (dd, *J* = 17.0, 11.1 Hz, 1H, H-4′_ax_), 2.16 (dd, *J* = 16.0, 1.0 Hz, 1H, H-4_eq_), 2.04 (dd, *J* = 17.0, 3.3 Hz, 1H, H-4′_eq_), 1.19 (d, *J* = 6.1 Hz, 3H, H-9), 1.05 (d, *J* = 6.1 Hz, 3H, H-11′); ^13^C NMR (175 MHz, acetonitrile-*d*_3_) *δ* = 158.4 (1C, C-6′), 157.1 (1C, C-8′), 144.3 (1C, C-10), 143.0 (1C, C-12′), 142.7, 141.2 (2C, C-7, C-8), 135.8 (1C, C-4a′), 131.8 (4C, C-12, C-14, C-14′, C-16′), 131.4 (2C, C-13′, C-17′), 131.3 (2C, C-11, C-15), 127.9 (1C, C-4a), 127.7 (1C, C-5), 125.0 (1C, C-8a), 121.6 (1C, C-15′), 121.4 (1C, C-13), 121.0 (1C, C-5′), 117.0 (1C, C-6), 116.2 (1C, C-8a′), 94.4 (1C, C-7′), 77.4 (1C, C-1), 73.7 (1C, C-1′), 71.2 (1C, C-3), 64.2 (1C, C-3′), 56.5 (1C, C-9′), 56.1 (1C, C-10′), 35.7 (1C, C-4), 35.1 (1C, C-4′), 21.9 (1C, C-9), 21.8 (1C, C-11′). IR (KBr): 3434, 2968, 2928, 1595, 1485, 1321, 1207, 1118, 1071, 1011, 816 cm^−1^. HRMS (ESI) data was identical with that of the *cis*,*cis*-(a*S*,1*R*,3*S*,1′*R*,3′*S*)-**21** stereoisomer.

**(a*S*,1*S*,3*S*,1′*R*,3′*S*)-1,1′-*bis*(4-bromophenyl)-6′,8′-dimethoxy-3,3′-dimethyl-[5,5′-*bis*-isochroman]-7,8-diol [*trans*,*cis*-(a*S*,1*S*,3*S*,1′*R*,3′*S*)-21]**

Flash chromatography: hexanes/acetone 4:1. HPLC: Lux i-Cellulose-5 (150 × 21.2 mm), *n*-heptane/2-PrOH 90:10, 215 nm, t_R, prep_ = 5.67 min. *trans*,*cis*-(a*S*,1*S*,3*S*,1′*R*,3′*S*)-**21**: 20 mg (yield: 6%) white-beige crystals, mp 211–214 °C. R_f_ = 0.62 (hexanes/acetone 1.5:1). [α${]}_{D}^{20}$ −40, (*c* = 0.22; CHCl_3_). ECD: (*c* = 8.46 × 10^−5^ M; MeCN) λ [nm], (Δε) = 292 (−3.94), 251 (4.51), 228 (−40.42), 214 (1.88), 205 (−9.15), 195 (49.08). ^1^H NMR (700 MHz, acetonitrile-*d*_3_) *δ* = 7.44–7.42 (m, 2H, H-12, H-14), 7.41–7.38 (m, 2H, H-14′, H-16′), 7.17–7.14 (m, 2H, H-13′, H-17′), 7.09–7.06 (m, 2H, H-11, H-15), 6.56 (s, 1H, H-6), 6.40 (s, 1H, H-7′), 5.90 (s, 1H, H-1), 5.69 (s, 1H, H-1′), 3.64 (s, 3H, H-9′), 3.63–3.59 (m, 1H, H-3′), 3.46 (s, 3H, H-10′), 3.45–3.41 (m, 1H, H-3), 2.36 (dd, *J* = 16.2, 10.8 Hz, 1H, H-4′_ax_), 2.09 (dd, *J* = 17.0, 11.5 Hz, 1H, H-4_ax_), 2.06 (dd, *J* = 16.5, 0.9 Hz, 1H, H-4′_eq_), 1.91 (dd, *J* = 16.6, 3.5 Hz, 1H, H-4_eq_), 1.10 (d, *J* = 6.1 Hz, 3H, H-11′), 0.93 (d, *J* = 6.2 Hz, 3H, H-9); ^13^C NMR (175 MHz, acetonitrile-*d*_3_) *δ* = 157.8 (1C, C-6′), 157.2 (1C, C-8′), 145.0 (1C, C-10), 142.4, 141.1 (2C, C-7, C-8), 142.3 (1C, C-12′), 136.6 (1C, C-4a′), 131.9 (2C, C-12, C-14), 131.6 (4C, C-14′, C-16′, C-11, C-15), 131.1 (2C, C-13′, C-17′), 128.5 (1C, C-5), 126.6 (1C, C-4a), 123.1 (1C, C-8a), 121.8 (1C, C-13), 121.1 (1C, C-15′), 120.8 (1C, C-5′), 118.7 (1C, C-8a′), 117.6 (1C, C-6), 94.9 (1C, C-7′), 77.5 (1C, C-1′), 73.8 (1C, C-1), 70.9 (1C, C-3′), 64.2 (1C, C-3), 56.2 (1C, C-9′), 55.8 (1C, C-10′), 36.6 (1C, C-4′), 33.7 (1C, C-4), 21.8 (2C, C-9, C-11′). IR (KBr): 3328, 2925, 1592, 1484, 1451, 1306, 1207, 811, 486 cm^−1^. HRMS (ESI) data was identical with that of the *cis*,*cis*-(a*S*,1*R*,3*S*,1′*R*,3′*S*)-**21** stereoisomer.

**(a*R*,1*R*,3*S*,1′*R*,3′*S*)-1,1′-*bis*(4-bromophenyl)-6′,8′-dimethoxy-3,3′-dimethyl-[5,5′-*bis*-isochroman]-7,8-diol [*cis*,*cis*-(a*R*,1*R*,3*S*,1′*R*,3′*S*)-21**]

Flash chromatography: hexanes/acetone 4:1. HPLC: Lux i-Cellulose-5 (150 × 21.2 mm), *n*-hexane/(MeOH:2-PrOH 1:1) 80:20, 254 nm, t_R, prep_ = 7.30 min. *cis*,*cis*-(a*R*,1*R*,3*S*,1′*R*,3′*S*)-**21**: 15 mg (yield: 2%) white-beige crystals, mp 108–111 °C. R_f_ = 0.58 (hexanes/acetone 1.5:1). [α${]}_{D}^{20}$ −186 (*c* = 0.08; CHCl_3_). ECD: (*c* = 1.34 × 10^−4^ M; MeCN) λ [nm], (Δε) = 292 (−2.06), 230 (−77.69), 213 (−8.00), 207 (−15.18), 195 (66.65). ^1^H NMR (700 MHz, acetonitrile-*d*_3_) *δ* = 7.45–7.43 (m, 2H, H-12, H-14), 7.43–7.40 (m, 2H, H-14′, H-16′), 7.21–7.18 (m, 2H, H-11, H-15), 7.18–7.16 (m, 2H, H-13′, H-17′), 6.46 (s, 1H, H-7′), 6.45 (s, 1H, H-6), 5.85 (s, 1H, H-1), 5.76 (s, 1H, H-1′), 3.71–3.67 (m, 2H, H-3, H-3′), 3.66 (s, 3H, H-9′), 3.52 (s, 3H, H-10′), 2.36–2.31 (m, 3H, H-4, H-4′_ax_), 2.15–2.14 (m, 1H, H-4′_eq_), 1.15 (d, *J* = 6.2 Hz, 3H, H-11′), 1.15 (d, *J* = 6.2 Hz, 3H, H-9); ^13^C NMR (175 MHz, acetonitrile-*d*_3_) *δ* = 157.6 (1C, C-6′), 157.2 (1C, C-8′), 145.0 (1C, C-12′), 144.1 (1C, C-10), 142.7, 141.2 (2C, C-7, C-8), 137.4 (1C, C-4a′), 131.8 (2C, C-12, C-14), 131.6 (2C, C-14′, C-16′), 131.3 (2C, C-11, C-15), 131.1 (2C, C-13′, C-17′), 128.1 (2C, C-5, C-4a), 125.3 (1C, C-8a), 121.4 (1C, C-13), 121.1 (1C, C-15′), 120.9 (1C, C-5′), 118.8 (1C, C-8a′), 116.9 (1C, C-6), 95.3 (1C, C-7′), 77.3 (2C, C-1, C-1′), 71.2 (1C, C-3), 70.8 (1C, C-3′), 56.3 (1C, C-9′), 55.8 (1C, C-10′), 35.5 (1C, C-4′), 35.2 (1C, C-4), 21.8 (2C, C-9, C-11′). IR (KBr): 3356, 2968, 2927, 2853, 1593, 1484, 1321, 1208, 815, 736 cm^−1^. HRMS (ESI) data was identical with that of the *cis*,*cis*-(a*S*,1*R*,3*S*,1′*R*,3′*S*)-**21** stereoisomer.

**(a*S*,1*R*,3*S*,1′*R*,3′*S*)-6′,8′-dimethoxy-3,3′-dimethyl-1,1′-*bis*(3,4,5-trimethoxyphenyl)-[5,5′-*bis*-isochroman]-7,8-diol [*cis*,*cis*-(a*S*,1*R*,3*S*,1′*R*,3′*S*)-22]**

Flash chromatography: CHCl_3_/MeOH 90:0.5. HPLC: Lux i-Amylose-5 (150 × 10 mm), *n*-heptane/(MeOH:2-PrOH 1:1) 80:20, 230 nm, t_R, prep_ = 5.07 min. *cis*,*cis*-(a*S*,1*R*,3*S*,1′*R*,3′*S*)-**22**: 10 mg (yield: 5%) white-beige crystals, mp 113–117 °C. R_f_ = 0.20 (CHCl_3_/MeOH 90:1). [α${]}_{D}^{20}$ −140 (*c* = 0.21; CHCl_3_). ECD: (*c* = 7.30 × 10^−5^ M; MeCN) λ [nm], (Δε) = 292 (−2.27), 234sh (−40.98), 220 (−48.86), 201sh (34.73), 198 (43.62). ^1^H NMR (700 MHz, CDCl_3_) *δ* = 6.70 (s, 2H, H-11, H-15), 6.68 (s, 1H, H-6), 6.55 (s, 2H, H-13′, H-17′), 6.36 (s, 1H, H-7′), 5.81 (s, 1H, H-1′), 5.78 (s, 1H, H-1), 5.46 (bs, 1H, OH), 4.54 (bs, 1H, OH), 3.86 (s, 6H, H-16, H-18), 3.85 (s, 3H, H-17), 3.81 (s, 6H, H-18′, H-20′), 3.80 (s, 3H, H-19′), 3.83–3.78 (m, 1H, H-3), 3.73 (s, 3H, H-9′), 3.74–3.69 (m, 1H, H-3′), 3.55 (s, 3H, H-10′), 2.58 (dd, *J* = 16.3, 10.9 Hz, 1H, H-4′_ax_), 2.50 (ddd, *J* = 16.3, 11.1, 1.5 Hz, 1H, H-4_ax_), 2.11–2.06 (m, 1H, H-4′_eq_), 2.09–2.06 (m, 1H, H-4_eq_), 1.27 (d, *J* = 6.1 Hz, 6H, H-9, H-11′); ^13^C NMR (175 MHz, CDCl_3_) *δ* = 156.9 (1C, C-6′), 156.8 (1C, C-8′), 154.0 (2C, C-12, C-14), 152.9 (2C, C-14′, C-16′), 142.8, 138.8 (2C, C-7, C-8), 139.9 (1C, C-12′), 138.7 (1C, C-13), 137.4 (1C, C-15′), 136.6 (1C, C-10), 135.8 (1C, C-4a′), 128.3 (1C, C-5), 127.0 (1C, C-4a), 124.2 (1C, C-8a), 120.2 (1C, C-5′), 118.8 (1C, C-8a′), 116.7 (1C, C-6), 105.7 (2C, C-11, C-15), 105.5 (2C, C-13′, C-17′), 94.3 (1C, C-7′), 78.2 (1C, C-1), 78.0 (1C, C-1′), 71.5 (1C, C-3), 70.6 (1C, C-3′), 60.9 (2C, C-17, C-19′), 56.2 (4C, C-18′, C-20′, C-16, C-18), 56.0 (1C, C-9′), 55.5 (1C, C-10′), 36.6 (1C, C-4′), 34.3 (1C, C-4), 21.9 (2C, C-9, C-11′). IR (KBr): 3442, 2966, 2935, 2838, 1593, 1505, 1462, 1421, 1328, 1231, 1124, 1009, 831, 734 cm^−1^. HRMS (ESI) data was identical with that of the *cis*,*trans*-(a*S*,1*R*,3*S*,1′*S*,3′*S*)-**22** stereoisomer.

**(a*S*,1*R*,3*S*,1′*S*,3′*S*)-6′,8′-dimethoxy-3,3′-dimethyl-1,1′-*bis*(3,4,5-trimethoxyphenyl)-[5,5′-*bis*-isochroman]-7,8-diol [*cis*,*trans*-(a*S*,1*R*,3*S*,1′*S*,3′*S*)-22]**

Flash chromatography: CHCl_3_/MeOH 90:0.5. HPLC: Lux i-Amylose-5 (150 × 10 mm), *n*-heptane/(MeOH:2-PrOH 1:1) 80:20, 230 nm, t_R, prep_ = 8.66 min. *cis*,*trans*-(a*S*,1*R*,3*S*,1′*S*,3′*S*)-**22**: 23 mg (yield: 12%) white-beige crystals, mp 118–121 °C. R_f_ = 0.20 (CHCl_3_/MeOH 90:1). [α${]}_{D}^{20}$ −68 (*c* = 0.23; CHCl_3_). Crystals were grown in CHCl_3_:hexanes 1:3 at room temperature. ECD: (*c* = 8.80 × 10^−5^ M; MeCN) λ [nm], (Δε) = 278sh (−3.15), 240 (−37.15), 219 (−5.88), 208 (48.10), 196 (13.84). ^1^H NMR (700 MHz, CDCl_3_) *δ* = 6.67 (s, 2H, H-11, H-15), 6.62 (s, 1H, H-6), 6.47 (s, 1H, H-7′), 6.42 (s, 2H, H-13′, H-17′), 6.04 (s, 1H, H-1′), 5.83 (s, 1H, H-1), 4.93 (bs, 1H, OH), 3.86 (s, 6H, H-16, H-18), 3.85 (s, 3H, H-19′), 3.84 (s, 3H, H-17), 3.79–3.74 (m, 1H, H-3), 3.77 (s, 3H, H-9′), 3.77 (s, 6H, H-18′, H-20′), 3.73 (s, 3H, H-10′), 3.70–3.65 (m, 1H, H-3′), 2.49 (ddd, *J* = 15.9, 11.2, 1.1 Hz, 1H, H-4_ax_), 2.46 (dd, *J* = 15.9, 11.2 Hz, 1H, H-4′_ax_), 2.08–2.01 (m, 2H, H-4_eq_, H-4′_eq_), 1.21 (d, *J* = 6.2 Hz, 3H, H-9), 1.12 (d, *J* = 6.1 Hz, 3H, H-11′); ^13^C NMR (175 MHz, CDCl_3_) *δ* = 157.4 (1C, C-6′), 156.3 (1C, C-8′), 153.6 (2C, C-12, C-14), 152.9 (2C, C-14′, C-16′), 142.3, 139.6 (2C, C-7, C-8), 138.1 (1C, C-13), 137.7 (1C, C-12′), 137.6 (1C, C-10), 137.4 (1C, C-15′), 134.6 (1C, C-4a′), 127.2 (1C, C-5), 126.6 (1C, C-4a), 124.1 (1C, C-8a), 120.3 (1C, C-5′), 116.1 (1C, C-8a′), 116.0 (1C, C-6), 105.8 (2C, C-13′, C-17′), 105.7 (2C, C-11, C-15), 93.5 (1C, C-7′), 78.0 (1C, C-1), 73.8 (1C, C-1′), 71.0 (1C, C-3), 64.0 (1C, C-3′), 60.9 (2C, C-19′, C-17), 56.2 (1C, C-9′), 56.1 (4C, C-16, C-18, C-18′, C-20′), 55.6 (1C, C-10′), 35.5 (1C, C-4′), 34.5 (1C, C-4), 22.0 (1C, C-9), 21.7 (1C, C-11′). IR (KBr): 3435, 2966, 2935, 2837, 1595, 1505, 1462, 1419, 1324, 1233, 1207, 1125, 1008 cm^−1^. HRMS (ESI) calcd. for C_40_H_46_NaO_12_ [M+Na]^+^ 741.2881, found 741.2881.

**(a*S*,1*S*,3*S*,1′*R*,3′*S*)-6′,8′-dimethoxy-3,3′-dimethyl-1,1′-*bis*(3,4,5-trimethoxyphenyl)-[5,5′-*bis*-isochroman]-7,8-diol [*trans*,*cis*-(a*S*,1*S*,3*S*,1′*R*,3′*S*)-22]**

Flash chromatography: CHCl_3_/MeOH 90:0.5. HPLC: Lux i-Amylose-5 (150 × 10 mm), *n*-heptane/(MeOH:2-PrOH 1:1) 80:20, 254 nm, t_R, prep_ = 4.98 min. *trans*,*cis*-(a*S*,1*S*,3*S*,1′*R*,3′*S*)-**22**: 9 mg (yield: 5%) white-beige crystals, mp 133–136 °C. R_f_ = 0.15 (CHCl_3_/MeOH 90:1). [α${]}_{D}^{20}$ −29 (*c* = 0.19; CHCl_3_). ECD: (*c* = 8.09 × 10^−5^ M; MeCN) λ [nm], (Δε) = 290 (−4.18), 272sh (−0.56), 252 (4.96), 231 (−26.97), 203 (47.32). ^1^H NMR (700 MHz, CDCl_3_) *δ* = 6.65 (s, 1H, H-6), 6.54 (s, 2H, H-13′, H-17′), 6.51 (s, 2H, H-11, H-15), 6.37 (s, 1H, H-7′), 6.04 (s, 1H, H-1), 5.80 (s, 1H, H-1′), 5.07 (bs, 1H, OH), 3.85 (s, 3H, H-17), 3.80 (s, 3H, H-19′), 3.78 (s, 6H, H-18′, H-20′), 3.76 (s, 6H, H-16, H-18), 3.75 (s, 3H, H-9′), 3.74–3.70 (m, 1H, H-3′), 3.70–3.64 (m, 1H, H-3), 3.54 (s, 3H, H-10′), 2.53 (dd, *J* = 16.1, 11.0 Hz, 1H, H-4′_ax_), 2.33 (dd, *J* = 16.6, 11.1 Hz, 1H, H-4_ax_), 2.03 (d, *J* = 16.1 Hz, 1H, H-4′_eq_), 2.02 (dd, *J* = 16.6, 3.4 Hz, 1H, H-4_eq_), 1.19 (d, *J* = 6.2 Hz, 3H, H-11′), 1.13 (d, *J* = 6.1 Hz, 3H, H-9); ^13^C NMR (175 MHz, CDCl_3_) *δ* = 156.9 (1C, C-6′), 156.8 (1C, C-8′), 153.2 (2C, C-12, C-14), 153.0 (2C, C-14′, C-16′), 141.3 139.6 (2C, C-7, C-8), 139.7 (1C, C-12′), 137.8 (1C, C-13), 137.6 (1C, C-15′), 136.7 (1C, C-10), 135.5 (1C, C-4a′), 128.1 (1C, C-5), 126.3 (1C, C-4a), 122.8 (1C, C-8a), 119.9 (1C, C-5′), 118.4 (1C, C-8a′), 117.0 (1C, C-6), 105.9 (2C, C-11, C-15), 105.6 (2C, C-13′, C-17′), 94.1 (1C, C-7′), 78.2 (1C, C-1′), 73.8 (1C, C-1), 70.4 (1C, C-3′), 64.0 (1C, C-3), 61.0 (1C, C-17), 60.9 (1C, C-19′), 56.3 (2C, C-18′, C-20′), 56.0 (2C, C-16, C-18), 55.8 (1C, C-9′), 55.6 (1C, C-10′), 36.4 (1C, C-4′), 33.2 (1C, C-4), 21.8 (1C, C-9), 21.7 (1C, C-11′). IR (KBr): 3448, 2965, 2934, 2836, 1593, 1505, 1461, 1419, 1324, 1232, 1209, 1125, 1067, 1007 cm^−1^. HRMS (ESI) data was identical with that of the *cis*,*trans*-(a*S*,1*R*,3*S*,1′*S*,3′*S*)-**22** stereoisomer.

**(a*S*,1*S*,3*S*,1′*S*,3′*S*)-6′,8′-dimethoxy-3,3′-dimethyl-1,1′-*bis*(3,4,5-trimethoxyphenyl)-[5,5′-*bis*-isochroman]-7,8-diol [*trans*,*trans*-(a*S*,1*S*,3*S*,1′*S*,3′*S*)-22]**

Flash chromatography: CHCl_3_/MeOH 90:0.5. HPLC: Lux i-Amylose-5 (150 × 10 mm), *n*-heptane/(MeOH:2-PrOH 1:1) 80:20, 254 nm, t_R, prep_ = 9.95 min. *trans*,*trans*-(a*S*,1*S*,3*S*,1′*S*,3′*S*)-**22**: 12 mg (yield: 6%) white-beige crystals, mp 111–114 °C. R_f_ = 0.15 (CHCl_3_/MeOH 90:1). [α${]}_{D}^{20}$ +25 (*c* = 0.21; CHCl_3_). ECD: (*c* = 8.36 × 10^−5^ M; MeCN) λ [nm], (Δε) = 290 (−1.80), 268 (0.36), 240 (−8.12), 219 (56.17), 210 (−7.29), 201 (37.95). ^1^H NMR (700 MHz, CDCl_3_) *δ* = 6.84 (bs, 1H, OH), 6.57 (s, 1H, H-6), 6.48 (s, 2H, H-11, H-15), 6.47 (s, 1H, H-7′), 6.40 (s, 2H, H-13′, H-17′), 6.09 (s, 1H, H-1), 6.04 (s, 1H, H-1′), 5.50 (bs, 1H, OH), 3.83 (s, 3H, H-19′), 3.82 (s, 3H, H-17), 3.80 (s, 3H, H-9′), 3.74 (s, 6H, H-18′, H-20′), 3.74 (s, 3H, H-10′), 3.73 (s, 6H, H-16, H-18), 3.69–3.62 (m, 2H, H-3′, H-3), 2.36 (dd, *J* = 17.2, 11.3 Hz, 1H, H-4′_ax_), 2.29 (dd, *J* = 16.7, 11.3 Hz, 1H, H-4_ax_), 1.99 (dd, *J* = 16.9, 3.3 Hz, 2H, H-4_eq_, H-4′_eq_), 1.07 (d, *J* = 6.1 Hz, 2 × 3H, H-9, H-11′); ^13^C NMR (175 MHz, CDCl_3_) *δ* = 157.2 (1C, C-6′), 156.4 (1C, C-8′), 153.1 (2C, C-12, C-14), 152.9 (2C, C-14′, C-16′), 141.2, 140.3 (2C, C-7, C-8), 137.7 (1C, C-13), 137.6 (1C, C-12′), 137.5 (1C, C-15′), 136.7 (1C, C-10), 134.4 (1C, C-4a′), 127.6 (1C, C-5), 125.7 (1C, C-4a), 122.7 (1C, C-8a), 120.1 (1C, C-5′), 116.6 (1C, C-6), 116.1 (1C, C-8a′), 105.8 (4C, C-11, C-15, C-13′, C-17′), 93.2 (1C, C-7′), 73.8 (2C, C-1, C-1′), 63.8 (2C, C-3, C-3′), 60.9 (2C, C-19′, C-17), 56.1 (2C, C-18′, C-20′), 56.0 (2C, C-16, C-18), 55.9 (1C, C-9′), 55.6 (1C, C-10′), 35.1 (1C, C-4′), 33.2 (1C, C-4), 21.9 (1C, C-9), 21.7 (1C, C-11′). IR (KBr): 3420, 2965, 2934, 2836, 1593, 1505, 1461, 1418, 1322, 1233, 1207, 1125, 1062, 1008 cm^−1^. HRMS (ESI) data was identical with that of the *cis*,*trans*-(a*S*,1*R*,3*S*,1′*S*,3′*S*)-**22** stereoisomer.

**(a*R*,1*S*,3*S*,1′*S*,3′*S*)-1,1′-*bis*(benzo[*d*][1,3]dioxol-5-yl)-6′,8′-dimethoxy-3,3′-dimethyl-[5,5′-*bis*-isochroman]-7,8-diol [*trans*,*trans*-(a*R*,1*S*,3*S*,1′*S*,3′*S*)-23]**

Flash chromatography: CHCl_3_/hexanes 10:0.6 → CHCl_3_. HPLC: Lux i-Cellulose-5 (150 × 21.2 mm), *n*-hexane/(MeOH:2-PrOH = 1:3) 80:20, 296 nm, t_R, prep_ = 10.77 min. *trans*,*trans*-(a*R*,1*S*,3*S*,1′*S*,3′*S*)-**23**: 5 mg (yield: 3%) white-yellow crystals, mp 131–133 °C. R_f_ = 0.33 (CHCl_3_/MeOH 10:0.2). [α${]}_{D}^{20}$ +36 (*c* = 0.17; CHCl_3_). ECD: (*c* = 9.26 × 10^−5^ M; MeCN) λ [nm], (Δε) = 283 (−1.38), 264 (−0.36), 245 (−6.47), 231 (4.08), 211 (20.74), 205sh (11.31), 195 (−1.58), 194 (−3.16). ^1^H NMR (700 MHz, CDCl_3_) *δ* = 6.87 (d, *J* = 1.1 Hz, 1H, H-11), 6.82 (dd, *J* = 8.0, 1.1 Hz, 1H, H-16), 6.79 (d, *J* = 8.0 Hz, 1H, H-15), 6.77 (d, *J* = 1.1 Hz, 1H, H-13′), 6.74 (d, *J* = 8.0 Hz, 1H, H-17′), 6.66 (dd, *J* = 8.0, 1.1 Hz, 1H, H-18′), 6.64 (s, 1H, H-6), 6.43 (s, 1H, H-7′), 5.98 (s, 2H, H-13), 5.96–5.94 (m, 2H, H-15′), 5.94 (2s, 2 × 1H, H-1, H-1′), 5.20 (bs, 1H, OH), 4.74 (bs, 1H, OH), 3.77 (s, 3H, H-9′), 3.76–3.73 (m, 1H, H-3), 3.72 (s, 3H, H-10′), 3.69–3.64 (m, 1H, H-3′), 2.39 (dd, *J* = 17.1, 3.3 Hz, 1H, H-4′_eq_), 2.24 (dd, *J* = 16.5, 3.3 Hz, 1H, H-4_eq_), 2.08 (dd, *J* = 16.5, 10.7 Hz, 1H, H-4_ax_), 2.03 (dd, *J* = 17.1, 11.0 Hz, 1H, H-4′_ax_), 1.11 (d, *J* = 6.1 Hz, 1H, H-9), 1.09 (d, *J* = 6.1 Hz, 1H, H-11′); ^13^C NMR (175 MHz, CDCl_3_) *δ* = 156.5 (1C, C-6′), 156.3 (1C, C-8′), 148.0, 147.7 (2C, C-11a, C-14a), 147.5, 146.8 (2C, C-13a′, C-16a′), 141.5, 138.9 (2C, C-7, C-8), 136.7 (1C, C-12′), 135.5 (1C, C-4a′), 134.8 (1C, C-10), 128.9 (1C, C-5), 126.9 (1C, C-4a), 123.3 (1C, C-8a), 122.6 (1C, C-16), 122.1 (1C, C-18′), 120.6 (1C, C-5′), 116.8 (1C, C-8a′), 116.3 (1C, C-6), 109.6 (1C, C-11), 109.2 (1C, C-13′), 108.2 (1C, C-15), 107.6 (1C, C-17′), 101.3 (1C, C-13), 101.1 (1C, C-15′), 93.1 (1C, C-7′), 73.7 (1C, C-1), 73.4 (1C, C-1′), 63.9 (1C, C-3), 63.4 (1C, C-3′), 56.1 (1C, C-9′), 55.6 (1C, C-10′), 34.1 (1C, C-4′), 33.3 (1C, C-4), 21.9 (1C, C-11′), 21.6 (1C, C-9). IR (KBr): 3291, 2968, 2928, 2896, 1710, 1594, 1502, 1487, 1438, 1319, 1287, 1234, 1040, 935, 737 cm^−1^. HRMS (ESI) data was identical with that of the *trans*,*trans*-(a*S*,1*S*,3*S*,1′*S*,3′*S*)-**23** stereoisomer.

**(a*S*,1*S*,3*S*,1′*R*,3′*S*)-1,1′-*bis*(benzo[d][1,3]dioxol-5-yl)-6′,8′-dimethoxy-3,3′-dimethyl-[5,5′-*bis*-isochroman]-7,8-diol [*trans*,*cis*-(a*S*,1*S*,3*S*,1′*R*,3′*S*)-23]**

Flash chromatography: CHCl_3_/hexanes 10:0.6 → CHCl_3_. HPLC: Lux i-Cellulose-5 (150 × 21.2 mm), *n*-hexane/(MeOH:2-PrOH = 1:3) 80:20, 296 nm, t_R, prep_ = 16.04 min. *trans*,*cis*-(a*S*,1*S*,3*S*,1′*R*,3′*S*)-**23**: 18 mg (yield: 10%) white-yellow crystals, mp 131–134 °C. R_f_ = 0.31 (CHCl_3_/MeOH 10:0.2). [α${]}_{D}^{20}$ −21 (*c* = 0.22; CHCl_3_). ECD: (*c* = 9.18 × 10^−5^ M; MeCN) λ [nm], (Δε) = 291 (−4.04), 249 (1.69), 234 (−11.72), 216 (−8.19), 198 (35.80). ^1^H NMR (700 MHz, CDCl_3_) *δ* = 6.82 (dd, *J* = 8.0, 1.5 Hz, 1H, H-18′), 6.80 (d, *J* = 1.5 Hz, 1H, H-11), 6.76 (d, *J* = 8.0 Hz, 1H, H-15), 6.74 (d, *J* = 1.5 Hz, 1H, H-13′), 6.71 (dd, *J* = 8.0, 1.5 Hz, 1H, H-16), 6.69 (d, *J* = 8.0 Hz, 1H, H-17′), 6.55 (s, 1H, H-6), 6.35 (s, 1H, H-7′), 6.00 (s, 1H, H-1), 5.97–5.94 (m, 2H, H-13), 5.90–5.86 (m, 2H, H-15′), 5.80 (s, 1H, H-1′), 5.22 (bs, 1H, OH), 3.74 (s, 3H, H-9′), 3.73–3.69 (m, 1H, H-3′), 3.69–3.63 (m, 1H, H-3), 3.55 (s, 3H, H-10′), 2.49 (dd, *J* = 16.5, 10.8 Hz, 1H, H-4′_ax_), 2.32 (dd, *J* = 16.7, 11.1 Hz, 1H, H-4_ax_), 2.05 (d, *J* = 16.1 Hz, 1H, H-4′_eq_), 1.99 (dd, *J* = 16.7, 3.3 Hz, 1H, H-4_eq_), 1.24 (d, *J* = 6.2 Hz, 3H, H-11′), 1.10 (d, *J* = 6.2 Hz, 3H, H-9); ^13^C NMR (175 MHz, CDCl_3_) *δ* = 156.7 (1C, C-6′), 156.6(1C, C-8′), 147.9, 147.4 (2C, C-11a, C-14a), 147.3, 146.8 (2C, C-13a′, C-16a′), 141.1, 139.9 (2C, C-7, C-8), 138.2 (1C, C-12′), 135.8 (1C, C-4a′), 135.2 (1C, C-10), 127.8 (1C, C-5), 126.5 (1C, C-4a), 122.7 (1C, C-8a), 122.3 (1C, C-16), 121.8 (1C, C-18′), 120.0 (1C, C-5′), 118.7 (1C, C-8a′), 117.2 (1C, C-6), 109.3 (1C, C-11), 108.6 (1C, C-13′), 108.0 (1C, C-15), 107.9 (1C, C-17′), 101.2 (1C, C-13), 100.9 (1C, C-15′), 94.0 (1C, C-7′), 77.5 (1C, C-1′), 73.6 (1C, C-1), 70.5 (1C, C-3′), 63.7 (1C, C-3), 55.7 (1C, C-9′), 55.5 (1C, C-10′), 36.5 (1C, C-4′), 33.3 (1C, C-4), 21.8 (2C, C-11′, C-9). IR (KBr): 3444, 2967, 2893, 1706, 1593, 1503, 1487, 1439, 1237, 1207, 1039, 934, 812 cm^−1^. HRMS (ESI) data was identical with that of the *trans*,*trans*-(a*S*,1*S*,3*S*,1′*S*,3′*S*)-**23** stereoisomer.

**(a*S*,1*S*,3*S*,1′*S*,3′*S*)-1,1′-*bis*(benzo[d][1,3]dioxol-5-yl)-6′,8′-dimethoxy-3,3′-dimethyl-[5,5′-*bis*-isochroman]-7,8-diol [*trans*,*trans*-(a*S*,1*S*,3*S*,1′*S*,3′*S*)-23**]

Flash chromatography: CHCl_3_/hexanes 10:0.6 → CHCl_3_. HPLC (not necessary): Lux i-Cellulose-5 (150 × 21.2 mm), *n*-hexane/(MeOH:2-PrOH = 1:3) 80:20, 296 nm, t_R, prep_ = 17.23 min. *trans*,*trans*-(a*S*,1*S*,3*S*,1′*S*,3′*S*)-**23**: 65 mg (yield: 36%) white-yellow crystals, mp 133–135 °C. R_f_ = 0.29 (CHCl_3_/MeOH 10:0.2). [α${]}_{D}^{20}$ +90 (*c* = 0.21; CHCl_3_). ECD: (*c* = 1.19 × 10^−4^ M; MeCN) λ [nm], (Δε) = 294 (3.51), 245 (−10.44), 210 (77.56), 200 (−24.38), 193 (−5.47). ^1^H NMR (700 MHz, CDCl_3_) *δ* = 7.37 (bs, 1H, OH), 6.79 (d, *J* = 1.6 Hz, 1H, H-11), 6.73 (d, *J* = 8.0 Hz, 1H, H-15), 6.71 (dd, *J* = 7.9, 1.4 Hz, 1H, H-16), 6.71 (d, *J* = 8.0 Hz, 1H, H-17′), 6.70 (d, *J* = 1.6 Hz, 1H, H-13′), 6.60 (dd, *J* = 8.0, 1.6 Hz, 1H, H-18′), 6.48 (s, 1H, H-6), 6.40 (s, 1H, H-7′), 6.04 (s, 1H, H-1), 6.01 (s, 1H, H-1′), 5.94–5.91 (m, 4H, H-13, H-15′), 5.41 (bs, 1H, OH), 3.75 (s, 3H, H-9′), 3.72–3.66 (m, 2H, H-3′, H-3), 3.69 (s, 3H, H-10′), 2.37 (dd, *J* = 17.3, 11.3 Hz, 1H, H-4′_ax_), 2.31 (dd, *J* = 16.7, 11.2 Hz, 1H, H-4_ax_), 2.06 (dd, *J* = 17.3, 3.5 Hz, 1H, H-4′_eq_), 2.03 (dd, *J* = 16.7, 3.4 Hz, 1H, H-4_eq_), 1.14 (d, *J* = 6.2 Hz, 3H, H-11′), 1.12 (d, *J* = 6.1 Hz, 3H, H-9); ^13^C NMR (175 MHz, CDCl_3_) *δ* = 157.1 (1C, C-6′), 156.1 (1C, C-8′), 147.7, 147.2 (2C, C-11a, C-14a), 147.5, 147.0 (2C, C-13a′, C-16a′), 141.3, 140.2 (2C, C-7, C-8), 135.9 (1C, C-12′), 135.5 (1C, C-10), 134.4 (1C, C-4a′), 127.3 (1C, C-5), 125.7 (1C, C-4a), 122.5 (1C, C-8a), 122.3 (1C, C-16), 122.1 (1C, C-18′), 120.3 (1C, C-5′), 116.1 (1C, C-6), 115.9 (1C, C-8a′), 109.4 (1C, C-11), 109.1 (1C, C-13′), 107.9 (1C, C-15), 107.8 (1C, C-17′), 101.1 (2C, C-13, C-15′), 93.2 (1C, C-7′), 73.6 (2C, C-1, C-1′), 63.9 (1C, C-3′), 63.6 (1C, C-3), 55.8 (1C, C-9′), 55.5 (1C, C-10′), 35.3 (1C, C-4′), 33.3 (1C, C-4), 21.7 (2C, C-9, C-11′). IR (KBr): 3434, 2968, 2897, 1595, 1502, 1487, 1438, 1321, 1235, 1207, 1040, 936, 814 cm^−1^. HRMS (ESI) calcd. for C_36_H_34_NaO_10_ [M+Na]^+^ 649.2044, found 649.2044.

**(a*S*)-4-[(1*R*,3*S*)-1-(4-fluorophenyl)-6,8-dimethoxy-3-methylisochroman-5-yl]-5-[(*S*)-2-hydroxypropyl]benzene-1,2-diol [*cis*-(a*S*,2*S*,1′*R*,3′*S*)-24]**

The mono-cyclization reaction was carried out by 4-fluorobenzaldeyde (1.2 equiv.) and (1*S*)-(+)-10-camphorsulfonic acid (0.5 equiv.). Flash chromatography: hexanes/acetone 1.5:1. *cis*-(a*S*,2*S*,1′*R*,3′*S*)-**24**: 91 mg (yield: 88%) white crystals, mp 88–91 °C. R_f_ = 0.58 (CH_2_Cl_2_/MeOH 10:1). [α${]}_{D}^{20}$ −40 (*c* = 0.24; CHCl_3_). ECD: (*c* = 1.66 × 10^−4^ M; MeCN) λ [nm], (Δε) = 305 (0.20), 296 (−1.14), 275 (0.51), 257sh (1.47), 249 (3.08), 231 (−9.92), 225 (−8.64), 220 (−10.39), 212 (−4.02), 204 (−5.75), 199 (−1.67). Crystals were grown in EtOAc at room temperature. ^1^H NMR (400 MHz, acetone-*d*_6_) *δ* = 7.70 (bs, 1H, OH), 7.35–7.27 (m, 2H, H-13′, H-17′), 7.05–6.97 (m, 2H, H-14′, H-16′), 6.84 (s, 1H, H-6), 6.57 (s, 1H, H-9), 6.53 (s, 1H, H-7′), 5.82 (s, 1H, H-1′), 3.73–3.65 (m, 2H, H-2, H-3′), 3.68 (s, 3H, H-9′), 3.55 (s, 3H, H-10′), 2.91 (bs, 1H, OH), 2.88 (bs, 1H, OH), 2.35–2.29 (m, 4H, H-1, H-4′), 1.14 (d, *J* = 6.1 Hz, 3H, H-11′), 0.92 (d, *J* = 6.1 Hz, 3H, H-3); ^13^C NMR (100 MHz, acetone-*d*_6_) *δ* = 162.6 (d, *J_C-F_* = 242.4 Hz, 1C, C-15′), 157.5 (1C, C-6′) 157.1 (1C, C-8′), 144.8, 144.0 (2C, C-7, C-8), 142.0 (d, *J_C-F_* = 2.8 Hz, 1C, C-12′), 136.7 (1C, C-4a′), 130.8 (d, 2C, *J_C-F_* = 8.0 Hz, C-13′, C-17′), 130.7 (1C, C-4), 128.8 (1C, C-5), 121.9 (1C, C-5′), 119.0 (1C, C-8a′), 118.7 (1C, C-9), 117.8 (1C, C-6), 114.9 (d, 2C, *J_C-F_* = 21.4 Hz, C-14′, C-16′), 94.8 (1C, C-7′), 77.2 (1C, C-1′), 70.7, 68.2 (2C, C-2, C-3′), 55.7 (1C, C-9′), 55.5 (1C, C-10′), 43.6 (1C, C-1), 36.9 (1C, C-4′), 23.6 (1C, C-3), 22.0 (1C, C-11′). IR (KBr): 3433, 2970, 1595, 1509, 1456, 1322, 1209, 830 cm^−1^. HRMS (ESI) calcd. for C_27_H_29_FNaO_6_ [M+Na]^+^ 491.1840, found 491.1840.

**(a*S*,1*S*,3*S*,1′*R*,3′*S*)-1-(benzo[*d*][1,3]dioxol-5-yl)-1′-(4-fluorophenyl)-6′,8′-dimethoxy-3,3′-dimethyl-[5,5′-*bis*-isochroman]-7,8-diol [*trans*,*cis*-(a*S*,1*S*,3*S*,1′*R*,3′*S*)-25]**

The second oxa-Pictet–Spengler reaction of the mono-cyclized 4-fluorophenyl derivate was carried out by piperonal (2.0 equiv.) and (1*S*)-(+)-10-camphorsulfonic acid (1.0 equiv.). Flash chromatography: CHCl_3_/MeOH 10:0.025 → 10:0.05 → 10:0.2. *trans*,*cis*-(a*S*,1*S*,3*S*,1′*R*,3′*S*)-**25**: 18 mg (yield 16%) beige-light brown crystals, mp 113–116 °C. R_f_ = 0.25 (CHCl_3_/MeOH 10:0.2). [α${]}_{D}^{20}$ −28 (*c* = 0.19; CHCl_3_). ECD: (*c* = 1.20 × 10^−4^ M; MeCN) λ [nm], (Δε) = 254 (0.47), 248 (1.04), 229 (−8.12), 220 (−13.91), 205sh (9.76), 200 (13.24). ^1^H NMR (700 MHz, CDCl_3_) *δ* = 7.28–7.23 (m, 2H, H-13′, H-17′), 6.93–6.87 (m, 2H, H-14′, H-16′), 6.80 (d, *J* = 1.7 Hz, 1H, H-11), 6.76 (d, *J* = 8.0 Hz, 1H, H-15), 6.72 (dd, *J* = 8.0, 1.7 Hz, 1H, H-16), 6.50 (s, 1H, H-6), 6.35 (s, 1H, H-7′), 6.00 (s, 1H, H-1), 5.97–5.93 (m, 2H, H-13), 5.85 (s, 1H, H-1′), 5.17 (bs, 1H, OH), 3.77–3.69 (m, 1H, H-3′), 3.74 (s, 3H, H-9′), 3.70–3.62 (m, 1H, H-3), 3.50 (s, 3H, H-10′), 2.51 (dd, *J* = 16.2, 10.9 Hz, 1H, H-4′_ax_) 2.33 (dd, *J* = 16.7, 11.2 Hz, 1H, H-4_ax_), 2.07 (d, *J* = 15.6 Hz, 1H, H-4′_eq_), 2.00 (dd, *J* = 16.7, 3.3 Hz, 1H, H-4_eq_), 1.25 (d, *J* = 6.2 Hz, 3H, H-11′), 1.10 (d, *J* = 6.1 Hz, 3H, H-9); ^13^C NMR (175 MHz, CDCl_3_) *δ* = 162.2 (d, *J_C-F_* = 244.9 Hz, 1C, C-15′), 156.9 (1C, C-6′), 156.5 (1C, C-8′), 147.9, 147.4 (2C, C-11a, C-14a), 141.1, 139.8 (2C, C-7, C-8), 139.8 (d, *J_C-F_* = 2.8 Hz, 1C, C-12′), 135.7 (1C, C-4a′), 135.2 (1C, C-10), 129.8 (d, *J_C-F_* = 8.1 Hz, 2C, C-13′, C-17′), 127.7 (1C, C-5), 126.4 (1C, C-4a), 122.7 (1C, C-8a), 122.3 (1C, C-16), 120.0 (1C, C-5′), 118.4 (1C, C-8a′), 117.1 (1C, C-6), 114.9 (d, *J_C-F_* = 21.4 Hz, 2C, C-14′, C-16′), 109.3 (1C, C-11), 108.0 (1C, C-15), 101.2 (1C, C-13), 93.9 (1C, C-7′), 77.1 (1C, C-1′), 73.6 (1C, C-1), 70.7 (1C, C-3′), 63.6 (1C, C-3), 55.7 (1C, C-9′), 55.3 (1C, C-10′), 36.5 (1C, C-4′), 33.3 (1C, C-4), 21.8 (2C, C-11′, C-9). IR (KBr): 3308, 2968, 2928, 1710, 1662, 1594, 1507, 1486, 1438, 1321, 1234, 1209, 1040, 936 cm^−1^. HRMS (ESI) calcd. for C_35_H_33_FNaO_8_ [M+Na]^+^ 623.2052, found 623.2049.

##### General Procedures for Epimerization Reaction (Isomerization) of C-1/C-1′ Substituted bis-Isochroman Derivatives

**Method A:** the corresponding C-1/C-1′ substituted *bis*-isochroman derivative (stereopure or mixture of C-1/C-1′ stereoisomers, 1.0 equiv.) was dissolved in 1,4-dioxane then TfOH (4.0 equiv.) was added to the solution. The mixture was stirred for 3 h at 100 °C. After that l-ascorbic acid (0.5 equiv.) was added to the mixture at room temperature and stirred for 2 min. The solvent was evaporated in *vacuo*, the residue was diluted with EtOAc and water, then the phases were separated in a separatory funnel. The aqueous phase was washed three times with EtOAc. The combined organic phases were washed with brine, dried over anhydrous MgSO_4_, filtered, and the solvent was evaporated in *vacuo*. The residue was purified by column chromatography and preparative chiral HPLC to yield the other stereoisomers in different ratios.

**Method B:** the corresponding C-1/C-1′ substituted *bis*-isochroman derivative (stereopure or mixture of C-1/C-1′ stereoisomers, 1.0 equiv.) was dissolved in acetic acid-water (9:1) then TfOH (4.5 equiv.) was added to the solution. The mixture was stirred at 100 °C until it reached the maximum conversion of stereoisomers (ca. 4 h) on the basis of TLC monitoring. After that, EtOAc and water were added to the mixture and the phases were separated in a separatory funnel. The aqueous phase was washed three times with EtOAc, and the combined organic phases were washed with a saturated solution of NaHCO_3_, dried over anhydrous MgSO_4_, filtered, and the solvent was evaporated in *vacuo*. The residue can be purified by column chromatography and preparative chiral HPLC (new stereoisomers detected by TLC were not isolated).

**(a*S*,1*S*,3*S*,1′*R*,3′*S*)-1,1′-*bis*(4-fluorophenyl)-6′,8′-dimethoxy-3,3′-dimethyl-[5,5′-*bis*-isochroman]-7,8-diol [*trans*,*cis*-(a*S*,1*S*,3*S*,1′*R*,3′*S*)-20]**

**Prepared by method A**. Flash column chromatography: hexanes/acetone 4:1. HPLC: Lux i-Cellulose-5 (150 × 21.2 mm), *n*-hexane/(MeOH:2-PrOH 1:1) 80:20, 254 nm, t_R, prep_ = 4.82 min. *trans*,*cis*-(a*S*,1*S*,3*S*,1′*R*,3′*S*)-**20**: 12 mg (yield: 6%) white crystals, mp 233–236 °C. R_f_ = 0.48 (hexanes/acetone 1.5:1). [α${]}_{D}^{20}$ −39 (*c* = 0.17; CHCl_3_). ECD: (*c* = 1.13 × 10^−4^ M; MeCN) λ [nm], (Δε) = 291 (−3.04), 276sh (−0.59), 265 (−2.06), 249 (1.77), 233sh (−7.48), 220 (−19.53), 210 (0.66), 204 (−4.02), 200 (−0.21), 194 (−4.25). ^1^H NMR (700 MHz, acetone-*d*_6_) *δ* = 7.32–7.29 (m, 2H, H-13′, H-17′), 7.29–7.27 (m, 2H, H-11, H-15), 7.09–7.05 (m, 2H, H-12, H-14), 7.03–6.99 (m, 2H, H-14′, H-16′), 6.70 (s, 1H, H-6), 6.56 (s, 1H, H-7′), 6.04 (s, 1H, H-1), 5.80 (s, 1H, H-1′), 3.72 (s, 3H, H-9′), 3.71–3.65 (m, 1H, H-3′), 3.55 (s, 3H, H-10′), 3.56–3.52 (m, 1H, H-3), 2.45 (dd, *J* = 16.3, 10.8 Hz, 1H, H-4′_ax_), 2.25 (dd, *J* = 16.4, 11.2 Hz, 1H, H-4_ax_), 2.23 (ddd, *J* = 16.3, 2.3, 1.2 Hz, 1H, H-4′_eq_), 2.06–2.02 (m, 1H, H-4_eq_), 1.15 (d, *J* = 6.1 Hz, 3H, H-11′), 1.00 (d, *J* = 6.1 Hz, 3H, H-9); ^13^C NMR (175 MHz, acetone-*d_6_*) *δ* = 162.9 (d, *J_C-F_* = 243.2 Hz, 1C, C-13), 162.6 (d, *J_C-F_* = 242.5 Hz, 1C, C-15′), 157.9 (1C, C-6′), 157.2 (1C, C-8′), 142.7, 141.4 (2C, C-7, C-8), 141.9 (d, *J_C-F_* = 2.9 Hz, 1C, C-12′), 139.4 (d, *J_C-F_* = 2.8 Hz, 1C, C-10), 136.4 (1C, C-4a′), 131.4 (d, *J_C-F_* = 8.1 Hz, 2C, C-11, C-15), 130.8 (d, *J_C-F_* = 8.1 Hz, 2C, C-13′, C-17′), 128.1 (1C, C-5), 126.1 (1C, C-4a), 123.4 (1C, C-8a), 121.2 (1C, C-5′), 119.1 (1C, C-8a′), 117.6 (1C, C-6), 115.2 (d, *J_C-F_* = 21.3 Hz, 2C, C-12, C-14), 114.9 (d, *J_C-F_* = 21.4 Hz, 2C, C-14′, C-16′), 94.9 (1C, C-7′), 77.4 (1C, C-1′), 73.8 (1C, C-1), 70.7 (1C, C-3′), 64.0 (1C, C-3), 55.8 (1C, C-9′), 55.5 (1C, C-10′), 37.0 (1C, C-4′), 34.1 (1C, C-4), 22.1 (2C, C-9, C-11′). IR (KBr): 3421, 2970, 2931, 1595, 1508, 1320, 1210, 1119, 833, 792 cm^−1^. HRMS (ESI) data was identical with that of the *cis*,*cis*-(a*S*,1*R*,3*S*,1′*R*,3′*S*)-**20** stereoisomer.

**(a*S*,1*R*,3*S*,1′*S*,3′*S*)-1,1′-*bis*(4-fluorophenyl)-6′,8′-dimethoxy-3,3′-dimethyl-[5,5′-*bis*-isochroman]-7,8-diol [*cis*,*trans*-(a*S*,1*R*,3*S*,1′*S*,3′*S*)-20]**

**Prepared by method A**. Flash column chromatography: hexanes/acetone 4:1. HPLC: Lux i-Cellulose-5 (150 × 21.2 mm), *n*-hexane/(MeOH:2-PrOH 1:1) 80:20, 254 nm, t_R, prep_ = 7.32 min. *cis*,*trans*-(a*S*,1*R*,3*S*,1′*S*,3′*S*)-**20**: 14 mg (yield: 7%) white crystals, mp 122–125 °C. R_f_ = 0.48 (hexanes/acetone 1.5:1). [α${]}_{D}^{20}$ −85 (*c* = 0.28; CHCl_3_). ECD: (*c* = 1.08 × 10^−4^ M; MeCN) λ [nm], (Δε) = 313 (−0.23), 308 (0.50), 285 (−3.07), 269 (−2.16), 239 (−23.40), 228sh (−4.89), 213 (9.13), 207 (6.20), 198 (22.47), 193 (7.36). ^1^H NMR (700 MHz, acetone-*d*_6_) *δ* = 7.38–7.32 (m, 2H, H-11, H-15), 7.23–7.17 (m, 2H, H-13′, H-17′), 7.07–7.04 (m, 2H, H-14′, H-16′), 7.03–7.00 (m, 2H, H-12, H-14), 6.70 (s, 1H, H-7′), 6.59 (s, 1H, H-6), 5.92 (s, 1H, H-1′), 5.91 (s, 1H, H-1), 3.81 (s, 3H, H-9′), 3.73 (s, 3H, H-10′), 3.73–3.68 (m, 1H, H-3), 3.53–3.46 (m, 1H, H-3′), 2.40 (ddd, *J* = 15.9, 10.9, 1.4 Hz, 1H, H-4_ax_), 2.31 (dd, *J* = 17.0, 11.1 Hz, 1H, H-4′_ax_), 2.18 (ddd, *J* = 15.9, 2.2, 1.0 Hz, 1H, H-4_eq_), 2.09 (dd, *J* = 17.0, 3.4 Hz, 1H, H-4′_eq_), 1.13 (d, *J* = 6.1 Hz, 3H, H-9), 1.01 (d, *J* = 6.1 Hz, 3H, H-11′); ^13^C NMR (175 MHz, acetone-*d_6_*) *δ* = 162.8 (d, *J_C-F_* = 243.2 Hz, 1C, C-15′), 162.7 (d, *J_C-F_* = 242.2 Hz, 1C, C-13), 158.5 (1C, C-6′), 156.9 (1C, C-8′), 143.0, 141.6 (2C, C-7, C-8), 141.4 (d, *J_C-F_* = 2.9 Hz, 1C, C-10), 139.8 (d, *J_C-F_* = 2.9 Hz, 1C, C-12′), 135.6 (1C, C-4a′), 131.2 (d, *J_C-F_* = 8.1 Hz, 2C, C-11, C-15), 131.1 (d, *J_C-F_* = 8.1 Hz, 2C, C-13′, C-17′), 127.3 (1C, C-4a), 127.1 (1C, C-5), 125.1 (1C, C-8a), 121.4 (1C, C-5′), 116.7 (1C, C-6), 116.6 (1C, C-8a′), 115.2 (d, *J_C-F_* = 21.3 Hz, 2C, C-14′, C-16′), 114.9 (d, *J_C-F_* = 21.4 Hz, 2C, C-12, C-14), 94.4 (1C, C-7′), 77.4 (1C, C-1), 73.5 (1C, C-1′), 71.0 (1C, C-3), 63.9 (1C, C-3′), 56.2 (1C, C-9′), 55.7 (1C, C-10′), 36.0 (1C, C-4′), 35.5 (1C, C-4), 22.1 (2C, C-9), 22.0 (1C, C-11′). IR (KBr): 3247, 2969, 2930, 2840, 1596, 1508, 1322, 1305, 1208, 1118, 829 cm^−1^. HRMS (ESI) data was identical with that of the *cis*,*cis*-(a*S*,1*R*,3*S*,1′*R*,3′*S*)-**20** stereoisomer.

**(a*S*,1*S*,3*S*,1′*S*,3′*S*)-1,1′-*bis*(4-fluorophenyl)-6′,8′-dimethoxy-3,3′-dimethyl-[5,5′-*bis*-isochroman]-7,8-diol [*trans*,*trans*-(a*S*,1*S*,3*S*,1′*S*,3′*S*)-20]**

**Prepared by method A**. Flash column chromatography: hexanes/acetone 4:1. *trans*,*trans*-(a*S*,1*S*,3*S*,1′*S*,3′*S*)-**20**: 36 mg (yield: 18%) white crystals, mp 117–120 °C. R_f_ = 0.45 (hexanes/acetone 1.5:1). [α${]}_{D}^{20}$ +40 (*c* = 0.19; CHCl_3_). ECD: (*c* = 1.11 × 10^−4^ M; MeCN) λ [nm], (Δε) = 289 (−3.30), (240 (−16.47), 214 (48.57), 203sh (27.34). ^1^H NMR (700 MHz, acetone-*d*_6_) *δ* = 7.29–7.25 (m, 2H, H-11, H-15), 7.22–7.18 (m, 2H, H-13′, H-17′), 7.06–7.03 (m, 2H, H-12, H-14), 7.04–7.01 (m, 2H, H-14′, H-16′), 6.70 (s, 1H, H-7′), 6.63 (s, 1H, H-6), 6.04 (s, 1H, H-1), 5.92 (s, 1H, H-1′), 3.78 (s, 3H, H-9′), 3.73 (s, 3H, H-10′), 3.58–3.53 (m, 1H, H-3), 3.53–3.49 (m, 1H, H-3′), 2.29 (dd, *J* = 17.2, 11.0 Hz, 1H, H-4′_ax_), 2.25 (dd, *J* = 16.5, 11.1 Hz, 1H, H-4_ax_), 2.18 (dd, *J* = 17.2, 3.4 Hz, 1H, H-4′_eq_), 2.15 (dd, *J* = 16.5, 3.7 Hz, 1H, H-4_eq_), 1.01 (d, *J* = 6.1 Hz, 3H, H-11′), 1.01 (d, *J* = 6.1 Hz, 3H, H-9); ^13^C NMR (175 MHz, acetone-*d_6_*) *δ* = 162.9 (d, *J_C-F_* = 243.2 Hz, 1C, C-13), 162.8 (d, *J_C-F_* = 243.2 Hz, 1C, C-15′), 158.2, 156.9 (2C, C-6′, C-8′), 142.7, 141.3 (2C, C-7, C-8), 139.7 (d, *J_C-F_* = 2.9 Hz, 1C, C-12′), 139.3 (d, *J_C-F_* = 2.9 Hz, 1C, C-10), 135.6 (1C, C-4a′), 131.4 (d, *J_C-F_* = 8.1 Hz, 2C, C-11, C-15), 131.1 (d, *J_C-F_* = 8.1 Hz, 2C, C-13′, C-17′), 128.1 (1C, C-5), 125.9 (1C, C-4a), 123.5 (1C, C-8a), 121.3 (1C, C-5′), 117.1 (1C, C-6), 116.7 (1C, C-8a′), 115.2 (d, *J_C-F_* = 21.3 Hz, 2C, C-12, C-14, d, *J_C-F_* = 21.3 Hz, 2C, C-14′, C-16′), 94.3 (1C, C-7′), 73.8 (1C, C-1), 73.5 (1C, C-1′), 64.0 (1C, C-3), 63.9 (1C, C-3′), 55.9 (1C, C-9′), 55.7 (1C, C-10′), 35.8 (1C, C-4′), 34.1 (1C, C-4), 22.1 (2C, C-9, C-11′). IR (KBr): 3421, 2969, 2930, 2839, 1599, 1507, 1320, 1222, 1207, 1120, 837 cm^−1^. HRMS (ESI) data was identical with that of the *cis*,*cis*-(a*S*,1*R*,3*S*,1′*R*,3′*S*)-**20** stereoisomer.

##### General Procedure for Oxidation-Reduction Reaction of *bis*-Isochroman Derivatives Containing Pyrocatechol Unit

**Oxidation:** the corresponding pyrocatechol *bis*-isochroman derivative (1.0 equiv.) was dissolved in MeOH-water (5:1) in an Erlenmeyer flask and NaIO_4_ (1.0 equiv.) was added to the solution. The reaction mixture was shaken up two to three times in a few minutes while the color of the mixture changed fast from colorless to deep dark brown. After the starting material was consumed on the basis of TLC monitoring, the mixture was diluted with EtOAc and water, then the phases were separated in a separatory funnel. The aqueous phase was washed three times with EtOAc. The combined organic layers were washed with a saturated solution of NaHCO_3_ and with brine, dried over anhydrous MgSO_4_. After filtration, the solvent was evaporated in *vacuo* to yield the desired *ortho*-quinone *bis*-isochroman.

**Reduction:** the corresponding *ortho*-quinone *bis*-isochroman derivate (1.0 equiv.) was dissolved in MeOH-water (5:1) in a penicillin bottle. After adding exceed amount of l-ascorbic acid to the deep dark brown solution and shaking up the reaction mixture two to three times, the color of the deep dark brown mixture changed fast to colorless. The mixture in the penicillin bottle was extracted (microextraction) with EtOAc, water and a saturated solution of NaHCO_3_. The upper organic phase contained the pyrocatechol *bis*-isochroman derivative on the basis of TLC monitoring.

**(a*S*,1*R*,3*S*,1′*R*,3′*S*)-1,1′-*bis*(4-fluorophenyl)-6′,8′-dimethoxy-3,3′-dimethyl-[5,5′-*bis*-isochroman]-7,8-dione [*cis*,*cis*-(a*S*,1*R*,3*S*,1′*R*,3′*S*)-26]**

Purification was not required for the crude product. *cis*,*cis*-(a*S*,1*R*,3*S*,1′*R*,3′*S*)-**26**: 48 mg (yield: 96%) dark brown crystals, mp 121–123 °C. R_f_ = 0.47 (hexanes/acetone 2:1). [α${]}_{D}^{20}$ −106 (*c* = 0.25; CHCl_3_). ECD: (*c* = 1.26 × 10^−4^ M; MeCN) λ [nm], (Δε) = 485 (−0.94), 375 (2.88), 282 (0.98), 260 (−3.13), 246sh (−7.73), 222 (−25.87), 212 (−13.35), 203sh (−21.57), 196 (−30.05). Crystals were grown in CH_2_Cl_2_:acetone 1:1 at room temperature. ^1^H NMR (360 MHz, CDCl_3_) *δ* = 7.47–7.33, 7.30–7.20 (2m, 2 × 2H, H-11, H-15, H-13′, H-17′), 7.07–7.00, 7.00–6.92 (2m, 2 × 2H, H-12, H-14, H-14′, H-16′), 6.34, 6.26 (2s, 2 × 1H, H-6, H-7′), 5.81, 5.61 (2s, 2 × 1H, H-1, H-1′), 3.89, 3.54 (2s, 2 × 3H, H-9′, H-10′), 3.85–3.65 (m, 2H, H-3, H-3′), 2.75, 2.37, 2.27, 1.87 (dd, *J* = 16.3, 10.6 Hz, 1H, d, *J* = 16.8 Hz, 1H, ddd, *J* = 19.0, 10.1, 3.9 Hz, 1H, dd, *J* = 19.0, 2.4 Hz, 1H, H-4, H-4′), 1.34, 1.25 (d, *J* = 6.1 Hz, 3H, d, *J* = 6.1 Hz, 3H, H-9, H-11′); ^13^C NMR (90 MHz, CDCl_3_) *δ* = 179.3, 177.8 (2C, C-7, C-8), 162.7, 162.2 (d, *J_C-F_* = 246.4 Hz, 1C, d, *J_C-F_* = 245.1 Hz, 1C, C-13, C-15′), 158.2, 156.1, 151.9, 148.4, 137.1, 135.0, 119.4, 115.6 (8C, C-4a, C-5, C-8a, C-4a′, C-5′, C-6′, C-8′, C-8a′), 139.3, 136.2 (d, *J_C-F_* = 3.0 Hz, 1C, d, *J_C-F_* = 3.0 Hz, 1C, C-10, C-12′), 130.1, 129.7 (d, *J_C-F_* = 8.4 Hz, 2C, d, *J_C-F_* = 8.1 Hz, 2C, C-11, C-15, C-13′, C-17′), 115.4, 114.97 (d, *J_C-F_* = 21.5 Hz, 2C, d, *J_C-F_* = 21.4 Hz, 2C, C-12, C-14, C-14′, C-16′), 129.6, 93.7 (2C, C-6, C-7′), 77.0, 76.1 (2C, C-1, C-1′), 70.1, 69.5 (2C, C-3, C-3′), 55.9, 55.4 (2C, C-9′, C-10′), 36.4, 35.3 (2C, C-4, C-4′), 21.8, 21.2 (2C, C-9, C-11′). IR (KBr): 3434, 2973, 2932, 2844, 1661, 1595, 1509, 1326, 1211, 1117, 1069, 830, 549 cm^−1^. HRMS (ESI) calcd. for C_34_H_30_F_2_NaO_6_ [M+Na]^+^ 595.1903, found 595.1903.

**(a*S*,1*R*,3*S*,1′*R*,3′*S*)-1,1′-*bis*(4-bromophenyl)-6′,8′-dimethoxy-3,3′-dimethyl-[5,5′-*bis*-isochroman]-7,8-dione [*cis*,*cis*-(a*S*,1*R*,3*S*,1′*R*,3′*S*)-27]**

Purification was not required for the crude product. *cis*,*cis*-(a*S*,1*R*,3*S*,1′*R*,3′*S*)-**27**: 49 mg (yield: 98%) dark brown crystals, mp 140–143 °C. R_f_ = 0.53 (hexanes/acetone 2:1). [α${]}_{D}^{20}$ −107 (*c* = 0.21; CHCl_3_). ECD: (*c* = 7.87 × 10^−5^ M; MeCN) λ [nm], (Δε) = 276 (0.93), 228 (−41.78), 213 (6.58), 203 (−19.94). Crystals were grown in MeOH at room temperature. ^1^H NMR (360 MHz, CDCl_3_) *δ* = 7.47, 7.41, 7.32, 7.16 (d, *J* = 8.4 Hz, 2H, d, *J* = 8.3 Hz, 2H, d, *J* = 8.3 Hz, 2H, d, *J* = 8.3 Hz, 2H, H-11, H-12, H-14, H-15, H-13′, H-14′, H-16′, H-17′), 6.34, 6.26 (2s, 2 × 1H, H-6, H-7′), 5.78, 5.58 (2s, 2 × 1H, H-1, H-1′), 3.88, 3.56 (2s, 2 × 3H, H-9′, H10′), 3.84–3.66 (m, 2H, H-3, H-3′), 2.74, 2.37, 2.28, 1.87 (dd, *J* = 16.1, 10.8 Hz, 1H, d, *J* = 16.0 Hz, 1H, ddd, *J* = 18.9, 10.1, 3.8 Hz, 1H, d, *J* = 19.1 Hz, 1H, H-4, H-4′), 1.33, 1.25 (d, *J* = 6.1 Hz, 3H, d, *J* = 6.1 Hz, 3H, H-9, H-11′); ^13^C NMR (90 MHz, CDCl_3_) *δ* = 179.2, 177.7 (2C, C-7, C-8), 158.1, 156.1, 151.7, 148.6, 142.5, 139.3, 136.7, 135.0, 122.3, 121.3, 119.0, 115.6 (12C, C-4a, C-5, C-8a, C-10, C-13, C-4a′, C-5′, C-6′, C-8′, C-8a′, C-12′ C-15′), 131.7, 131.2, 130.1, 129.9 (8C, C-11, C-12, C-14, C-15, C-13′, C-14′, C-16′, C-17′), 129.6, 93.6 (2C, C-6, C-7′), 77.0, 76.1 (2C, C-1, C-1′), 70.1, 69.5 (2C, C-3, C-3′), 55.9, 55.4 (2C, C-9′, C-10′), 36.3, 35.2 (2C, C-4, C-4′), 21.7, 21.2 (2C, C-9, C-11′). IR (KBr): 3445, 2971, 2930, 2842, 1660, 1594, 1487, 1345, 1326, 1209, 1070, 1012, 816 cm^−1^. HRMS (ESI) calcd. for C_34_H_30_Br_2_NaO_6_ [M+Na]^+^ 715.0301, found 715.0298.

### 3.2. Computational Section

Mixed torsional/low-frequency mode conformational searches were carried out by means of the Macromodel 10.8.011 software, using the Merck Molecular Force Field (MMFF) with an implicit solvent model for CHCl_3_ [21]. All quantum chemical calculations were carried out with the Gaussian 09 software package [22,23]. The B3LYP (VCD) and ωB97X [24] (ECD) functionals with the TZVP basis set and PCM solvent model for CHCl_3_ (VCD) and MeCN (ECD) were used to re-optimize the initial MMFF geometries. TDDFT-ECD and -OR calculations were performed at the B3LYP/TZVP, BH&HLYP/TZVP, CAM-B3LYP/TZVP and the PBE0/TZVP levels of theory with the PCM solvent model for MeCN. ECD spectra were generated as sums of Gaussians with 3000 cm^−1^ widths at half-height, using dipole-velocity-computed rotational strength values [25]. VCD calculations were performed at the B3LYP/TZVP PCM/CHCl_3_ level, while the spectra were gained by applying an 8 cm^−1^ half-height width and scaled by a factor of 0.98. Boltzmann distributions were estimated from the B3LYP and ωB97X energies. The MOLEKEL 5.4 software package was used for visualization of the results [26].

### 3.3. Determining Antimicrobial Activity

The efficacy of the prepared compounds was determined using the broth microdilution method in accordance with the recommendation of the European Committee on Antimicrobial Susceptibility Testing (EUCAST). EUCAST recommends testing according to the International Standard ISO 20776-1 (EUCAST reading guide for broth microdilution Version 5.0 January 2024, https://www.eucast.org/ast_of_bacteria/mic_determination, accessed on 14 September, 2024). Minimal inhibitory concentrations (MICs) of the compounds were measured against four Gram-positive and one Gram-negative bacterial strains [*Bacillus subtilis* ATCC 6633, Methicillin-sensitive *Staphylococcus aureus* (MSSA) ATCC 29213, Methicillin-resistant *Staphylococcus aureus* (MRSA) ATCC 33591, *Enterococcus faecalis* ATCC 51299 and *Acinetobacter baumannii* ATCC BAA1605]. Bacterial strains were grown on Mueller–Hinton (MH) agar plates (bioMérieux, Marcy-l’Étoile, France) at 35 °C overnight. Appropriate numbers of colonies were suspended in physiological saline to reach the density of 0.5 McFarland for inoculation. The given preparations of the compounds were two-fold serially diluted from 64 to 0.125 μg/mL in MH broth, and then 100 μL of each dilution was transferred into microplate holes. Inoculation was carried out with 10 μL of each bacterial suspension. Incubation was performed at 35.5 °C for 18 h, and the determination of the MIC was made with the naked eye. For MIC determination, a positive growth control consisting solely of broth and the respective bacterial strain was used in duplicate in all cases. To exclude contamination, a negative control containing the test compound at a concentration of 0.25 mg/L in broth without bacterial inoculation was employed. Table 2 was supplemented with the MIC values of several antibiotics commonly used in clinical practice against the respective bacterial strains to facilitate evaluation of the efficacy of the compounds under investigation.

### 3.4. General Description of the X-Ray Crystallographic Study

X-ray quality crystals could be grown from appropriate solvents by slow evaporation. A chosen crystal was then fixed under a microscope onto a Mitegen loop using high-density oil. Diffraction Intensity data was collected at ambient or low (150 K) temperature on a Bruker-D8 Venture diffractometer (Bruker AXS GmbH, Karlsruhe, Germany) equipped with INCOATEC IµS 3.0 (Incoatec GmbH, Geesthacht, Germany) dual (Cu and Mo) sealed tube micro sources and a Photon II Charge-Integrating Pixel Array detector (Bruker AXS GmbH, Karlsruhe, Germany) using Mo Kα (λ = 0.71073 Å) or Cu Kα (λ = 1.541 Å) radiation.

The low quality of the crystals caused a few A and B level errors but the structures are considered to be correct based on chemical evidences. The Flack parameter was meaningless in a few cases as Mo Kα radiation was used for light atom structures or quality of the crystal. Nevertheless, the stereoselective synthetic scheme enabled to assign the stereogenic elements based on the known (*S*) absolute configuration of C-3 and C-3′.

High-multiplicity data collection and integration were performed using APEX5 (version 2017.3-0, Bruker AXS Inc., 2017, Madison, WI, USA) software. Data reduction and multiscan absorption correction were performed using SAINT (version 8.38A, Bruker AXS Inc., 2017, Madison, WI, USA). The structure was solved using direct methods and refined on F^2^ using the SHELXL program [27] incorporated into the APEX5 suite. Refinement was performed anisotropically for all non-hydrogen atoms. Hydrogen atoms were placed in idealized positions on parent atoms in the final refinement except O-H protons in which could be found at the difference electron density map and the respective O-H distances were constrained. Nevertheless the orientation of the –OH groups is not well-defined also resulting crystallographic errors but this only means that the description of the hydrogen bond network is ambiguous.

The CIF file was manually merged using publCIF software (version 1.9.6) [28], while graphics were designed using the Mercury program [29]. The results for the X-ray diffraction structure determinations followed the Checkcif functionality of PLATON software (version 2023.1) (Utrecht University, Utrecht, The Netherlands) [30].

## 4. Conclusions

We have developed a stereoselective synthetic route for preparing 5,5′-linked axially chiral heterodimeric *bis*-1-arylisochromans, which consists of a diastereoselective Suzuki–Miyaura cross-coupling of two optically active 1-arylpropan-2-ol derivatives and subsequent oxa-Pictet–Spengler cyclizations of the two subunits using aryl aldehydes. The *ortho*-trisubstituted stereogenic biaryl axis was introduced with high diastereoselectivity via a Suzuki–Miyaura cross-coupling. The diastereomers obtained in the oxa-Pictet–Spengler cyclization were separated, and their absolute configurations were determined using a combination of VCD calculations, NMR measurements and six single-crystal X-ray analysis. ECD and VCD calculations of *bis*-isochromans lacking chirality centers at the C-1 and C-1′ positions revealed that ECD transitions do not reflect the axial chirality; however, characteristic VCD transitions could be used to determine it. We produced stereoisomeric target compounds in acid-catalyzed isomerization of the C-1 and C-1′ chirality centers for stereochemistry–activity relationship studies. We oxidized the 7,8-catechol moiety of our *bis*-isochromans to an *ortho*-quinone subunit using sodium metaperiodate to produce axially chiral *ortho*-quinone-isochroman conjugates. The reduction, the reverse reaction, could also be facilitated with L-ascorbic acid. We identified the antibacterial activity of the axially chiral target *bis*-isochromans against *Bacillus subtilis* and *Enterococcus faecalis* with MIC values down to 4.0 and 0.5 μg/mL, respectively.

## Figures and Tables

**Table 1:** Optimization of the Suzuki biaryl coupling reaction of aryl halides (*S*)-**2** and (*S*)-**13** and aryl pinacolatoboronate ester (*S*)-**3**.

Entry	Aryl Halide	Phosphine Ligand	Yield of (a*S*,2*S*,2′*S*)-15
1	(*S*)-**13**	XPhos	trace
2	(*S*)-**2**	PPh_3_	36%
3	(*S*)-**2**	XPhos	47%
4	(*S*)-**2**	Xantphos	63%

**Table 2:** Minimum inhibitory concentration (MIC) values of target compounds against *B. subtilis* ATCC 6633 and *E. faecalis* ATCC 51299 in µg/mL.

		MIC [µg/mL]	
Entry	Compound	*B. subtilis* ATCC 6633	*E. faecalis* ATCC 51299
1	(a*S*,3*S*,3′*S*)-**19**	8	4
2	*cis*,*cis*-(a*S*,1*R*,3*S*,1′*R*,3′*S*)-**20**	>64	>64
3	*trans*,*cis*-(a*S*,1*S*,3*S*,1′*R*,3′*S*)-**20**	16	0.5
4	*cis*,*trans*-(a*S*,1*R*,3*S*,1′*S*,3′*S*)-**20**	4	4
5	*trans*,*trans*-(a*S*,1*S*,3*S*,1′*S*,3′*S*)-**20**	4	0.5
6	*cis*,*cis*-(a*S*,1*R*,3*S*,1′*R*,3′*S*)-**21**	4	1
7	*cis*,*cis*-(a*S*,1*R*,3*S*,1′*R*,3′*S*)-**22**	16	16
8	*cis*,*trans*-(a*S*,1*R*,3*S*,1′*S*,3′*S*)-**22**	4	4
9	*trans*,*cis*-(a*S*,1*S*,3*S*,1′*R*,3′*S*)-**22**	4	32
10	*trans*,*trans*-(a*S*,1*S*,3*S*,1′*S*,3′*S*)-**22**	4	32
11	*trans*,*trans*-(a*S*,1*S*,3*S*,1′*S*,3′*S*)-**23**	4	4
12	*trans*,*trans*-(a*R*,1*S*,3*S*,1′*S*,3′*S*)-**23**	16	1
13	*trans*,*cis*-(a*S*,1*S*,3*S*,1′*R*,3′*S*)-**23**	32	32
14	*cis*-(a*S*,2*S*,1′*R*,3′*S*)-**24**	16	16
15	*trans*,*cis*-(a*S*,1*S*,3*S*,1′*R*,3′*S*)-**25**	8	8
16	*cis*,*cis*-(a*S*,1*R*,3*S*,1′*R*,3′*S*)-**26**	8	8
17	*cis*,*cis*-(a*S*,1*R*,3*S*,1′*R*,3′*S*)-**27**	8	8
18	Teicoplanin *	0.64	4
19	Vancomycin *	0.64	128
20	Ciprofloxacin *	0.05	2
21	Colistin *	>64	>64

* Positive controls: teicoplanin, vancomycin are glycopeptide, ciprofloxacin is a fluoroquinolone, colistin (polymyxin E) is a cyclic lipopeptide antibiotic.

## Data Availability

The original contributions presented in the study are included in the article/Supplementary Materials, further inquiries can be directed to the corresponding author/s. The supplementary crystallographic data for each compound can be obtained free of charge from the Cambridge Crystallographic Data Centre via http://www.ccdc.cam.ac.uk/data_request/cif using reference deposition numbers: 2467523 for *cis*,*cis*-(a*S*,1*R*,3*S*,1′*R*,3′*S*)-**20**, 2467524 for *cis*,*cis*-(a*S*,1*R*,3*S*,1′*R*,3′*S*)-**21**, 2467525 for *cis*,*trans*-(a*S*,1*R*,3*S*,1′*S*,3′*S*)-**22**, 2467526 for *cis*-(a*S*,2*S*,1′*R*,3′*S*)-**24**, 2467527 for *cis*,*cis*-(a*S*,1*R*,3*S*,1′*R*,3′*S*)-**26** and 2467528 for *cis*,*cis*-(a*S*,1*R*,3*S*,1′*R*,3′*S*)-**27**.

## Supplementary Materials

The following supporting information can be downloaded at: https://www.mdpi.com/article/10.3390/ijms26167777/s1, synthetic procedures, spectroscopic characterization and X-ray diffraction analysis of compounds, details of ECD and VCD calculations.
