# Supplementary material for: Stereoselective Synthesis of Axially Chiral 5,5′-Linked bis-1-Arylisochromans with Antibacterial Activity
Source: Int J Mol Sci. 2025 Aug 12;26(16):7777. doi: 10.3390/ijms26167777 (PMC12386651; doi:10.3390/ijms26167777)
Supplement: Supplementary file 1 [file ijms-26-07777-s001.zip › ijms-3772420-supplementary.pdf]

Supporting information

Stereoselective synthesis of axially chiral 5,5'-linked *bis*-1-arylisochromans with antibacterial activity

Zoltán Czenke,[1,2] Attila Mándi,[1] Gergely Miklós Fedics,[1,2] Roland Albert Barta,[1,2] Attila Kiss-Szikszai,[1] Anna Kurucz-Szabados,[1,2] István Timári,[1] Attila Bényei,[3] Sándor Balázs Király,[1] Eszter Ostorházi,[4] Changsheng Zhang,[5] Máté Kicsák[1,*] and Tibor Kurtán[1,*]

1 Department of Organic Chemistry, University of Debrecen, P. O. Box 400, 4002 Debrecen, Hungary; czenke.zoltan@science.unideb.hu (Z.Cz.), mandi.attila@science.unideb.hu (A.M.), gergofs@gmail.com (G.M.F.), barta.roland@science.unideb.hu (R.A.B.), kiss.attila@science.unideb.hu (A.K-Sz.), szabados.anna@science.unideb.hu (A.K-Sz.), timari.istvan@science.unideb.hu (I.T.), kiraly.sandor.balazs@science.unideb.hu (S.B.K.)

2 Doctoral School of Chemistry, University of Debrecen, Egyetem tér 1., 4032 Debrecen, Hungary

3 Department of Physical Chemistry, University of Debrecen, Egyetem tér 1., 4032 Debrecen, Hungary; benyei.attila@science.unideb.hu (A.B.)

4 Department of Medical Microbiology, Semmelweis University, 1085 Budapest, Hungary; droeszter@gmail.com (E.O.)

5 Key Laboratory of Tropical Marine Bioresources and Ecology, Guangdong Key Laboratory of Marine Materia Medica, Institutions of South China Sea Ecology and Environmental Engineering, South China Sea Institute of Oceanology, Chinese Academy of Sciences, Guangzhou 510301, China, czhang@scsio.ac.cn (C.Z.)

* Correspondence: kicsak.mate@science.unideb.hu (M.K.), kurtan.tibor@science.unideb.hu (T.K.)

1. Table of Contents

1. **Table of Contents** 2
   1.1. **Table of Figures** 3
   1.2. **List of Tables** 10
2. **General Information** 11
   2.1. **Computational section** 12
3. **Spectra of the compounds** 13
   3.1. **NMR spectra** 13
   3.2. **ECD and VCD spectra** 82
   3.3. **IR spectra** 84
   3.4. **MS spectra** 102
4. **Calculations** 110
5. **X-ray Crystallographic Study** 123
   5.1. **General description** 123
   5.2. **Molecular Structure Analysis** 126
6. **References** 127

1.1. Table of Figures

Figure S1. ^{1}H NMR (500 MHz) spectrum of (*S*)-**8** in $CDCl_3$ 13

Figure S2. ^{13}C NMR (125 MHz) spectrum of (*S*)-**8** in $CDCl_3$ 13

Figure S3. ^{1}H NMR (400 MHz) spectrum of (*S*)-**6** in $CDCl_3$ 14

Figure S4. ^{13}C NMR (100 MHz) spectrum of (*S*)-**6** in $CDCl_3$ 14

Figure S5. ^{1}H NMR (500 MHz) spectrum of (*S*)-**12** in $CDCl_3$ 15

Figure S6. ^{13}C NMR (125 MHz) spectrum of (*S*)-**12** in $CDCl_3$ 15

Figure S7. ^{1}H NMR (400 MHz) spectrum of (*S*)-**7** in $CDCl_3$ 16

Figure S8. ^{13}C NMR (100 MHz) spectrum of (*S*)-**7** in $CDCl_3$ 16

Figure S9. ^{1}H NMR (500 MHz) spectrum of (*S*)-**2** in $CDCl_3$ 17

Figure S10. ^{13}C NMR (125 MHz) spectrum of (*S*)-**2** in $CDCl_3$ 17

Figure S11. ^{1}H NMR (500 MHz) spectrum of (*S*)-**13** in $CDCl_3$ 18

Figure S12. ^{13}C NMR (125 MHz) spectrum of (*S*)-**13** in $CDCl_3$ 18

Figure S13. ^{1}H NMR (400 MHz) spectrum of (*S*)-**9** in $CDCl_3$ 19

Figure S14. ^{13}C NMR (100 MHz) spectrum of (*S*)-**9** in $CDCl_3$ 19

Figure S15. ^{1}H NMR (360 MHz) spectrum of (*S*)-**8a** and (*S*)-**8b** (regioisomers ratio 1:1) in $CDCl_3$... 20

Figure S16. ^{13}C NMR (90 MHz) spectrum of (*S*)-**8a** and (*S*)-**8b** (regioisomers ratio 1:1) in $CDCl_3$.... 20

Figure S17. ^{1}H NMR (360 MHz) spectrum of (*S*)-**8c** in $CDCl_3$. 21

Figure S18. ^{13}C NMR (90 MHz) spectrum of (*S*)-**8c** in $CDCl_3$. 21

Figure S19. ^{1}H NMR (400 MHz) spectrum of in (*S*)-**14** $CDCl_3$ 22

Figure S20. ^{13}C NMR (100 MHz) spectrum of in (*S*)-**14** $CDCl_3$ 22

Figure S21. ^{1}H NMR (360 MHz) spectrum of (*S*)-**3** in $CDCl_3$ 23

Figure S22. ^{13}C NMR (90 MHz) spectrum of (*S*)-**3** in $CDCl_3$ 23

Figure S23. ^{1}H NMR (400 MHz) spectrum of (a*S*,2*S*,2'*S*)-**15** in $CDCl_3$ 24

Figure S24. ^{13}C NMR (100 MHz) spectrum of (a*S*,2*S*,2'*S*)-**15** in $CDCl_3$ 24

Figure S25. ^{1}H NMR (360 MHz) spectrum of in (a*S*,2*S*,2'*S*)-**16** $CDCl_3$ 25

Figure S26. ^{13}C NMR (90 MHz) spectrum of (a*S*,2*S*,2'*S*)-**16** in $CDCl_3$ 25

Figure S27. ^{1}H NMR (400 MHz) spectrum of (a*S*,2*S*,2'*S*)-**17** in $CDCl_3$ 26

Figure S28. ^{13}C NMR (100 MHz) spectrum of (a*S*,2*S*,2'*S*)-**17** in $CDCl_3$ 26

Figure S29. ^{1}H NMR (400 MHz) spectrum of (a*S*,3*S*,3'*S*)-**19** in $CDCl_3$ 27

Figure S30. ^{13}C NMR (100 MHz) spectrum of (a*S*,3*S*,3'*S*)-**19** in $CDCl_3$ 27

Figure S31. ^{1}H NMR (400 MHz) spectrum of (a*S*,3*S*,3'*S*)-**18** in $CDCl_3$ 28

Figure S32. ^{13}C NMR (100 MHz) spectrum of (a*S*,3*S*,3'*S*)-**18** in $CDCl_3$ 28

Figure S33. ^{1}H NMR (700 MHz) spectrum of *cis*,*cis*-(a*S*,1*R*,3*S*,1'*R*,3'*S*)-**21** in acetonitrile-d_3. 29

Figure S34. ^{13}C NMR (175 MHz) spectrum of *cis*,*cis*-(a*S*,1*R*,3*S*,1'*R*,3'*S*)-**21** in acetonitrile-d_3. 29

Figure S35. ^{1}H-^{1}H COSY NMR (700 MHz) spectrum of *cis*,*cis*-(a*S*,1*R*,3*S*,1'*R*,3'*S*)-**21** in acetonitrile-d_3. 30

Figure S36. ^{1}H-^{13}C HSQC NMR (700 MHz) spectrum of *cis*,*cis*-(a*S*,1*R*,3*S*,1’*R*,3’*S*)-**21** in acetonitrile-*d*3 30
Figure S37. ^{1}H-^{13}C HMBC NMR (700 MHz) spectrum of *cis*,*cis*-(a*S*,1*R*,3*S*,1’*R*,3’*S*)-**21** in acetonitrile-*d*3 31
Figure S38. ^{1}H-^{1}H ROESY NMR (700 MHz) spectrum of *cis*,*cis*-(a*S*,1*R*,3*S*,1’*R*,3’*S*)-**21** in acetonitrile-*d*3 31
Figure S39. ^{1}H NMR (700 MHz) spectrum of *cis*,*trans*-(a*S*,1*R*,3*S*,1’*S*,3’*S*)-**21** in acetonitrile-*d*3. 32
Figure S40. ^{13}C NMR (175 MHz) spectrum of *cis*,*trans*-(a*S*,1*R*,3*S*,1’*S*,3’*S*)-**21** in acetonitrile-*d*3. 32
Figure S41. ^{1}H-^{1}H COSY NMR (700 MHz) spectrum of *cis*,*trans*-(a*S*,1*R*,3*S*,1’*S*,3’*S*)-**21** in acetonitrile-*d*3 33
Figure S42. ^{1}H-^{13}C HSQC NMR (700 MHz) spectrum of *cis*,*trans*-(a*S*,1*R*,3*S*,1’*S*,3’*S*)-**21** in acetonitrile-*d*3 33
Figure S43. ^{1}H-^{13}C HMBC NMR (700 MHz) spectrum of *cis*,*trans*-(a*S*,1*R*,3*S*,1’*S*,3’*S*)-**21** in acetonitrile-*d*3 34
Figure S44. ^{1}H-^{1}H ROESY NMR (700 MHz) spectrum of *cis*,*trans*-(a*S*,1*R*,3*S*,1’*S*,3’*S*)-**21** in acetonitrile-*d*3. 34
Figure S45. ^{1}H NMR (700 MHz) spectrum of *trans*,*cis*-(a*S*,1*S*,3*S*,1’*R*,3’*S*)-**21** in acetonitrile-*d*3. 35
Figure S46. ^{13}C NMR (175 MHz) spectrum of *trans*,*cis*-(a*S*,1*S*,3*S*,1’*R*,3’*S*)-**21** in acetonitrile-*d*3. 35
Figure S47. ^{1}H-^{1}H COSY NMR (700 MHz) spectrum of *trans*,*cis*-(a*S*,1*S*,3*S*,1’*R*,3’*S*)-**21** in acetonitrile-*d*3 36
Figure S48. ^{1}H-^{13}C HSQC NMR (700 MHz) spectrum of *trans*,*cis*-(a*S*,1*S*,3*S*,1’*R*,3’*S*)-**21** in acetonitrile-*d*3 36
Figure S49. ^{1}H-^{13}C HMBC NMR (700 MHz) spectrum of *trans*,*cis*-(a*S*,1*S*,3*S*,1’*R*,3’*S*)-**21** in acetonitrile-*d*3 37
Figure S50. ^{1}H-^{1}H ROESY NMR (700 MHz) spectrum of *trans*,*cis*-(a*S*,1*S*,3*S*,1’*R*,3’*S*)-**21** in acetonitrile-*d*3. 37
Figure S51. ^{1}H NMR (700 MHz) spectrum of *cis*,*cis*-(a*R*,1*R*,3*S*,1’*R*,3’*S*)-**21** in acetonitrile-*d*3. 38
Figure S52. ^{13}C NMR (175 MHz) spectrum of *cis*,*cis*-(a*R*,1*R*,3*S*,1’*R*,3’*S*)-**21** in acetonitrile-*d*3. 38
Figure S53. ^{1}H-^{1}H COSY NMR (700 MHz) spectrum of *cis*,*cis*-(a*R*,1*R*,3*S*,1’*R*,3’*S*)-**21** in acetonitrile-*d*3 39
Figure S54. ^{1}H-^{13}C HSQC NMR (700 MHz) spectrum of *cis*,*cis*-(a*R*,1*R*,3*S*,1’*R*,3’*S*)-**21** in acetonitrile-*d*3 39
Figure S55. ^{1}H-^{13}C HMBC NMR (700 MHz) spectrum of *cis*,*cis*-(a*R*,1*R*,3*S*,1’*R*,3’*S*)-**21** in acetonitrile-*d*3 40
Figure S56. ^{1}H-^{1}H ROESY NMR (700 MHz) spectrum of *cis*,*cis*-(a*R*,1*R*,3*S*,1’*R*,3’*S*)-**21** in acetonitrile-*d*3 40
Figure S57. ^{1}H NMR (400 MHz) spectrum of *cis*,*cis*-(a*S*,1*R*,3*S*,1’*R*,3’*S*)-**20** in $CDCl_3$. 41
Figure S58. ^{13}C NMR (100 MHz) spectrum of *cis*,*cis*-(a*S*,1*R*,3*S*,1’*R*,3’*S*)-**20** in $CDCl_3$. 41

Figure S59. ^{1}H-^{1}H COSY NMR (400 MHz) spectrum of *cis*,*cis*-(a*S*,1*R*,3*S*,1'*R*,3'*S*)-**20** in $CDCl_3$...... 42
Figure S60. ^{1}H-^{13}C HSQC NMR (400 MHz) spectrum of *cis*,*cis*-(a*S*,1*R*,3*S*,1'*R*,3'*S*)-**20** in $CDCl_3$..... 42
Figure S61. ^{1}H-^{13}C HMBC NMR (400 MHz) spectrum of *cis*,*cis*-(a*S*,1*R*,3*S*,1'*R*,3'*S*)-**20** in $CDCl_3$.... 43
Figure S62. ^{1}H-^{1}H ROESY NMR (400 MHz) spectrum of *cis*,*cis*-(a*S*,1*R*,3*S*,1'*R*,3'*S*)-**20** in $CDCl_3$.... 43
Figure S63. ^{1}H NMR (700 MHz) spectrum of *trans*,*cis*-(a*S*,1*S*,3*S*,1'*R*,3'*S*)-**20** in acetone-d_6............. 44
Figure S64. ^{13}C NMR (175 MHz) spectrum of *trans*,*cis*-(a*S*,1*S*,3*S*,1'*R*,3'*S*)-**20** in acetone-d_6............ 44
Figure S65. ^{1}H-^{1}H COSY NMR (700 MHz) spectrum of *trans*,*cis*-(a*S*,1*S*,3*S*,1'*R*,3'*S*)-**20** in acetone-d_6. 45
Figure S66. ^{1}H-^{13}C HSQC NMR (700 MHz) spectrum of *trans*,*cis*-(a*S*,1*S*,3*S*,1'*R*,3'*S*)-**20** in acetone-d_6. 45
Figure S67. ^{1}H-^{13}C HMBC NMR (700 MHz) spectrum of *trans*,*cis*-(a*S*,1*S*,3*S*,1'*R*,3'*S*)-**20** in acetone-d_6. 46
Figure S68. ^{1}H-^{1}H ROESY NMR (700 MHz) spectrum of *trans*,*cis*-(a*S*,1*S*,3*S*,1'*R*,3'*S*)-**20** in acetone-d_6. 46
Figure S69. ^{1}H NMR (700 MHz) spectrum of *cis*,*trans*-(a*S*,1*R*,3*S*,1'*S*,3'*S*)-**20** in acetone-d_6............. 47
Figure S70. ^{13}C NMR (175 MHz) spectrum of *cis*,*trans*-(a*S*,1*R*,3*S*,1'*S*,3'*S*)-**20** in acetone-d_6............ 47
Figure S71. ^{1}H-^{1}H COSY NMR (700 MHz) spectrum of *cis*,*trans*-(a*S*,1*R*,3*S*,1'*S*,3'*S*)-**20** in acetone-d_6. 48
Figure S72. ^{1}H-^{13}C HSQC NMR (700 MHz) spectrum of *cis*,*trans*-(a*S*,1*R*,3*S*,1'*S*,3'*S*)-**20** in acetone-d_6. 48
Figure S73. ^{1}H-^{13}C HMBC NMR (700 MHz) spectrum of *cis*,*trans*-(a*S*,1*R*,3*S*,1'*S*,3'*S*)-**20** in acetone-d_6. 49
Figure S74. ^{1}H-^{1}H ROESY NMR (700 MHz) spectrum of *cis*,*trans*-(a*S*,1*R*,3*S*,1'*S*,3'*S*)-**20** in acetone-d_6. 49
Figure S75. ^{1}H NMR (700 MHz) spectrum of *trans*,*trans*-(a*S*,1*S*,3*S*,1'*S*,3'*S*)-**20** in acetone-d_6. 50
Figure S76. ^{13}C NMR (175 MHz) spectrum of *trans*,*trans*-(a*S*,1*S*,3*S*,1'*S*,3'*S*)-**20** in acetone-d_6. 50
Figure S77. ^{1}H-^{1}H COSY NMR (700 MHz) spectrum of *trans*,*trans*-(a*S*,1*S*,3*S*,1'*S*,3'*S*)-**20** in acetone-d_6.. 51
Figure S78. ^{1}H-^{13}C HSQC NMR (700 MHz) spectrum of *trans*,*trans*-(a*S*,1*S*,3*S*,1'*S*,3'*S*)-**20** in acetone-d_6.. 51
Figure S79. ^{1}H-^{13}C HMBC NMR (700 MHz) spectrum of *trans*,*trans*-(a*S*,1*S*,3*S*,1'*S*,3'*S*)-**20** in acetone-d_6.. 52
Figure S80. ^{1}H-^{1}H ROESY NMR (700 MHz) spectrum of *trans*,*trans*-(a*S*,1*S*,3*S*,1'*S*,3'*S*)-**20** in acetone-d_6.. 52
Figure S81. ^{1}H NMR (700 MHz) spectrum of *cis*,*cis*-(a*S*,1*R*,3*S*,1'*R*,3'*S*)-**22** in $CDCl_3$. 53
Figure S82. ^{13}C NMR (175 MHz) spectrum of *cis*,*cis*-(a*S*,1*R*,3*S*,1'*R*,3'*S*)-**22** in $CDCl_3$. 53
Figure S83. ^{1}H-^{1}H COSY NMR (700 MHz) spectrum of *cis*,*cis*-(a*S*,1*R*,3*S*,1'*R*,3'*S*)-**22** in $CDCl_3$...... 54
Figure S84. ^{1}H-^{13}C HSQC NMR (700 MHz) spectrum of (*cis*,*cis*-(a*S*,1*R*,3*S*,1'*R*,3'*S*)-**22** in $CDCl_3$.... 54

Figure S85. ^{1}H-^{13}C HMBC NMR (700 MHz) spectrum of *cis*,*cis*-(a*S*,1*R*,3*S*,1'*R*,3'*S*)-**22** in $CDCl_3$.... 55
Figure S86. ^{1}H-^{1}H ROESY NMR (700 MHz) spectrum of *cis*,*cis*-(a*S*,1*R*,3*S*,1'*R*,3'*S*)-**22** in $CDCl_3$.... 55
Figure S87. ^{1}H NMR (700 MHz) spectrum of *cis*,*trans*-(a*S*,1*R*,3*S*,1'*S*,3'*S*)-**22** in $CDCl_3$. 56
Figure S88. ^{13}C NMR (175 MHz) spectrum of *cis*,*trans*-(a*S*,1*R*,3*S*,1'*S*,3'*S*)-**22** in $CDCl_3$. 56
Figure S89. ^{1}H-^{1}H COSY NMR (700 MHz) spectrum of *cis*,*trans*-(a*S*,1*R*,3*S*,1'*S*,3'*S*)-**22** in $CDCl_3$... 57
Figure S90. ^{1}H-^{13}C HSQC NMR (700 MHz) spectrum of *cis*,*trans*-(a*S*,1*R*,3*S*,1'*S*,3'*S*)-**22** in $CDCl_3$.. 57
Figure S91. ^{1}H-^{13}C HMBC NMR (700 MHz) spectrum of *cis*,*trans*-(a*S*,1*R*,3*S*,1'*S*,3'*S*)-**22** in $CDCl_3$. 58
Figure S92. ^{1}H-^{1}H ROESY NMR (700 MHz) spectrum of *cis*,*trans*-(a*S*,1*R*,3*S*,1'*S*,3'*S*)-**22** in $CDCl_3$. 58
Figure S93. ^{1}H NMR (700 MHz) spectrum of *trans*,*cis*-(a*S*,1*S*,3*S*,1'*R*,3'*S*)-**22** in $CDCl_3$. 59
Figure S94. ^{13}C NMR (175 MHz) spectrum of *trans*,*cis*-(a*S*,1*S*,3*S*,1'*R*,3'*S*)-**22** in $CDCl_3$. 59
Figure S95. ^{1}H-^{1}H COSY NMR (700 MHz) spectrum of *trans*,*cis*-(a*S*,1*S*,3*S*,1'*R*,3'*S*)-**22** in $CDCl_3$... 60
Figure S96. ^{1}H-^{13}C HSQC NMR (700 MHz) spectrum of *trans*,*cis*-(a*S*,1*S*,3*S*,1'*R*,3'*S*)-**22** in $CDCl_3$.. 60
Figure S97. ^{1}H-^{13}C HMBC NMR (700 MHz) spectrum of *trans*,*cis*-(a*S*,1*S*,3*S*,1'*R*,3'*S*)-**22** in $CDCl_3$. 61
Figure S98. ^{1}H-^{1}H ROESY NMR (700 MHz) spectrum of *trans*,*cis*-(a*S*,1*S*,3*S*,1'*R*,3'*S*)-**22** in $CDCl_3$. 61
Figure S99. ^{1}H NMR (700 MHz) spectrum of *trans*,*trans*-(a*S*,1*S*,3*S*,1'*S*,3'*S*)-**22** in $CDCl_3$................ 62
Figure S100. ^{13}C NMR (175 MHz) spectrum of *trans*,*trans*-(a*S*,1*S*,3*S*,1'*S*,3'*S*)-**22** in $CDCl_3$............. 62
Figure S101. ^{1}H-^{1}H COSY NMR (700 MHz) spectrum of *trans*,*trans*-(a*S*,1*S*,3*S*,1'*S*,3'*S*)-**22** in $CDCl_3$. 63
Figure S102. ^{1}H-^{13}C HSQC NMR (700 MHz) spectrum of *trans*,*trans*-(a*S*,1*S*,3*S*,1'*S*,3'*S*)-**22** in $CDCl_3$. 63
Figure S103. ^{1}H-^{13}C HMBC NMR (700 MHz) spectrum of *trans*,*trans*-(a*S*,1*S*,3*S*,1'*S*,3'*S*)-**22** in $CDCl_3$. 64
Figure S104. ^{1}H-^{1}H ROESY NMR (700 MHz) spectrum of *trans*,*trans*-(a*S*,1*S*,3*S*,1'*S*,3'*S*)-**22** in $CDCl_3$. 64
Figure S105. ^{1}H NMR (700 MHz) spectrum of *trans*,*trans*-(a*R*,1*S*,3*S*,1'*S*,3'*S*)-**23** in $CDCl_3$. 65
Figure S106. ^{13}C NMR (175 MHz) spectrum of *trans*,*trans*-(a*R*,1*S*,3*S*,1'*S*,3'*S*)-**23** in $CDCl_3$. 65
Figure S107. ^{1}H-^{1}H COSY NMR (700 MHz) spectrum of *trans*,*trans*-(a*R*,1*S*,3*S*,1'*S*,3'*S*)-**23** in $CDCl_3$. 66
Figure S108. ^{1}H-^{13}C HSQC NMR (700 MHz) spectrum of *trans*,*trans*-(a*R*,1*S*,3*S*,1'*S*,3'*S*)-**23** in $CDCl_3$. 66
Figure S109. ^{1}H-^{13}C HMBC NMR (700 MHz) spectrum of *trans*,*trans*-(a*R*,1*S*,3*S*,1'*S*,3'*S*)-**23** in $CDCl_3$. 67
Figure S110. ^{1}H-^{1}H ROESY NMR (700 MHz) spectrum of *trans*,*trans*-(a*R*,1*S*,3*S*,1'*S*,3'*S*)-**23** in $CDCl_3$. 67
Figure S111. ^{1}H NMR (700 MHz) spectrum of *trans*,*cis*-(a*S*,1*S*,3*S*,1'*R*,3'*S*)-**23** in $CDCl_3$. 68
Figure S112. ^{13}C NMR (175 MHz) spectrum of *trans*,*cis*-(a*S*,1*S*,3*S*,1'*R*,3'*S*)-**23** in $CDCl_3$. 68
Figure S113. ^{1}H-^{1}H COSY NMR (700 MHz) spectrum of *trans*,*cis*-(a*S*,1*S*,3*S*,1'*R*,3'*S*)-**23** in $CDCl_3$. 69
Figure S114. ^{1}H-^{13}C HSQC NMR (700 MHz) spectrum of *trans*,*cis*-(a*S*,1*S*,3*S*,1'*R*,3'*S*)-**23** in $CDCl_3$.69

Figure S115. ^{1}H-^{13}C HMBC NMR (700 MHz) spectrum of *trans*,*cis*-(a*S*,1*S*,3*S*,1'*R*,3'*S*)-**23** in $CDCl_3$. 70
Figure S116. ^{1}H-^{1}H ROESY NMR (700 MHz) spectrum of *trans*,*cis*-(a*S*,1*S*,3*S*,1'*R*,3'*S*)-**23** in $CDCl_3$. 70
Figure S117. ^{1}H NMR (700 MHz) spectrum of *trans*,*trans*-(a*S*,1*S*,3*S*,1'*S*,3'*S*)-**23** in $CDCl_3$.............. 71
Figure S118. ^{13}C NMR (175 MHz) spectrum of *trans*,*trans*-(a*S*,1*S*,3*S*,1'*S*,3'*S*)-**23** in $CDCl_3$............. 71
Figure S119. ^{1}H-^{1}H COSY NMR (700 MHz) spectrum of *trans*,*trans*-(a*S*,1*S*,3*S*,1'*S*,3'*S*)-**23** in $CDCl_3$. 72
Figure S120. ^{1}H-^{13}C HSQC NMR (700 MHz) spectrum of *trans*,*trans*-(a*S*,1*S*,3*S*,1'*S*,3'*S*)-**23** in $CDCl_3$. 72
Figure S121. ^{1}H-^{13}C HMBC NMR (700 MHz) spectrum of *trans*,*trans*-(a*S*,1*S*,3*S*,1'*S*,3'*S*)-**23** in $CDCl_3$. 73
Figure S122. ^{1}H-^{1}H ROESY NMR (700 MHz) spectrum of *trans*,*trans*-(a*S*,1*S*,3*S*,1'*S*,3'*S*)-**23** in $CDCl_3$. 73
Figure S123. ^{1}H NMR (400 MHz) spectrum of *cis*-(a*S*,2*S*,1'*R*,3'*S*)-**24** in acetone-*d*$_6$......................... 74
Figure S124. ^{13}C NMR (100 MHz) spectrum of *cis*-(a*S*,2*S*,1'*R*,3'*S*)-**24** in acetone-*d*$_6$........................ 74
Figure S125. ^{1}H-^{1}H COSY NMR (400 MHz) spectrum of of *cis*-(a*S*,2*S*,1'*R*,3'*S*)-**24** in acetone-*d*$_6$. ... 75
Figure S126. ^{1}H-^{13}C HSQC NMR (400 MHz) spectrum of of *cis*-(a*S*,2*S*,1'*R*,3'*S*)-**24** in acetone-*d*$_6$. .. 75
Figure S127. ^{1}H-^{13}C HMBC NMR (400 MHz) spectrum of of *cis*-(a*S*,2*S*,1'*R*,3'*S*)-**24** in acetone-*d*$_6$. . 76
Figure S128. ^{1}H-^{1}H NOESY NMR (400 MHz) spectrum of of *cis*-(a*S*,2*S*,1'*R*,3'*S*)-**24** in acetone-*d*$_6$.. 76
Figure S129. ^{1}H NMR (700 MHz) spectrum of *trans*,*cis*-(a*S*,1*S*,3*S*,1'*R*,3'*S*)-**25** in $CDCl_3$................. 77
Figure S130. ^{13}C NMR (175 MHz) spectrum of *trans*,*cis*-(a*S*,1*S*,3*S*,1'*R*,3'*S*)-**25** in $CDCl_3$................ 77
Figure S131. ^{1}H-^{1}H COSY NMR (700 MHz) spectrum of *trans*,*cis*-(a*S*,1*S*,3*S*,1'*R*,3'*S*)-**25** in $CDCl_3$. 78
Figure S132. ^{1}H-^{13}C HSQC NMR (700 MHz) spectrum of *trans*,*cis*-(a*S*,1*S*,3*S*,1'*R*,3'*S*)-**25** in $CDCl_3$.78
Figure S133. ^{1}H-^{13}C HMBC NMR (700 MHz) spectrum of *trans*,*cis*-(a*S*,1*S*,3*S*,1'*R*,3'*S*)-**25** in $CDCl_3$. 79
Figure S134. ^{1}H-^{1}H ROESY NMR (700 MHz) spectrum of *trans*,*cis*-(a*S*,1*S*,3*S*,1'*R*,3'*S*)-**25** in $CDCl_3$. 79
Figure S135. ^{1}H NMR (360 MHz) spectrum of *cis*,*cis*-(a*S*,1*R*,3*S*,1'*R*,3'*S*)-**26** in $CDCl_3$.................... 80
Figure S136. ^{13}C NMR (90 MHz) spectrum of *cis*,*cis*-(a*S*,1*R*,3*S*,1'*R*,3'*S*)-**26** in $CDCl_3$..................... 80
Figure S137. ^{1}H NMR (360 MHz) spectrum of *cis*,*cis*-(a*S*,1*R*,3*S*,1'*R*,3'*S*)-**27** in $CDCl_3$.................... 81
Figure S138. ^{13}C NMR (90 MHz) spectrum of in *cis*,*cis*-(a*S*,1*R*,3*S*,1'*R*,3'*S*)-**27** $CDCl_3$..................... 81
Figure S139. Comparison of the experimental ECD spectrum of (a*S*,3*S*,3'*S*)-**19** (black) with the corresponding computed Neg CAM-B3LYP/TZVP PCM/MeCN ECD spectrum (red). 82
Figure S140. Comparison of the experimental ECD spectrum of (a*S*,3*S*,3'*S*)-**19** (black) with the corresponding computed Pos CAM-B3LYP/TZVP PCM/MeCN ECD spectrum (red). 82
Figure S141. Comparison of the experimental VCD spectrum of (a*S*,3*S*,3'*S*)-**19** (black) with the corresponding computed Neg B3LYP/TZVP PCM/$CHCl_3$ VCD spectrum (red)................................ 83

Figure S142. Comparison of the experimental VCD spectrum of (a*S*,3*S*,3'*S*)-**19** (black) with the corresponding computed Pos B3LYP/TZVP PCM/$CHCl_3$ VCD spectrum (red). 83
Figure S143. Experimental IR spectrum of (*S*)-**11** recorded as KBr disc. 84
Figure S144. Experimental IR spectrum of (*S*)-**6** recorded as KBr disc. 84
Figure S145. Experimental IR spectrum of (*S*)-**12** recorded as KBr disc. 85
Figure S146. Experimental IR spectrum of (*S*)-**7** recorded as KBr disc. 85
Figure S147. Experimental IR spectrum of (*S*)-**2** recorded as KBr disc. 86
Figure S148. Experimental IR spectrum of (*S*)-**13** recorded as KBr disc. 86
Figure S149. Experimental IR spectrum of (*S*)-**9** recorded as KBr disc. 87
Figure S150. Experimental IR spectrum of (*S*)-**8a** and (*S*)-**8b** recorded as KBr disc. 87
Figure S151. Experimental IR spectrum of (*S*)-**8c** recorded as KBr disc... 88
Figure S152. Experimental IR spectrum of (*S*)-**14** recorded as KBr disc. 88
Figure S153. Experimental IR spectrum of (*S*)-**3** recorded as KBr disc. 89
Figure S154. Experimental IR spectrum of (a*S*,2*S*,2'*S*)-**15** recorded as KBr disc. 89
Figure S155. Experimental IR spectrum of (a*S*,2*S*,2'*S*)-**16** recorded as KBr disc. 90
Figure S156. Experimental IR spectrum of (a*S*,2*S*,2'*S*)-**17** recorded as KBr disc. 90
Figure S157. Experimental IR spectrum of (a*S*,3*S*,3'*S*)-**19** recorded as KBr disc. 91
Figure S158. Experimental IR spectrum of (a*S*,3*S*,3'*S*)-**18** recorded as KBr disc. 91
Figure S159. Experimental IR spectrum of *cis*,*cis*-(a*S*,1*R*,3*S*,1'*R*,3'*S*)-**20** recorded as KBr disc......... 92
Figure S160. Experimental IR spectrum of *trans*,*cis*-(a*S*,1*S*,3*S*,1'*R*,3'*S*)-**20** recorded as KBr disc...... 92
Figure S161. Experimental IR spectrum of *cis*,*trans*-(a*S*,1*R*,3*S*,1'*S*,3'*S*)-**20** recorded as KBr disc...... 93
Figure S162. Experimental IR spectrum of *trans*,*trans*-(a*S*,1*S*,3*S*,1'*S*,3'*S*)-**20** recorded as KBr disc. . 93
Figure S163. Experimental IR spectrum of *cis*,*cis*-(a*S*,1*R*,3*S*,1'*R*,3'*S*)-**21** recorded as KBr disc......... 94
Figure S164. Experimental IR spectrum of *cis*,*trans*-(a*S*,1*R*,3*S*,1'*S*,3'*S*)-**21** recorded as KBr disc...... 94
Figure S165. Experimental IR spectrum of *trans*,*cis*-(a*S*,1*S*,3*S*,1'*R*,3'*S*)-**21** recorded as KBr disc...... 95
Figure S166. Experimental IR spectrum of *cis*,*cis*-(a*R*,1*R*,3*S*,1'*R*,3'*S*)-**21** recorded as KBr disc......... 95
Figure S167. Experimental IR spectrum of *cis*,*cis*-(a*S*,1*R*,3*S*,1'*R*,3'*S*)-**22** recorded as KBr disc......... 96
Figure S168. Experimental IR spectrum of *cis*,*trans*-(a*S*,1*R*,3*S*,1'*S*,3'*S*)-**22** recorded as KBr disc...... 96
Figure S169. Experimental IR spectrum of *trans*,*cis*-(a*S*,1*S*,3*S*,1'*R*,3'*S*)-**22** recorded as KBr disc...... 97
Figure S170. Experimental IR spectrum of *trans*,*trans*-(a*S*,1*S*,3*S*,1'*S*,3'*S*)-**22** recorded as KBr disc. . 97
Figure S171. Experimental IR spectrum of *trans*,*trans*-(a*R*,1*S*,3*S*,1'*S*,3'*S*)-**23** recorded as KBr disc.. 98
Figure S172. Experimental IR spectrum of *trans*,*cis*-(a*S*,1*S*,3*S*,1'*R*,3'*S*)-**23** recorded as KBr disc...... 98
Figure S173. Experimental IR spectrum of *trans*,*trans*-(a*S*,1*S*,3*S*,1'*S*,3'*S*)-**23** recorded as KBr disc. . 99
Figure S174. Experimental IR spectrum of *cis*-(a*S*,2*S*,1'*R*,3'*S*)-**24** recorded as KBr disc................... 99
Figure S175. Experimental IR spectrum of *trans*,*cis*-(a*S*,1*S*,3*S*,1'*R*,3'*S*)-**25** recorded as KBr disc.... 100
Figure S176. Experimental IR spectrum of *cis*,*cis*-(a*S*,1*R*,3*S*,1'*R*,3'*S*)-**26** recorded as KBr disc....... 100
Figure S177. Experimental IR spectrum of *cis*,*cis*-(a*S*,1*R*,3*S*,1'*R*,3'*S*)-**27** recorded as KBr disc....... 101
Figure S178. Experimental ESI-HRMS spectrum of (*S*)-**11**. 102

Figure S179. Experimental ESI-HRMS spectrum of (*S*)-**6**. 102
Figure S180. Experimental ESI-HRMS spectrum of (*S*)-**12**. 102
Figure S181. Experimental ESI-HRMS spectrum of (*S*)-**7**. 103
Figure S182. Experimental ESI-HRMS spectrum of (*S*)-**2**. 103
Figure S183. Experimental ESI-HRMS spectrum of (*S*)-**13**. 103
Figure S184. Experimental ESI-HRMS spectrum of (*S*)-**9**. 104
Figure S185. Experimental ESI-HRMS spectrum of (*S*)-**8a** and (*S*)-**8b**. 104
Figure S186. Experimental ESI-HRMS spectrum of (*S*)-**8c**. 104
Figure S187. Experimental ESI-HRMS spectrum of (*S*)-**14**. 104
Figure S188. Experimental ESI-HRMS spectrum of (*S*)-**3**. 105
Figure S189. Experimental ESI-HRMS spectrum of (a*S*,2*S*,2'*S*)-**15**. 105
Figure S190. Experimental ESI-HRMS spectrum of (a*S*,2*S*,2'*S*)-**16**. 105
Figure S191. Experimental ESI-HRMS spectrum of (a*S*,2*S*,2'*S*)-**17**. 106
Figure S192. Experimental ESI-HRMS spectrum of (a*S*,3*S*,3'*S*)-**19**. 106
Figure S193. Experimental ESI-HRMS spectrum of (a*S*,3*S*,3'*S*)-**18**. 106
Figure S194. Experimental ESI-HRMS spectrum of *cis*,*cis*-(a*S*,1*R*,3*S*,1'*R*,3'*S*)-**20**. 107
Figure S195. Experimental ESI-HRMS spectrum of *cis*,*cis*-(a*S*,1*R*,3*S*,1'*R*,3'*S*)-**21**. 107
Figure S 196. Experimental ESI-HRMS spectrum of *cis*,*trans*-(a*S*,1*R*,3*S*,1'*S*,3'*S*)-**22**............ 107
Figure S197. Experimental ESI-HRMS spectrum of *trans*,*trans*-(a*S*,1*S*,3*S*,1'*S*,3'*S*)-**23**. 108
Figure S198. Experimental ESI-HRMS spectrum of *cis*-(a*S*,2*S*,1'*R*,3'*S*)-**24**............ 108
Figure S199. Experimental ESI-HRMS spectrum of *trans*,*cis*-(a*S*,1*S*,3*S*,1'*R*,3'*S*)-**25**............ 108
Figure S200. Experimental ESI-HRMS spectrum of *cis*,*cis*-(a*S*,1*R*,3*S*,1'*R*,3'*S*)-**26**. 109
Figure S201. Experimental ESI-HRMS spectrum of *cis*,*cis*-(a*S*,1*R*,3*S*,1'*R*,3'*S*)-**27**. 109
Figure S202. Low-energy conformers (≥1%) of (a*S*,3*S*,3'*S*)-**19** computed at the ωB97X/TZVP PCM/MeCN level............ 110
Figure S203. Low-energy conformers (≥1%) of (a*R*,3*S*,3'*S*)-**19** computed at the ωB97X/TZVP PCM/MeCN level............ 110
Figure S204. Low-energy conformers (≥1%) of (a*S*,3*S*,3'*S*)-**19** computed at the B3LYP/TZVP PCM/$CHCl_3$ level............ 111
Figure S205. Low-energy conformers (≥1%) of (a*R*,3*S*,3'*S*)-**19** computed at the B3LYP/TZVP PCM/$CHCl_3$ level. 111
Figure S206. Overlapped conformers in the asymmetric unit of *cis*,*cis*-(a*S*,1*R*,3*S*,1'*R*,3'*S*)-**20** showing conformational differences. Hydrogen atoms are omitted for clarity. 126

1.2. List of Tables

Table S1. Cartesian coordinates and energies of the low-energy conformers calculated at the ωB97X/TZVP PCM/MeCN level. 112

Table S2. Cartesian coordinates and energies of the low-energy conformers calculated at the B3LYP/TZVP PCM/$CHCl_3$ level.. 119

Table S3. Experimental details of structure determination. 124

Table S4. Experimental details of structure determination. 125

2. General Information

Chemicals were purchased puriss p.a. from commercial suppliers. The indicated higher/lower temperature values (°C) other than room temperature (25 °C) for the reactions were referred to the temperature of the heating/cooling units (oil bath, iced water, acetone cooled by liquid nitrogen). Thin layer chromatography (TLC) was performed on Silica gel 60 F_{254} (Merck) with visualization by UV-light (254 nm) and immersed into ethanolic solution of sulfuric acidic vanillin (2 g vanillin and 2 ml cc. H_2SO_4 in 98 ml 96% ethanol) followed by heating. Column chromatography was performed on Silica gel 60 (Merck 0.040-0.063 mm for flash column chromatography and 0.063-0.200 mm for conventional column chromatography). Melting points were determined on a Kofler hot-stage apparatus and are uncorrected. Anhydrous solvents were used for all the reactions and distilled solvents were used as eluent for column chromatography. HPLC-grade solvents were used for chiral HPLC separations. Preparative chiral HPLC was performed by Agilent 1260 Infinity II apparatus using Lux i-Amylose-5 and Lux i-Cellulose-5 columns.

The ^{1}H NMR (360 MHz, 400 MHz, 500 MHz, 700 MHz) and ^{13}C NMR (90 MHz, 100 MHz, 125 MHz, 175 MHz) spectra were recorded with Bruker Avance DRX 360 MHz, Bruker Avance I 400 MHz, Bruker Avance II 500 MHz and Bruker Avance Neo 700 MHz spectrometers at 298 K. Chemical shifts are referenced to Me_4Si ($CDCl_3$, acetone-d_6: 0.00 ppm for ^{1}H) and to the residual solvent signals ($CDCl_3$: 77.16 ppm for ^{13}C, acetone-d_6: 29.84 ppm for ^{13}C, acetonitril-d_3: 2.13 ppm for ^{1}H and 118.26 ppm for ^{13}C). Chemical shifts were reported as δ in ppm, and $^{1}J_{C\text{-}F}$, $^{2}J_{H\text{-}H}$, $^{2}J_{C\text{-}F}$, $^{3}J_{H\text{-}H}$, $^{3}J_{C\text{-}F}$, $^{4}J_{H\text{-}H}$ and $^{4}J_{C\text{-}F}$ coupling constants in Hz. IR spectra were recorded on a JASCO FT/IR-4100 spectrometer and absorption bands are presented as wavenumber in cm^{-1}. Optical rotations were measured at room temperature with a Perkin-Elmer 241 automatic polarimeter (*c* [g/100ml]). ECD spectra were recorded on a J-810 spectropolarimeter. VCD measurements were performed on a BioTools ChiralIR-2X spectrometer at a resolution of 4 cm^{-1} under ambient temperature for 18 x 3000 scans, respectively. Sample were dissolved in $CDCl_3$ and the solutions were placed in a 100 μm BaF_2 cell. For spectroscopic measurements spectroscopic grade solvents were used. For single-crystal X-ray diffraction studies the crystals were grown from the corresponding crystallization solvents/solvent mixtures by slow evaporation of the solvents. Diffraction intensity data were collected at room temperature using a Bruker-D8 Venture diffractometer (Bruker AXS GmbH, Karlsruhe, Germany) equipped with INCOATEC IμS 3.0 (Incoatec GmbH, Geesthacht, Germany) dual (Cu and Mo) sealed tube micro sources and a Photon II Charge-Integrating Pixel Array detector (Bruker AXS GmbH, Karlsruhe, Germany) using Mo Kα (λ = 0.71073 Å) radiation. Electrospray quadrupole time-of-flight HRMS measurements were performed with a

maXis II UHR ESI-QTOF MS instrument from Bruker (Bruker Daltoniks, Bremen, Germany).

2.1. Computational section

Mixed torsional/low-frequency mode conformational searches were carried out by means of the Macromodel 10.8.011 software, using the Merck Molecular Force Field (MMFF) with an implicit solvent model for $CHCl_3$. [1] All quantum chemical calculations were carried out with the Gaussian 09 software package. [2,3] The B3LYP (VCD) and ωB97X [4] (ECD) functionals with the TZVP basis set and PCM solvent model for $CHCl_3$ (VCD) and MeCN (ECD) were used to re-optimize the initial MMFF geometries. TDDFT-ECD and -OR calculations were performed at the B3LYP/TZVP, BH&HLYP/TZVP, CAM-B3LYP/TZVP and the PBE0/TZVP levels of theory with the PCM solvent model for MeCN. ECD spectra were generated as sums of Gaussians with 3000 cm^{-1} widths at half-height, using dipole-velocity-computed rotational strength values. [5] VCD calculations were performed at the B3LYP/TZVP PCM/$CHCl_3$ level, while the spectra were gained by applying a 8 cm^{-1} half-height width and scaled by a factor of 0.98. Boltzmann distributions were estimated from the B3LYP and ωB97X energies. The MOLEKEL software package was used for visualization of the results. [6]

3. Spectra of the compounds

3.1. NMR spectra

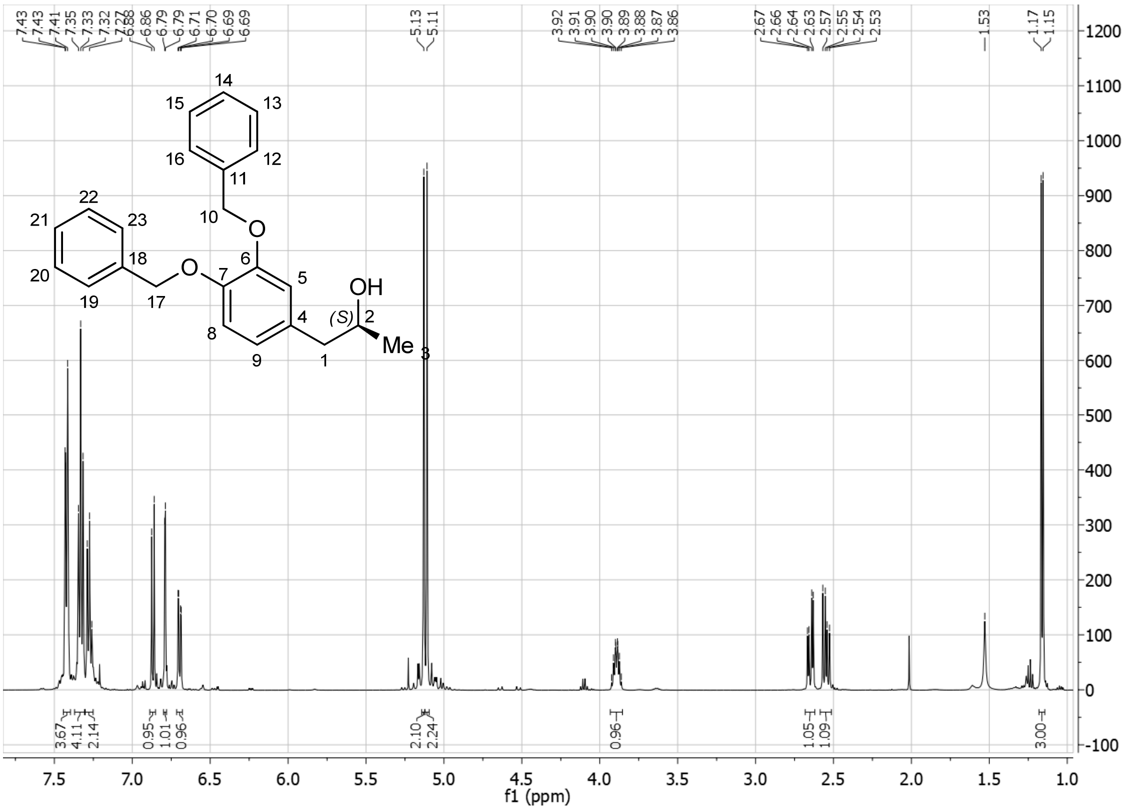

Figure S1. ^{1}H NMR (500 MHz) spectrum of (*S*)-**8** in $CDCl_3$.

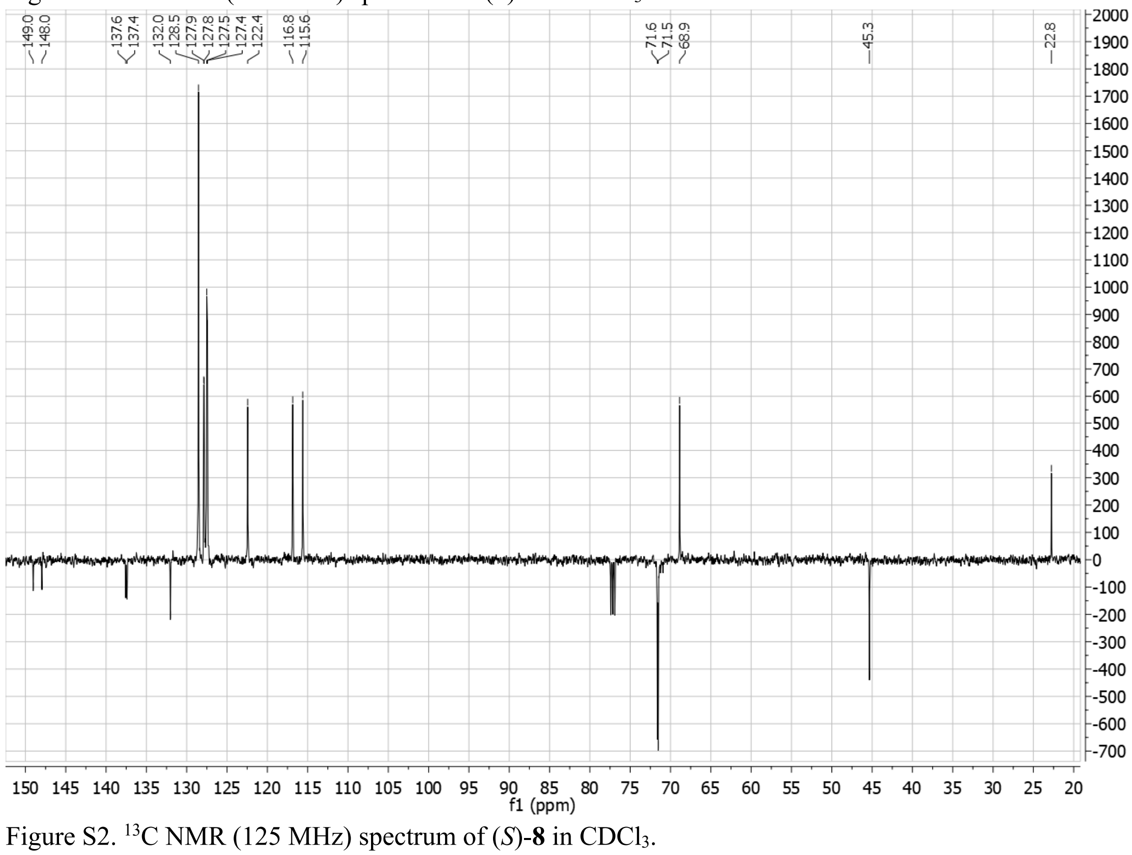

Figure S2. ^{13}C NMR (125 MHz) spectrum of (*S*)-**8** in $CDCl_3$.

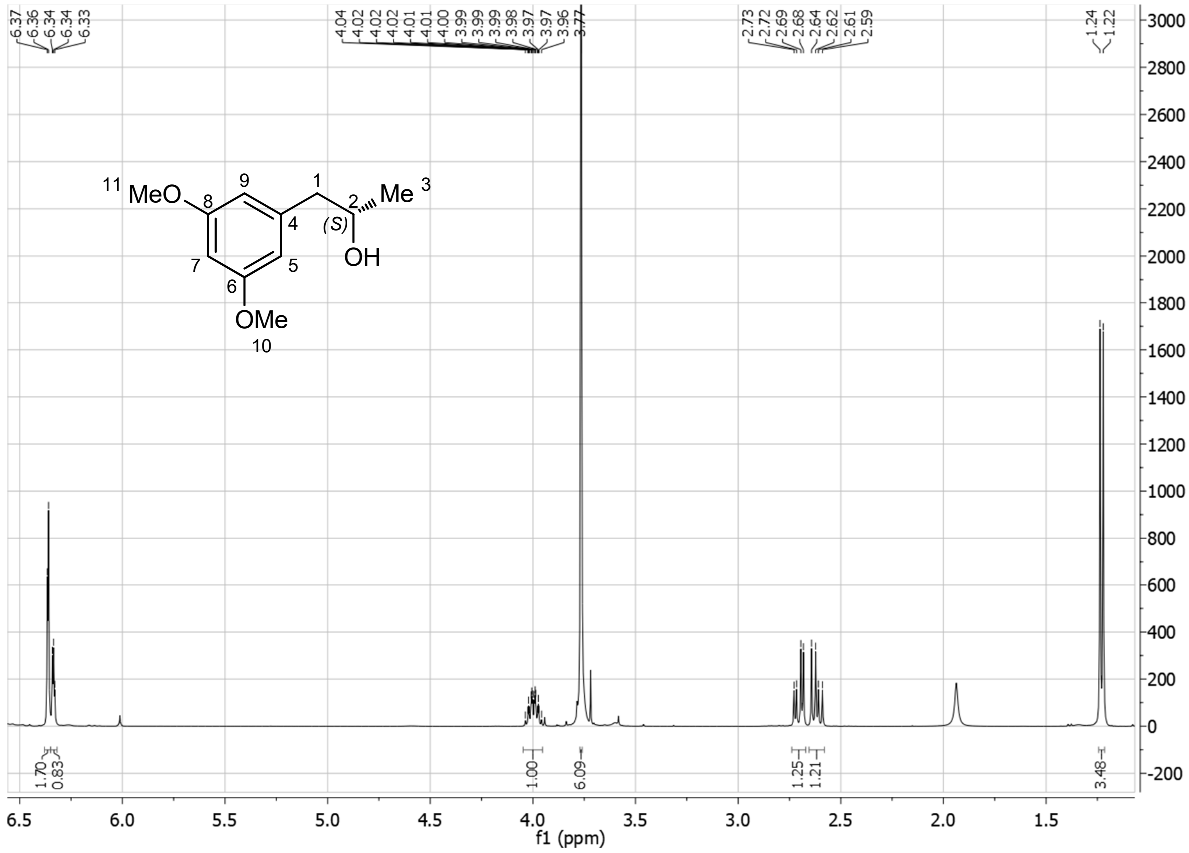

Figure S3. ^{1}H NMR (400 MHz) spectrum of (*S*)-**6** in $CDCl_3$.

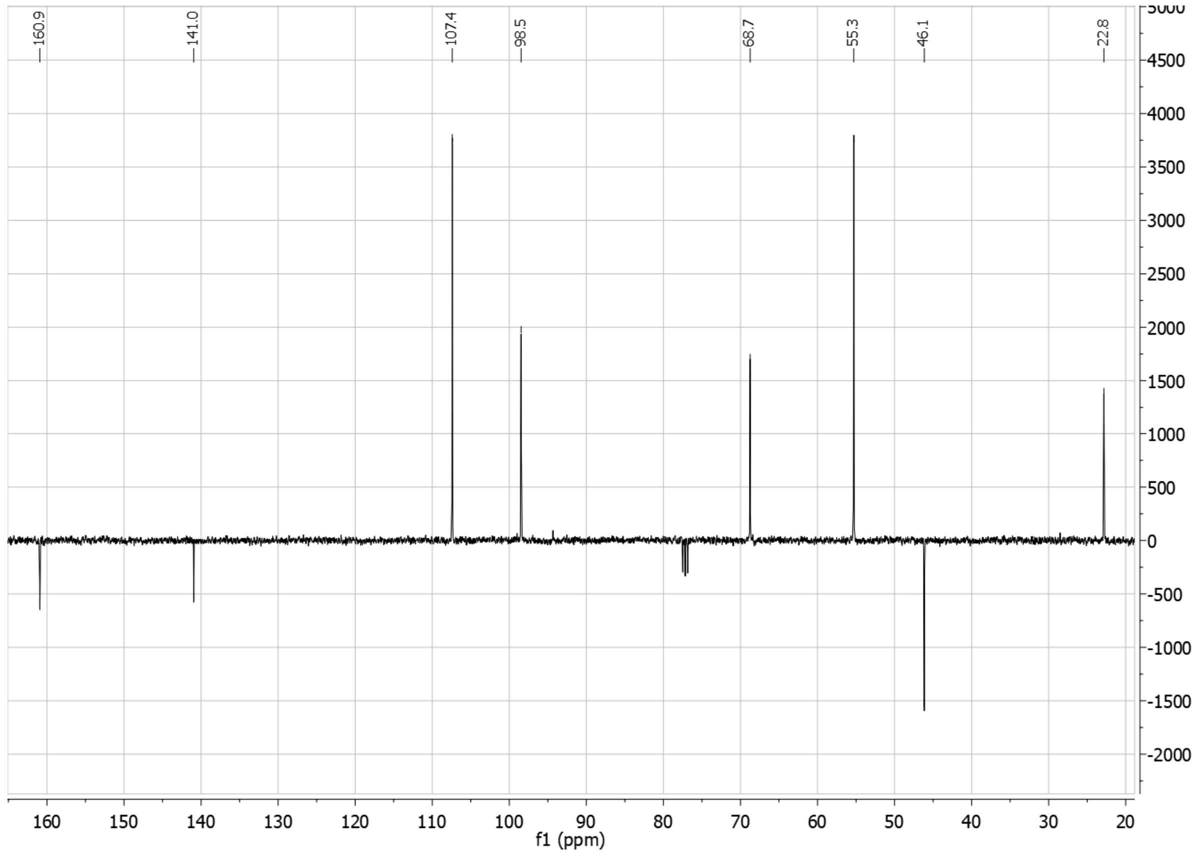

Figure S4. ^{13}C NMR (100 MHz) spectrum of (*S*)-**6** in $CDCl_3$.

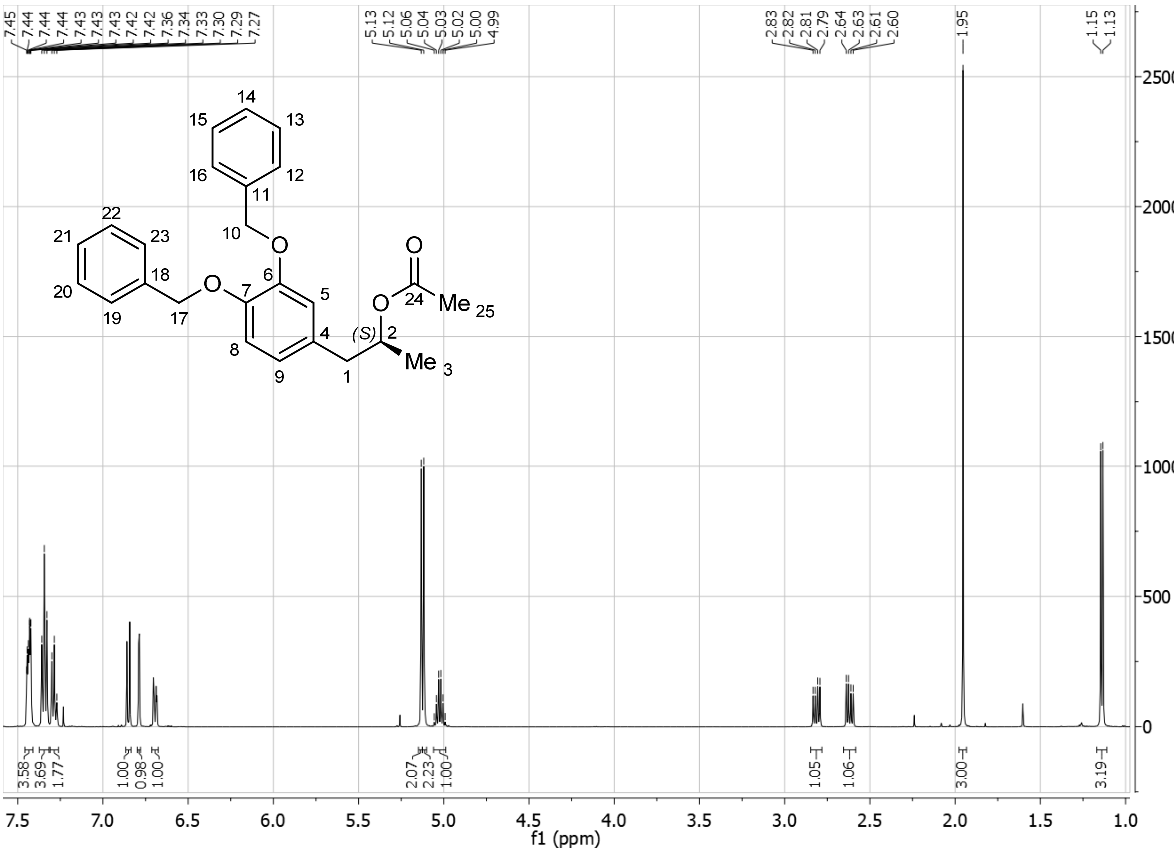

Figure S5. ^{1}H NMR (500 MHz) spectrum of (*S*)-**12** in $CDCl_3$.

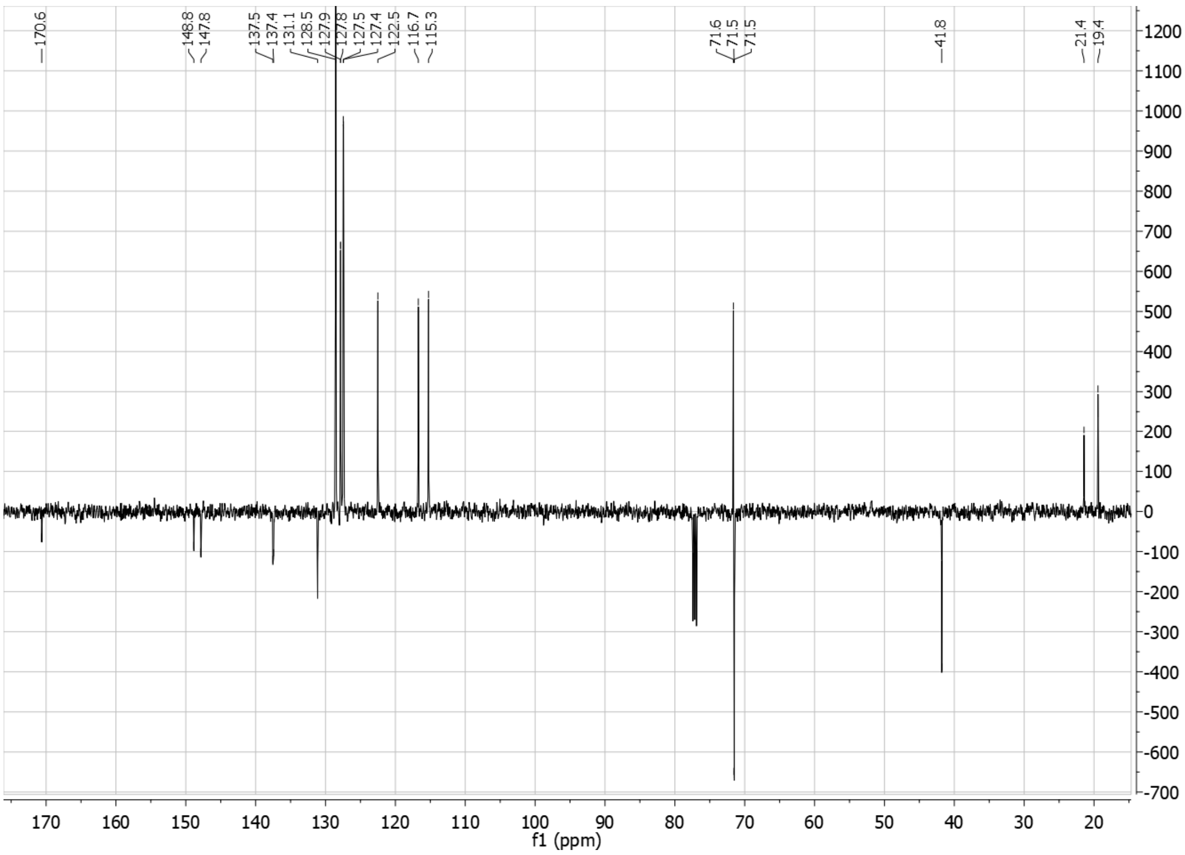

Figure S6. ^{13}C NMR (125 MHz) spectrum of (*S*)-**12** in $CDCl_3$.

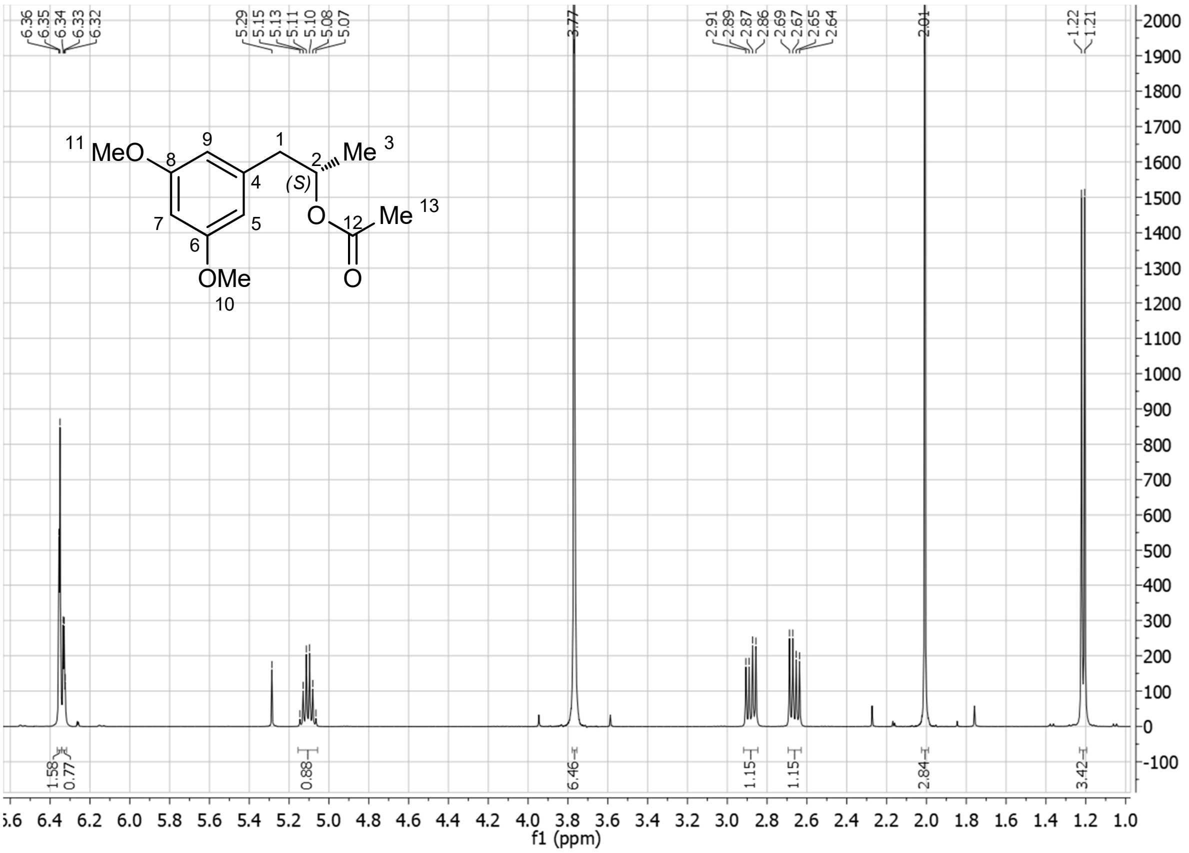

Figure S7. ^{1}H NMR (400 MHz) spectrum of (*S*)-**7** in $CDCl_3$.

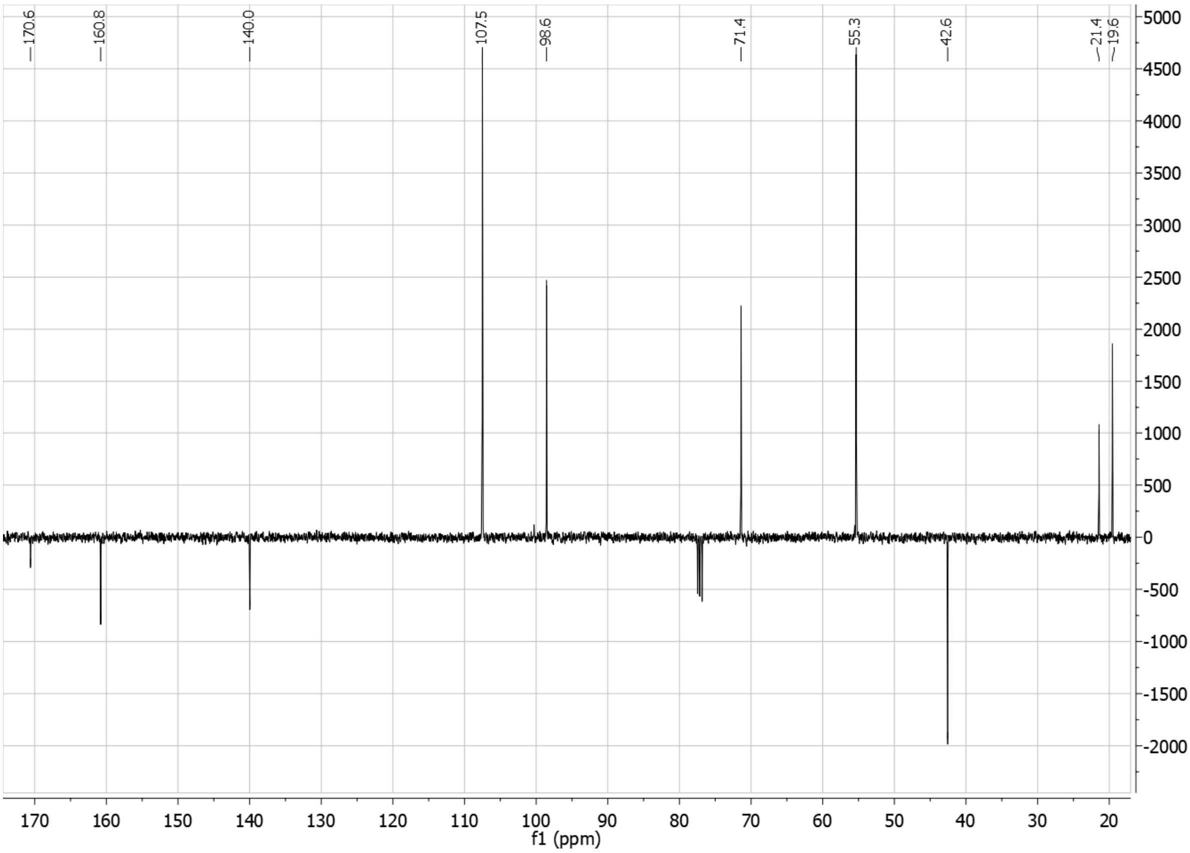

Figure S8. ^{13}C NMR (100 MHz) spectrum of (*S*)-**7** in $CDCl_3$.

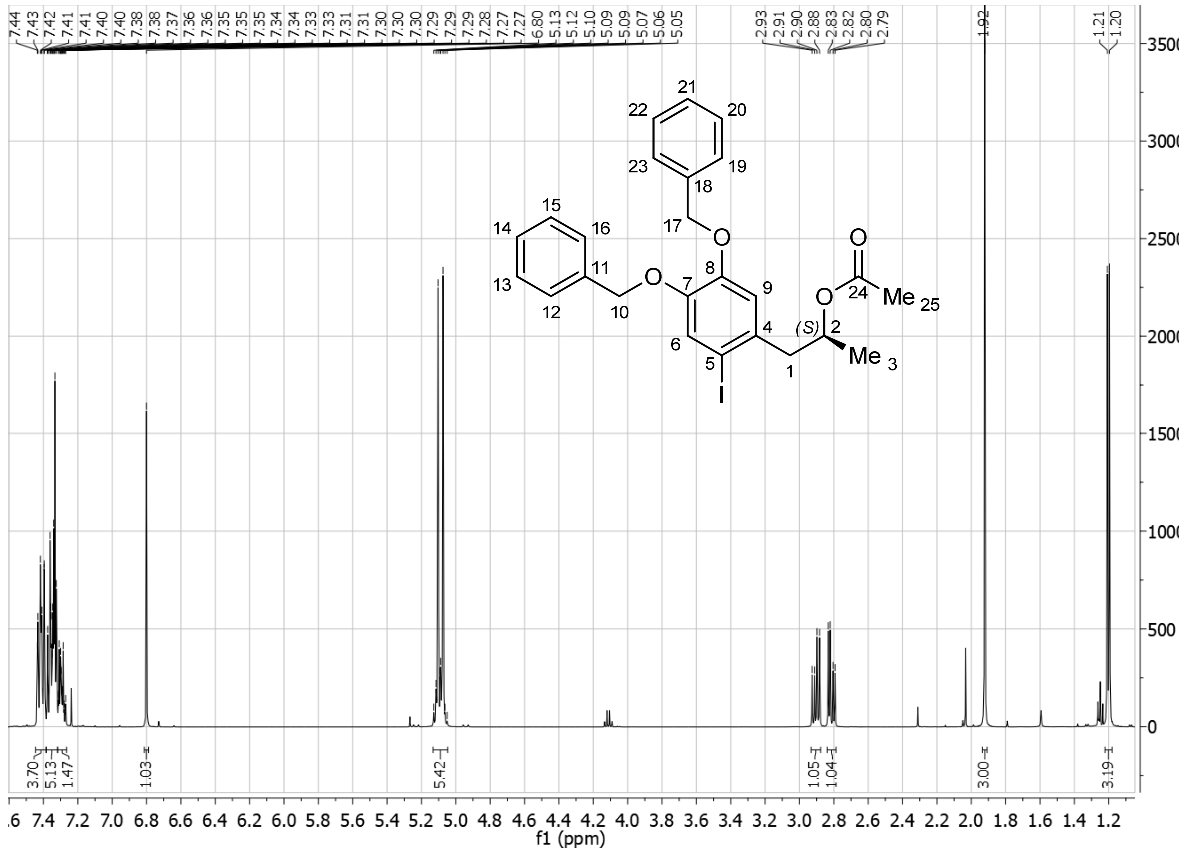

Figure S9. ^{1}H NMR (500 MHz) spectrum of (*S*)-**2** in $CDCl_3$.

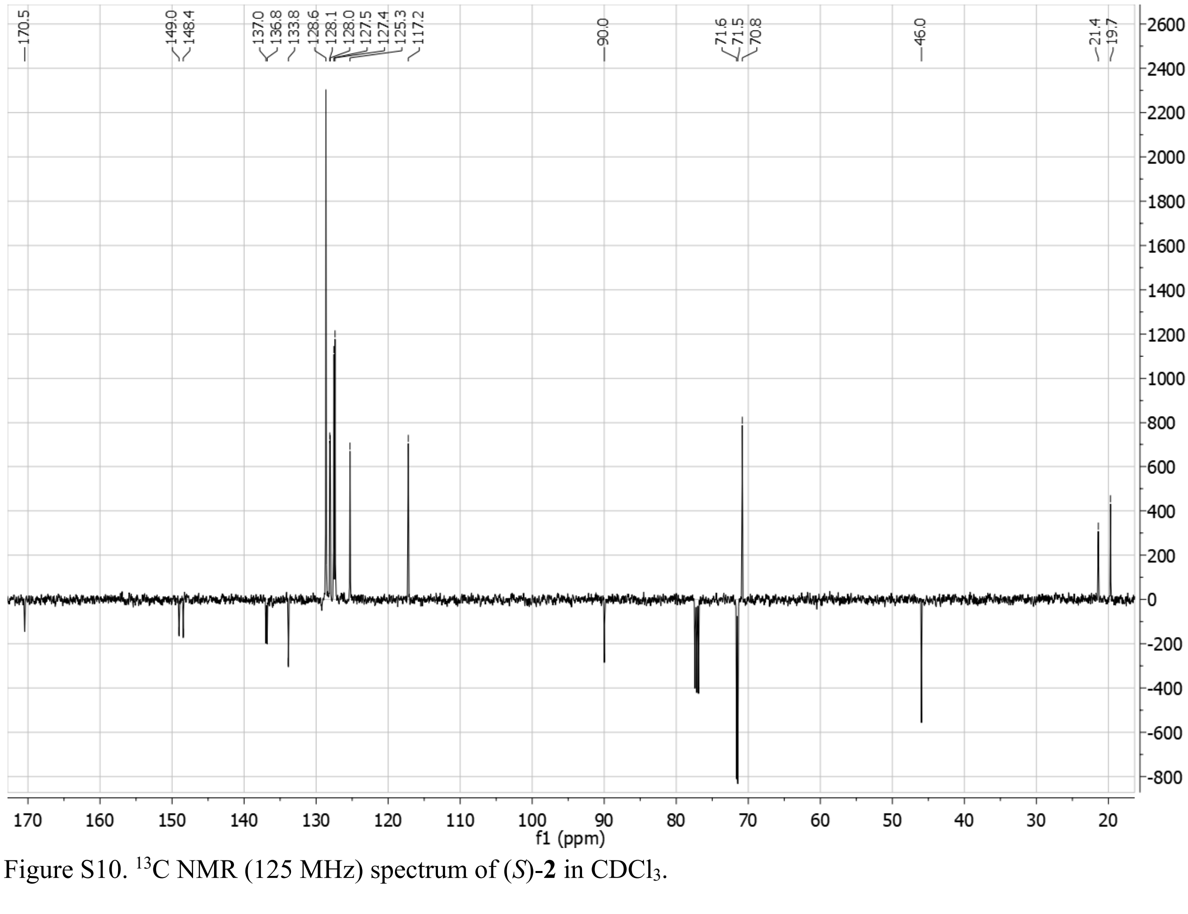

Figure S10. ^{13}C NMR (125 MHz) spectrum of (*S*)-**2** in $CDCl_3$.

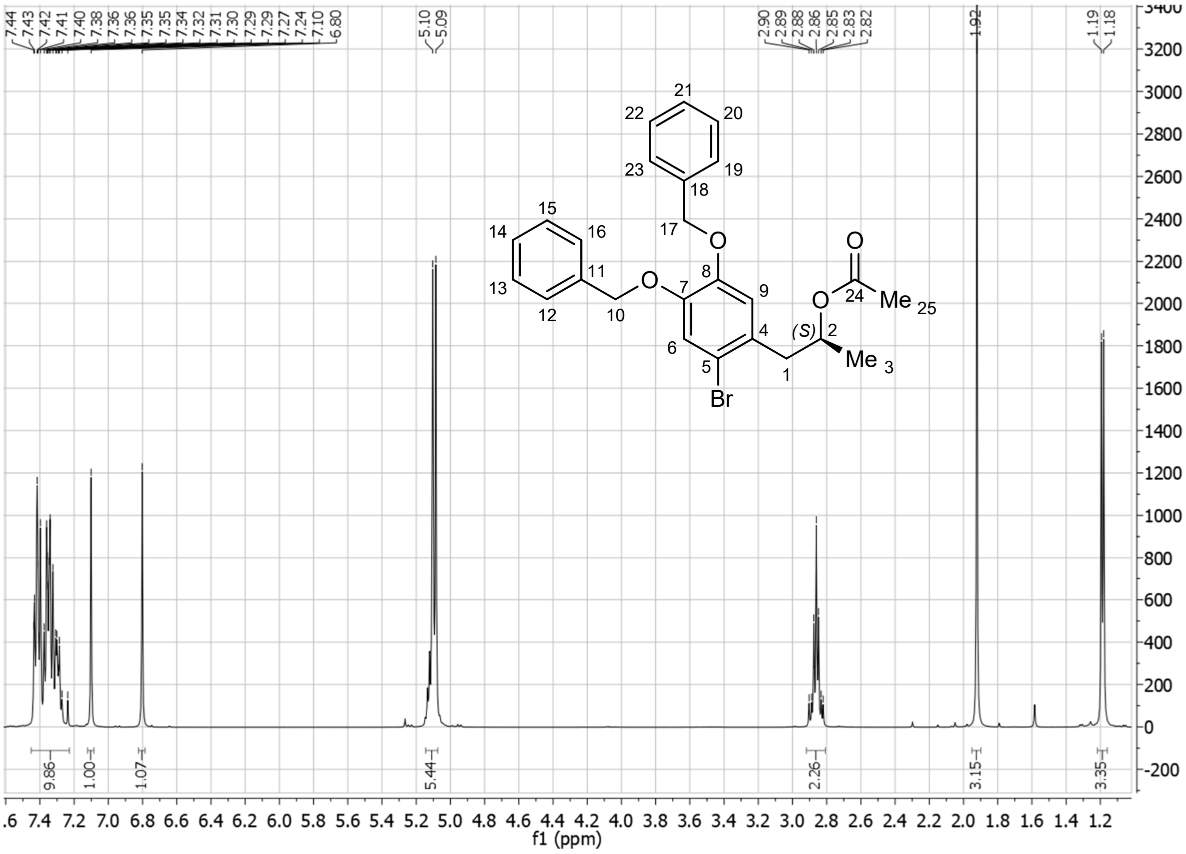

Figure S11. ^{1}H NMR (500 MHz) spectrum of (*S*)-**13** in $CDCl_3$.

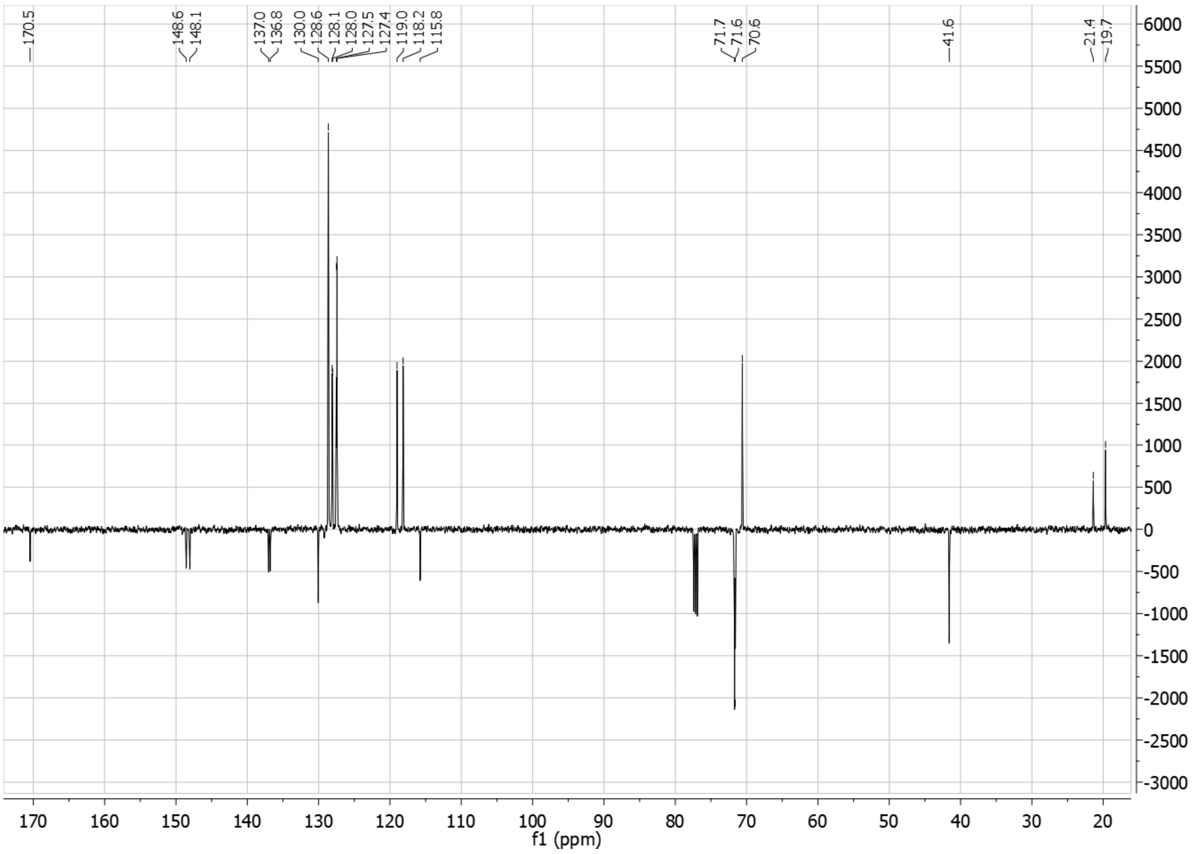

Figure S12. ^{13}C NMR (125 MHz) spectrum of (*S*)-**13** in $CDCl_3$.

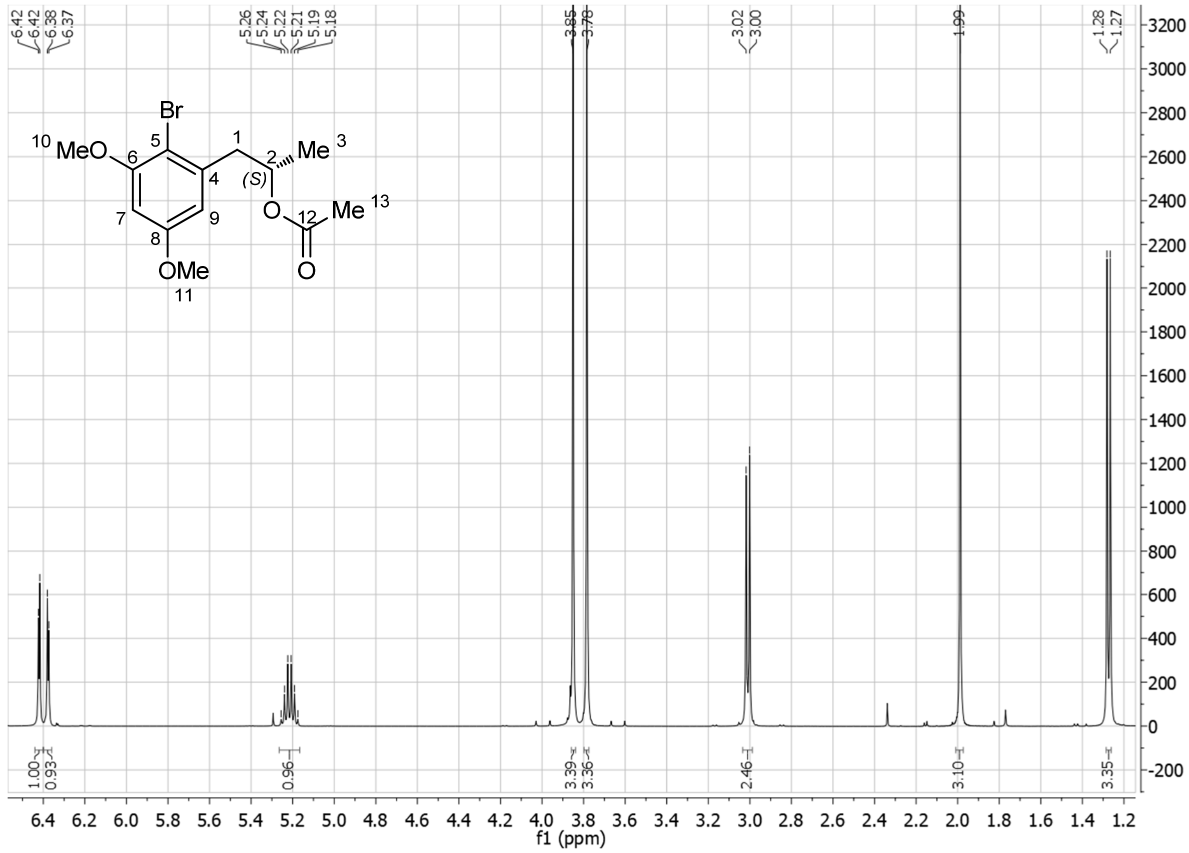

Figure S13. ^{1}H NMR (400 MHz) spectrum of (*S*)-**9** in $CDCl_3$.

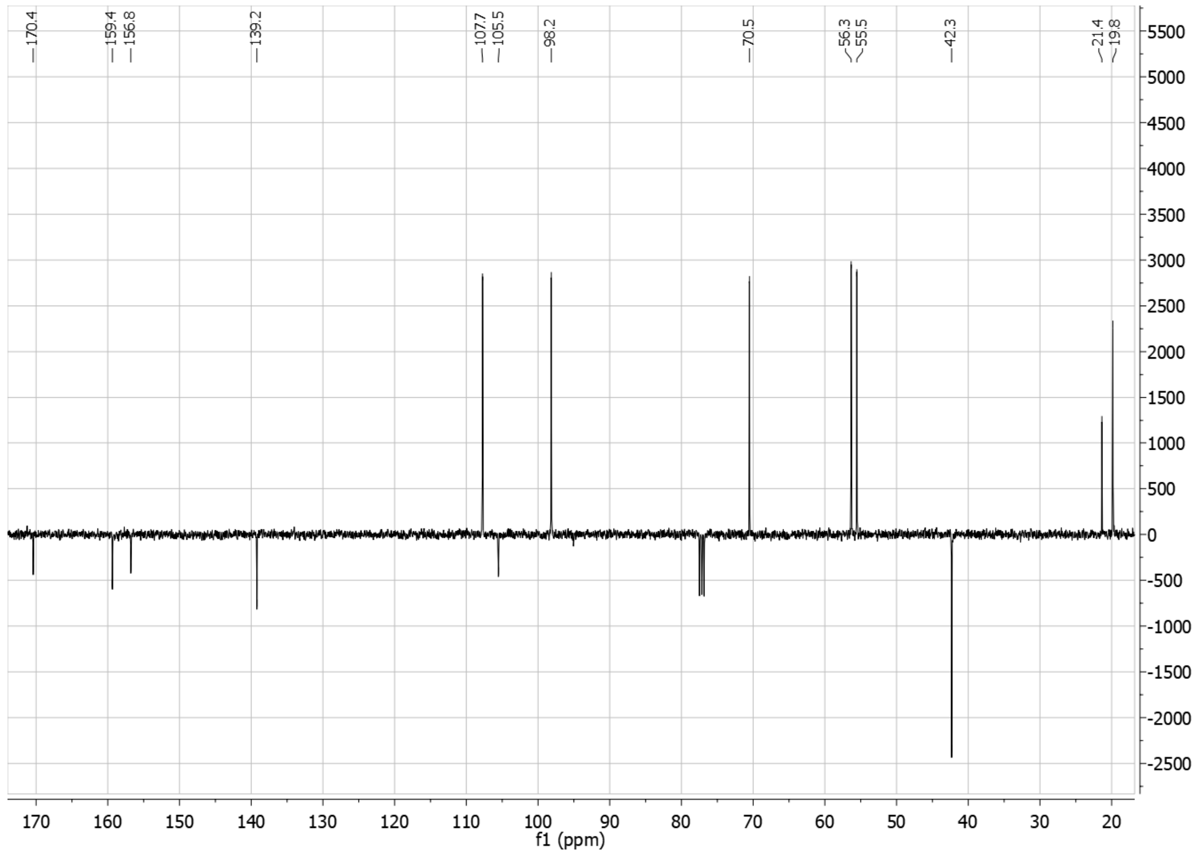

Figure S14. ^{13}C NMR (100 MHz) spectrum of (*S*)-**9** in $CDCl_3$.

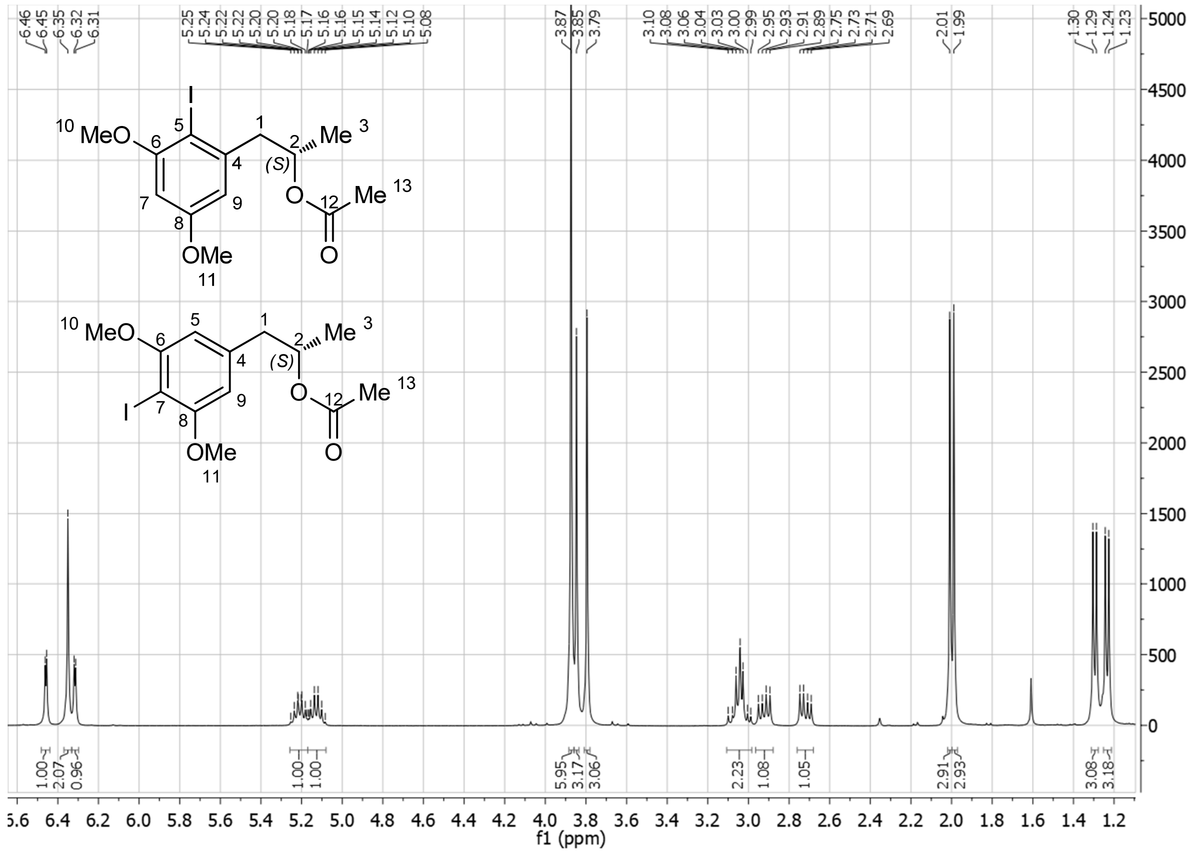

Figure S15. ^{1}H NMR (360 MHz) spectrum of (*S*)-**8a** and (*S*)-**8b** (regioisomers ratio 1:1) in $CDCl_3$.

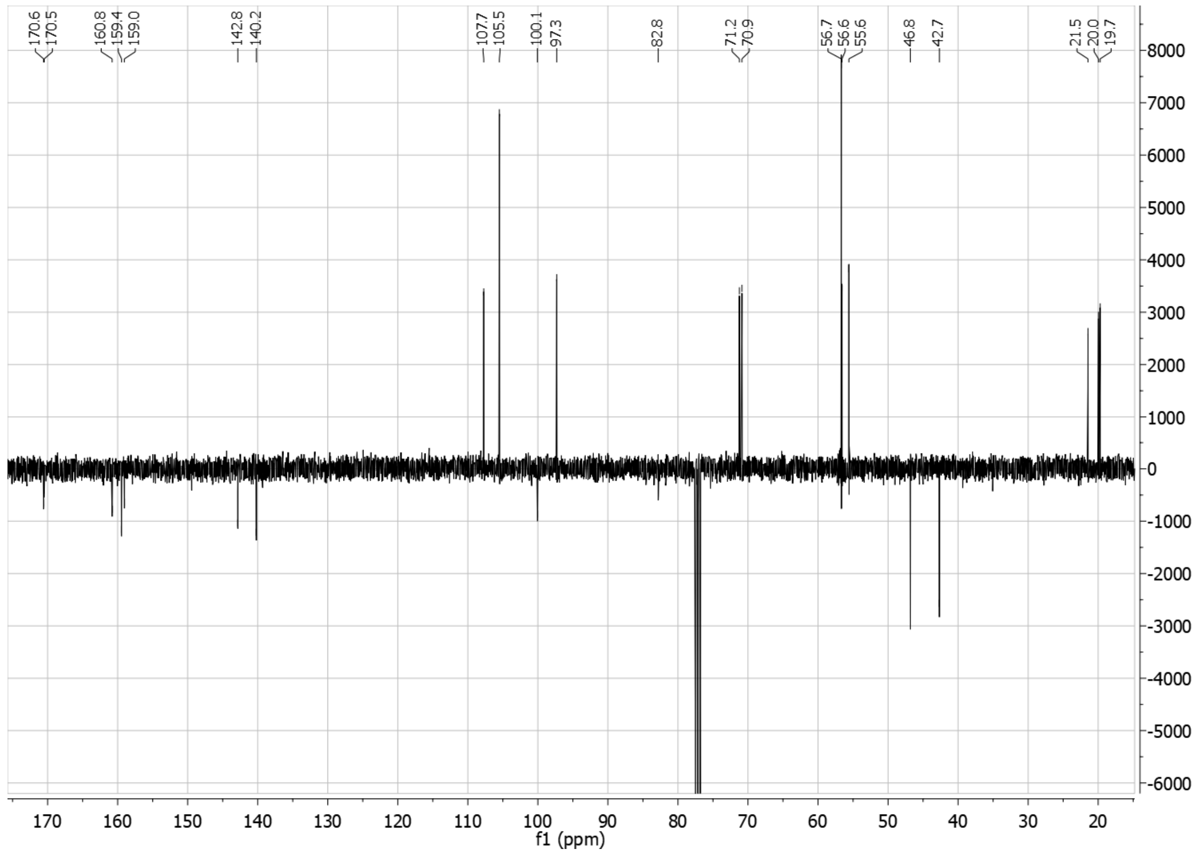

Figure S16. ^{13}C NMR (90 MHz) spectrum of (*S*)-**8a** and (*S*)-**8b** (regioisomers ratio 1:1) in $CDCl_3$.

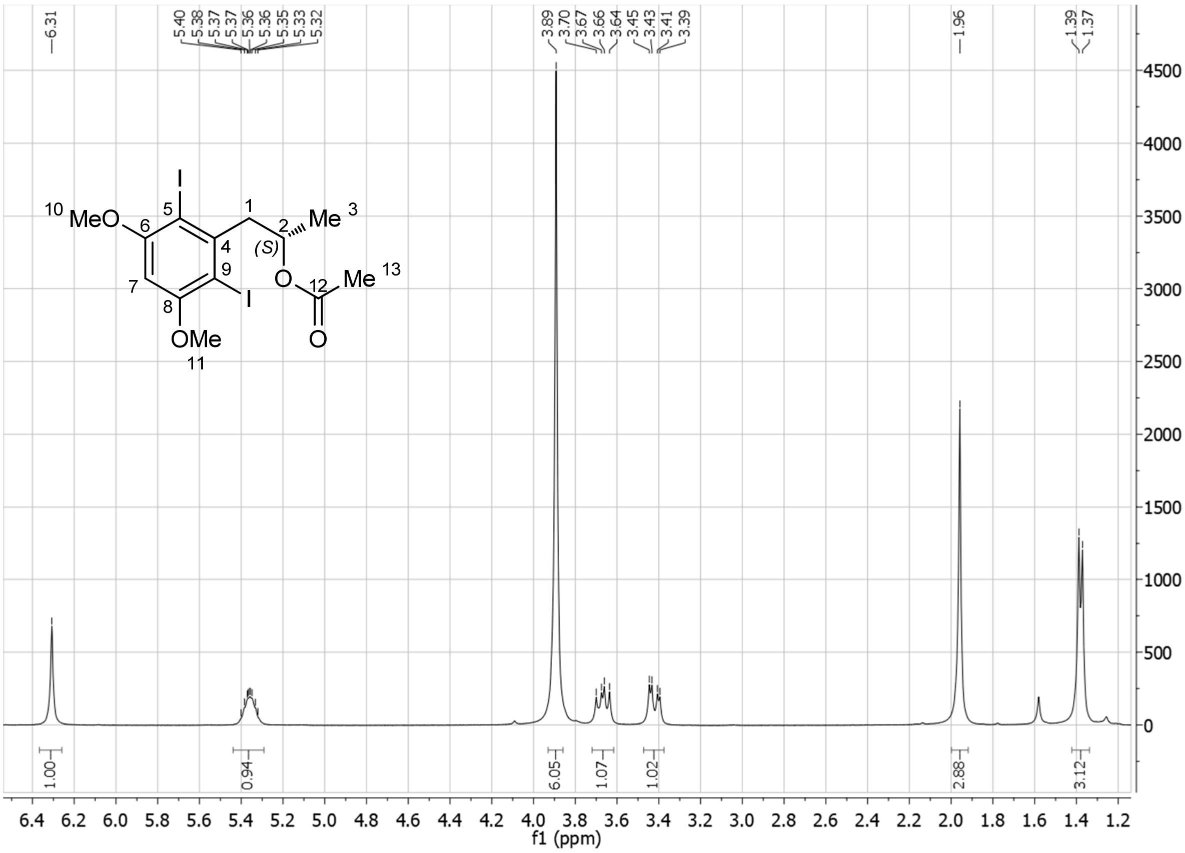

Figure S17. ^{1}H NMR (360 MHz) spectrum of (*S*)-**8c** in $CDCl_3$.

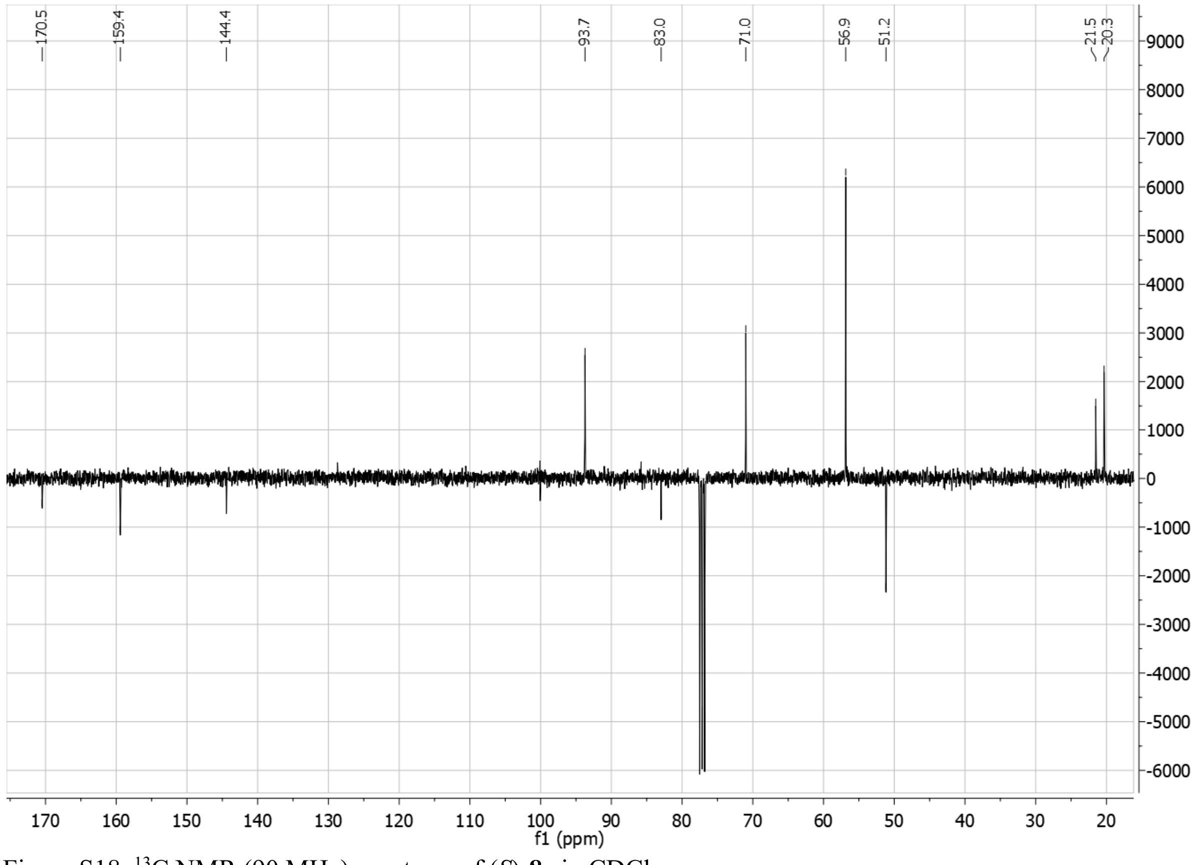

Figure S18. ^{13}C NMR (90 MHz) spectrum of (*S*)-**8c** in $CDCl_3$.

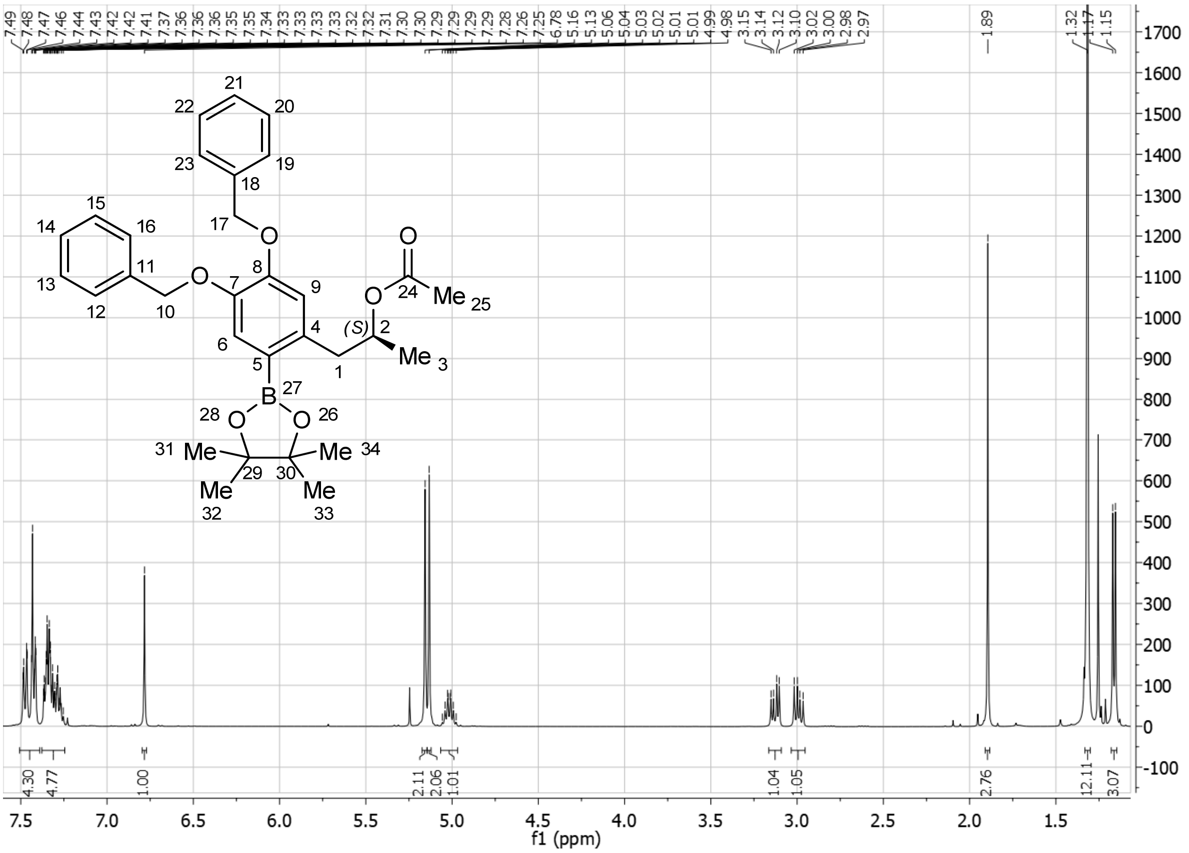

Figure S19. ^{1}H NMR (400 MHz) spectrum of in (S)-**14** $CDCl_3$.

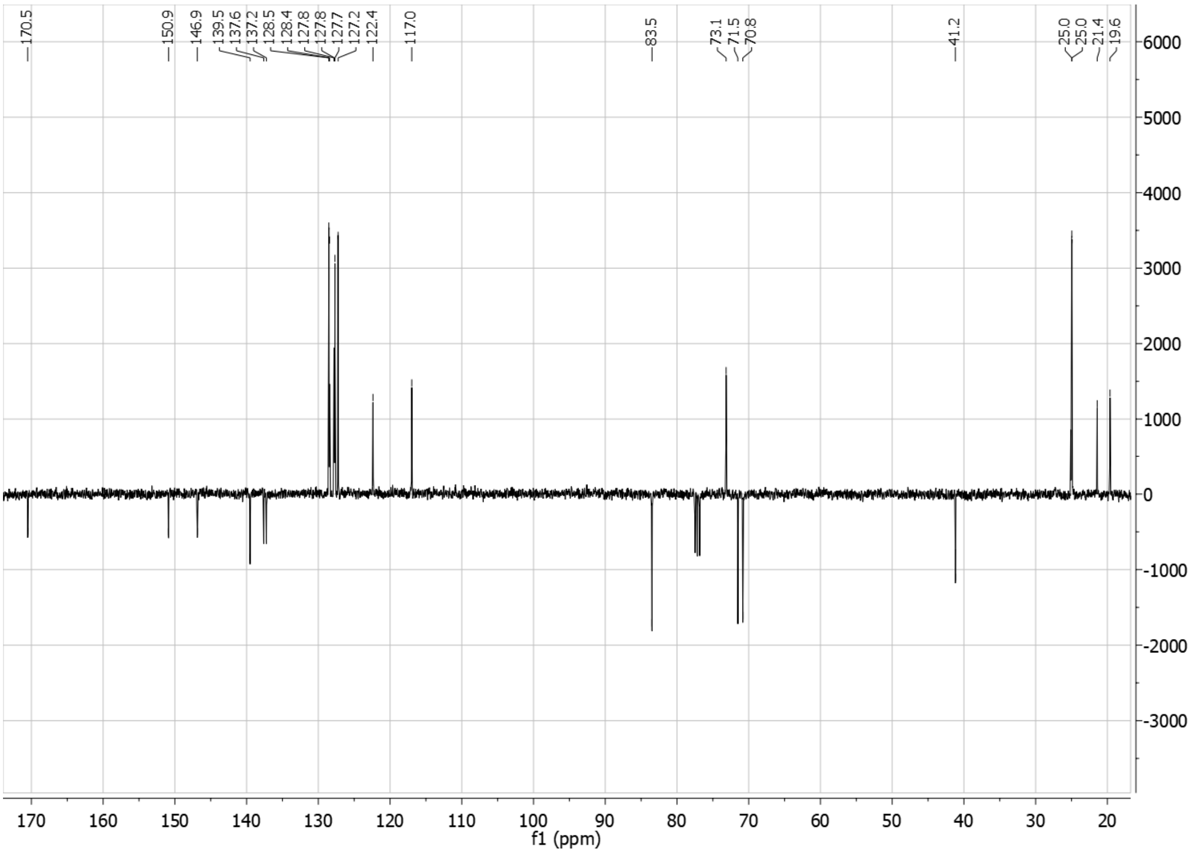

Figure S20. ^{13}C NMR (100 MHz) spectrum of in (S)-**14** $CDCl_3$.

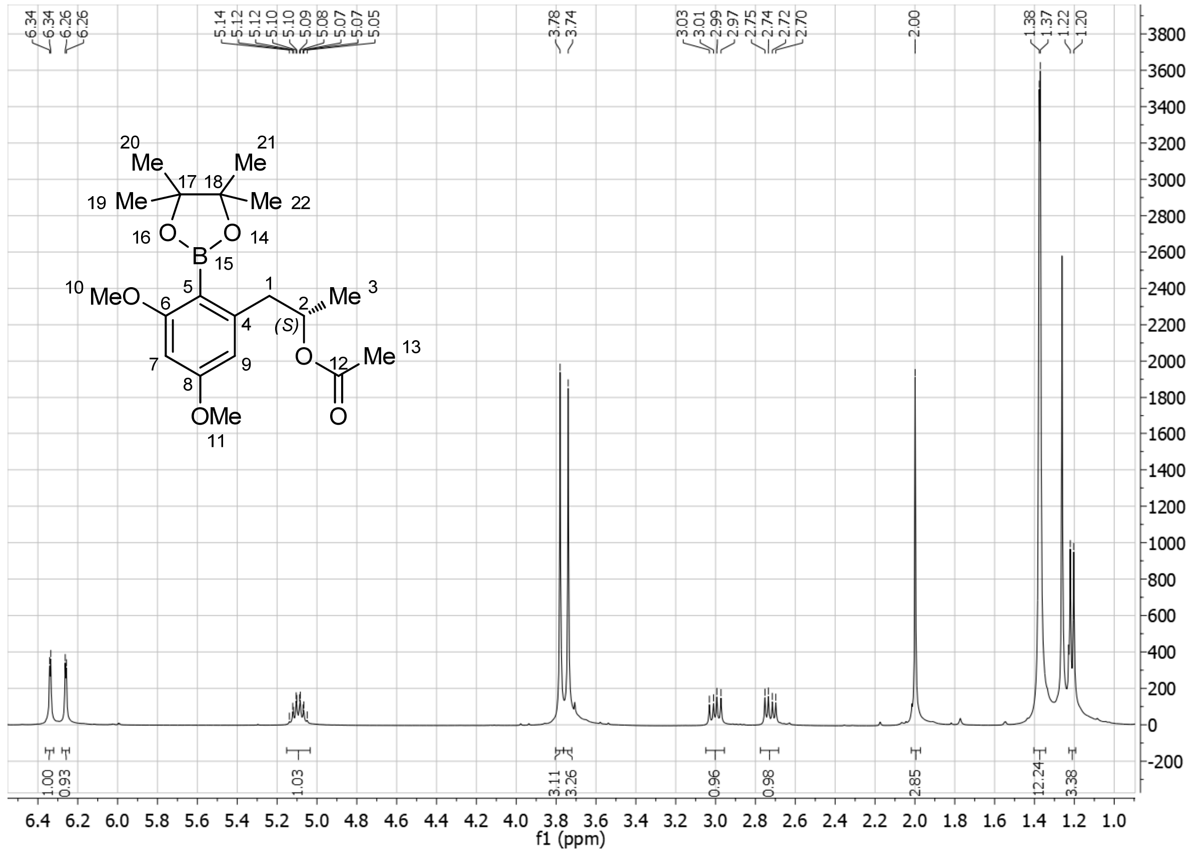

Figure S21. ^{1}H NMR (360 MHz) spectrum of (*S*)-**3** in $CDCl_3$.

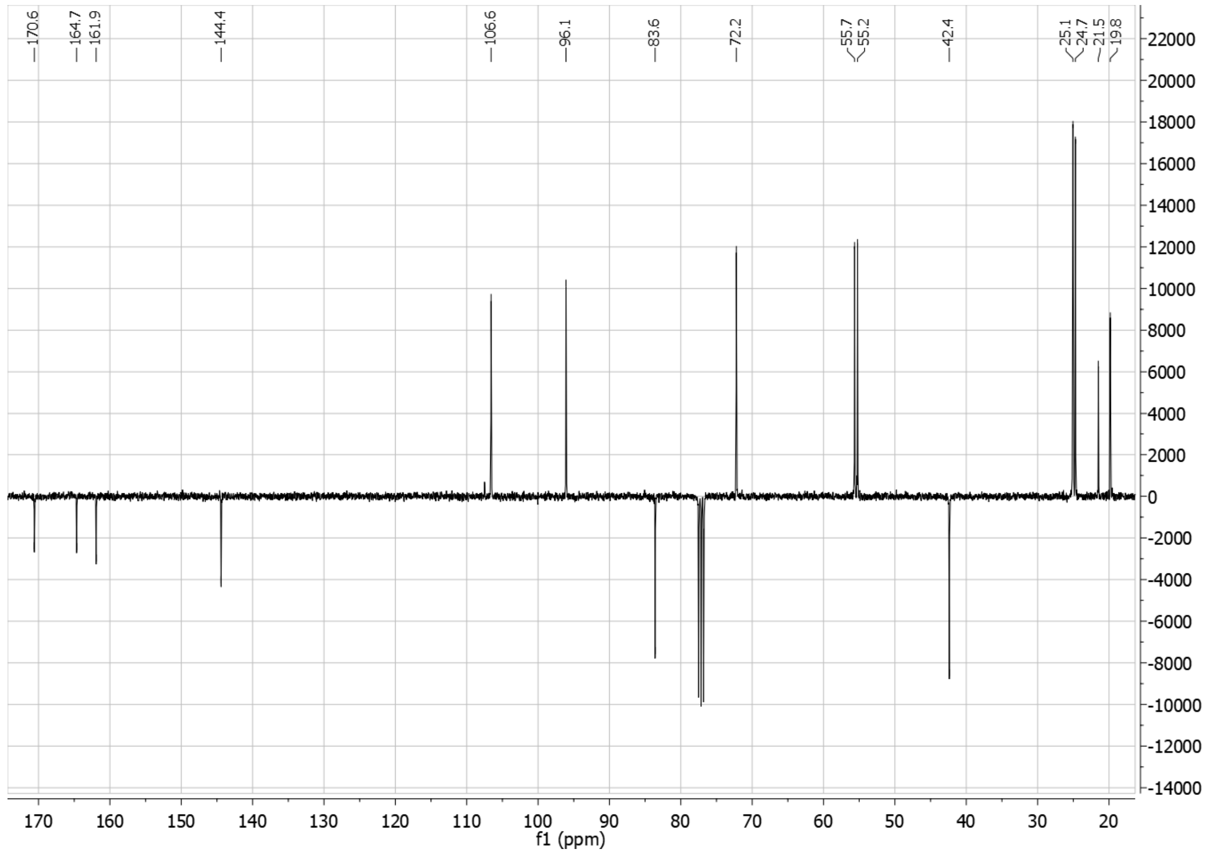

Figure S22. ^{13}C NMR (90 MHz) spectrum of (*S*)-**3** in $CDCl_3$.

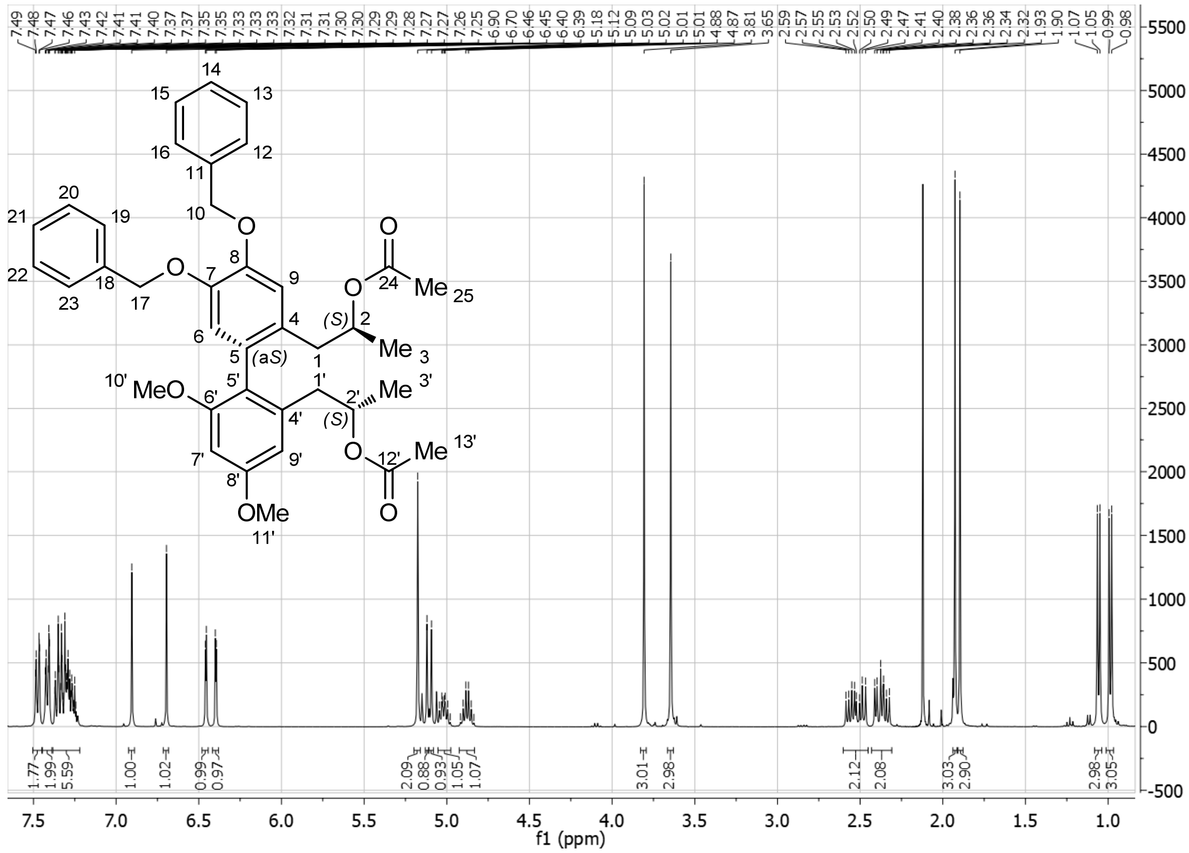

Figure S23. ^{1}H NMR (400 MHz) spectrum of (a*S*,2*S*,2'*S*)-**15** in $CDCl_3$.

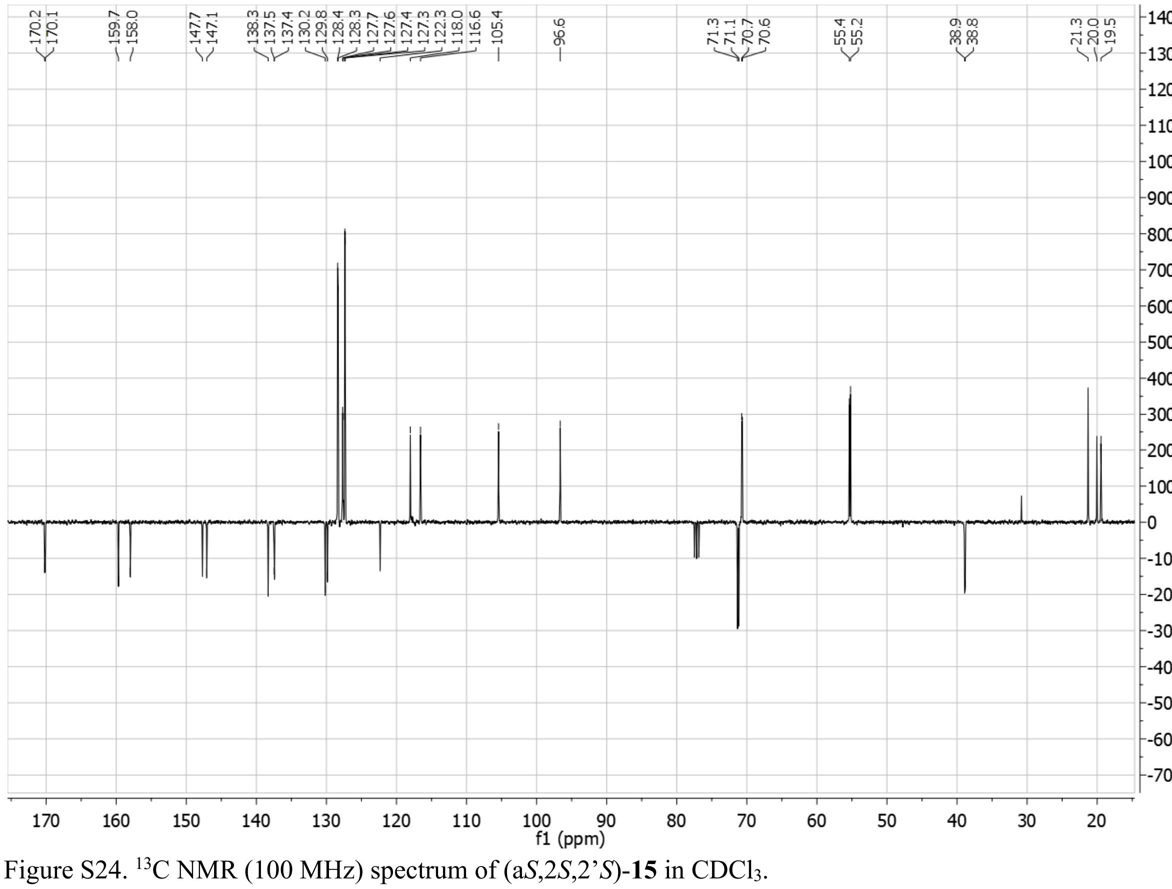

Figure S24. ^{13}C NMR (100 MHz) spectrum of (a*S*,2*S*,2'*S*)-**15** in $CDCl_3$.

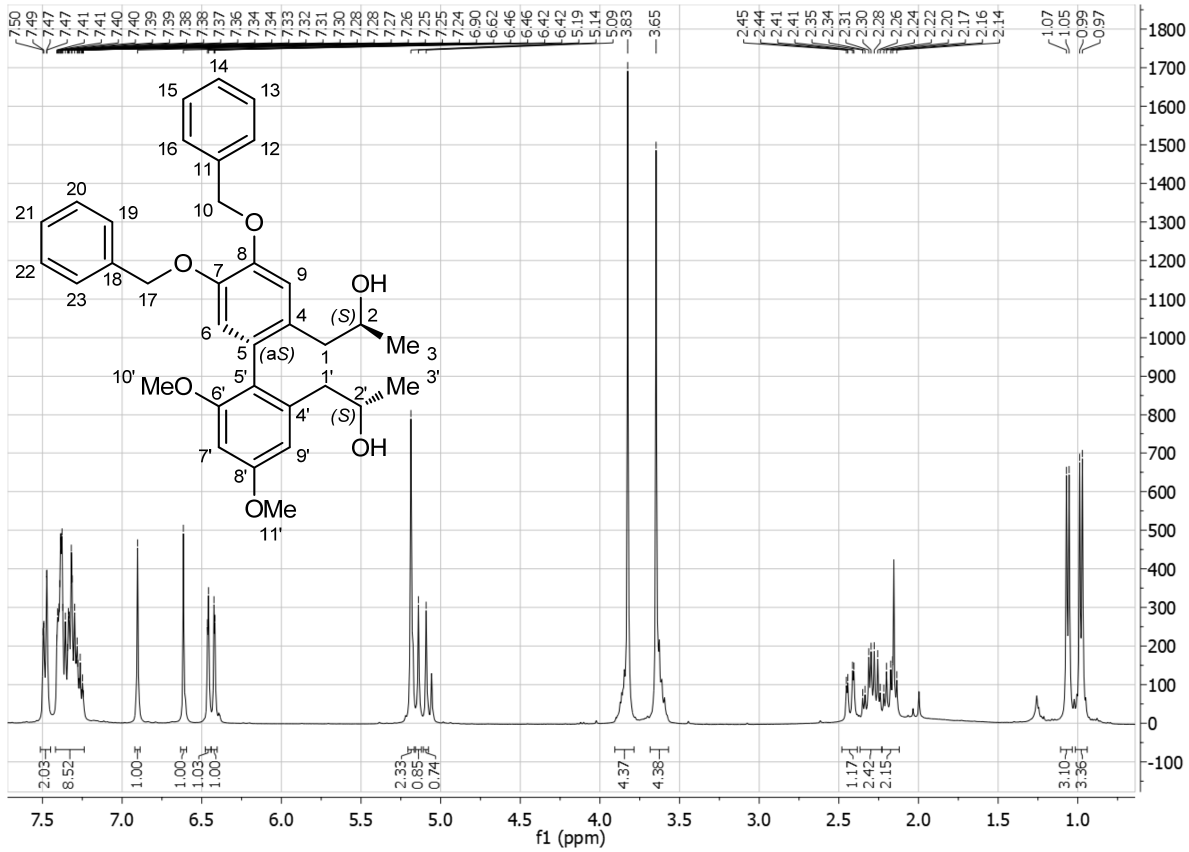

Figure S25. ^{1}H NMR (360 MHz) spectrum of in (a*S*,2*S*,2'*S*)-**16** $CDCl_3$.

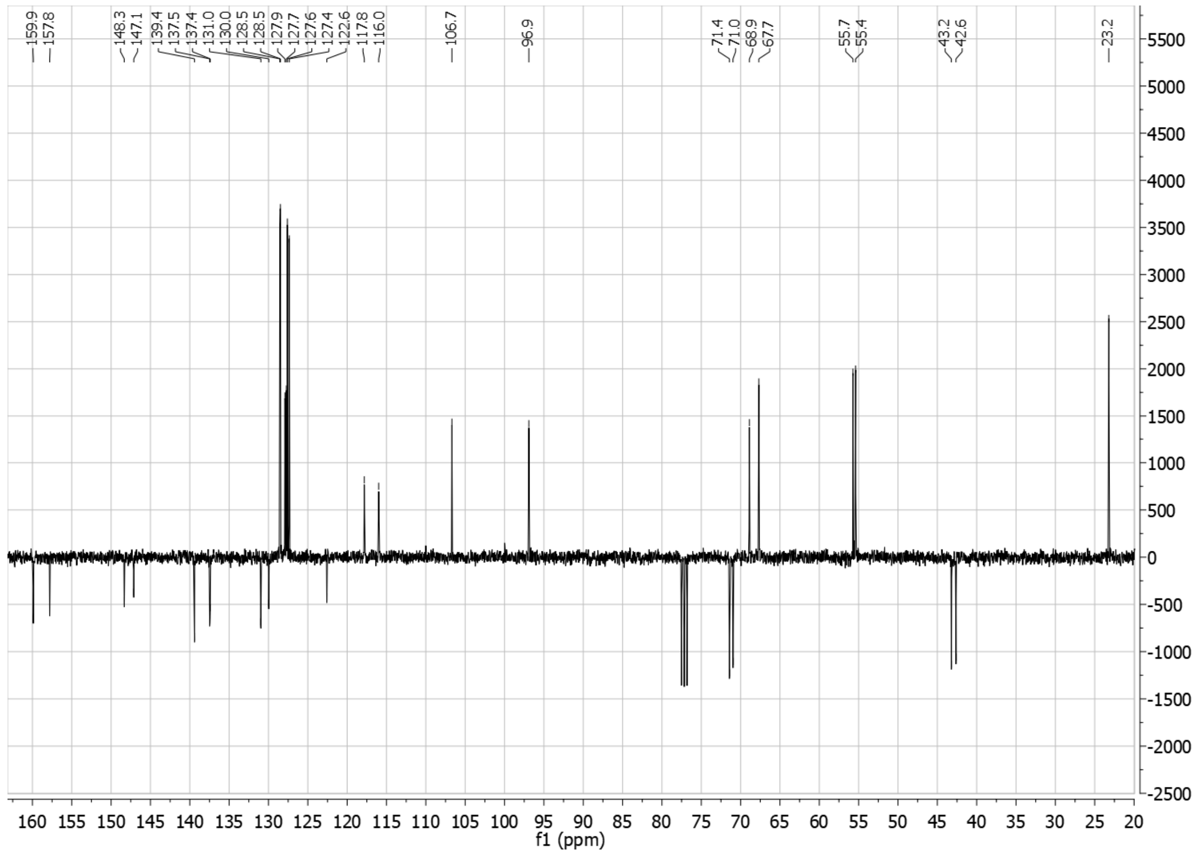

Figure S26. ^{13}C NMR (90 MHz) spectrum of (a*S*,2*S*,2'*S*)-**16** in $CDCl_3$.

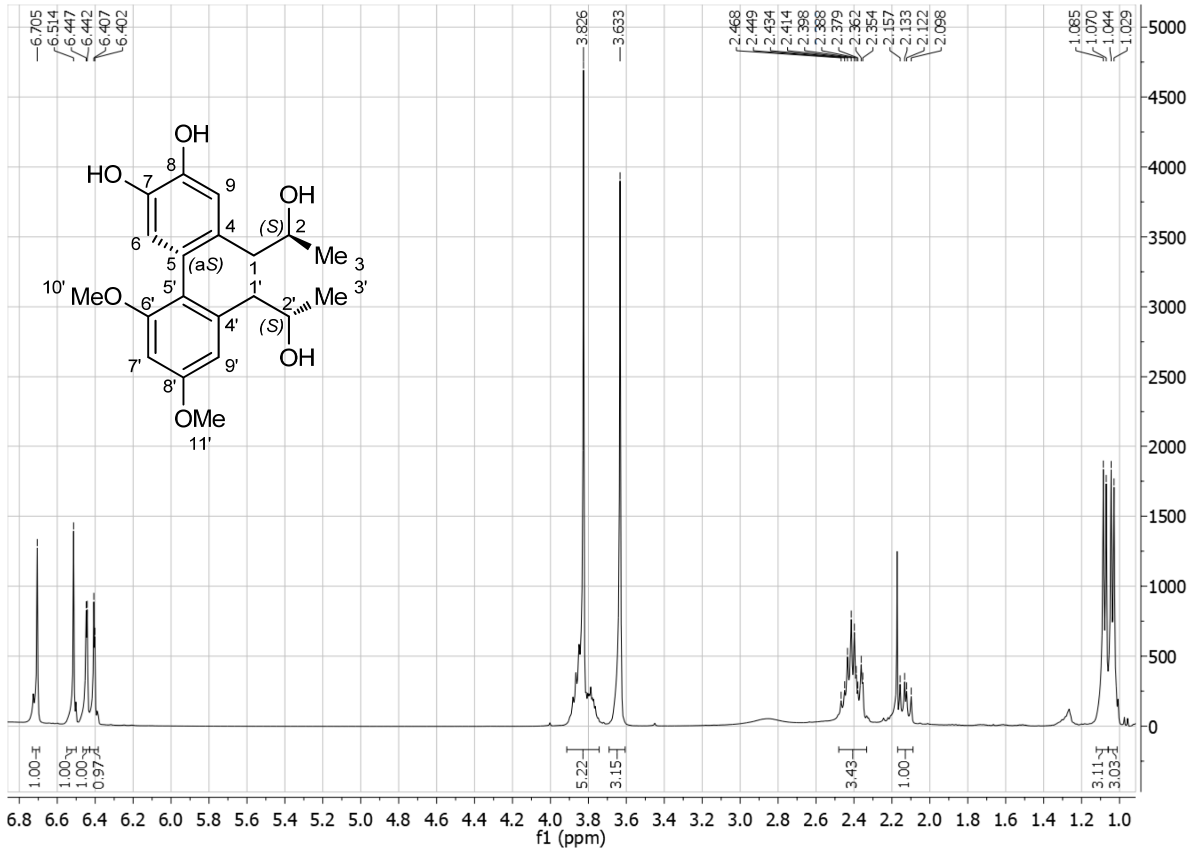

Figure S27. ^{1}H NMR (400 MHz) spectrum of (a*S*,2*S*,2'*S*)-**17** in $CDCl_3$.

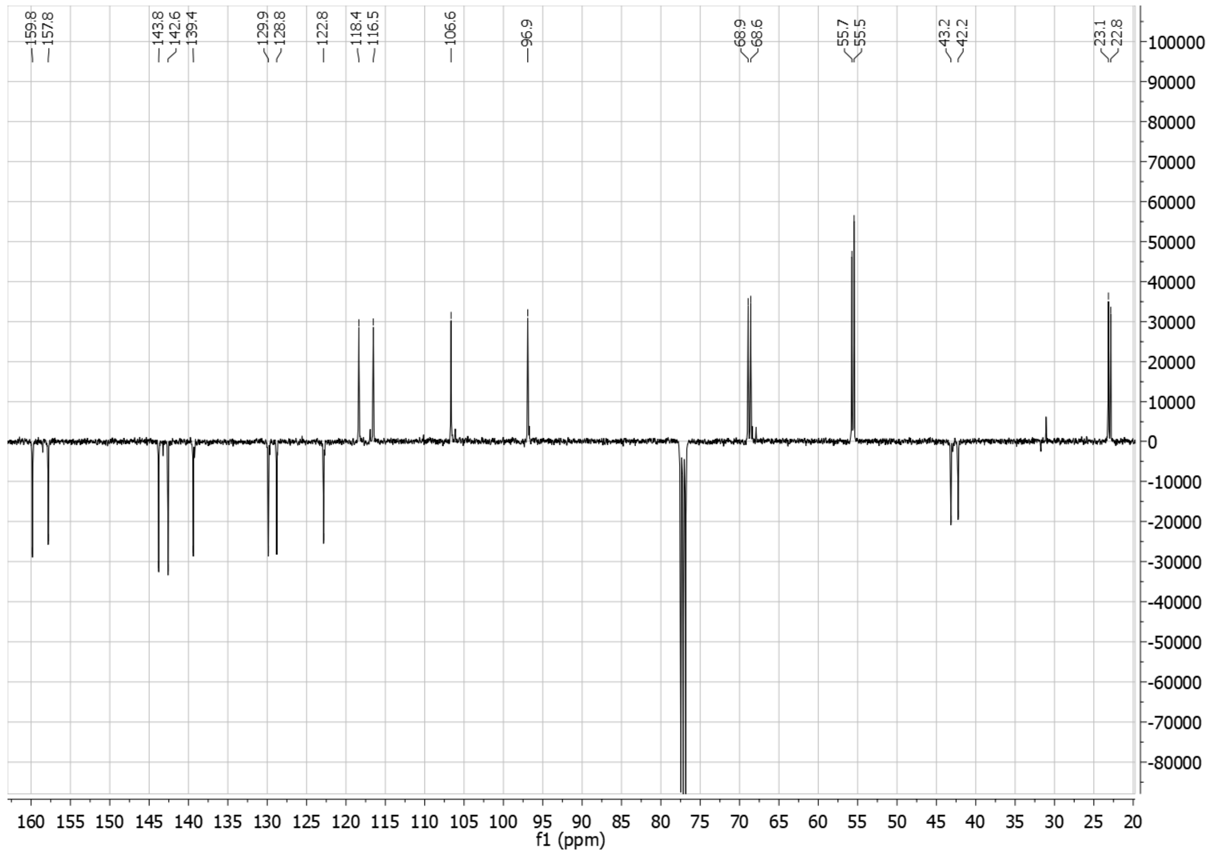

Figure S28. ^{13}C NMR (100 MHz) spectrum of (a*S*,2*S*,2'*S*)-**17** in $CDCl_3$.

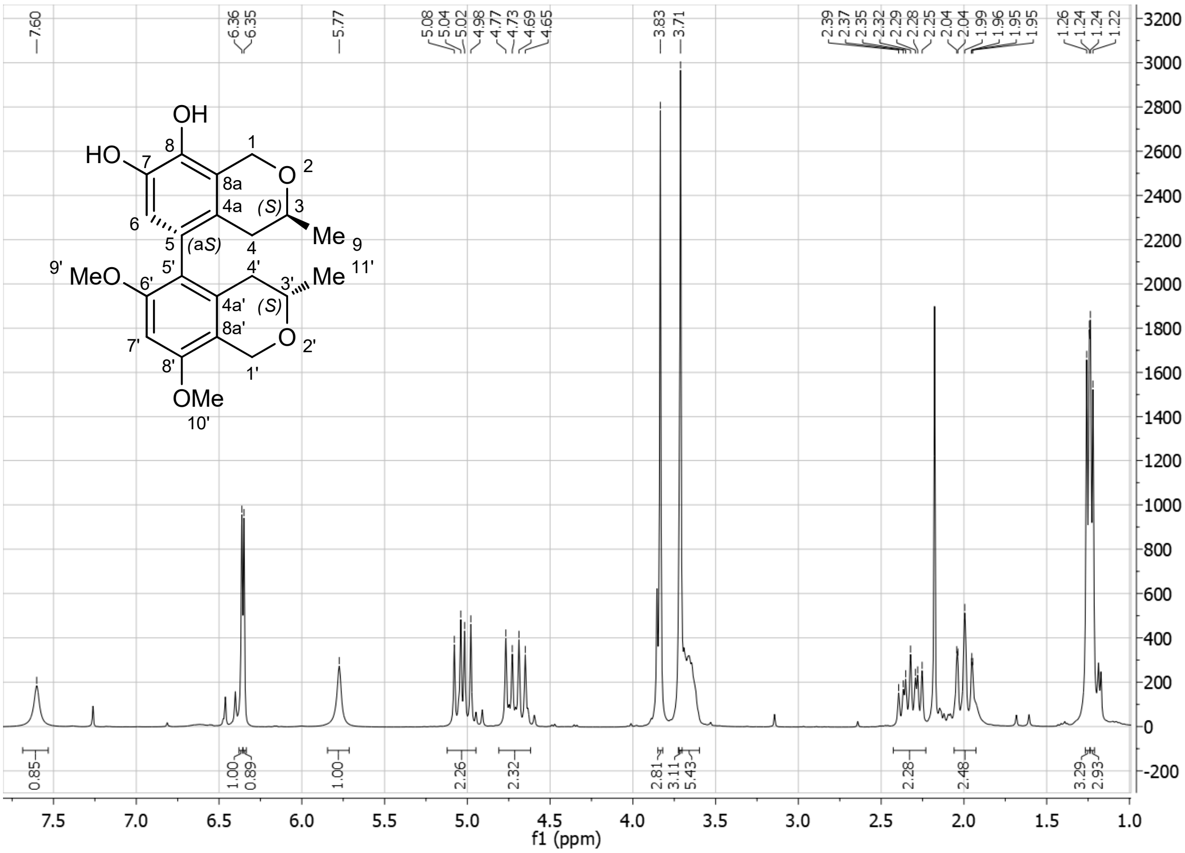

Figure S29. ^{1}H NMR (400 MHz) spectrum of (a*S*,3*S*,3’*S*)-**19** in $CDCl_3$.

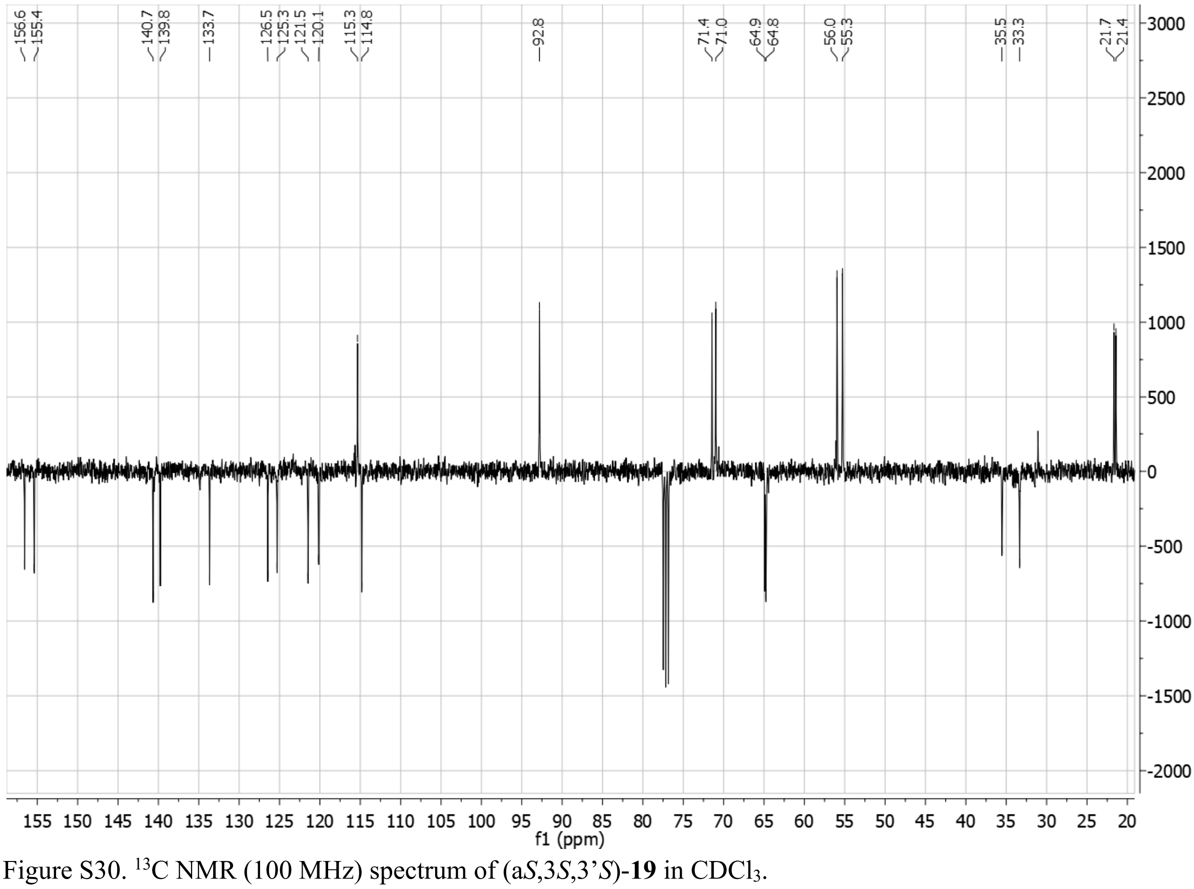

Figure S30. ^{13}C NMR (100 MHz) spectrum of (a*S*,3*S*,3’*S*)-**19** in $CDCl_3$.

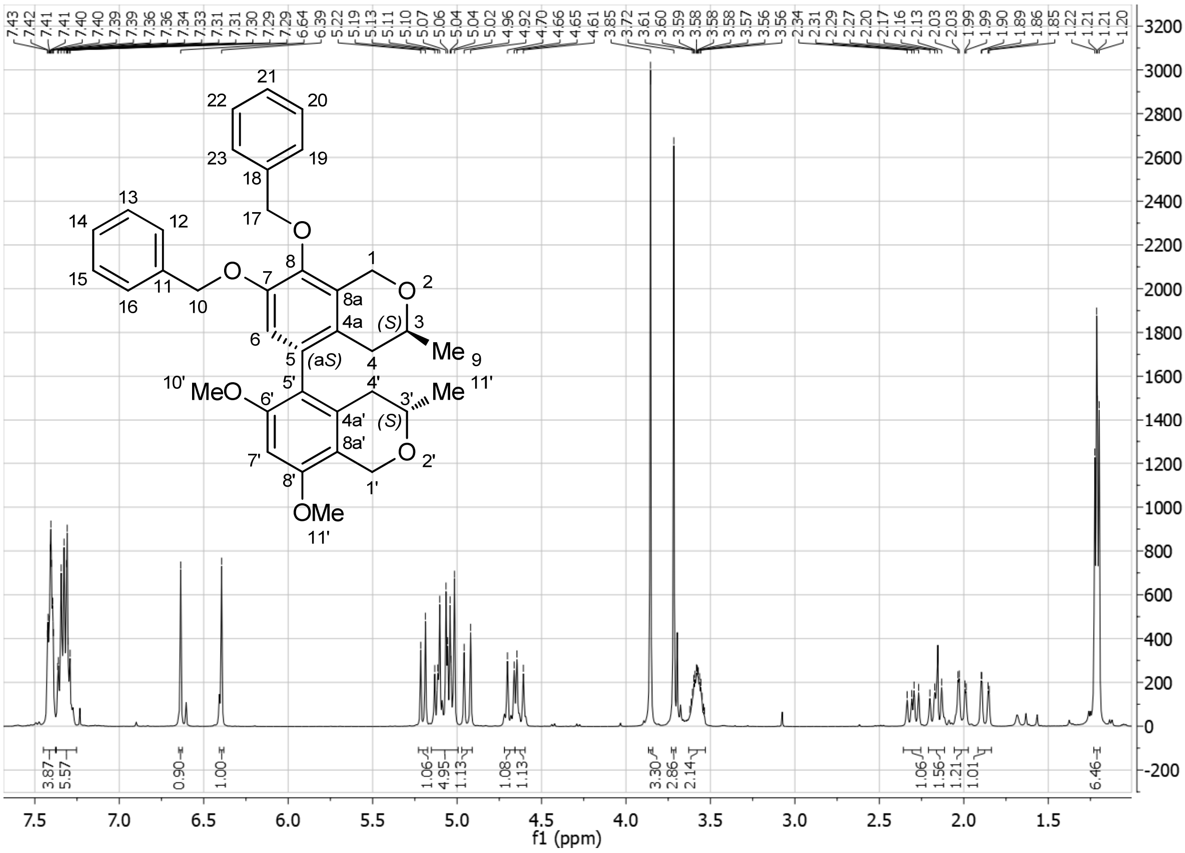

Figure S31. ^{1}H NMR (400 MHz) spectrum of (a*S*,3*S*,3’*S*)-**18** in CDCl$_3$.

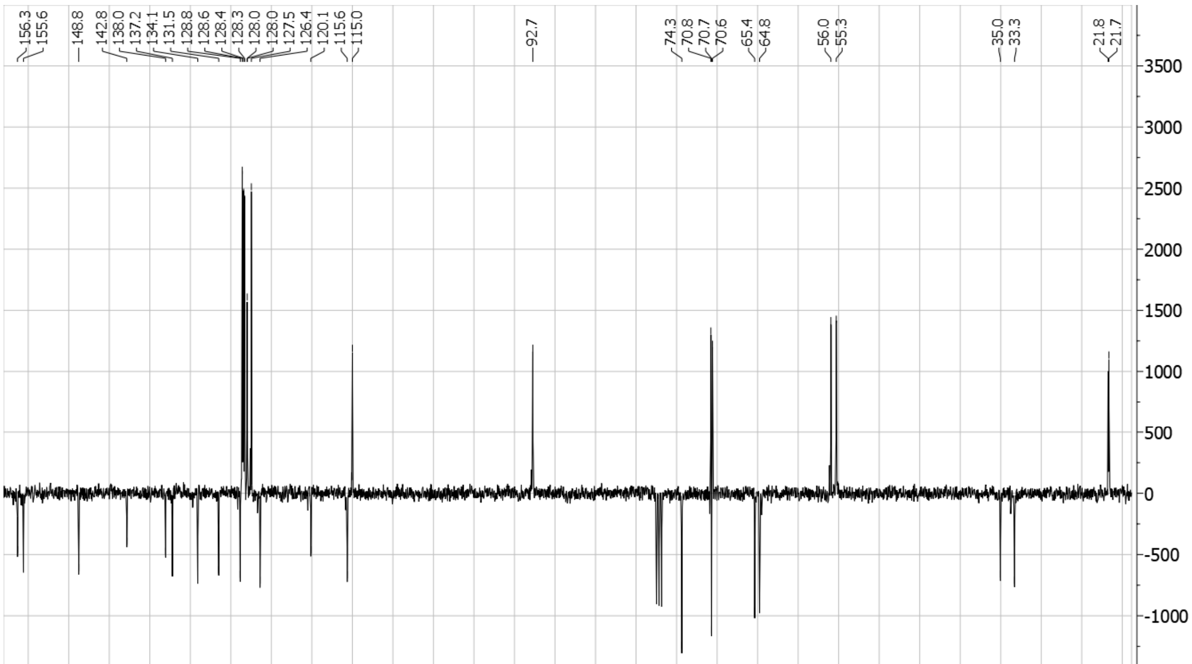

Figure S32. ^{13}C NMR (100 MHz) spectrum of (a*S*,3*S*,3’*S*)-**18** in CDCl$_3$.

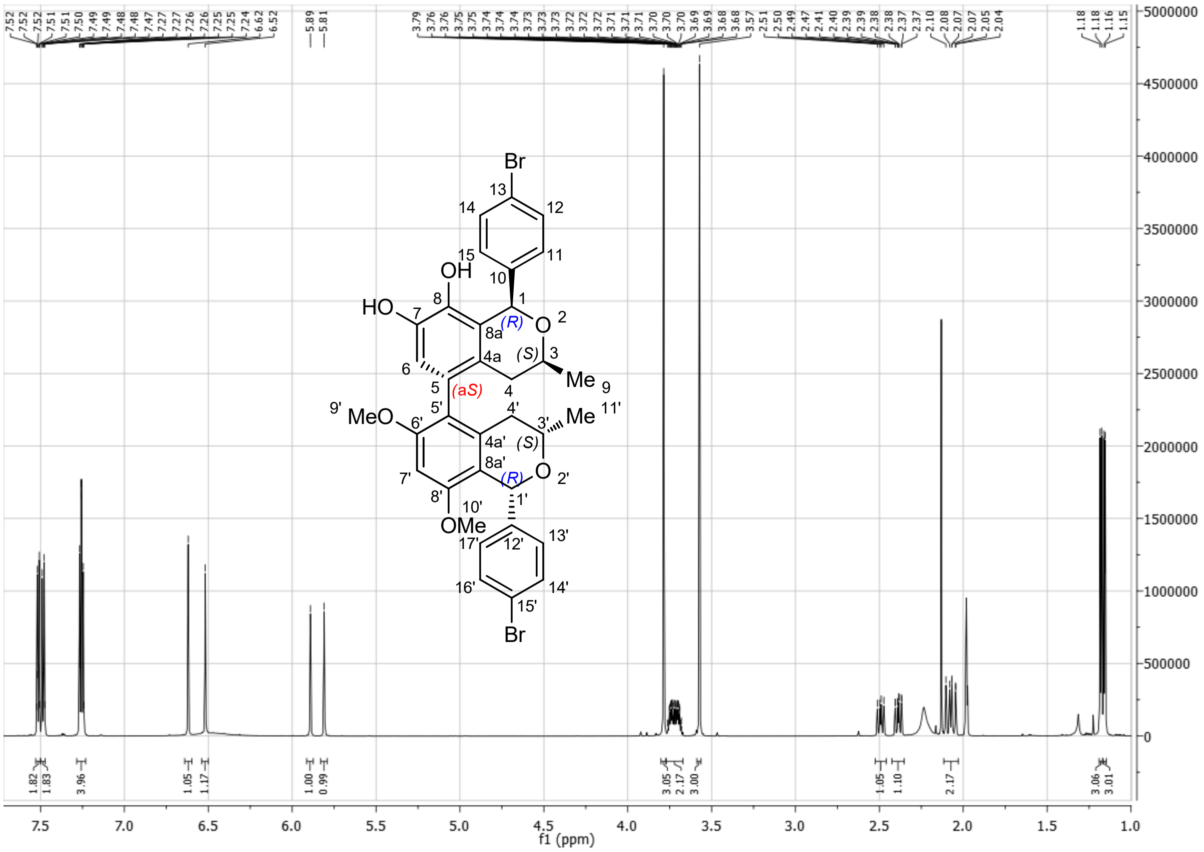

Figure S33. ^{1}H NMR (700 MHz) spectrum of *cis*,*cis*-(a*S*,1*R*,3*S*,1’*R*,3’*S*)-**21** in acetonitrile-d_3.

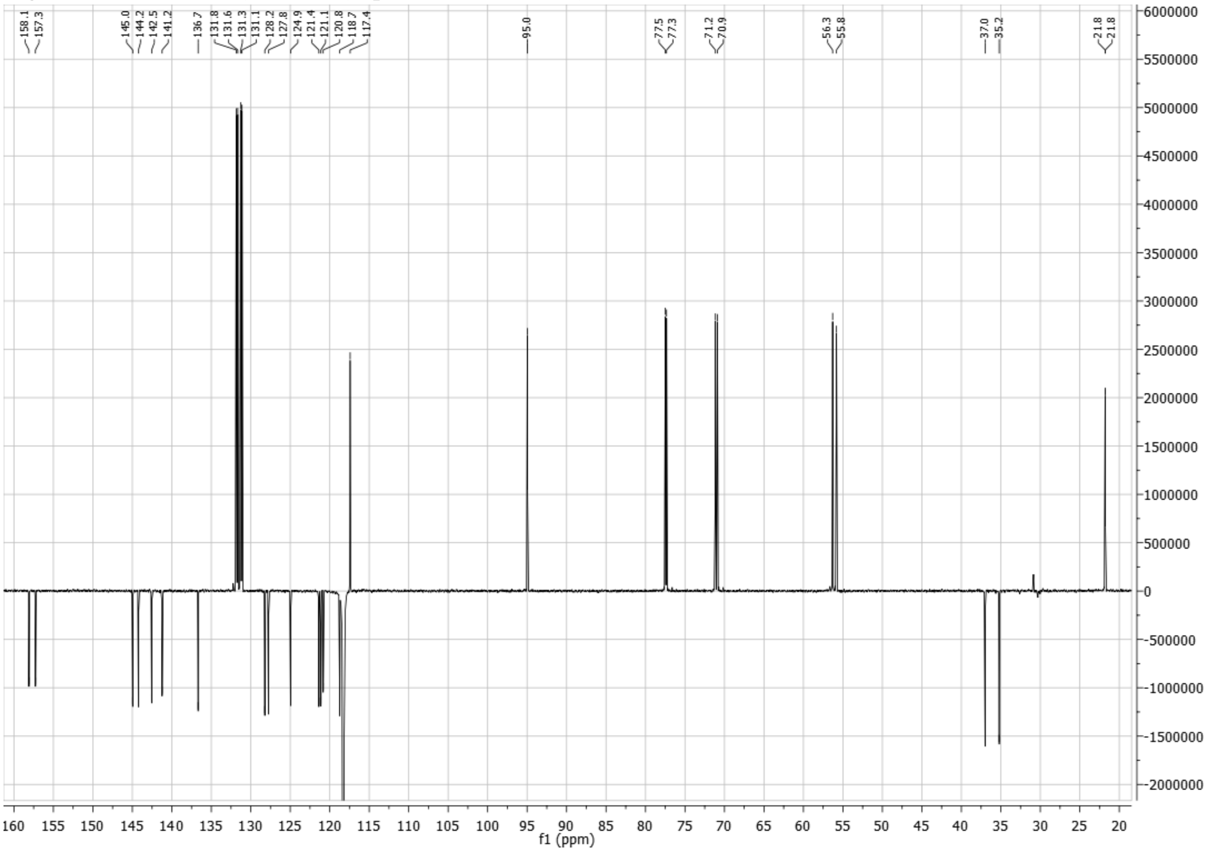

Figure S34. ^{13}C NMR (175 MHz) spectrum of *cis*,*cis*-(a*S*,1*R*,3*S*,1’*R*,3’*S*)-**21** in acetonitrile-d_3.

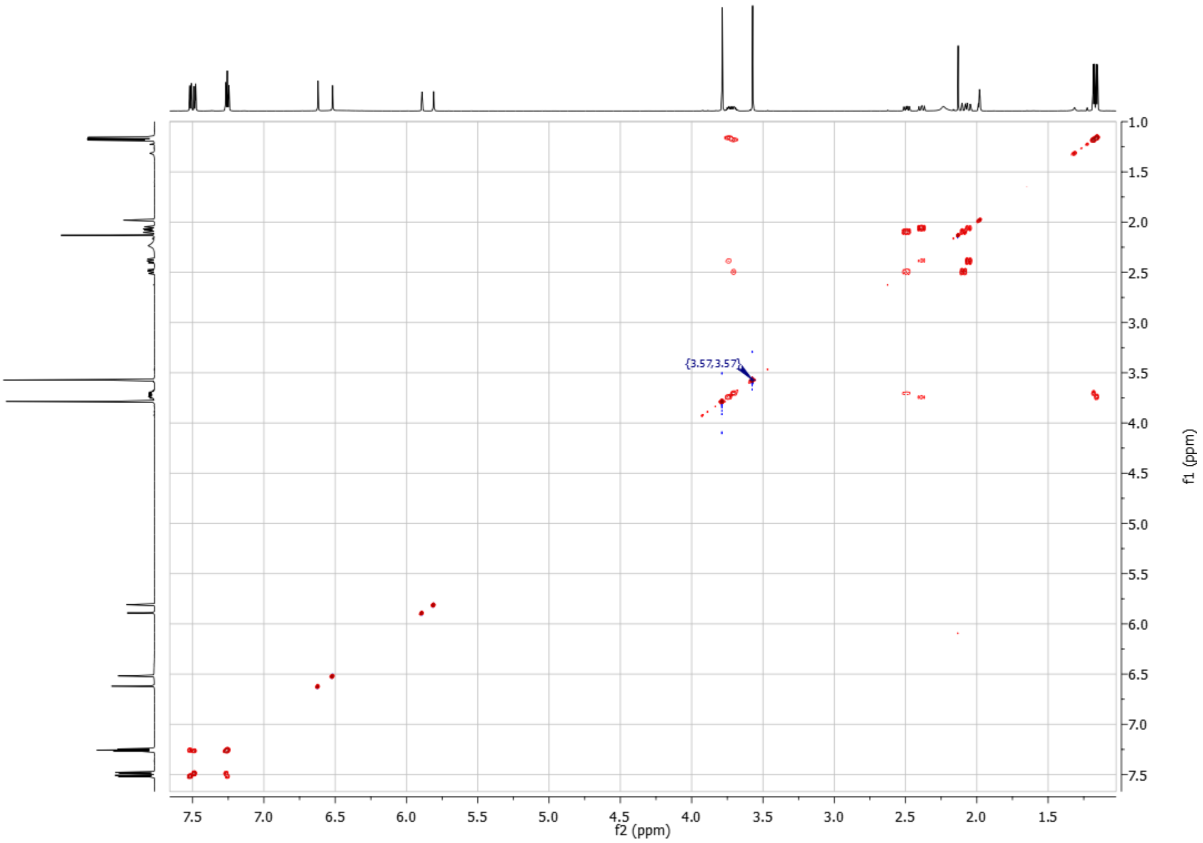

Figure S35. ^{1}H-^{1}H COSY NMR (700 MHz) spectrum of *cis*,*cis*-(a*S*,1*R*,3*S*,1'*R*,3'*S*)-**21** in acetonitrile-d_3.

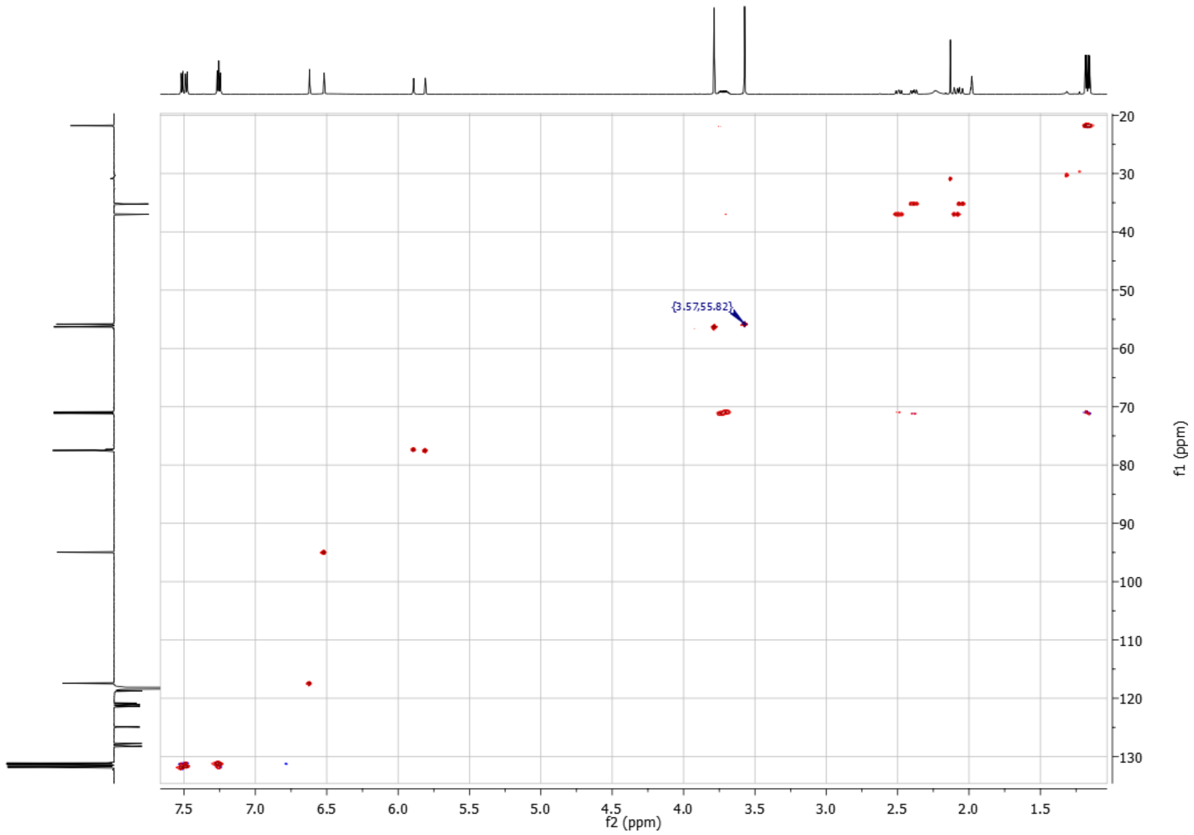

Figure S36. ^{1}H-^{13}C HSQC NMR (700 MHz) spectrum of *cis*,*cis*-(a*S*,1*R*,3*S*,1'*R*,3'*S*)-**21** in acetonitrile-d_3.

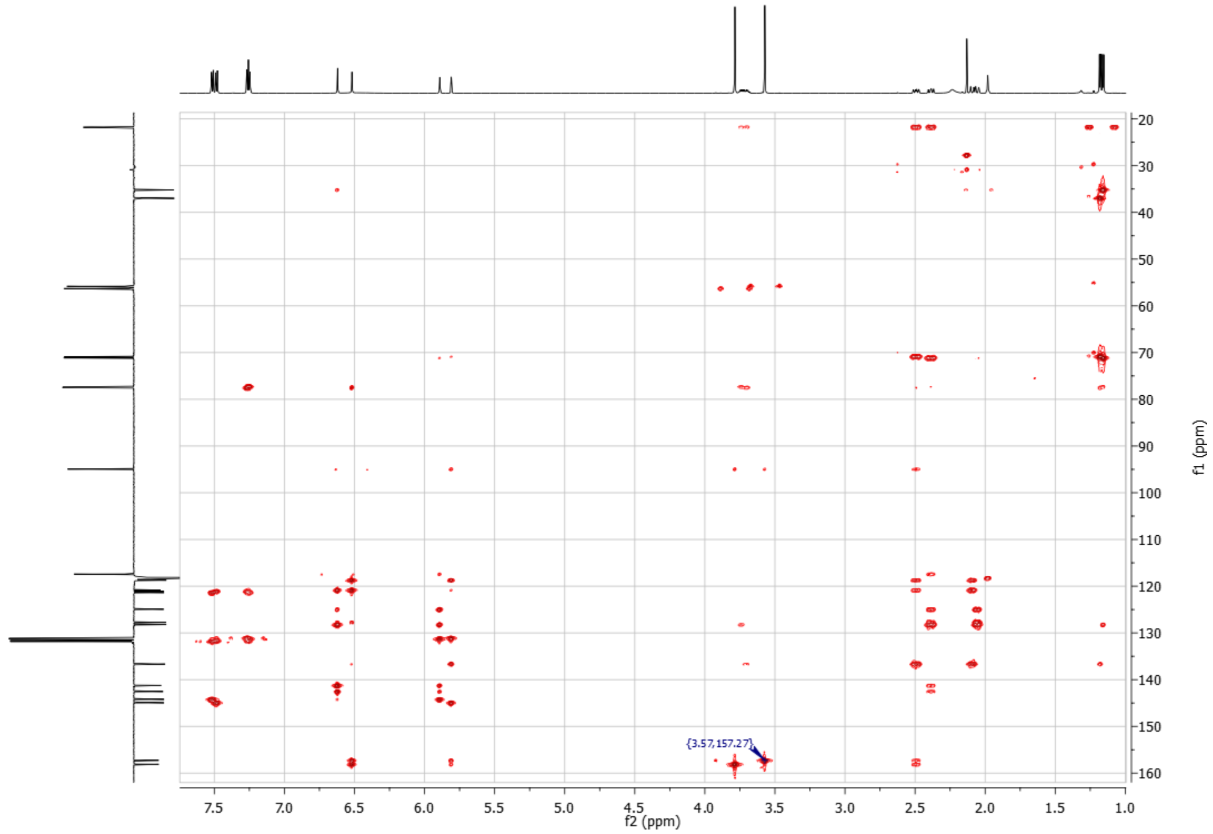

Figure S37. ^{1}H-^{13}C HMBC NMR (700 MHz) spectrum of *cis*,*cis*-(a*S*,1*R*,3*S*,1'*R*,3'*S*)-**21** in acetonitrile-d_3.

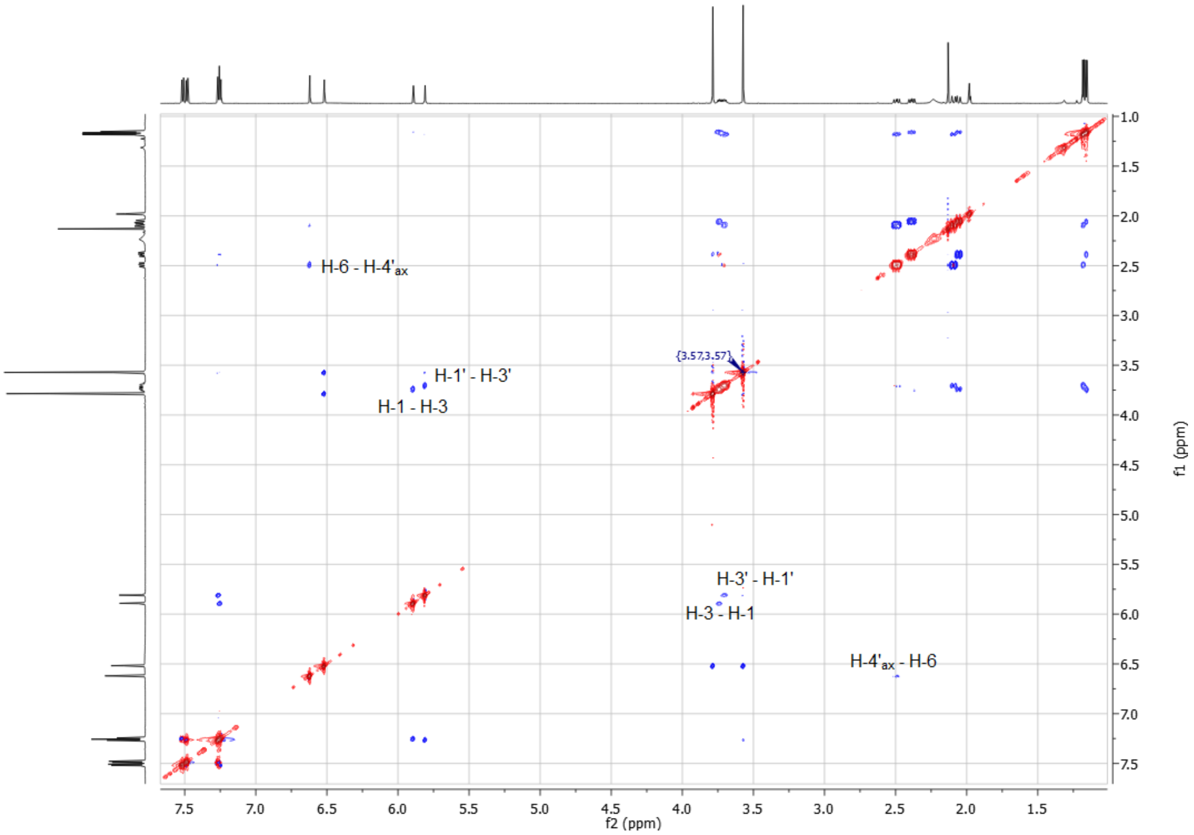

Figure S38. ^{1}H-^{1}H ROESY NMR (700 MHz) spectrum of *cis*,*cis*-(a*S*,1*R*,3*S*,1'*R*,3'*S*)-**21** in acetonitrile-d_3.

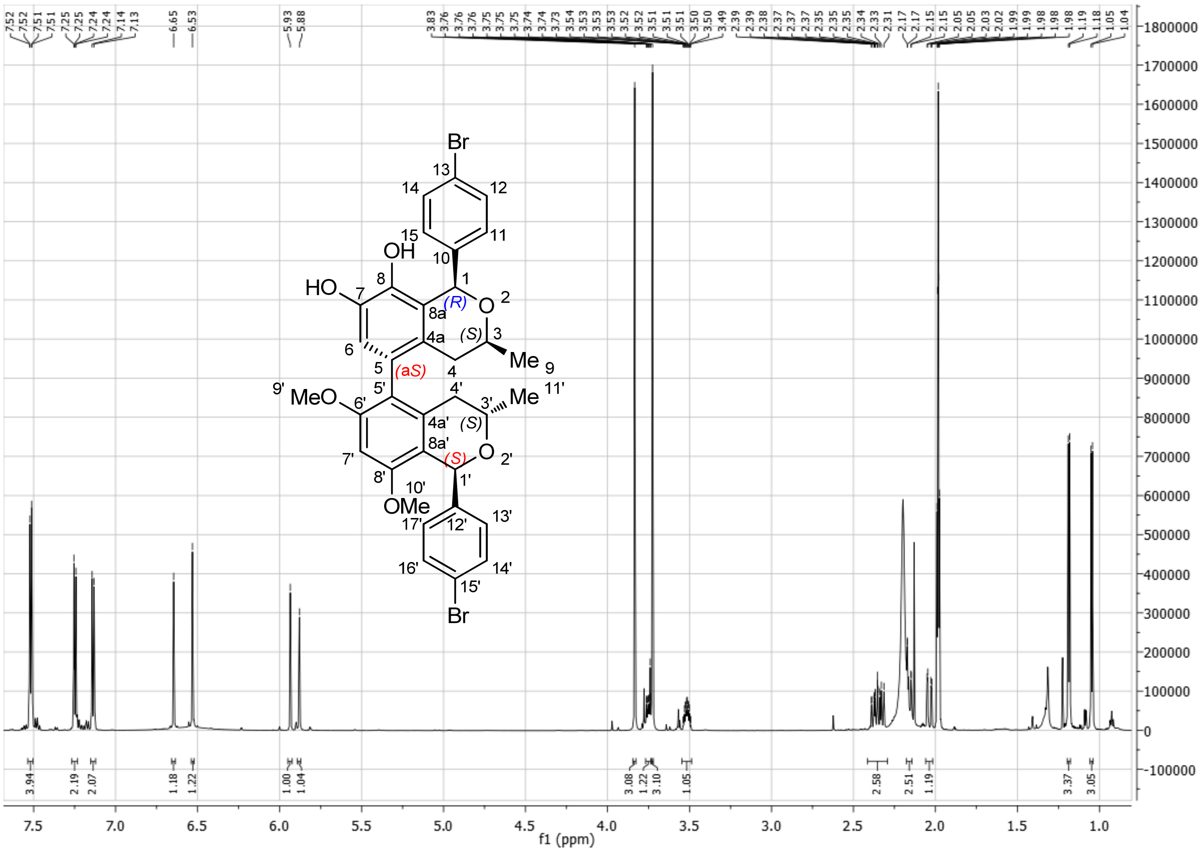

Figure S39. ^{1}H NMR (700 MHz) spectrum of *cis*,*trans*-(a*S*,1*R*,3*S*,1'*S*,3'*S*)-**21** in acetonitrile-d_3.

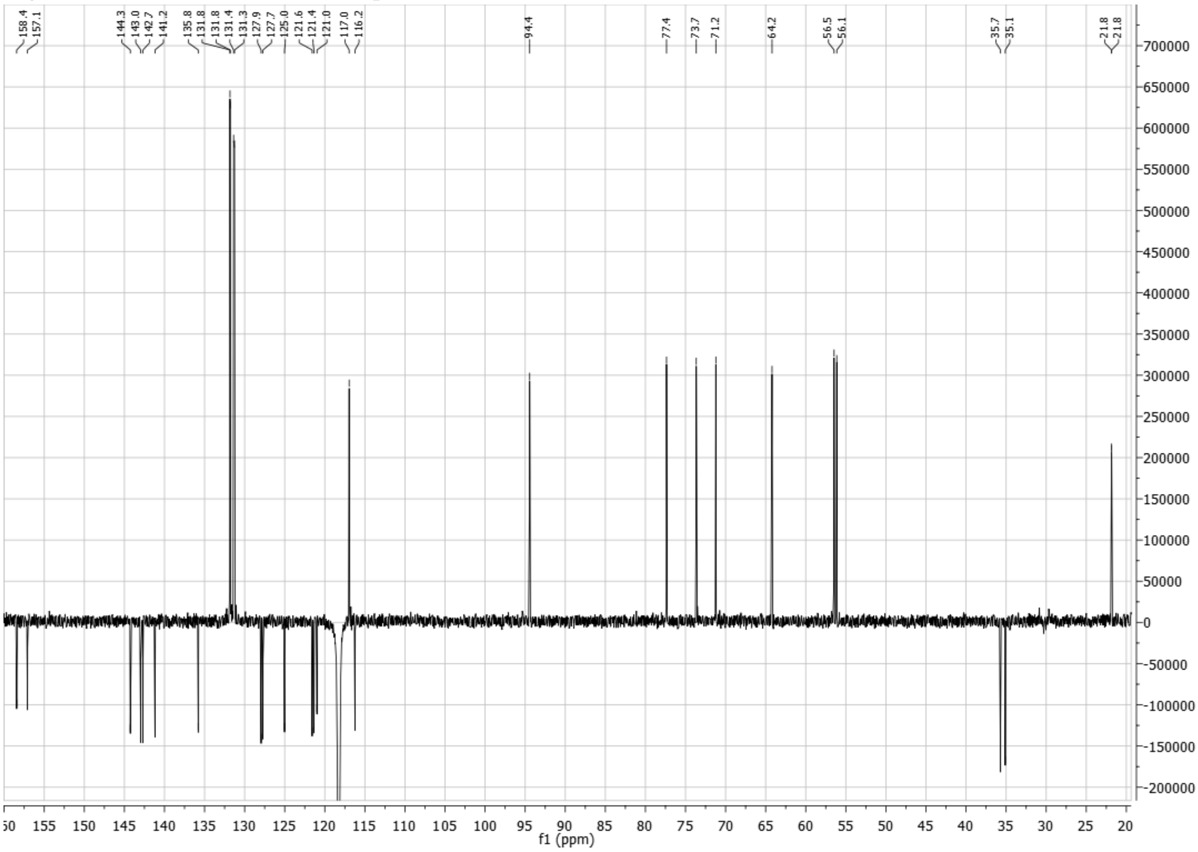

Figure S40. ^{13}C NMR (175 MHz) spectrum of *cis*,*trans*-(a*S*,1*R*,3*S*,1'*S*,3'*S*)-**21** in acetonitrile-d_3.

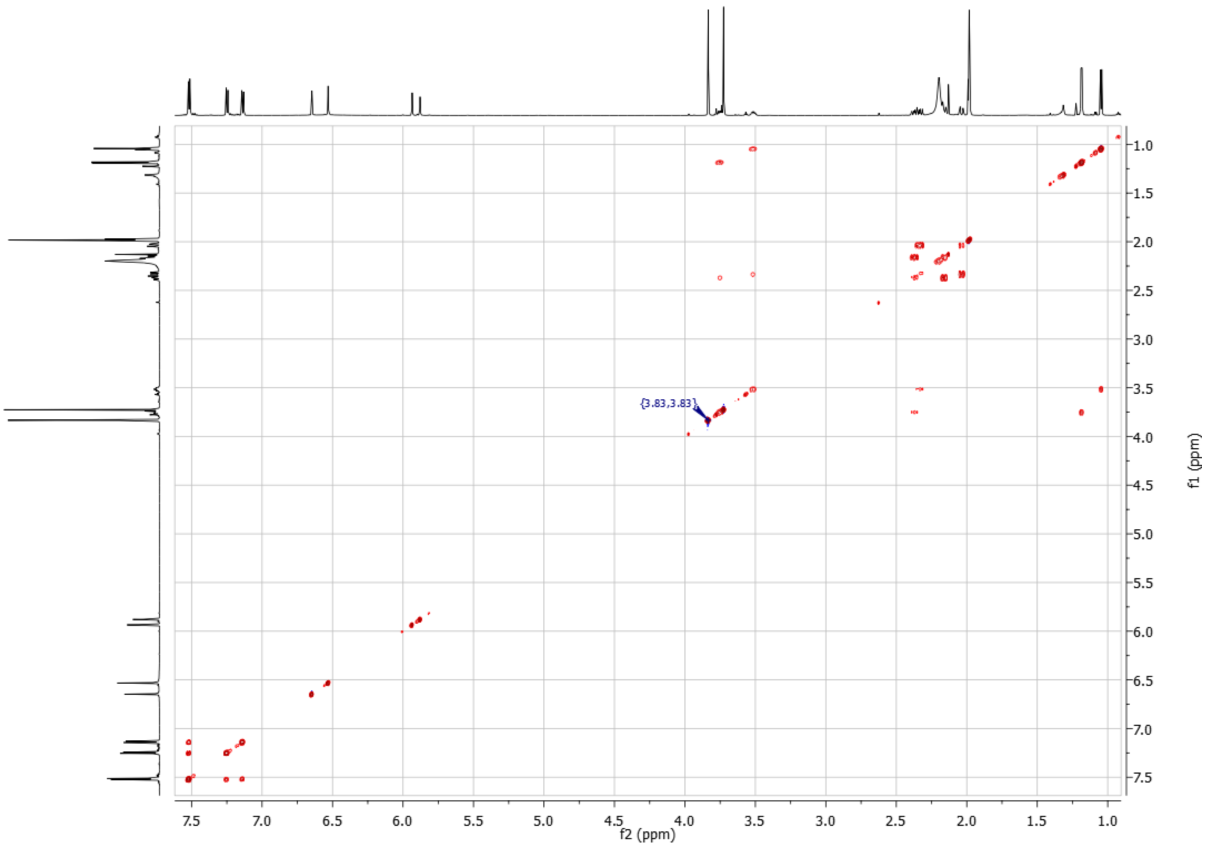

Figure S41. ^{1}H-^{1}H COSY NMR (700 MHz) spectrum of *cis*,*trans*-(a*S*,1*R*,3*S*,1'*S*,3'*S*)-**21** in acetonitrile-d_3.

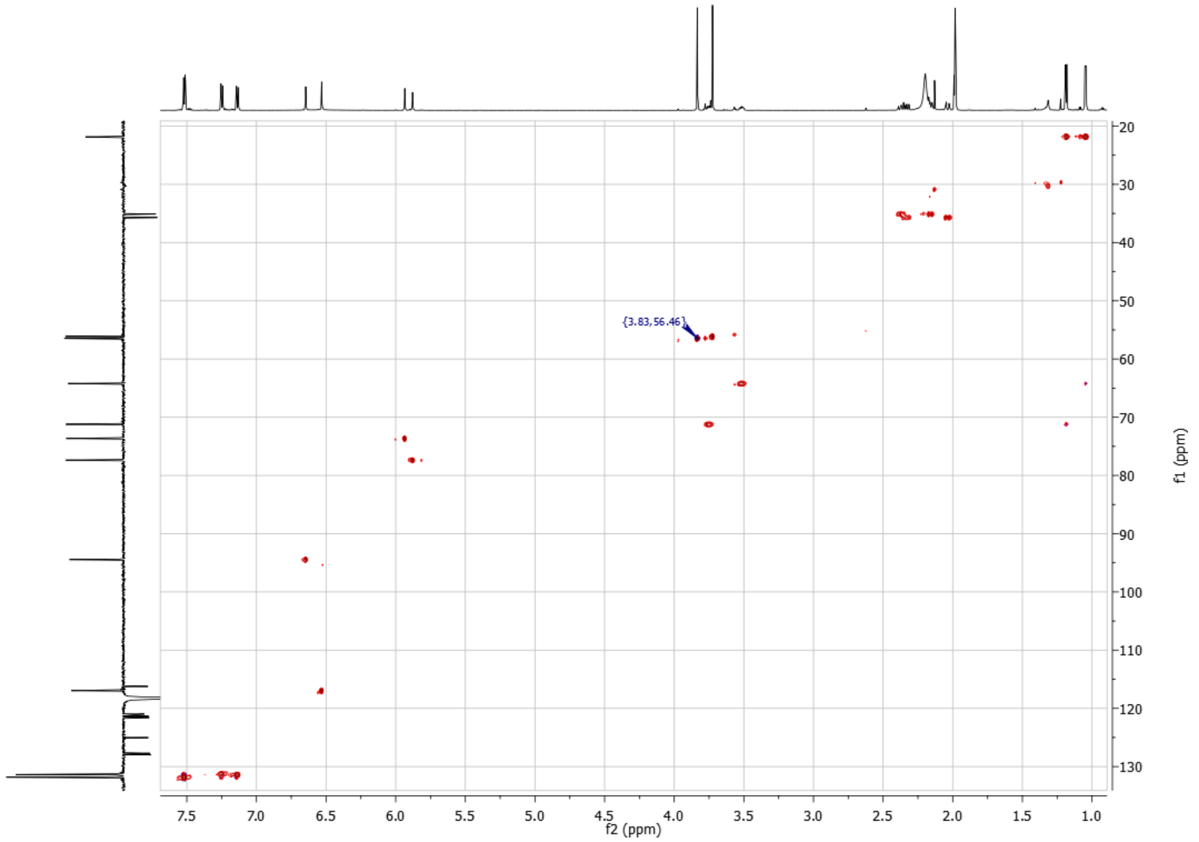

Figure S42. ^{1}H-^{13}C HSQC NMR (700 MHz) spectrum of *cis*,*trans*-(a*S*,1*R*,3*S*,1'*S*,3'*S*)-**21** in acetonitrile-d_3.

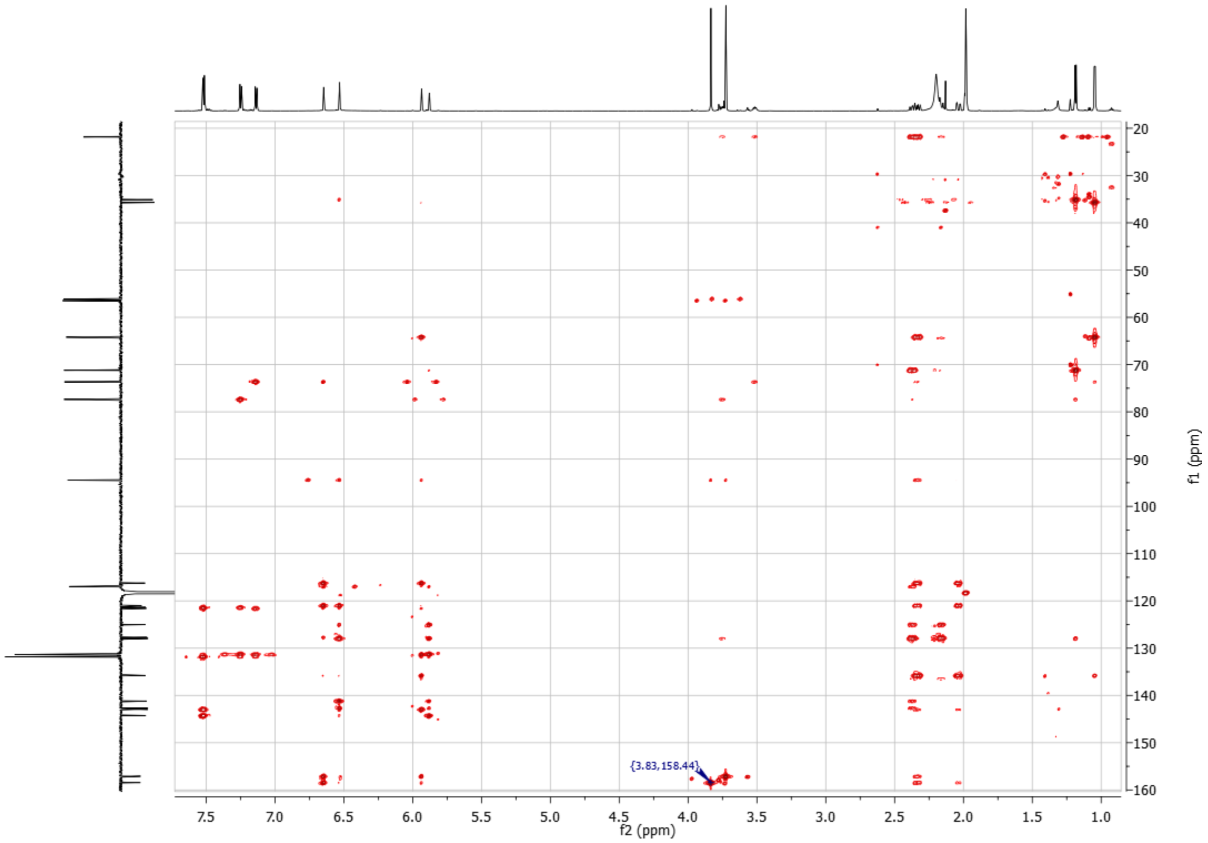

Figure S43. ^{1}H-^{13}C HMBC NMR (700 MHz) spectrum of *cis*,*trans*-(a*S*,1*R*,3*S*,1'*S*,3'*S*)-**21** in acetonitrile-d_3.

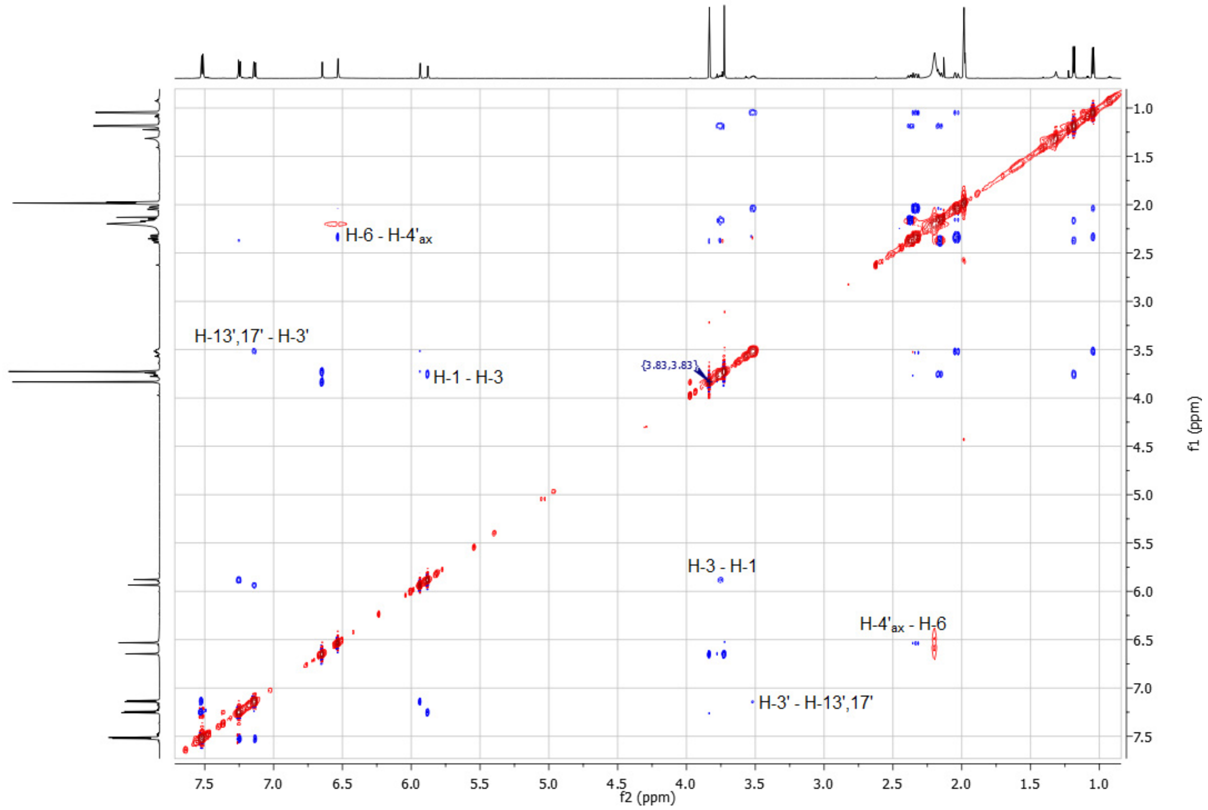

Figure S44. ^{1}H-^{1}H ROESY NMR (700 MHz) spectrum of *cis*,*trans*-(a*S*,1*R*,3*S*,1'*S*,3'*S*)-**21** in acetonitrile-d_3.

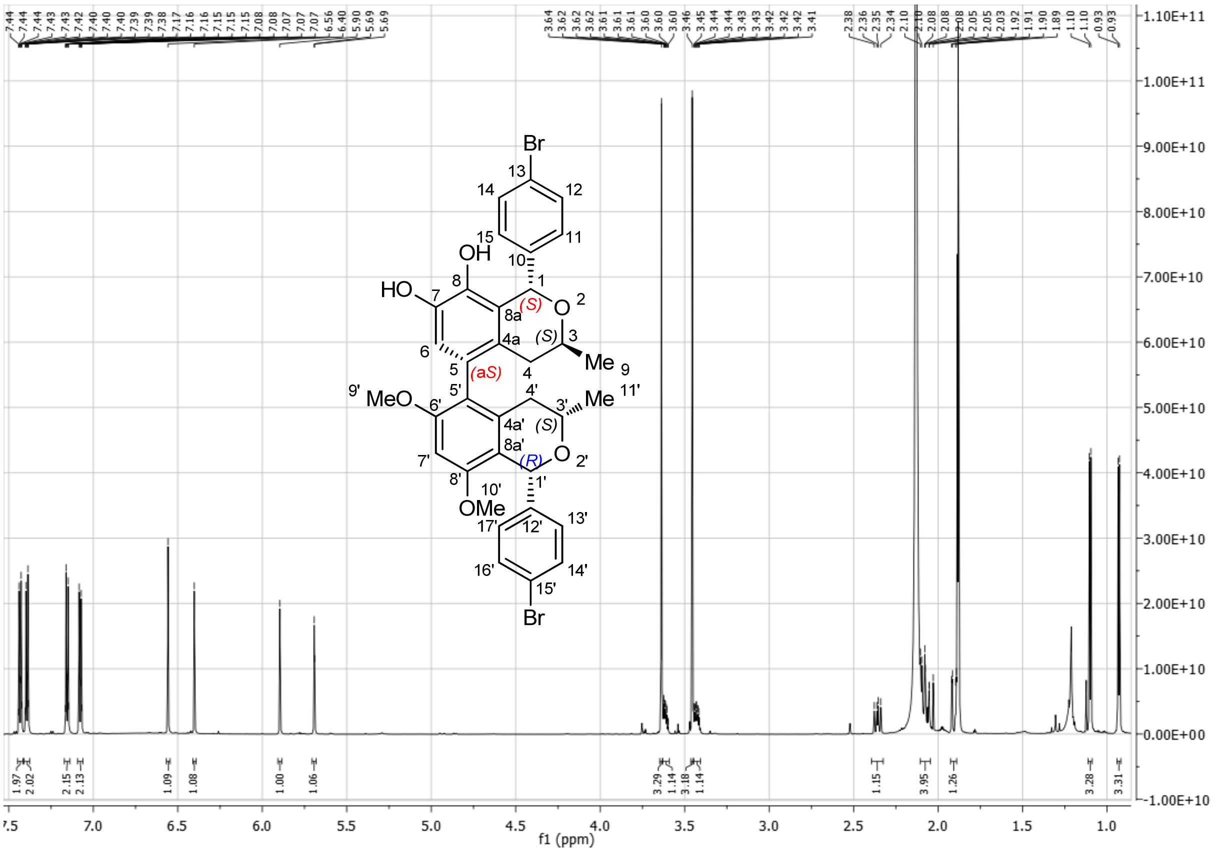

Figure S45. ^{1}H NMR (700 MHz) spectrum of *trans*,*cis*-(a*S*,1*S*,3*S*,1'*R*,3'*S*)-**21** in acetonitrile-d_3.

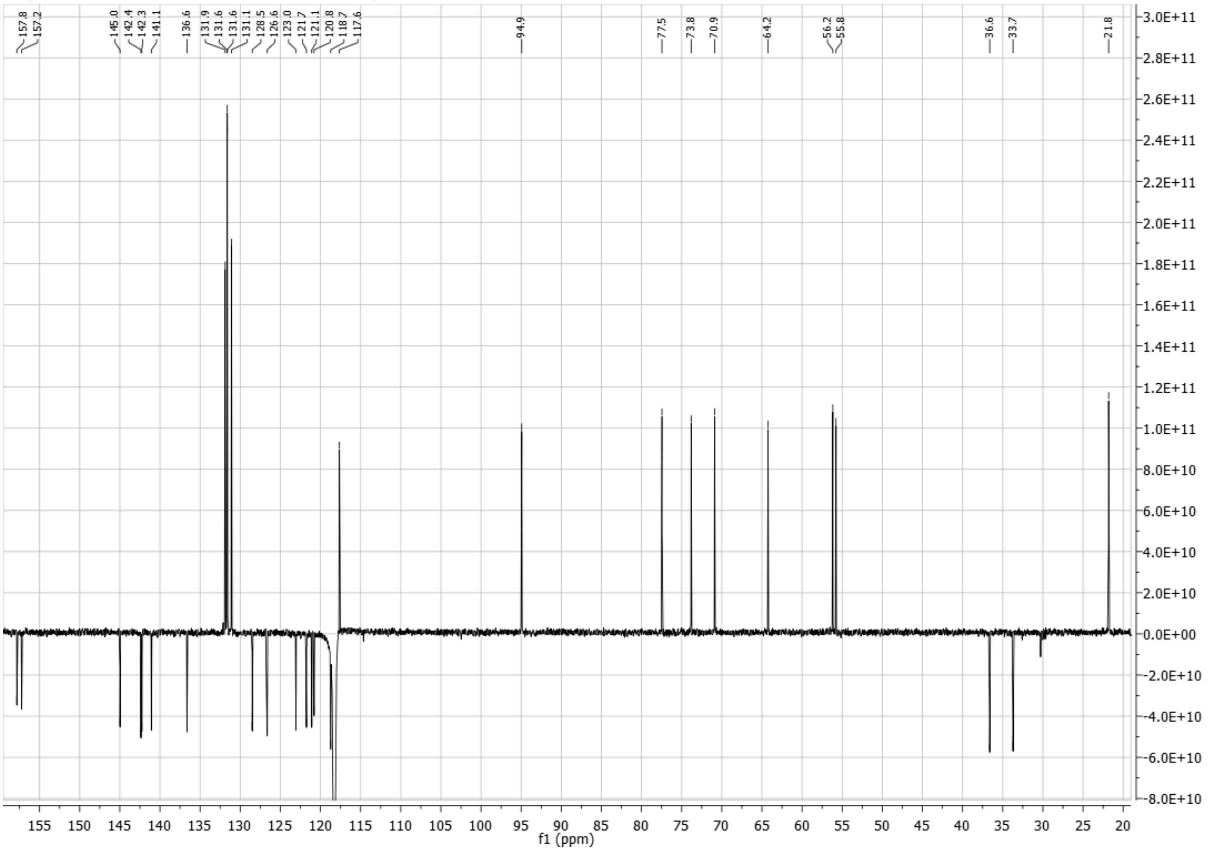

Figure S46. ^{13}C NMR (175 MHz) spectrum of *trans*,*cis*-(a*S*,1*S*,3*S*,1'*R*,3'*S*)-**21** in acetonitrile-d_3.

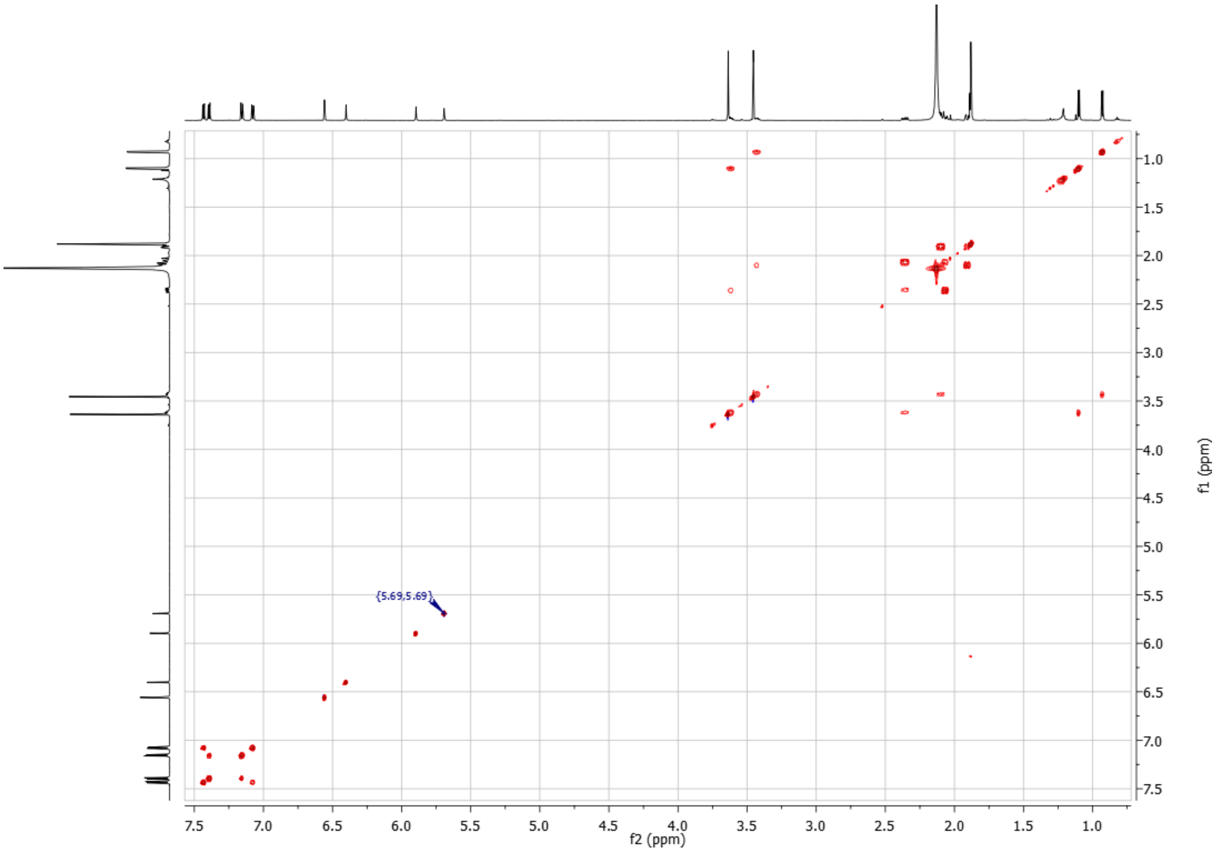

Figure S47. ^{1}H-^{1}H COSY NMR (700 MHz) spectrum of *trans*,*cis*-(a*S*,1*S*,3*S*,1'*R*,3'*S*)-**21** in acetonitrile-d_3.

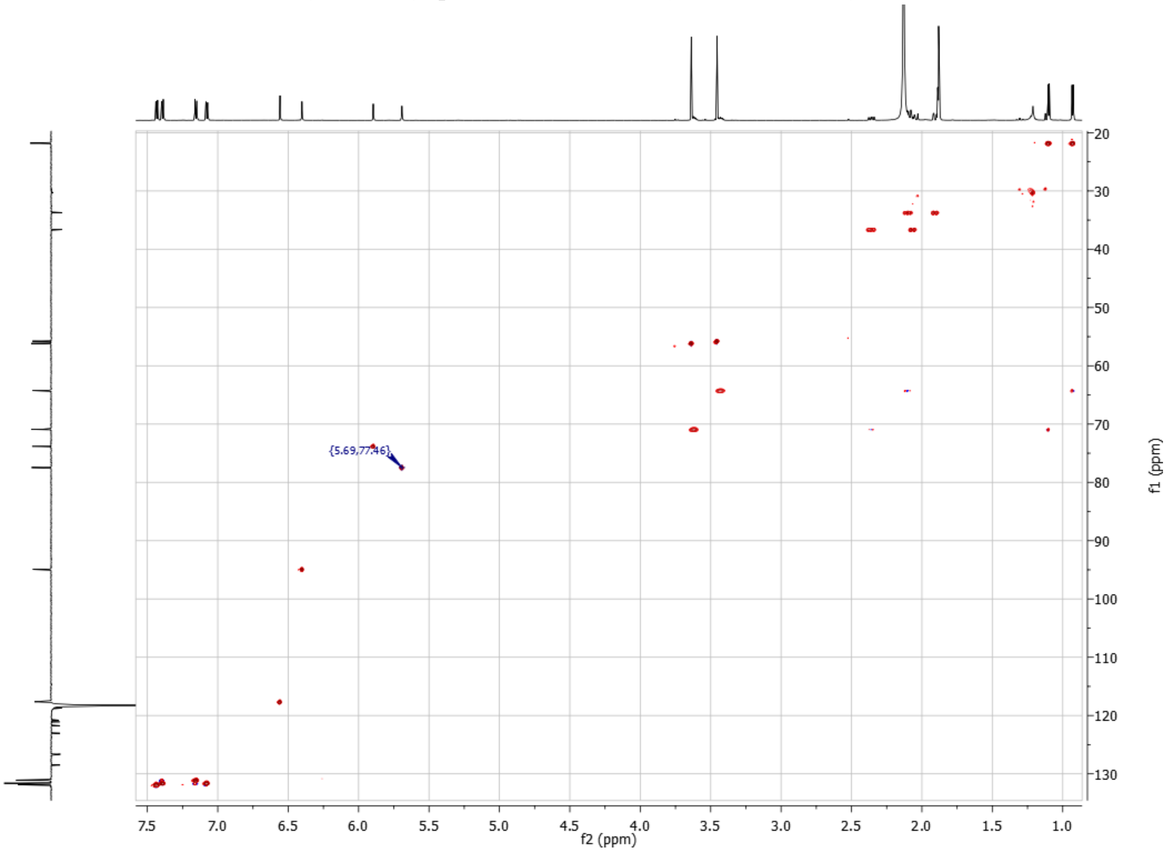

Figure S48. ^{1}H-^{13}C HSQC NMR (700 MHz) spectrum of *trans*,*cis*-(a*S*,1*S*,3*S*,1'*R*,3'*S*)-**21** in acetonitrile-d_3.

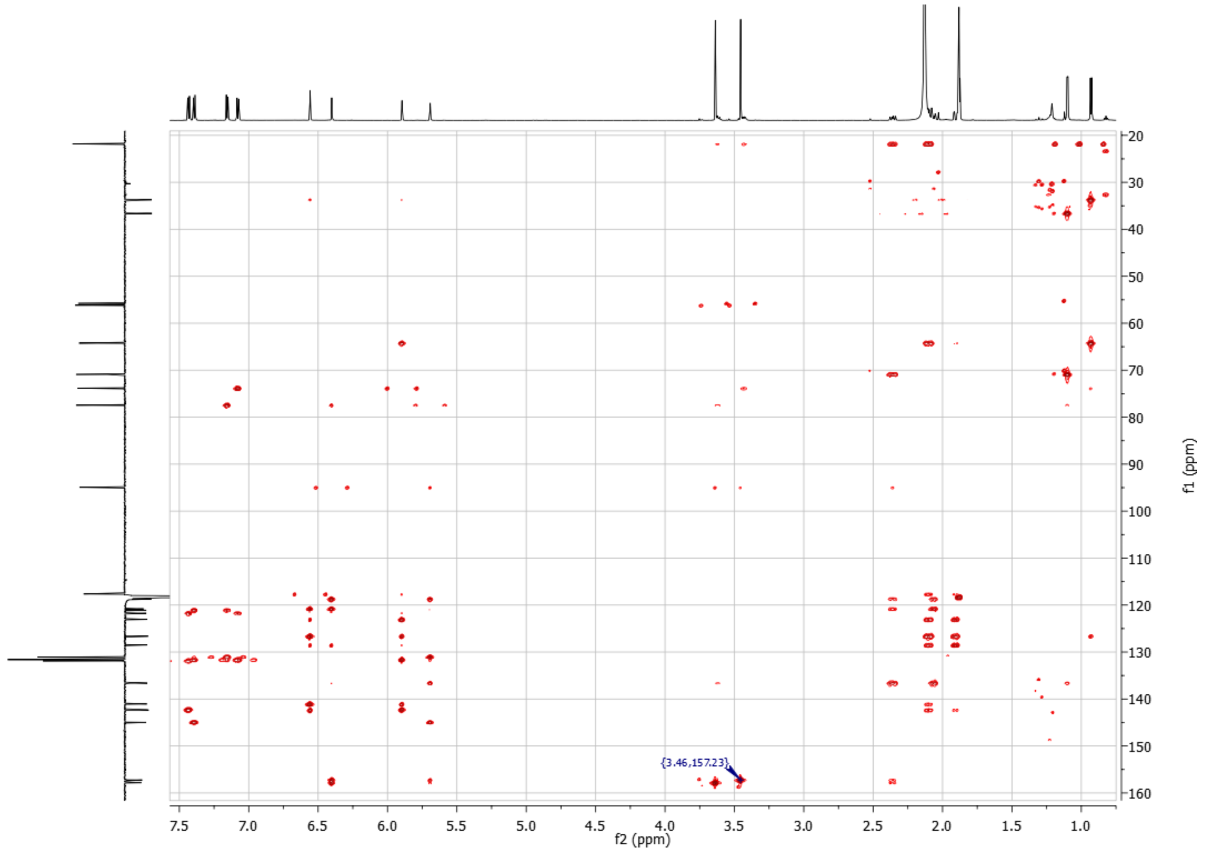

Figure S49. ^{1}H-^{13}C HMBC NMR (700 MHz) spectrum of *trans*,*cis*-(a*S*,1*S*,3*S*,1'*R*,3'*S*)-**21** in acetonitrile-d_3.

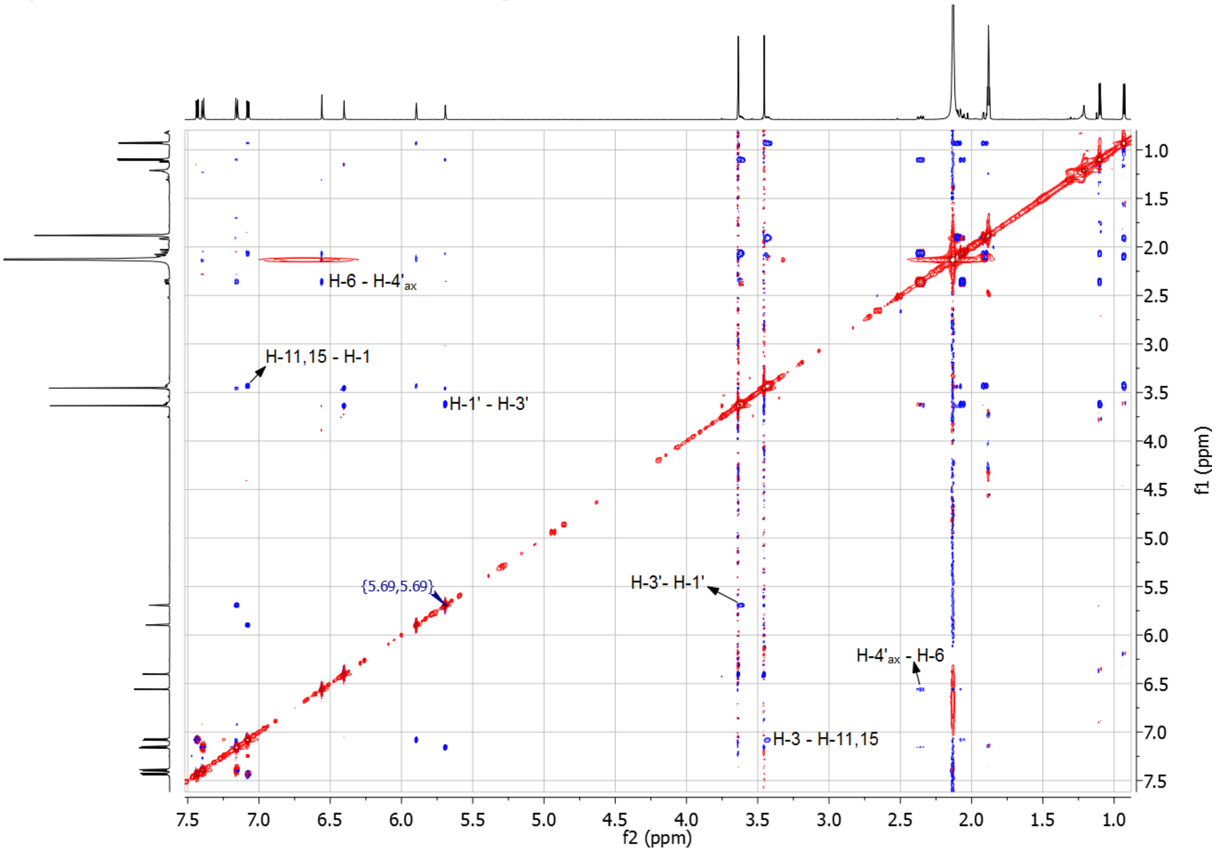

Figure S50. ^{1}H-^{1}H ROESY NMR (700 MHz) spectrum of *trans*,*cis*-(a*S*,1*S*,3*S*,1'*R*,3'*S*)-**21** in acetonitrile-d_3.

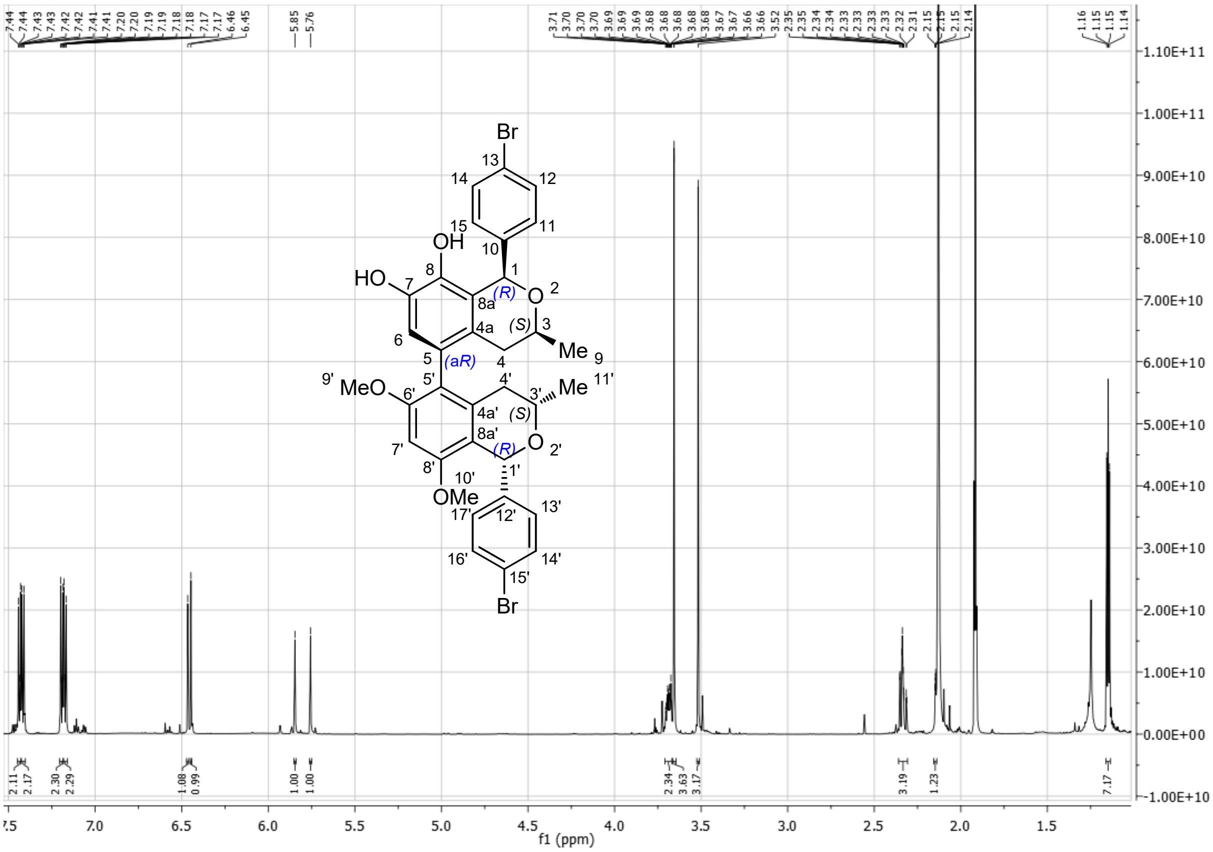

Figure S51. ^{1}H NMR (700 MHz) spectrum of *cis*,*cis*-(a*R*,1*R*,3*S*,1'*R*,3'*S*)-**21** in acetonitrile-d_3.

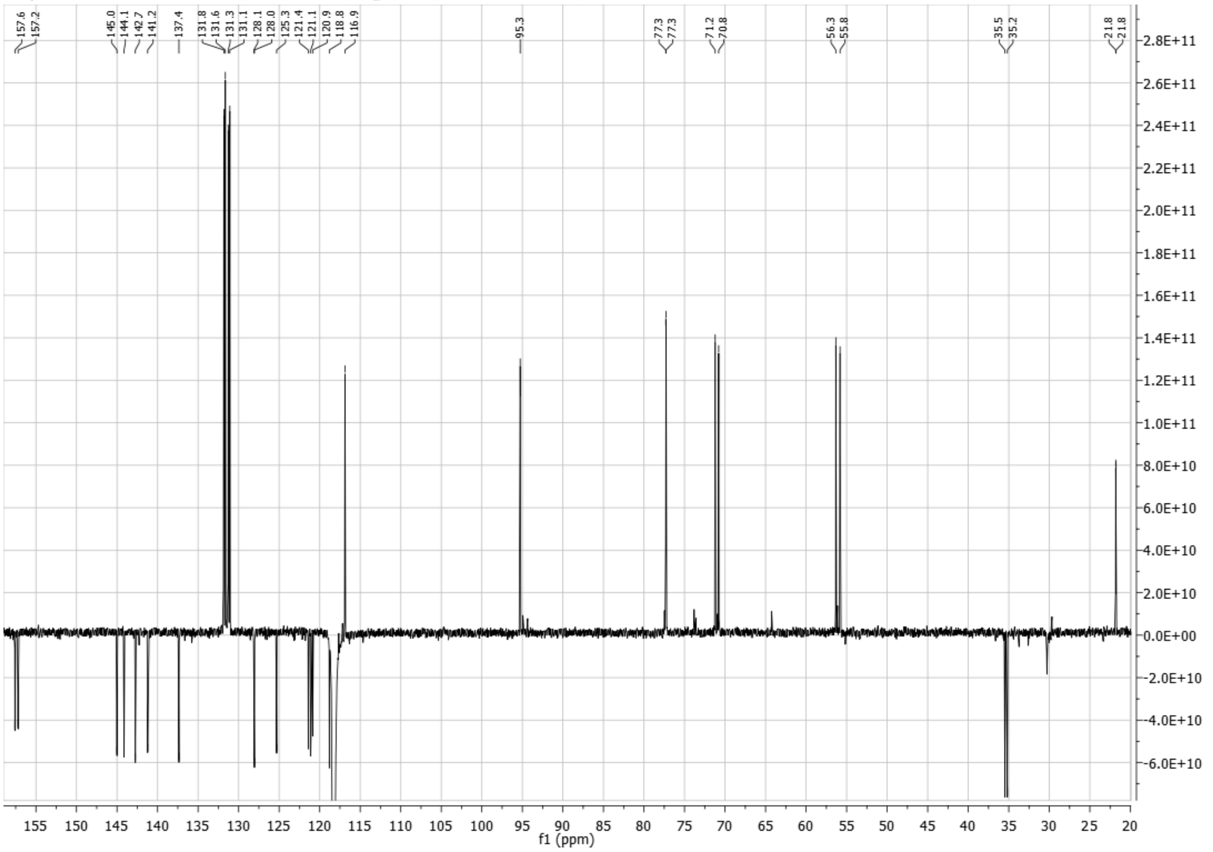

Figure S52. ^{13}C NMR (175 MHz) spectrum of *cis*,*cis*-(a*R*,1*R*,3*S*,1'*R*,3'*S*)-**21** in acetonitrile-d_3.

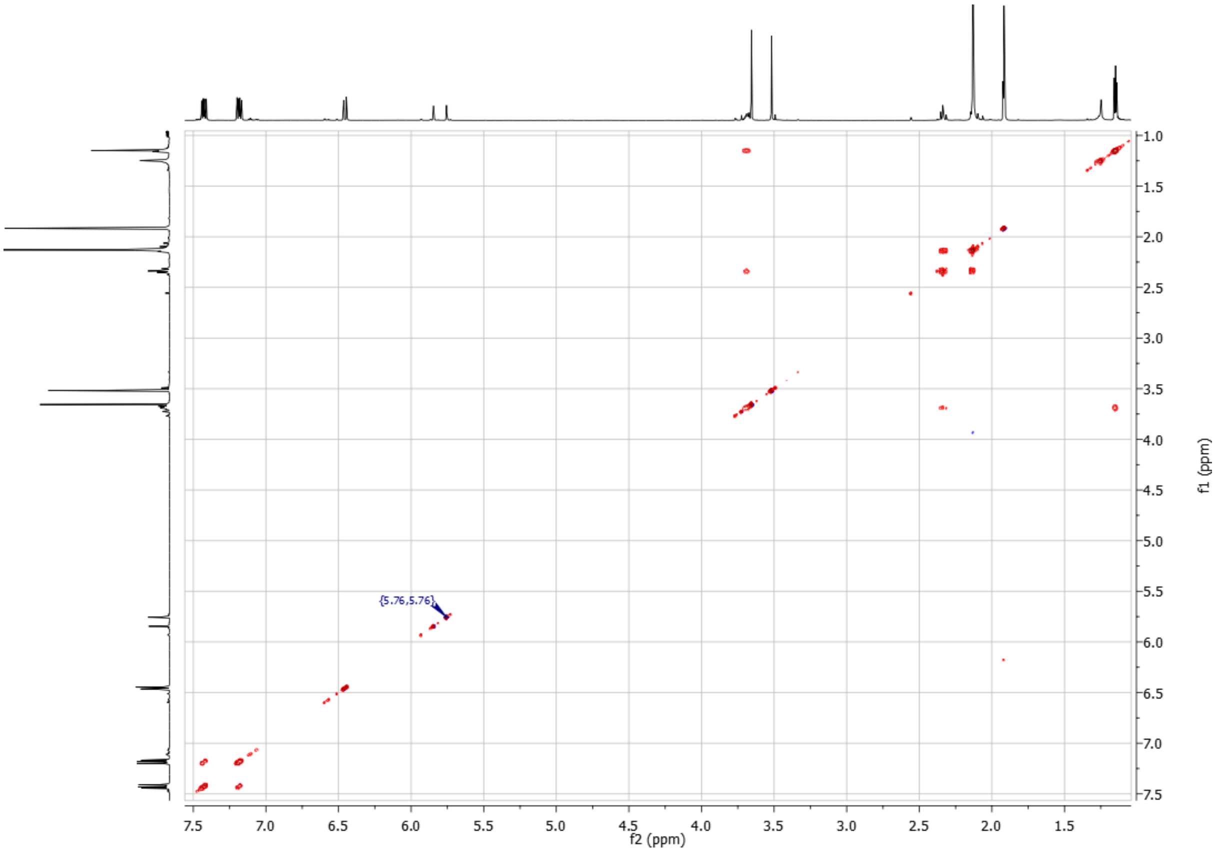

Figure S53. ^{1}H-^{1}H COSY NMR (700 MHz) spectrum of *cis*,*cis*-(a*R*,1*R*,3*S*,1’*R*,3’*S*)-**21** in acetonitrile-d_3.

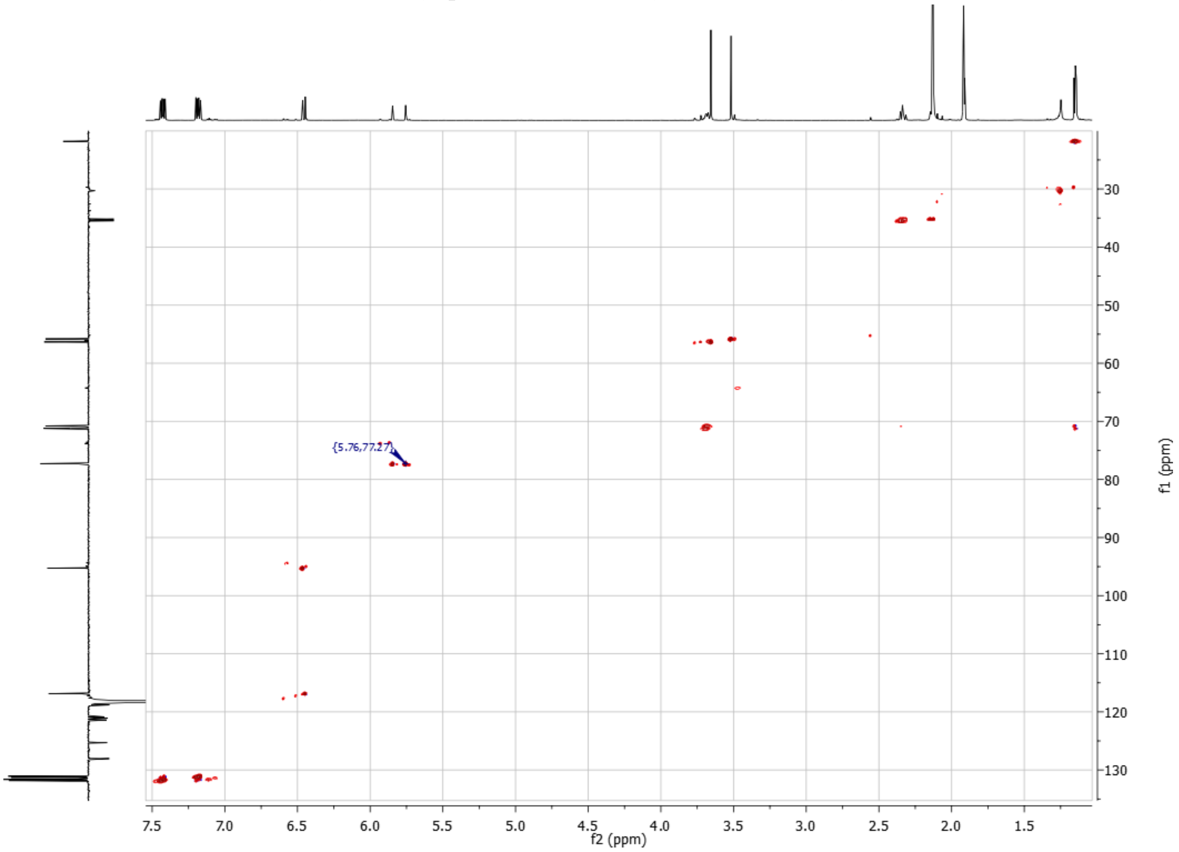

Figure S54. ^{1}H-^{13}C HSQC NMR (700 MHz) spectrum of *cis*,*cis*-(a*R*,1*R*,3*S*,1’*R*,3’*S*)-**21** in acetonitrile-d_3.

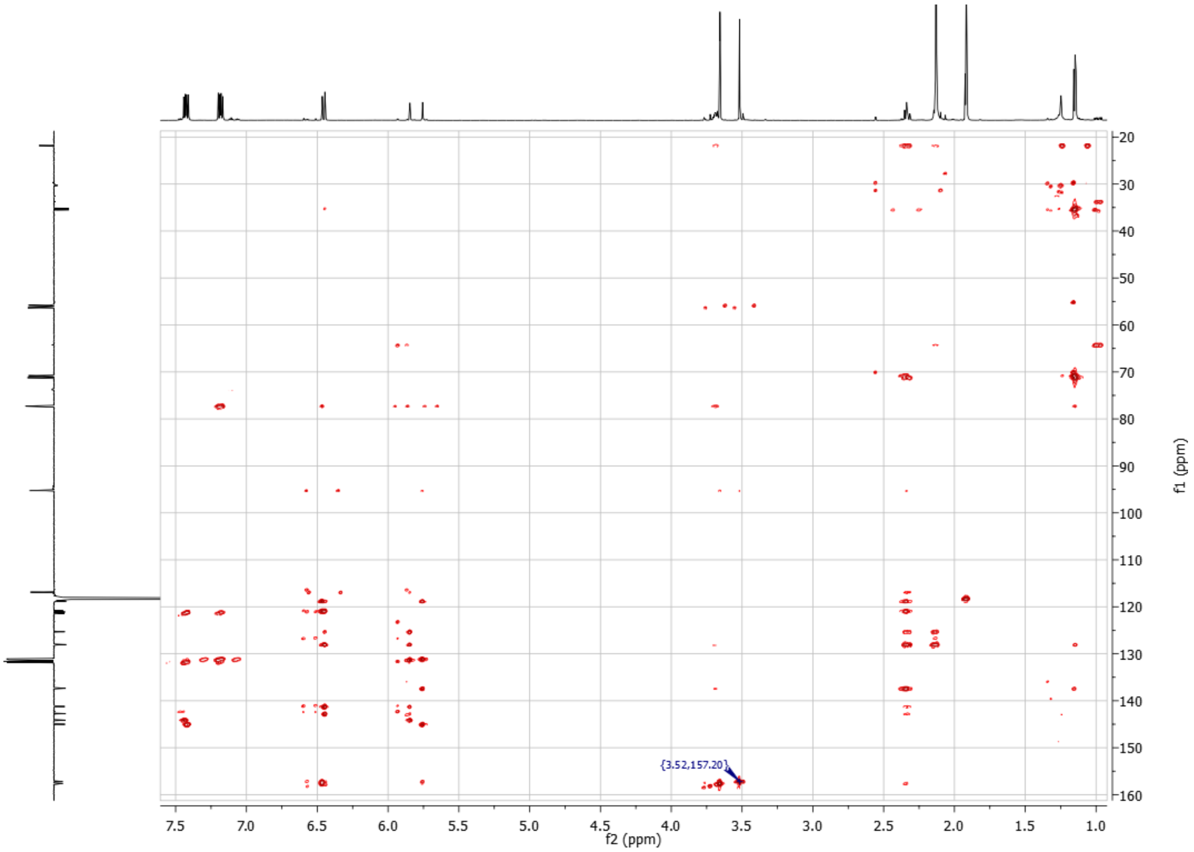

Figure S55. ^{1}H-^{13}C HMBC NMR (700 MHz) spectrum of *cis*,*cis*-(a*R*,1*R*,3*S*,1'*R*,3'*S*)-**21** in acetonitrile-d_3.

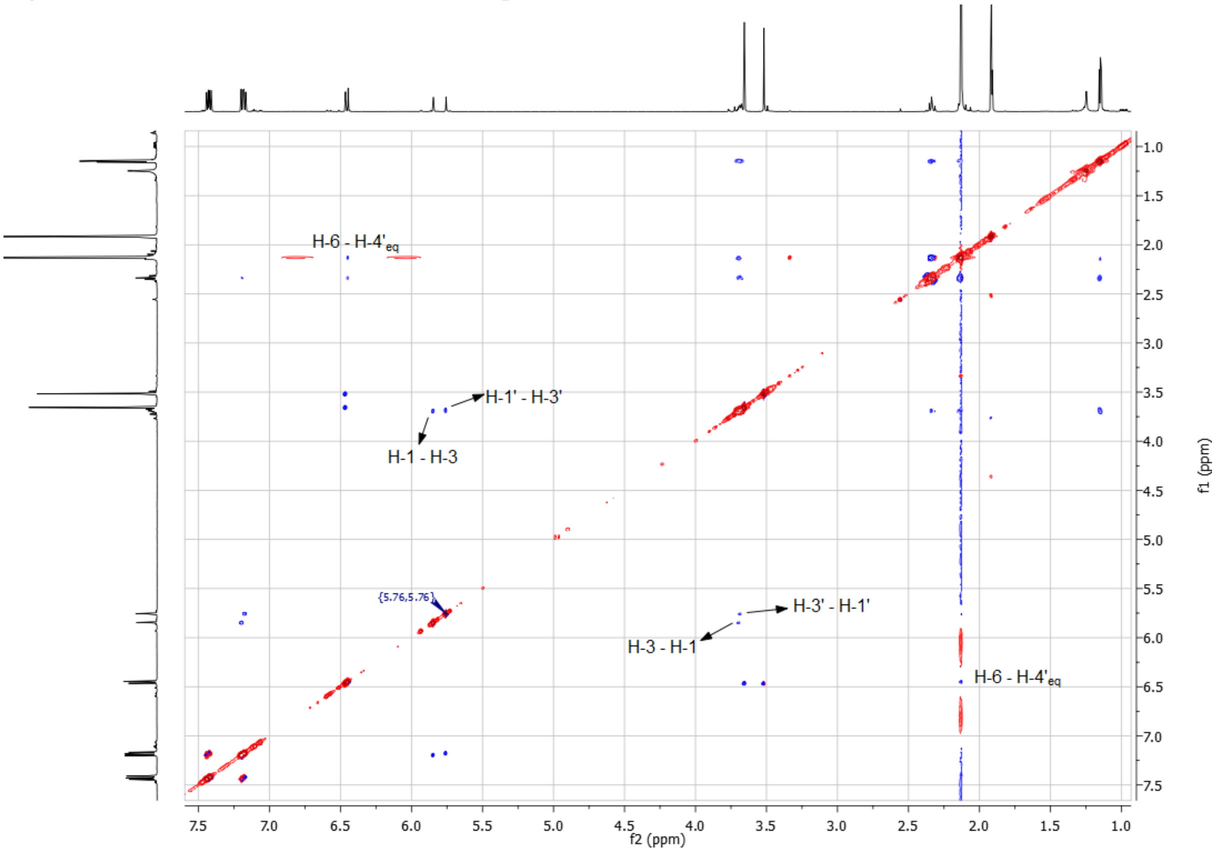

Figure S56. ^{1}H-^{1}H ROESY NMR (700 MHz) spectrum of *cis*,*cis*-(a*R*,1*R*,3*S*,1'*R*,3'*S*)-**21** in acetonitrile-d_3.

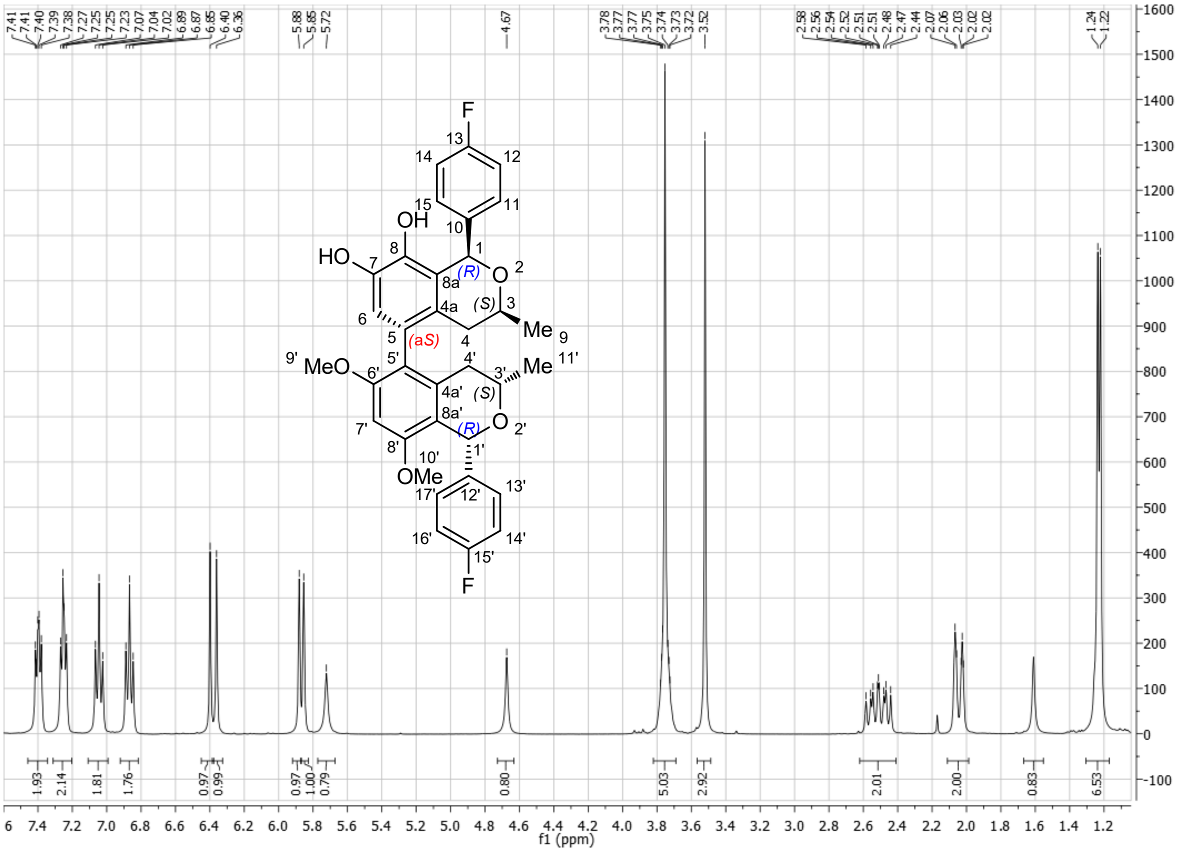

Figure S57. ^{1}H NMR (400 MHz) spectrum of *cis*,*cis*-(a*S*,1*R*,3*S*,1'*R*,3'*S*)-**20** in $CDCl_3$.

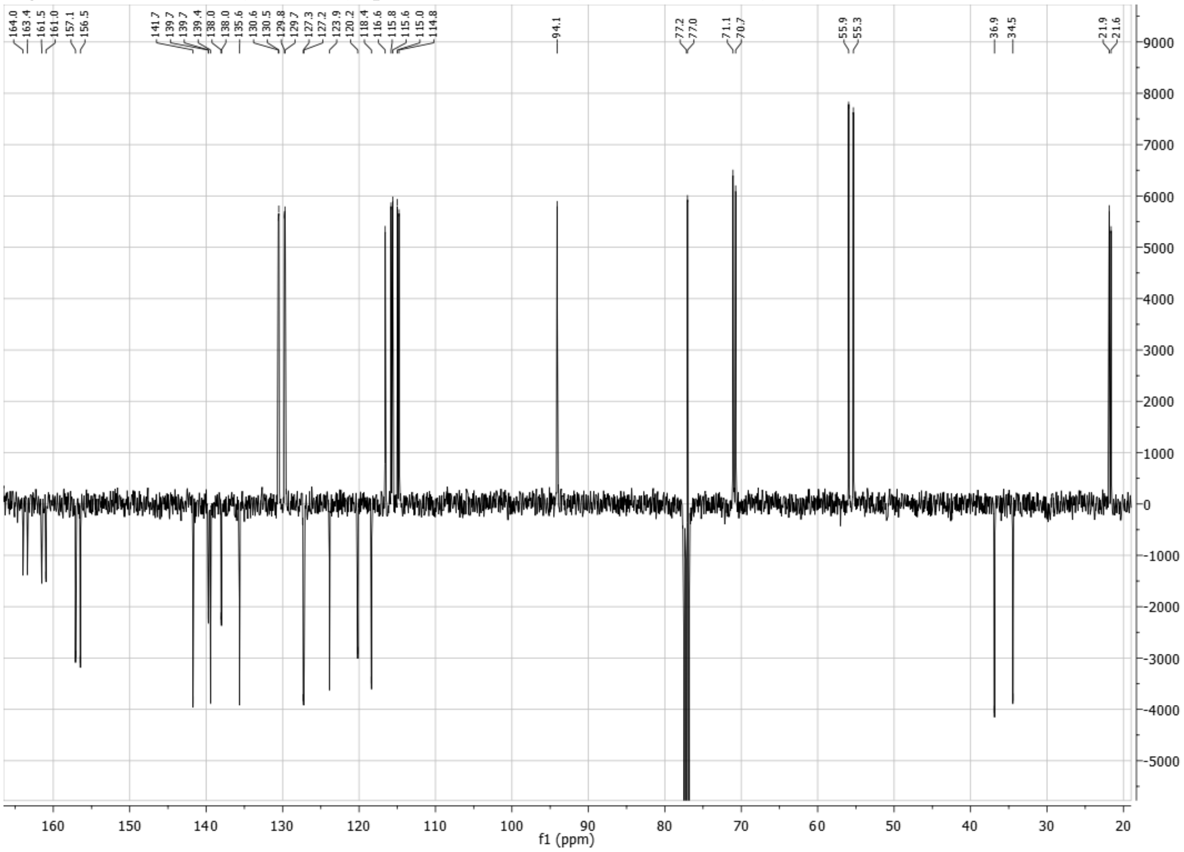

Figure S58. ^{13}C NMR (100 MHz) spectrum of *cis*,*cis*-(a*S*,1*R*,3*S*,1'*R*,3'*S*)-**20** in $CDCl_3$.

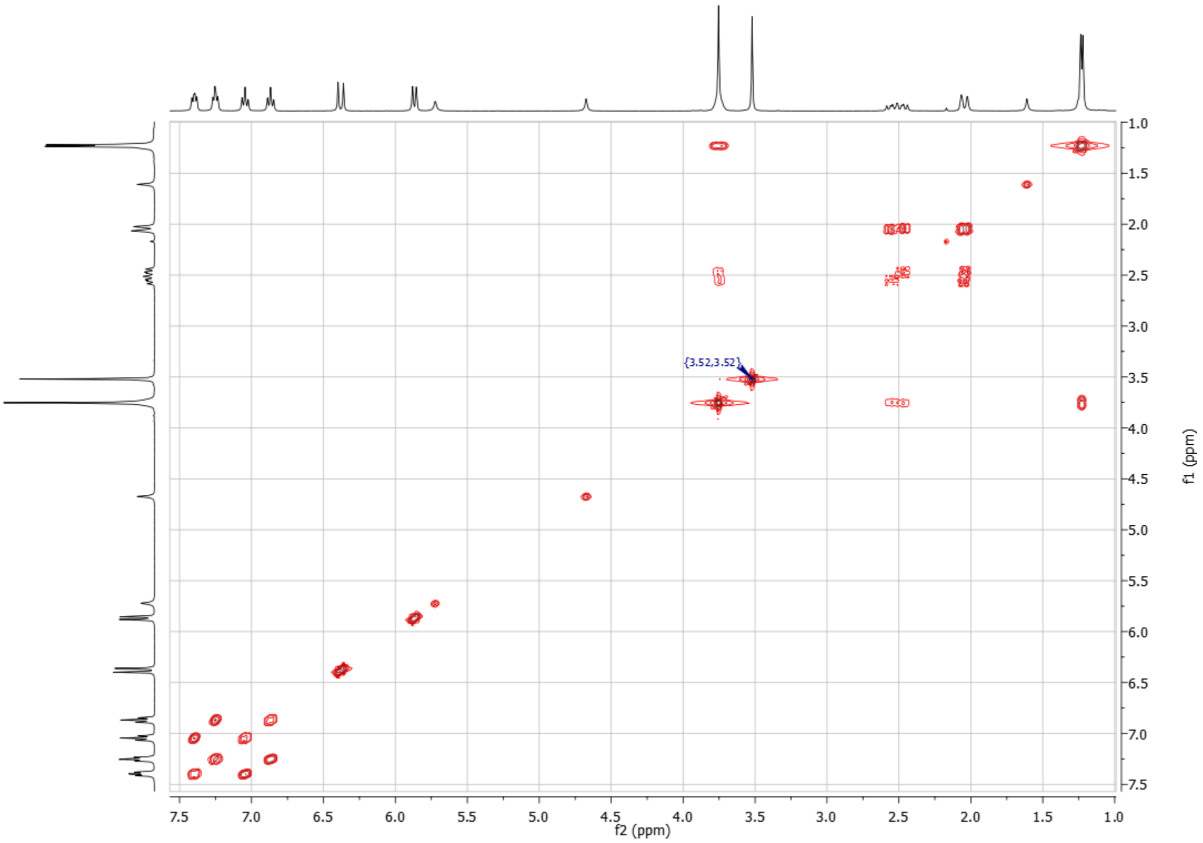

Figure S59. ^{1}H-^{1}H COSY NMR (400 MHz) spectrum of *cis*,*cis*-(a*S*,1*R*,3*S*,1'*R*,3'*S*)-**20** in $CDCl_3$.

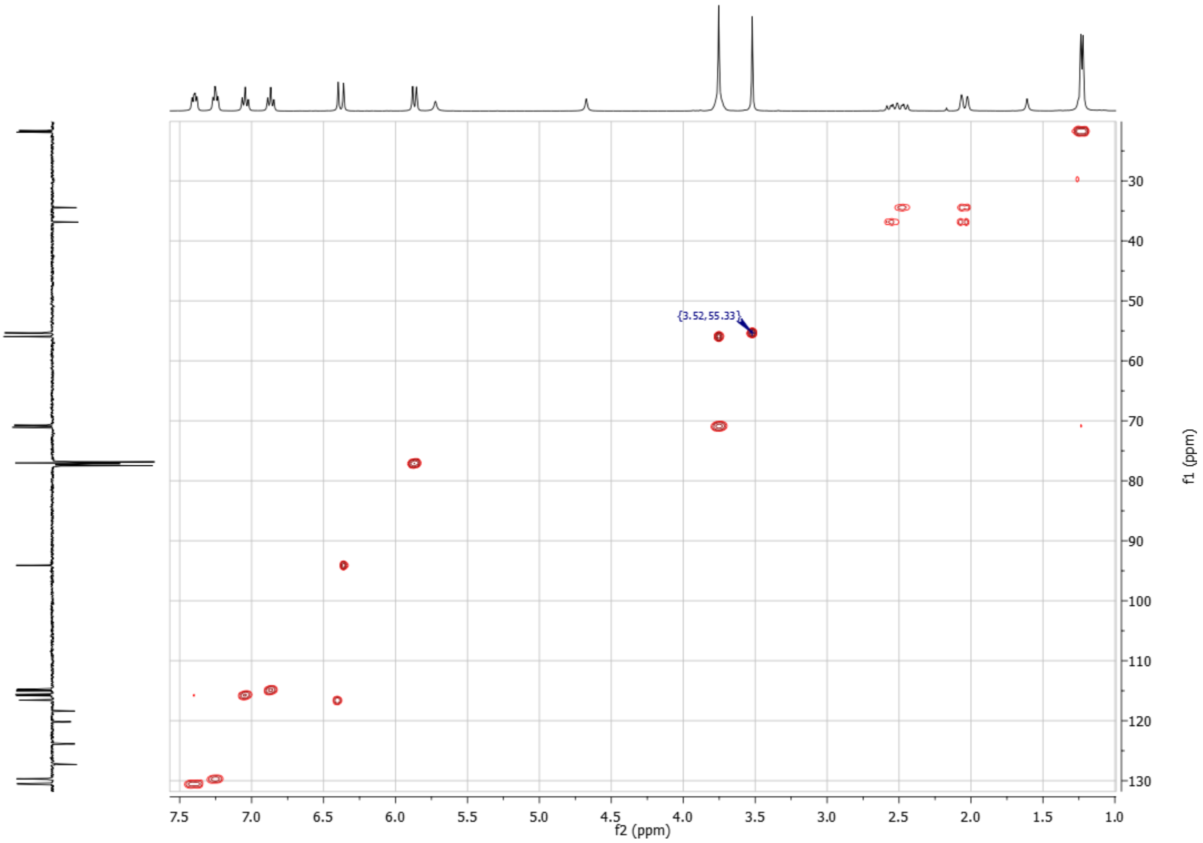

Figure S60. ^{1}H-^{13}C HSQC NMR (400 MHz) spectrum of *cis*,*cis*-(a*S*,1*R*,3*S*,1'*R*,3'*S*)-**20** in $CDCl_3$.

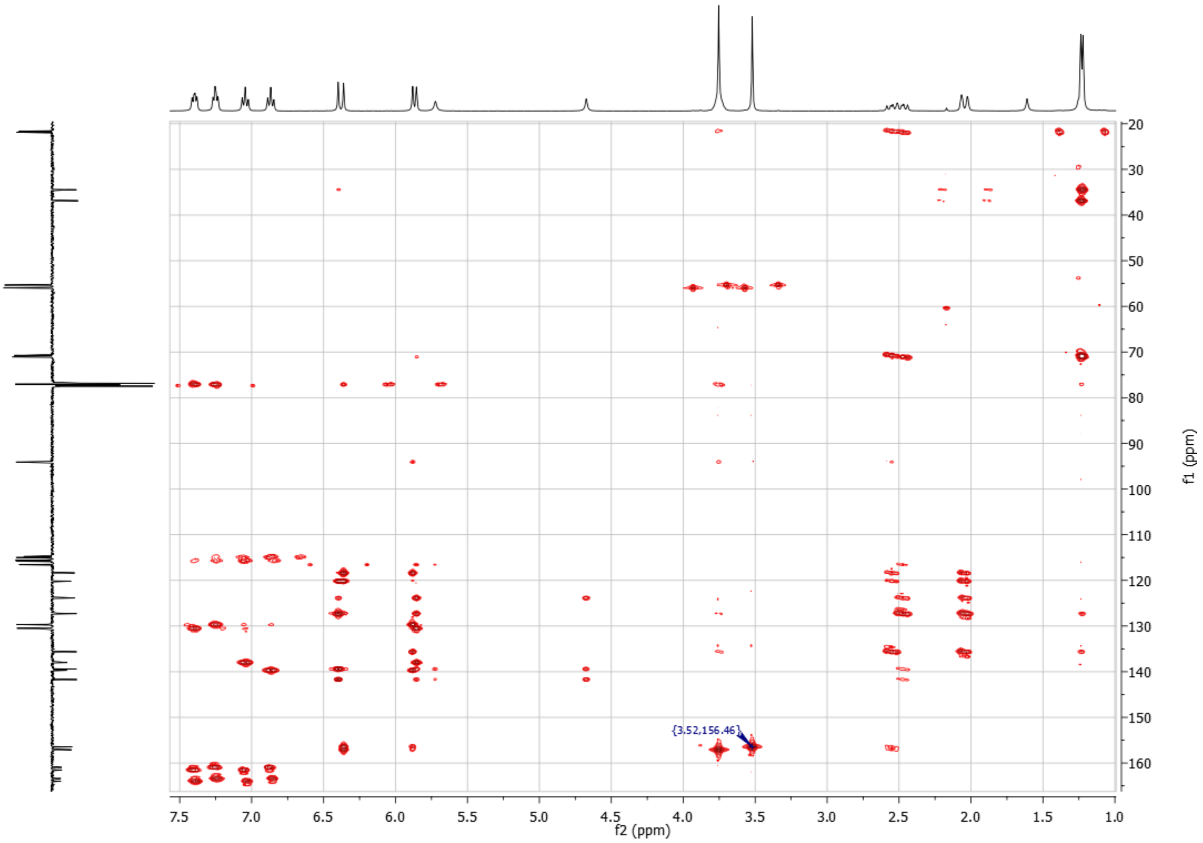

Figure S61. ^{1}H-^{13}C HMBC NMR (400 MHz) spectrum of *cis*,*cis*-(a*S*,1*R*,3*S*,1'*R*,3'*S*)-**20** in $CDCl_3$.

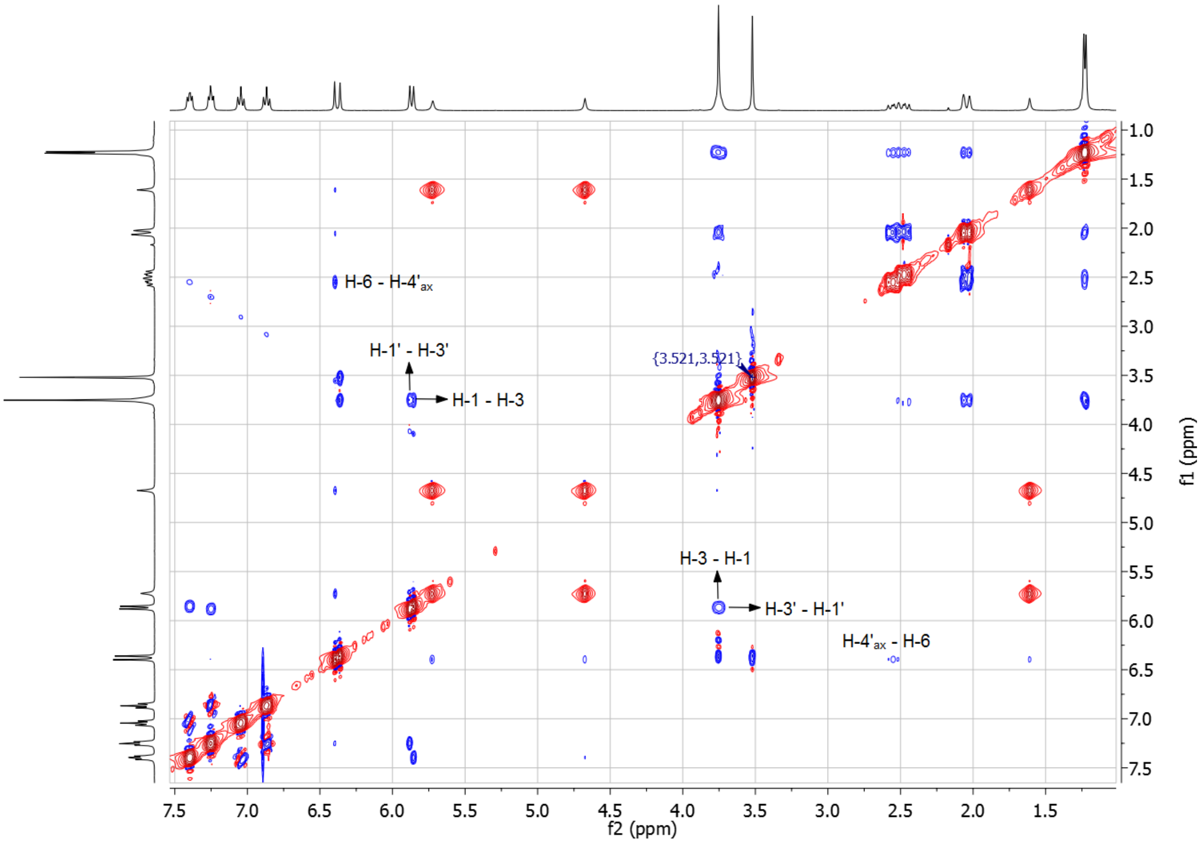

Figure S62. ^{1}H-^{1}H ROESY NMR (400 MHz) spectrum of *cis*,*cis*-(a*S*,1*R*,3*S*,1'*R*,3'*S*)-**20** in $CDCl_3$.

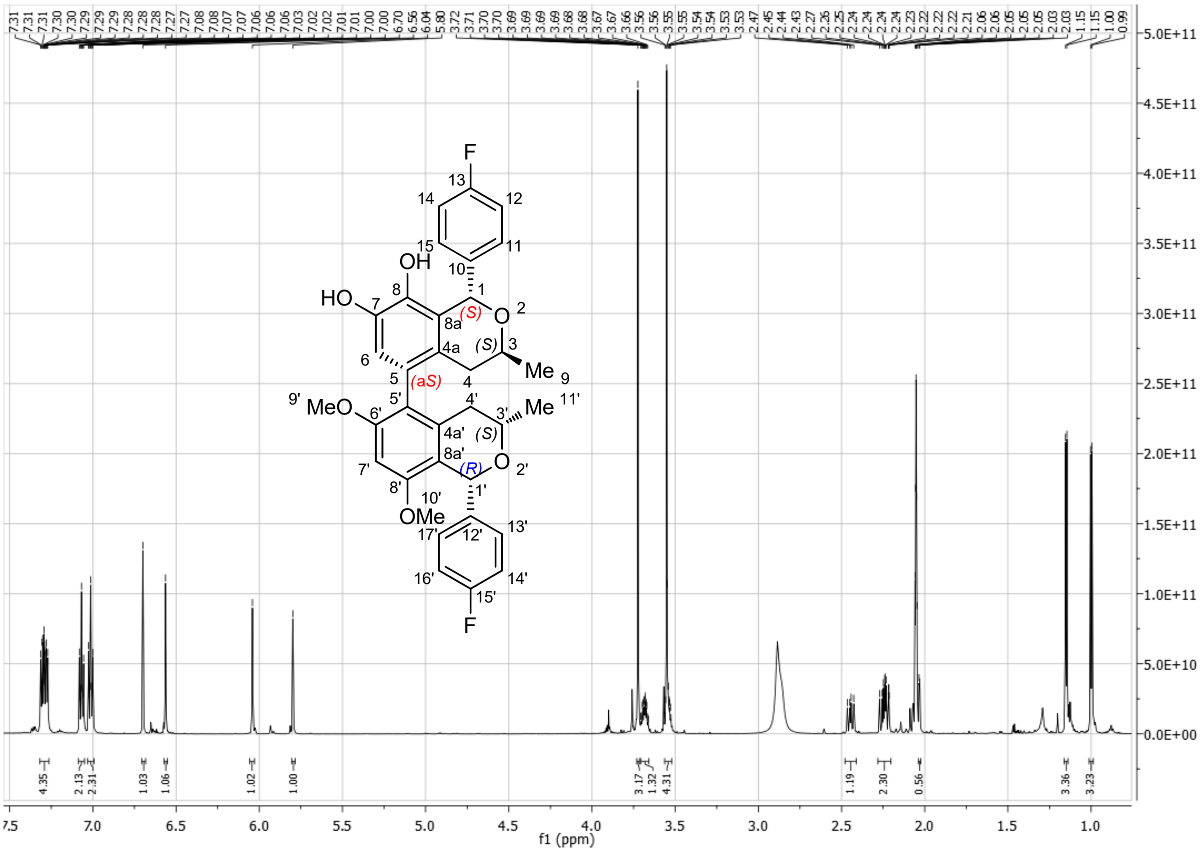

Figure S63. ^{1}H NMR (700 MHz) spectrum of *trans*,*cis*-(a*S*,1*S*,3*S*,1'*R*,3'*S*)-**20** in acetone-d_6.

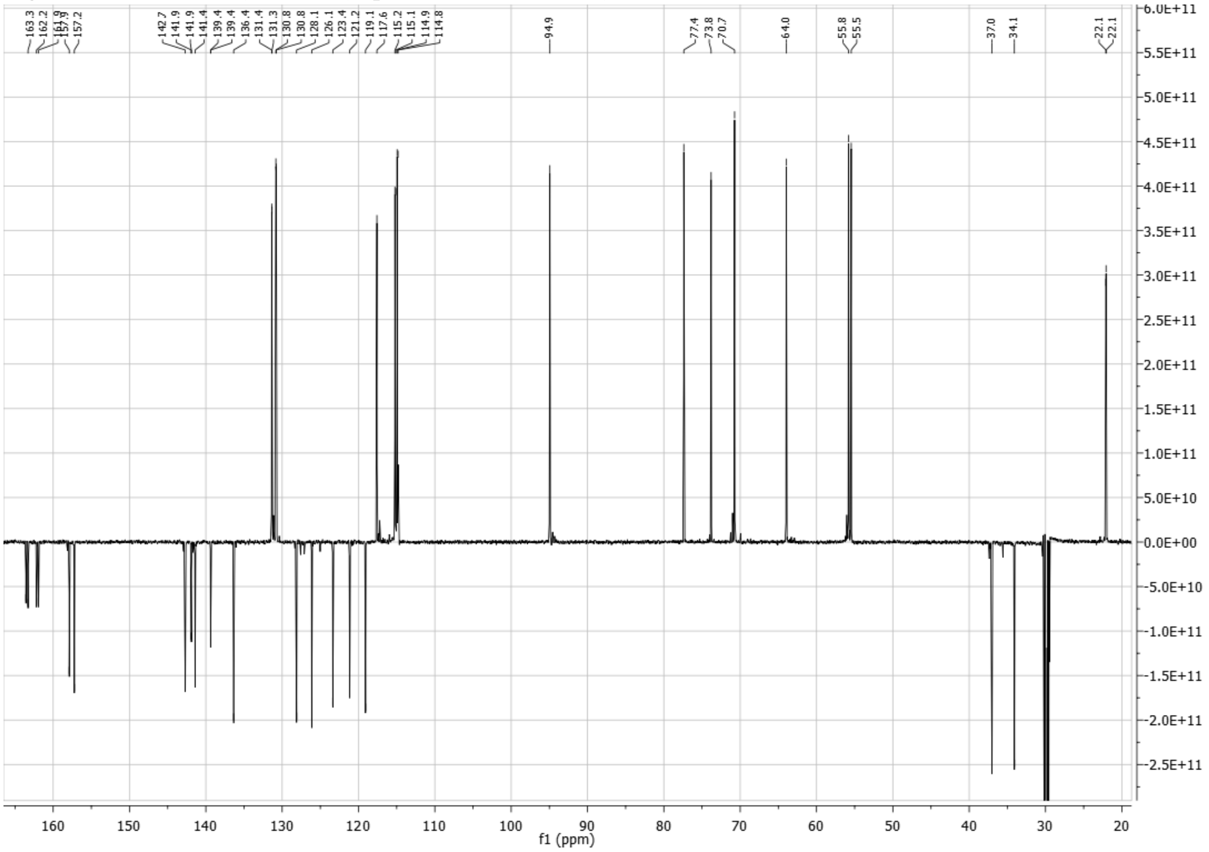

Figure S64. ^{13}C NMR (175 MHz) spectrum of *trans*,*cis*-(a*S*,1*S*,3*S*,1'*R*,3'*S*)-**20** in acetone-d_6.

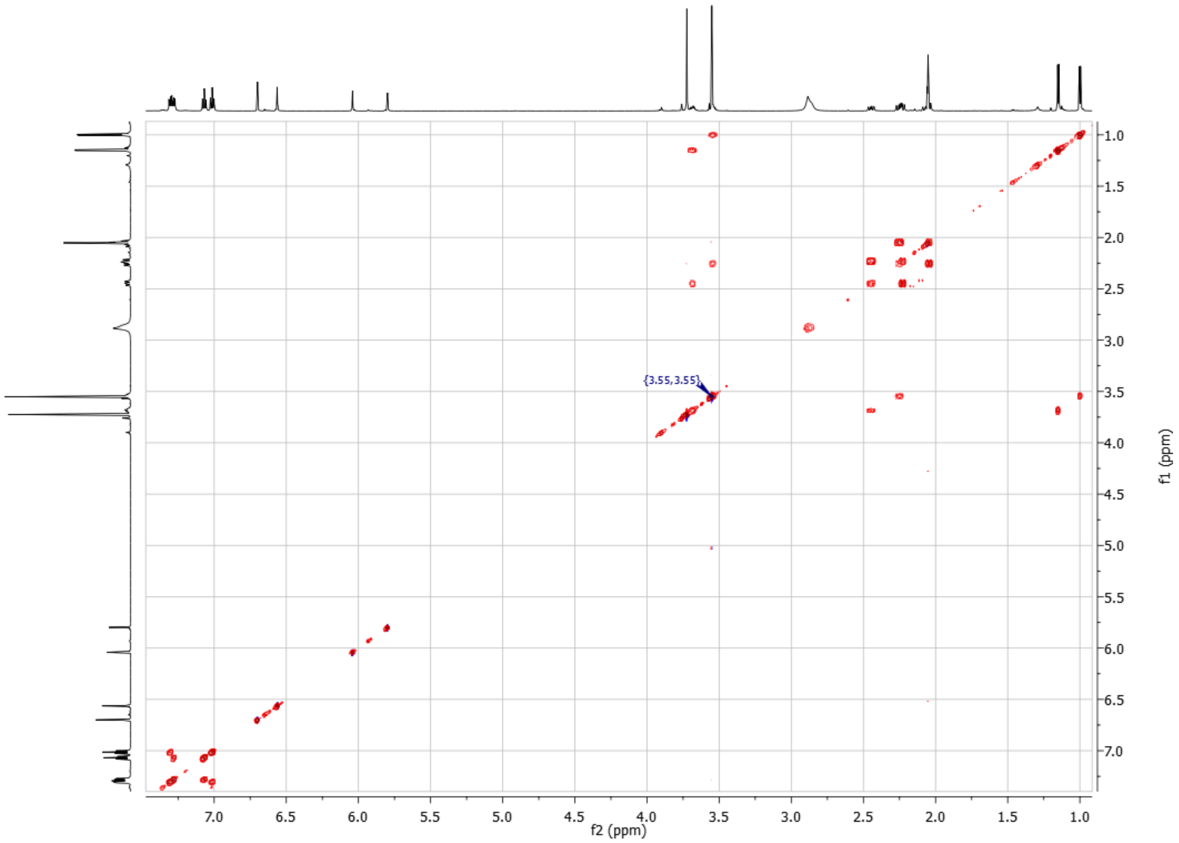

Figure S65. ^{1}H-^{1}H COSY NMR (700 MHz) spectrum of *trans*,*cis*-(a*S*,1*S*,3*S*,1’*R*,3’*S*)-**20** in acetone-d_6.

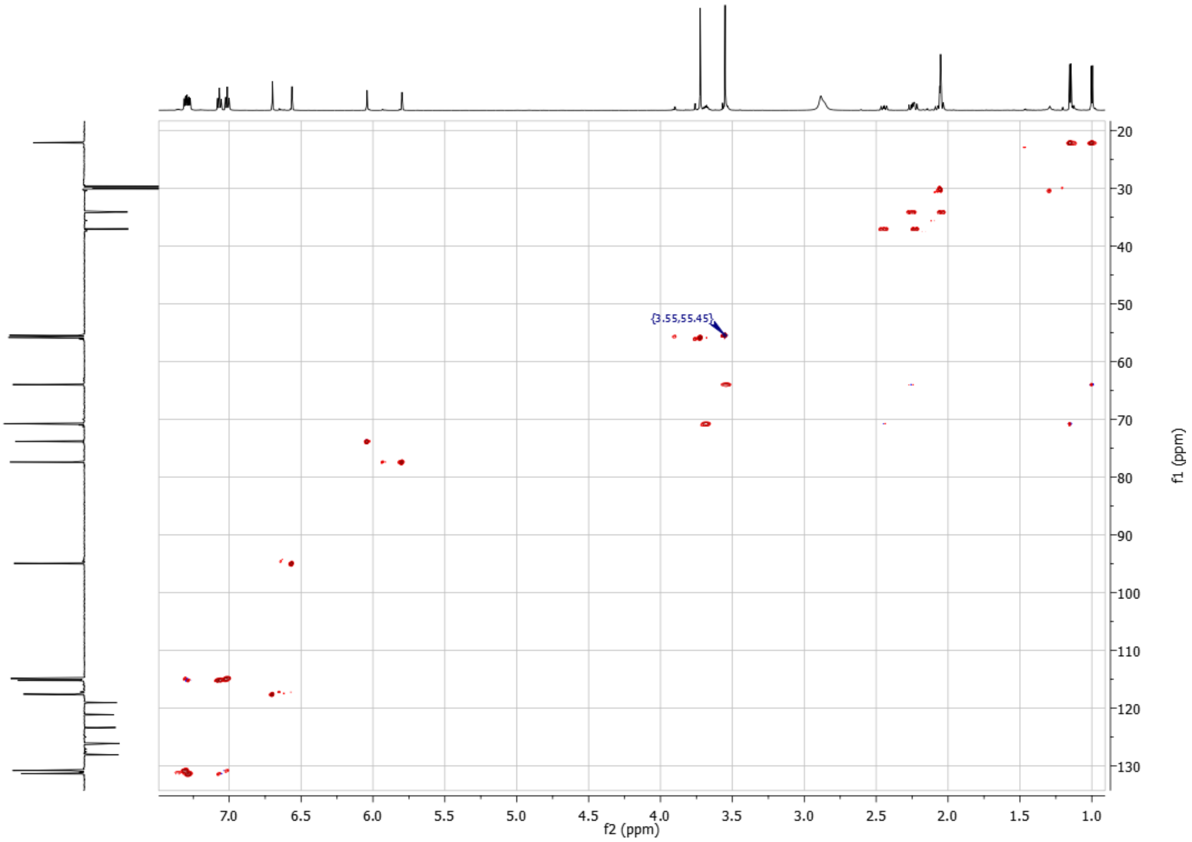

Figure S66. ^{1}H-^{13}C HSQC NMR (700 MHz) spectrum of *trans*,*cis*-(a*S*,1*S*,3*S*,1’*R*,3’*S*)-**20** in acetone-d_6.

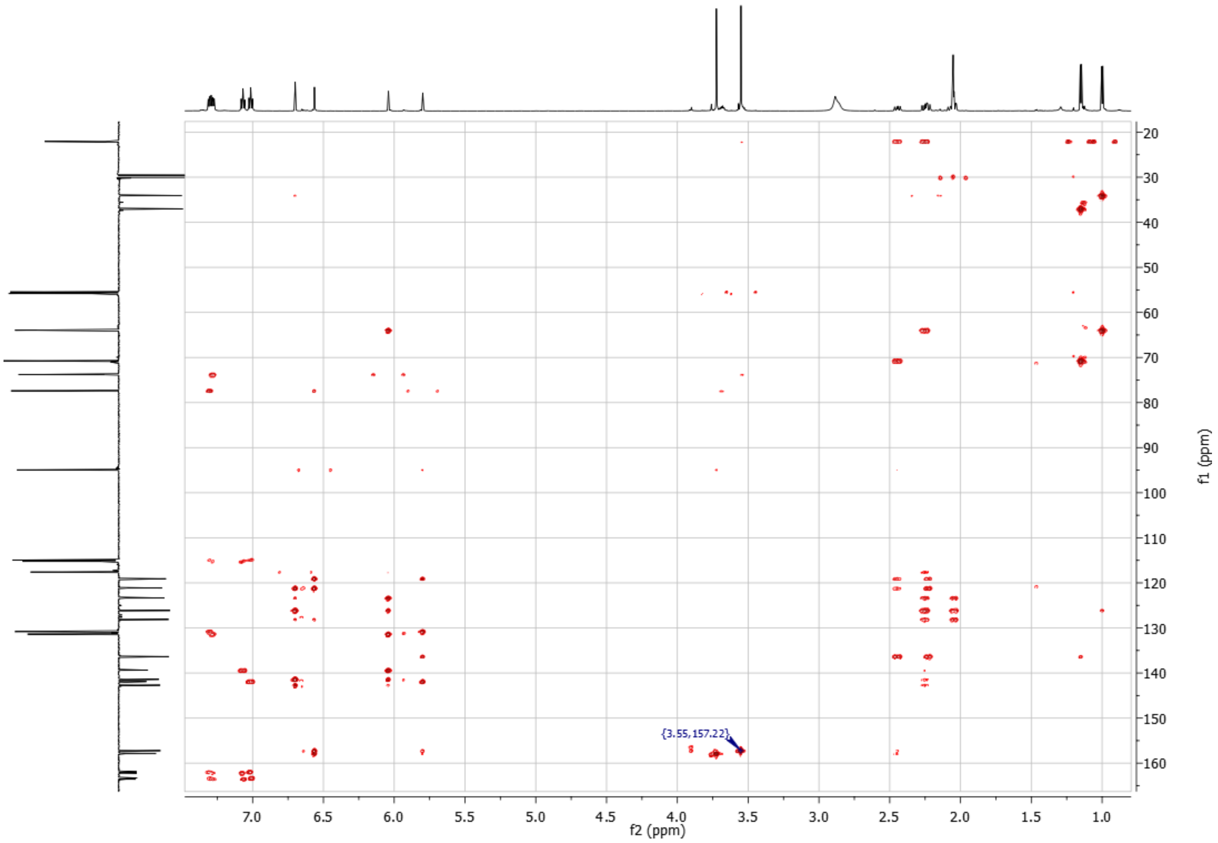

Figure S67. ^{1}H-^{13}C HMBC NMR (700 MHz) spectrum of *trans*,*cis*-(a*S*,1*S*,3*S*,1'*R*,3'*S*)-**20** in acetone-d_6.

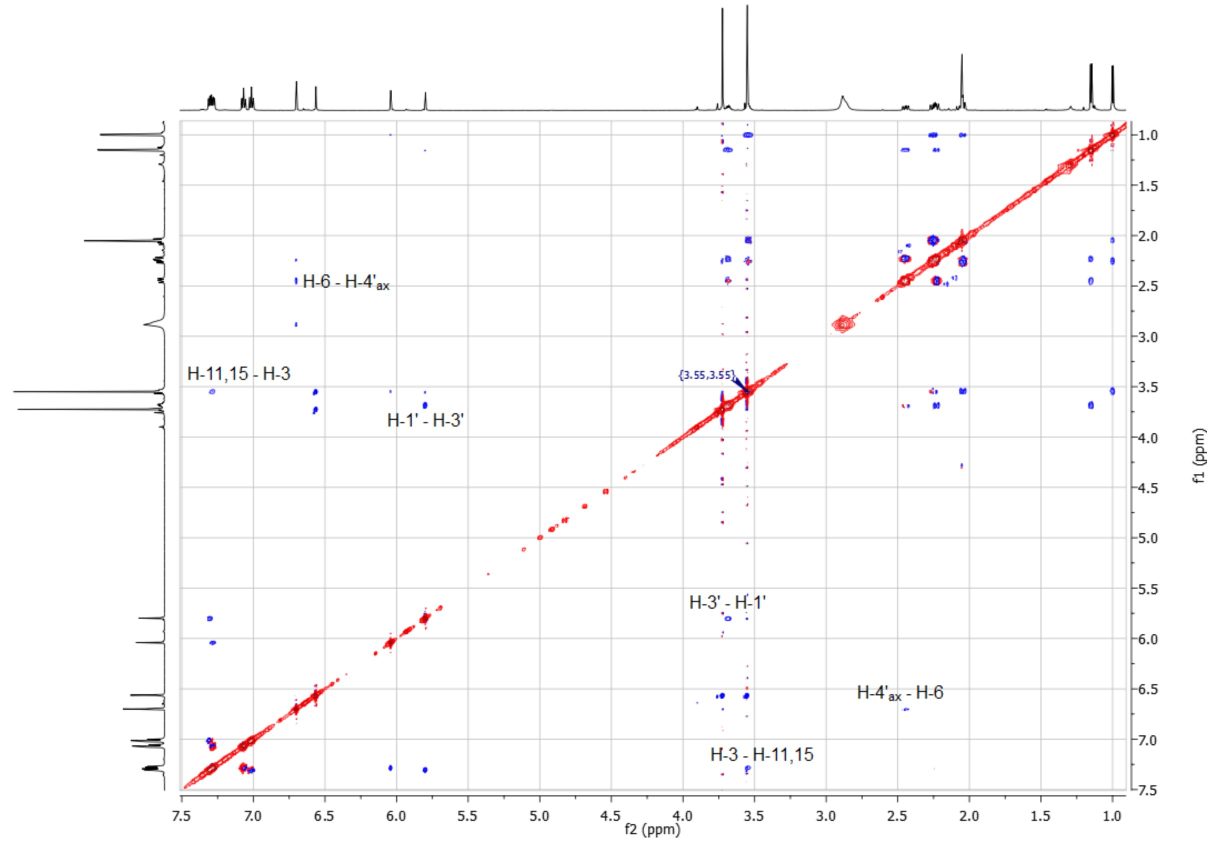

Figure S68. ^{1}H-^{1}H ROESY NMR (700 MHz) spectrum of *trans*,*cis*-(a*S*,1*S*,3*S*,1'*R*,3'*S*)-**20** in acetone-d_6.

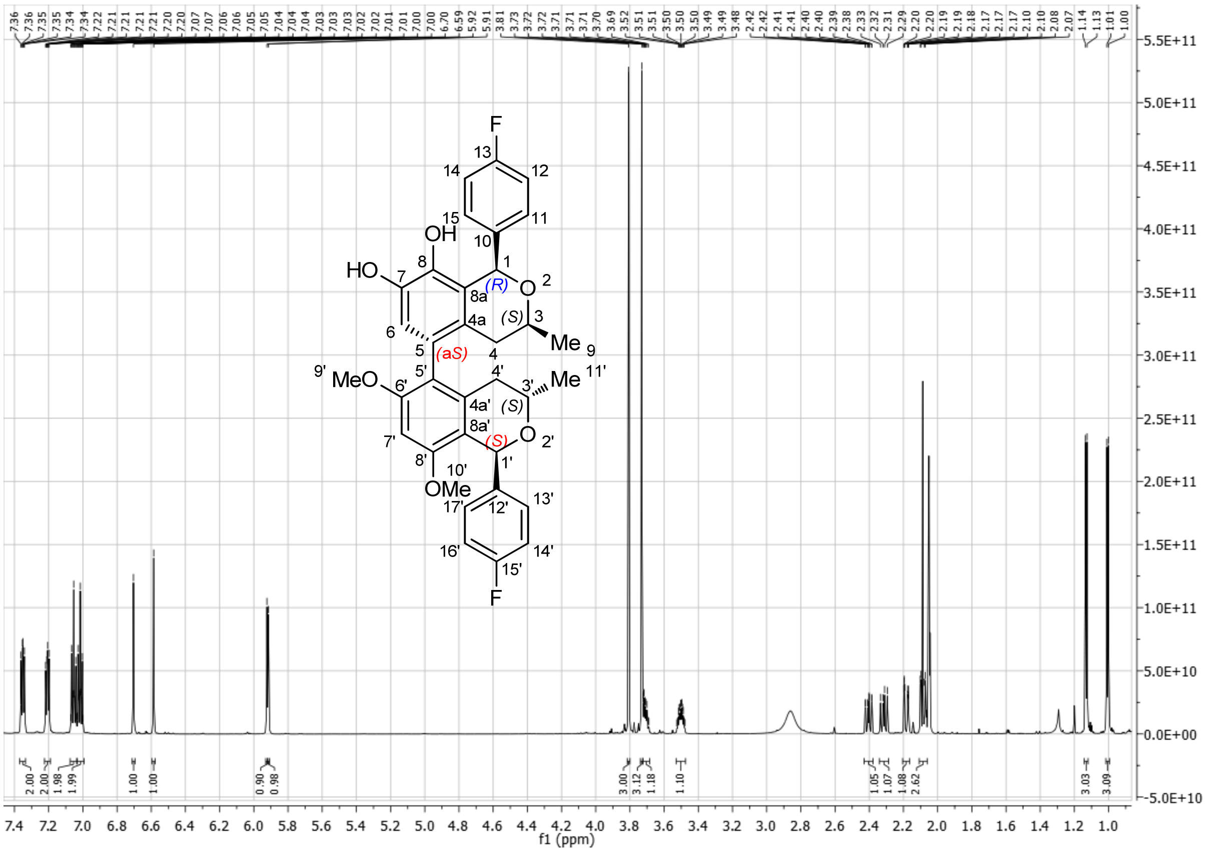

Figure S69. ^{1}H NMR (700 MHz) spectrum of *cis*,*trans*-(a*S*,1*R*,3*S*,1’*S*,3’*S*)-**20** in acetone-d_6.

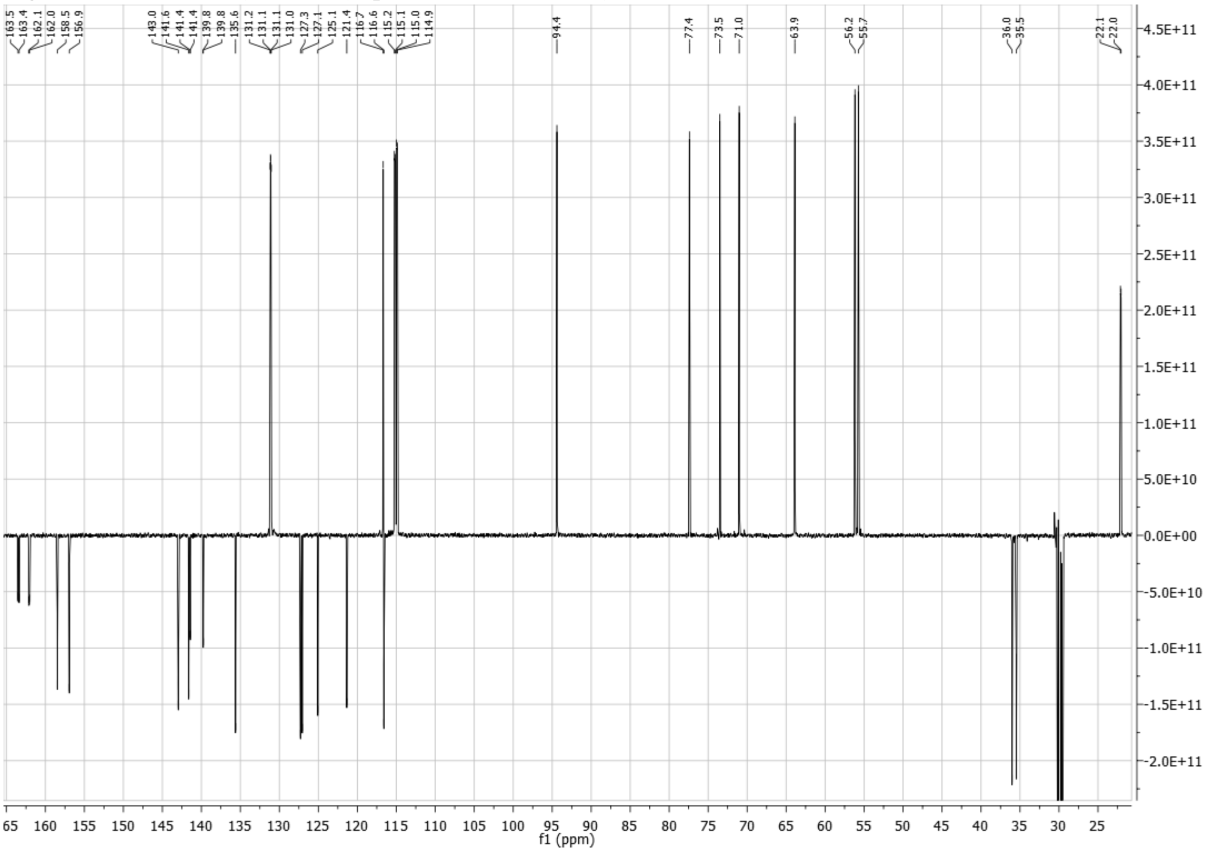

Figure S70. ^{13}C NMR (175 MHz) spectrum of *cis*,*trans*-(a*S*,1*R*,3*S*,1’*S*,3’*S*)-**20** in acetone-d_6.

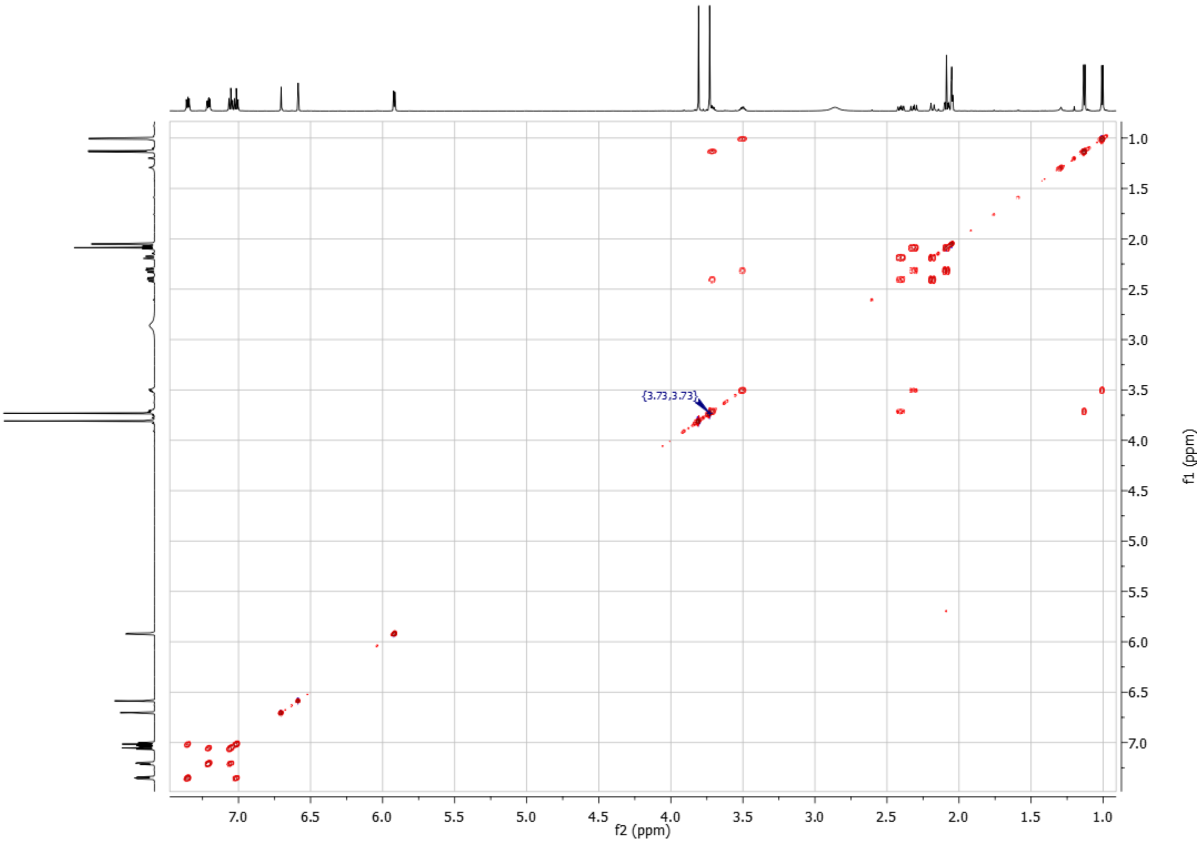

Figure S71. ^{1}H-^{1}H COSY NMR (700 MHz) spectrum of *cis*,*trans*-(a*S*,1*R*,3*S*,1'*S*,3'*S*)-**20** in acetone-d_6.

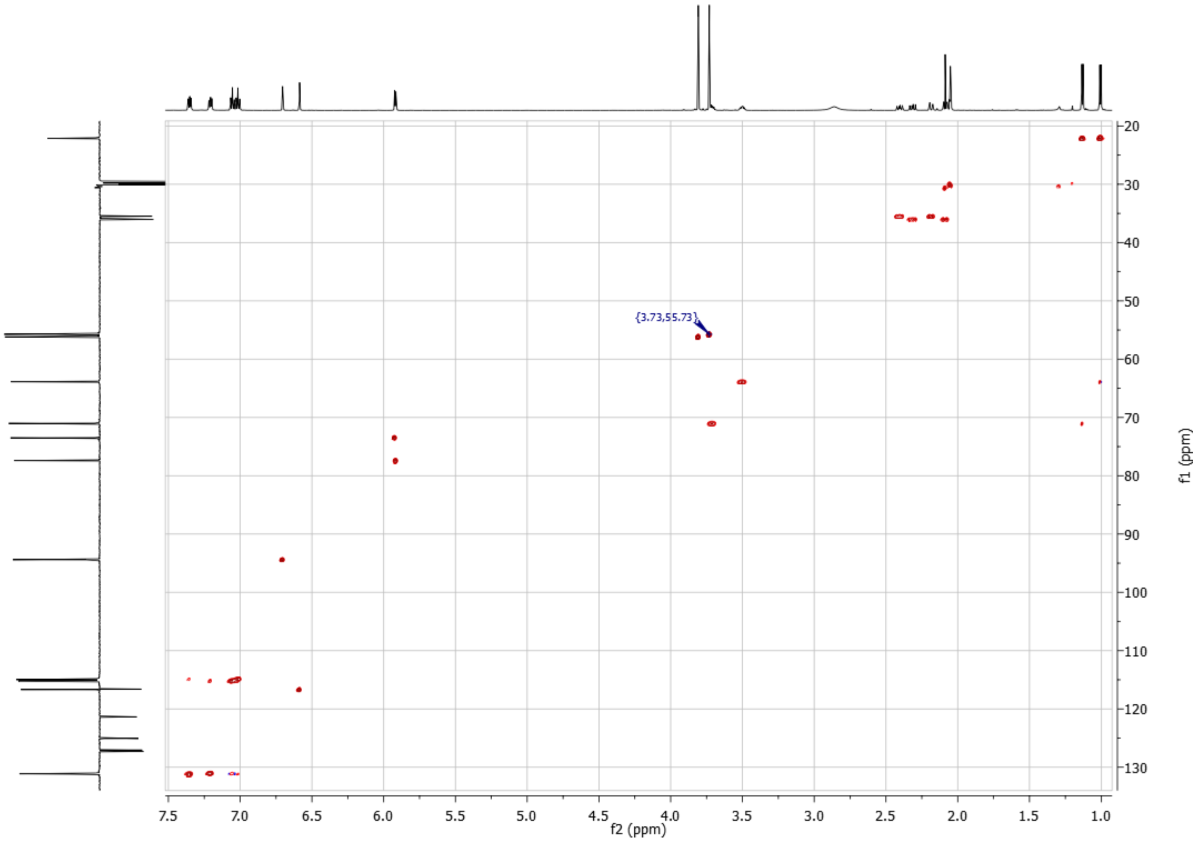

Figure S72. ^{1}H-^{13}C HSQC NMR (700 MHz) spectrum of *cis*,*trans*-(a*S*,1*R*,3*S*,1'*S*,3'*S*)-**20** in acetone-d_6.

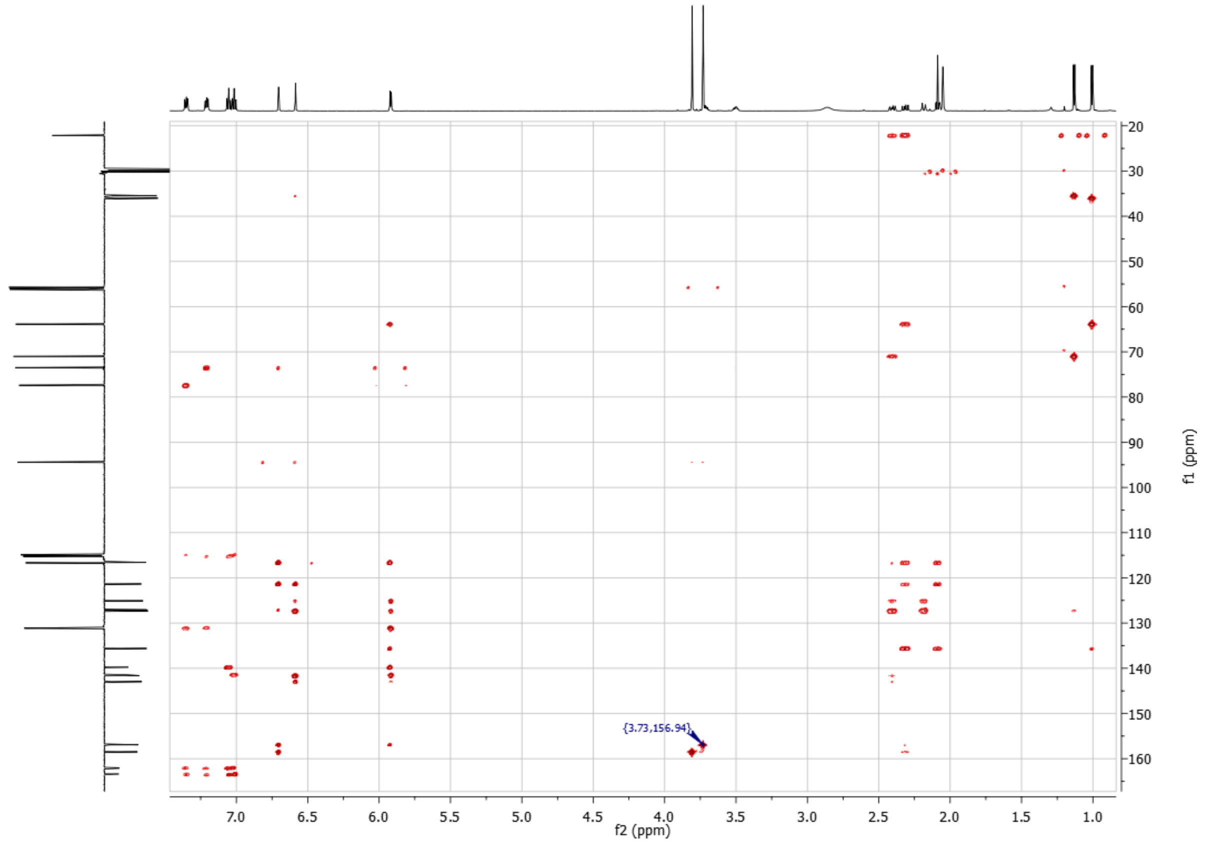

Figure S73. ^{1}H-^{13}C HMBC NMR (700 MHz) spectrum of *cis*,*trans*-(a*S*,1*R*,3*S*,1'*S*,3'*S*)-**20** in acetone-d_6.

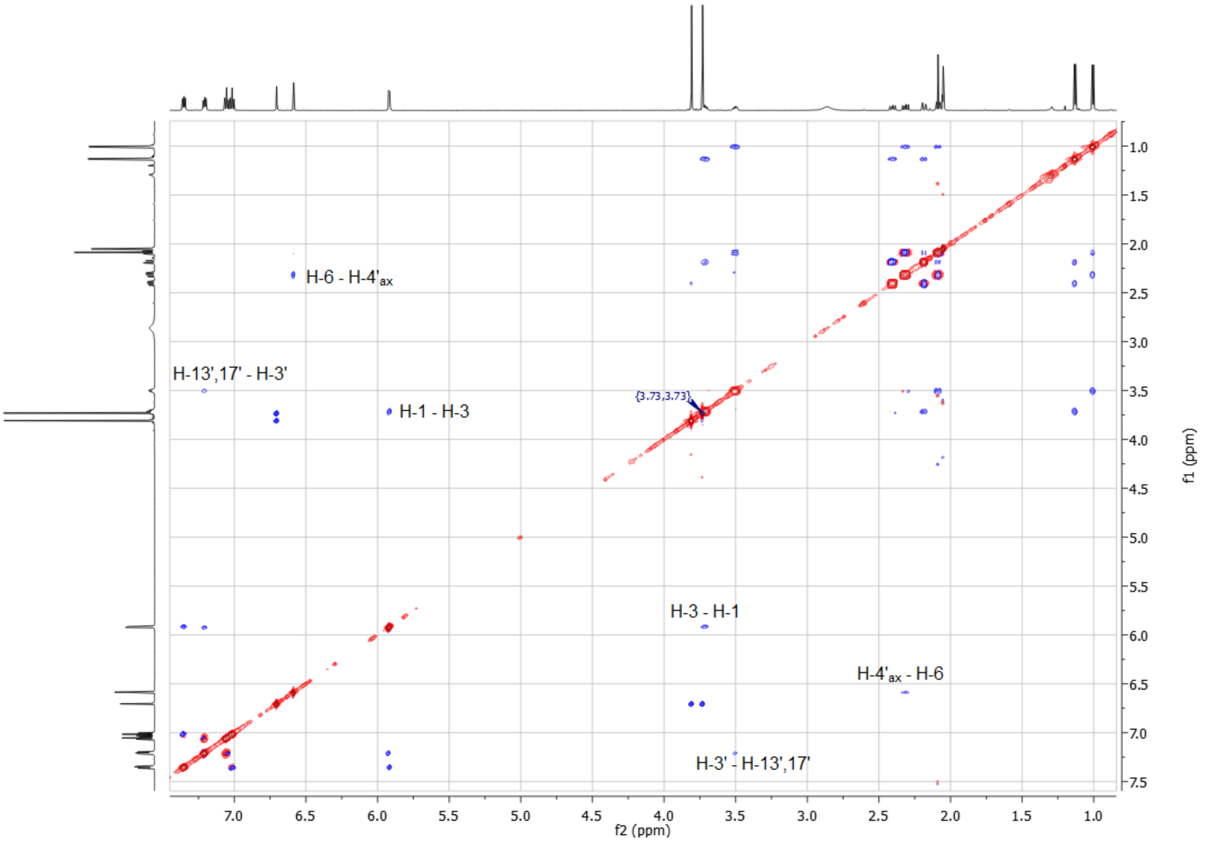

Figure S74. ^{1}H-^{1}H ROESY NMR (700 MHz) spectrum of *cis*,*trans*-(a*S*,1*R*,3*S*,1'*S*,3'*S*)-**20** in acetone-d_6.

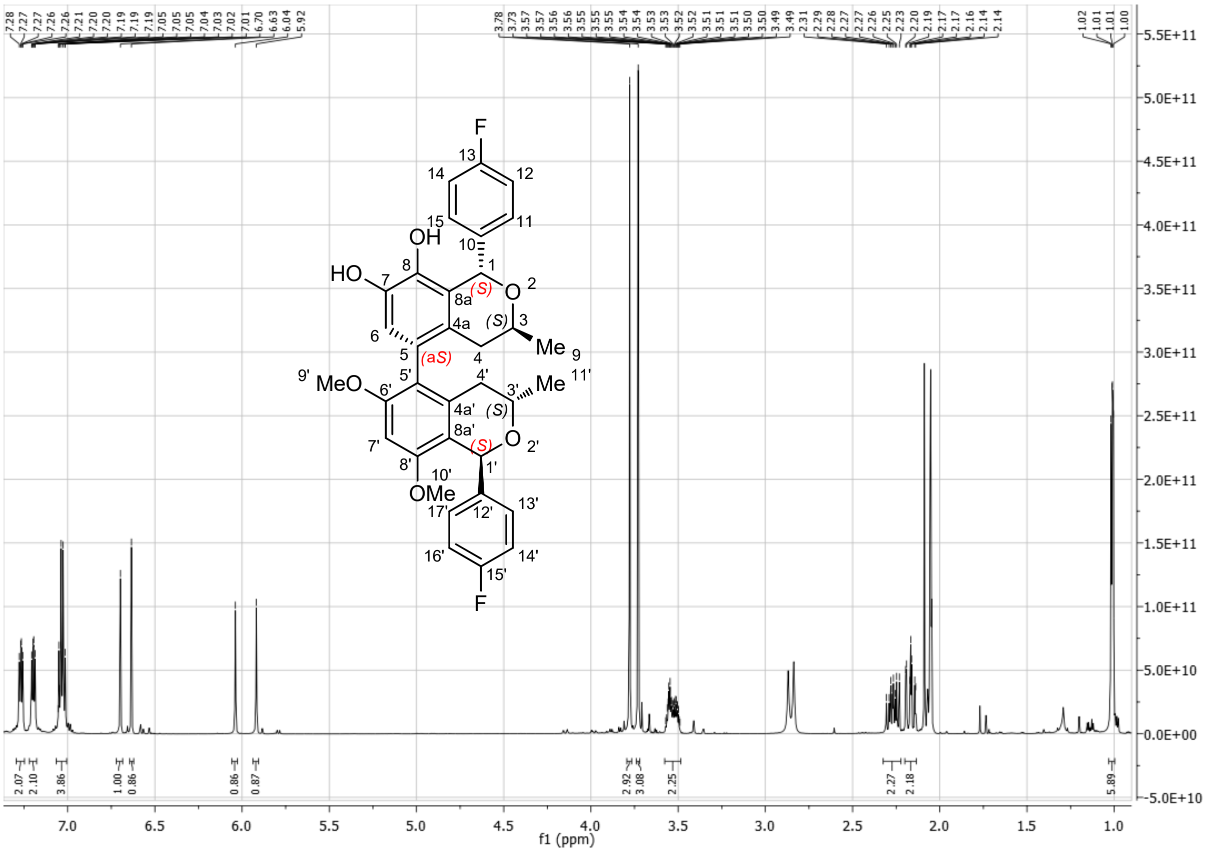

Figure S75. ¹H NMR (700 MHz) spectrum of *trans*,*trans*-(a*S*,1*S*,3*S*,1'*S*,3'*S*)-**20** in acetone-d_6.

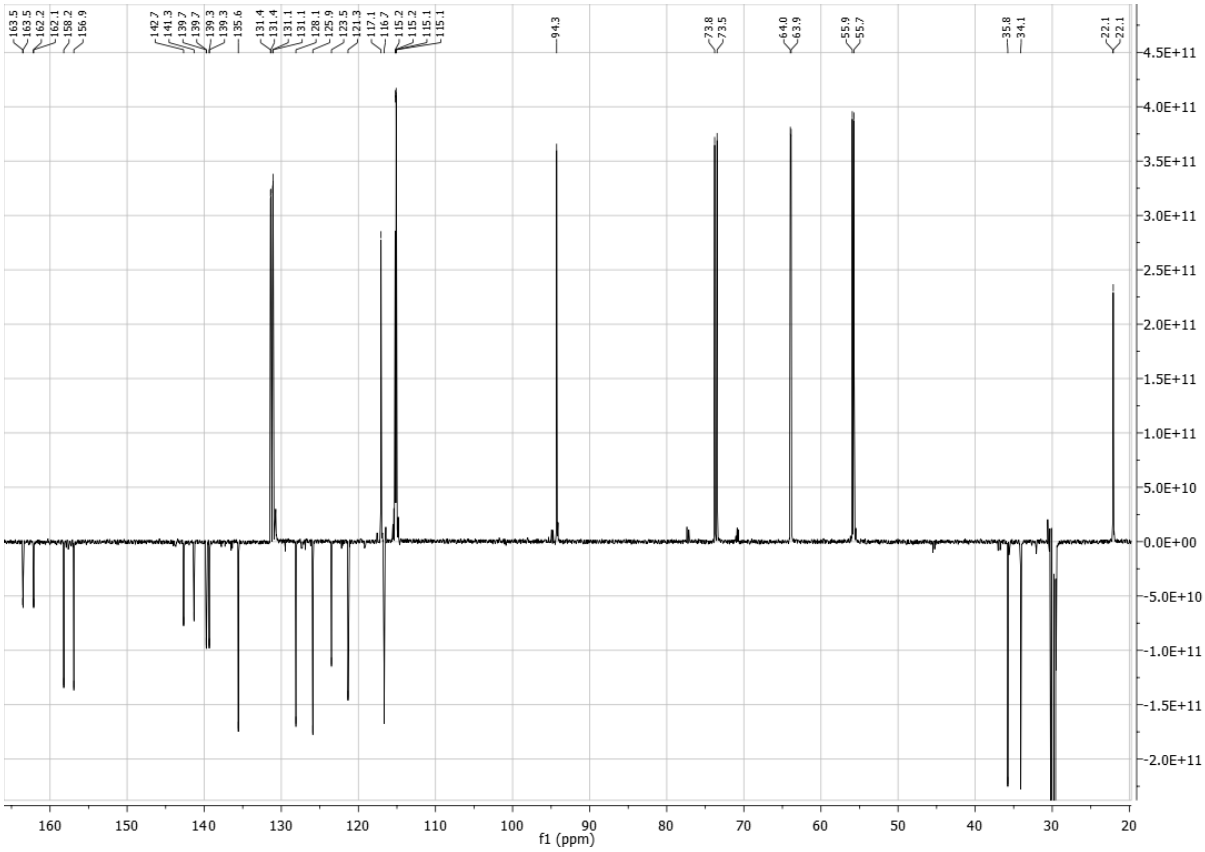

Figure S76. ¹³C NMR (175 MHz) spectrum of *trans*,*trans*-(a*S*,1*S*,3*S*,1'*S*,3'*S*)-**20** in acetone-d_6.

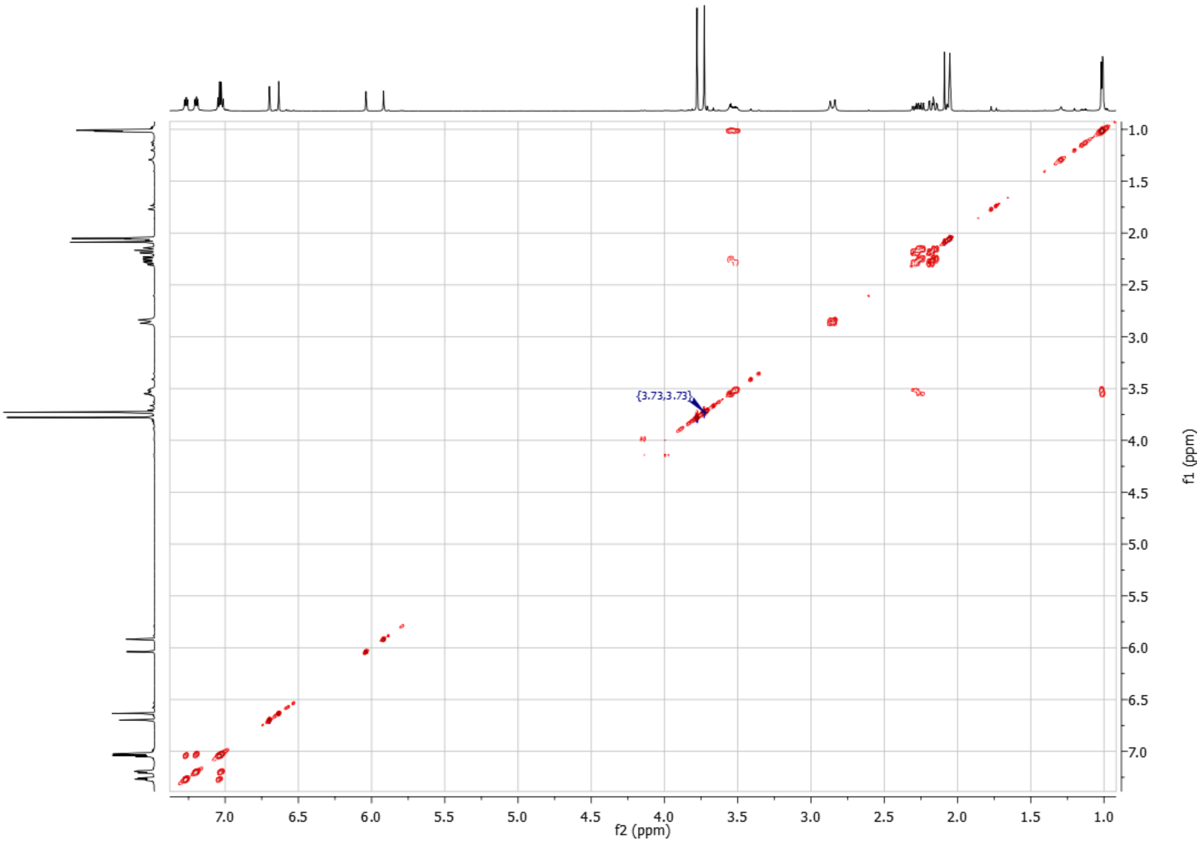

Figure S77. ^{1}H-^{1}H COSY NMR (700 MHz) spectrum of *trans*,*trans*-(a*S*,1*S*,3*S*,1'*S*,3'*S*)-**20** in acetone-d_6.

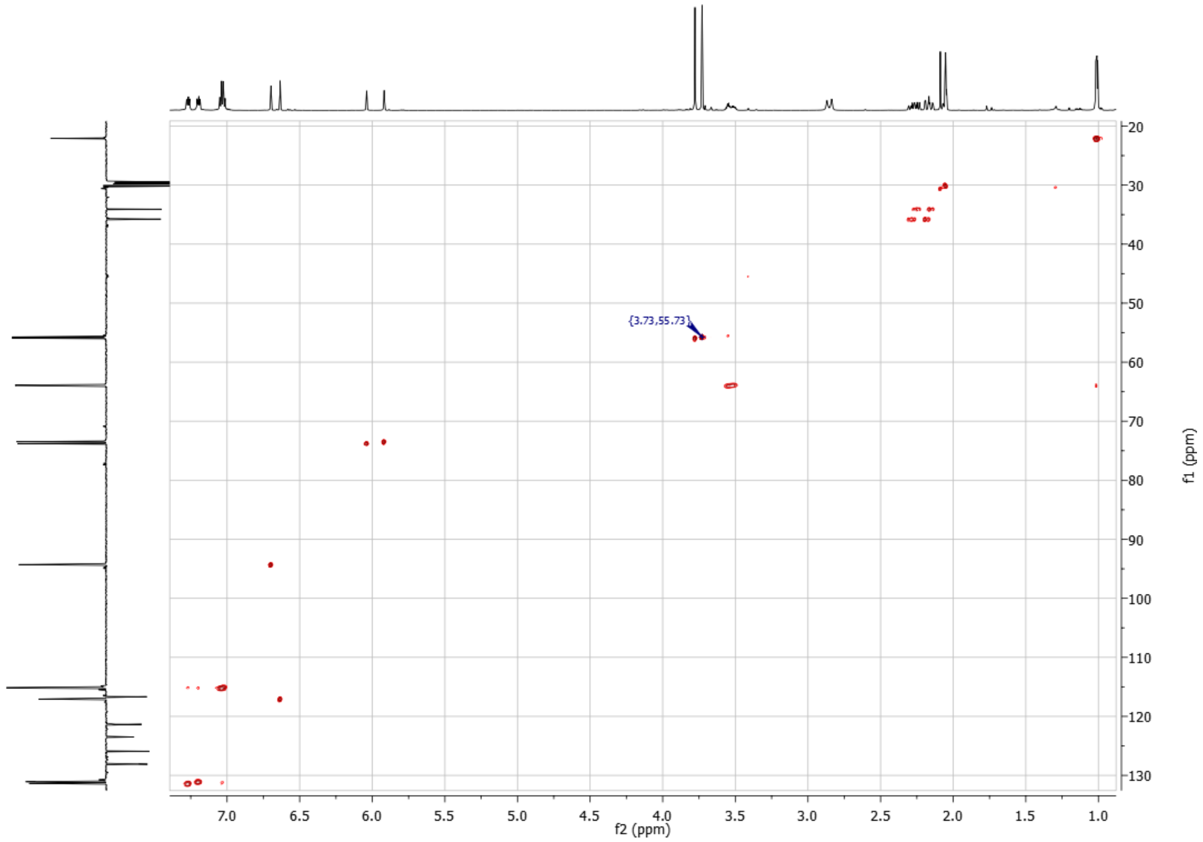

Figure S78. ^{1}H-^{13}C HSQC NMR (700 MHz) spectrum of *trans*,*trans*-(a*S*,1*S*,3*S*,1'*S*,3'*S*)-**20** in acetone-d_6.

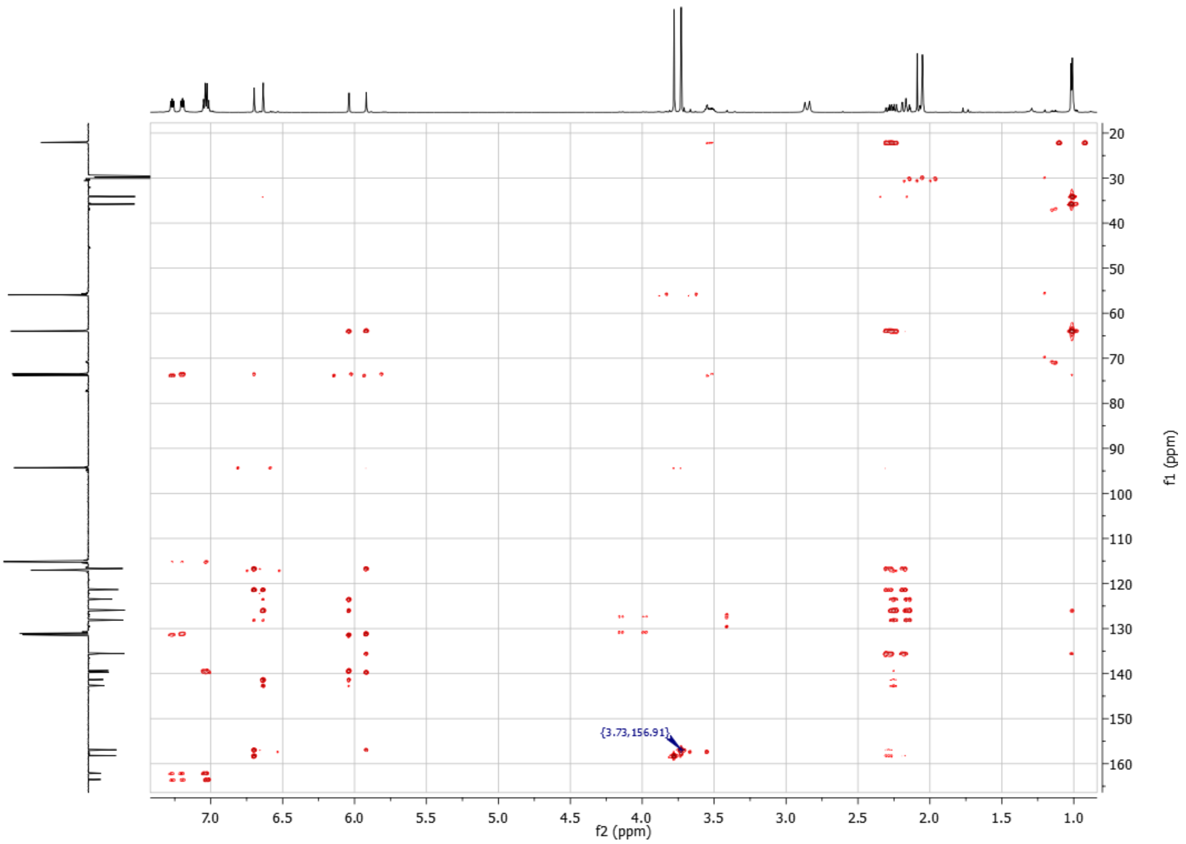

Figure S79. ^{1}H-^{13}C HMBC NMR (700 MHz) spectrum of *trans*,*trans*-(a*S*,1*S*,3*S*,1'*S*,3'*S*)-**20** in acetone-d_6.

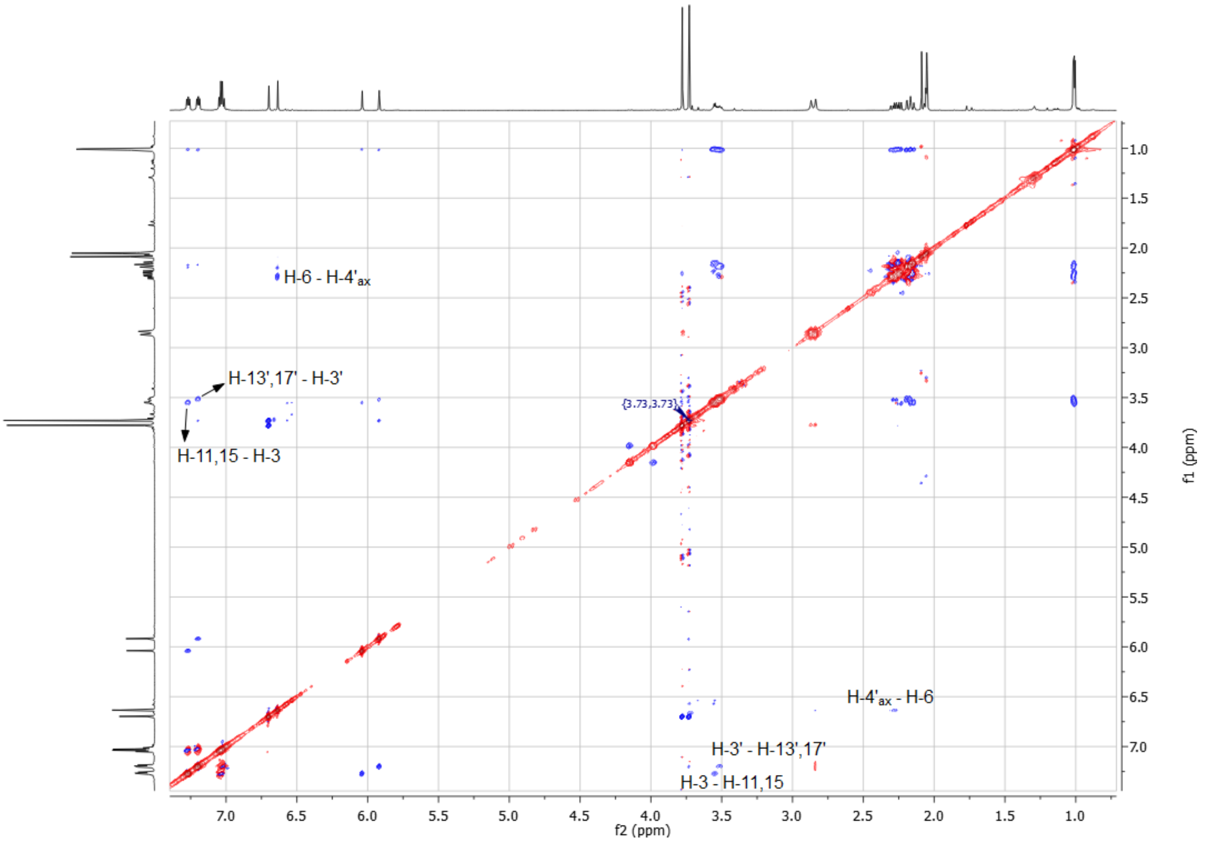

Figure S80. ^{1}H-^{1}H ROESY NMR (700 MHz) spectrum of *trans*,*trans*-(a*S*,1*S*,3*S*,1'*S*,3'*S*)-**20** in acetone-d_6.

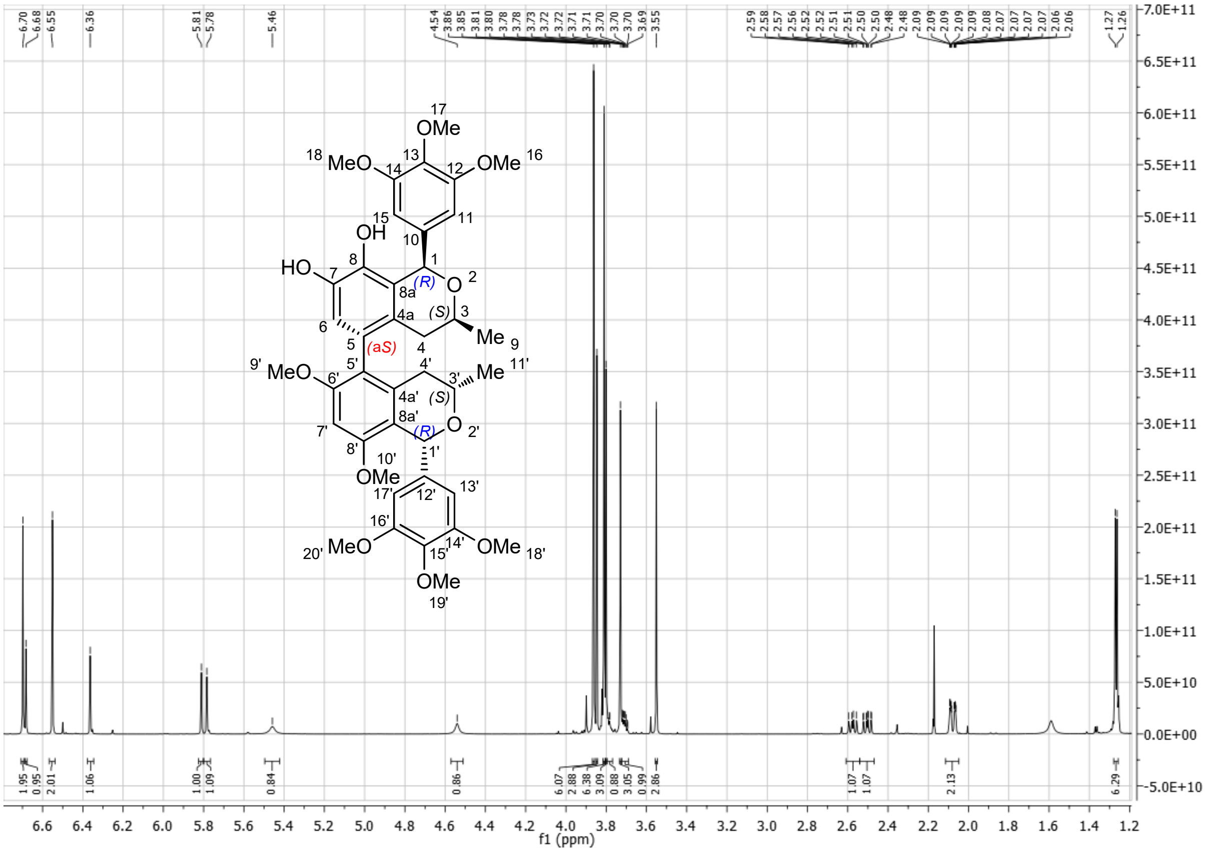

Figure S81. ^{1}H NMR (700 MHz) spectrum of *cis*,*cis*-(a*S*,1*R*,3*S*,1'*R*,3'*S*)-**22** in $CDCl_3$.

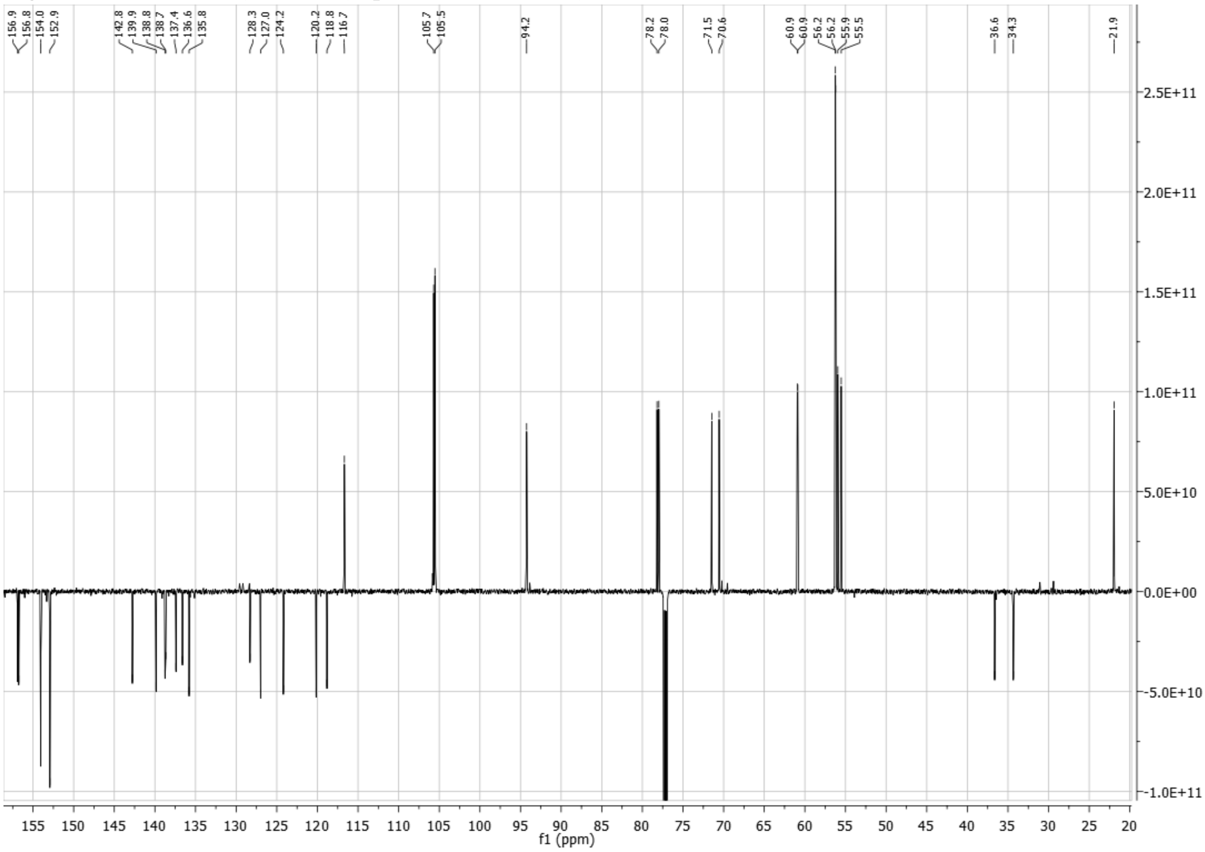

Figure S82. ^{13}C NMR (175 MHz) spectrum of *cis*,*cis*-(a*S*,1*R*,3*S*,1'*R*,3'*S*)-**22** in $CDCl_3$.

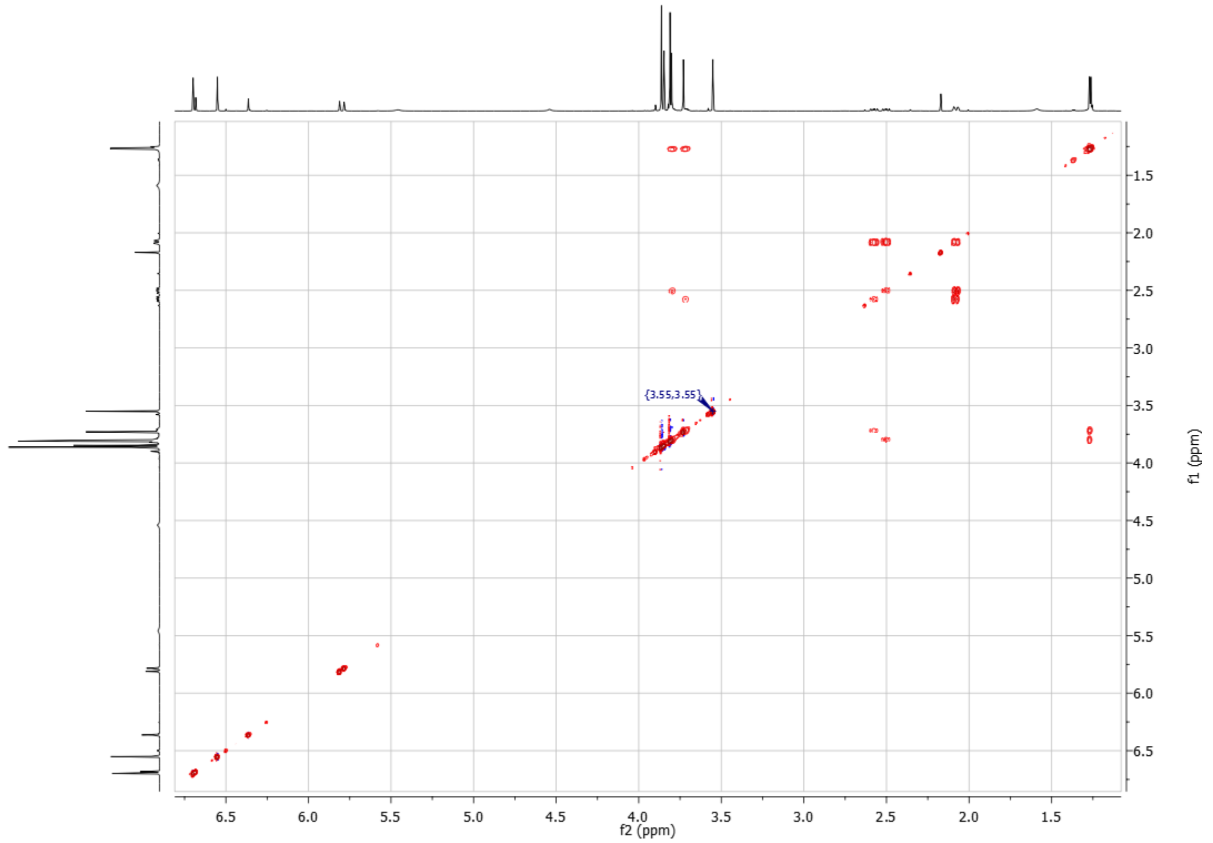

Figure S83. ^{1}H-^{1}H COSY NMR (700 MHz) spectrum of *cis*,*cis*-(a*S*,1*R*,3*S*,1'*R*,3'*S*)-**22** in $CDCl_3$.

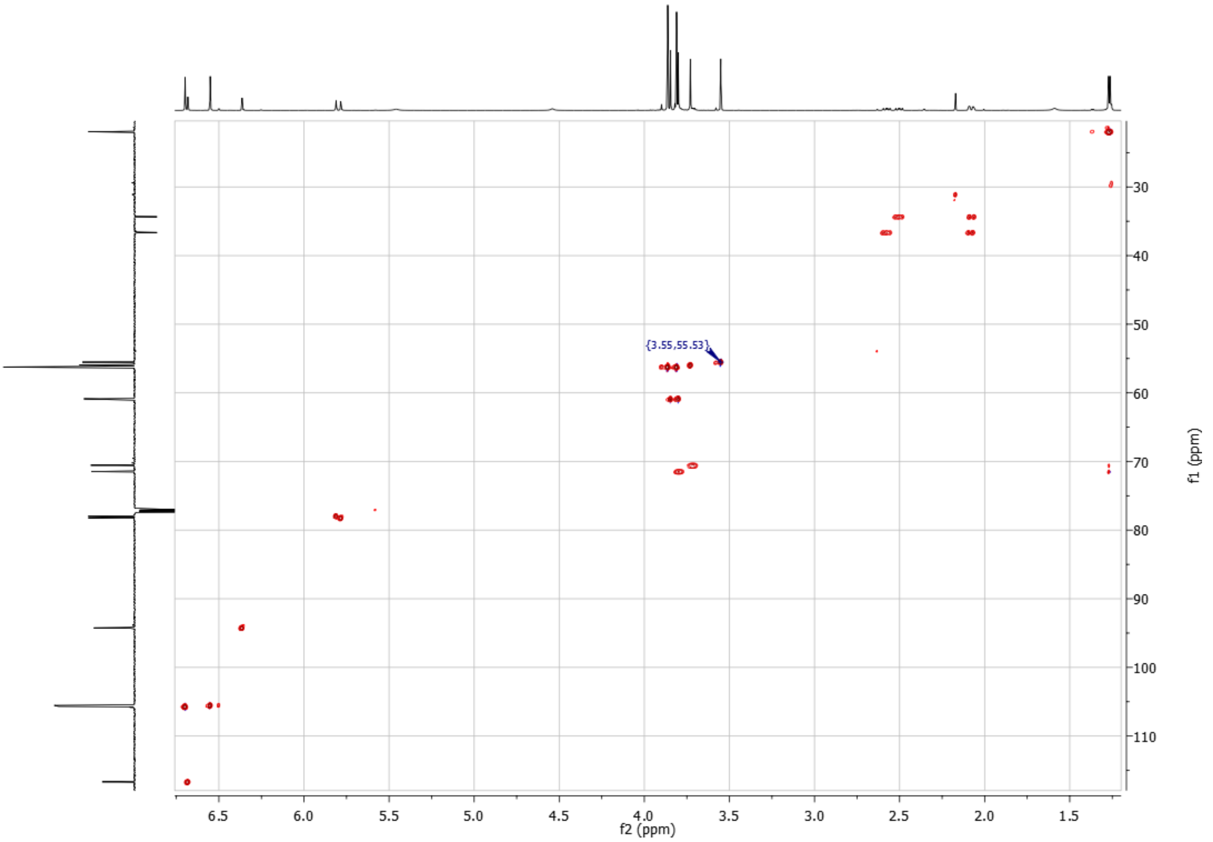

Figure S84. ^{1}H-^{13}C HSQC NMR (700 MHz) spectrum of (*cis*,*cis*-(a*S*,1*R*,3*S*,1'*R*,3'*S*)-**22** in $CDCl_3$.

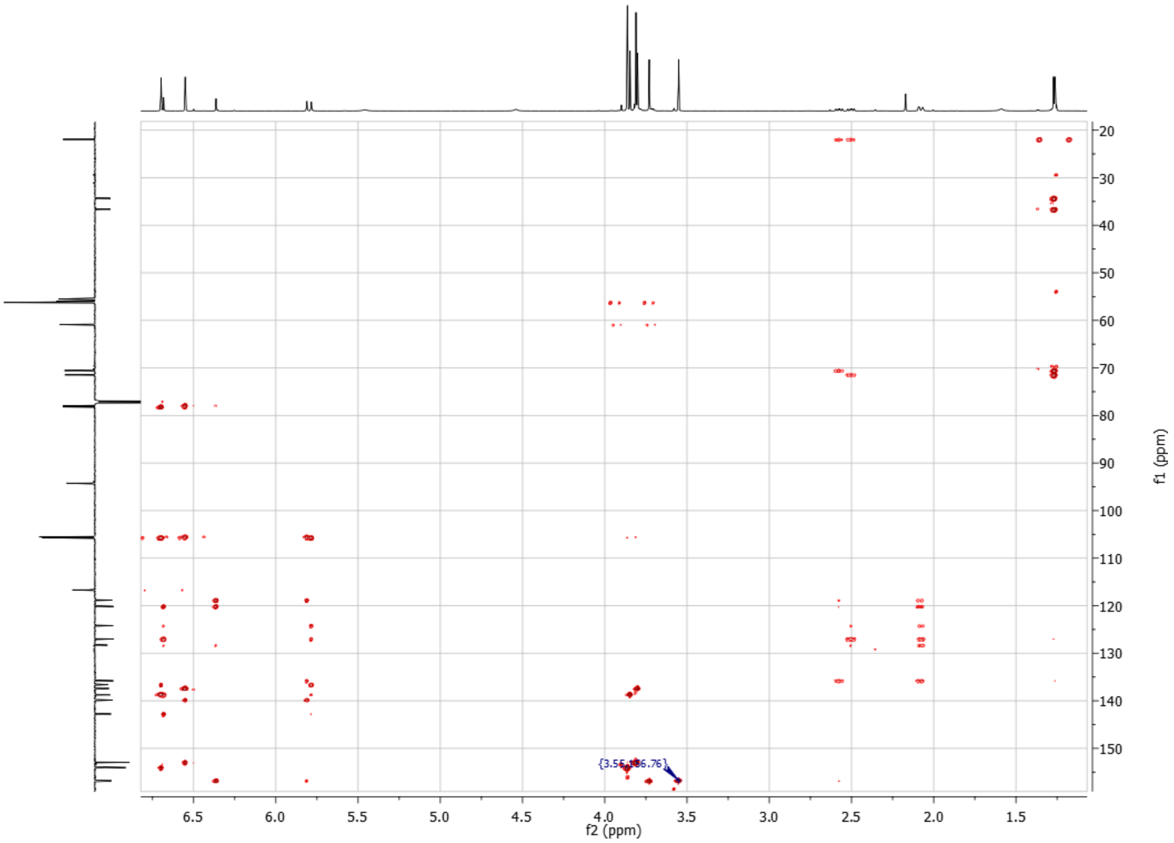

Figure S85. ^{1}H-^{13}C HMBC NMR (700 MHz) spectrum of *cis*,*cis*-(a*S*,1*R*,3*S*,1'*R*,3'*S*)-**22** in $CDCl_3$.

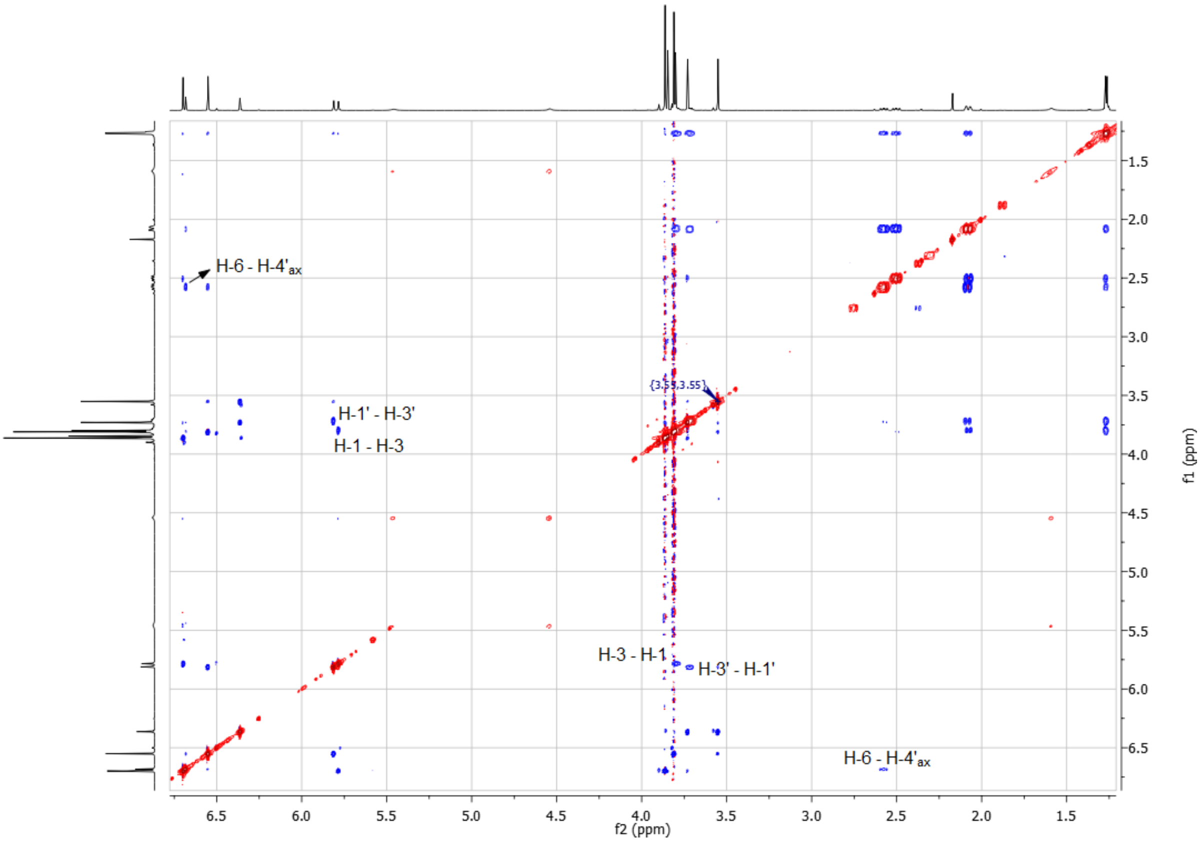

Figure S86. ^{1}H-^{1}H ROESY NMR (700 MHz) spectrum of *cis*,*cis*-(a*S*,1*R*,3*S*,1'*R*,3'*S*)-**22** in $CDCl_3$.

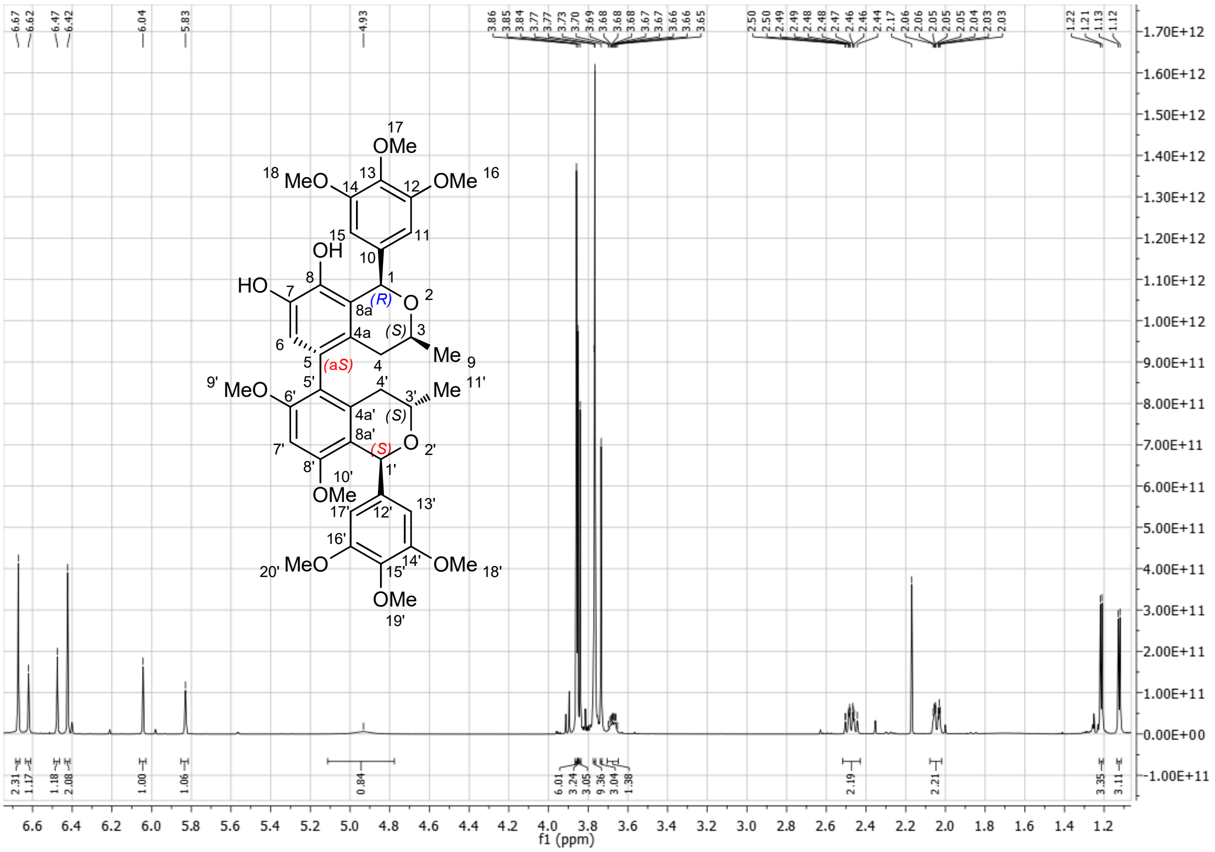

Figure S87. ^{1}H NMR (700 MHz) spectrum of *cis*,*trans*-(a*S*,1*R*,3*S*,1’*S*,3’*S*)-**22** in $CDCl_3$.

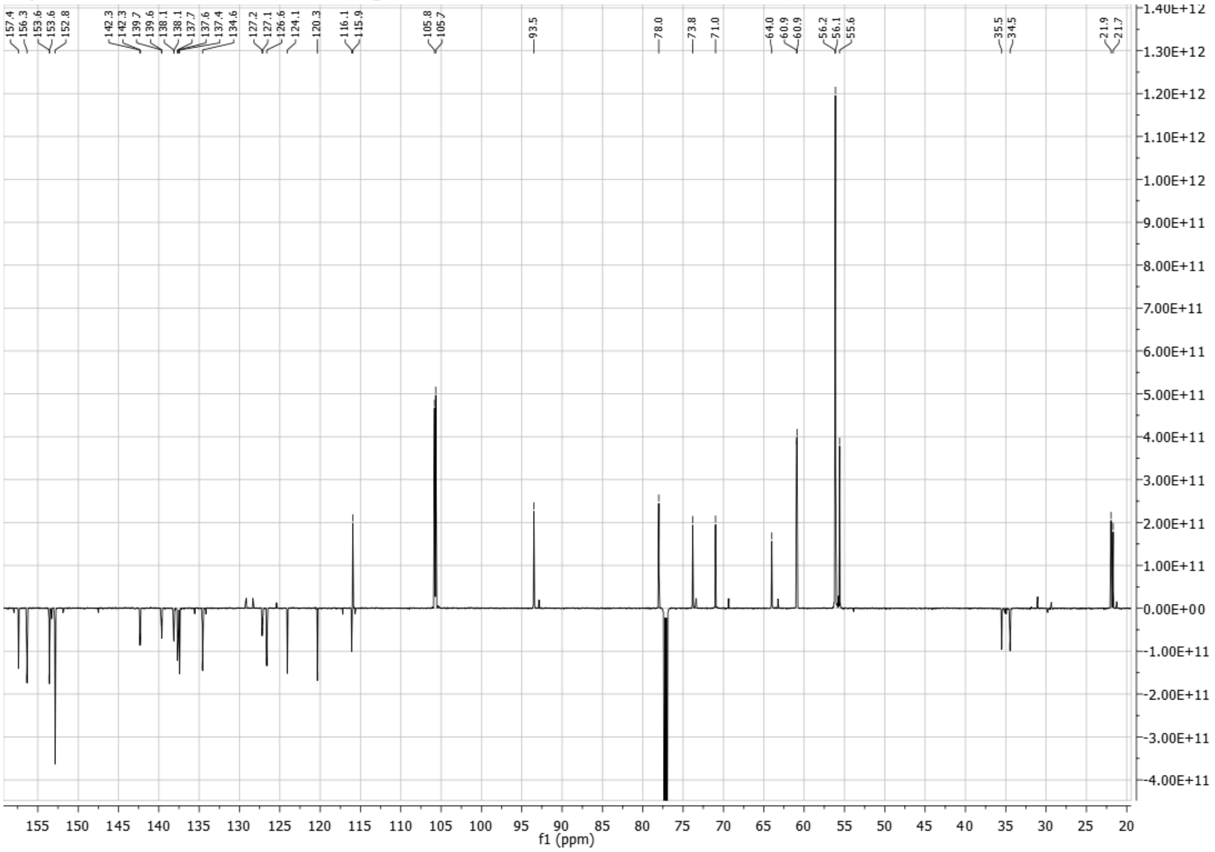

Figure S88. ^{13}C NMR (175 MHz) spectrum of *cis*,*trans*-(a*S*,1*R*,3*S*,1’*S*,3’*S*)-**22** in $CDCl_3$.

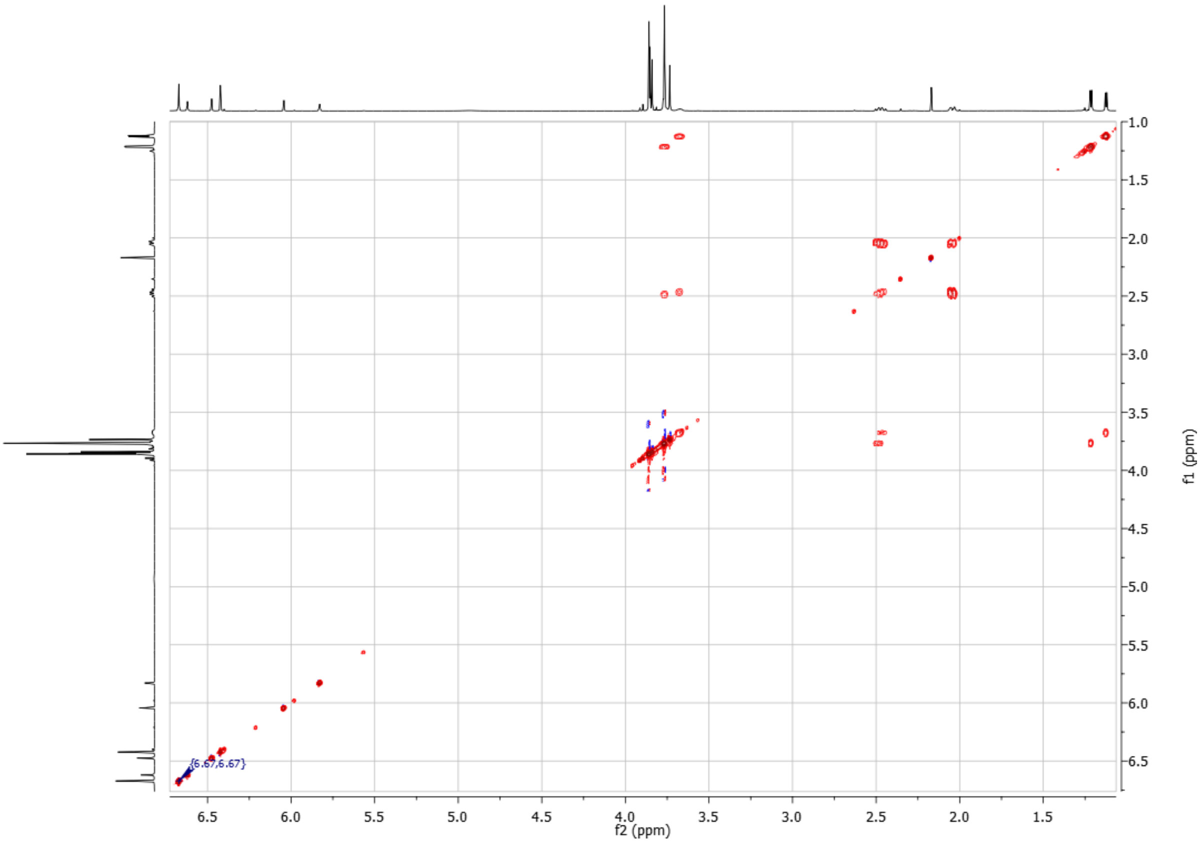

Figure S89. ^{1}H-^{1}H COSY NMR (700 MHz) spectrum of *cis*,*trans*-(a*S*,1*R*,3*S*,1'*S*,3'*S*)-**22** in $CDCl_3$.

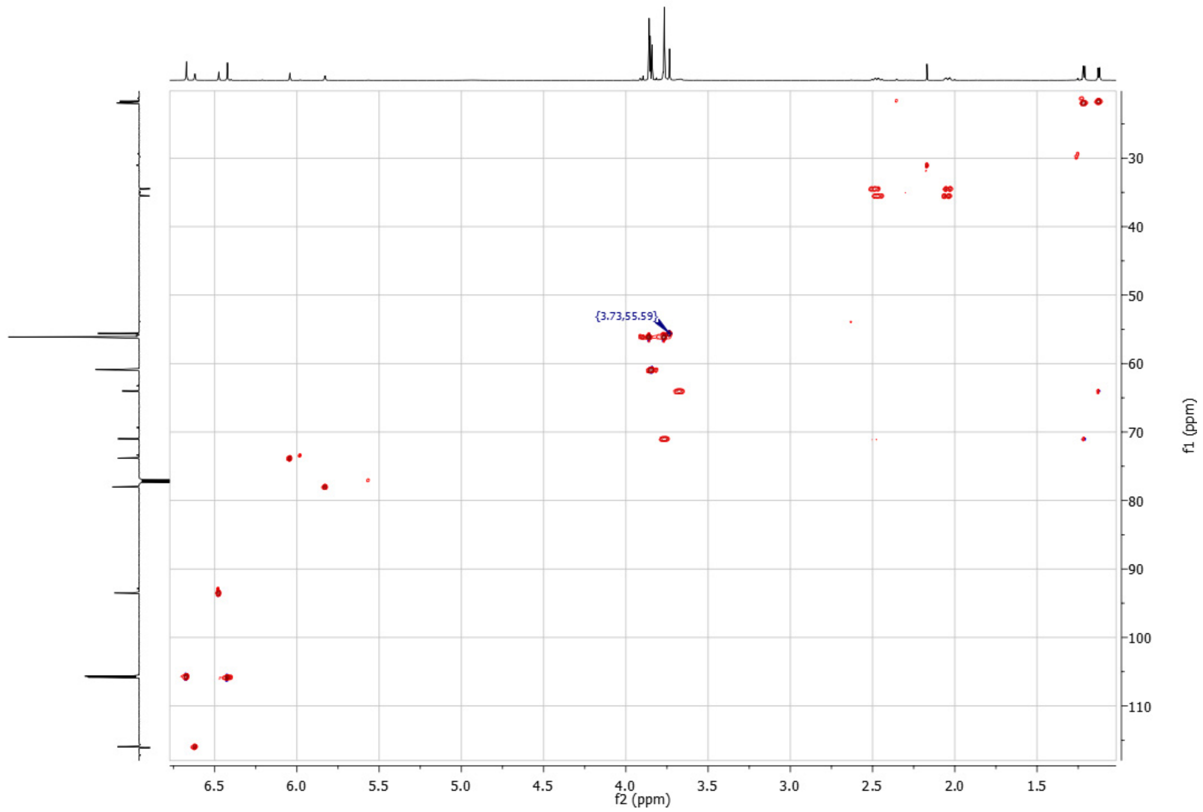

Figure S90. ^{1}H-^{13}C HSQC NMR (700 MHz) spectrum of *cis*,*trans*-(a*S*,1*R*,3*S*,1'*S*,3'*S*)-**22** in $CDCl_3$.

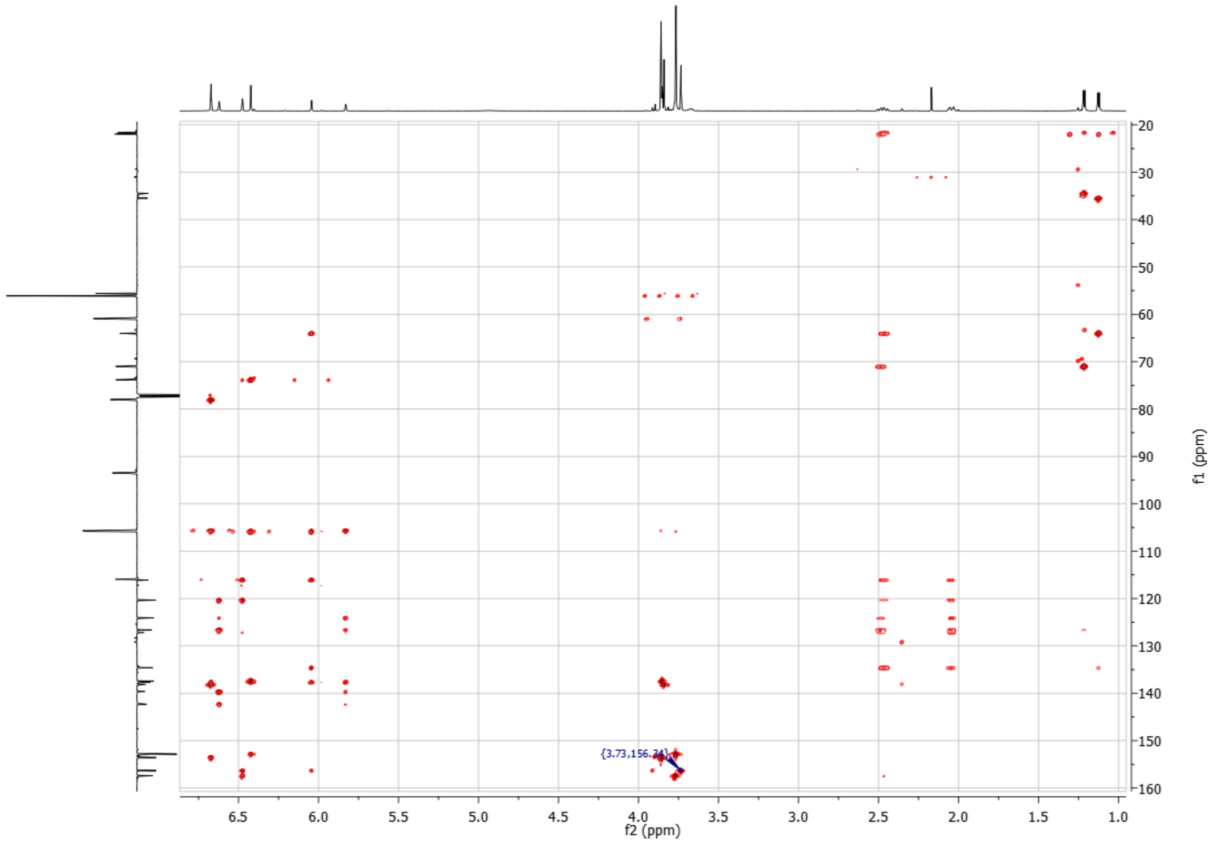

Figure S91. ^{1}H-^{13}C HMBC NMR (700 MHz) spectrum of *cis*,*trans*-(a*S*,1*R*,3*S*,1'*S*,3'*S*)-**22** in $CDCl_3$.

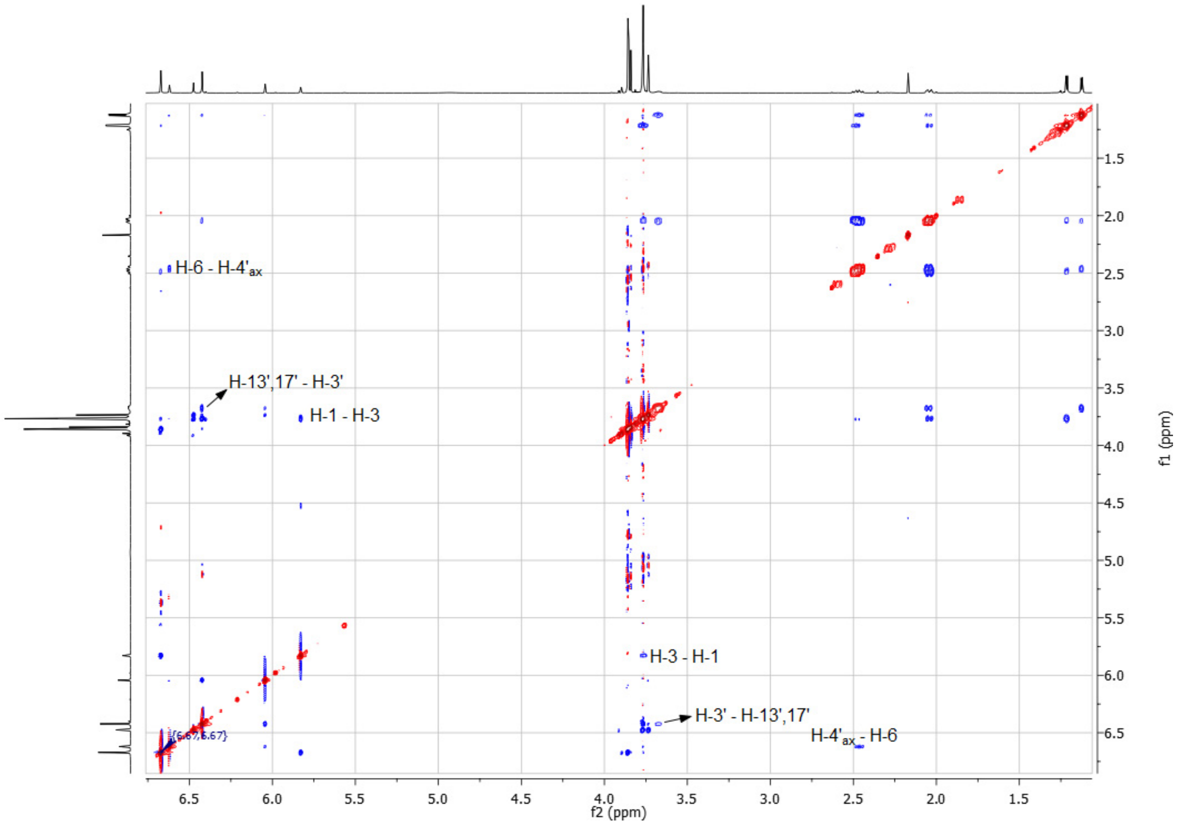

Figure S92. ^{1}H-^{1}H ROESY NMR (700 MHz) spectrum of *cis*,*trans*-(a*S*,1*R*,3*S*,1'*S*,3'*S*)-**22** in $CDCl_3$.

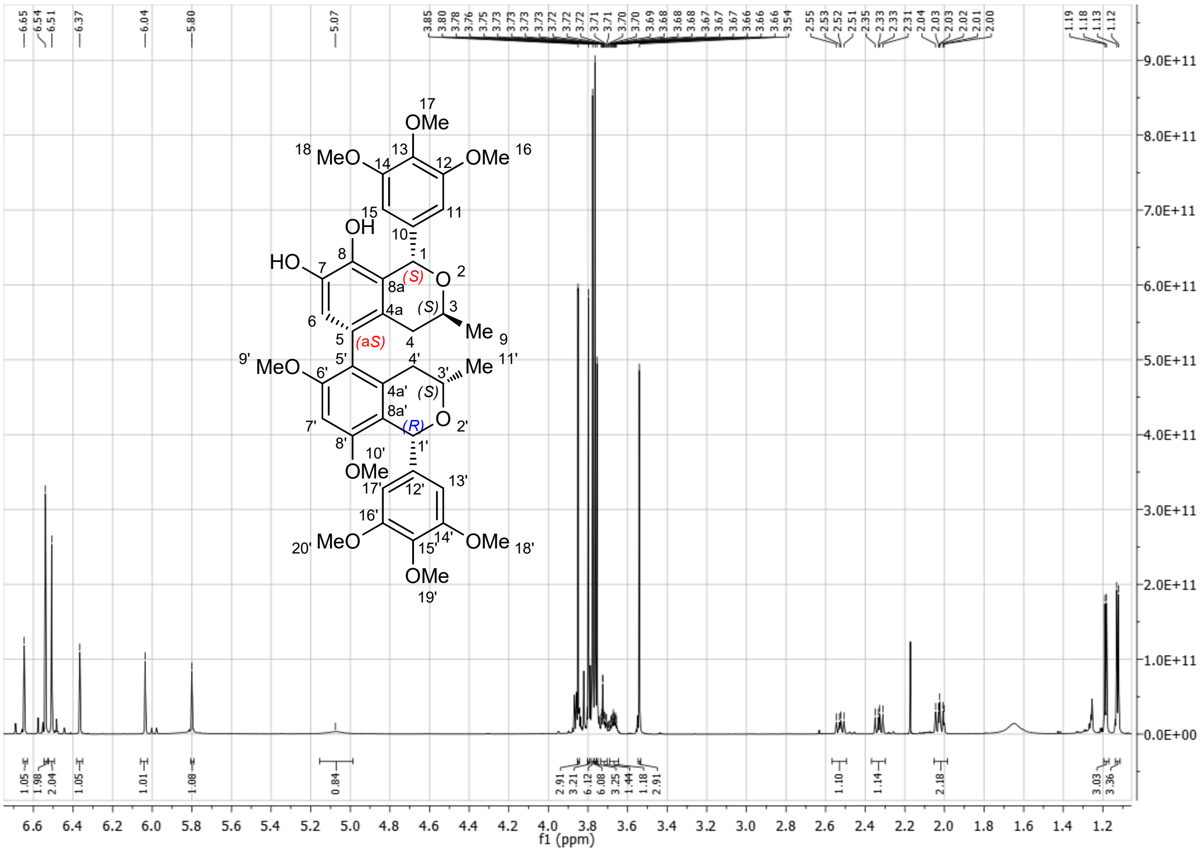

Figure S93. ^{1}H NMR (700 MHz) spectrum of *trans*,*cis*-(a*S*,1*S*,3*S*,1'*R*,3'*S*)-**22** in $CDCl_3$.

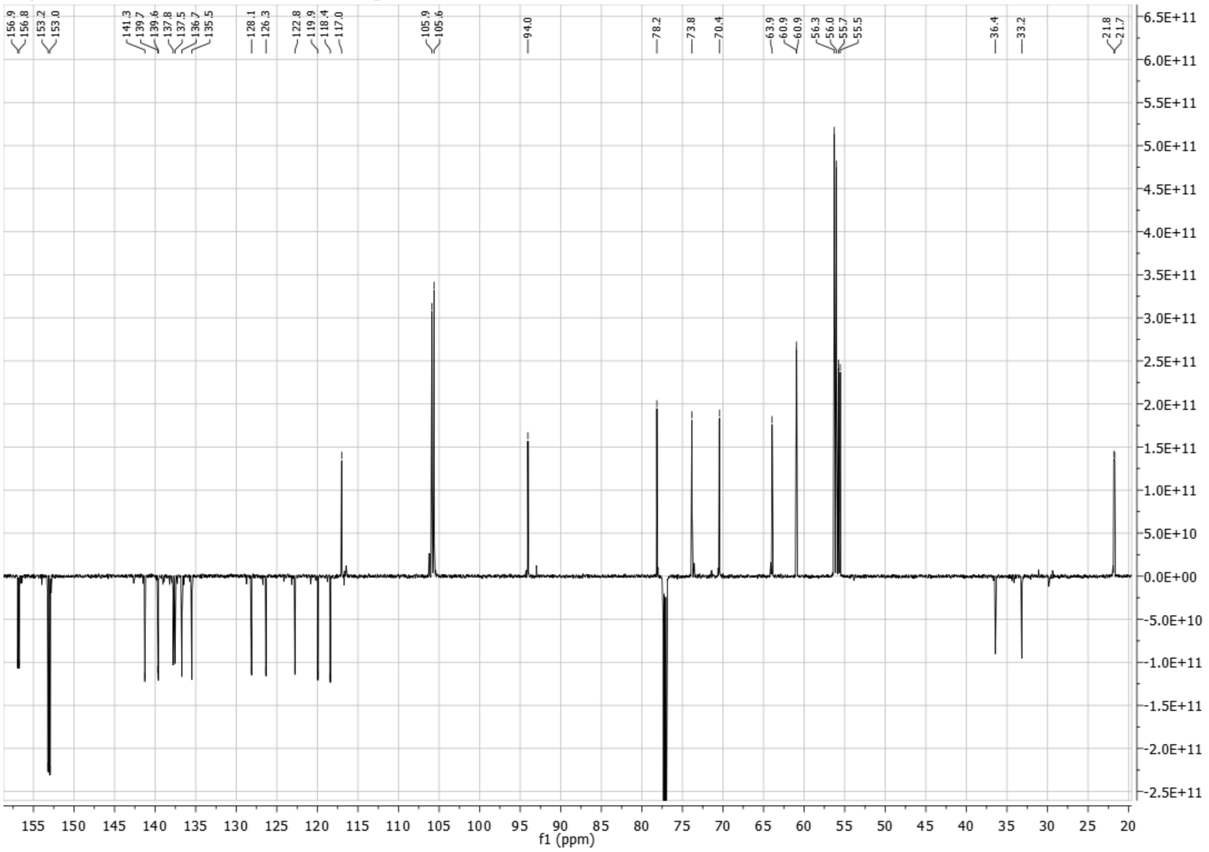

Figure S94. ^{13}C NMR (175 MHz) spectrum of *trans*,*cis*-(a*S*,1*S*,3*S*,1'*R*,3'*S*)-**22** in $CDCl_3$.

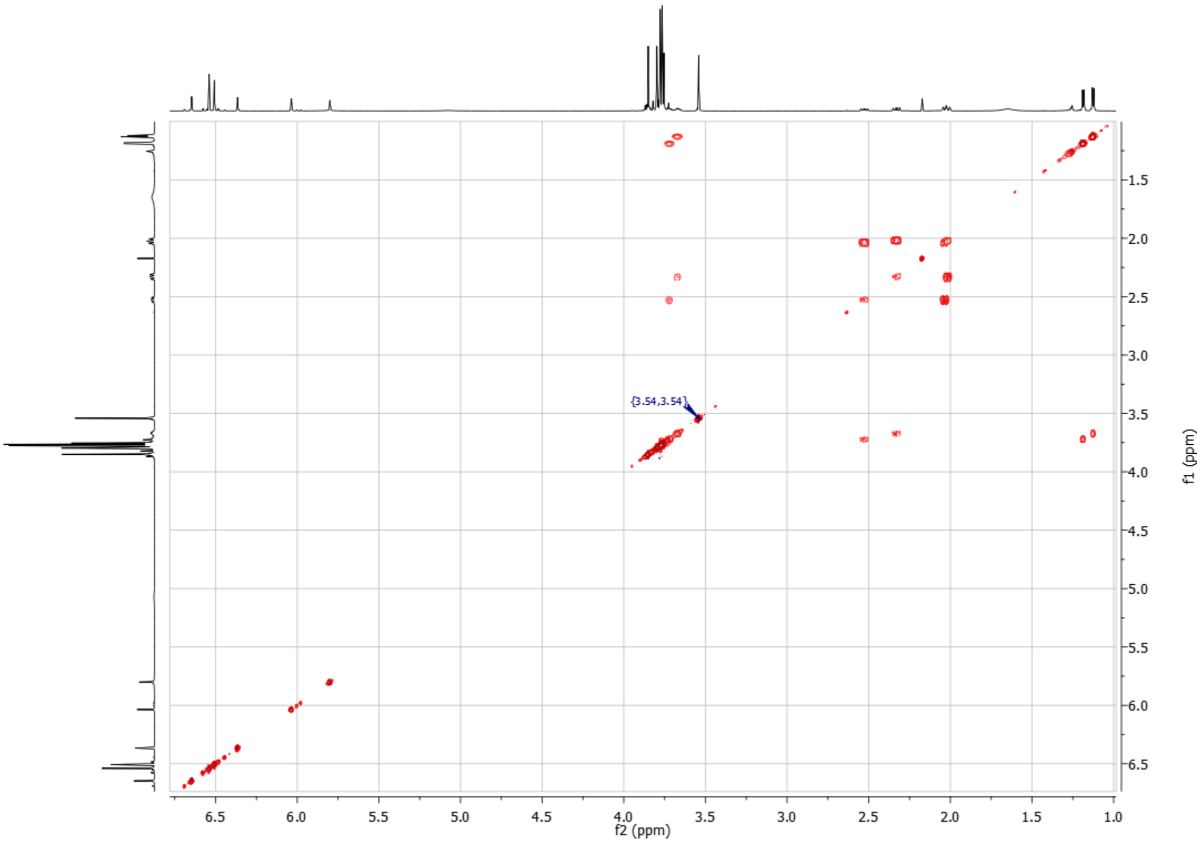

Figure S95. ^{1}H-^{1}H COSY NMR (700 MHz) spectrum of *trans*,*cis*-(a*S*,1*S*,3*S*,1’*R*,3’*S*)-**22** in $CDCl_3$.

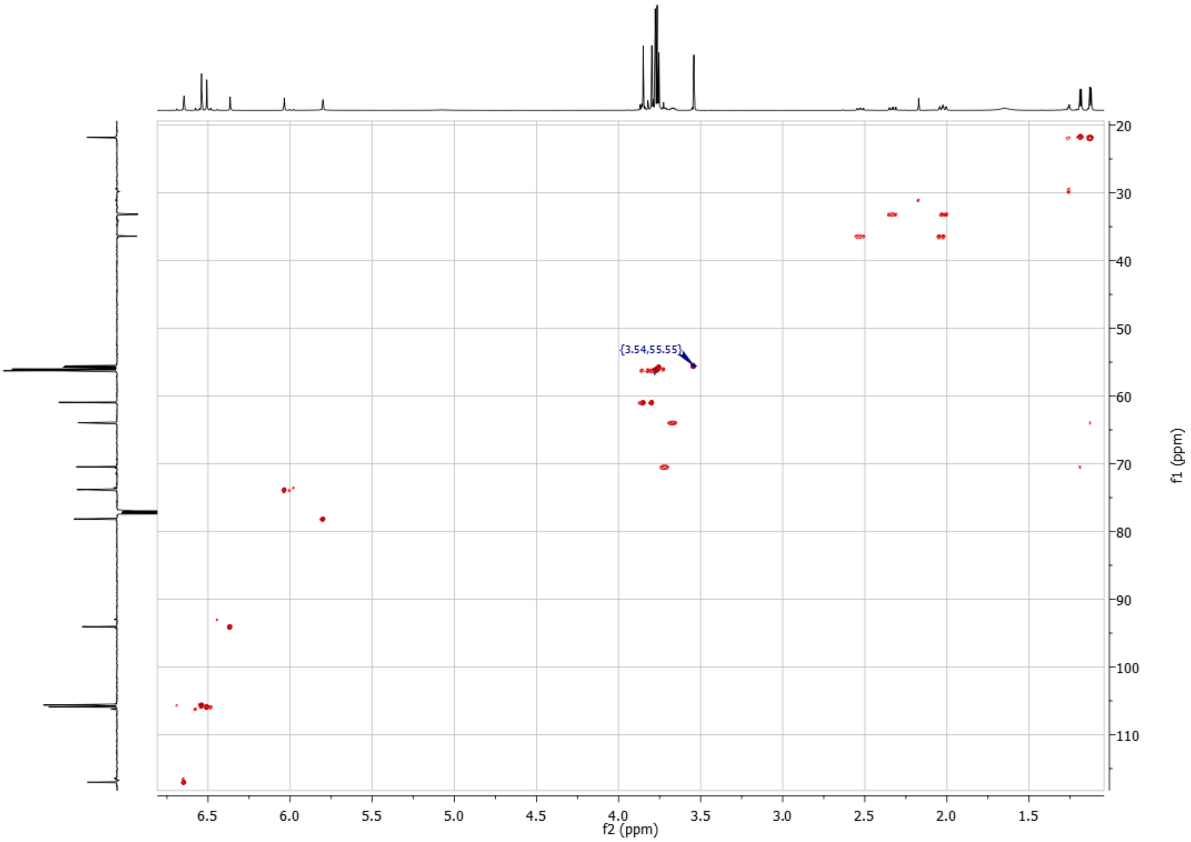

Figure S96. ^{1}H-^{13}C HSQC NMR (700 MHz) spectrum of *trans*,*cis*-(a*S*,1*S*,3*S*,1’*R*,3’*S*)-**22** in $CDCl_3$.

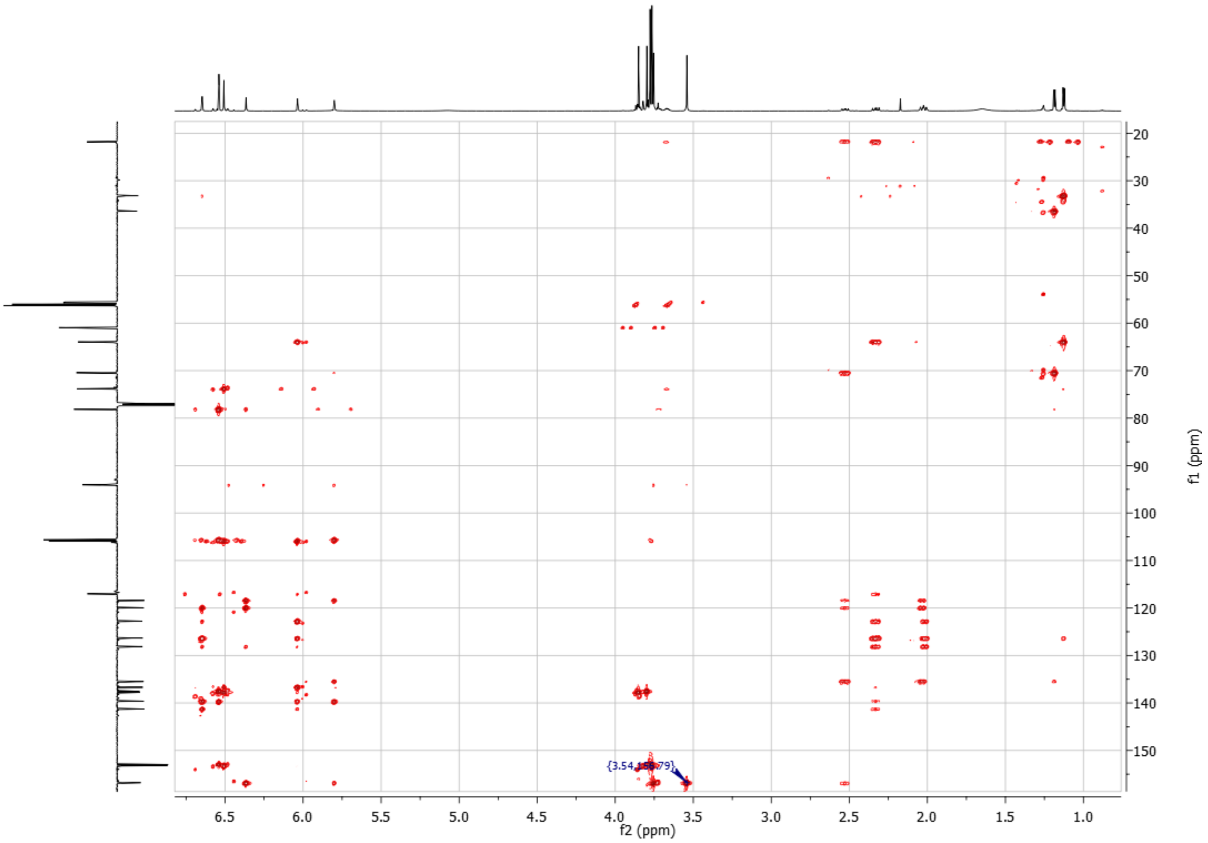

Figure S97. ^{1}H-^{13}C HMBC NMR (700 MHz) spectrum of *trans*,*cis*-(a*S*,1*S*,3*S*,1'*R*,3'*S*)-**22** in $CDCl_3$.

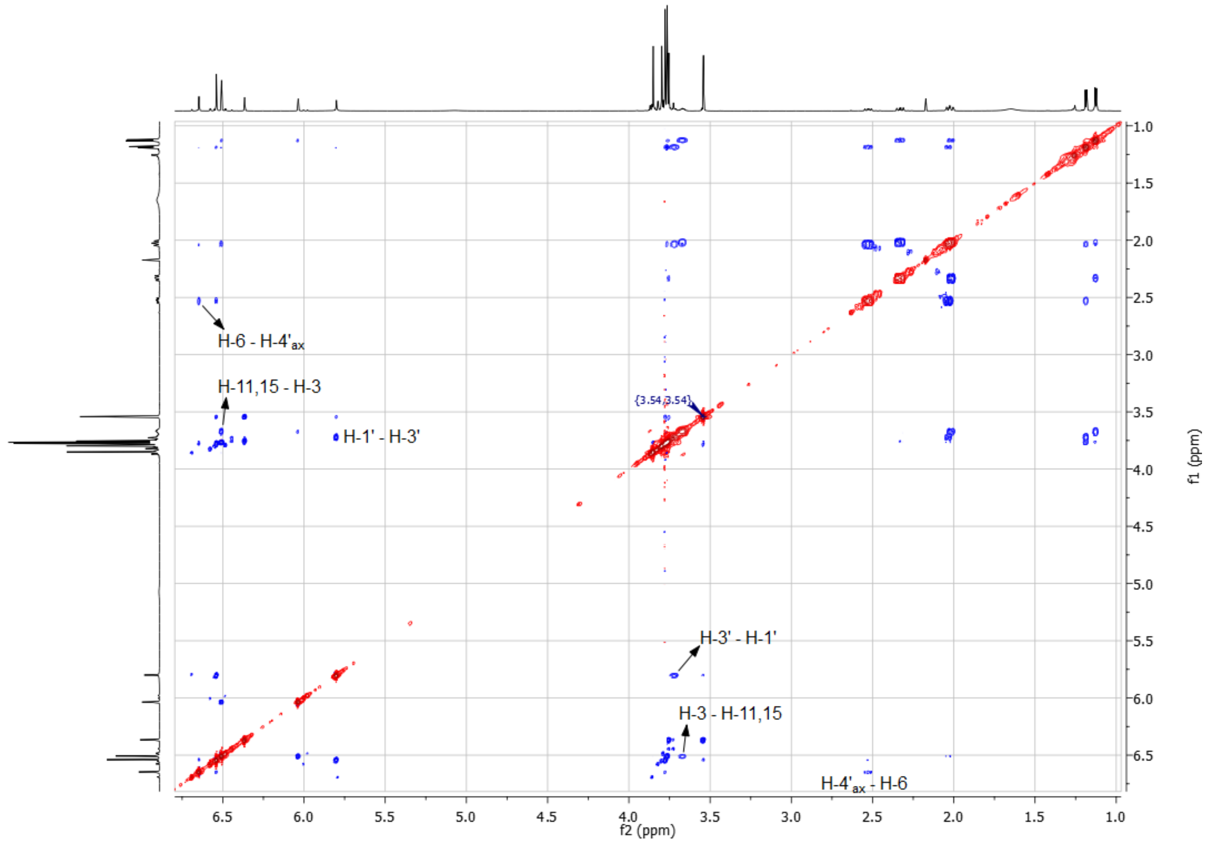

Figure S98. ^{1}H-^{1}H ROESY NMR (700 MHz) spectrum of *trans*,*cis*-(a*S*,1*S*,3*S*,1'*R*,3'*S*)-**22** in $CDCl_3$.

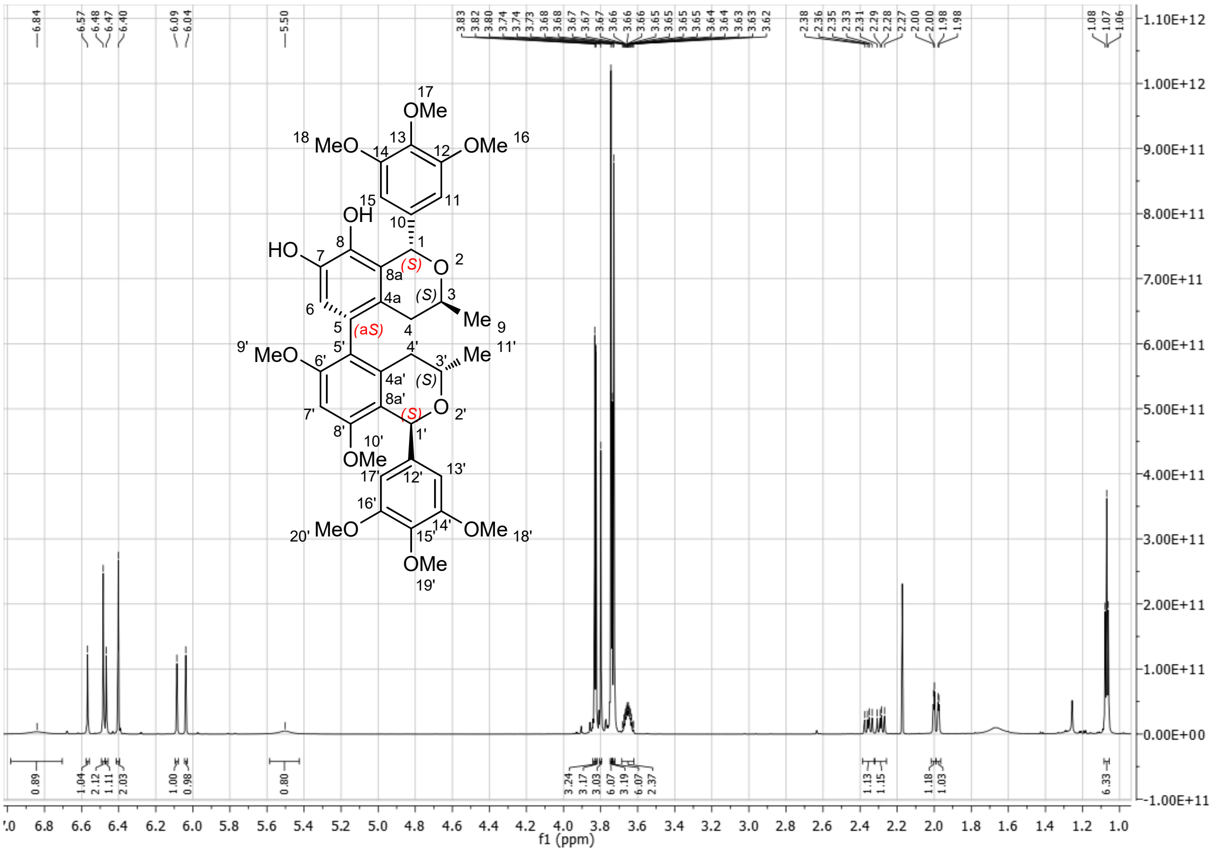

Figure S99. ^{1}H NMR (700 MHz) spectrum of *trans*,*trans*-(a*S*,1*S*,3*S*,1'*S*,3'*S*)-**22** in $CDCl_3$.

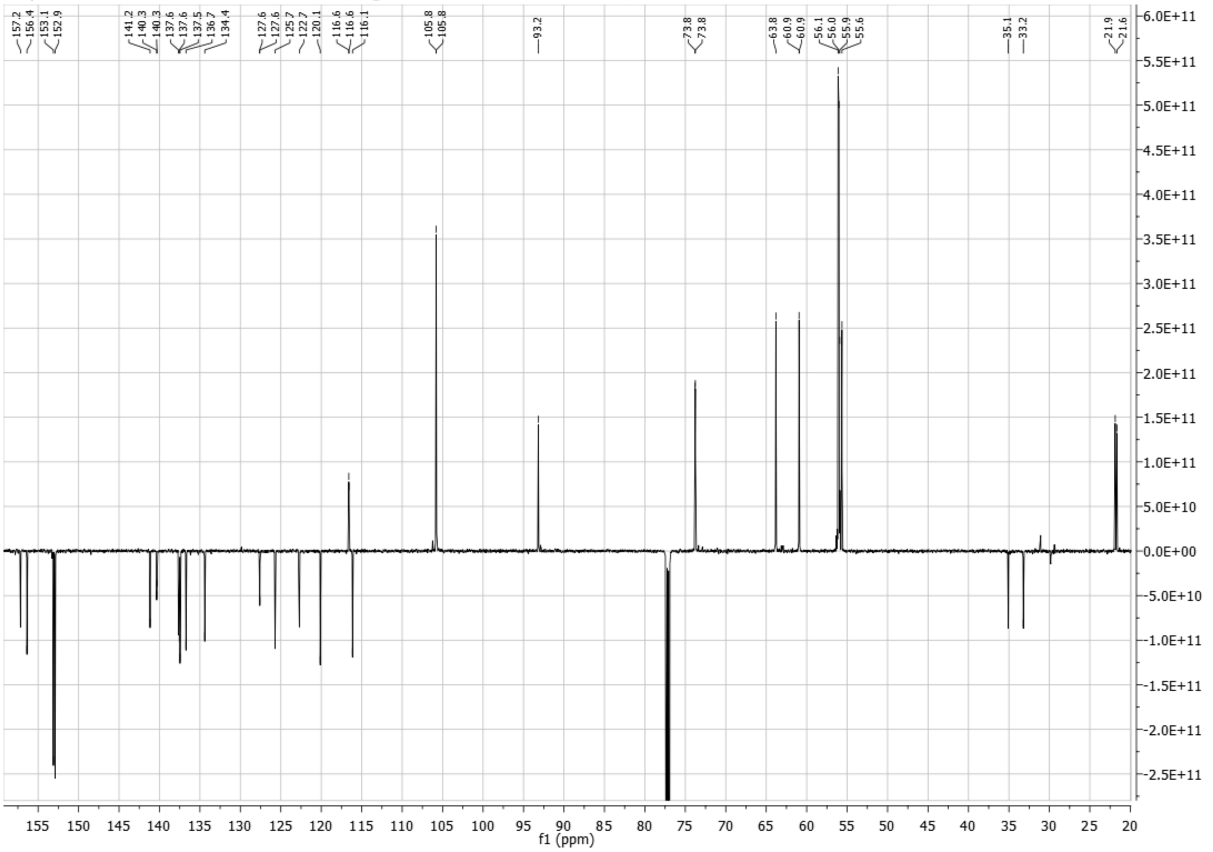

Figure S100. ^{13}C NMR (175 MHz) spectrum of *trans*,*trans*-(a*S*,1*S*,3*S*,1'*S*,3'*S*)-**22** in $CDCl_3$.

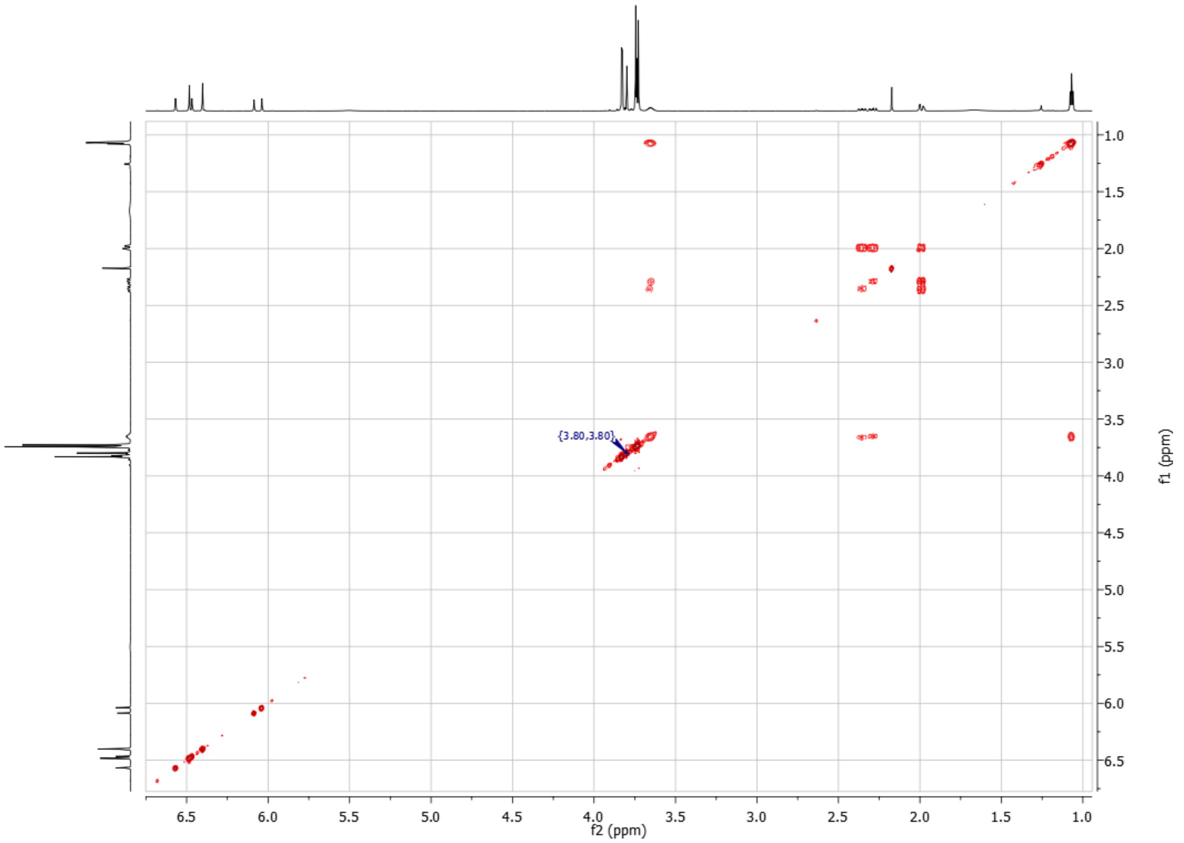

Figure S101. ^{1}H-^{1}H COSY NMR (700 MHz) spectrum of *trans*,*trans*-(a*S*,1*S*,3*S*,1'*S*,3'*S*)-**22** in $CDCl_3$.

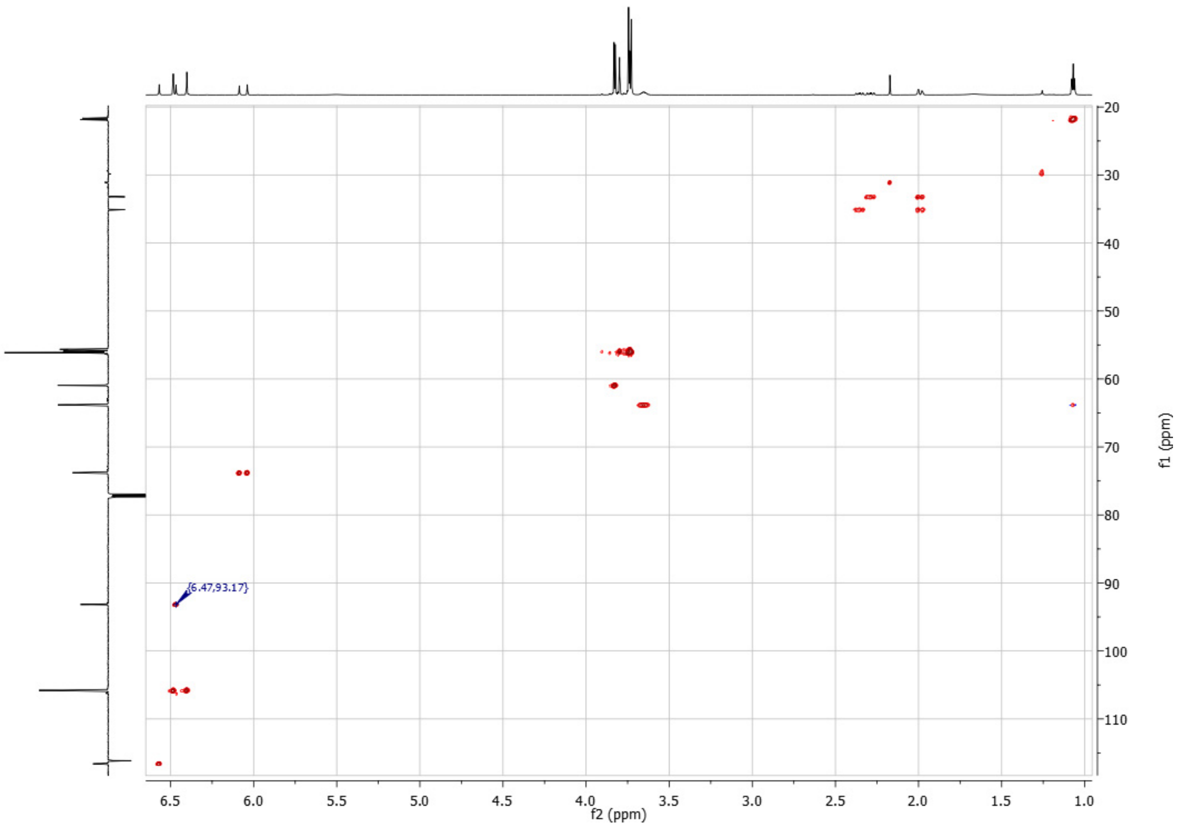

Figure S102. ^{1}H-^{13}C HSQC NMR (700 MHz) spectrum of *trans*,*trans*-(a*S*,1*S*,3*S*,1'*S*,3'*S*)-**22** in $CDCl_3$.

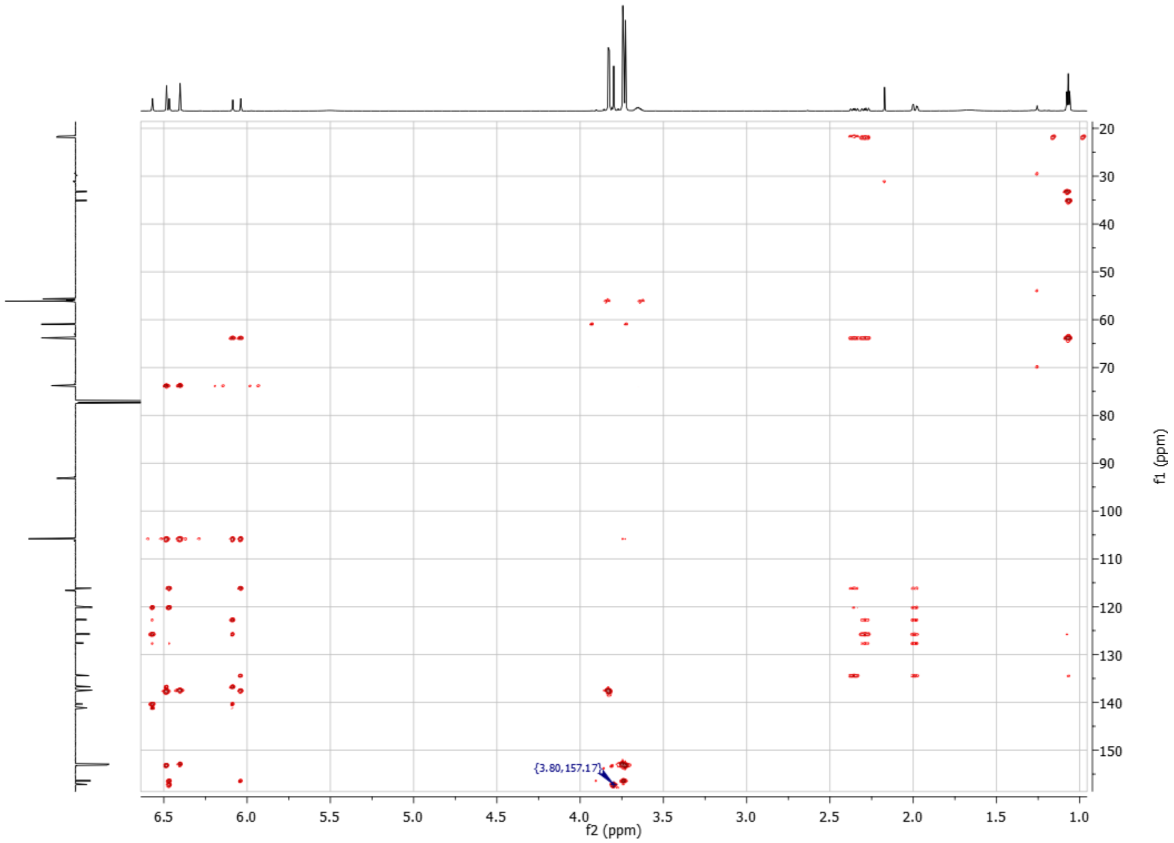

Figure S103. ^{1}H-^{13}C HMBC NMR (700 MHz) spectrum of *trans*,*trans*-(a*S*,1*S*,3*S*,1'*S*,3'*S*)-**22** in $CDCl_3$.

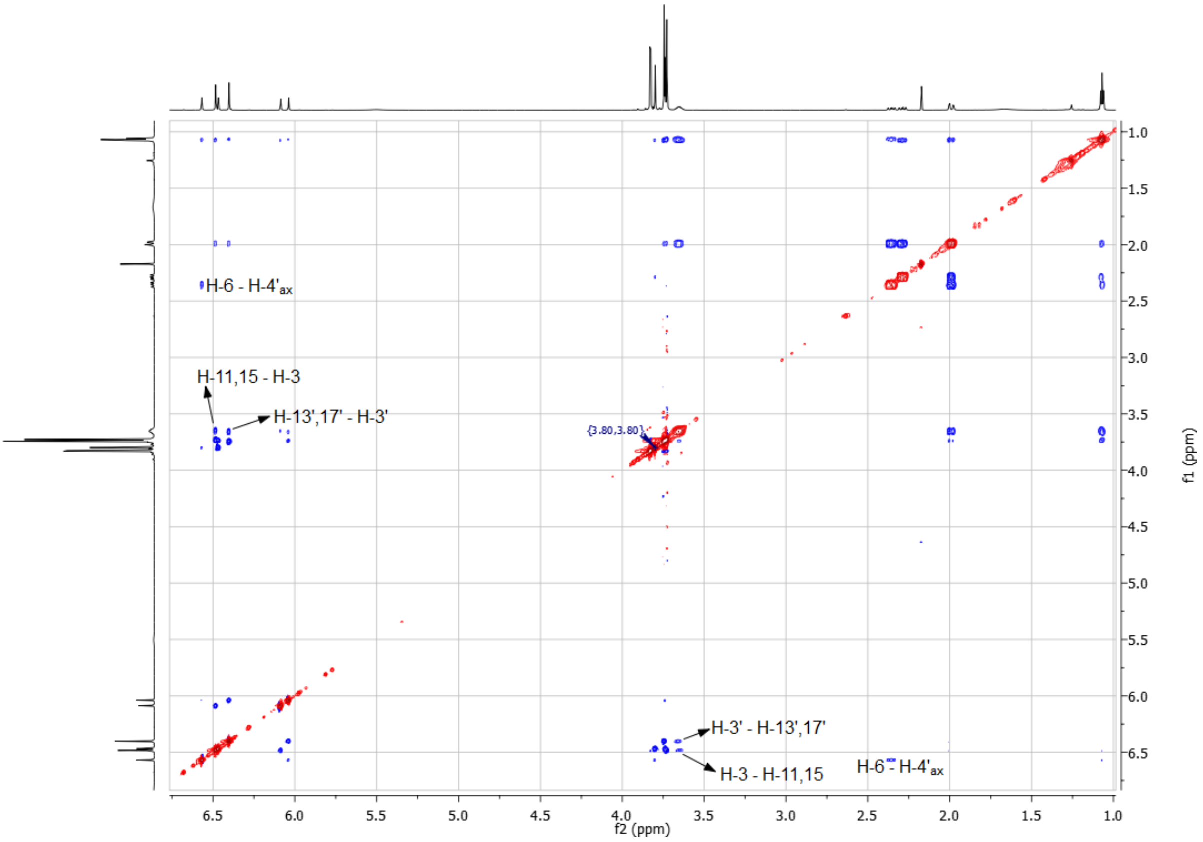

Figure S104. ^{1}H-^{1}H ROESY NMR (700 MHz) spectrum of *trans*,*trans*-(a*S*,1*S*,3*S*,1'*S*,3'*S*)-**22** in $CDCl_3$.

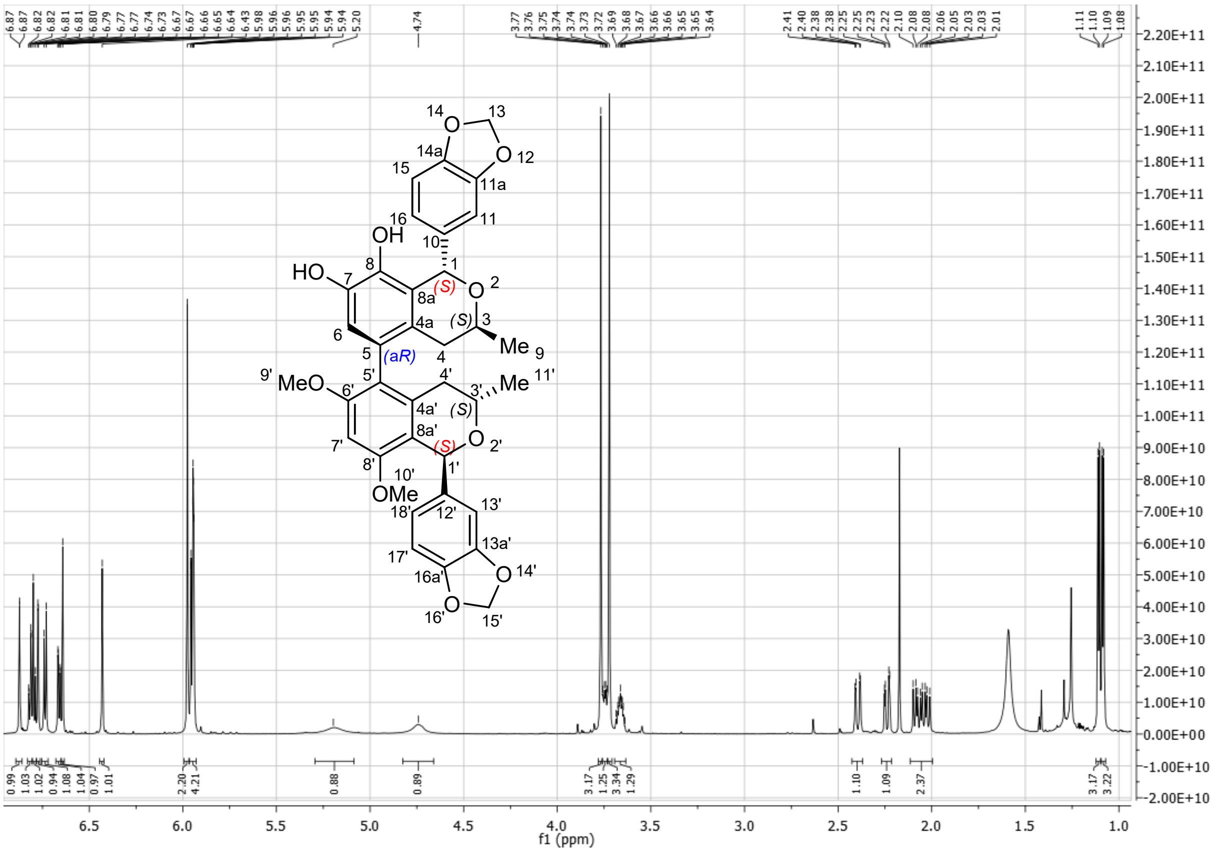

Figure S105. ^{1}H NMR (700 MHz) spectrum of *trans*,*trans*-(a*R*,1*S*,3*S*,1'*S*,3'*S*)-**23** in $CDCl_3$.

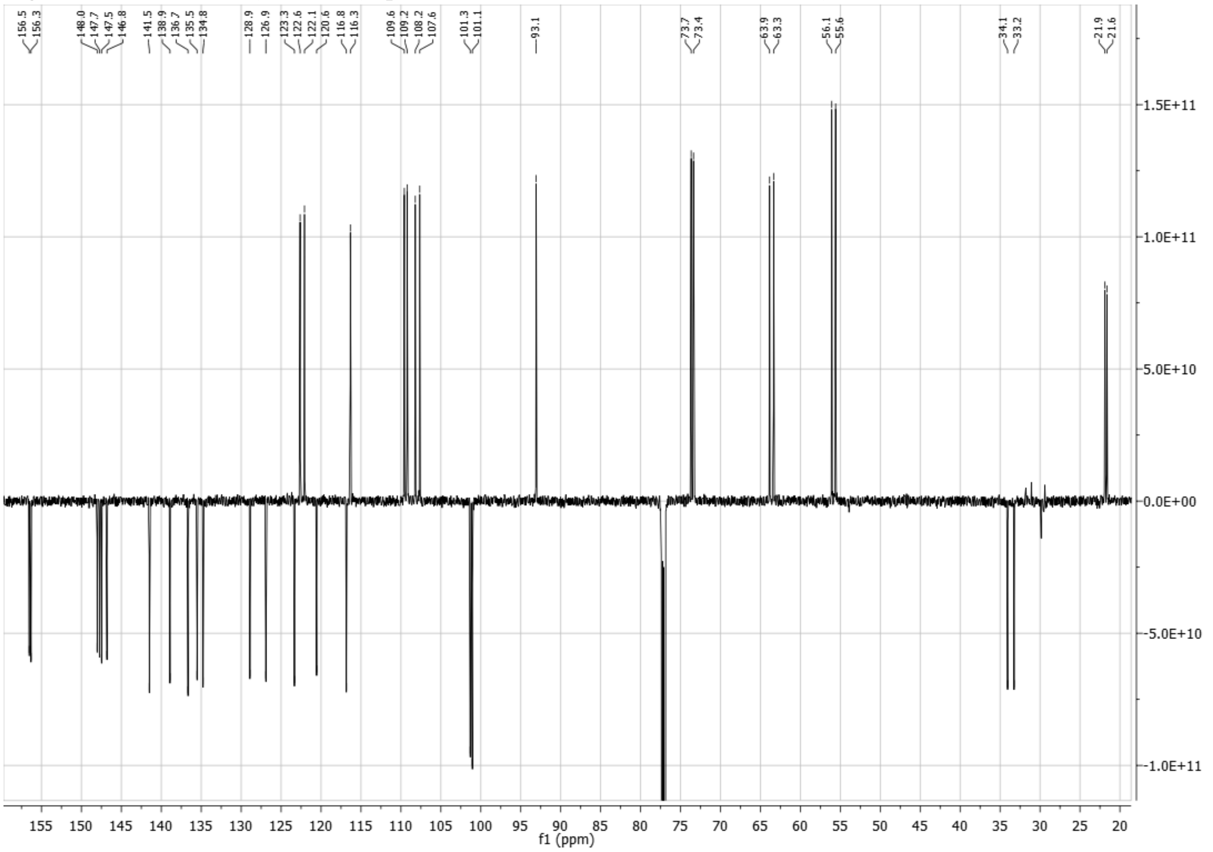

Figure S106. ^{13}C NMR (175 MHz) spectrum of *trans*,*trans*-(a*R*,1*S*,3*S*,1'*S*,3'*S*)-**23** in $CDCl_3$.

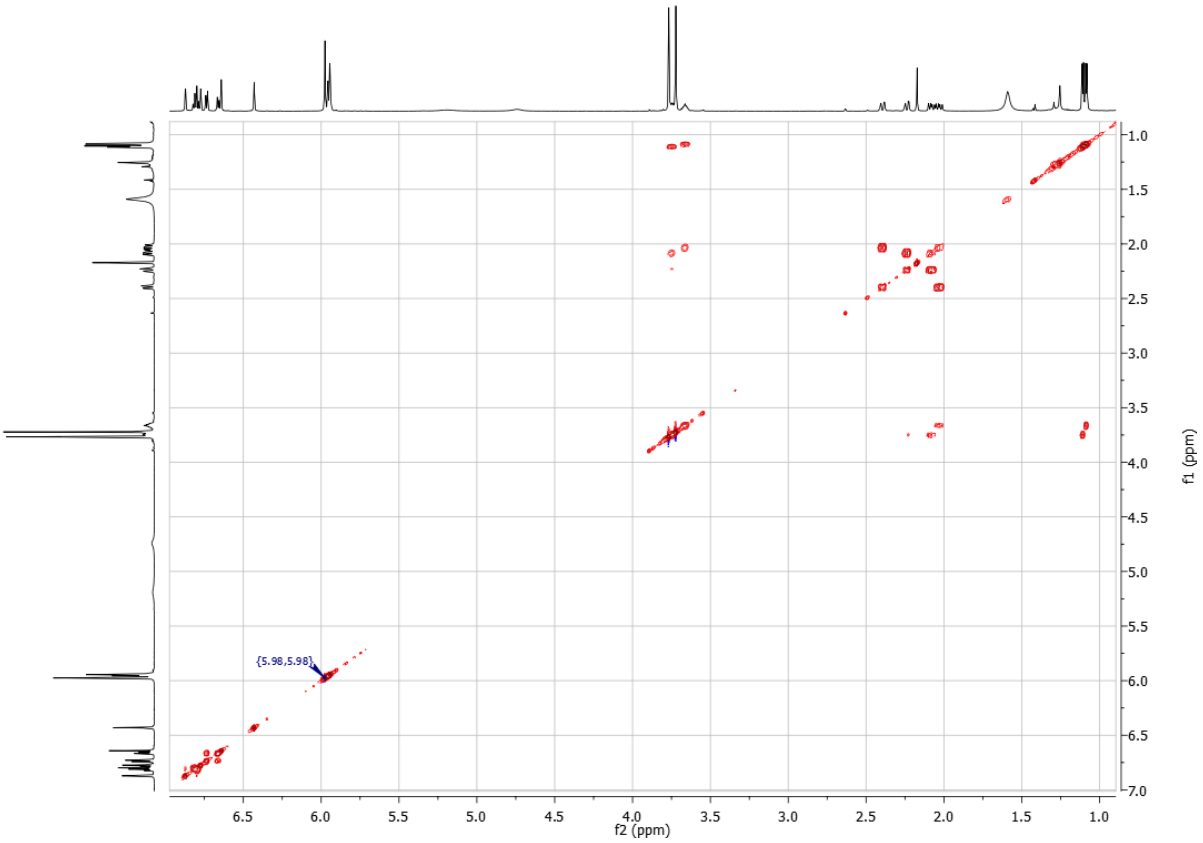

Figure S107. ^{1}H-^{1}H COSY NMR (700 MHz) spectrum of *trans*,*trans*-(a*R*,1*S*,3*S*,1'*S*,3'*S*)-**23** in $CDCl_3$.

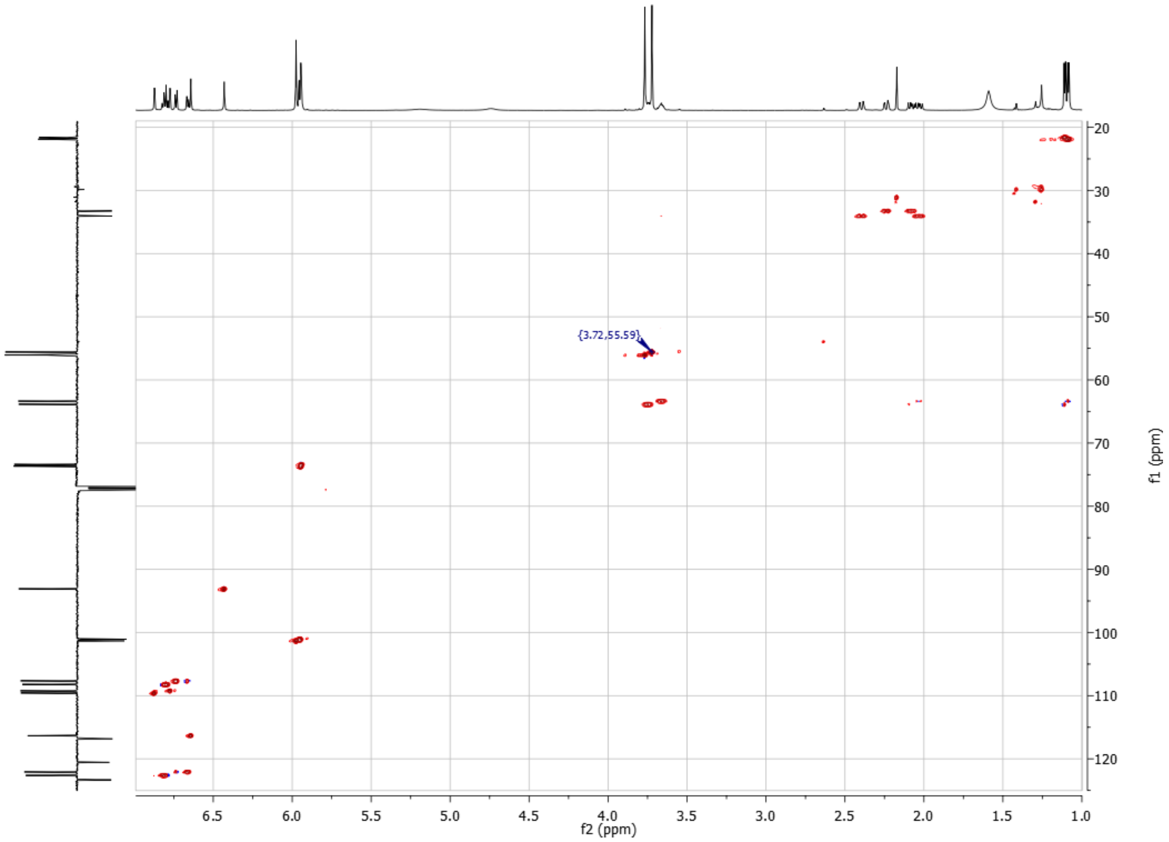

Figure S108. ^{1}H-^{13}C HSQC NMR (700 MHz) spectrum of *trans*,*trans*-(a*R*,1*S*,3*S*,1'*S*,3'*S*)-**23** in $CDCl_3$.

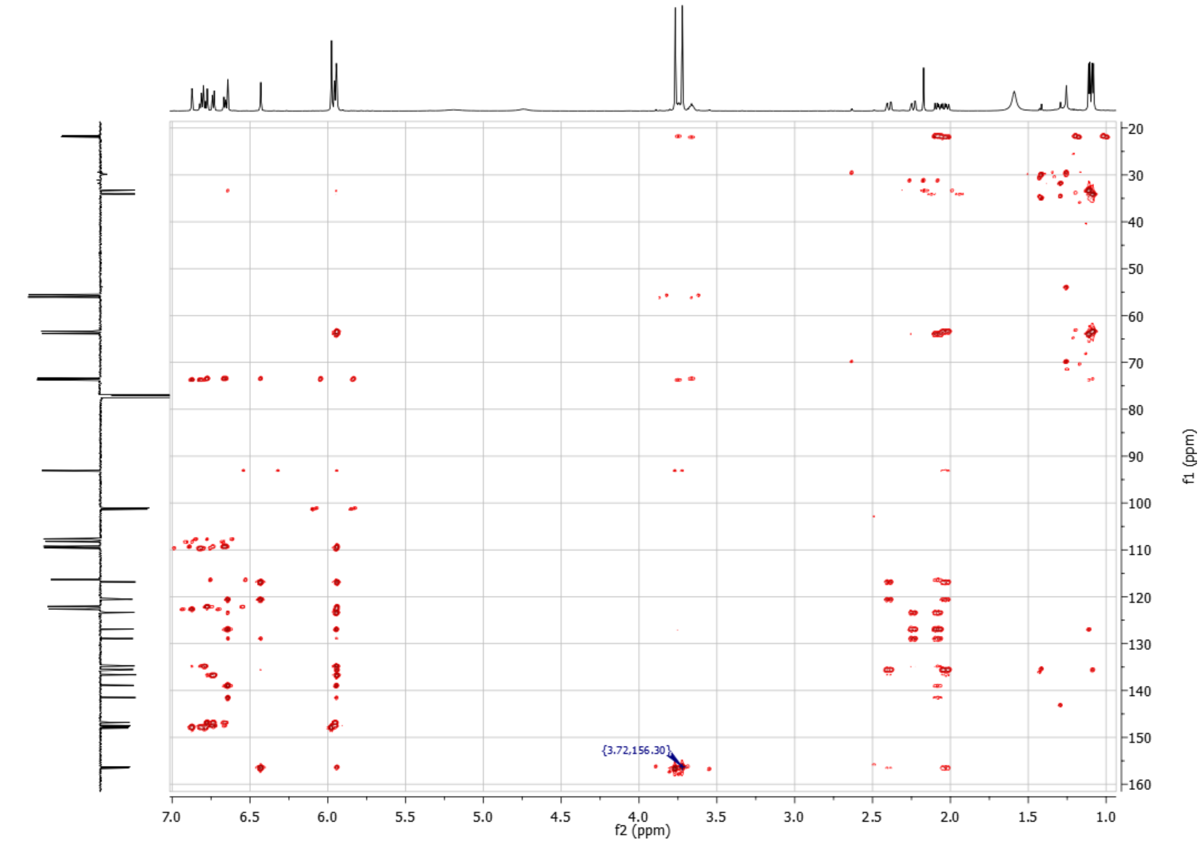

Figure S109. ^{1}H-^{13}C HMBC NMR (700 MHz) spectrum of *trans*,*trans*-(a*R*,1*S*,3*S*,1'*S*,3'*S*)-**23** in $CDCl_3$.

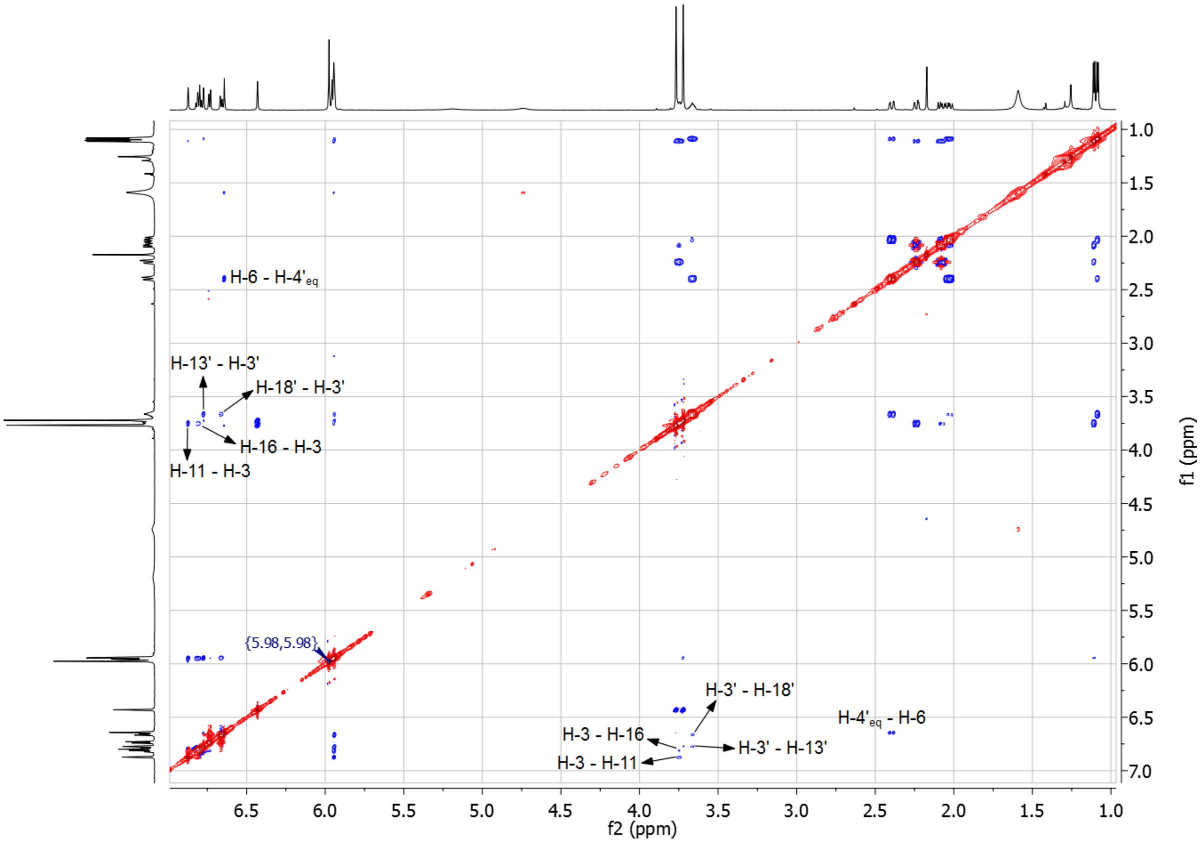

Figure S110. ^{1}H-^{1}H ROESY NMR (700 MHz) spectrum of *trans*,*trans*-(a*R*,1*S*,3*S*,1'*S*,3'*S*)-**23** in $CDCl_3$.

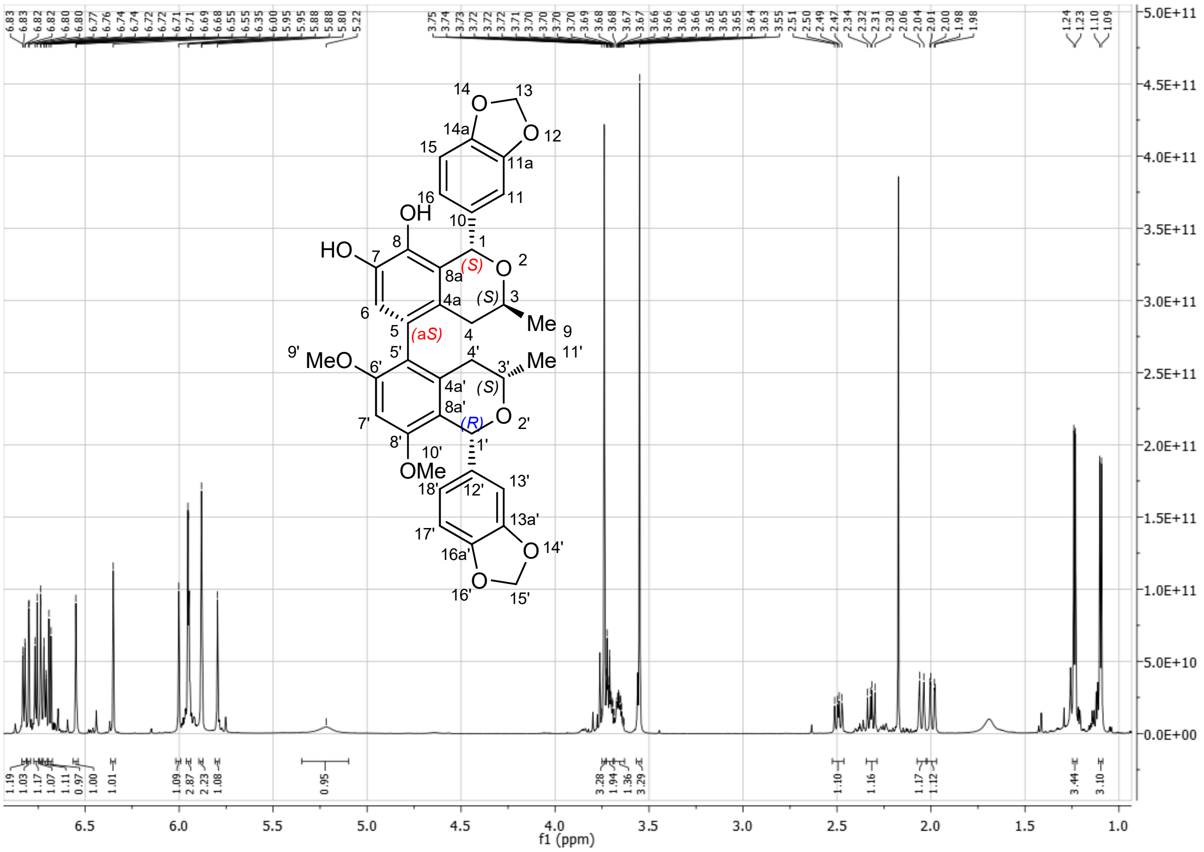

Figure S111. ^{1}H NMR (700 MHz) spectrum of *trans*,*cis*-(a*S*,1*S*,3*S*,1'*R*,3'*S*)-**23** in $CDCl_3$.

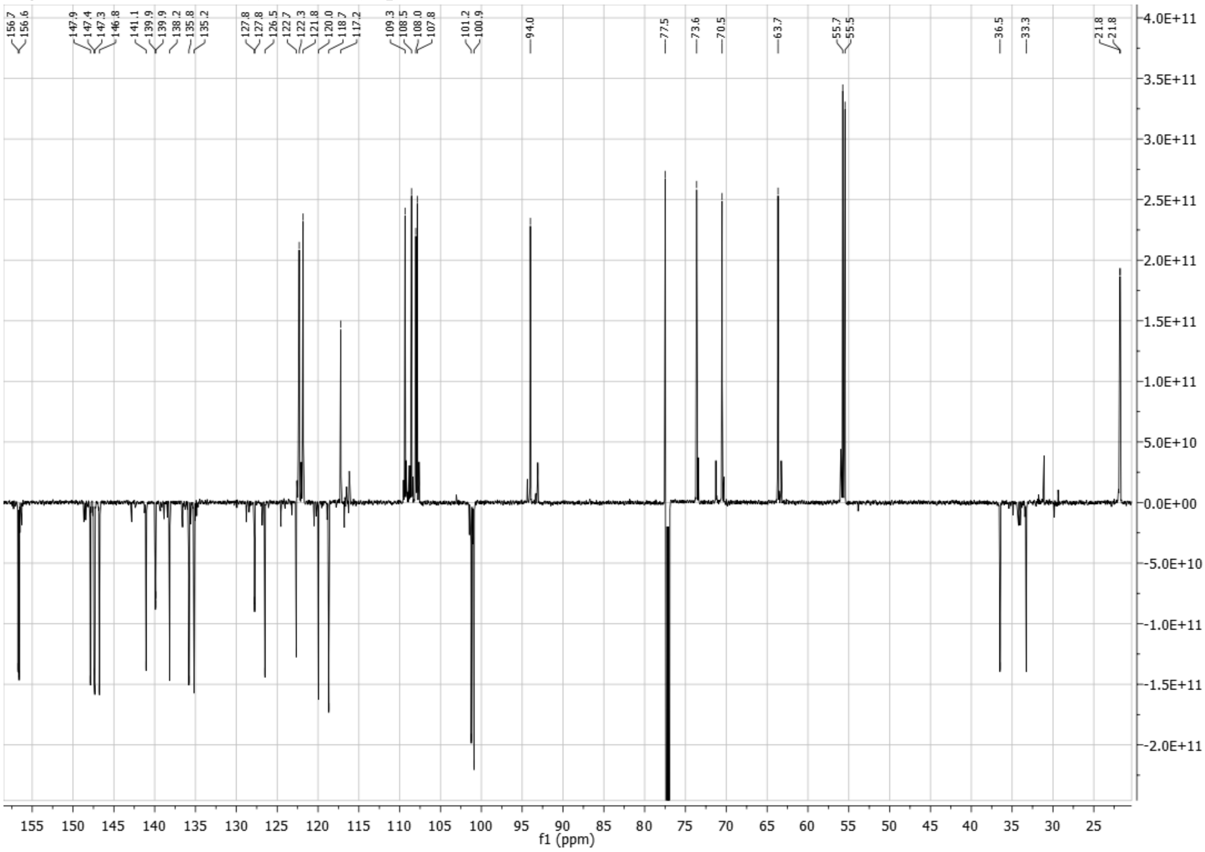

Figure S112. ^{13}C NMR (175 MHz) spectrum of *trans*,*cis*-(a*S*,1*S*,3*S*,1'*R*,3'*S*)-**23** in $CDCl_3$.

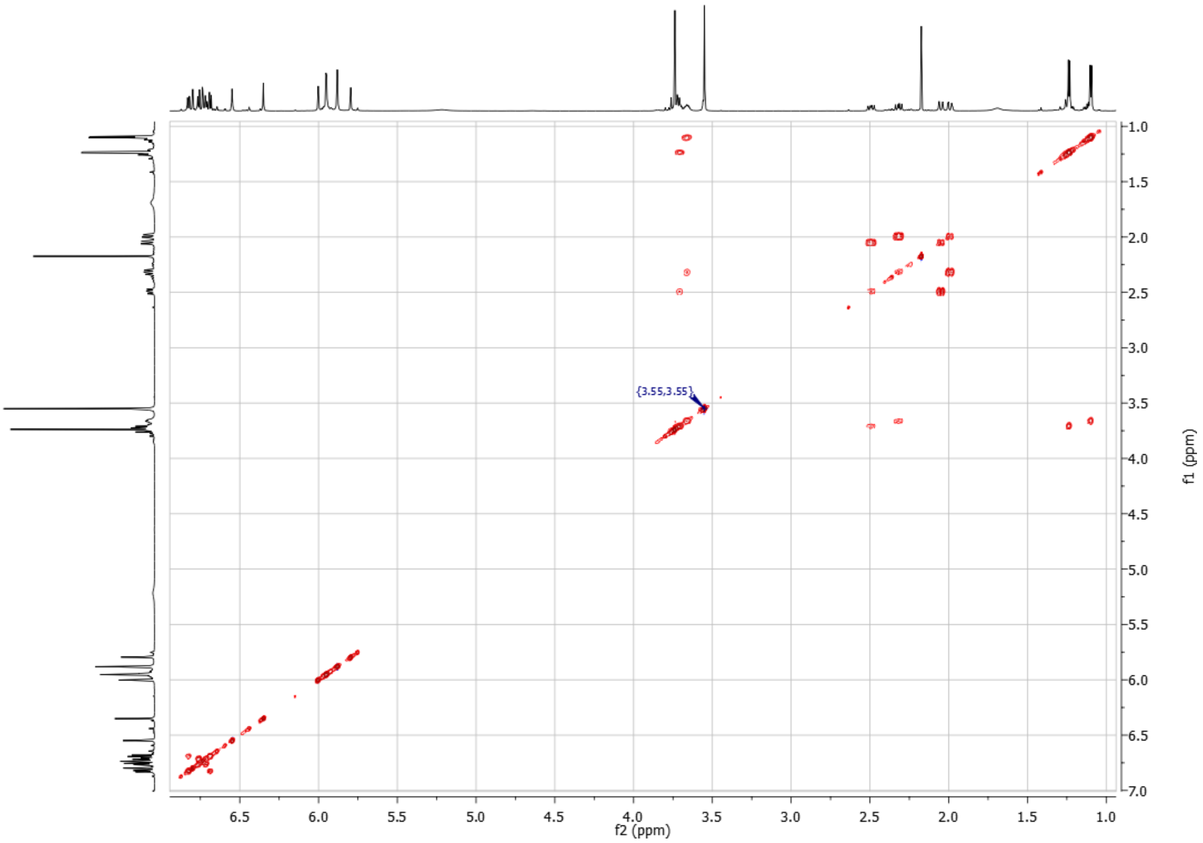

Figure S113. ^{1}H-^{1}H COSY NMR (700 MHz) spectrum of *trans*,*cis*-(a*S*,1*S*,3*S*,1'*R*,3'*S*)-**23** in $CDCl_3$.

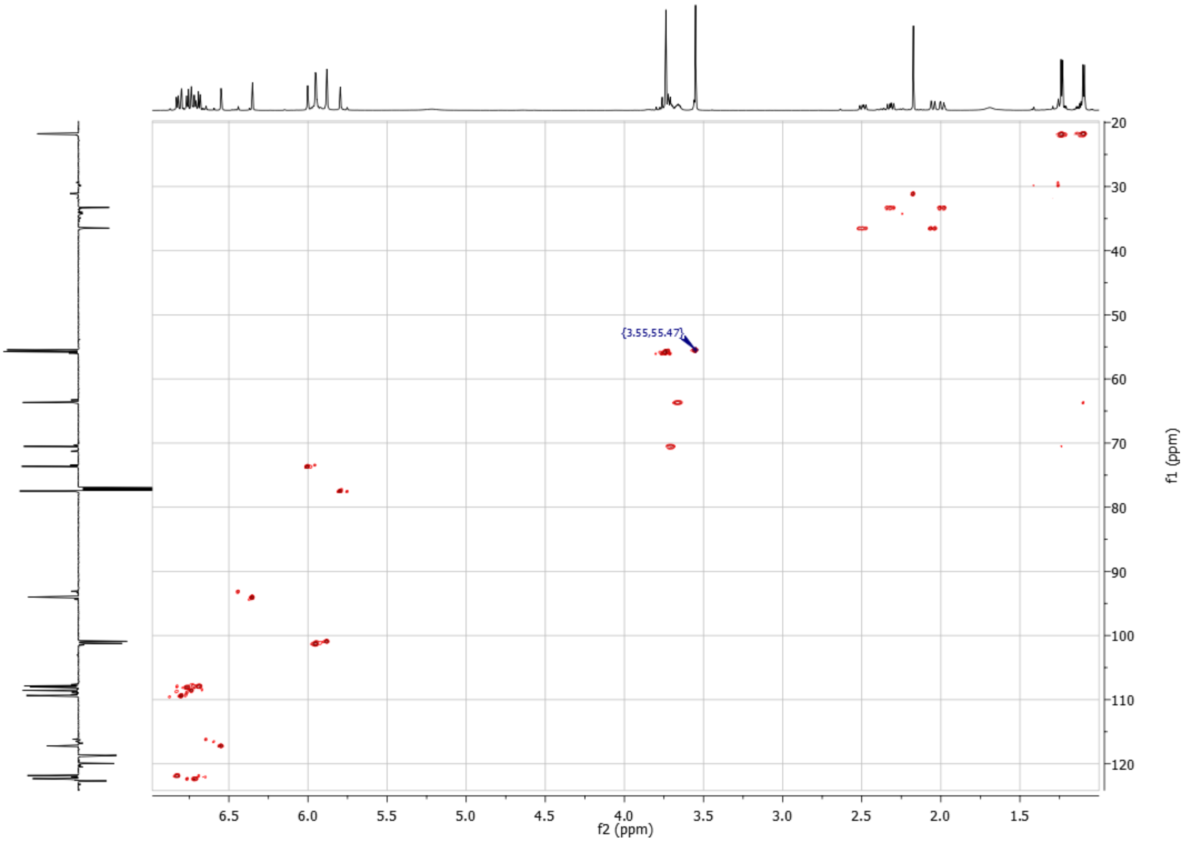

Figure S114. ^{1}H-^{13}C HSQC NMR (700 MHz) spectrum of *trans*,*cis*-(a*S*,1*S*,3*S*,1'*R*,3'*S*)-**23** in $CDCl_3$.

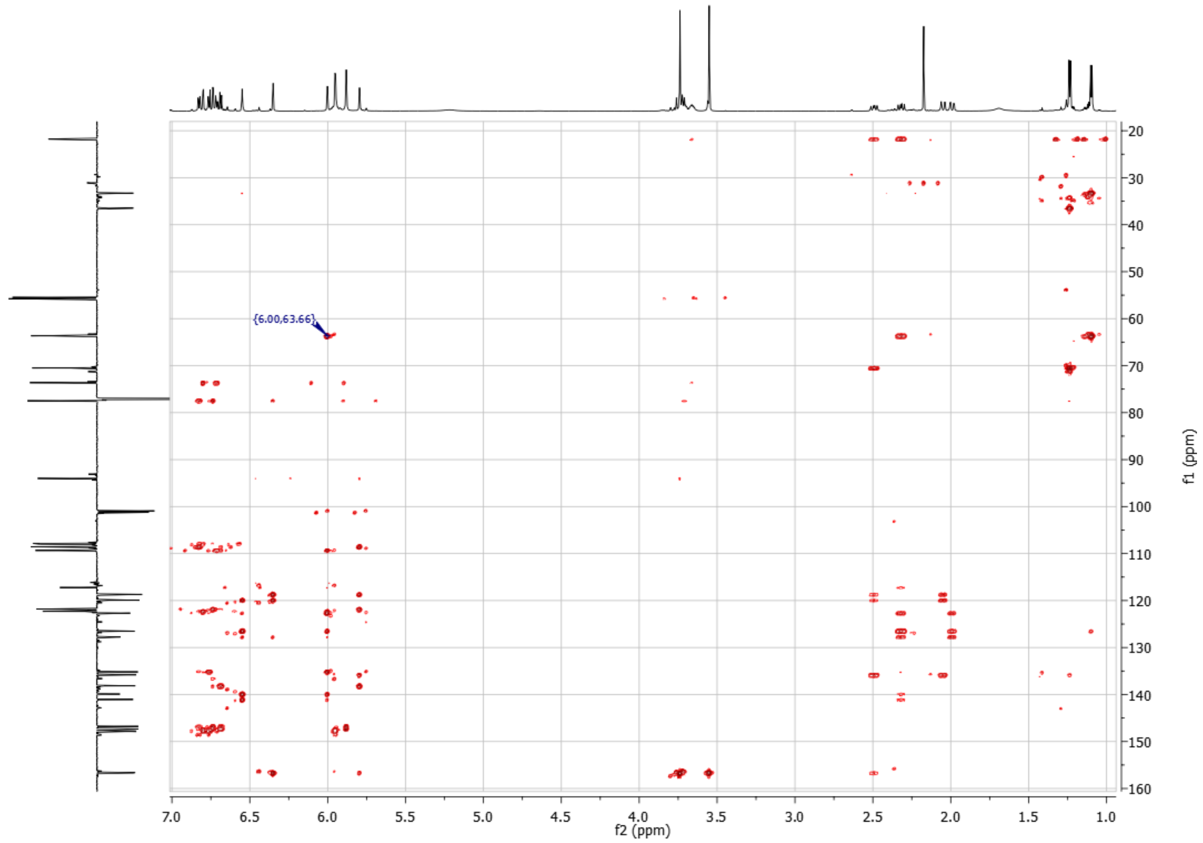

Figure S115. ^{1}H-^{13}C HMBC NMR (700 MHz) spectrum of *trans*,*cis*-(a*S*,1*S*,3*S*,1'*R*,3'*S*)-**23** in $CDCl_3$.

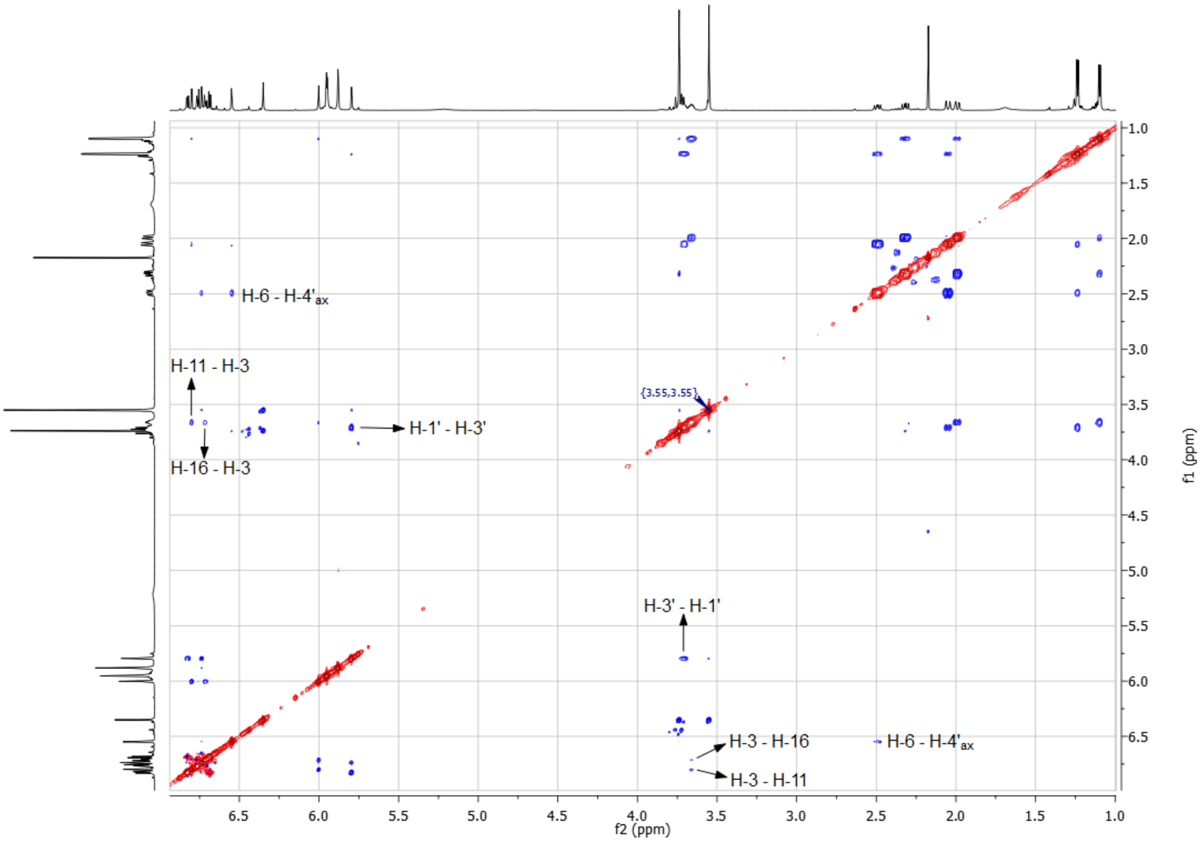

Figure S116. ^{1}H-^{1}H ROESY NMR (700 MHz) spectrum of *trans*,*cis*-(a*S*,1*S*,3*S*,1'*R*,3'*S*)-**23** in $CDCl_3$.

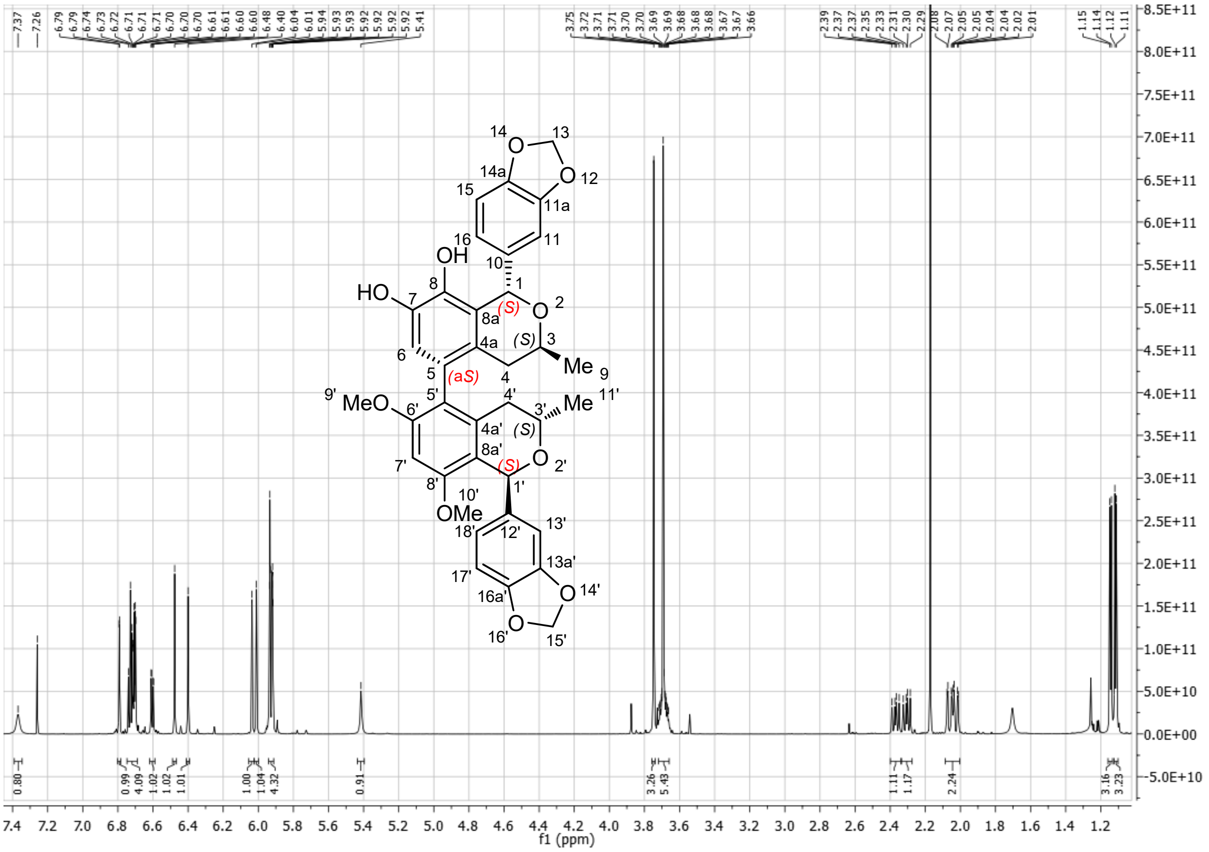

Figure S117. ^{1}H NMR (700 MHz) spectrum of *trans*,*trans*-(a*S*,1*S*,3*S*,1'*S*,3'*S*)-**23** in $CDCl_3$.

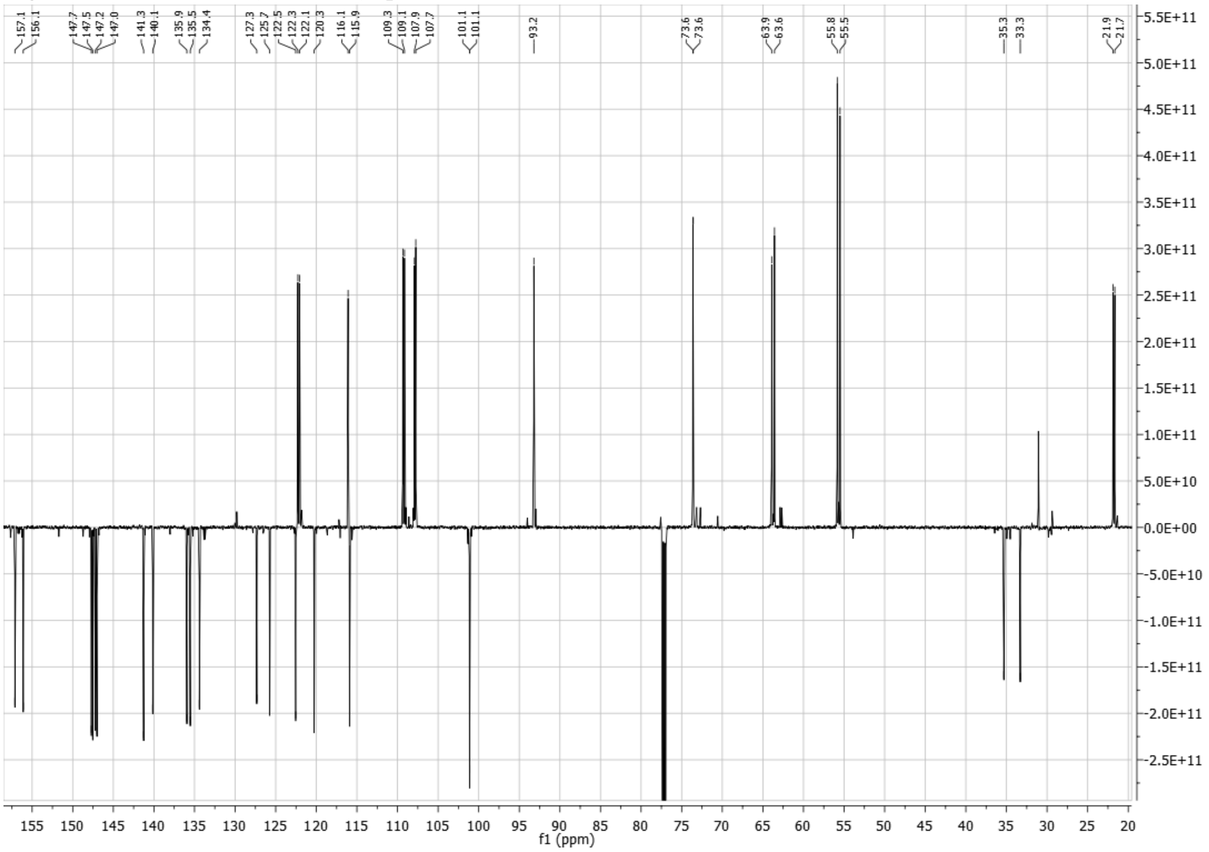

Figure S118. ^{13}C NMR (175 MHz) spectrum of *trans*,*trans*-(a*S*,1*S*,3*S*,1'*S*,3'*S*)-**23** in $CDCl_3$.

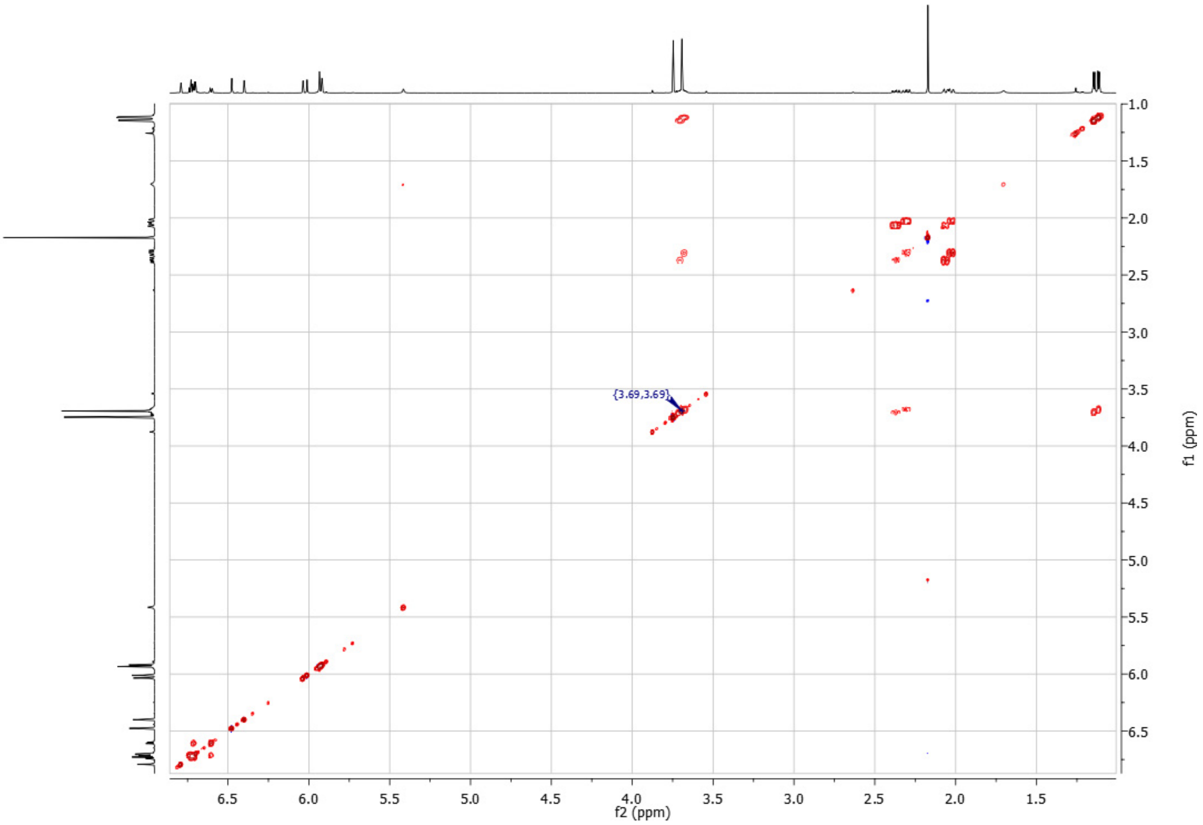

Figure S119. ^{1}H-^{1}H COSY NMR (700 MHz) spectrum of *trans*,*trans*-(a*S*,1*S*,3*S*,1'*S*,3'*S*)-**23** in $CDCl_3$.

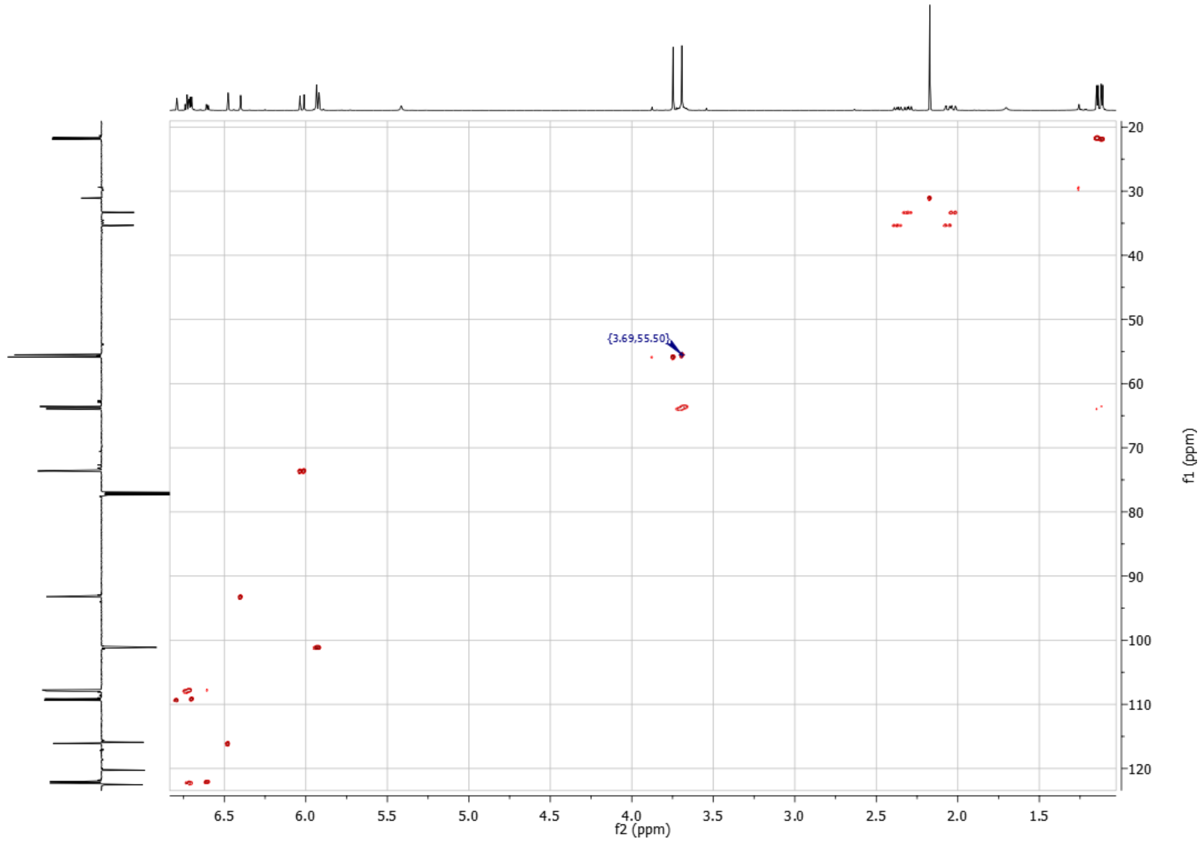

Figure S120. ^{1}H-^{13}C HSQC NMR (700 MHz) spectrum of *trans*,*trans*-(a*S*,1*S*,3*S*,1'*S*,3'*S*)-**23** in $CDCl_3$.

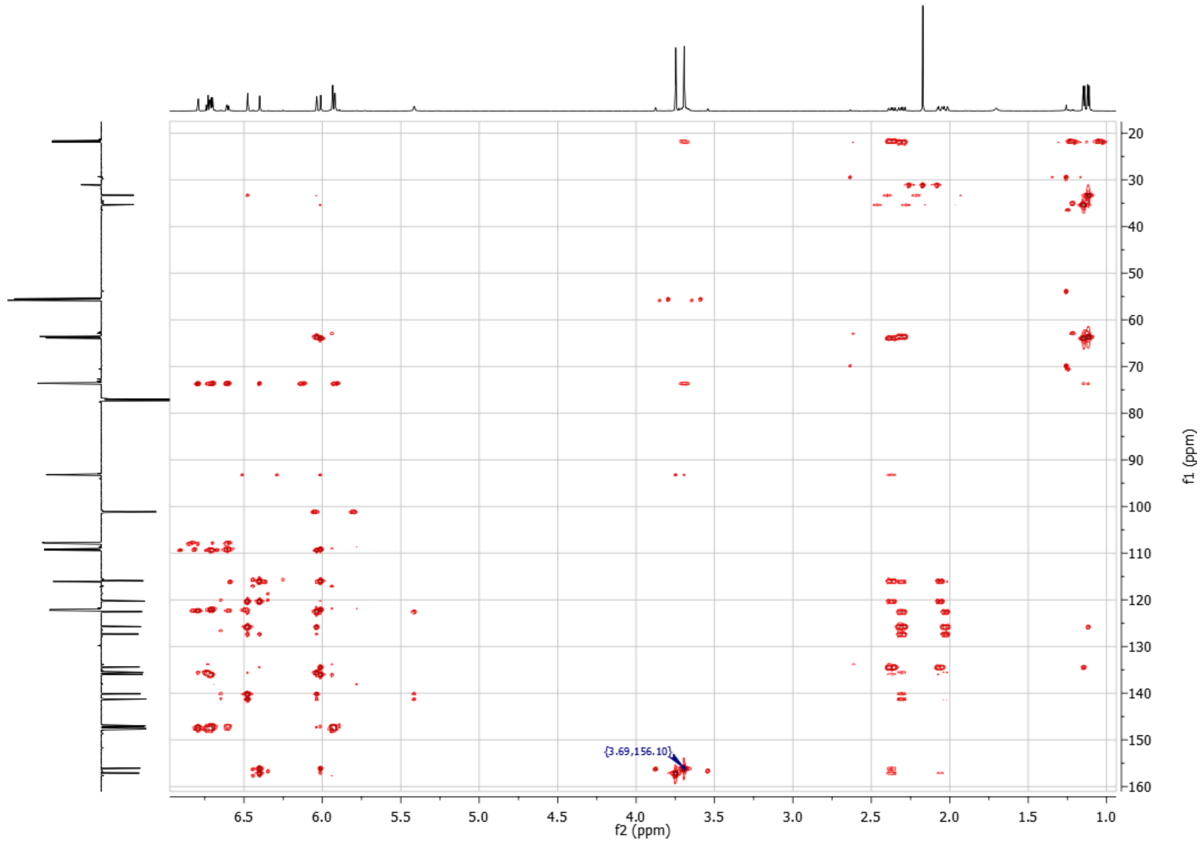

Figure S121. ^{1}H-^{13}C HMBC NMR (700 MHz) spectrum of *trans*,*trans*-(a*S*,1*S*,3*S*,1’*S*,3’*S*)-**23** in $CDCl_3$.

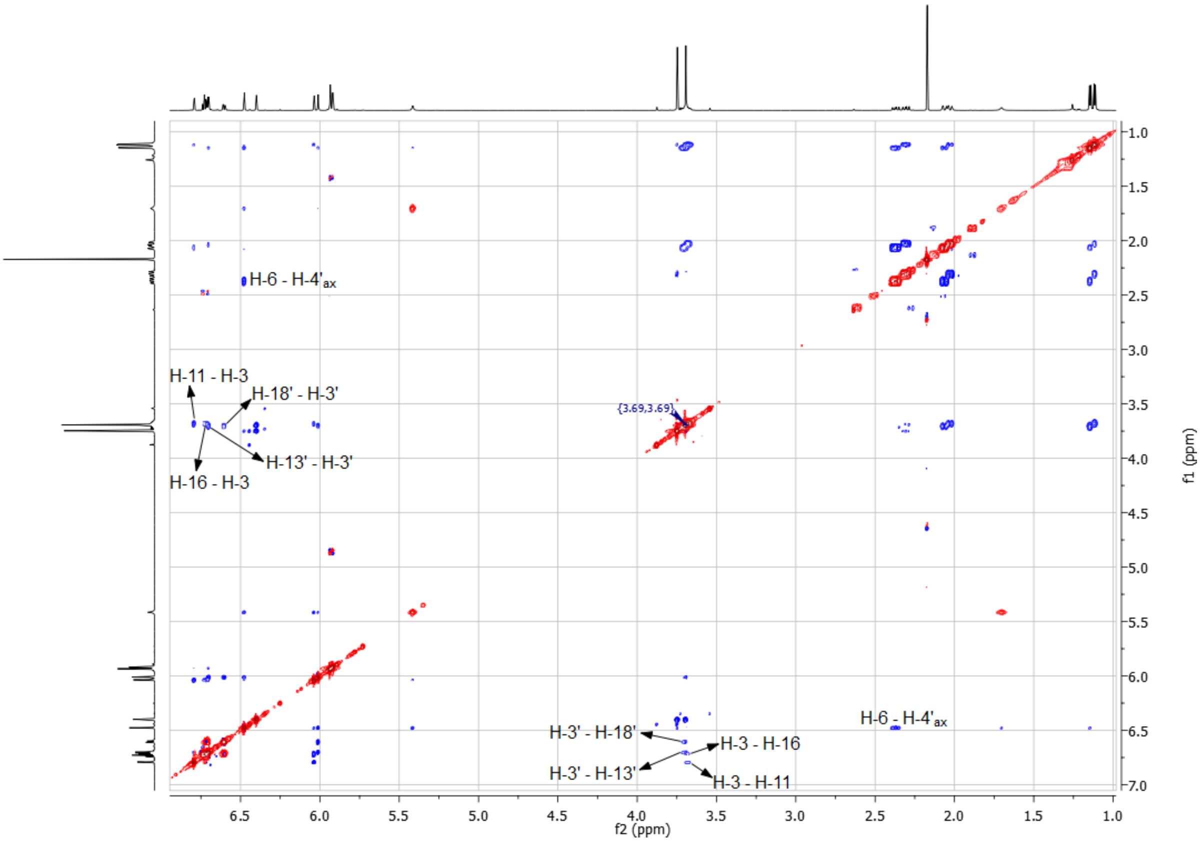

Figure S122. ^{1}H-^{1}H ROESY NMR (700 MHz) spectrum of *trans*,*trans*-(a*S*,1*S*,3*S*,1’*S*,3’*S*)-**23** in $CDCl_3$.

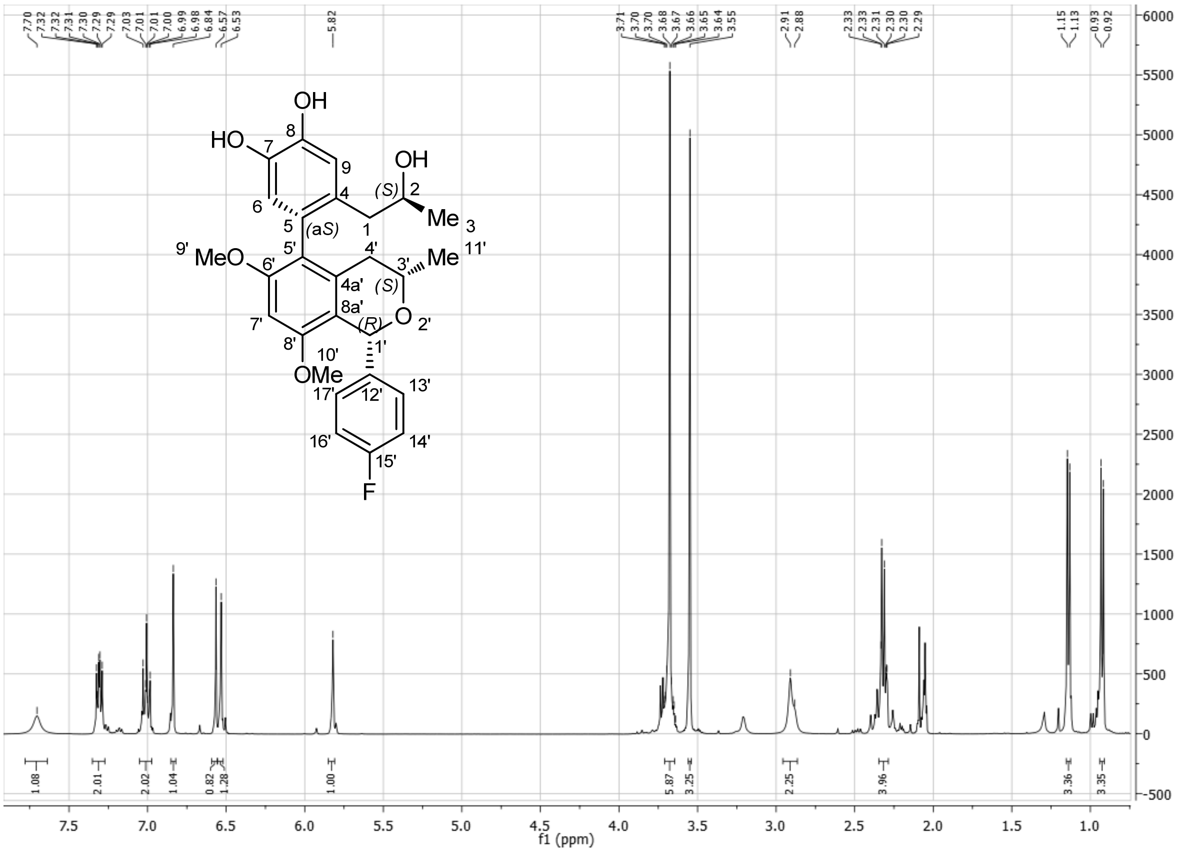

Figure S123. ^{1}H NMR (400 MHz) spectrum of *cis*-(a*S*,2*S*,1'*R*,3'*S*)-**24** in acetone-d_6.

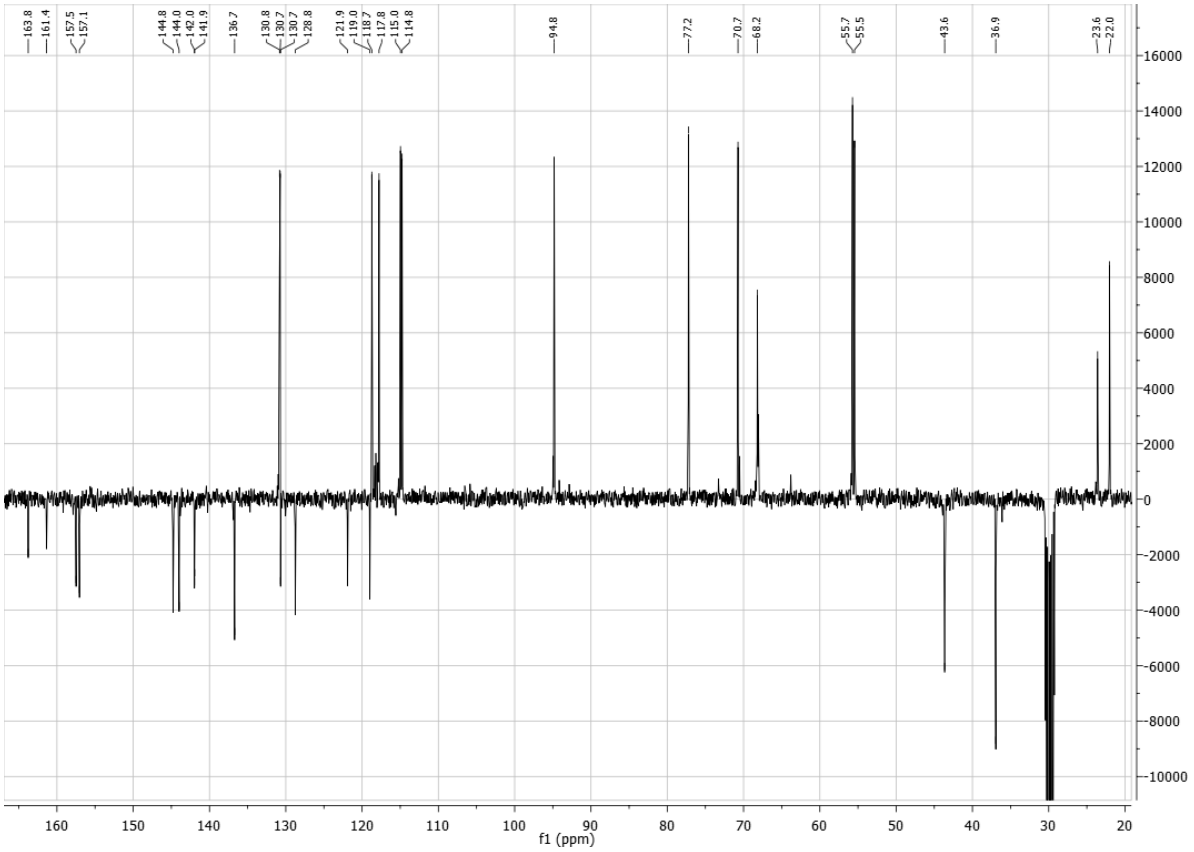

Figure S124. ^{13}C NMR (100 MHz) spectrum of *cis*-(a*S*,2*S*,1'*R*,3'*S*)-**24** in acetone-d_6.

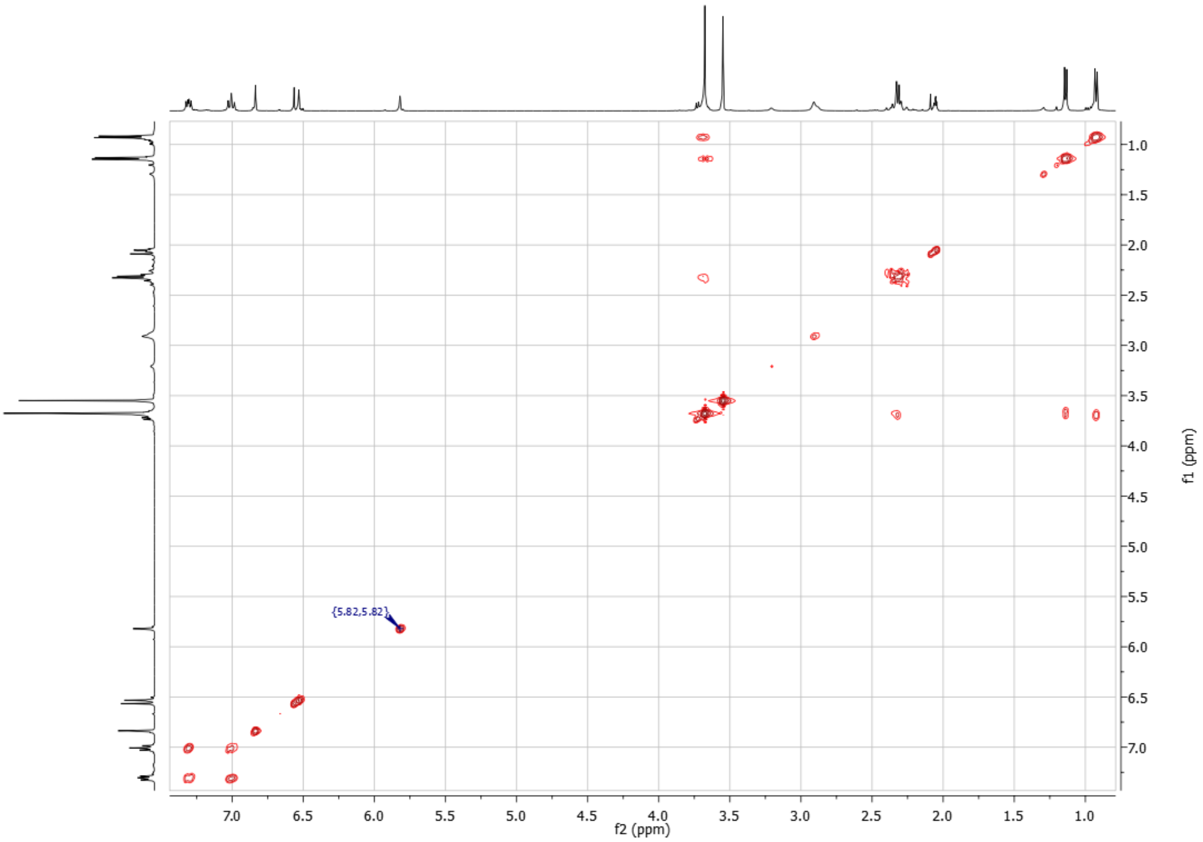

Figure S125. ^{1}H-^{1}H COSY NMR (400 MHz) spectrum of of *cis*-(a*S*,2*S*,1'*R*,3'*S*)-**24** in acetone-d_6.

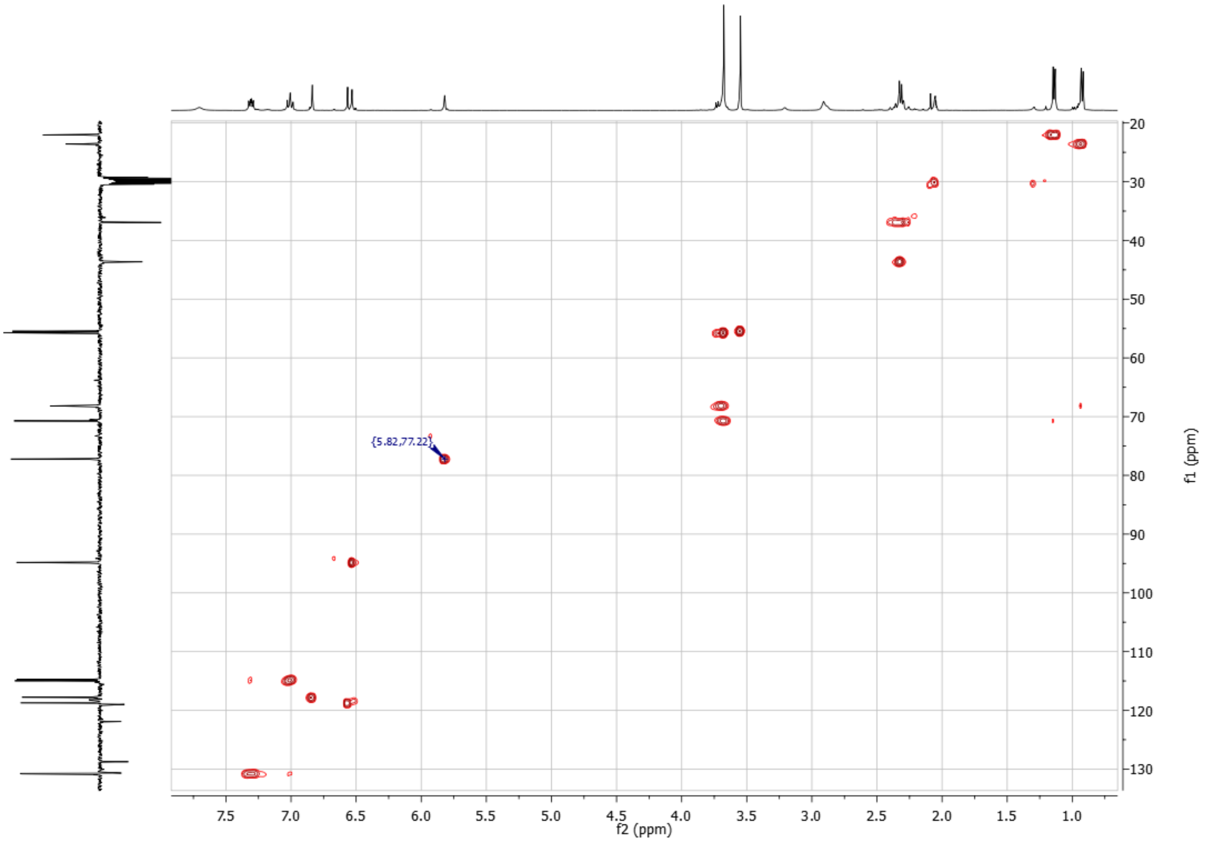

Figure S126. ^{1}H-^{13}C HSQC NMR (400 MHz) spectrum of of *cis*-(a*S*,2*S*,1'*R*,3'*S*)-**24** in acetone-d_6.

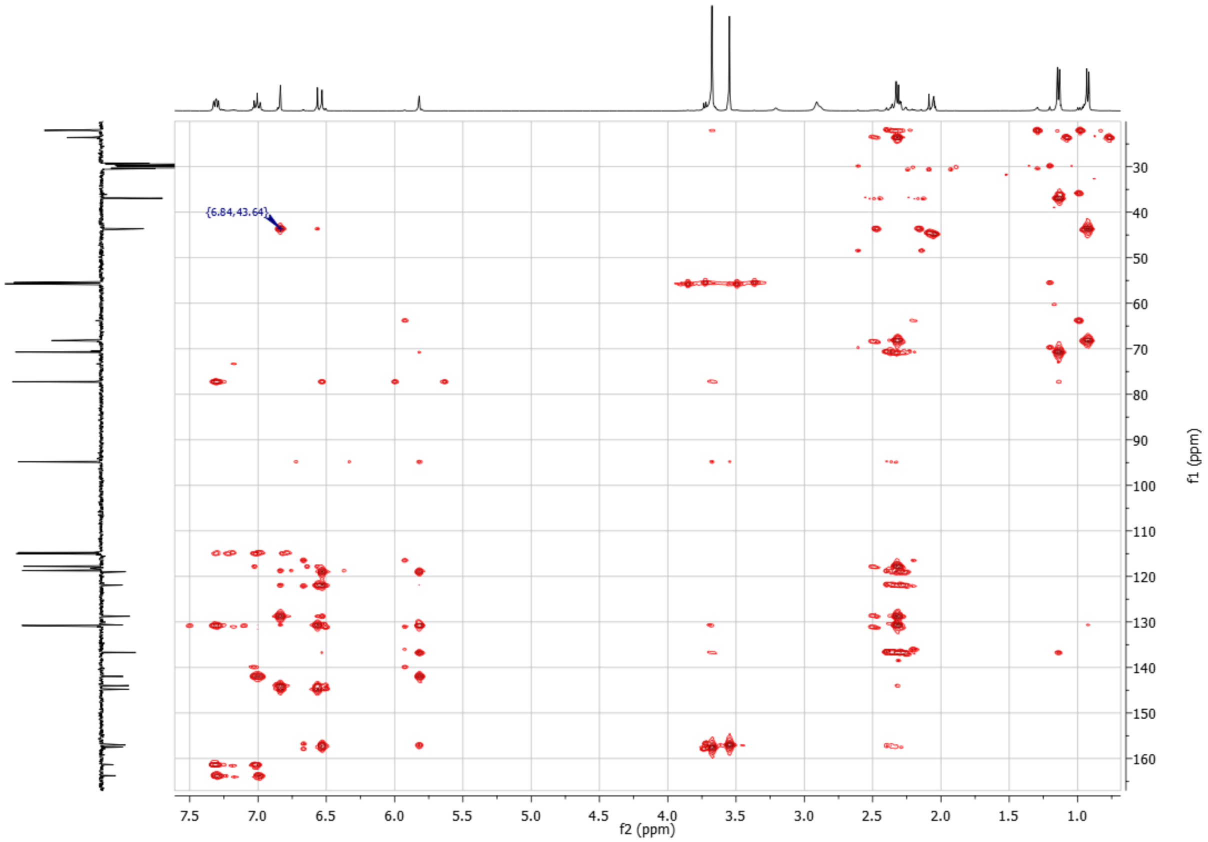

Figure S127. ^{1}H-^{13}C HMBC NMR (400 MHz) spectrum of of *cis*-(a*S*,2*S*,1'*R*,3'*S*)-**24** in acetone-d_6.

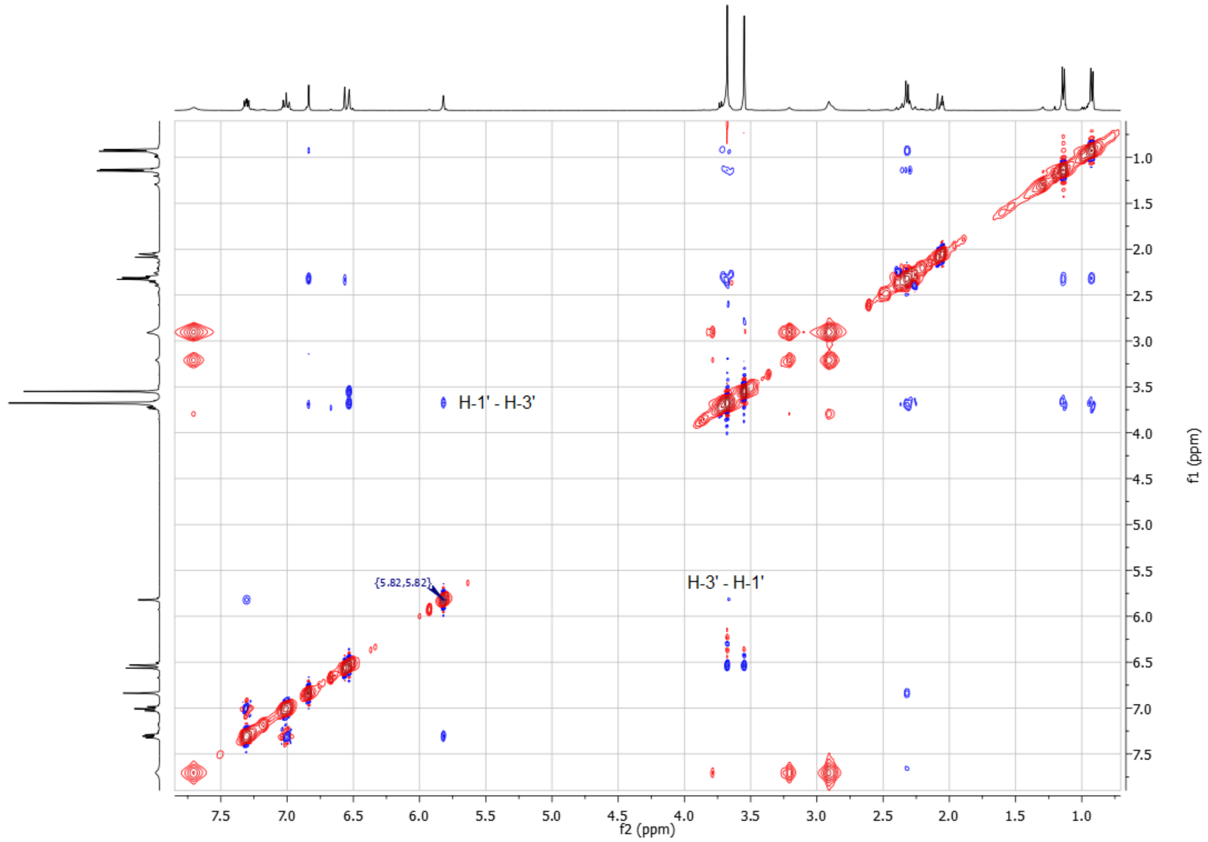

Figure S128. ^{1}H-^{1}H NOESY NMR (400 MHz) spectrum of of *cis*-(a*S*,2*S*,1'*R*,3'*S*)-**24** in acetone-d_6.

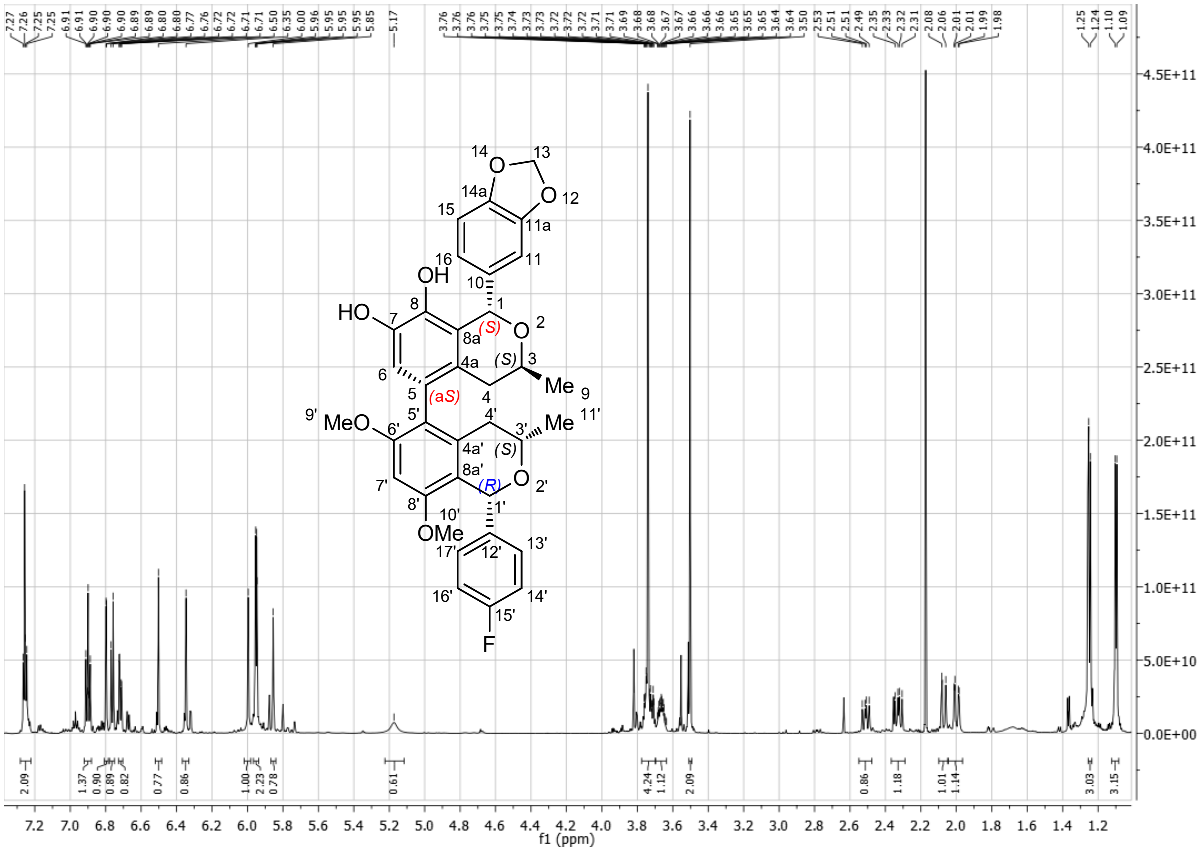

Figure S129. ^{1}H NMR (700 MHz) spectrum of *trans*,*cis*-(a*S*,1*S*,3*S*,1'*R*,3'*S*)-**25** in $CDCl_3$.

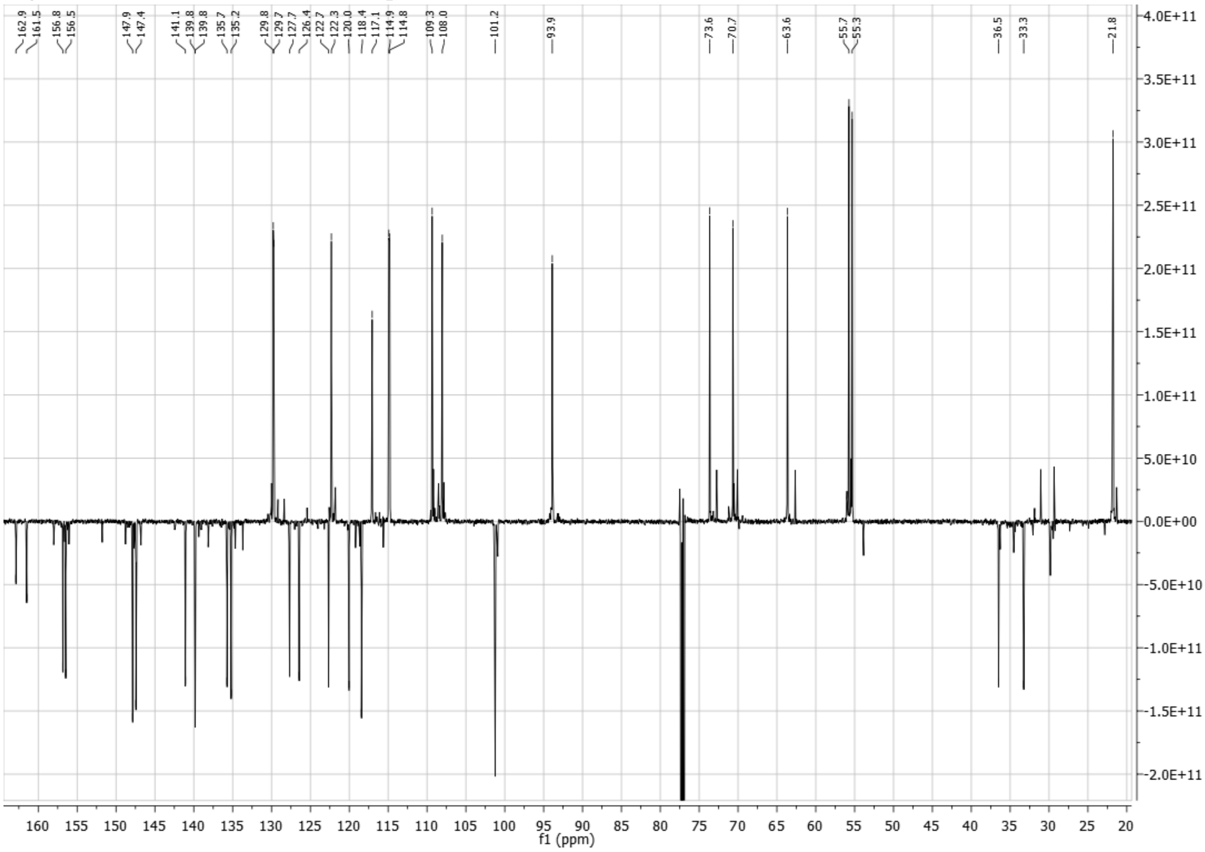

Figure S130. ^{13}C NMR (175 MHz) spectrum of *trans*,*cis*-(a*S*,1*S*,3*S*,1'*R*,3'*S*)-**25** in $CDCl_3$.

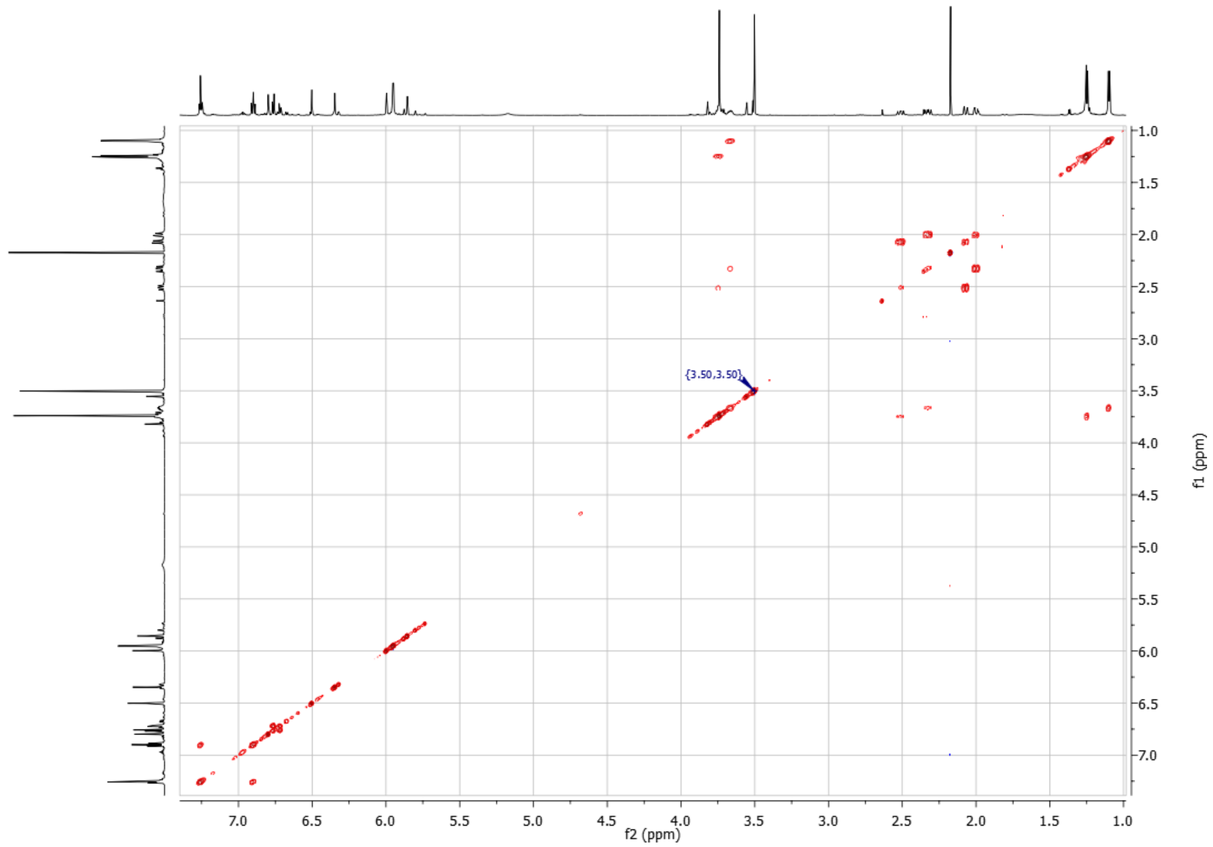

Figure S131. ^{1}H-^{1}H COSY NMR (700 MHz) spectrum of *trans*,*cis*-(a*S*,1*S*,3*S*,1'*R*,3'*S*)-**25** in $CDCl_3$.

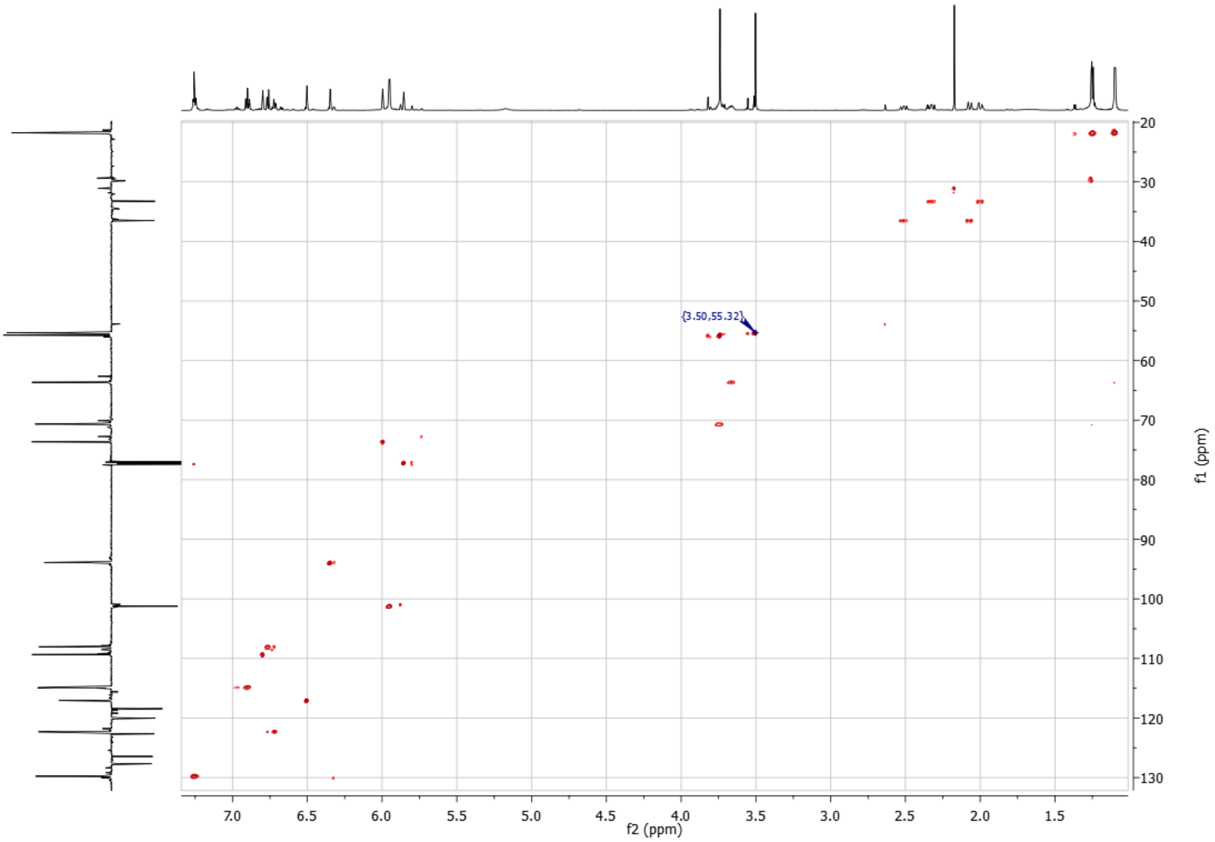

Figure S132. ^{1}H-^{13}C HSQC NMR (700 MHz) spectrum of *trans*,*cis*-(a*S*,1*S*,3*S*,1'*R*,3'*S*)-**25** in $CDCl_3$.

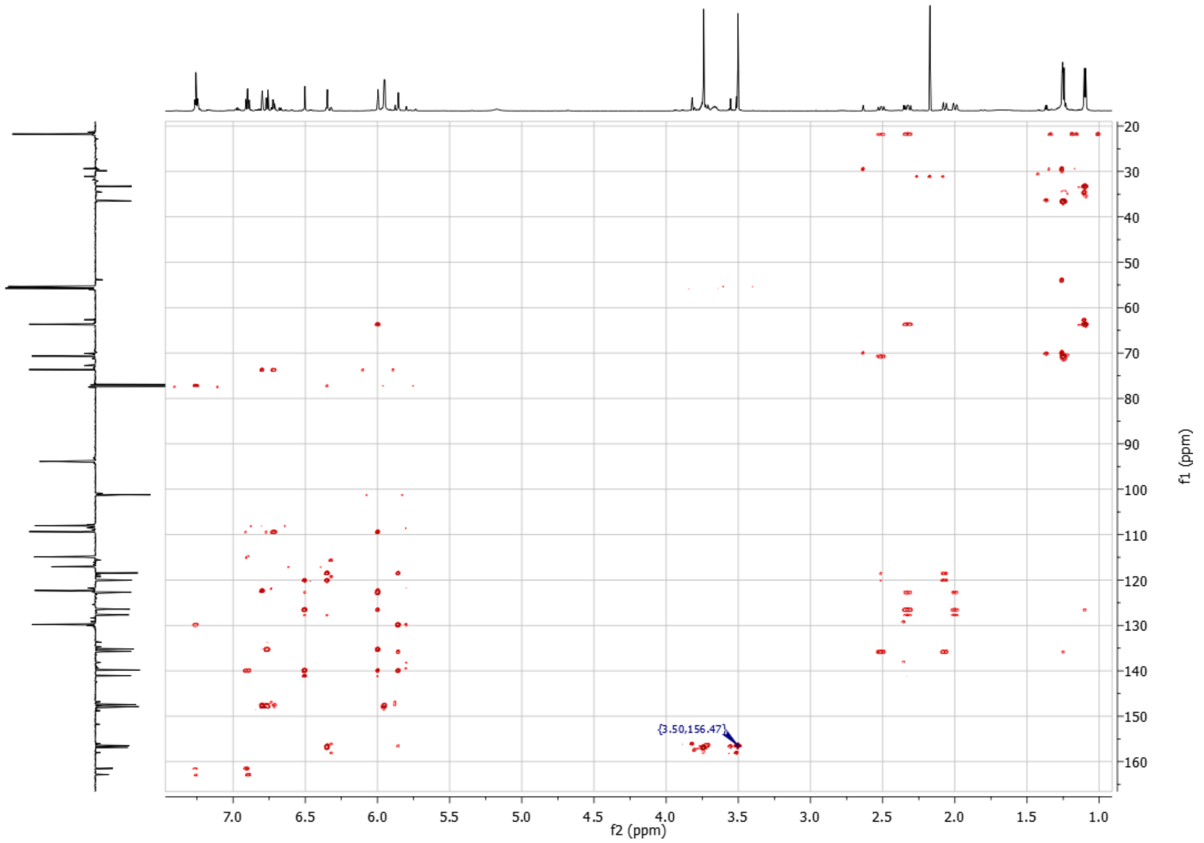

Figure S133. ^{1}H-^{13}C HMBC NMR (700 MHz) spectrum of *trans*,*cis*-(a*S*,1*S*,3*S*,1'*R*,3'*S*)-**25** in $CDCl_3$.

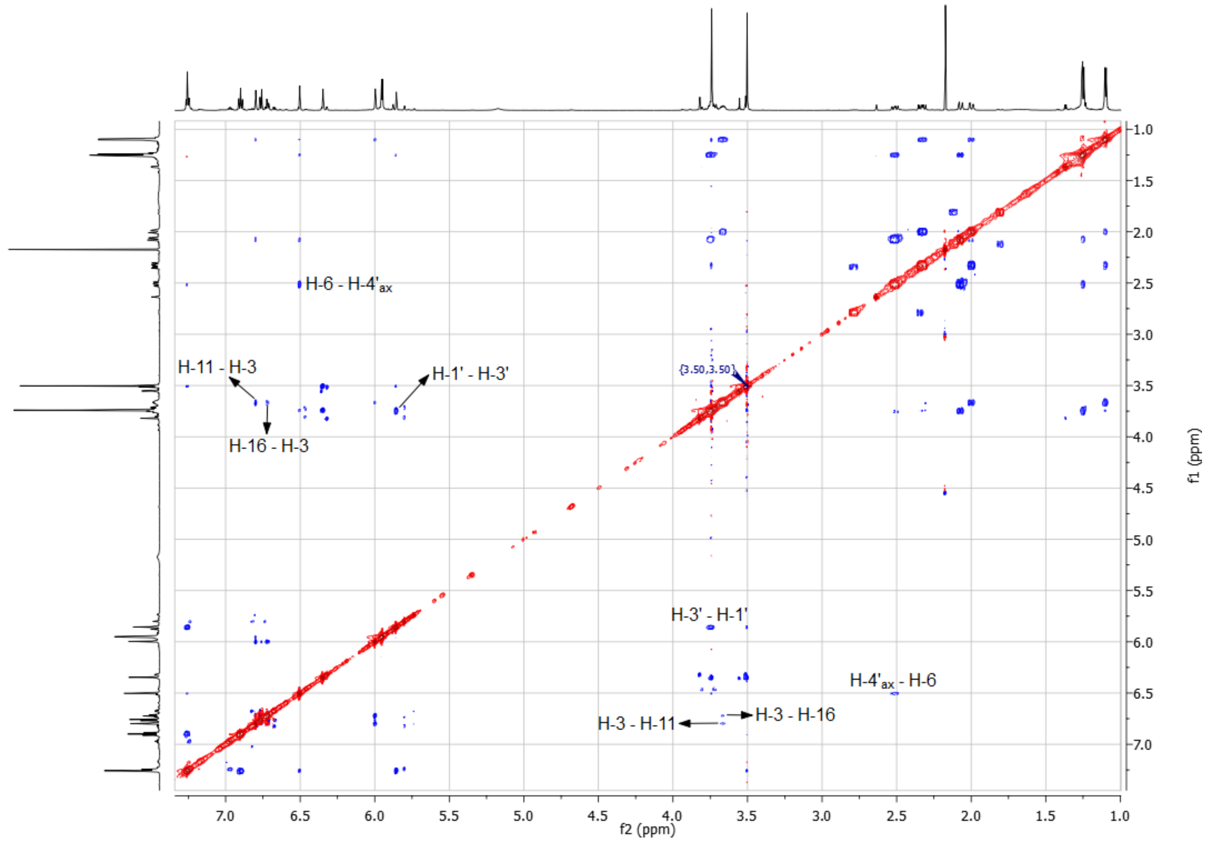

Figure S134. ^{1}H-^{1}H ROESY NMR (700 MHz) spectrum of *trans*,*cis*-(a*S*,1*S*,3*S*,1'*R*,3'*S*)-**25** in $CDCl_3$.

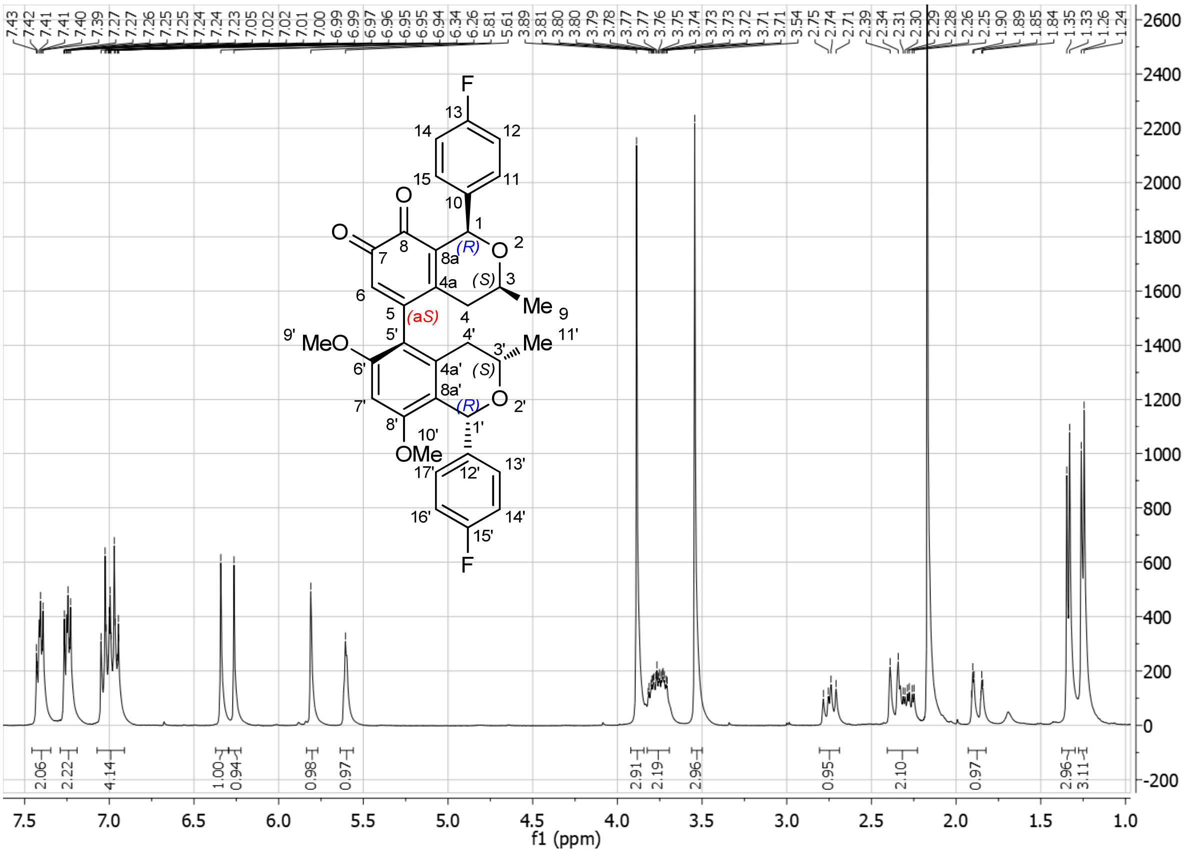

Figure S135. ^{1}H NMR (360 MHz) spectrum of *cis*,*cis*-(a*S*,1*R*,3*S*,1'*R*,3'*S*)-**26** in $CDCl_3$.

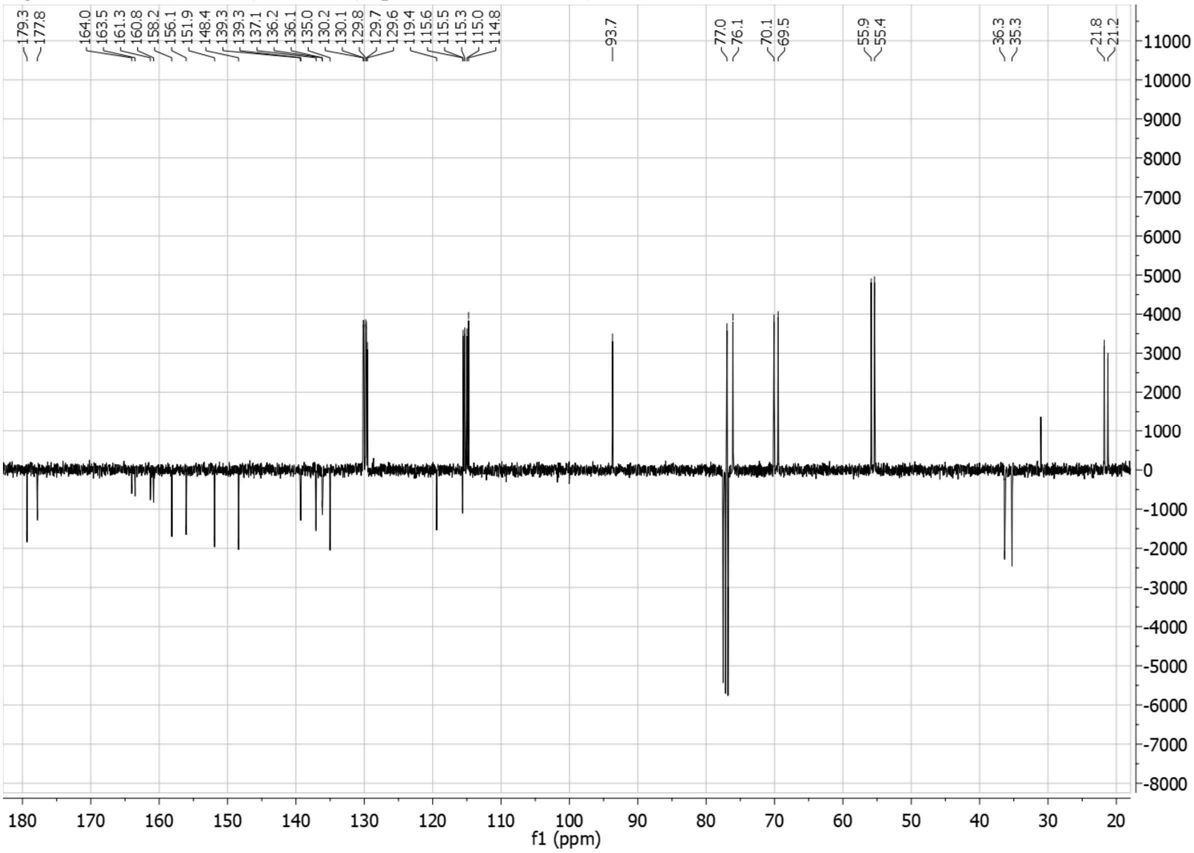

Figure S136. ^{13}C NMR (90 MHz) spectrum of *cis*,*cis*-(a*S*,1*R*,3*S*,1'*R*,3'*S*)-**26** in $CDCl_3$.

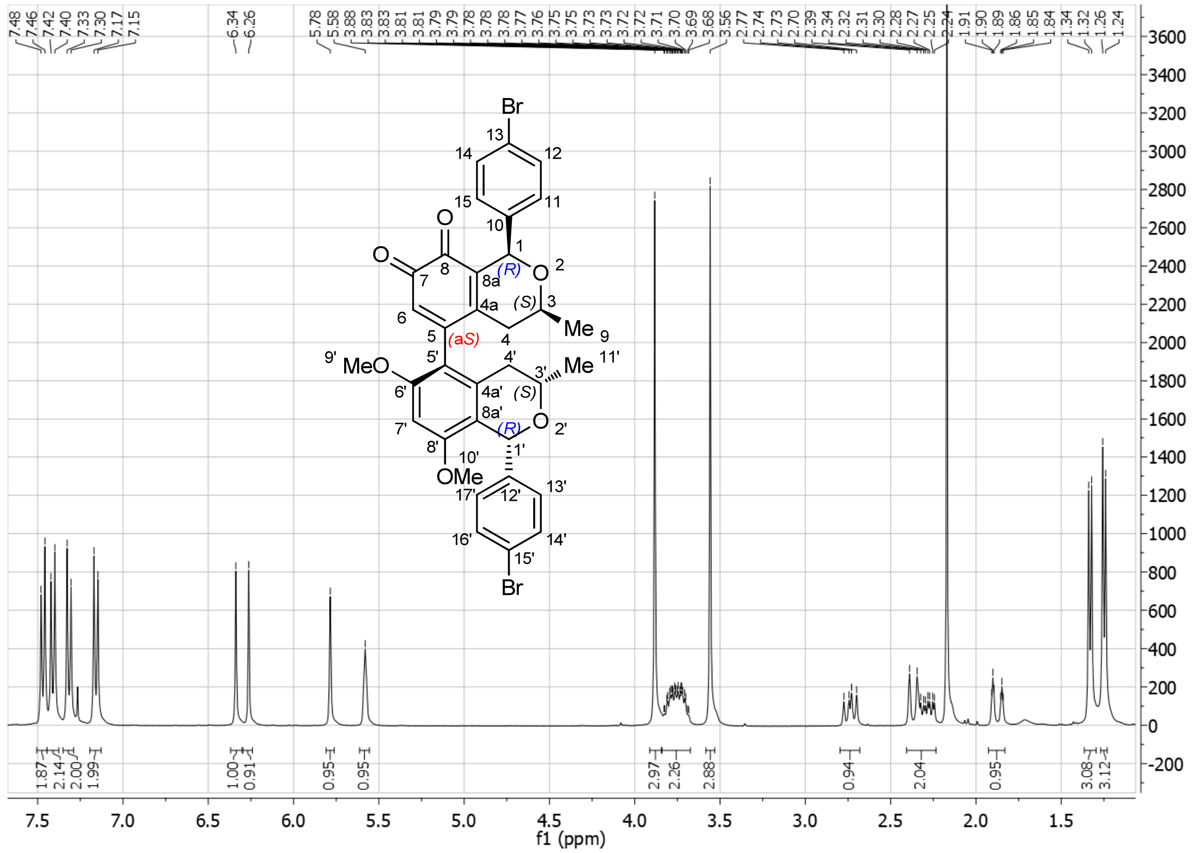

Figure S137. ^{1}H NMR (360 MHz) spectrum of *cis*,*cis*-(a*S*,1*R*,3*S*,1’*R*,3’*S*)-**27** in $CDCl_3$.

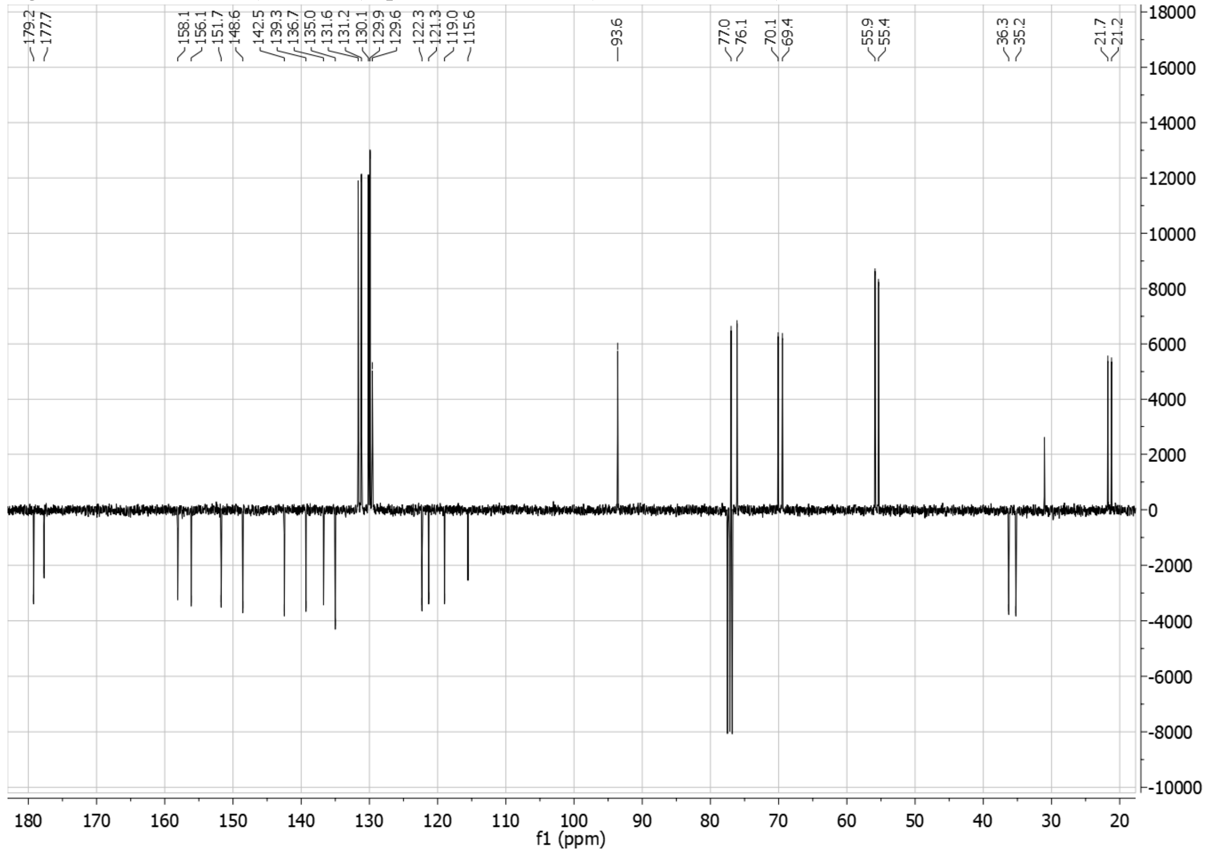

Figure S138. ^{13}C NMR (90 MHz) spectrum of in *cis*,*cis*-(a*S*,1*R*,3*S*,1’*R*,3’*S*)-**27** $CDCl_3$.

3.2. ECD and VCD spectra

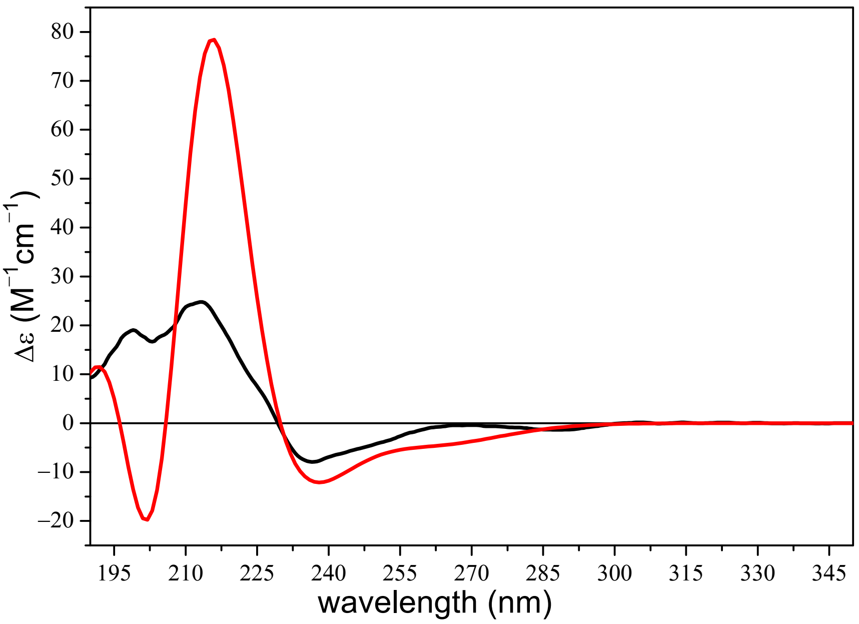

Figure S139. Comparison of the experimental ECD spectrum of (a*S*,3*S*,3'*S*)-**19** (black) with the corresponding computed Neg CAM-B3LYP/TZVP PCM/MeCN ECD spectrum (red).

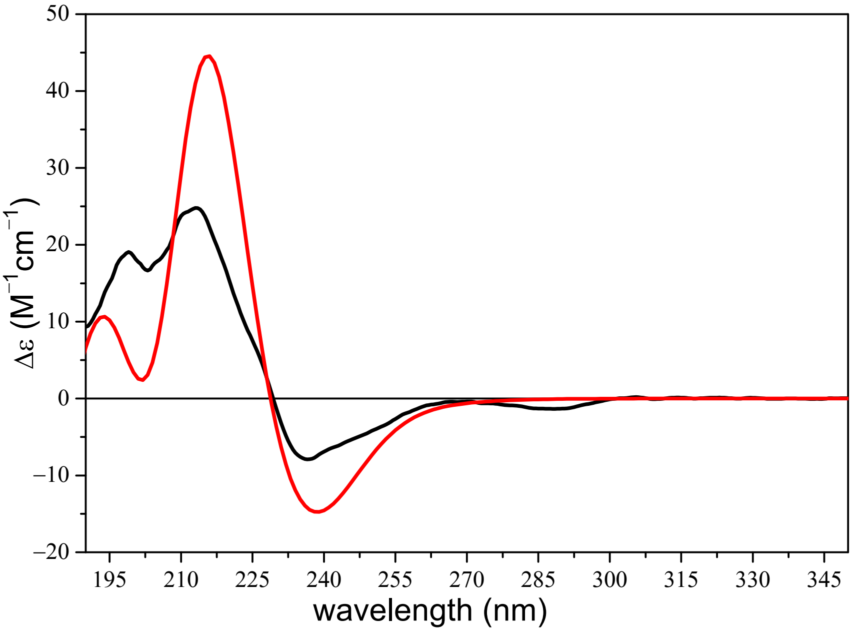

Figure S140. Comparison of the experimental ECD spectrum of (a*S*,3*S*,3'*S*)-**19** (black) with the corresponding computed Pos CAM-B3LYP/TZVP PCM/MeCN ECD spectrum (red).

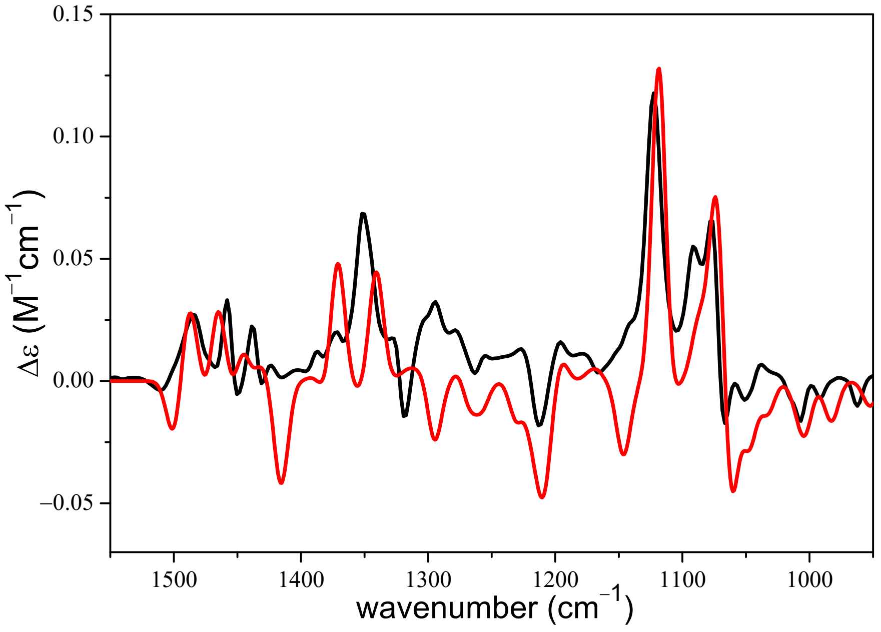

Figure S141. Comparison of the experimental VCD spectrum of (a*S*,3*S*,3’*S*)-**19** (black) with the corresponding computed Neg B3LYP/TZVP PCM/$CHCl_3$ VCD spectrum (red).

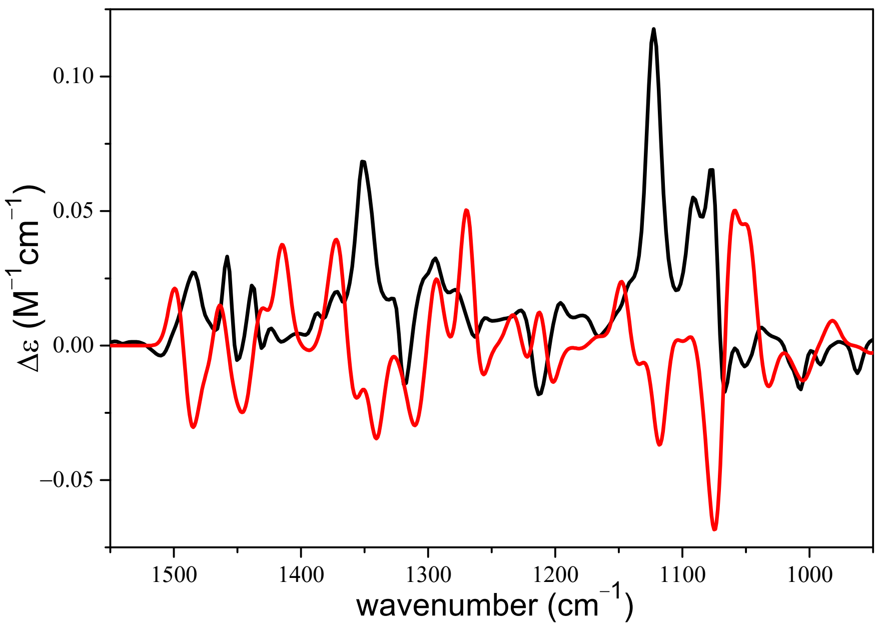

Figure S142. Comparison of the experimental VCD spectrum of (a*S*,3*S*,3’*S*)-**19** (black) with the corresponding computed Pos B3LYP/TZVP PCM/$CHCl_3$ VCD spectrum (red).

3.3. IR spectra

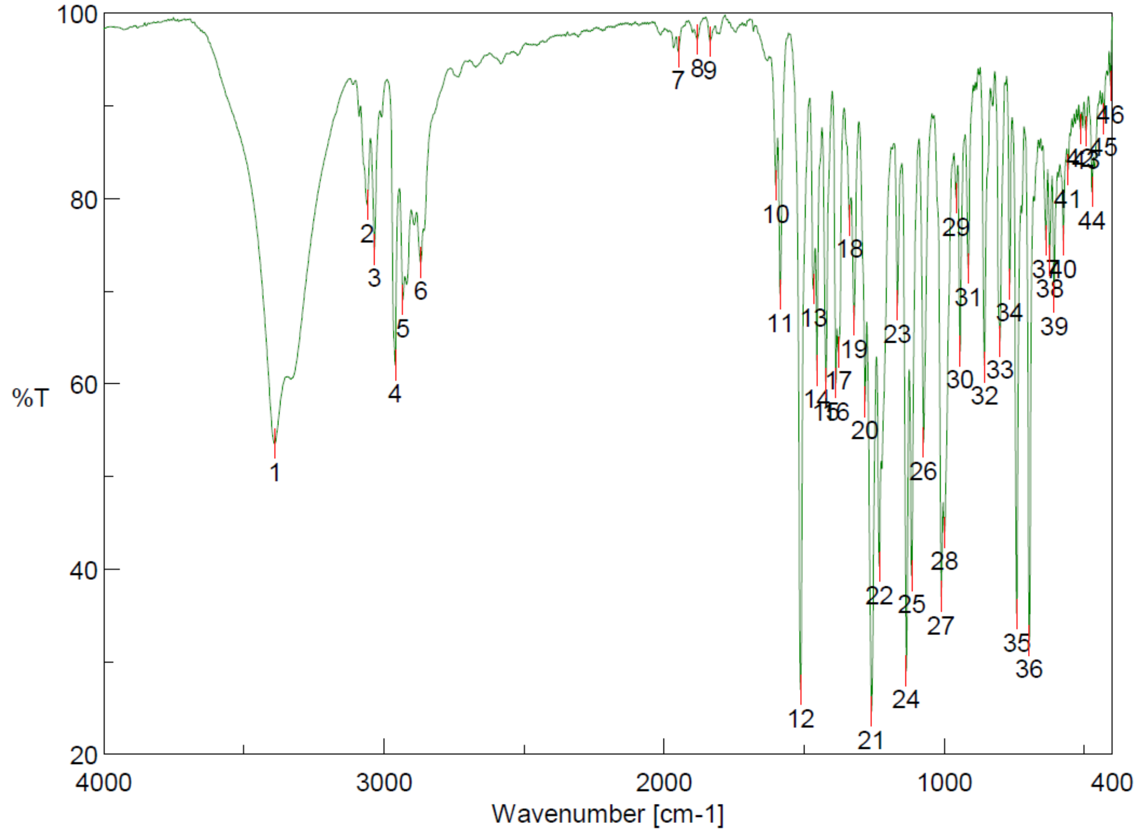

Figure S143. Experimental IR spectrum of (*S*)-**11** recorded as KBr disc.

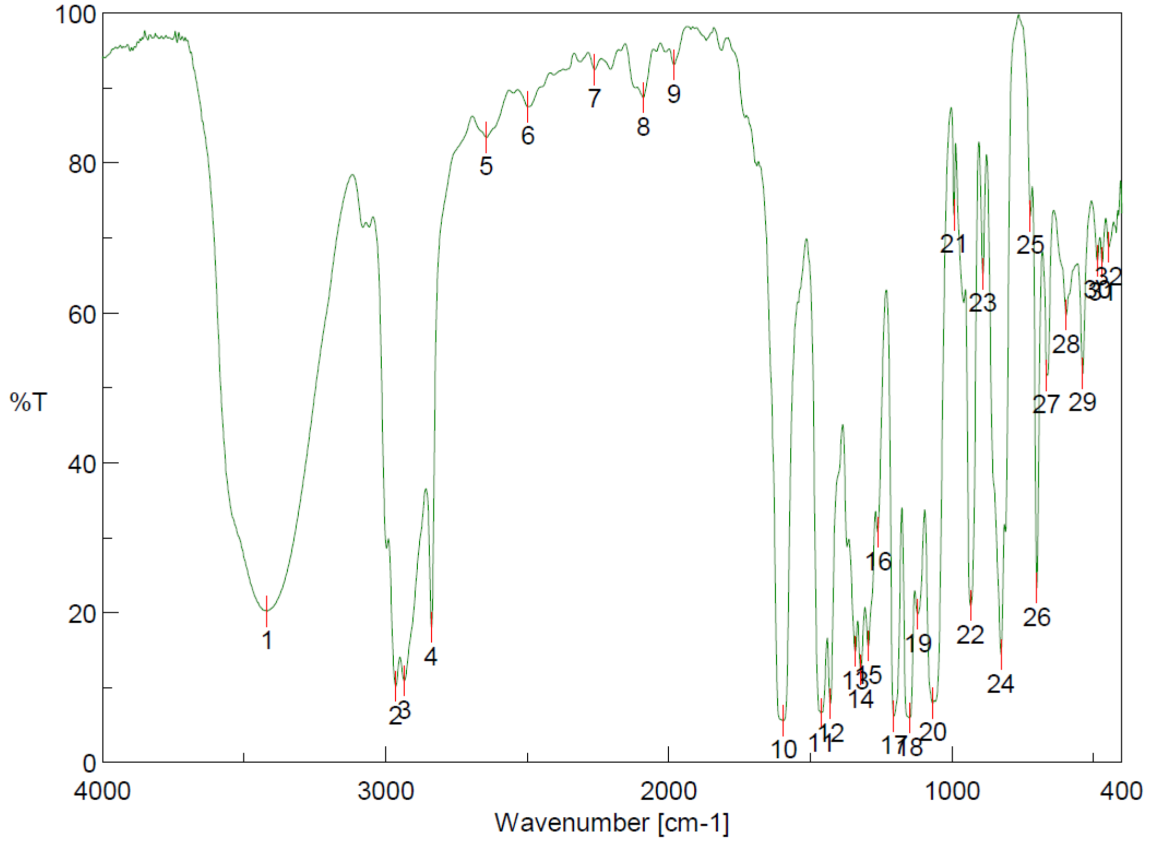

Figure S144. Experimental IR spectrum of (*S*)-**6** recorded as KBr disc.

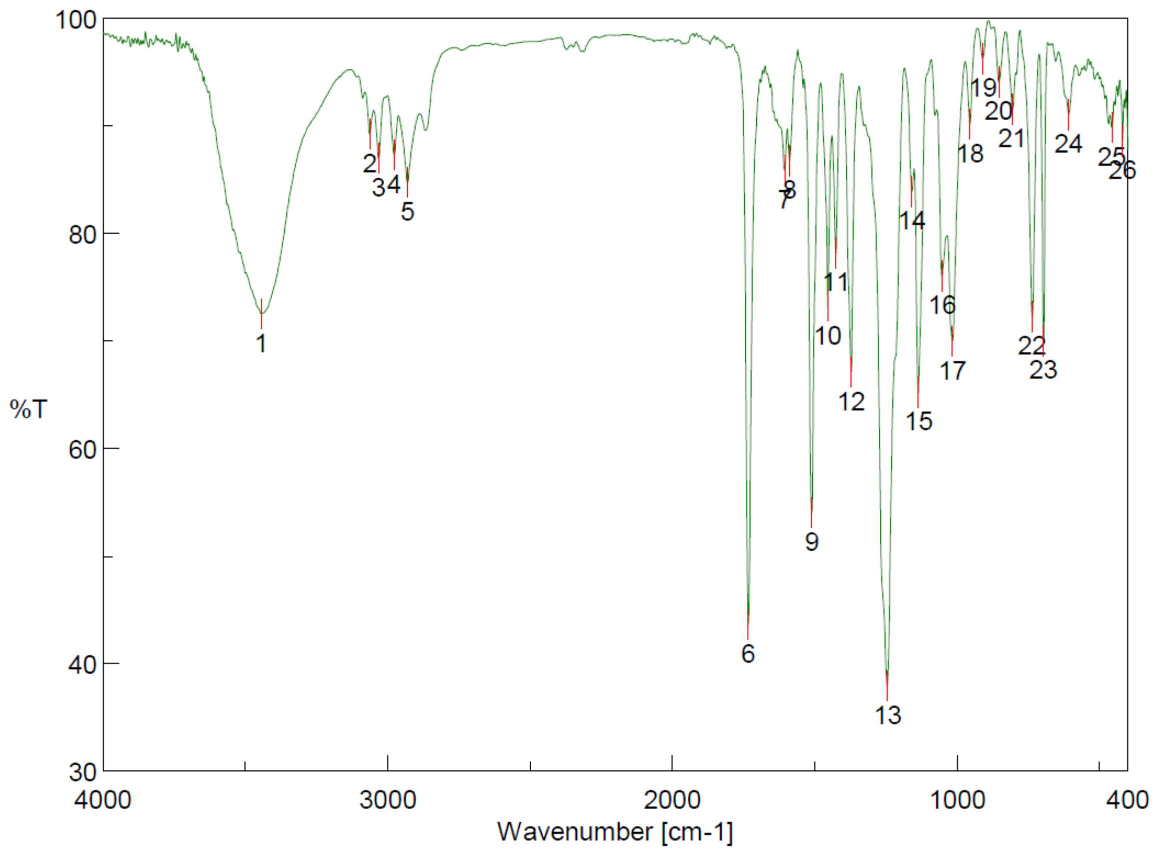

Figure S145. Experimental IR spectrum of (*S*)-**12** recorded as KBr disc.

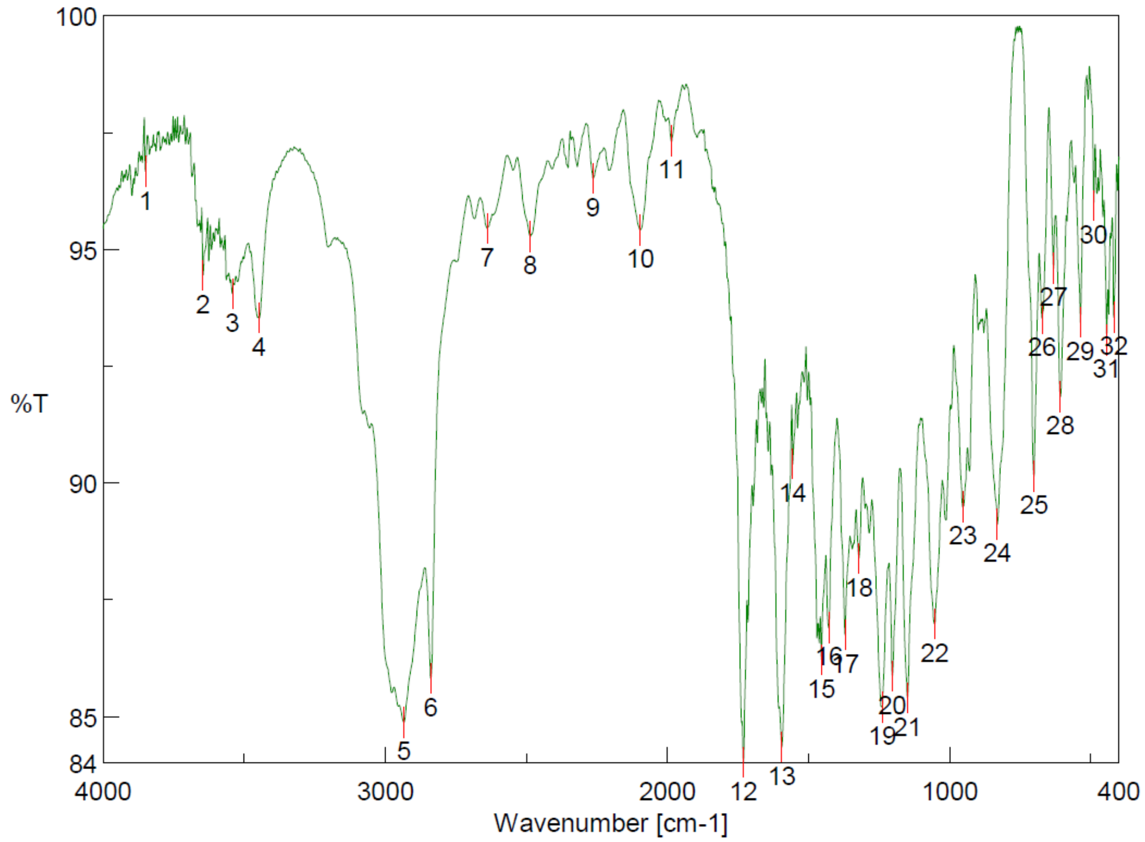

Figure S146. Experimental IR spectrum of (*S*)-**7** recorded as KBr disc.

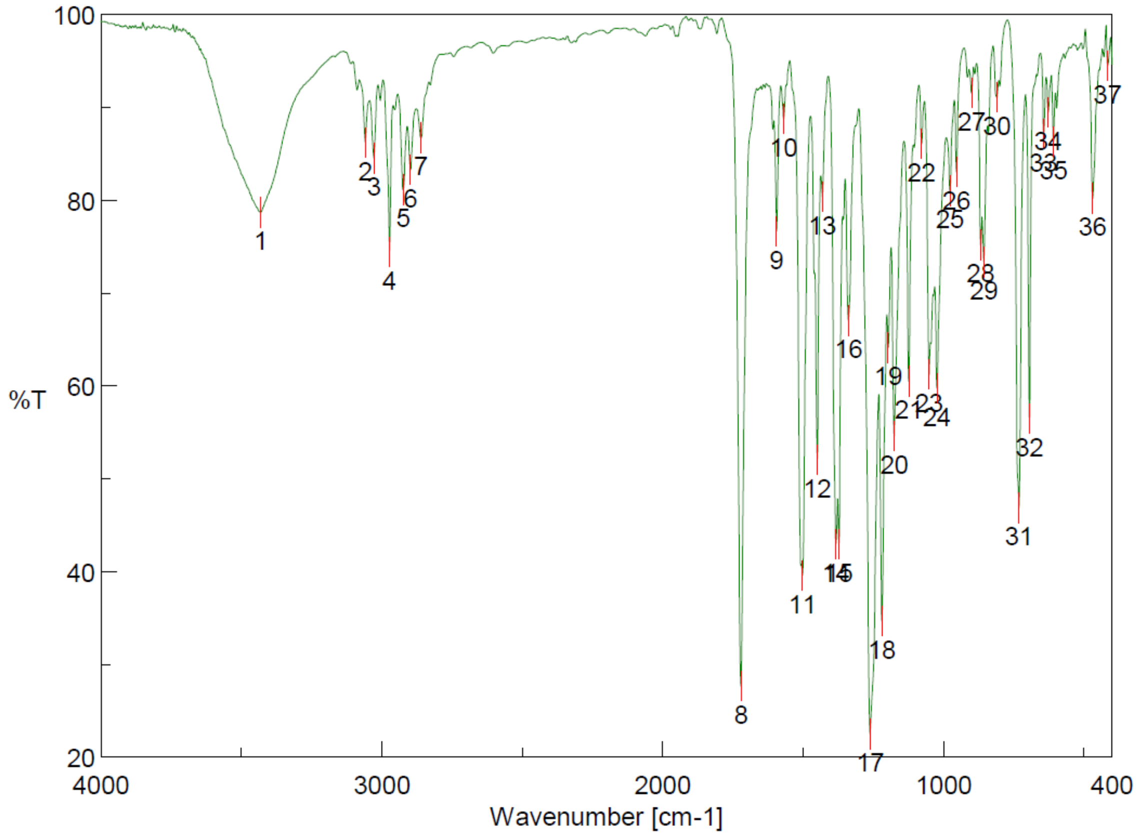

Figure S147. Experimental IR spectrum of (*S*)-**2** recorded as KBr disc.

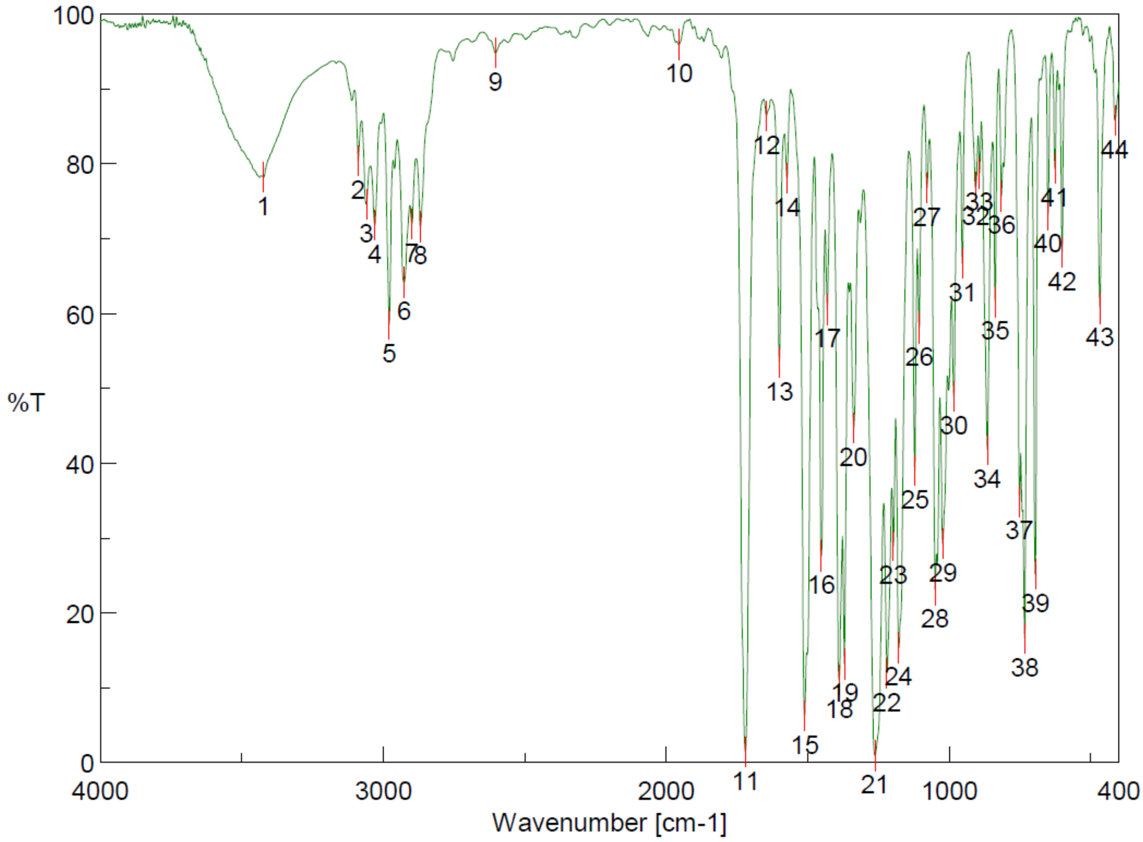

Figure S148. Experimental IR spectrum of (*S*)-**13** recorded as KBr disc.

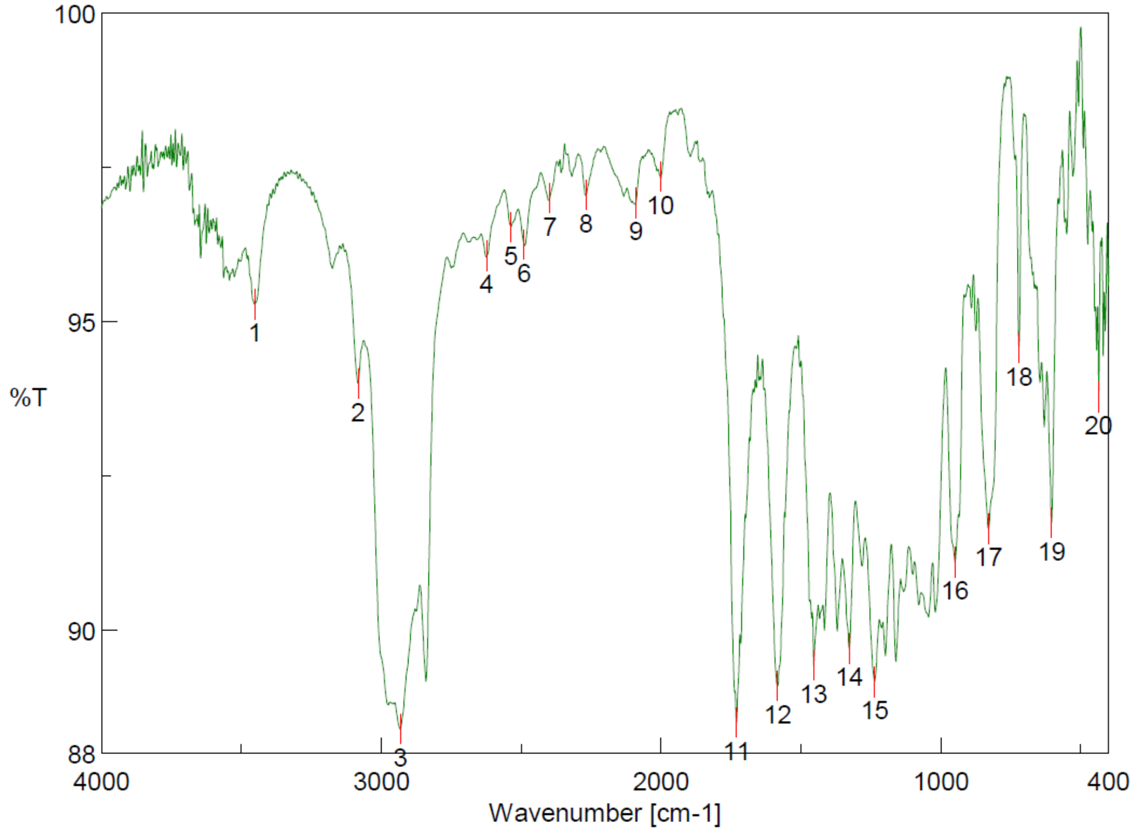

Figure S149. Experimental IR spectrum of (*S*)-**9** recorded as KBr disc.

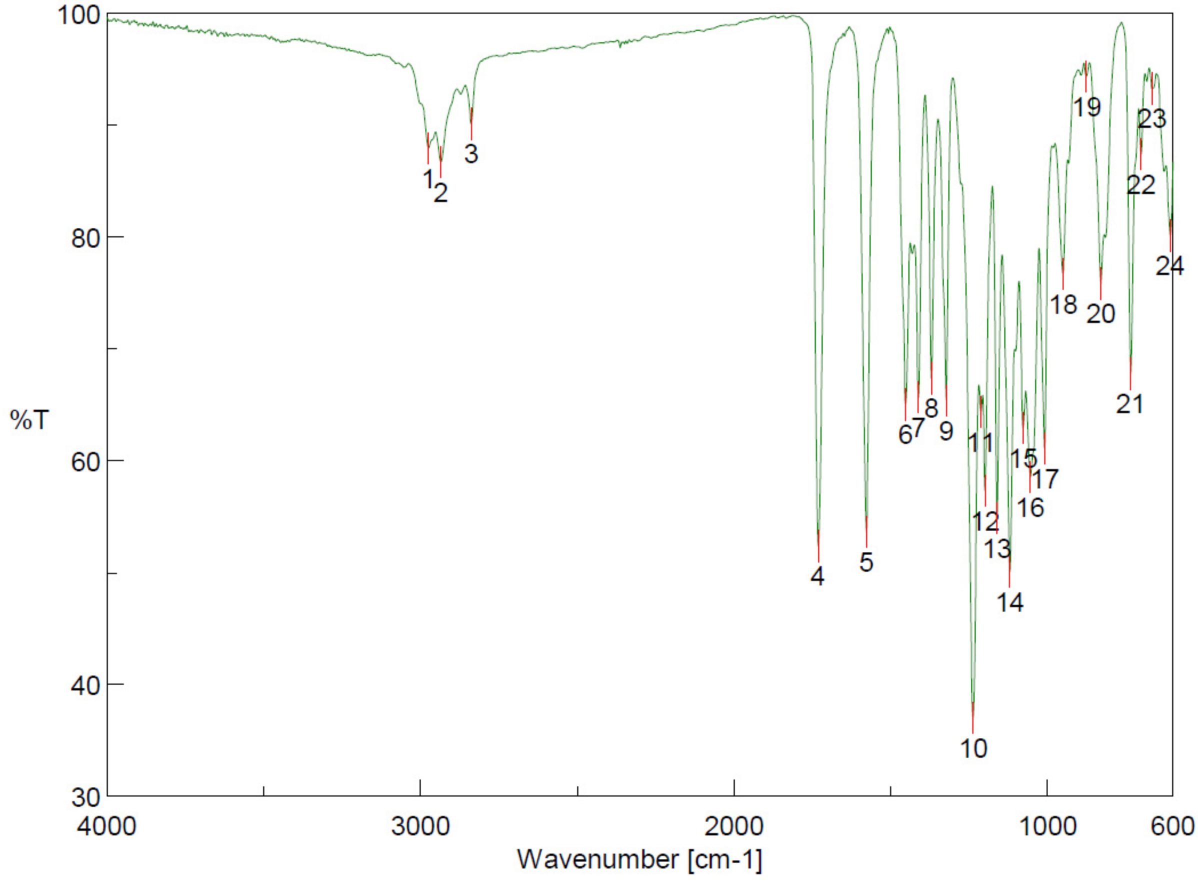

Figure S150. Experimental IR spectrum of (*S*)-**8a** and (*S*)-**8b** recorded as KBr disc.

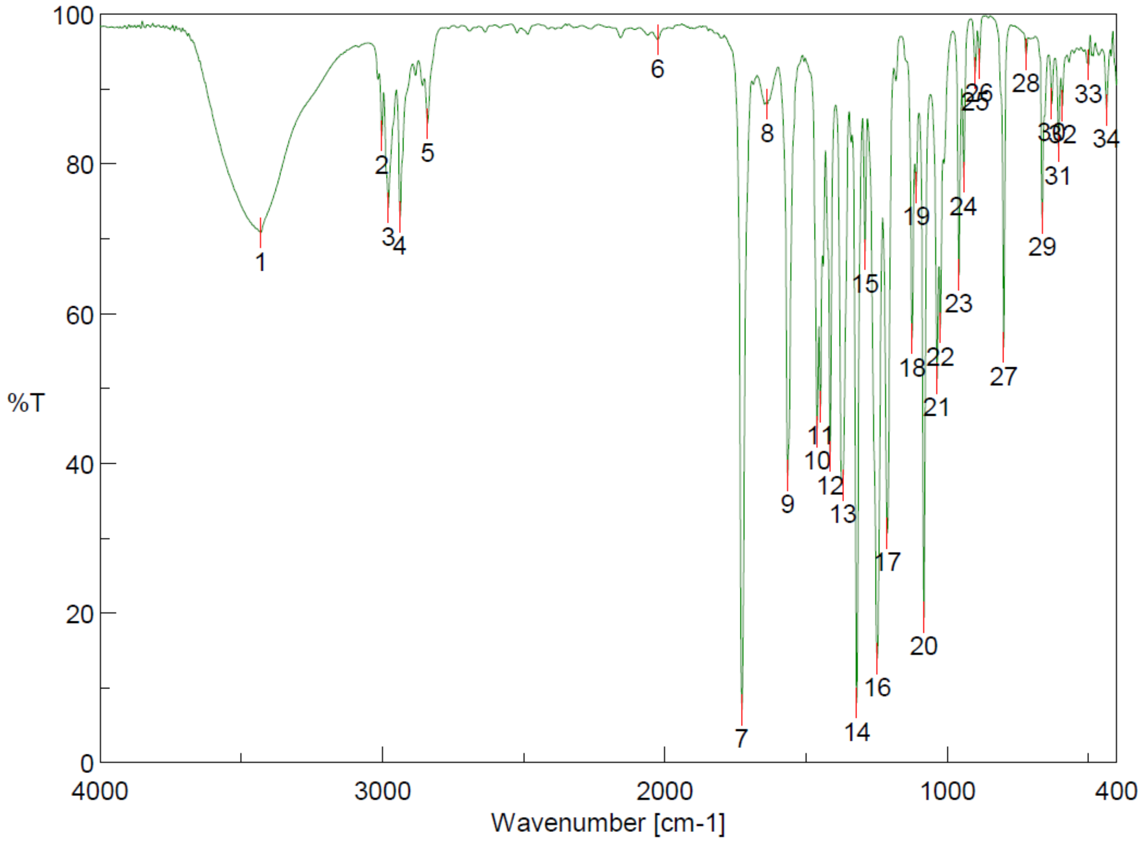

Figure S151. Experimental IR spectrum of (*S*)-**8c** recorded as KBr disc.

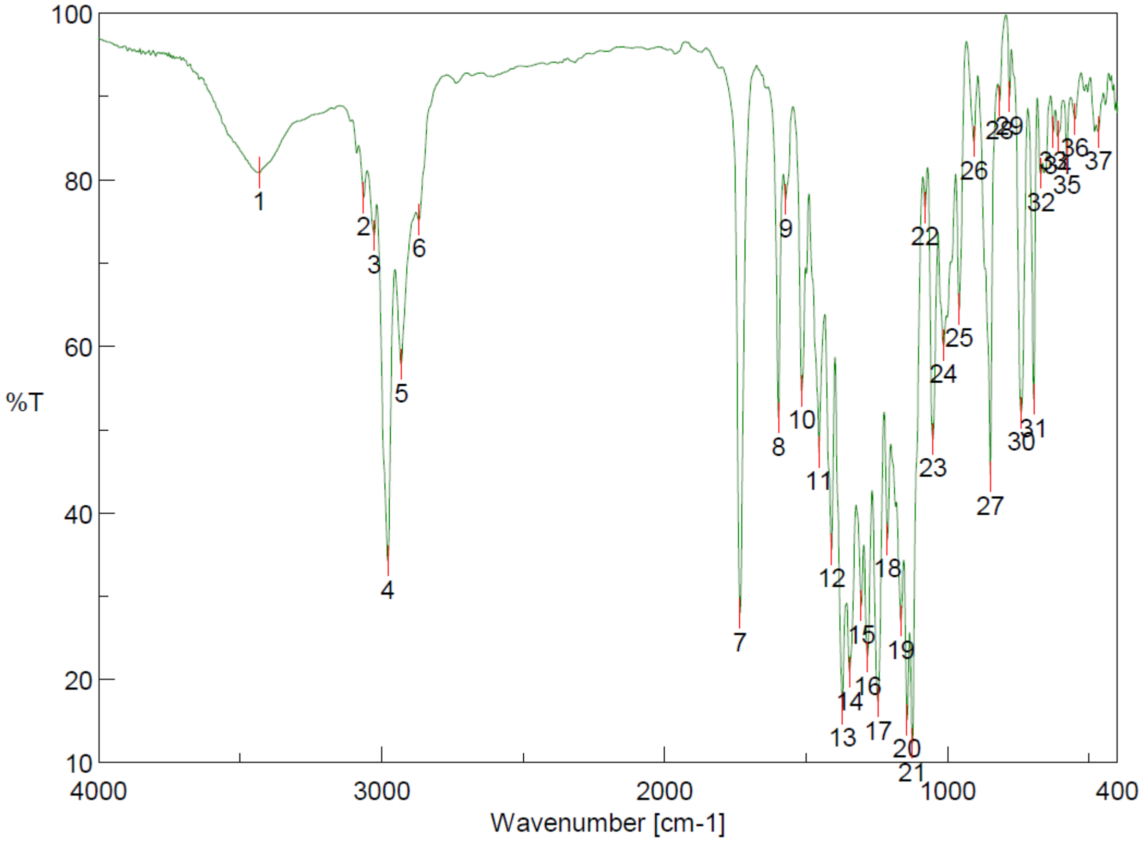

Figure S152. Experimental IR spectrum of (*S*)-**14** recorded as KBr disc.

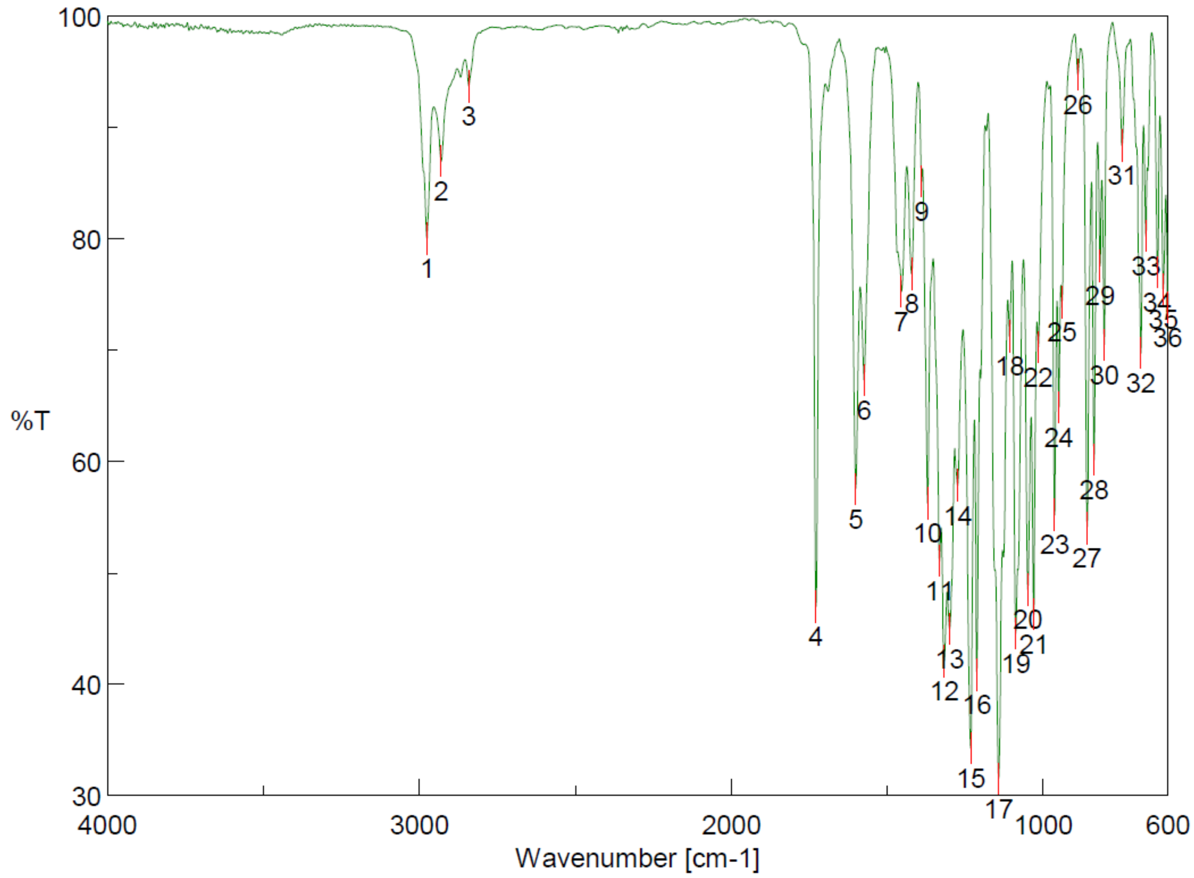

Figure S153. Experimental IR spectrum of (*S*)-**3** recorded as KBr disc.

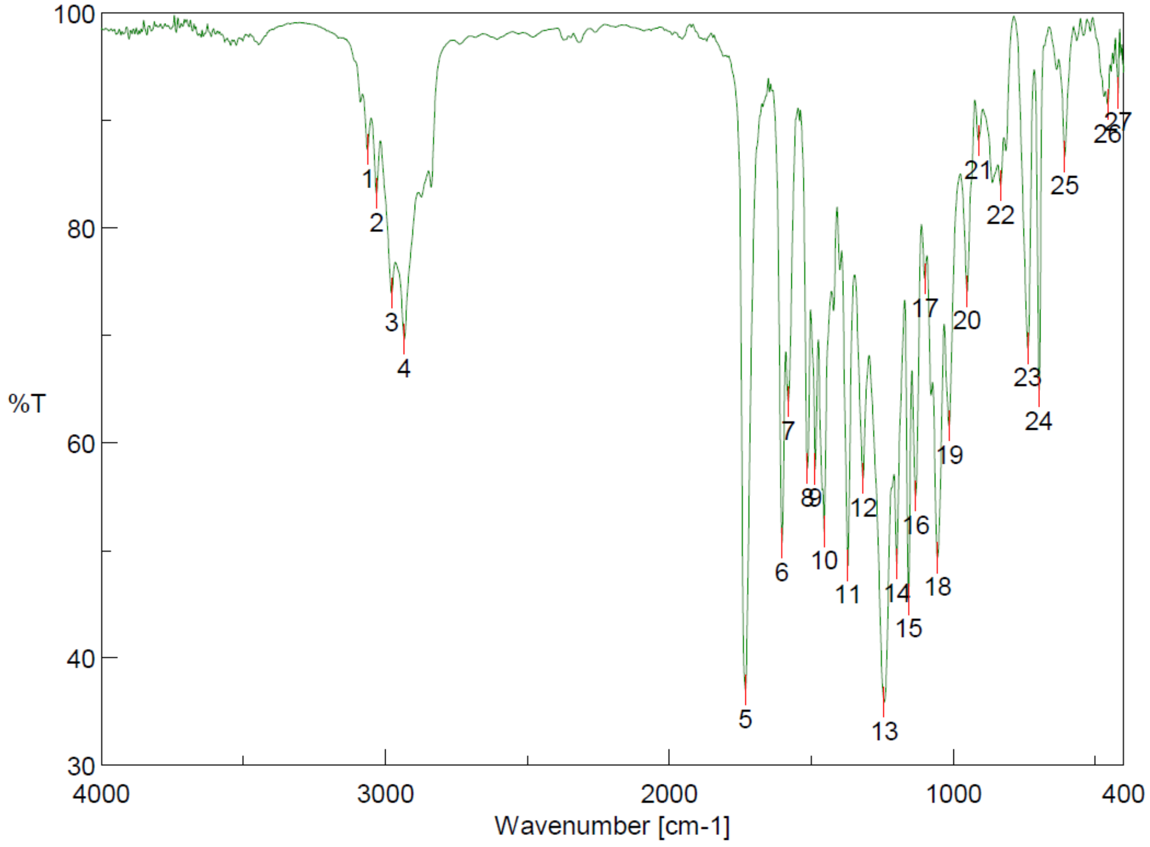

Figure S154. Experimental IR spectrum of (a*S*,2*S*,2'*S*)-**15** recorded as KBr disc.

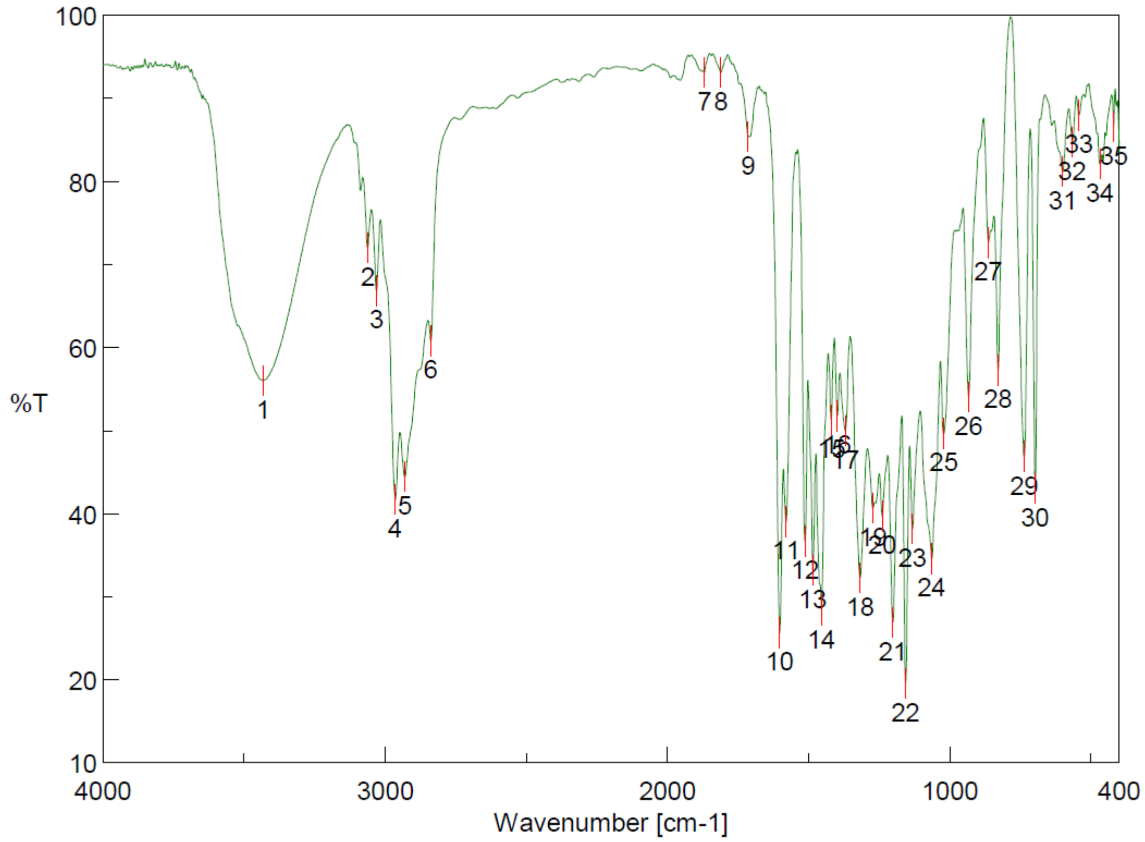

Figure S155. Experimental IR spectrum of (a*S*,2*S*,2'*S*)-**16** recorded as KBr disc.

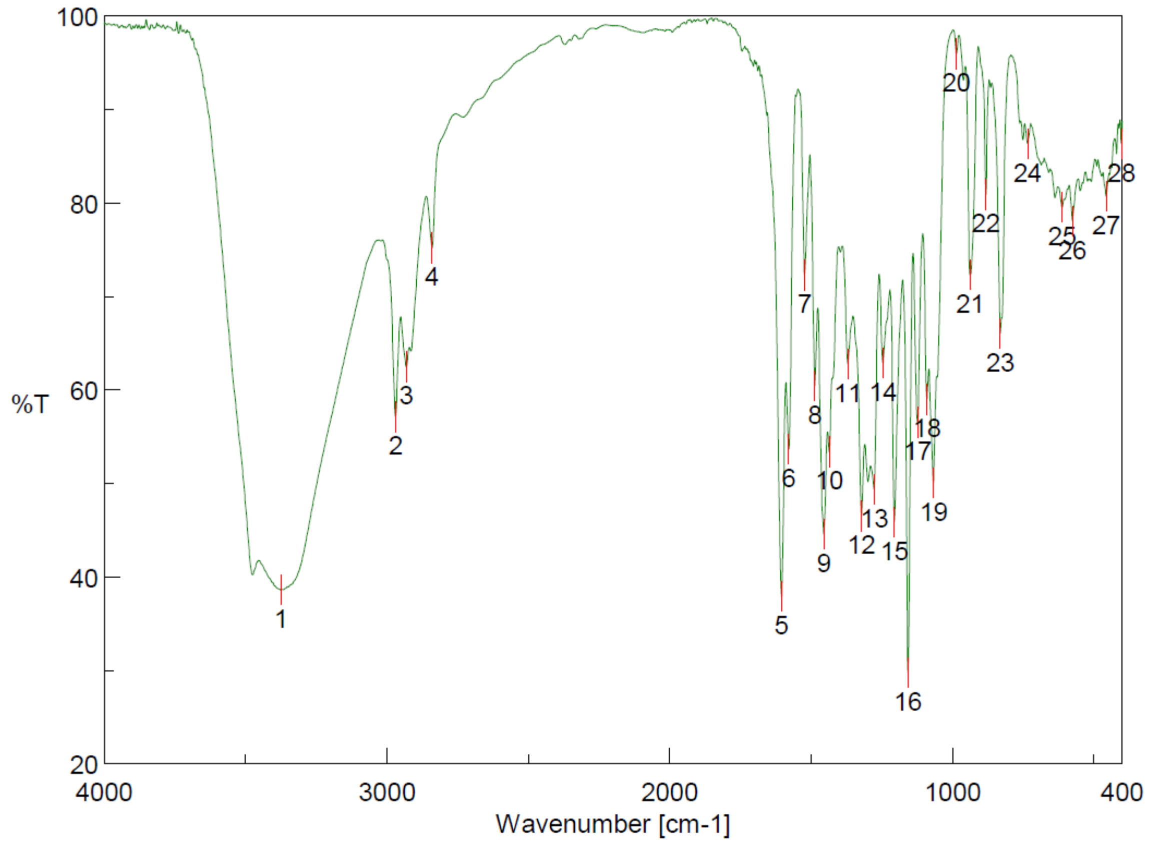

Figure S156. Experimental IR spectrum of (a*S*,2*S*,2'*S*)-**17** recorded as KBr disc.

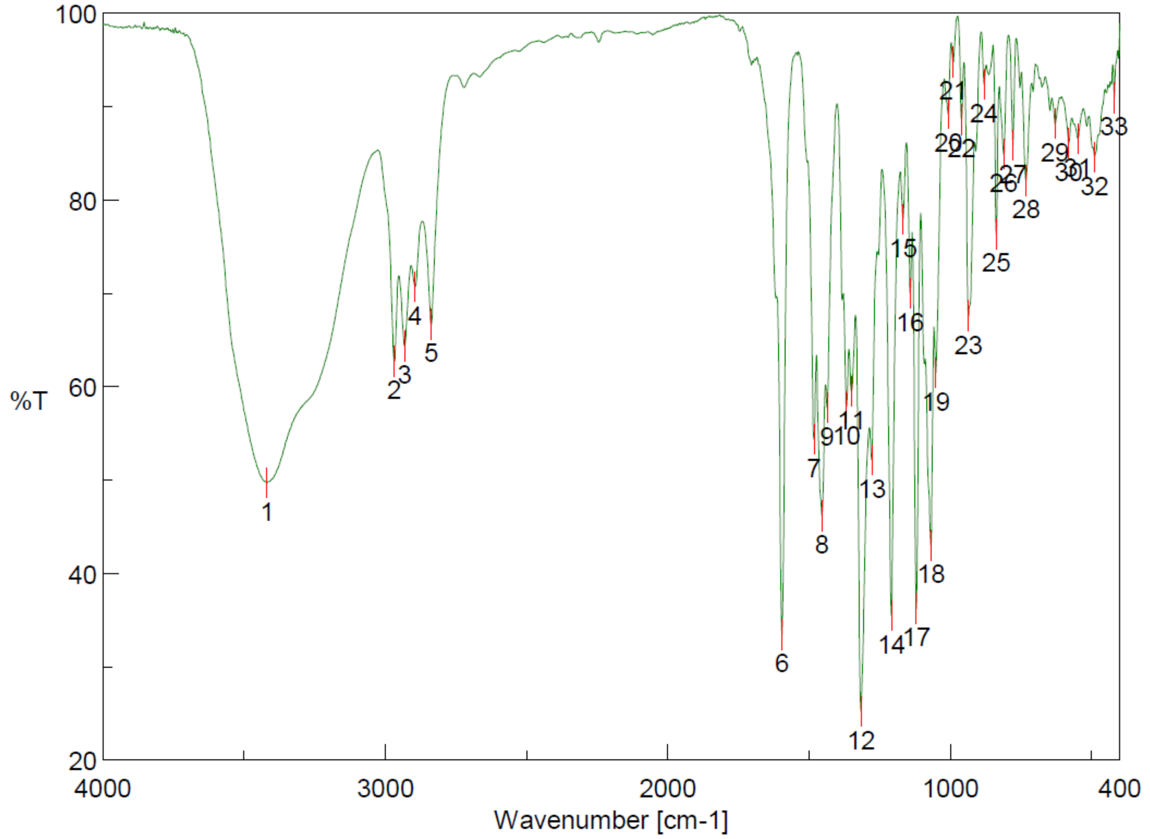

Figure S157. Experimental IR spectrum of (a*S*,3*S*,3’*S*)-**19** recorded as KBr disc.

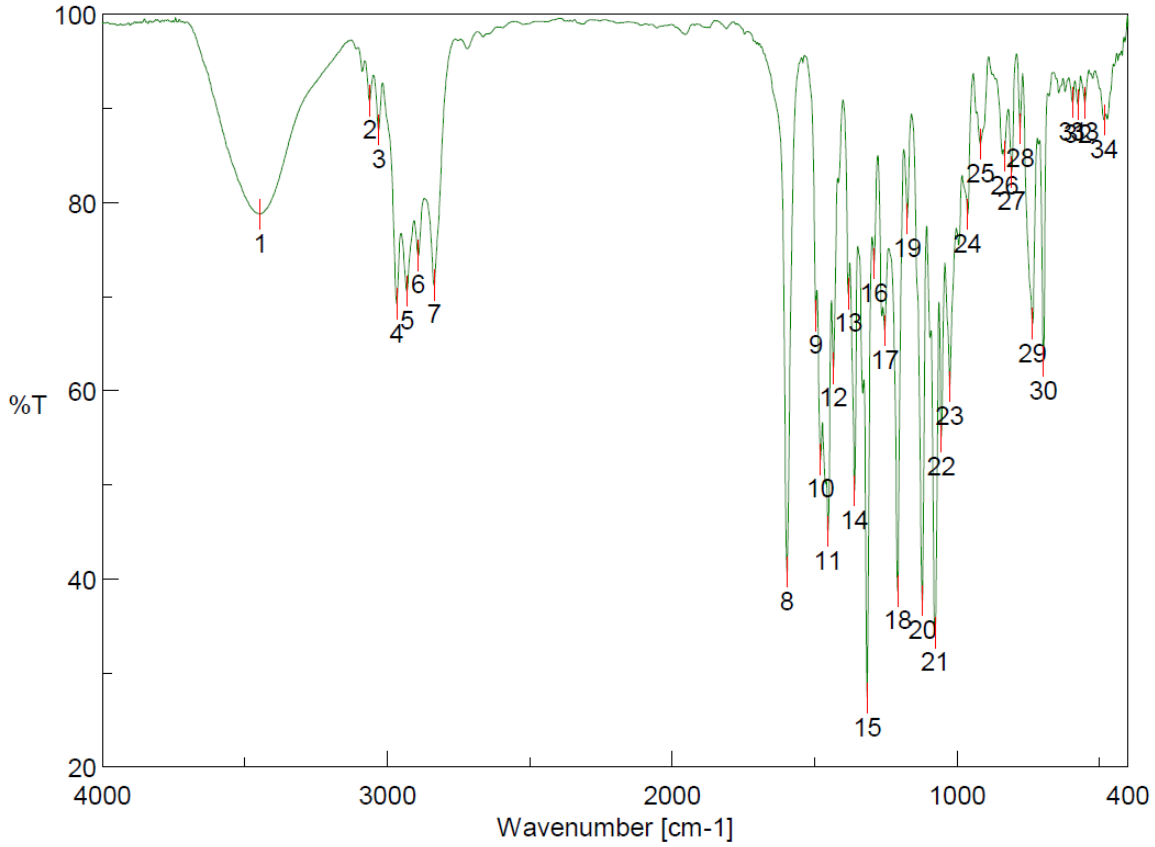

Figure S158. Experimental IR spectrum of (a*S*,3*S*,3’*S*)-**18** recorded as KBr disc.

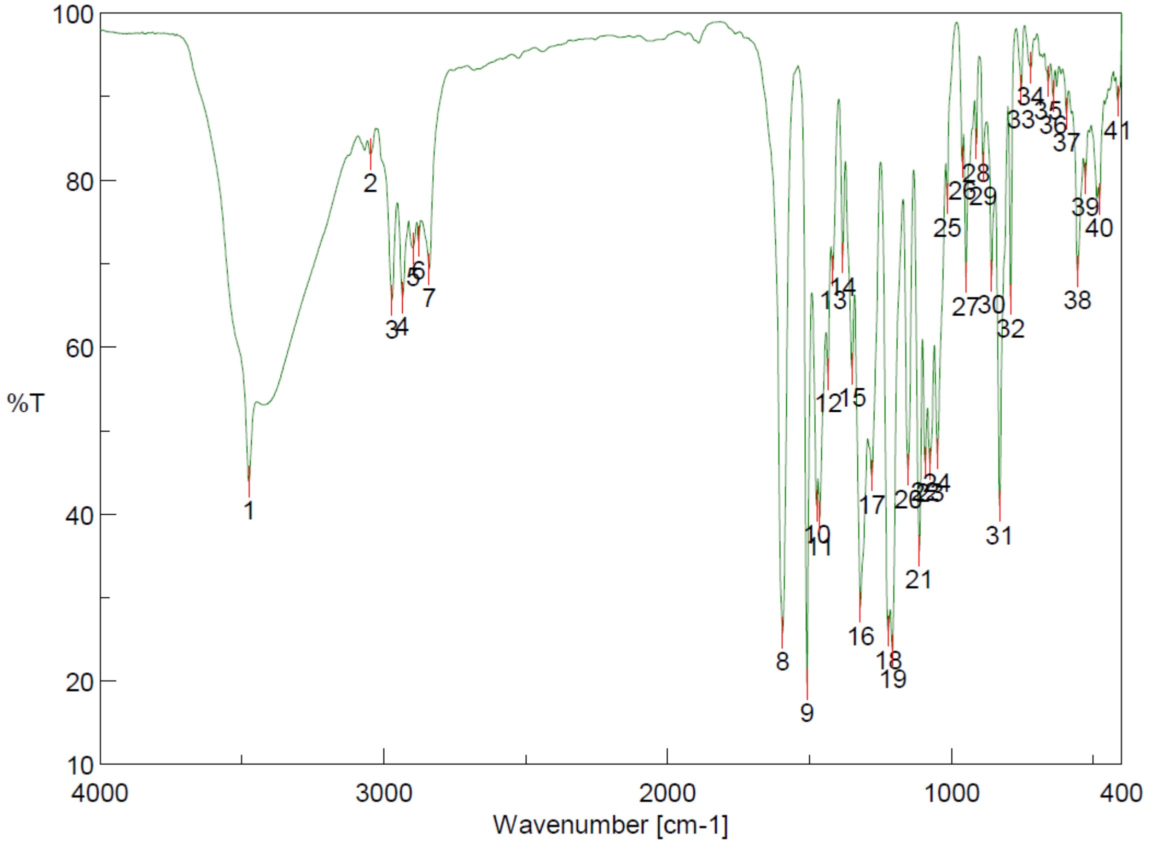

Figure S159. Experimental IR spectrum of *cis*,*cis*-(a*S*,1*R*,3*S*,1'*R*,3'*S*)-**20** recorded as KBr disc.

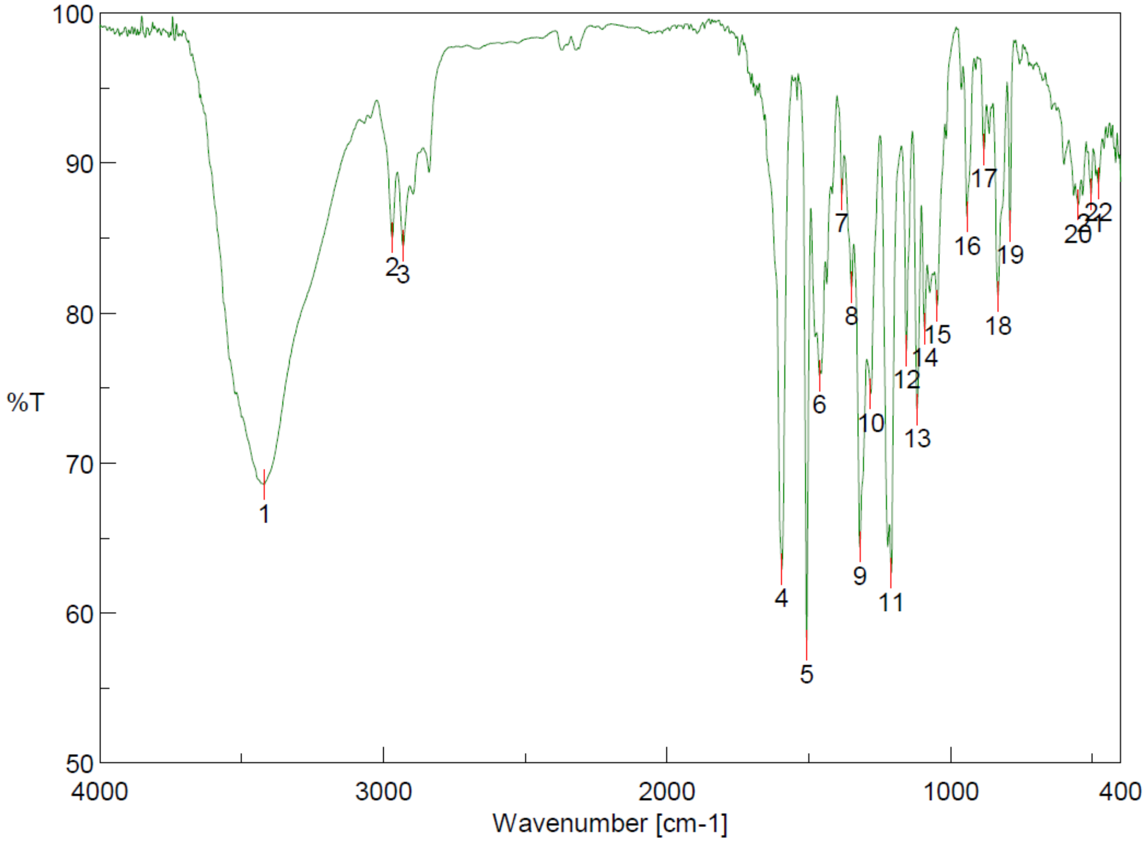

Figure S160. Experimental IR spectrum of *trans*,*cis*-(a*S*,1*S*,3*S*,1'*R*,3'*S*)-**20** recorded as KBr disc.

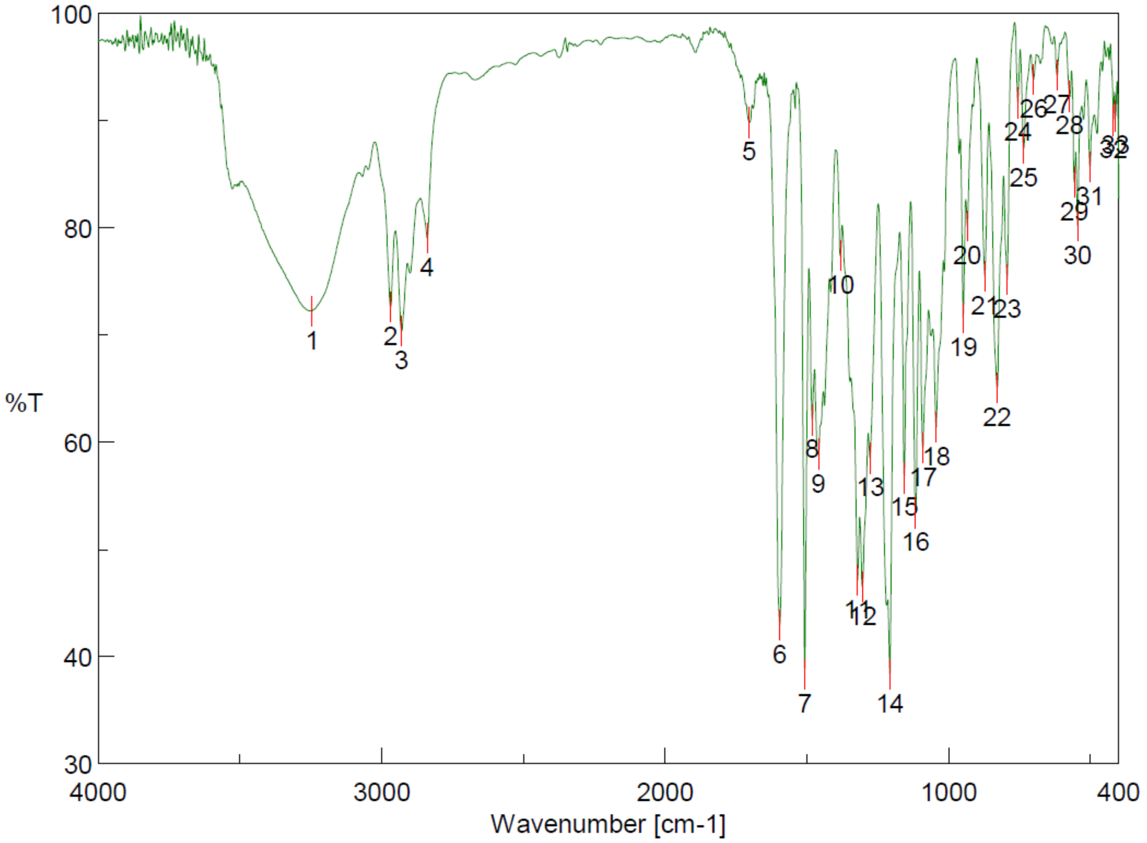

Figure S161. Experimental IR spectrum of *cis*,*trans*-(a*S*,1*R*,3*S*,1'*S*,3'*S*)-**20** recorded as KBr disc.

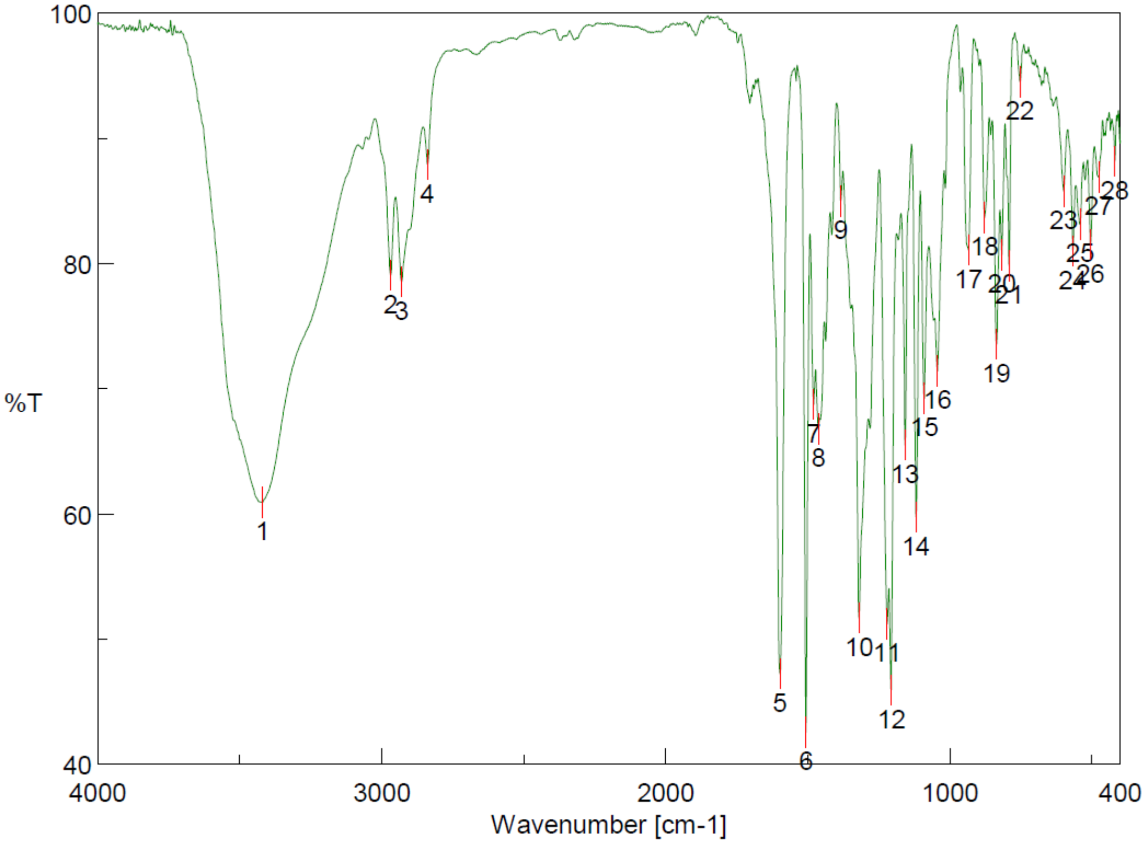

Figure S162. Experimental IR spectrum of *trans*,*trans*-(a*S*,1*S*,3*S*,1'*S*,3'*S*)-**20** recorded as KBr disc.

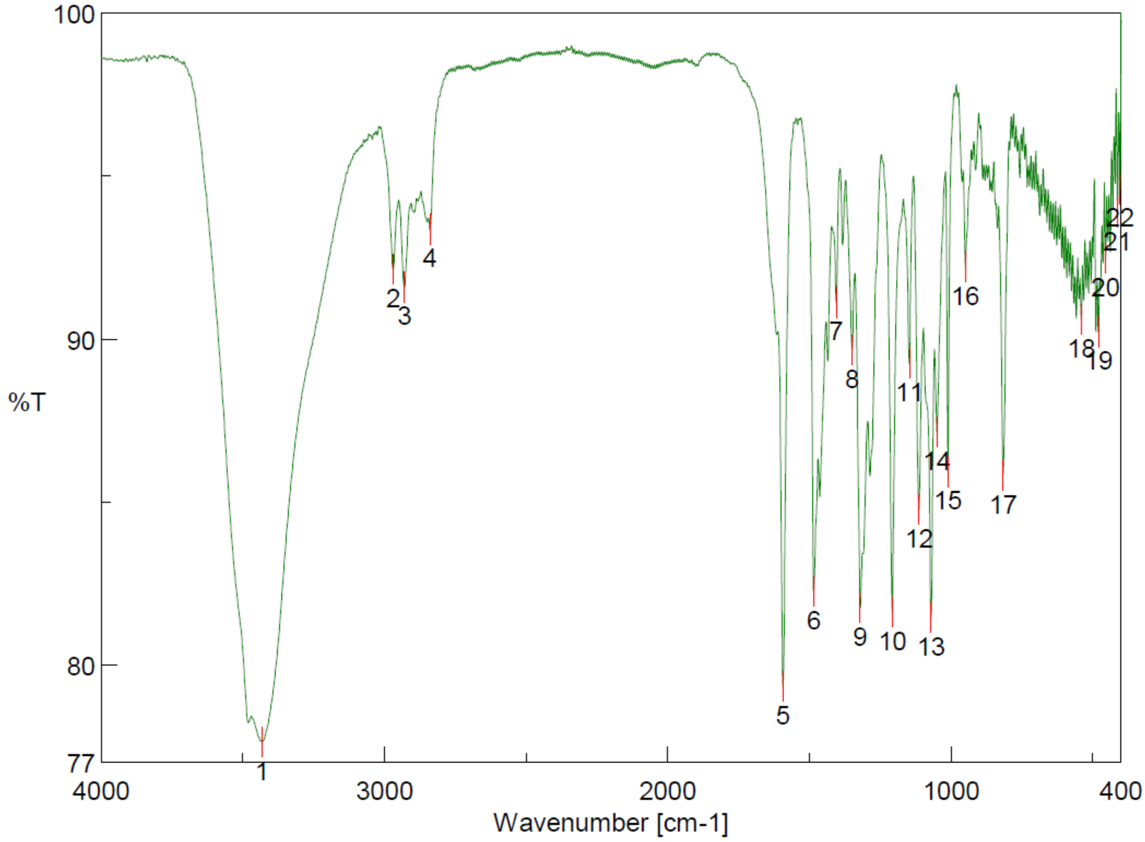

Figure S163. Experimental IR spectrum of *cis*,*cis*-(a*S*,1*R*,3*S*,1’*R*,3’*S*)-**21** recorded as KBr disc.

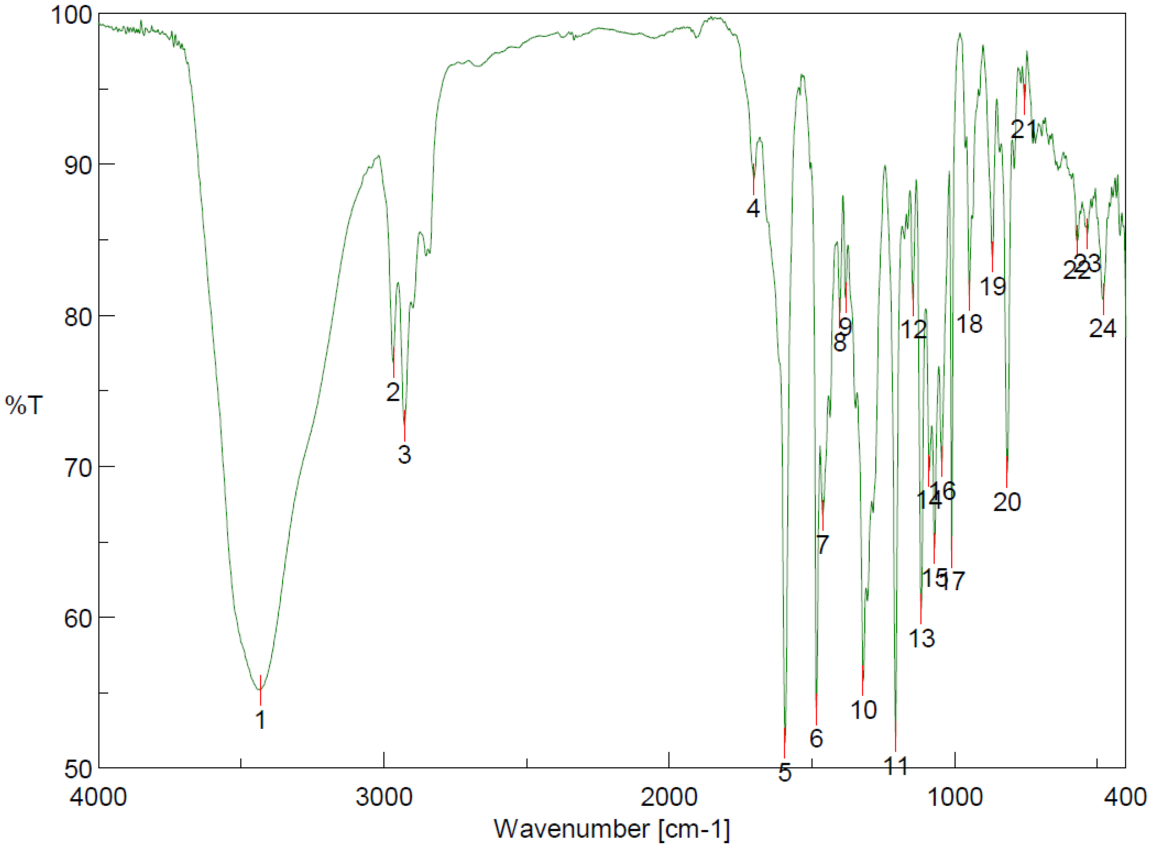

Figure S164. Experimental IR spectrum of *cis*,*trans*-(a*S*,1*R*,3*S*,1’*S*,3’*S*)-**21** recorded as KBr disc.

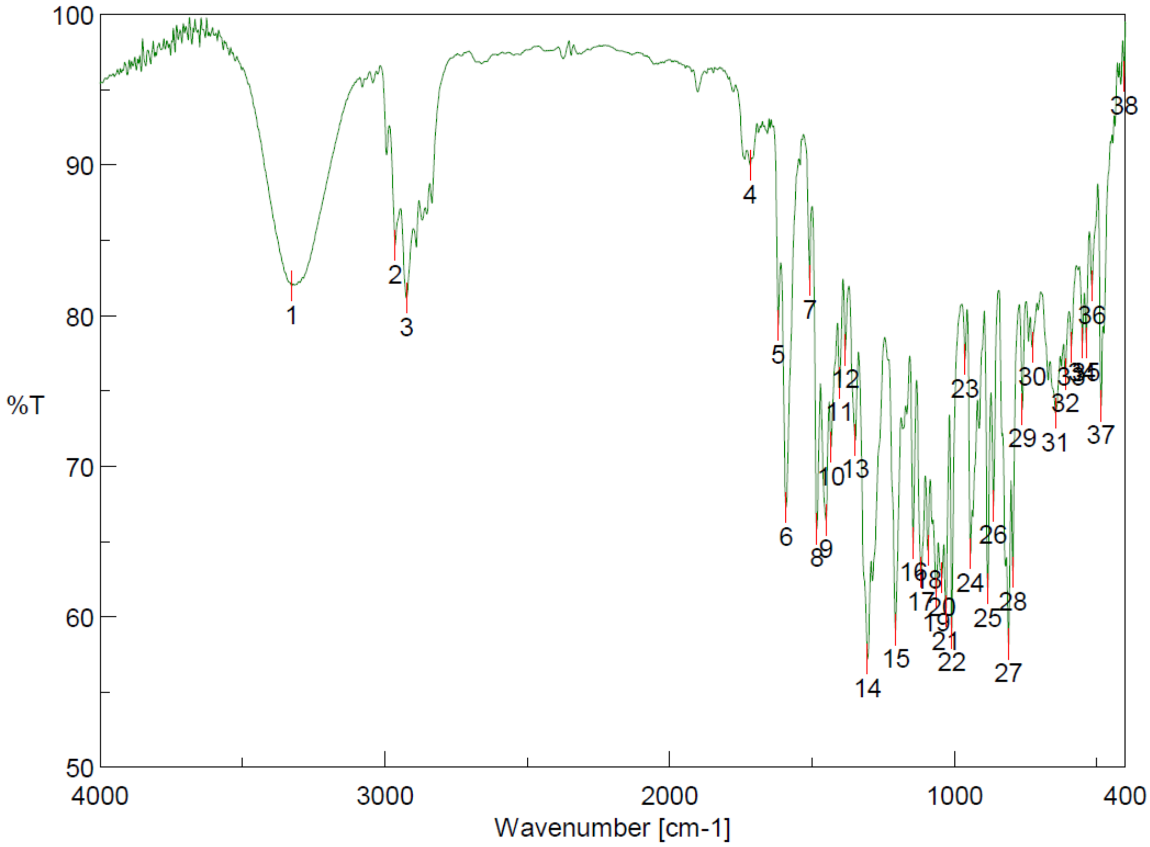

Figure S165. Experimental IR spectrum of *trans*,*cis*-(a*S*,1*S*,3*S*,1'*R*,3'*S*)-**21** recorded as KBr disc.

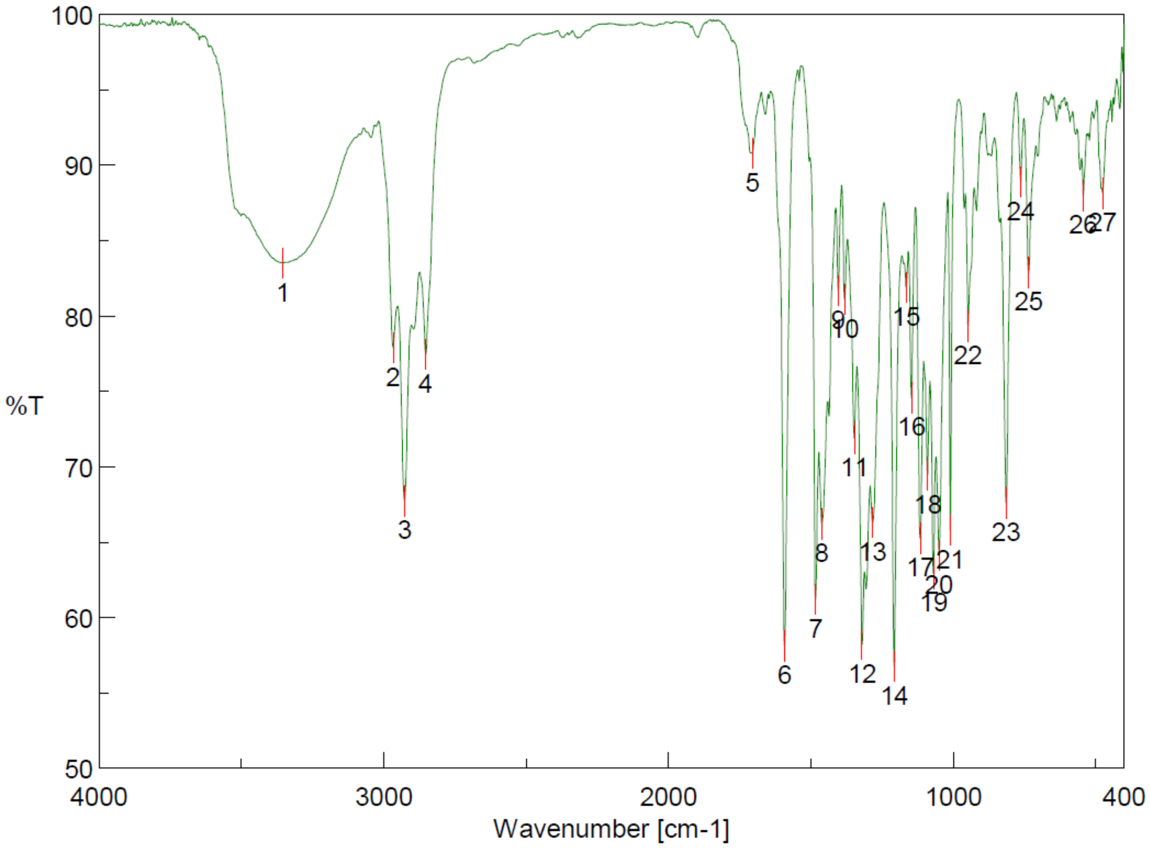

Figure S166. Experimental IR spectrum of *cis*,*cis*-(a*R*,1*R*,3*S*,1'*R*,3'*S*)-**21** recorded as KBr disc.

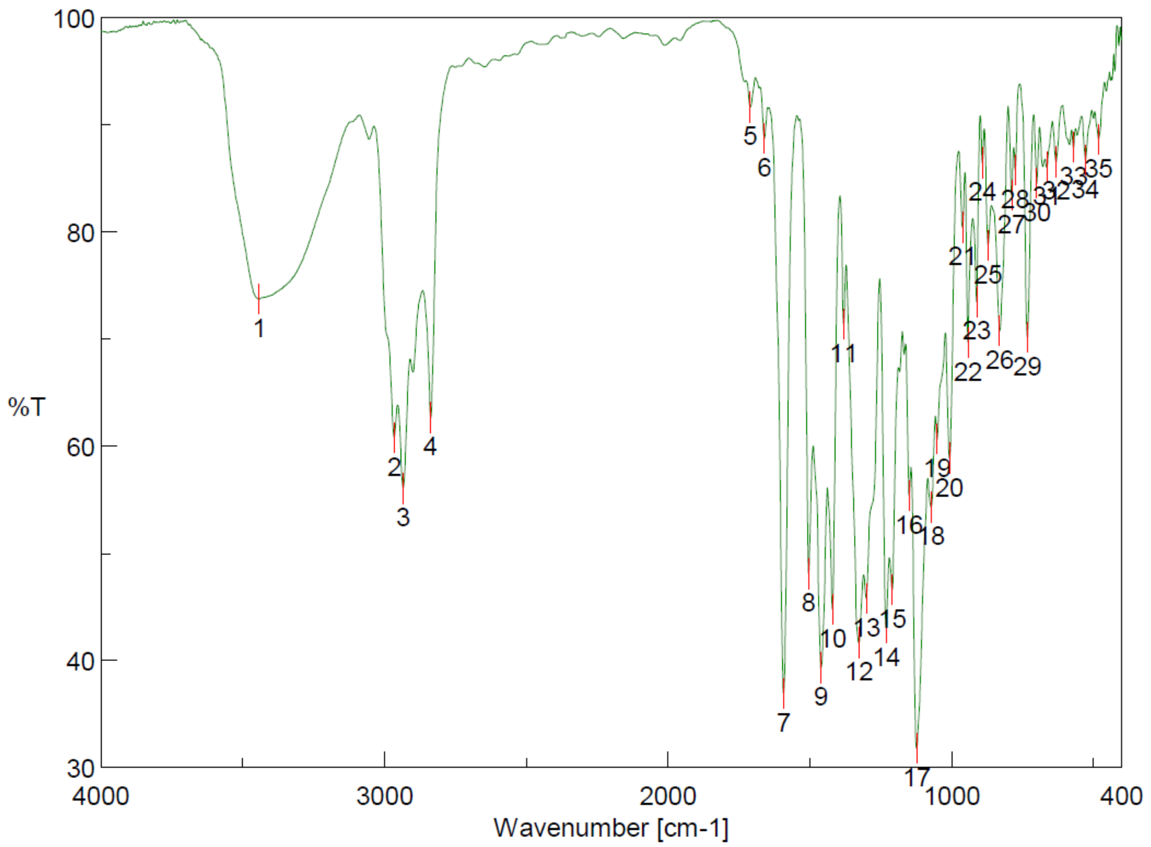

Figure S167. Experimental IR spectrum of *cis*,*cis*-(a*S*,1*R*,3*S*,1'*R*,3'*S*)-**22** recorded as KBr disc.

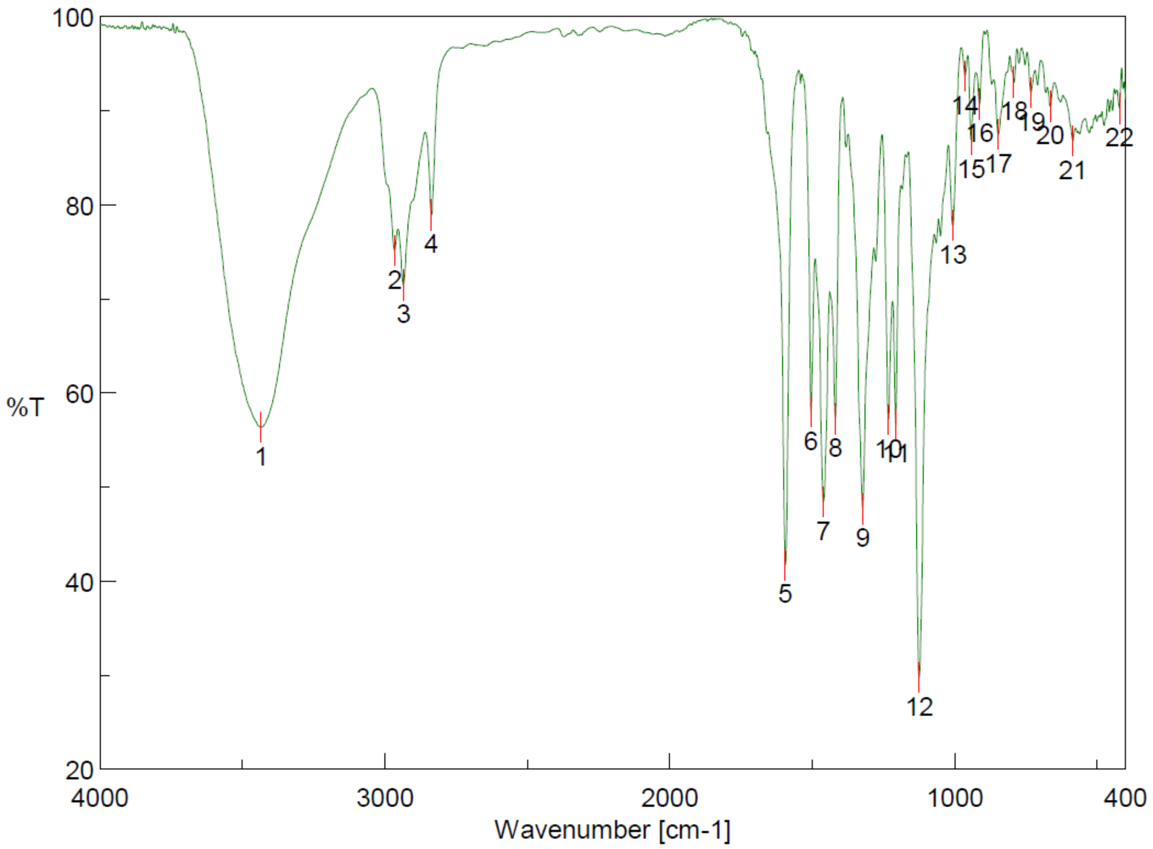

Figure S168. Experimental IR spectrum of *cis*,*trans*-(a*S*,1*R*,3*S*,1'*S*,3'*S*)-**22** recorded as KBr disc.

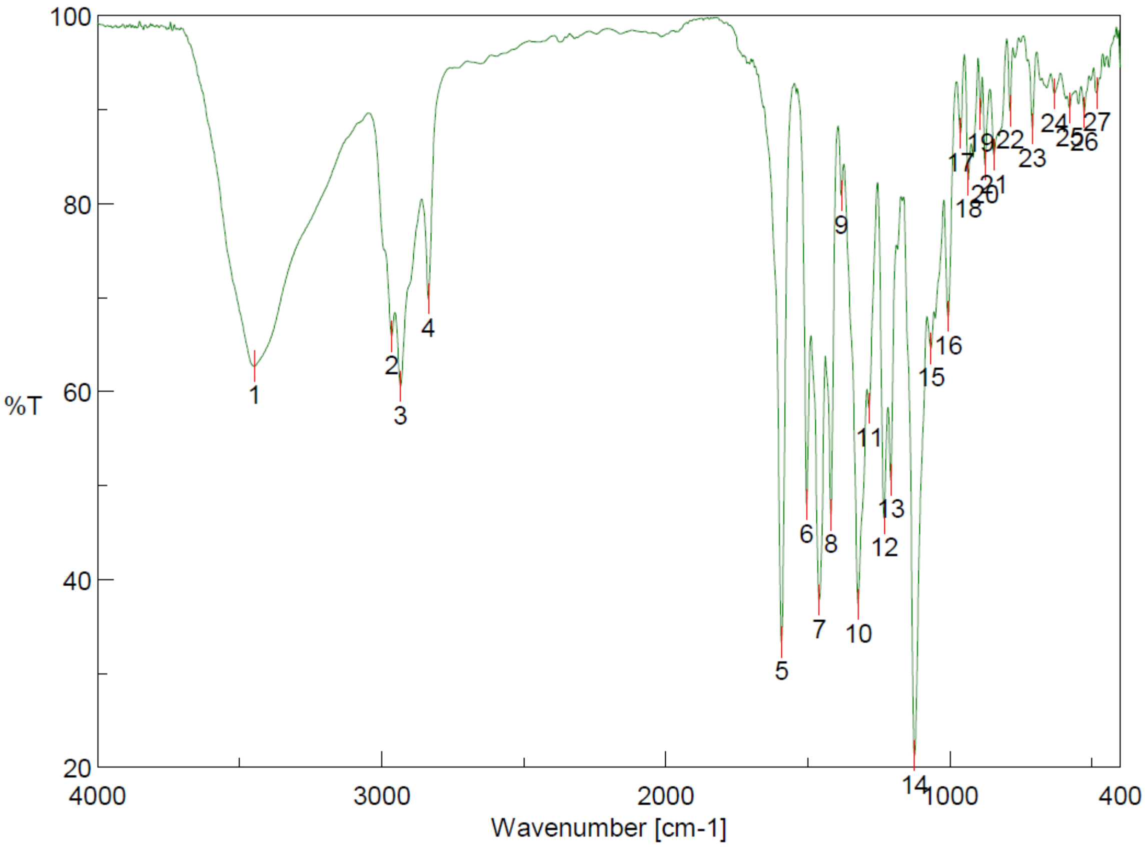

Figure S169. Experimental IR spectrum of *trans*,*cis*-(a*S*,1*S*,3*S*,1’*R*,3’*S*)-**22** recorded as KBr disc.

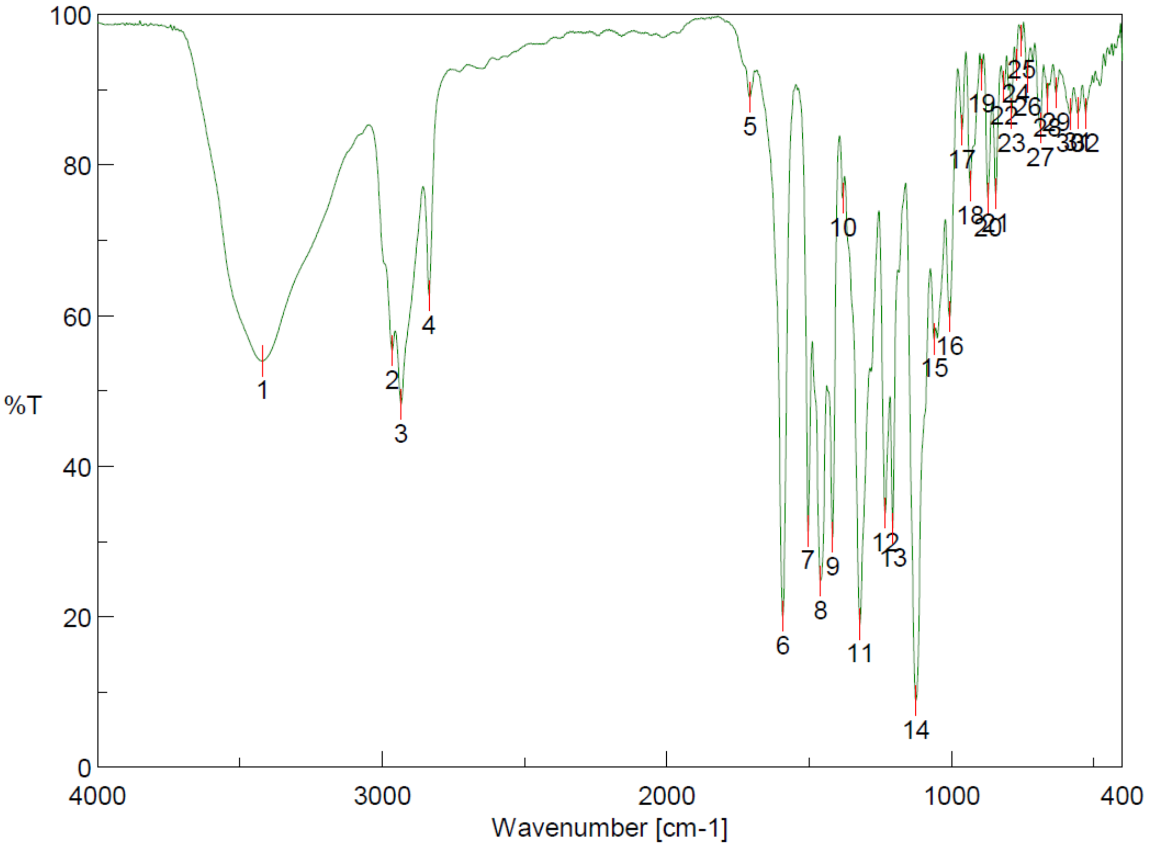

Figure S170. Experimental IR spectrum of *trans*,*trans*-(a*S*,1*S*,3*S*,1’*S*,3’*S*)-**22** recorded as KBr disc.

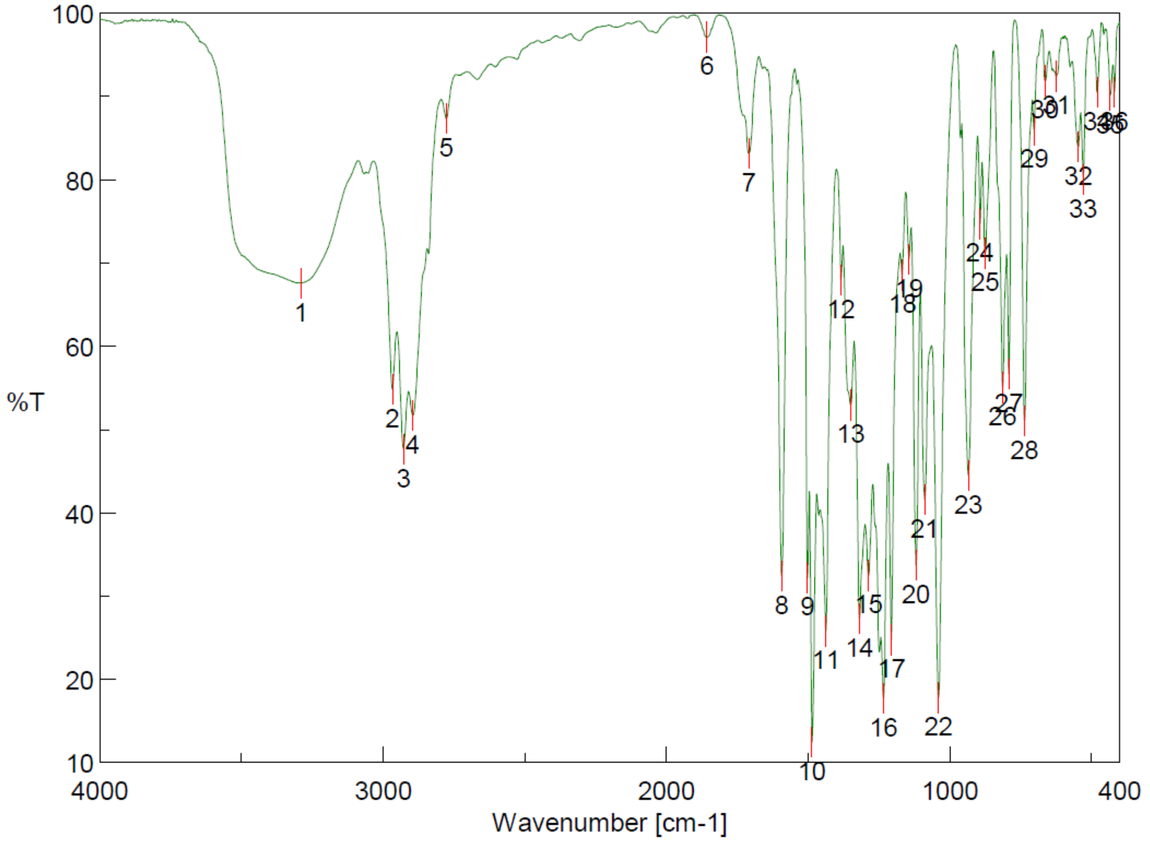

Figure S171. Experimental IR spectrum of *trans*,*trans*-(a*R*,1*S*,3*S*,1'*S*,3'*S*)-**23** recorded as KBr disc.

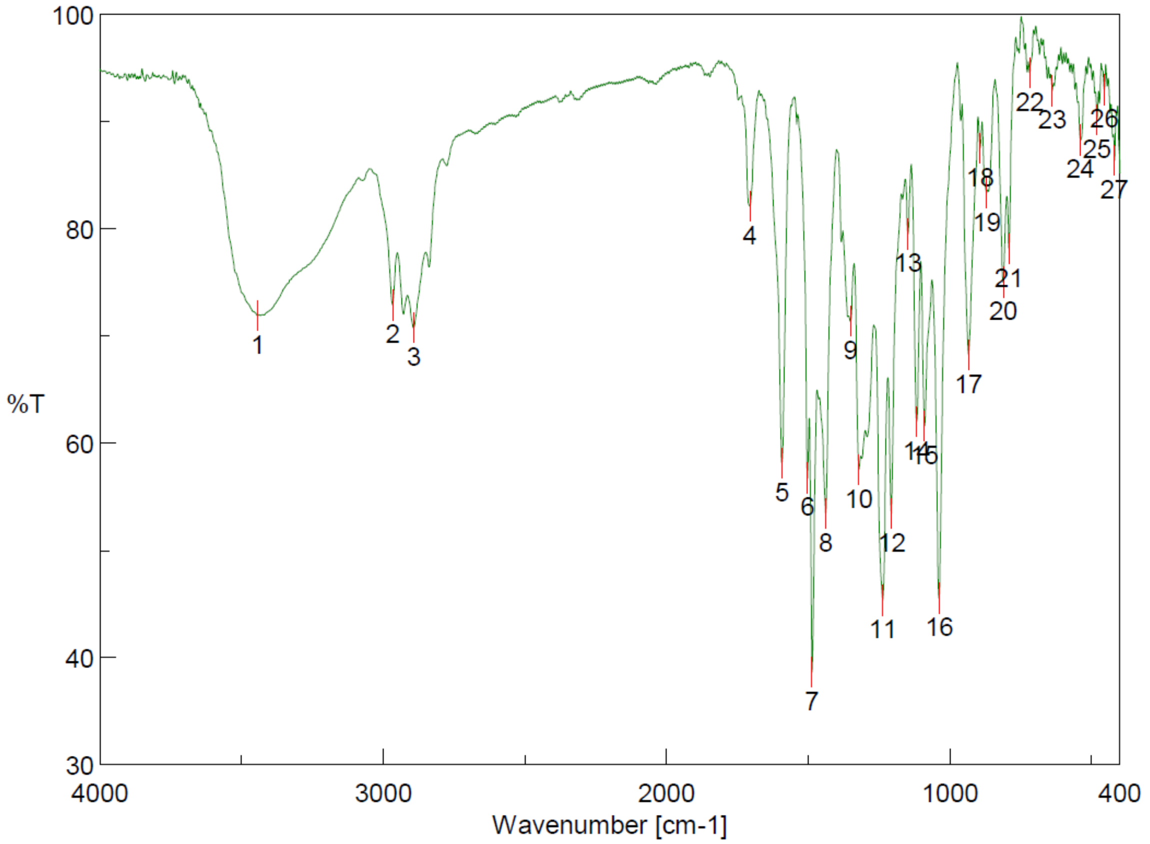

Figure S172. Experimental IR spectrum of *trans*,*cis*-(a*S*,1*S*,3*S*,1'*R*,3'*S*)-**23** recorded as KBr disc.

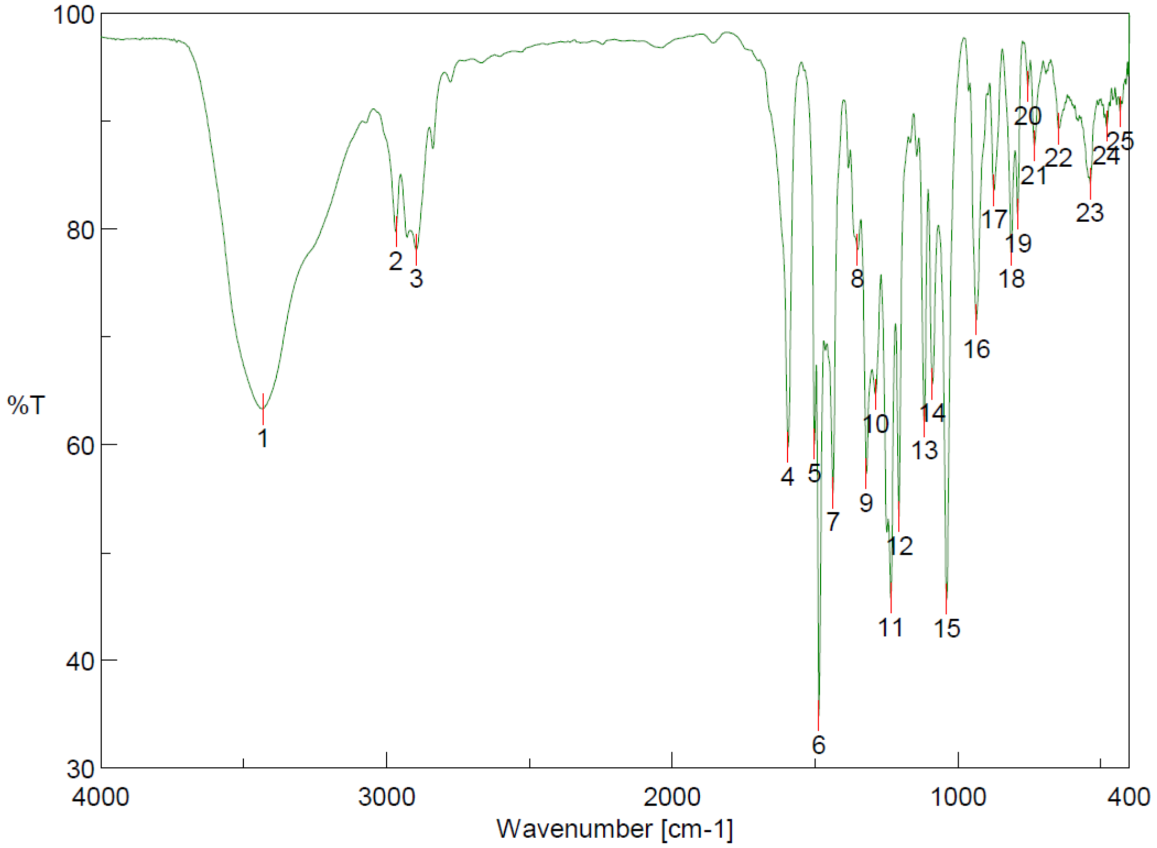

Figure S173. Experimental IR spectrum of *trans*,*trans*-(a*S*,1*S*,3*S*,1’*S*,3’*S*)-**23** recorded as KBr disc.

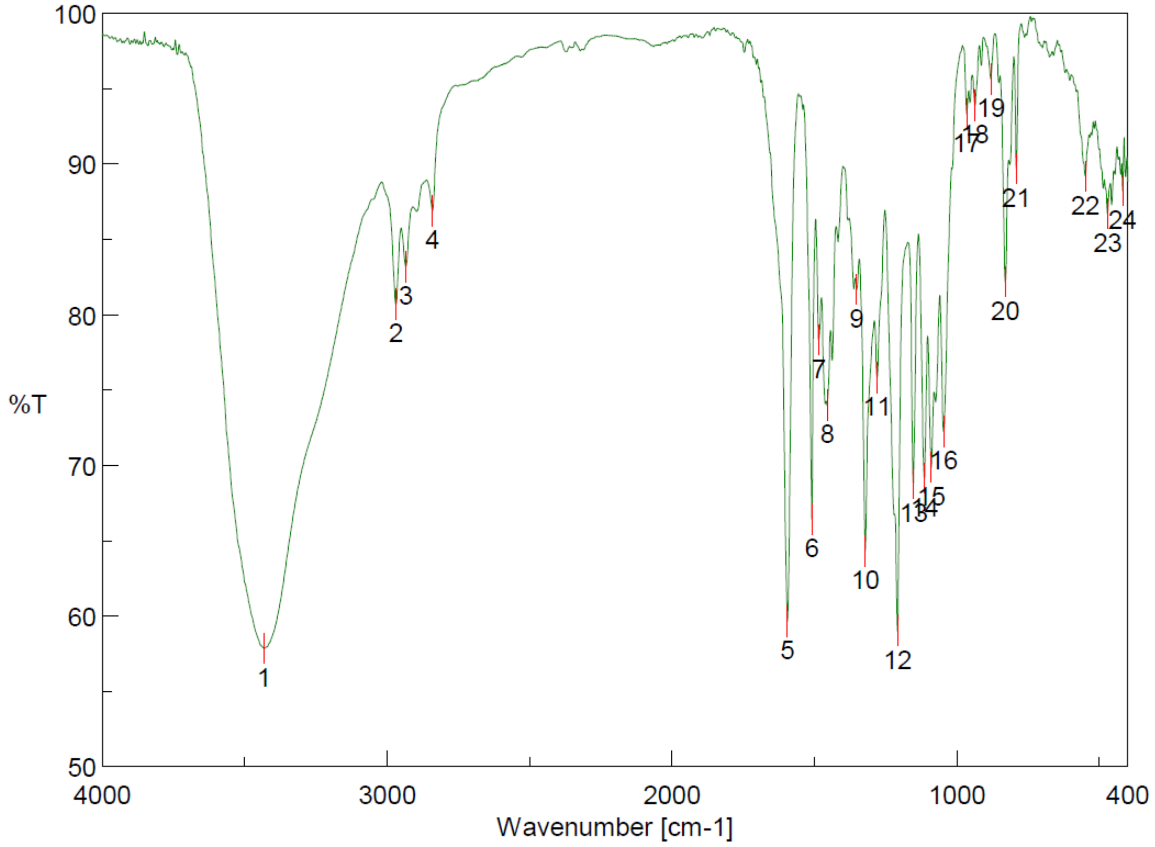

Figure S174. Experimental IR spectrum of *cis*-(a*S*,2*S*,1’*R*,3’*S*)-**24** recorded as KBr disc.

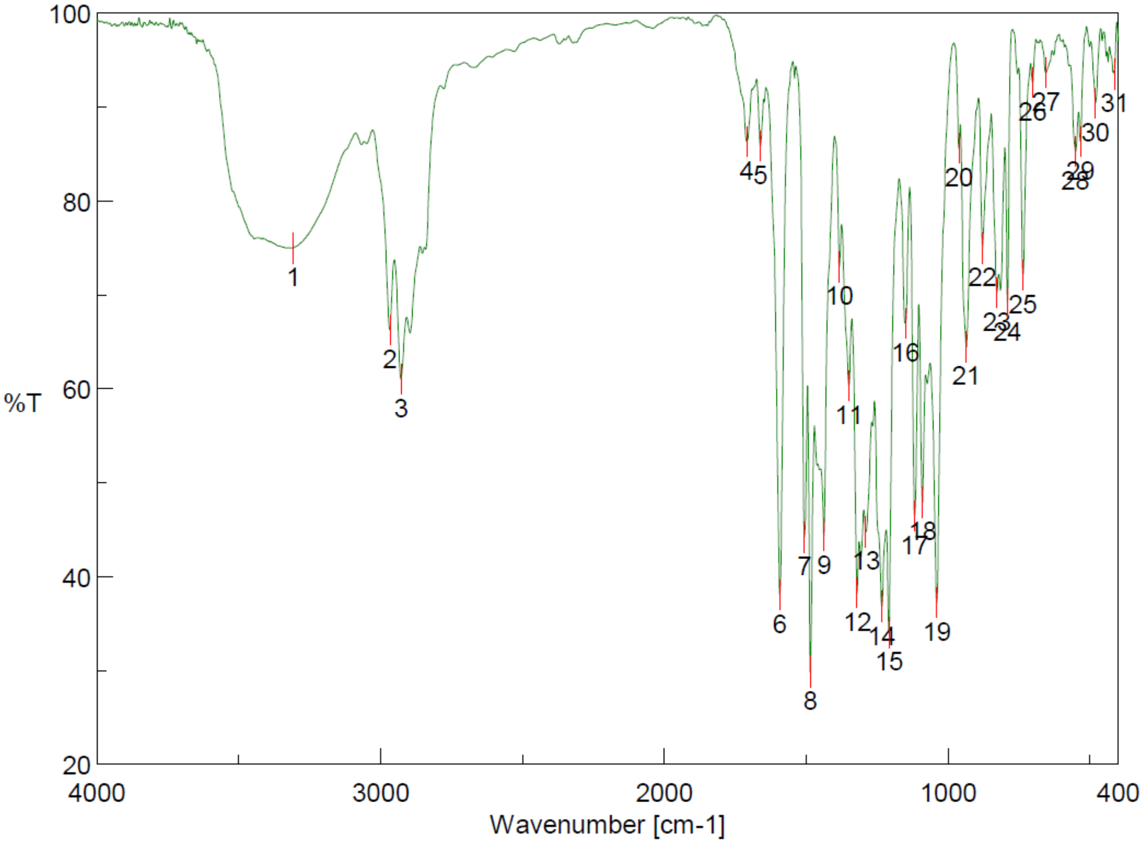

Figure S175. Experimental IR spectrum of *trans*,*cis*-(a*S*,1*S*,3*S*,1'*R*,3'*S*)-**25** recorded as KBr disc.

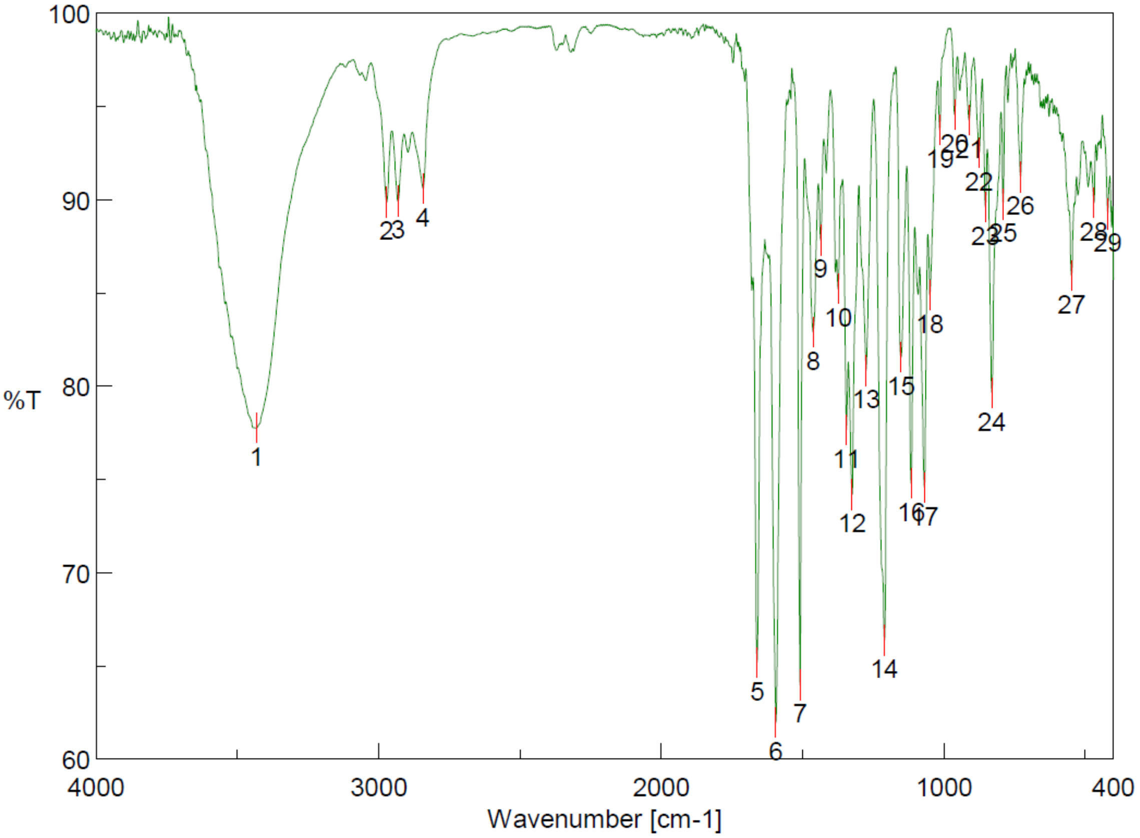

Figure S176. Experimental IR spectrum of *cis*,*cis*-(a*S*,1*R*,3*S*,1'*R*,3'*S*)-**26** recorded as KBr disc.

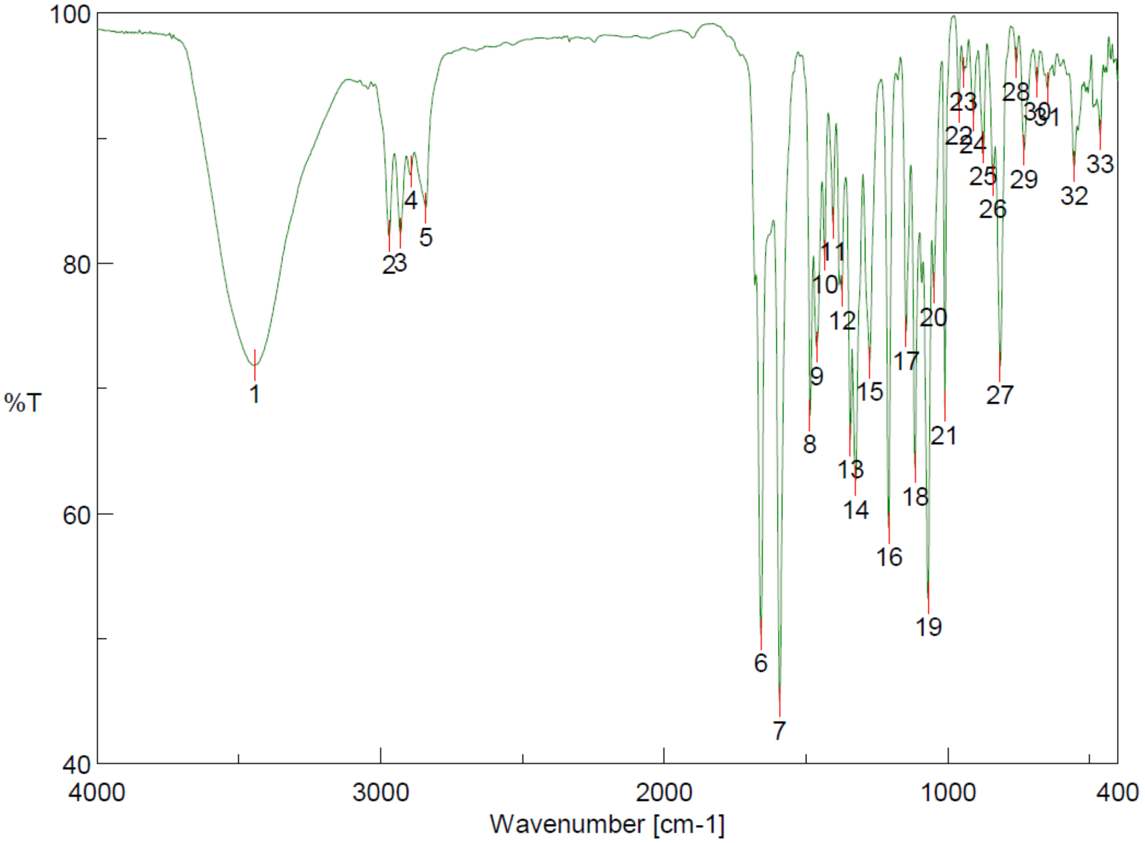

Figure S177. Experimental IR spectrum of *cis*,*cis*-(a*S*,1*R*,3*S*,1'*R*,3'*S*)-**27** recorded as KBr disc.

3.4. MS spectra

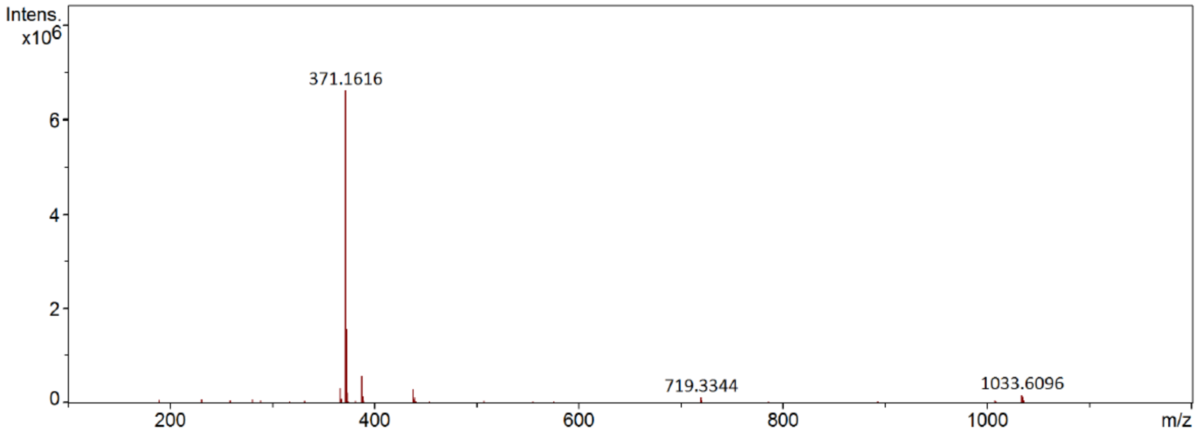

Figure S178. Experimental ESI-HRMS spectrum of (*S*)-**11**.

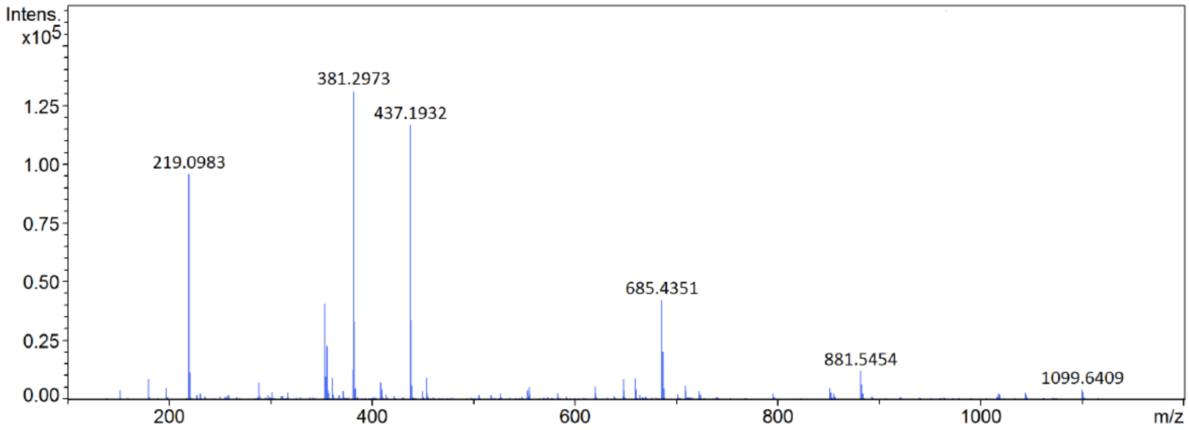

Figure S179. Experimental ESI-HRMS spectrum of (*S*)-**6**.

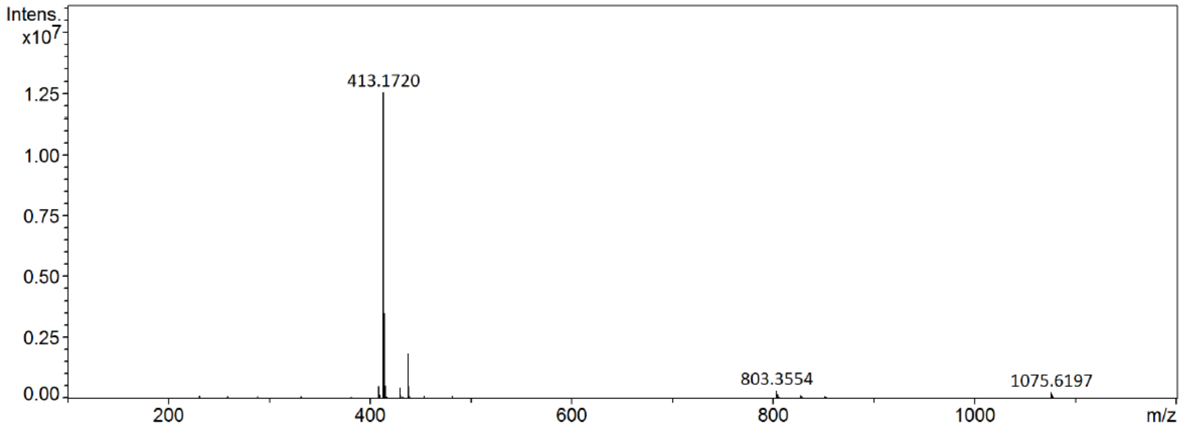

Figure S180. Experimental ESI-HRMS spectrum of (*S*)-**12**.

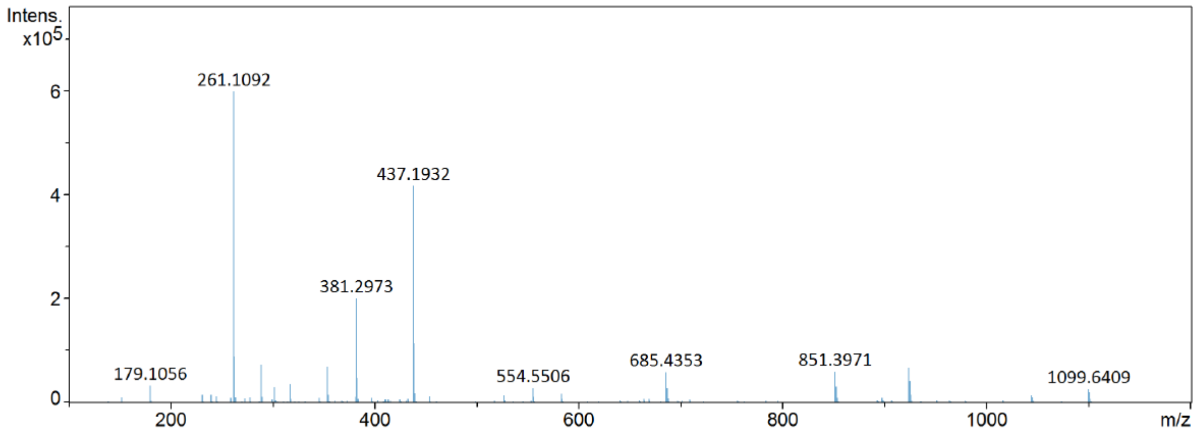

Figure S181. Experimental ESI-HRMS spectrum of (*S*)-**7**.

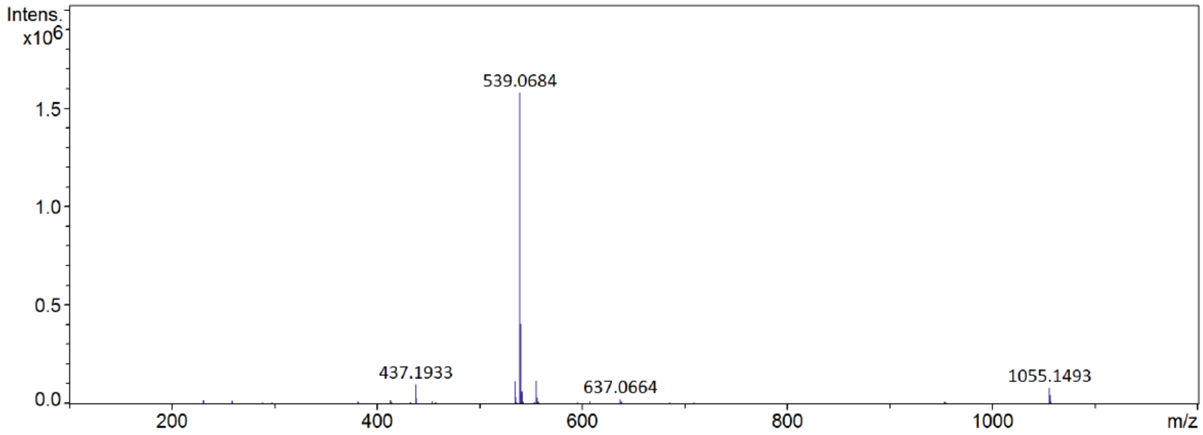

Figure S182. Experimental ESI-HRMS spectrum of (*S*)-**2**.

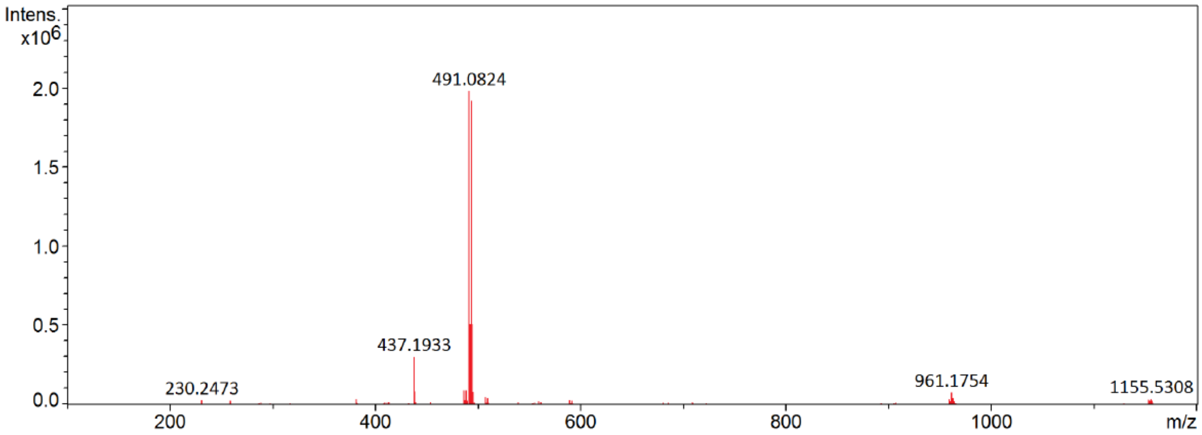

Figure S183. Experimental ESI-HRMS spectrum of (*S*)-**13**.

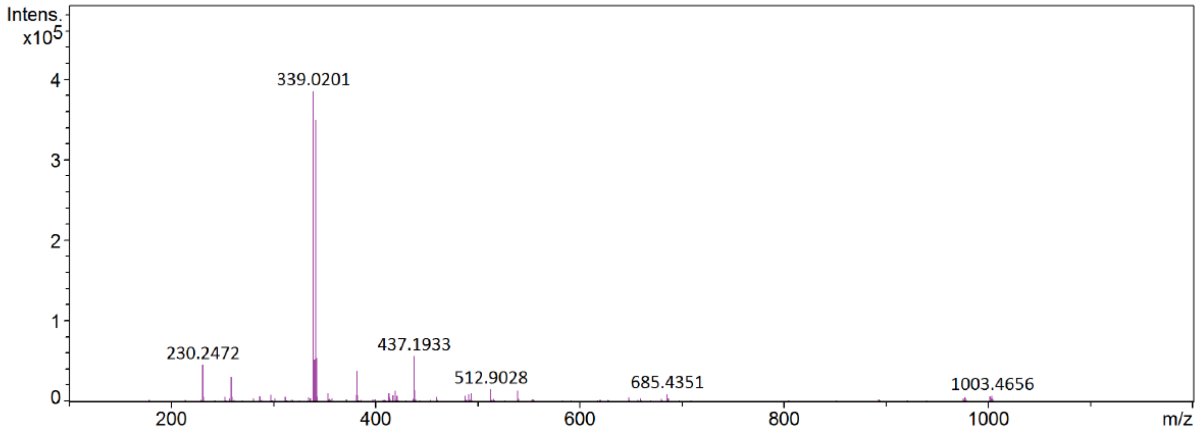

Figure S184. Experimental ESI-HRMS spectrum of (*S*)-**9**.

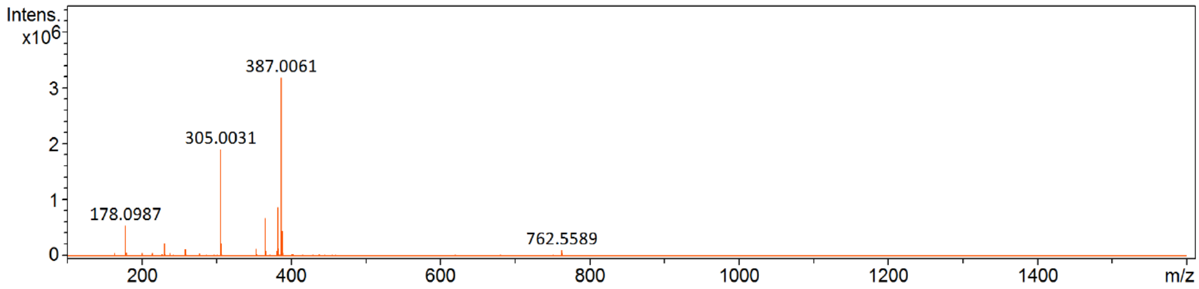

Figure S185. Experimental ESI-HRMS spectrum of (*S*)-**8a** and (*S*)-**8b**.

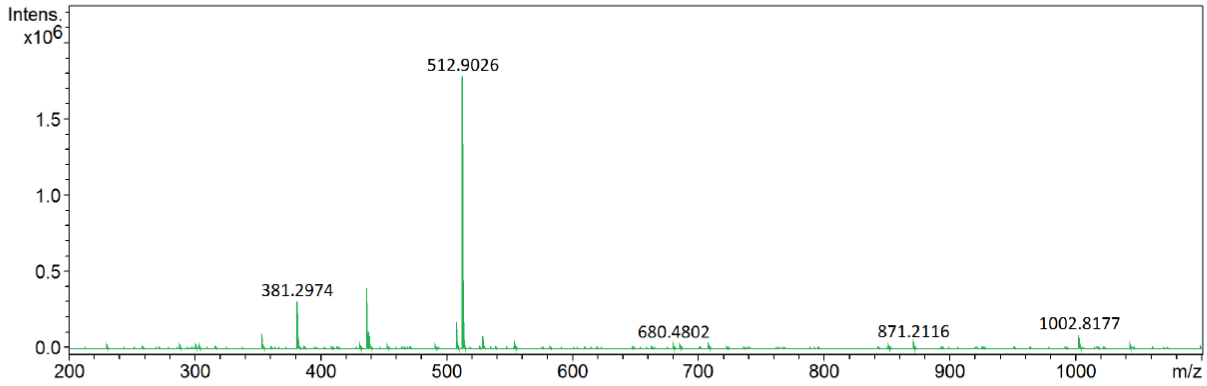

Figure S186. Experimental ESI-HRMS spectrum of (*S*)-**8c**.

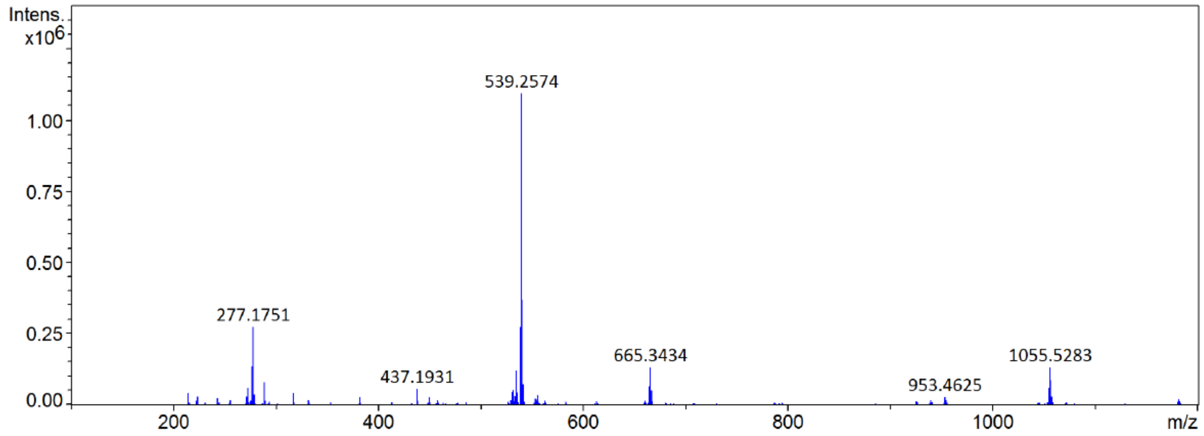

Figure S187. Experimental ESI-HRMS spectrum of (*S*)-**14**.

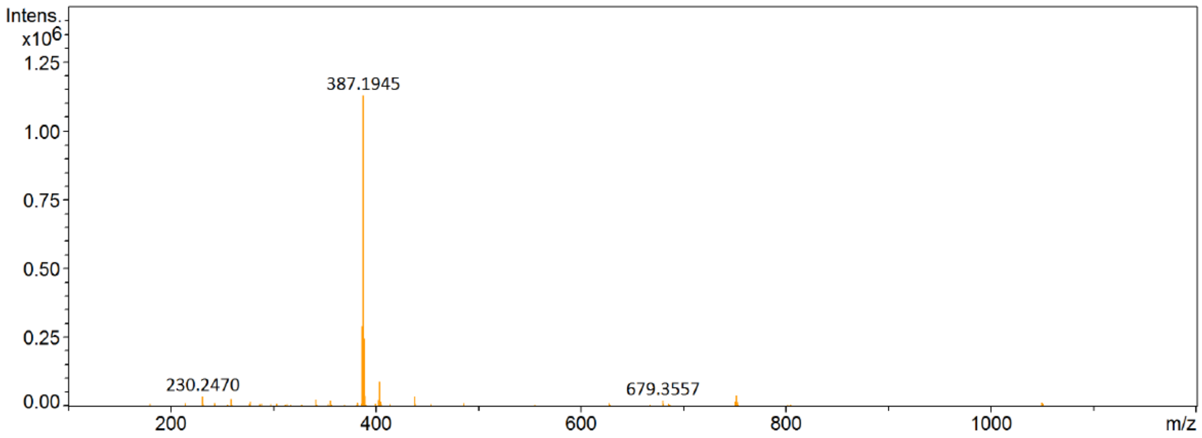

Figure S188. Experimental ESI-HRMS spectrum of (*S*)-**3**.

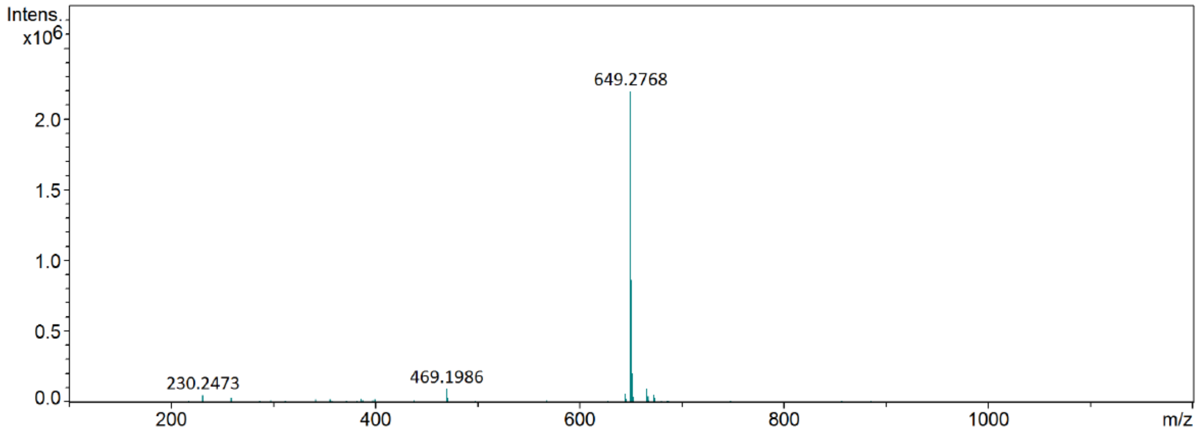

Figure S189. Experimental ESI-HRMS spectrum of (a*S*,2*S*,2’*S*)-**15**.

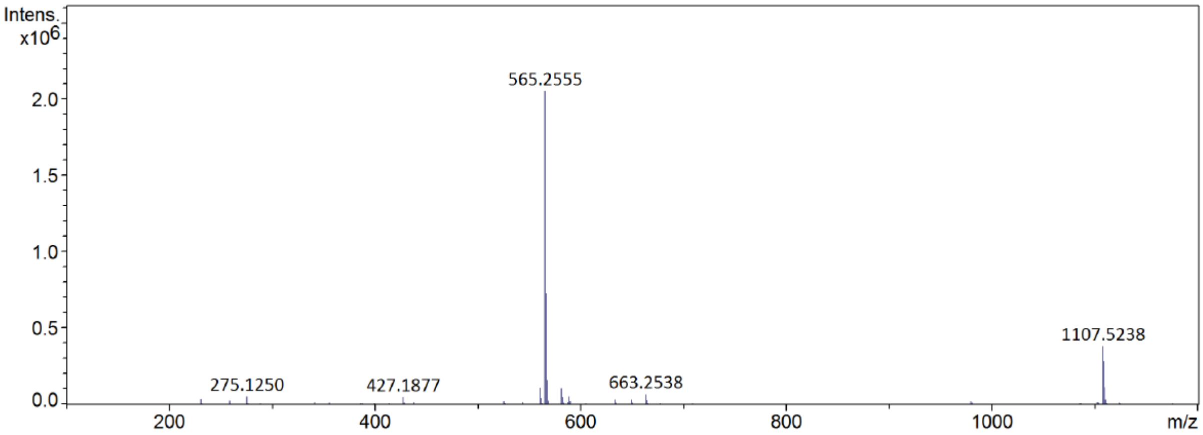

Figure S190. Experimental ESI-HRMS spectrum of (a*S*,2*S*,2’*S*)-**16**.

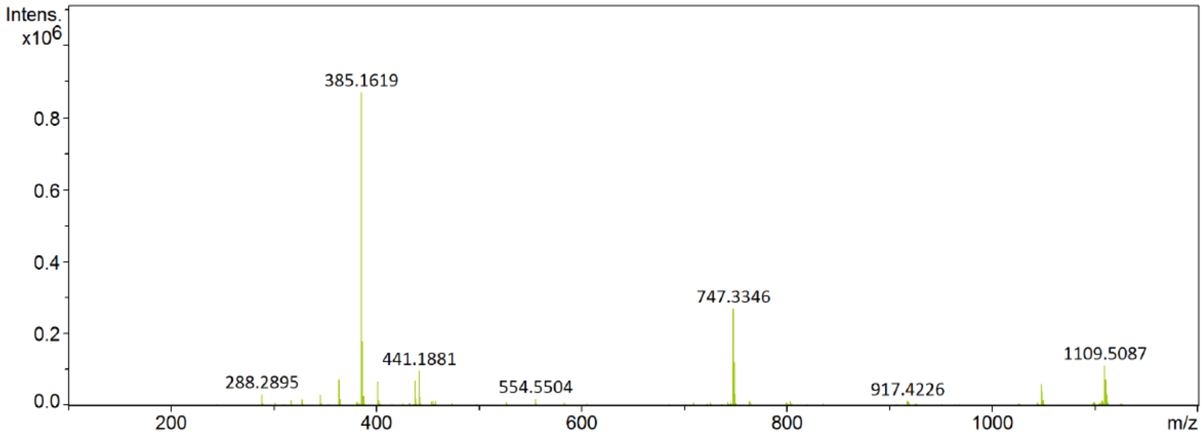

Figure S191. Experimental ESI-HRMS spectrum of (a*S*,2*S*,2'*S*)-**17**.

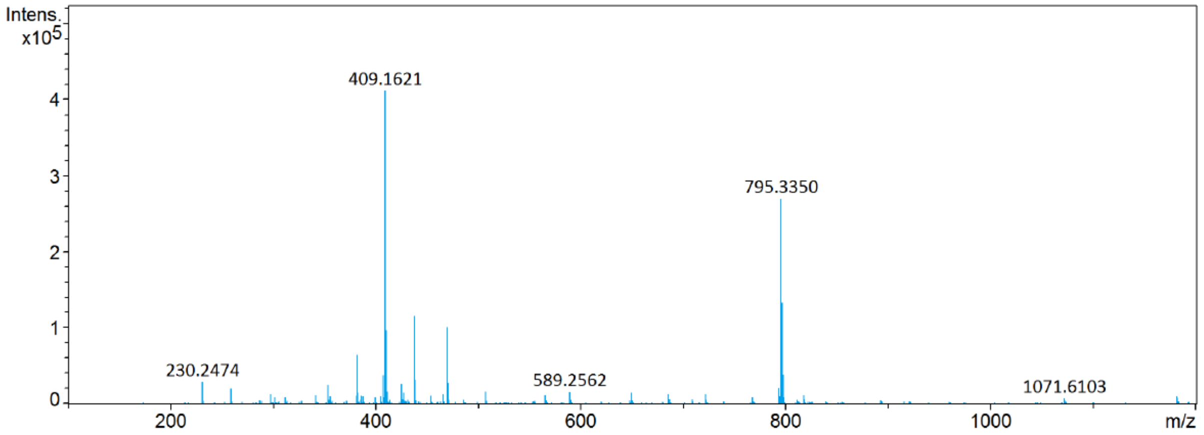

Figure S192. Experimental ESI-HRMS spectrum of (a*S*,3*S*,3'*S*)-**19**.

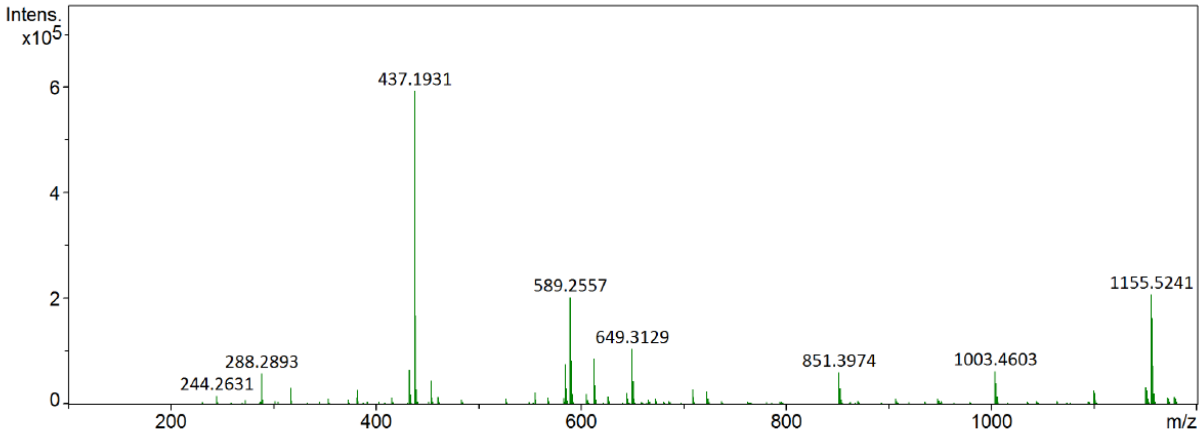

Figure S193. Experimental ESI-HRMS spectrum of (a*S*,3*S*,3'*S*)-**18**.

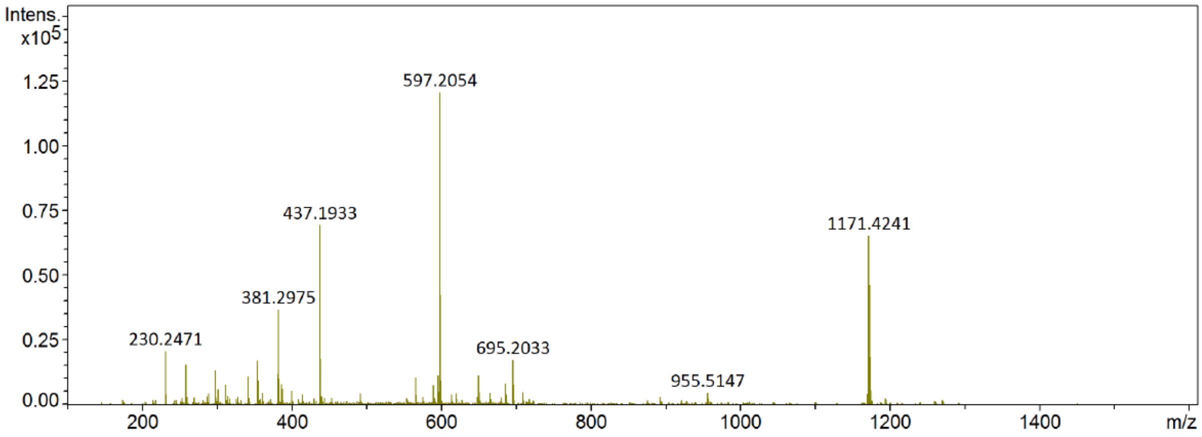

Figure S194. Experimental ESI-HRMS spectrum of *cis*,*cis*-(a*S*,1*R*,3*S*,1'*R*,3'*S*)-**20**.

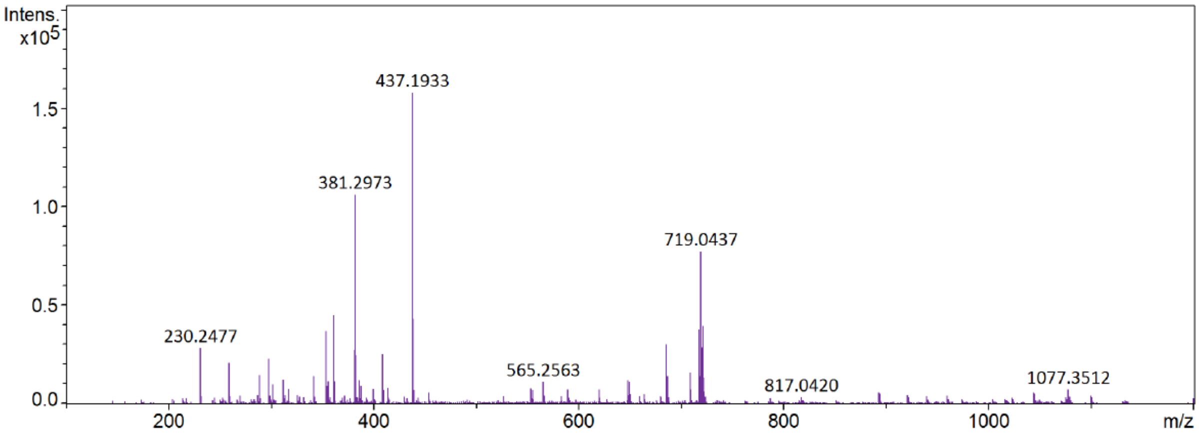

Figure S195. Experimental ESI-HRMS spectrum of *cis*,*cis*-(a*S*,1*R*,3*S*,1'*R*,3'*S*)-**21**.

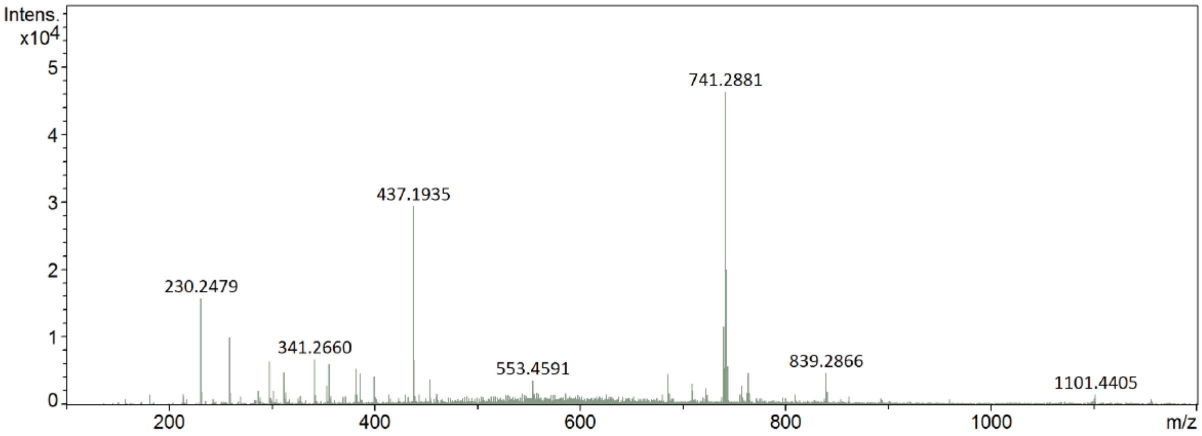

Figure S 196. Experimental ESI-HRMS spectrum of *cis*,*trans*-(a*S*,1*R*,3*S*,1'*S*,3'*S*)-**22**.

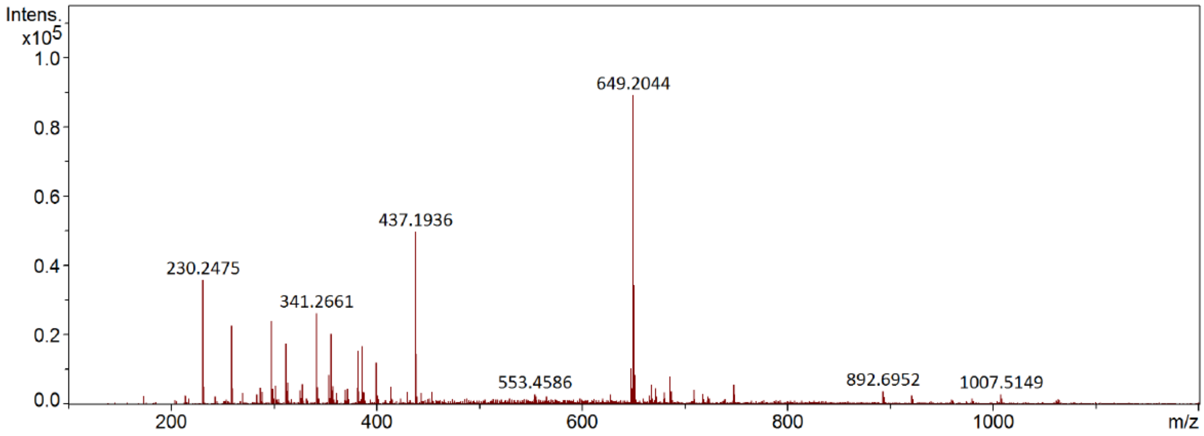

Figure S197. Experimental ESI-HRMS spectrum of *trans*,*trans*-(a*S*,1*S*,3*S*,1’*S*,3’*S*)-**23**.

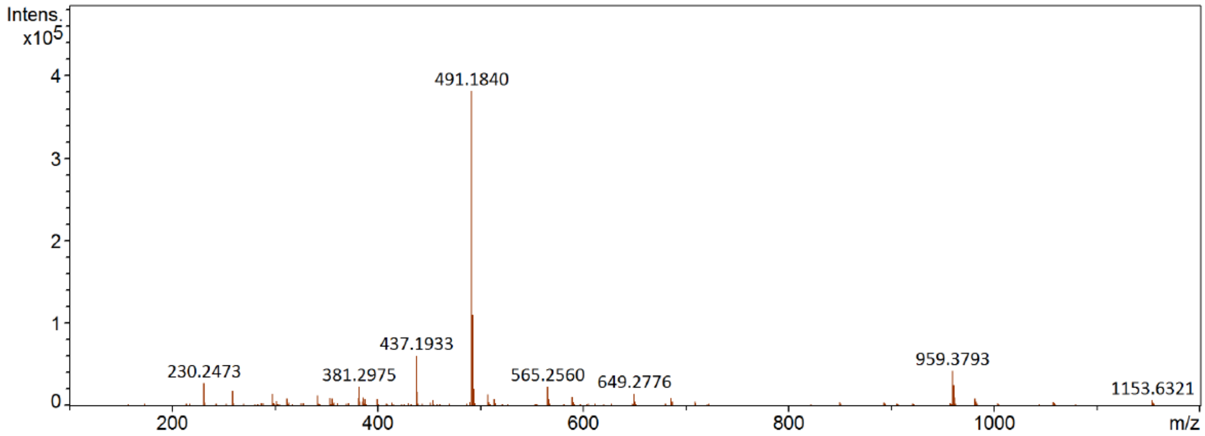

Figure S198. Experimental ESI-HRMS spectrum of *cis*-(a*S*,2*S*,1’*R*,3’*S*)-**24**.

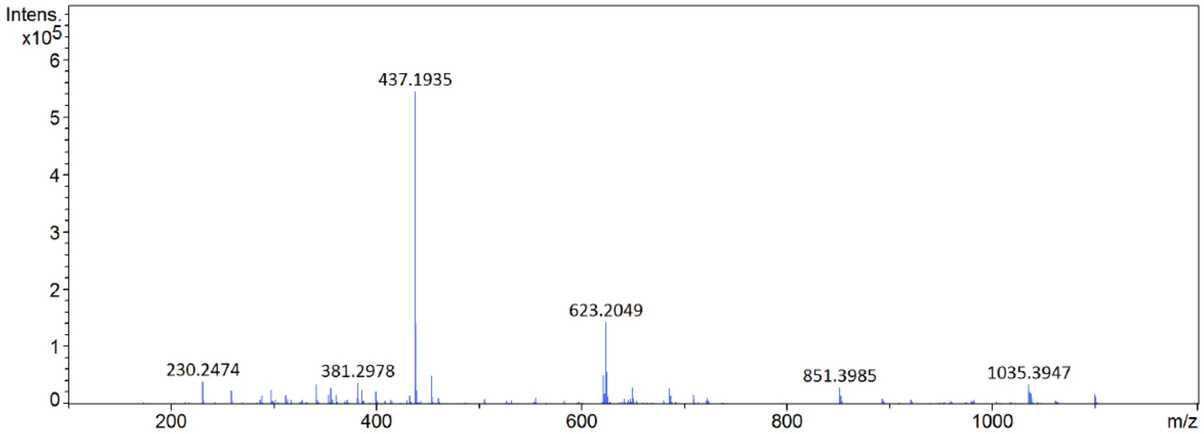

Figure S199. Experimental ESI-HRMS spectrum of *trans*,*cis*-(a*S*,1*S*,3*S*,1’*R*,3’*S*)-**25**.

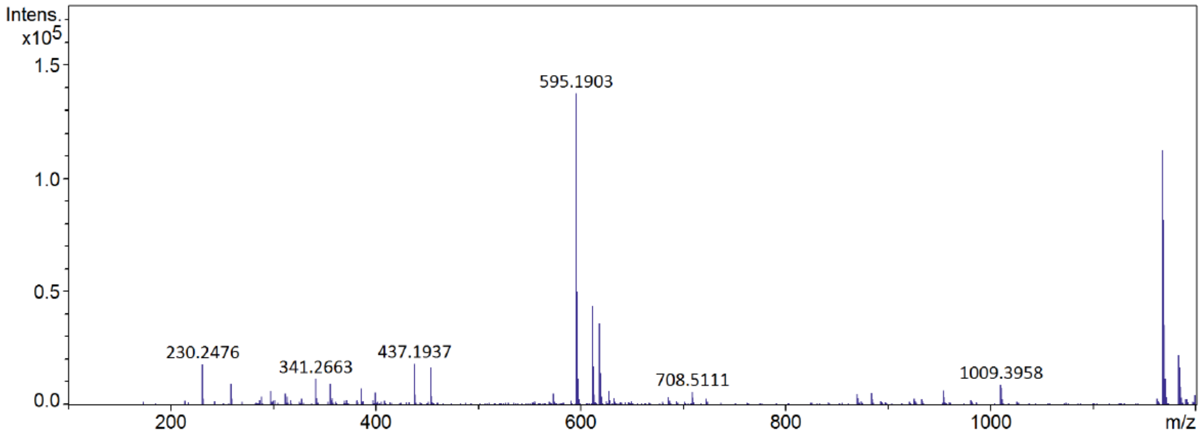

Figure S200. Experimental ESI-HRMS spectrum of *cis*,*cis*-(a*S*,1*R*,3*S*,1'*R*,3'*S*)-**26**.

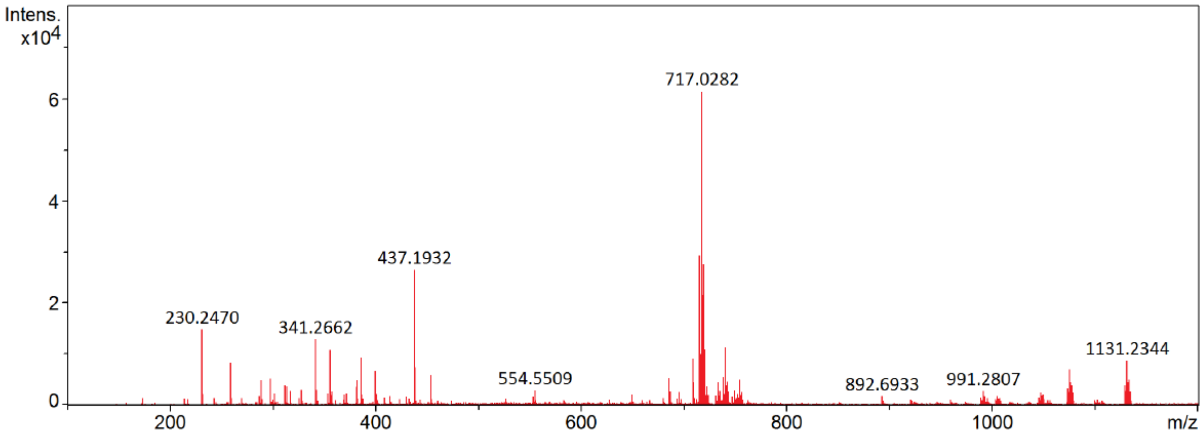

Figure S201. Experimental ESI-HRMS spectrum of *cis*,*cis*-(a*S*,1*R*,3*S*,1'*R*,3'*S*)-**27**.

4. Calculations

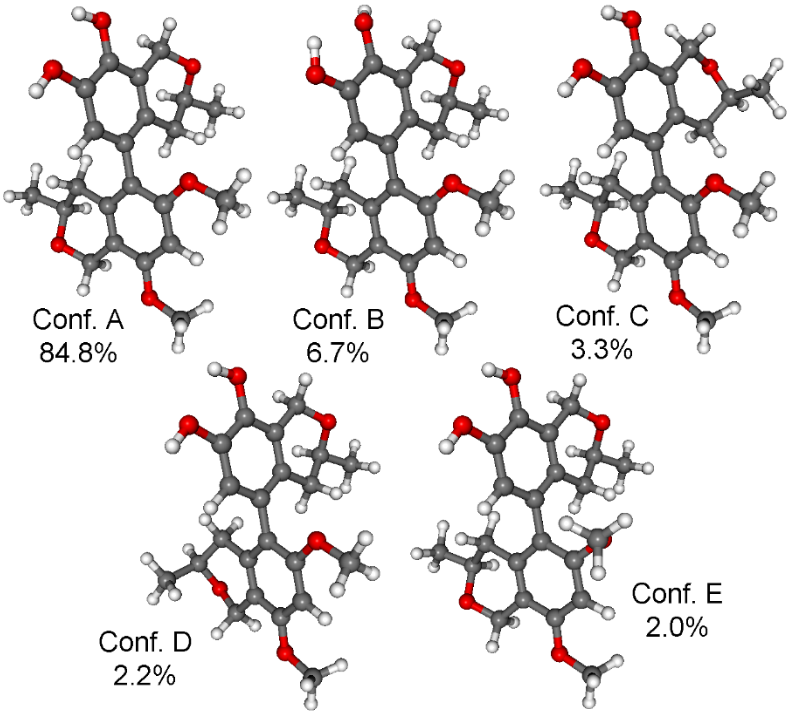

Figure S202. Low-energy conformers (≥1%) of (a*S*,3*S*,3'*S*)-**19** computed at the ωB97X/TZVP PCM/MeCN level.

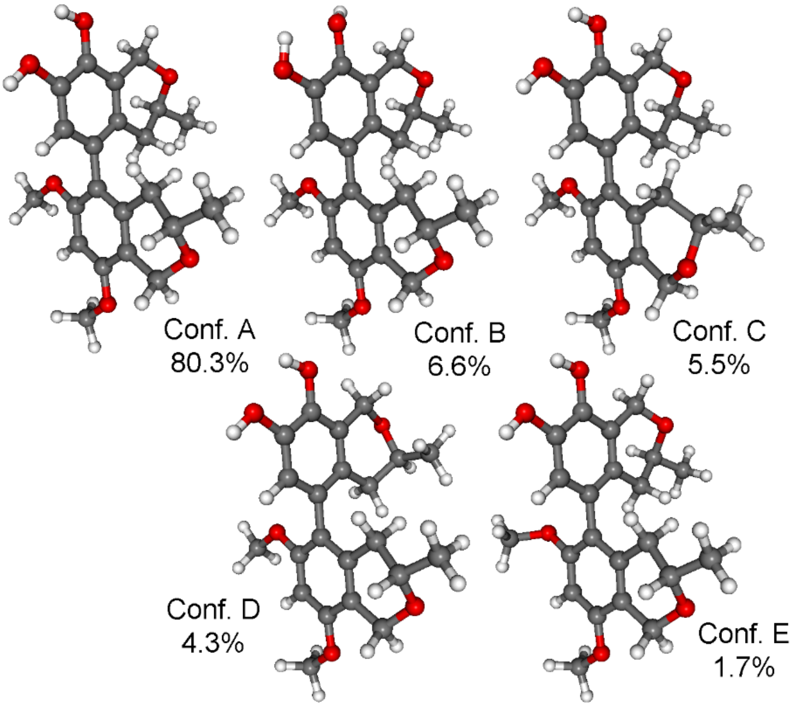

Figure S203. Low-energy conformers (≥1%) of (a*R*,3*S*,3'*S*)-**19** computed at the ωB97X/TZVP PCM/MeCN level.

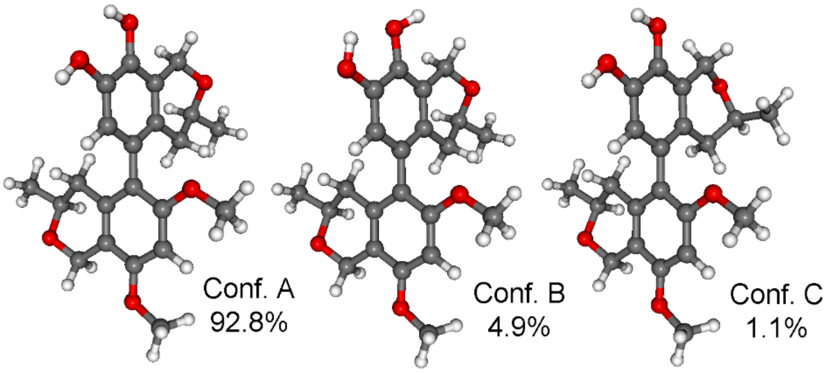

Figure S204. Low-energy conformers (≥1%) of (a*S*,3*S*,3'*S*)-**19** computed at the B3LYP/TZVP PCM/$CHCl_3$ level.

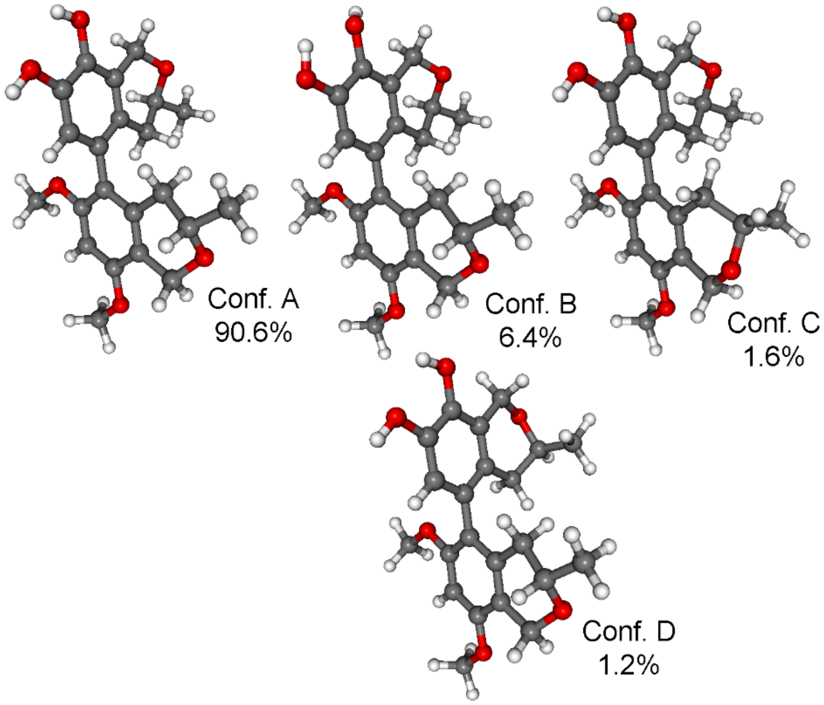

Figure S205. Low-energy conformers (≥1%) of (a*R*,3*S*,3'*S*)-**19** computed at the B3LYP/TZVP PCM/$CHCl_3$ level.

Table S1. Cartesian coordinates and energies of the low-energy conformers calculated at the ωB97X/TZVP PCM/MeCN level.

(a*S*,3*S*,3'*S*)-**19**, Conf A

C	−2.567114	1.325095	−1.909815
C	−1.237986	0.976600	−1.761291
C	−0.833982	0.167883	−0.701860
C	−1.780196	−0.291578	0.216837
C	−3.123790	0.049182	0.054593
C	−3.510712	0.864061	−0.998336
C	0.610759	−0.178856	−0.558635
C	1.069778	−1.439562	−0.943415
C	2.413773	−1.786637	−0.817062
C	3.303657	−0.854625	−0.299169
C	2.874143	0.415387	0.085313
C	1.527241	0.738851	−0.033199
O	−4.827339	1.201025	−1.132295
O	−3.057457	2.115176	−2.916199
O	0.135634	−2.294380	−1.440368
C	0.544710	−3.598872	−1.819059
O	4.630987	−1.099671	−0.130933
C	5.140211	−2.362824	−0.530619
C	−1.379949	−1.162150	1.383584
C	−2.474607	−1.243292	2.437467
O	−3.722273	−1.527732	1.807300
C	−4.171940	−0.443701	1.022294
C	3.873392	1.400443	0.641601
O	3.374037	2.722103	0.677577
C	2.098381	2.787866	1.308424
C	1.063886	2.101115	0.429222
C	−2.218660	−2.324553	3.462420
C	1.777986	4.244931	1.552881
H	−0.505687	1.336564	−2.477834
H	2.758255	−2.764603	−1.116275
H	−4.922978	1.742879	−1.924598
H	−2.353616	2.375302	−3.516735
H	−0.351122	−4.101957	−2.174973
H	0.954727	−4.149012	−0.967462
H	1.284269	−3.566287	−2.623849
H	4.680646	−3.173589	0.041055
H	4.981717	−2.532529	−1.599043
H	6.207322	−2.335960	−0.324112
H	−1.151867	−2.172625	1.024522
H	−0.464214	−0.775982	1.840232
H	−2.561559	−0.269039	2.942235
H	−4.491742	0.382908	1.674268
H	−5.052627	−0.789537	0.479511
H	4.181867	1.092556	1.651968
H	4.770158	1.420038	0.021190
H	2.162449	2.257697	2.270865
H	0.857697	2.736581	−0.439304
H	0.121203	2.005312	0.974941
H	−1.261149	−2.155835	3.958798
H	−3.004749	−2.334882	4.218586
H	−2.187329	−3.302587	2.976568
H	1.765911	4.790564	0.606425
H	2.522798	4.698762	2.208040
H	0.797024	4.344506	2.021280

ωB97X Energy = −1305.45035591 a.u.

(a*S*,3*S*,3'*S*)-**19**, Conf B

C	−2.561024	1.311735	−1.931403
C	−1.231628	0.959631	−1.777683
C	−0.831021	0.167585	−0.707354
C	−1.774423	−0.280506	0.222092
C	−3.118313	0.053669	0.052468
C	−3.498724	0.862505	−1.009177
C	0.613583	−0.179425	−0.559369
C	1.071068	−1.442692	−0.936367
C	2.414730	−1.789878	−0.807851
C	3.305084	−0.855240	−0.295420
C	2.876291	0.417268	0.081701
C	1.529745	0.741155	−0.039346
O	−4.808573	1.216449	−1.264919
O	−2.943756	2.093167	−2.979424
O	0.135442	−2.299282	−1.427275
C	0.543387	−3.605290	−1.802196
O	4.632209	−1.100061	−0.125783
C	5.141268	−2.364531	−0.521579
C	−1.368811	−1.138339	1.396495
C	−2.468218	−1.229860	2.443760
O	−3.708898	−1.529317	1.803879
C	−4.164807	−0.451813	1.018100
C	3.875851	1.405424	0.631833
O	3.377654	2.727788	0.657251
C	2.101861	2.799632	1.287009
C	1.066984	2.107007	0.412803
C	−2.209056	−2.307657	3.471162
C	1.782655	4.258796	1.520166
H	−0.506480	1.315000	−2.501641
H	2.758574	−2.769819	−1.101309
H	−5.261075	1.476683	−0.457675
H	−3.892883	2.250941	−2.896242
H	−0.353102	−4.108924	−2.155619
H	0.953909	−4.152885	−0.949215
H	1.282116	−3.575462	−2.607804
H	4.682566	−3.173380	0.053496
H	4.981551	−2.537925	−1.589219
H	6.208601	−2.336657	−0.316376
H	−1.122698	−2.147295	1.045302
H	−0.462321	−0.735342	1.857121
H	−2.570617	−0.256274	2.946461
H	−4.498263	0.368387	1.673741
H	−5.037551	−0.812348	0.469448
H	4.182715	1.104976	1.644933
H	4.773331	1.419511	0.012338
H	2.165121	2.276755	2.253505
H	0.860990	2.735418	−0.460858
H	0.124375	2.016136	0.959612
H	−2.165118	−3.285666	2.986374
H	−1.256261	−2.129125	3.973057
H	−2.999609	−2.324609	4.222471

H	1.771069	4.797131	0.569561
H	2.527792	4.717011	2.171890
H	0.801744	4.362772	1.987742

ωB97X Energy = −1305.44795245 a.u.

(a*S*,3*S*,3’*S*)-**19**, Conf C

C	2.552486	1.065539	1.982654
C	1.228272	0.710026	1.808532
C	0.808379	0.107980	0.625291
C	1.732385	−0.134292	−0.393487
C	3.066259	0.237038	−0.219084
C	3.473560	0.823508	0.969970
C	−0.633493	−0.243255	0.461209
C	−1.073215	−1.544010	0.710342
C	−2.415745	−1.890143	0.568244
C	−3.323428	−0.916488	0.171397
C	−2.912822	0.392965	−0.077095
C	−1.566928	0.716175	0.055177
O	4.786474	1.163201	1.129180
O	3.059072	1.656596	3.110254
O	−0.119664	−2.439341	1.084714
C	−0.508701	−3.782968	1.321884
O	−4.651119	−1.156844	−0.001121
C	−5.141674	−2.461289	0.268111
C	1.315343	−0.773927	−1.696479
C	2.506532	−1.360244	−2.445710
O	3.536613	−0.371603	−2.537113
C	4.098383	−0.015808	−1.289805
C	−3.929199	1.422762	−0.507204
O	−3.444678	2.746054	−0.397488
C	−2.178648	2.898702	−1.032700
C	−1.125067	2.128601	−0.250740
C	3.004368	−2.666774	−1.843014
C	−1.876261	4.377622	−1.113878
H	0.511983	0.900563	2.602247
H	−2.745588	−2.899605	0.760453
H	4.891239	1.572280	1.996451
H	2.363283	1.808791	3.755552
H	−0.933911	−4.237802	0.422839
H	−1.229530	−3.849522	2.141402
H	0.399644	−4.313076	1.598086
H	−4.687791	−3.199983	−0.398021
H	−4.959705	−2.744217	1.308430
H	−6.212975	−2.422726	0.086678
H	0.578000	−1.558078	−1.509964
H	0.827213	−0.026856	−2.331369
H	2.221521	−1.541596	−3.483501
H	4.684641	0.887843	−1.464524
H	4.799440	−0.789459	−0.947097
H	−4.244898	1.228449	−1.543177
H	−4.819261	1.361155	0.119906
H	−2.250647	2.479637	−2.047961
H	−0.916925	2.660224	0.684674
H	−0.187797	2.110950	−0.813583
H	2.243211	−3.438890	−1.970195
H	3.915134	−2.996908	−2.346204
H	3.208614	−2.574160	−0.773783
H	−1.852132	4.812605	−0.111939
H	−2.636473	4.895193	−1.700547
H	−0.904581	4.540365	−1.583947

ωB97X Energy = −1305.44730456 a.u.

(a*S*,3*S*,3’*S*)-**19**, Conf D

C	−2.620376	1.443926	−1.777100
C	−1.286705	1.103714	−1.655232
C	−0.869146	0.233013	−0.651671
C	−1.805926	−0.298602	0.236148
C	−3.154359	0.038011	0.102983
C	−3.554722	0.916276	−0.891853
C	0.580752	−0.100795	−0.525811
C	1.100550	−1.216594	−1.182537
C	2.450085	−1.549978	−1.078286
C	3.280086	−0.757189	−0.296695
C	2.782310	0.349879	0.390878
C	1.437299	0.677410	0.261311
O	−4.874858	1.249582	−0.996822
O	−3.123937	2.292347	−2.727746
O	0.219768	−1.946631	−1.918814
C	0.697852	−3.094746	−2.601357
O	4.610126	−0.994306	−0.139355
C	5.177346	−2.117628	−0.796023
C	−1.393721	−1.249531	1.334806
C	−2.476196	−1.396781	2.393912
O	−3.729434	−1.647914	1.761131
C	−4.191949	−0.522153	1.045173
C	3.720414	1.176467	1.235261
O	3.049457	2.080128	2.092757
C	2.015742	2.823748	1.445717
C	0.904450	1.872762	1.016964
C	−2.205971	−2.535313	3.350927
C	2.540678	3.679321	0.300639
H	−0.560435	1.520558	−2.346607
H	2.844347	−2.410652	−1.596371
H	−4.980066	1.838652	−1.753409
H	−2.428540	2.586632	−3.322362
H	−0.162977	−3.519383	−3.112193
H	1.102981	−3.832188	−1.902854
H	1.461184	−2.830046	−3.338183
H	5.070243	−2.035993	−1.881017
H	4.720540	−3.049145	−0.451103
H	6.233359	−2.116221	−0.537310
H	−1.177523	−2.235664	0.906945
H	−0.469869	−0.902512	1.805566
H	−2.558776	−0.453639	2.955484
H	−4.513373	0.261251	1.747613
H	−5.073841	−0.841761	0.488431
H	4.314296	0.528405	1.881218
H	4.427714	1.715273	0.589542
H	1.634310	3.485108	2.225769
H	0.173988	2.409340	0.407944

H	0.369973	1.521201	1.906081
H	−2.177745	−3.483536	2.808958
H	−1.243703	−2.392402	3.846095
H	−2.983802	−2.592103	4.113570
H	2.840173	3.076404	−0.559877
H	3.398568	4.269322	0.628929
H	1.759041	4.366144	−0.028990

ωB97X Energy = −1305.44692846 a.u.

(a*S*,3*S*,3'*S*)-**19**, Conf E

C	−2.529567	1.407540	−1.784622
C	−1.207561	1.025776	−1.658913
C	−0.816963	0.156218	−0.642766
C	−1.767758	−0.326031	0.259051
C	−3.104522	0.051145	0.120246
C	−3.478645	0.922926	−0.890697
C	0.621071	−0.226790	−0.523333
C	1.051303	−1.485662	−0.917413
C	2.383541	−1.874058	−0.812005
C	3.306151	−0.978877	−0.297533
C	2.905076	0.301370	0.103173
C	1.570094	0.665397	0.006184
O	−4.787707	1.293559	−1.001262
O	−3.008052	2.254798	−2.748816
O	0.145610	−2.409850	−1.391898
C	−0.027045	−2.370446	−2.805721
O	4.627685	−1.254689	−0.139542
C	5.095094	−2.531862	−0.547133
C	−1.381401	−1.256095	1.383704
C	−2.470421	−1.352585	2.441947
O	−3.728065	−1.584361	1.810176
C	−4.158620	−0.460844	1.071610
C	3.937294	1.250417	0.662820
O	3.473819	2.582251	0.739893
C	2.206595	2.666807	1.385844
C	1.147153	2.029606	0.499486
C	−2.232056	−2.477360	3.423342
C	1.928185	4.125515	1.668285
H	−0.470322	1.406680	−2.359663
H	2.658027	−2.872019	−1.127284
H	−4.875933	1.874039	−1.766677
H	−2.305246	2.519349	−3.348677
H	0.920697	−2.563267	−3.317663
H	−0.421601	−1.400143	−3.122449
H	−0.741375	−3.151413	−3.059473
H	4.613245	−3.329166	0.025036
H	4.922029	−2.692381	−1.614668
H	6.163968	−2.539694	−0.348570
H	−1.182684	−2.254923	0.978721
H	−0.452743	−0.913459	1.848876
H	−2.531503	−0.396603	2.984085
H	−4.459713	0.344919	1.757596
H	−5.047957	−0.766962	0.519096
H	4.256460	0.906200	1.657807
H	4.821988	1.261226	0.025188
H	2.264842	2.111459	2.334228
H	0.952499	2.690429	−0.352528
H	0.206288	1.946554	1.050403
H	−2.226029	−3.436950	2.901113
H	−1.267826	−2.348324	3.918540
H	−3.013194	−2.498561	4.184416
H	1.925084	4.694486	0.735630
H	2.689328	4.542301	2.329233
H	0.953359	4.239917	2.145943

ωB97X Energy = −1305.44683658 a.u.

(a*R*,3*S*,3'*S*)-**19**, Conf A

C	2.672048	0.089892	−2.260694
C	1.323698	−0.101779	−2.026801
C	0.846669	−0.244614	−0.726016
C	1.738774	−0.198472	0.346818
C	3.098317	0.009597	0.107419
C	3.561247	0.142302	−1.192354
C	−0.618234	−0.418171	−0.496004
C	−1.184319	−1.693397	−0.497380
C	−2.549330	−1.876319	−0.281654
C	−3.349303	−0.763918	−0.054115
C	−2.806201	0.520471	−0.026638
C	−1.446066	0.685389	−0.261863
O	4.896213	0.329911	−1.409926
O	3.234816	0.240659	−3.500684
O	−0.330419	−2.730571	−0.712322
C	−0.856910	−4.047892	−0.721592
O	−4.690703	−0.837460	0.158913
C	−5.304706	−2.117092	0.163702
C	1.262417	−0.366431	1.770578
C	2.412662	−0.652504	2.725492
O	3.464464	0.283003	2.496285
C	4.086692	0.075272	1.246056
C	−3.705944	1.702564	0.241481
O	−2.985554	2.869194	0.584966
C	−1.953145	3.147238	−0.356530
C	−0.871329	2.082905	−0.249445
C	2.006019	−0.543214	4.177317
C	−1.429410	4.537059	−0.074942
H	0.632275	−0.138759	−2.863513
H	−2.977615	−2.866758	−0.291483
H	5.038344	0.423318	−2.359475
H	2.561031	0.211000	−4.185221
H	−1.307076	−4.302638	0.241841
H	−0.012831	−4.707133	−0.909455
H	−1.597665	−4.174418	−1.516060
H	−6.361199	−1.943006	0.352245
H	−4.894674	−2.750047	0.955337
H	−5.186525	−2.615403	−0.802284
H	0.746311	0.542329	2.103520
H	0.533427	−1.179212	1.825963
H	2.803222	−1.661948	2.526220
H	4.680739	−0.850522	1.272768

H	4.780954	0.903089	1.095713
H	−4.336835	1.902091	−0.637722
H	−4.373096	1.487150	1.076749
H	−2.390756	3.115113	−1.365905
H	−0.310750	2.240281	0.679790
H	−0.157619	2.200925	−1.068195
H	1.667873	0.472040	4.397494
H	1.189011	−1.233906	4.394287
H	2.844904	−0.781782	4.832577
H	−2.223849	5.277361	−0.178706
H	−0.628241	4.788200	−0.772570
H	−1.031747	4.591427	0.941237

ωB97X Energy = −1305.45001748 a.u.

(a*R*,3*S*,3'*S*)-**19**, Conf B

C	2.663272	0.113455	−2.271914
C	1.313803	−0.075602	−2.032894
C	0.843567	−0.237932	−0.734362
C	1.735874	−0.209137	0.340811
C	3.093349	0.008539	0.101264
C	3.549524	0.148415	−1.201463
C	−0.620913	−0.414196	−0.501857
C	−1.184725	−1.690026	−0.509112
C	−2.549354	−1.875780	−0.293914
C	−3.350558	−0.765448	−0.060677
C	−2.808897	0.519448	−0.026394
C	−1.449152	0.687431	−0.261405
O	4.869496	0.390015	−1.523818
O	3.117140	0.264012	−3.547373
O	−0.328736	−2.724335	−0.729470
C	−0.852883	−4.042561	−0.746749
O	−4.691821	−0.841558	0.152000
C	−5.304682	−2.121754	0.148713
C	1.257862	−0.397478	1.761405
C	2.409392	−0.673145	2.716855
O	3.448028	0.280439	2.493405
C	4.075609	0.087286	1.246511
C	−3.709702	1.699206	0.248411
O	−2.990216	2.864366	0.598869
C	−1.958394	3.149188	−0.341277
C	−0.875503	2.085306	−0.241267
C	2.000425	−0.576664	4.168677
C	−1.435959	4.537753	−0.051280
H	0.628541	−0.100329	−2.873132
H	−2.976360	−2.866706	−0.309315
H	5.460233	−0.150272	−0.991528
H	4.078441	0.347714	−3.509129
H	−0.007529	−4.699169	−0.938045
H	−1.592853	−4.165586	−1.542444
H	−1.303164	−4.303679	0.214920
H	−5.185264	−2.614332	−0.820046
H	−4.894867	−2.759000	0.937028
H	−6.361487	−1.949642	0.337327
H	0.724598	0.498684	2.101071
H	0.543418	−1.223739	1.806852
H	2.816132	−1.674988	2.512859
H	4.688269	−0.827813	1.277992
H	4.751762	0.932927	1.102882
H	−4.340541	1.903368	−0.629739
H	−4.376771	1.478363	1.082313
H	−2.396211	3.122877	−1.350673
H	−0.314256	2.237536	0.688483
H	−0.162890	2.208941	−1.060118
H	1.646268	0.432069	4.393259
H	1.193986	−1.280988	4.381025
H	2.842236	−0.805677	4.823508
H	−1.038418	4.586389	0.965246
H	−2.231099	5.277909	−0.150611
H	−0.635044	4.793798	−0.747403

ωB97X Energy = −1305.44765230 a.u.

(a*R*,3*S*,3'*S*)-**19**, Conf C

C	−2.743263	1.038341	−2.042986
C	−1.387090	1.087942	−1.780231
C	−0.878658	0.558545	−0.597230
C	−1.745255	−0.028377	0.326976
C	−3.111642	−0.091990	0.050664
C	−3.607256	0.451435	−1.124710
C	0.592137	0.604638	−0.345313
C	1.156417	1.690873	0.323673
C	2.526466	1.750038	0.574149
C	3.333085	0.701502	0.151373
C	2.796605	−0.393097	−0.527095
C	1.427035	−0.438589	−0.760514
O	−4.949301	0.396967	−1.370948
O	−3.336218	1.534410	−3.174192
O	0.295113	2.666268	0.721650
C	0.817903	3.785487	1.419243
O	4.676403	0.658930	0.359860
C	5.287342	1.740527	1.046325
C	−1.229208	−0.612852	1.620204
C	−2.349250	−0.859373	2.620615
O	−3.421742	−1.544474	1.976801
C	−4.072177	−0.728054	1.025658
C	3.709854	−1.517127	−0.949681
O	3.094602	−2.438743	−1.829713
C	1.785402	−2.839244	−1.420631
C	0.852642	−1.635305	−1.482136
C	−1.905306	−1.700759	3.795522
C	1.781088	−3.525779	−0.061385
H	−0.715668	1.544008	−2.501609
H	2.952829	2.595233	1.092352
H	−5.116596	0.796851	−2.232684
H	−2.680367	1.923940	−3.758822
H	1.531446	4.340010	0.803510
H	1.298605	3.482896	2.353707
H	−0.033693	4.423257	1.643861
H	6.346355	1.501456	1.104425
H	4.883314	1.845033	2.056929
H	5.157180	2.679465	0.501517

H	−0.715324	−1.562564	1.422870
H	−0.488040	0.058296	2.062290
H	−2.728473	0.108900	2.981171
H	−4.651311	0.054795	1.537700
H	−4.781630	−1.364146	0.494827
H	4.575522	−1.120059	−1.481867
H	4.094667	−2.035479	−0.060360
H	1.479247	−3.563826	−2.177180
H	−0.120352	−1.898163	−1.060354
H	0.675781	−1.372088	−2.529491
H	−1.585020	−2.686808	3.450581
H	−1.065695	−1.224685	4.305504
H	−2.719018	−1.828137	4.510756
H	2.531861	−4.317652	−0.031782
H	0.802094	−3.975052	0.116471
H	1.980183	−2.825018	0.753074

ωB97X Energy = −1305.44747799 a.u.

(a*R*,3*S*,3’*S*)-**19**, Conf D

C	2.688911	−0.748025	−2.047179
C	1.329597	−0.808942	−1.807335
C	0.826006	−0.563074	−0.532230
C	1.702481	−0.257068	0.510584
C	3.075868	−0.198839	0.265116
C	3.562349	−0.432220	−1.011963
C	−0.649264	−0.604218	−0.306797
C	−1.278676	−1.807935	0.010480
C	−2.654706	−1.865313	0.228002
C	−3.399796	−0.696798	0.137469
C	−2.792418	0.524304	−0.155485
C	−1.423083	0.558652	−0.391368
O	4.906695	−0.354084	−1.237743
O	3.276601	−0.972403	−3.264278
O	−0.476486	−2.903464	0.095380
C	−1.068969	−4.150722	0.420530
O	−4.745789	−0.650848	0.328430
C	−5.420270	−1.852983	0.666394
C	1.202767	−0.011729	1.914972
C	2.217855	0.760226	2.750024
O	3.491276	0.116193	2.652446
C	4.054786	0.155390	1.356973
C	−3.630878	1.777828	−0.221133
O	−2.852794	2.956505	−0.157455
C	−1.793924	2.941170	−1.110254
C	−0.773692	1.882675	−0.718947
C	2.281913	2.238878	2.392174
C	−1.197160	4.329089	−1.164207
H	0.650083	−1.047553	−2.620141
H	−3.134108	−2.802927	0.464074
H	5.071684	−0.566249	−2.164195
H	2.616466	−1.209441	−3.921314
H	−1.803934	−4.450077	−0.331723
H	−1.544636	−4.120219	1.404674
H	−0.257085	−4.873819	0.436325
H	−5.041319	−2.268404	1.604143
H	−6.467705	−1.587645	0.786991
H	−5.324692	−2.597272	−0.128835
H	0.254685	0.531148	1.892852
H	1.000106	−0.972279	2.399408
H	1.955653	0.667875	3.805381
H	4.883391	−0.554628	1.361802
H	4.485000	1.145998	1.153767
H	−4.233261	1.780711	−1.142012
H	−4.324122	1.812573	0.619806
H	−2.219376	2.684322	−2.092277
H	−0.208351	2.238194	0.151966
H	−0.049390	1.754047	−1.526918
H	3.090669	2.729375	2.937259
H	2.436172	2.397325	1.321986
H	1.341931	2.721114	2.667702
H	−0.812990	4.612199	−0.181281
H	−1.946822	5.060024	−1.470023
H	−0.372263	4.358323	−1.878589

ωB97X Energy = −1305.44726332 a.u.

(a*R*,3*S*,3’*S*)-**19**, Conf E

C	2.648063	−0.753202	−2.093537
C	1.302530	−0.847997	−1.791748
C	0.836233	−0.486783	−0.529813
C	1.734315	−0.028873	0.435942
C	3.090165	0.079444	0.121966
C	3.543333	−0.291815	−1.134388
C	−0.624845	−0.579356	−0.237566
C	−1.175987	−1.768438	0.214374
C	−2.537346	−1.888913	0.486936
C	−3.358187	−0.789293	0.306852
C	−2.826709	0.432413	−0.126253
C	−1.472648	0.529980	−0.405862
O	4.874923	−0.193179	−1.418973
O	3.201909	−1.084328	−3.301550
O	−0.346759	−2.840760	0.450001
C	−0.519905	−3.935963	−0.443318
O	−4.697476	−0.794780	0.539576
C	−5.290378	−2.001482	0.995355
C	1.270460	0.353504	1.821912
C	2.430357	0.455672	2.802162
O	3.472572	1.239904	2.225015
C	4.086551	0.579310	1.139118
C	−3.747269	1.617678	−0.291018
O	−3.050056	2.842164	−0.391310
C	−2.019278	2.785719	−1.373411
C	−0.920400	1.846658	−0.899043
C	2.033410	1.105694	4.107744
C	−1.519386	4.193841	−1.602647
H	0.605625	−1.201688	−2.545954
H	−2.919285	−2.833020	0.851919
H	5.010956	−0.464989	−2.334505
H	2.523913	−1.364887	−3.922102
H	−1.530936	−4.348038	−0.381192
H	0.196402	−4.699245	−0.145923

H	−0.317160	−3.627610	−1.473797
H	−5.163493	−2.803095	0.262666
H	−4.867041	−2.311524	1.954502
H	−6.349414	−1.789662	1.119812
H	0.749968	1.318476	1.791194
H	0.548894	−0.381042	2.189131
H	2.827371	−0.552314	2.995611
H	4.692469	−0.262481	1.506140
H	4.768882	1.294452	0.677742
H	−4.382011	1.475166	−1.178225
H	−4.409500	1.701702	0.571115
H	−2.452578	2.390753	−2.304821
H	−0.359886	2.335618	−0.093296
H	−0.210796	1.671922	−1.711383
H	1.697560	2.130018	3.930120
H	1.216556	0.551266	4.573592
H	2.875785	1.129506	4.800355
H	−1.128232	4.610736	−0.671615
H	−2.324899	4.836008	−1.961346
H	−0.718518	4.195023	−2.344353

ωB97X Energy = −1305.44635976 a.u.

Table S2. Cartesian coordinates and energies of the low-energy conformers calculated at the B3LYP/TZVP PCM/$CHCl_3$ level.

(a*S*,3*S*,3'*S*)-**19**, Conf A

C	−2.559115	1.214896	−1.995761
C	−1.229404	0.874732	−1.810420
C	−0.832204	0.126836	−0.698891
C	−1.796215	−0.281709	0.235437
C	−3.141823	0.054436	0.039253
C	−3.517004	0.810964	−1.067027
C	0.615518	−0.209877	−0.534834
C	1.085667	−1.480668	−0.894020
C	2.435543	−1.814828	−0.764898
C	3.325600	−0.865009	−0.271297
C	2.889856	0.415925	0.083763
C	1.535063	0.730148	−0.038211
O	−4.836388	1.144761	−1.234144
O	−3.037096	1.948599	−3.060144
O	0.151742	−2.356280	−1.371416
C	0.561156	−3.664396	−1.757132
O	4.661684	−1.101007	−0.100525
C	5.192861	−2.370539	−0.470552
C	−1.416548	−1.100351	1.449720
C	−2.513607	−1.109256	2.511528
O	−3.772189	−1.411578	1.894357
C	−4.204631	−0.376170	1.025258
C	3.887624	1.421217	0.613036
O	3.385299	2.750137	0.629280
C	2.096914	2.830612	1.250242
C	1.070340	2.109809	0.380562
C	−2.277887	−2.138650	3.600395
C	1.774175	4.298887	1.453461
H	−0.490789	1.189422	−2.539631
H	2.783901	−2.795638	−1.043791
H	−4.922754	1.634912	−2.063834
H	−2.325235	2.150072	−3.678639
H	0.984646	−4.217117	−0.913768
H	1.286938	−3.632027	−2.574459
H	−0.340558	−4.167379	−2.098004
H	4.744410	−3.176526	0.116404
H	5.046916	−2.567977	−1.535954
H	6.257714	−2.320359	−0.256722
H	−1.211588	−2.132483	1.144781
H	−0.491528	−0.716337	1.886985
H	−2.585007	−0.108832	2.964282
H	−4.537278	0.491507	1.615346
H	−5.078406	−0.759388	0.497510
H	4.209936	1.135431	1.626040
H	4.779925	1.437862	−0.013159
H	2.153436	2.330781	2.228986
H	0.872993	2.719563	−0.507693
H	0.121972	2.035137	0.917527
H	−2.253789	−3.143910	3.174017
H	−1.324745	−1.953373	4.099772
H	−3.072320	−2.098513	4.347003
H	1.764388	4.822069	0.494734
H	2.518146	4.770730	2.096856
H	0.792989	4.413247	1.918476

B3LYP Energy = −1305.77435059 a.u.

(a*S*,3*S*,3'*S*)-**19**, Conf B

C	−2.553043	1.166425	−2.036555
C	−1.222638	0.835438	−1.833512
C	−0.830644	0.109190	−0.708338
C	−1.793673	−0.287386	0.233056
C	−3.139939	0.044255	0.028668
C	−3.507898	0.772132	−1.100107
C	0.617686	−0.221004	−0.533121
C	1.091889	−1.495602	−0.871390
C	2.442917	−1.822760	−0.736893
C	3.329272	−0.862109	−0.257896
C	2.888648	0.422459	0.077496
C	1.533092	0.730403	−0.051269
O	−4.800640	1.188725	−1.367540
O	−2.918862	1.873640	−3.149630
O	0.160514	−2.382734	−1.332944
C	0.578838	−3.687559	−1.719962
O	4.666208	−1.090582	−0.083183
C	5.203228	−2.361325	−0.440238
C	−1.409506	−1.079209	1.463619
C	−2.491664	−1.046675	2.538250
O	−3.762289	−1.355030	1.942573
C	−4.191238	−0.345803	1.047564
C	3.882538	1.439477	0.591446
O	3.376421	2.766819	0.586394
C	2.086211	2.853640	1.202449
C	1.063814	2.115823	0.342239
C	−2.257322	−2.047504	3.653095
C	1.758864	4.324161	1.380579
H	−0.491521	1.142261	−2.570855
H	2.795089	−2.806349	−1.000759
H	−5.449374	0.591575	−0.977162
H	−3.881592	1.975349	−3.134819
H	1.009109	−4.237444	−0.878089
H	1.301915	−3.649054	−2.539335
H	−0.319857	−4.197470	−2.058573
H	4.759196	−3.163349	0.155534
H	5.057183	−2.570593	−1.503347
H	6.268007	−2.303750	−0.227904
H	−1.219706	−2.121618	1.184679
H	−0.475286	−0.694393	1.879530
H	−2.550395	−0.033342	2.962251
H	−4.508260	0.545611	1.611150
H	−5.082237	−0.753235	0.560474

H	4.204913	1.170696	1.609185
H	4.775302	1.448833	−0.034254
H	2.141772	2.370003	2.189491
H	0.866980	2.708991	−0.557161
H	0.114252	2.048483	0.878218
H	−2.247423	−3.064601	3.255443
H	−1.297502	−1.857936	4.137648
H	−3.043756	−1.977421	4.405876
H	1.749286	4.831064	0.413218
H	2.500192	4.808732	2.017578
H	0.776313	4.443457	1.841535

B3LYP Energy = −1305.77157114 a.u.

(a*S*,3*S*,3'*S*)-**19**, Conf C

C	2.574105	0.798240	2.103984
C	1.243440	0.481184	1.885814
C	0.812694	0.035855	0.633934
C	1.742060	−0.089834	−0.410260
C	3.084504	0.243654	−0.189710
C	3.497584	0.674836	1.067719
C	−0.636167	−0.283024	0.440779
C	−1.099657	−1.590444	0.639122
C	−2.450007	−1.910171	0.480699
C	−3.346994	−0.907739	0.121644
C	−2.917845	0.410107	−0.067406
C	−1.562537	0.710555	0.081648
O	4.819290	0.978974	1.269277
O	3.085304	1.241385	3.304750
O	−0.157344	−2.518344	0.984842
C	−0.564094	−3.861799	1.224162
O	−4.684097	−1.124923	−0.065913
C	−5.208104	−2.433725	0.142121
C	1.322355	−0.554910	−1.787561
C	2.503446	−1.060193	−2.619203
O	3.561581	−0.086622	−2.576527
C	4.118294	0.110935	−1.283768
C	−3.922133	1.474061	−0.449406
O	−3.426608	2.794528	−0.279232
C	−2.142350	2.970323	−0.889617
C	−1.106490	2.138000	−0.138665
C	2.976358	−2.460314	−2.231052
C	−1.828303	4.454404	−0.882227
H	0.530434	0.577647	2.697524
H	−2.793835	−2.920043	0.633069
H	4.926698	1.273096	2.184722
H	2.387185	1.302245	3.967108
H	0.341472	−4.401741	1.490210
H	−1.003261	−4.313870	0.330383
H	−1.276312	−3.922671	2.051761
H	−4.764956	−3.155012	−0.549811
H	−5.048693	−2.767289	1.170995
H	−6.275556	−2.360166	−0.050938
H	0.566607	−1.338206	−1.705917
H	0.850427	0.274193	−2.325826
H	2.211560	−1.076810	−3.670640
H	4.715282	1.021581	−1.350808
H	4.813134	−0.703230	−1.035815
H	−4.243713	1.331370	−1.492488
H	−4.813676	1.397460	0.173204
H	−2.202984	2.615621	−1.929497
H	−0.906346	2.613016	0.828084
H	−0.161209	2.148599	−0.685529
H	3.206294	−2.541999	−1.167404
H	2.193118	−3.186787	−2.457085
H	3.866473	−2.730762	−2.802276
H	−1.815223	4.835052	0.141490
H	−2.578480	5.009176	−1.447593
H	−0.850402	4.639541	−1.331014

B3LYP Energy = −1305.77018048 a.u.

(a*R*,3*S*,3'*S*)-**19**, Conf A

C	2.657077	0.195518	−2.272480
C	1.309748	−0.007039	−2.023738
C	0.845595	−0.191956	−0.718828
C	1.759287	−0.177321	0.345833
C	3.119644	0.041126	0.091650
C	3.565125	0.215962	−1.215012
C	−0.618927	−0.391197	−0.488266
C	−1.166405	−1.680511	−0.529995
C	−2.530962	−1.894261	−0.321755
C	−3.356806	−0.802997	−0.067144
C	−2.838140	0.494766	−0.005081
C	−1.474462	0.693282	−0.228710
O	4.902734	0.409191	−1.446637
O	3.200713	0.386355	−3.524665
O	−0.289913	−2.700186	−0.776687
C	−0.781317	−4.035102	−0.841503
O	−4.704377	−0.909503	0.139288
C	−5.305994	−2.201124	0.127299
C	1.307881	−0.393295	1.774529
C	2.470885	−0.740316	2.701087
O	3.537637	0.196556	2.497742
C	4.130716	0.060956	1.215736
C	−3.765088	1.653495	0.284387
O	−3.075538	2.837957	0.657366
C	−2.025767	3.161264	−0.262078
C	−0.927441	2.104851	−0.172818
C	2.095113	−0.696456	4.169890
C	−1.533022	4.556176	0.073897
H	0.609282	−0.023241	−2.851855
H	−2.938504	−2.891048	−0.358847
H	5.032211	0.525381	−2.398362
H	2.515730	0.335606	−4.201300
H	−1.513203	−4.154519	−1.645336
H	−1.227621	−4.345125	0.107532
H	0.084753	−4.658669	−1.050195
H	−5.189690	−2.685928	−0.845775
H	−4.888840	−2.842304	0.908448
H	−6.363037	−2.037968	0.322789

H	0.816357	0.509363	2.155039
H	0.563514	−1.191602	1.813094
H	2.842602	−1.744570	2.448618
H	4.740245	−0.854942	1.181088
H	4.812323	0.904169	1.099147
H	−4.401544	1.856070	−0.590758
H	−4.429937	1.410555	1.113373
H	−2.445341	3.158068	−1.279480
H	−0.376877	2.248762	0.763415
H	−0.207061	2.258213	−0.978800
H	1.769473	0.307791	4.449555
H	1.279240	−1.392432	4.374799
H	2.947494	−0.969691	4.793584
H	−1.157827	4.589887	1.099052
H	−2.340669	5.283123	−0.022935
H	−0.723564	4.847182	−0.598538

B3LYP Energy = −1305.77405659 a.u.

(a*R*,3*S*,3’*S*)-**19**, Conf B

C	2.640388	0.247356	−2.282796
C	1.293260	0.041780	−2.025544
C	0.841740	−0.179852	−0.724447
C	1.758339	−0.195627	0.339187
C	3.116313	0.034158	0.082704
C	3.548482	0.232081	−1.225735
C	−0.621757	−0.384442	−0.489506
C	−1.167024	−1.673619	−0.546976
C	−2.531267	−1.891359	−0.341460
C	−3.358200	−0.804086	−0.073518
C	−2.840788	0.493266	0.005247
C	−1.477529	0.696208	−0.216627
O	4.869129	0.488185	−1.568396
O	3.067379	0.456561	−3.565250
O	−0.288616	−2.688821	−0.805879
C	−0.782320	−4.019933	−0.916002
O	−4.705775	−0.914276	0.131165
C	−5.307509	−2.205344	0.095717
C	1.309484	−0.443289	1.763416
C	2.475984	−0.788848	2.684749
O	3.532285	0.165529	2.492384
C	4.124806	0.054873	1.211397
C	−3.768897	1.647785	0.307542
O	−3.080207	2.828446	0.694154
C	−2.031543	3.163384	−0.222761
C	−0.931783	2.107628	−0.146239
C	2.102927	−0.767023	4.154357
C	−1.540466	4.555258	0.127743
H	0.596635	0.047810	−2.854460
H	−2.937908	−2.887894	−0.392248
H	5.474053	−0.065271	−1.060843
H	4.031362	0.548659	−3.542387
H	0.083817	−4.638658	−1.138439
H	−1.508613	−4.111675	−1.728345
H	−1.235938	−4.359096	0.019562
H	−5.189529	−2.673192	−0.885397
H	−4.891960	−2.860129	0.866432
H	−6.364892	−2.045436	0.292107
H	0.802470	0.444077	2.159106
H	0.578522	−1.254513	1.787377
H	2.861515	−1.784224	2.419102
H	4.751351	−0.852296	1.174254
H	4.789553	0.915271	1.108043
H	−4.404979	1.859588	−0.565619
H	−4.433914	1.395013	1.133484
H	−2.451770	3.170503	−1.239690
H	−0.379907	2.241899	0.790852
H	−0.213507	2.270616	−0.952082
H	1.766123	0.229861	4.446533
H	1.295071	−1.474479	4.351080
H	2.958918	−1.038724	4.773694
H	−1.164336	4.578694	1.152892
H	−2.349281	5.282012	0.039450
H	−0.732278	4.854464	−0.542577

B3LYP Energy = −1305.77154768 a.u.

(a*R*,3*S*,3’*S*)-**19**, Conf C

C	−2.679701	−0.000212	−2.330995
C	−1.331506	0.172440	−2.063716
C	−0.866780	0.224269	−0.747341
C	−1.779609	0.102179	0.311137
C	−3.140053	−0.087988	0.037093
C	−3.586931	−0.126323	−1.280282
C	0.599333	0.394331	−0.503923
C	1.134425	1.673338	−0.302595
C	2.502404	1.859338	−0.090653
C	3.343734	0.750301	−0.081224
C	2.842057	−0.538706	−0.292374
C	1.471054	−0.708252	−0.492570
O	−4.924841	−0.294853	−1.528991
O	−3.224895	−0.060776	−3.595257
O	0.242449	2.709244	−0.319018
C	0.720787	4.037790	−0.134425
O	4.692793	0.830034	0.127011
C	5.286121	2.110026	0.327474
C	−1.322606	0.155498	1.752976
C	−2.481976	0.382809	2.720172
O	−3.549627	−0.522821	2.409014
C	−4.147805	−0.232718	1.154951
C	3.791544	−1.713884	−0.264828
O	3.215669	−2.925663	−0.737585
C	1.900166	−3.190298	−0.223357
C	0.940081	−2.108050	−0.720766
C	−2.099084	0.159044	4.170738
C	1.886140	−3.376418	1.293218
H	−0.630902	0.271453	−2.885888
H	2.901883	2.848206	0.063508
H	−5.054241	−0.322324	−2.487403
H	−2.536128	0.021215	−4.264980
H	1.426203	4.322087	−0.920158
H	1.193920	4.161999	0.843734

H	−0.156300	4.678005	−0.191731
H	6.349619	1.926968	0.460058
H	4.888913	2.596918	1.222169
H	5.135902	2.758448	−0.539854
H	−0.819138	−0.780874	2.020851
H	−0.585348	0.951151	1.879764
H	−2.855601	1.410092	2.595430
H	−4.751051	0.684625	1.232037
H	−4.835246	−1.052024	0.942673
H	4.650572	−1.522062	−0.909092
H	4.186786	−1.850609	0.751345
H	1.626744	−4.139829	−0.686668
H	−0.033969	−2.225654	−0.242288
H	0.772717	−2.257169	−1.792085
H	−1.280411	0.823138	4.455116
H	−2.947385	0.355850	4.827845
H	−1.774832	−0.872626	4.323859
H	2.075877	−2.444071	1.828004
H	2.639494	−4.107818	1.591613
H	0.908759	−3.747489	1.608486

B3LYP Energy = −1305.77021957 a.u.

(a*R*,3*S*,3’*S*)-**19**, Conf D

C	2.637865	−0.105720	−2.202053
C	1.286232	−0.244740	−1.932846
C	0.828126	−0.349672	−0.617076
C	1.752194	−0.321321	0.438508
C	3.119095	−0.188928	0.162284
C	3.555644	−0.069012	−1.153862
C	−0.640317	−0.491609	−0.370101
C	−1.204141	−1.767630	−0.235349
C	−2.573925	−1.933619	−0.019322
C	−3.388285	−0.807746	0.066887
C	−2.852824	0.479792	−0.043986
C	−1.484037	0.628640	−0.276689
O	4.895926	0.076592	−1.403614
O	3.176236	0.008349	−3.465936
O	−0.339348	−2.821326	−0.330120
C	−0.845243	−4.146169	−0.200862
O	−4.740060	−0.869467	0.264215
C	−5.358641	−2.144482	0.414461
C	1.310394	−0.471385	1.877544
C	2.359876	0.035449	2.869125
O	3.637633	−0.536952	2.538363
C	4.144310	−0.140307	1.271375
C	−3.766844	1.678994	0.068017
O	−3.065536	2.894204	0.290606
C	−1.998486	3.077352	−0.647061
C	−0.917297	2.028114	−0.401348
C	2.411215	1.557869	2.989511
C	−1.489115	4.498153	−0.494875
H	0.576957	−0.274016	−2.753163
H	−2.995594	−2.920753	0.075101
H	5.023777	0.112023	−2.361984
H	2.488936	−0.083768	−4.135805
H	−1.569195	−4.377112	−0.987393
H	−1.305625	−4.305611	0.778177
H	0.015972	−4.802110	−0.302736
H	−5.229278	−2.757344	−0.481594
H	−4.964769	−2.678044	1.283551
H	−6.416989	−1.945200	0.563854
H	0.364776	0.049781	2.040754
H	1.118399	−1.529190	2.084364
H	2.140422	−0.377936	3.854984
H	4.968296	−0.820966	1.052308
H	4.576872	0.868209	1.328732
H	−4.384038	1.769406	−0.839130
H	−4.450060	1.560046	0.908921
H	−2.404278	2.942300	−1.661120
H	−0.375854	2.286151	0.516098
H	−0.184378	2.065048	−1.209360
H	3.227355	1.858626	3.649135
H	2.546777	2.047851	2.023666
H	1.475709	1.923198	3.418244
H	−1.126705	4.665227	0.521837
H	−2.284256	5.216136	−0.700784
H	−0.666821	4.684702	−1.188467

B3LYP Energy = −1305.76995217 a.u.

5. X-ray Crystallographic Study

5.1. General description

X-ray quality crystals could be grown from appropriate solvents by slow evaporation. A chosen crystal was then fixed under a microscope onto a Mitegen loop using high-density oil. Diffraction Intensity data was collected at ambient or low (150 K) temperature on a Bruker-D8 Venture diffractometer (Bruker AXS GmbH, Karlsruhe, Germany) equipped with INCOATEC IμS 3.0 (Incoatec GmbH, Geesthacht, Germany) dual (Cu and Mo) sealed tube micro sources and a Photon II Charge-Integrating Pixel Array detector (Bruker AXS GmbH, Karlsruhe, Germany) using Mo Kα (λ = 0.71073 Å) or Cu Kα (λ = 1.541 Å) radiation.

The low quality of the crystals caused a few A and B level errors but the structures are considered to be correct based on chemical evidences. The Flack parameter was meaningless in a few cases as Mo Kα radiation was used for light atom structures or quality of the crystal. Nevertheless, the stereoselective synthetic scheme enabled to assign the stereogenic elements based on the known (*S*) absolute configuration of C-3 and C-3'.

High-multiplicity data collection and integration were performed using APEX5 (version 2017.3-0, Bruker AXS Inc., 2017, Madison, WI, USA) software. Data reduction and multiscan absorption correction were performed using SAINT (version 8.38A, Bruker AXS Inc., 2017, Madison, WI, USA). The structure was solved using direct methods and refined on F^2 using the SHELXL program [7] incorporated into the APEX5 suite. Refinement was performed anisotropically for all non-hydrogen atoms. Hydrogen atoms were placed in idealized positions on parent atoms in the final refinement except O-H protons in which could be found at the difference electron density map and the respective O-H distances were constrained. Nevertheless the orientation of the –OH groups is not well-defined also resulting crystallographic errors but this only means that the description of the hydrogen bond network is ambiguous.

The CIF file was manually merged using publCIF software [8], while graphics were designed using the Mercury program. [9] The results for the X-ray diffraction structure determinations followed the Checkcif functionality of PLATON software (Utrecht University, Utrecht, the Netherlands). [10]

Table S3. Experimental details of structure determination.

Compound	*cis,cis*-(a*S*,1*R*,3*S*,1'*R*,3'*S*)-**20**	*cis,cis*-(a*S*,1*R*,3*S*,1'*R*,3'*S*)-**21**	*cis,trans*-(a*S*,1*R*,3*S*,1'*S*,3'*S*)-**22**	*cis*-(a*S*,2*S*,1'*R*,3'*S*)-**24**
Crystal data				
Chemical formula	$C_{34}H_{32}F_2O_6$	$C_{34}H_{32}Br_2O_6$	$C_{40}H_{46}O_{12}.CHCl_3$	$C_{27}H_{29}FO_6.C_3H_6O$
M_r	574.59	696.41	838.13	526.58
Crystal system, space group	Monoclinic, $P2_1$	Monoclinic, $P2_1$	Orthorhombic, $P2_12_12_1$	Orthorhombic, $P2_12_12_1$
Temperature (K)	150	295	293	298
a, *b*, *c* (Å)	18.5265 (8), 8.5588 (4), 19.9910 (9)	12.0924 (3), 8.1811 (2), 15.9192 (4)	11.7006 (7), 11.9715 (6), 29.8020 (18)	9.4114 (5), 15.3373 (9), 20.6186 (11)
α, β, γ (°)	90, 116.251 (2), 90	90, 93.088 (2), 90	90, 90, 90	90, 90, 90
V (Å^3)	2842.9 (2)	1572.59 (7)	4174.5 (4)	2976.2 (3)
Z	4	2	4	4
Radiation type	Mo *K*α	Cu *K*α	Mo *K*α	Mo *K*α
μ (mm^{-1})	0.10	3.63	0.28	0.09
Crystal size (mm)	0.28 × 0.19 × 0.07	0.53 × 0.23 × 0.13	0.26 × 0.15 × 0.13	0.42 × 0.20 × 0.12
Data collection				
Diffractometer	Bruker D8 VENTURE			
Absorption correction	Multi-scan *SADABS2016*/2 - Bruker AXS area detector scaling and absorption correction			
T_{min}, T_{max}	0.97, 0.99	0.26, 0.66	0.90, 0.96	0.95, 0.99
No. of measured, independent and observed [$I > 2\sigma(I)$] reflections	104411, 10467, 8528	28809, 5820, 4466	56340, 7647, 5316	27939, 5661, 4670
R_{int}	0.094	0.121	0.088	0.044
$(\sin \theta/\lambda)_{max}$ (Å^{-1})	0.604	0.610	0.603	0.611
Refinement				
$R[F^2 > 2\sigma(F^2)]$, $wR(F^2)$, S	0.058, 0.156, 1.13	0.127, 0.316, 1.20	0.059, 0.160, 1.04	0.075, 0.232, 1.06
No. of reflections	10467	5820	7647	5661
No. of parameters	778	387	519	348
No. of restraints	5	347	1	7
$\Delta\rangle_{max}$, $\Delta\rangle_{min}$ (e Å^{-3})	0.41, -0.35	2.37, -1.20	0.39, -0.33	0.89, -0.40
Absolute structure parameter	0.3 (4)	0.18 (2)	0.01 (4)	-0.5 (3)

Table S4. Experimental details of structure determination.

Compound	*cis*,*cis*-(a*S*,1*R*,3*S*,1'*R*,3'*S*)-**26**	*cis*,*cis*-(a*S*,1*R*,3*S*,1'*R*,3'*S*)-**27**
Crystal data		
Chemical formula	$C_{34}H_{30}F_2O_6.2(C_3H_6O)$	$C_{34}H_{30}Br_2O_6$
M_r	688.73	694.40
Crystal system, space group	Monoclinic, $P2_1$	Orthorhombic, $P2_12_12_1$
Temperature (K)	293	295
a, b, c (Å)	8.8912 (6), 9.2003 (5), 22.8734 (15)	7.7409 (6), 19.3762 (18), 21.767 (2)
α, β, γ (°)	90, 99.924 (2), 90	90, 90, 90
V (Å^3)	1843.1 (2)	3264.8 (5)
Z	2	4
Radiation type	Mo $K\alpha$	Mo $K\alpha$
μ (mm^{-1})	0.09	2.52
Crystal size (mm)	0.63 × 0.44 × 0.09	0.33 × 0.10 × 0.07
Data collection		
Diffractometer	Bruker D8 VENTURE	
Absorption correction	Multi-scan *SADABS2016*/2 - Bruker AXS area detector scaling and absorption correction	
T_{min}, T_{max}	0.75, 0.99	0.49, 0.84
No. of measured, independent and observed [$I > 2\sigma(I)$] reflections	35758, 7004, 4669	41908, 5970, 3604
R_{int}	0.097	0.100
$(\sin \theta/\lambda)_{max}$ (Å^{-1})	0.611	0.603
Refinement		
$R[F^2 > 2\sigma(F^2)]$, $wR(F^2)$, S	0.059, 0.156, 1.06	0.053, 0.158, 1.02
No. of reflections	7004	5970
No. of parameters	460	384
No. of restraints	1	0
$\Delta\rangle_{max}$, $\Delta\rangle_{min}$ (e Å^{-3})	0.31, -0.18	0.84, -0.58
Absolute structure parameter	0.3 (6)	0.007 (7)

5.2. Molecular Structure Analysis

The single crystal X-ray diffraction analysis proved the absolute configuration of the the central and axial chirality elements unambiguously. In all cases, the space group is allowed for chiral compounds, as it is expected. In structure of *cis*,*cis*-(a*S*,1*R*,3*S*,1'*R*,3'*S*)-**20**, there are two molecules in the asymmetric unit with slightly different conformation (Figure S206). The main difference is the orientation of the 4-fluorophenyl rings with respect to the isochromane moieties i.e. rotation around the C1-C10 and C1'-C12' bonds. Other differences include the orientation of the O8' methoxy group and small conformational change of the O2 isochromane ring.

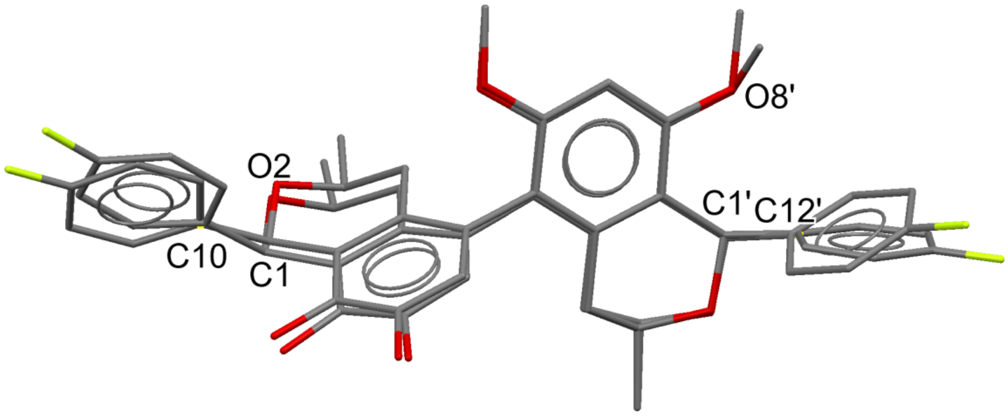

Figure S206. Overlapped conformers in the asymmetric unit of *cis*,*cis*-(a*S*,1*R*,3*S*,1'*R*,3'*S*)-**20** showing conformational differences. Hydrogen atoms are omitted for clarity.

In the structure of *cis*,*trans*-(a*S*,1*R*,3*S*,1'*S*,3'*S*)-**22**, there is a chloroform, in that of *cis*-(a*S*,2*S*,1'*R*,3'*S*)-**24** a disordered acetone and in that of *cis*,*cis*-(a*S*,1*R*,3*S*,1'*R*,3'*S*)-**26** two well defined acetone solvent molecules. The disorder in the structure *cis*-(a*S*,2*S*,1'*R*,3'*S*)-**24** resulted in several errors, but the structure of the *bis*-isochroman, hence the assignment of absolute configuration is unambiguous. The solid-state structures are stabilized by hydrogen bonds between C-H or O-H protons and O, F or Br as acceptors.

6. References

1. MacroModel; Schrödinger LLC, 2015. Available online: https://newsite.schrodinger.com/platform/products/macromodel (accessed on 12 July 2024).
2. Frisch, M. J.; Trucks, G. W.; Schlegel, H. B.; Scuseria, G. E.; Robb, M. A.; Cheeseman, J. R.; Scalmani, V.; Barone, G.; Mennucci, B.; Petersson, G. A., et al. *Gaussian 09 Revisions C.01*; Gaussian, Inc.: Wallingford, CT, USA, 2010.
3. Frisch, M. J.; Trucks, G. W.; Schlegel, H. B.; Scuseria, G. E.; Robb, M. A.; Cheeseman, J. R.; Scalmani, V.; Barone, G.; Mennucci, B.; Petersson, G. A., et al. *Gaussian 09 Revisions E.01*; Gaussian, Inc.: Wallingford, CT, USA, 2013.
4. Chai, J.-D.; Head-Gordon, M. Systematic optimization of long-range corrected hybrid density functionals. *J. Chem. Phys.*, **2008**, *128*, 084106.
5. Stephens, P. J.; Harada, N. ECD cotton effect approximated by the Gaussian curve and other methods. *Chirality*, **2010**, *22*, 229–233.
6. Varetto, U. *MOLEKEL 5.4*; Swiss National Supercomputing Centre: Manno, Switzerland, 2009.
7. Sheldrick, G. A short history of SHELX. *Acta Crystallogr., Sect. A: Found. Adv.*, **2008**, *64*, 112–122.
8. Westrip, S. publCIF: software for editing, validating and formatting crystallographic information files. *J. Appl. Crystallogr.*, **2010**, *43*, 920–925.
9. Macrae, C. F.; Sovago, I.; Cottrell, S. J.; Galek, P. T. A.; McCabe, P.; Pidcock, E.; Platings, M.; Shields, G. P.; Stevens, J. S.; Towler, M., et al. Mercury 4.0: from visualization to analysis, design and prediction. *J. Appl. Crystallogr.*, **2020**, *53*, 226–235.
10. Spek, A. Single-crystal structure validation with the program PLATON. *J. Appl. Crystallogr.*, **2003**, *36*, 7–13.
